# The Study of Yin-Chen-Hao-Tang Preventing and Treating Alcoholic Fatty Liver Disease through PPAR Signaling Pathway Based on Network Pharmacology and RNA-Seq Transcriptomics

**DOI:** 10.1155/2021/8917993

**Published:** 2021-12-31

**Authors:** Yi-Wei Zhu, Du Li, Ting-Jie Ye, Feng-Jun Qiu, Xiao-Ling Wang, Xiao-Feng Yan, Yan-Lin Lu, Wei Xu, Hua Li, Xu-Dong Hu

**Affiliations:** Department of Biology, School of Basic Medical Science, Shanghai University of Traditional Chinese Medicine, Shanghai 201203, China

## Abstract

**Background:**

Alcoholic fatty liver disease (AFLD) is the first stage of the alcoholic liver disease course. Yin-Chen-Hao-Tang (YCHT) has a good clinical effect on the treatment of AFLD, but its molecular mechanism has not been elucidated. In this study, we tried to explore the molecular mechanism of YCHT in improving hepatocyte steatosis in AFLD mice through network pharmacology and RNA sequencing (RNA-Seq) transcriptomics.

**Methods:**

Network pharmacological methods were used to analyze the potential therapeutic signaling pathways and targets of YCHT on AFLD. Then, the AFLD mice model was induced and YCHT was administered concurrently. Liver injury was measured by serum alanine aminotransferase (ALT) activity and liver tissue H&E staining, and liver steatosis was determined by serum triglyceride (TG) level and liver tissue Oil Red staining. The molecular mechanism of YCHT on prevention and treatment of mice AFLD was investigated according to the Kyoto Encyclopedia of Genes and Genomes (KEGG) pathway enrichment analysis of the differential expression genes data obtained by liver tissue RNA-Seq. Finally, ethanol-induced AFLD AML12 hepatocyte model was established, YCHT with or without PPAR*α* agonist pemafibrate or PPAR*γ* inhibitor GW9662 was administered, Nile Red fluorescent staining was used to evaluate steatosis levels in AML12 hepatocytes, and qRT-PCR was used to detect PPAR*α* and PPAR*γ* gene expression.

**Results:**

The results of network pharmacology analysis showed that YCHT may exert its pharmacological effect on AFLD through 312 potential targets which are involved in many signaling pathways including the PPAR signaling pathway. AFLD mice experiments results showed that YCHT markedly decreased mice serum ALT activity and serum TG levels. YCHT also significantly improved alcohol-induced hepatic injury and steatosis in mice livers. Furthermore, KEGG pathway enrichment results of RNA-Seq showed that the PPAR signaling pathway should be the most relevant pathway of YCHT in the prevention and treatment of AFLD. AFLD hepatocyte model experiment results showed that YCHT could remarkably reduce hepatocyte steatosis through reducing PPAR*γ* expression and increasing PPAR*α* expression.

**Conclusions:**

Our study discovered that PPAR*γ* and PPAR*α* are the key targets and the PPAR signaling pathway is the main signaling pathway for YCHT to prevent and treat AFLD.

## 1. Introduction

Globally, in 2010, the number of deaths from alcohol-attributable liver cirrhosis was 493,300, and alcoholic cirrhosis deaths accounted for 47.9% of all liver cirrhosis deaths [1]. In developed regions such as North America and Europe, alcoholic liver disease is the leading cause of cirrhosis. In China, with the improvement of living standards and the change of lifestyle, the proportion of alcoholics in the population has increased year by year. The percentage of regular alcohol drinkers among the general adult population in different areas increased from 27.0% in 2000 to 66.2% in 2015. The 2.27% alcoholic liver disease (ALD) prevalence in 2000 was increased to 8.74% in 2015 [2]. Alcoholic fatty liver disease (AFLD) is the first response of the liver to alcohol abuse, and it is the first stage in the progression of ALD; it can progress to steatohepatitis, fibrosis, and cirrhosis, leading to an increased probability of hepatic failure and death [3]. The prevention and treatment of AFLD are beneficial for blocking the progression of alcoholic liver disease. However, there is currently no specific drug for the therapy of AFLD.

The traditional Chinese medicine treatment of AFLD has achieved a good therapeutic effect. The classical prescription Yin-Chen-Hao-Tang (YCHT) has been used for the treatment of AFLD in clinic, and its curative effect is excellent [4, 5]. YCHT consists of Artemisiae Scopariae Herba, Gardeniae Fructus, and Radix Rhei Et Rhizome. Studies showed that YCHT can effectively alleviate hepatic fibrosis and steatosis [6]. YCHT could also improve AFLD in rats due to its effect on antialcoholic hepatotoxicity and reducing liver fat deposition [7], and it could protect rats [8] and mice [9] from acute alcoholic liver injury by scavenging oxygen free radicals and reducing lipid peroxidation. But the molecular mechanism underlying was still unknown.

In this study, we tried to elucidate the key signal pathways and main targets of YCHT in the prevention and treatment of AFLD through dry experiments (network pharmacological analysis) and wet experiments (AFLD mouse model and AFLD mouse hepatocyte model experiments).

## 2. Materials and Methods

### 2.1. Network Pharmacological Analysis of Therapeutic Pathways and Targets of Yin-Chen-Hao-Tang (YCHT) in the Prevention and Treatment of Alcoholic Fatty Liver Disease (AFLD)

Firstly, the effective components and potential pharmacological targets of Artemisiae Scopariae Herba, Gardeniae Fructus, and Radix Rhei Et Rhizome were obtained by TCMSP (https://tcmsp-e.com/) online database. Then, the total target set of YCHT was obtained by combining the target sets of these three herbs. Secondly, we got the disease-related target set of alcoholic fatty liver disease (AFLD) through searching for the keyword “alcoholic fatty liver disease” in GeneCards (https://www.genecards.org) online database. Thirdly, the intersection of the above two sets was collected to obtain the potential therapeutic target set of FZHY on AFLD through employing the Venny2.1 online tool (https://bioinfogp.cnb.csic.es/tools/venny/index.html). Fourthly, PPI analysis was carried out by the input of the potential therapeutic target set into STRING (https://www.string-db.org/) online database, and KEGG enrichment analysis was carried out by the input of the potential therapeutic target set into DAVID (https://david.ncifcrf.gov/) online database. Finally, the potential therapeutic molecular mechanism and key therapeutic targets of FZHY on AFLD were obtained.

### 2.2. Materials

The following reagents and instruments were used: absolute ethanol (Sinopharm Chemical Reagent Co., Ltd.), High Sensitivity DNA Kit (Agilent p/n 5067–4626), SuperScript II Reverse Transcriptase with 100 mM DTT and 5X First-Strand Buffer (Invitrogen, 18064014), TruSeq Stranded Total RNA LT Sample Prep Kit 48 Samples (Illumina, 15032612), TruSeq Stranded mRNA LT Sample Prep Kit (Illumina, 15032612), Ribo-Zero rRNA Removal Kits (Epicentre, MRZMB126), Agencourt AMPure XP Kit (Beckman p/n A63881), Qubit dsDNA HS Assay Kit (Invitrogen, p/n Q32850), Trizol (Takara), alanine aminotransferase (ALT/GPT) test kit (Nanjing Jiancheng Bioengineering Institute, C009-2), triglyceride (TG) test kit (Nanjing Jiancheng Bioengineering Institute, A110-1), Reverse Transcription Kit (Thermo Scientific), qPCR Kit (TaKaRa), SDS-PAGE gel rapid preparation kit (Biotime Biotechnology), PPAR*α* agonist pemafibrate (MedChemExpress), and PPAR*γ* inhibitor GW9662 (MedChemExpress).

### 2.3. Instruments

Magnetic stand (Invitrogen, k1585-01), 96-well plate (Thermo Fisher, AB0859), MicroCentrifuge (Eppendorf, 5418), centrifuge (Eppendorf, 5424), heat block (BIOER, HB-100), Synergy 2 Microplate Reader (Bio-Tek, Vermont), real-time PCR system (Roche, LightCycler96), and gradient PCR instrument gene amplification instrument (Eppendorf, Mastercycler Nexus Gradient) were used.

### 2.4. Granules

Artemisiae Scopariae Herba granules (Lot no. 18086954), Gardeniae Fructus granules (Lot NO.: 18076664), and Radix Rhei Et Rhizome granules (Lot no. 18096814) were from Jiangyin Tianjiang Pharmaceutical Co., Ltd. In vivo, YCHT recipe stock solution was made by dissolving Artemisiae Scopariae Herba granules 80 mg, Gardeniae Fructus granules 40 mg, and Radix Rhei Et Rhizome granules 66 mg in 2 mL normal saline. It was given once daily by gavage at 0.1 mL/10 g. This dose of the YCHT recipe was calculated according to the clinical adult dose. In vitro, 80 mg of Artemisiae Scopariae Herba granules, 40 mg of Gardeniae Fructus granules, and 66 mg of Radix Rhei Et Rhizome granules were dissolved in 4 mL of DMEM/F-12 culture medium, filtered by 0.45 mm filters, then divided into EP tube per 35 *μ*L, and stored at −20°C.

### 2.5. Grouping, AFLD Model Establishment, and Drug Administration

Twenty-four female C57BL/6 mice with an average weight of 20–22 g, purchased from the Experimental Animal Center of the Chinese Academy of Sciences, were raised in the SPF-level breeding room of the Experimental Animal Center of Shanghai University of Traditional Chinese Medicine. Control Lieber–DeCarli liquid diet (TP4030C) and ethanol Lieber–DeCarli liquid diet (TP4030D) were purchased from Trophic Animal Feed High-tech Co., Ltd., Nantong, Jiangsu Province, China. The caloric profile of the diet was shown in Table 1. All mice were randomly divided into three groups (8 mice per group) and were adaptively fed for 5 days with control Lieber–DeCarli liquid diet (TP4030C) which was ethanol-free. After that, mice in the control group were fed with control Lieber–DeCarli liquid diet (TP4030C) daily for 10 days. Mice in the ethanol group were fed with ethanol Lieber–DeCarli liquid diet (TP4030D) containing 5% ethanol daily for 10 days to induce alcoholic fatty liver disease (AFLD). Mice in the YCHT group were fed with ethanol Lieber–DeCarli liquid diet (TP4030D) containing 5% ethanol daily and administered with YCHT recipe stock solution at 0.1 mL/10 g body weight by gavage once a day for 10 days. At the same time, mice in the control and ethanol groups were given normal saline at 0.1 mL/10 g body weight by gavage once a day for 10 days. In the morning of the 11th day, mice in the YCHT and ethanol groups were given a binge dose of 31.5% vol/vol ethanol at 0.2 mL/10 g body weight by gavage after 1 h of the last drug or normal saline administration, and mice in control group were given isocaloric 45% wt/vol maltose dextrin at 0.2 mL/10 g body weight by gavage. 9 h after gavage, all mice were sacrificed. The serums and liver tissues were collected for the following study.

### 2.6. Serum ALT and TG Measurement

Test operation according to the operating instructions of the kits.

### 2.7. H&E Staining

Firstly, the paraffin sections of mice liver tissues were baked in an oven at 60°C for 1 to 2 hours and dewaxed with xylene and ethanol. Secondly, hematoxylin was dyed for 10 minutes and washed with running water to remove excess hematoxylin. Thirdly, we put the slices in 0.7% hydrochloric acid ethanol for a few seconds and rinsed with running water, and the slices turned blue for about 15 minutes. Fourthly, the slices were placed in 95% ethanol for 30 seconds and stained with eosin alcohol for 30 seconds. Fifthly, the slices were placed twice in 95% ethanol for 30 seconds and twice in 100% ethanol for 30 seconds. Finally, the slices were placed in xylene for 30 seconds three times and sealed with neutral gum.

### 2.8. Oil Red Staining

Frozen sections of mice liver tissues were washed with 75% ethanol and stained with Oil Red for 10 minutes. The slices were then placed in 75% ethanol for a few seconds and then washed with pure water. The sections were stained with hematoxylin for 3 minutes and washed with running water. Then, the sections were differentiated with hydrochloric acid and ethanol and rinsed with running water. Finally, glycerol gelatin was used to seal the film.

### 2.9. RNA-Seq

The total RNA from mice liver tissues of control/ethanol/YCHT groups was extracted by using RNAiso Plus Total RNA extraction reagent and RNeasy Mini Kit according to the standard operating procedures provided by the company. After the Agilent Bioanalyzer 2100 electrophoresis was passed, the total RNA was purified by using the RNAClean XP Kit and the RNase-Free DNase Set. Then, the purified Total RNA was subjected to mRNA isolation, fragmentation, first-strand cDNA synthesis, second-strand cDNA synthesis, terminal repair, 3′ end plus A, ligation, and enrichment, to complete the construction of the sequencing sample library. The library concentration was tested by using a Qubit® 2.0 Fluorometer and the library size was detected by using Agilent 2100. According to the corresponding procedure of the cBot User Guide, the cluster generation and the first-stage sequencing primer hybridization were performed by using the cBot of the Illumina sequencer. The sequencing reagents were provided according to the Illumina User Guide, and the flow cell with the cluster was loaded onto the machine and the paired-end program was used for double-end sequencing. The sequencing was controlled by the data collection software provided by Illumina and real-time data analysis was performed. The library construction and sequencing were performed at Shanghai Biotechnology Corporation, Shanghai, China.

### 2.10. Data Processing of Transcriptome Sequencing

There are a lot of useless data in the raw reads, such as unqualified data with low overall quality, sequencing primers, and low-end quality. These unqualified data will impact subsequent data analysis and need to be filtered and removed. Sertk screening was used to remove unqualified data. The linker sequence, the 3′ end mass *Q* below 20 bases, the length less than 25 reads, and ribosome RNA data from the species were all removed. Further analysis was performed by using the screened and removed reads.

### 2.11. Screening of Differential Expression Genes

The reads are not only proportional to the gene expression level but also related to the length of the gene itself and the amount of data that were sequenced. To obtain comparable data on the gene expression levels of different genes and different samples, the reads were converted into FPKM (fragments per kilobase of exon model per million mapped reads) to standardize the gene expression by three steps which were Stringtie count, TMM (trimmed mean of *M* values) normalization, and Perl script calculation. Using edgeR to perform differential gene analysis between samples, the *p* value was obtained, and a multihypothesis test was performed. The threshold of the *p*-value was determined by controlling the FDR (false discovery rate). The corrected *p* value was the *q* value. At the same time, the differential expression multiple was calculated basing on the FPKM value, which was fold change (FC). The screening conditions were *q* ≤ 0.05, FC ≥ 2, or FC ≤ 0.5.

### 2.12. qRT-PCR

Total RNA extraction and reverse transcription were performed according to the manufacturer's instructions of the kit. The ratio of RNA A260/280 extracted from liver tissue was 1.9–2.0, indicating that the RNA purity was high. Primers designed from the conserved regions of each cDNA were used for quantitative PCR (qPCR) analysis. All primer sequences were listed in Table 2. GAPDH primers were used as the internal control. SYBR green dye was used for qPCR (RR420A, Takara). The relative mRNA expression levels of target genes were calculated by the 2-ΔΔCt method. The amplification parameters were preheating at 95°C for 10 seconds, cycling at 95°C for 5 seconds, and 60°C for 30 seconds for 40 cycles.

### 2.13. Cell Grouping and Administration

A total of 5 × 10^4^ AML12 hepatocytes per well were grown in 96-well plates, were stimulated by 50 mM alcohol, and cocultured with 11, 33, 100, 300, and 900 *μ*g/mL YCHT for 12h. CCK-8 was used to detect the cytotoxicity of YCHT. The fluorescent staining of Nile Red was used to determine the lipid droplets in cells. From the above experiments, we determined that 300 *μ*g/mL was the optimal concentration for YCHT to reduce hepatocyte steatosis.

Then, a total of 1 × 10^6^ AML12 hepatocytes per well were grown in 6-well plates, stimulated by 50 mM alcohol, and cocultured with 1 *μ*M GW9662 (PPAR*γ* inhibitor) and with or without 300 *μ*g/mL YCHT for 12vh. 50 mM alcohol-stimulated AML12 hepatocytes were cocultured with 1 nM pemafibrate (PPAR*α* agonist) and with or without 300 *μ*g/mL YCHT for 3 h. The fluorescent staining of Nile Red was used to determine the lipid droplets in cells. Total RNA was extracted from cells for qRT-PCR.

### 2.14. Nile Red Staining

Firstly, after AML12 cells were incubated in 96-well plates for 12 hours, the supernatant was discarded, and the cells were washed with 200 *μ*L PBS three times. Secondly, 200 *μ*L 10% formalin was added and the cells were fixed for 2 minutes. Thirdly, formalin was removed. After washing with 200 *μ*L PBS three times, the lipid droplets in cells were stained with 1 *μ*g/mL Nile Red at 37°C for 10 minutes. Fourthly, Nile Red was discarded and the cells were washed with 200 *μ*L PBS three times. Finally, with adding 100 *μ*L PBS, the cells were observed and photographed under a fluorescence microscope, and the data were analyzed by ImageJ software. Relative Fluorescence Units (RFU) = total fluorescence staining intensity of all cells ÷ total fluorescence staining area of all cells.

### 2.15. Statistics Analysis

Data were presented as means ± SD. Statistical significance was determined by one-way analysis of variance (ANOVA) followed by Bonferroni's multiple comparisons test by using GraphPad Prism 9.0 software.

## 3. Results

### 3.1. PPAR Signaling Pathway Is One of the Important Pathways of Yin-Chen-Hao-Tang (YCHT) in the Treatment of Alcoholic Fatty Liver Disease (AFLD)

To explore the potential mechanism and target of YCHT in the prevention and treatment of AFLD, we carried out the network pharmacological analysis.

Through searching the TCMSP database, 41 active ingredients were obtained from Artemisiae Scopariae Herba, 68 active ingredients were obtained from Gardeniae Fructus, and 46 active ingredients were obtained from Radix Rhei Et Rhizome (please refer to the data of column “Mol ID” or “Molecule Name” in Supplementary Materials 1-1, 1-2, and 1-3, respectively, which were the raw data retrieved from the TCMSP database). Finally, 385 potential pharmacological targets of YCHT were obtained by statistical analysis of the corresponding targets of these active components (please refer to the sum data of column “Target Name” from Supplementary Materials 1-1, 1-2, and 1-3, which were the raw data retrieved from the TCMSP database).

**Figure 1:**
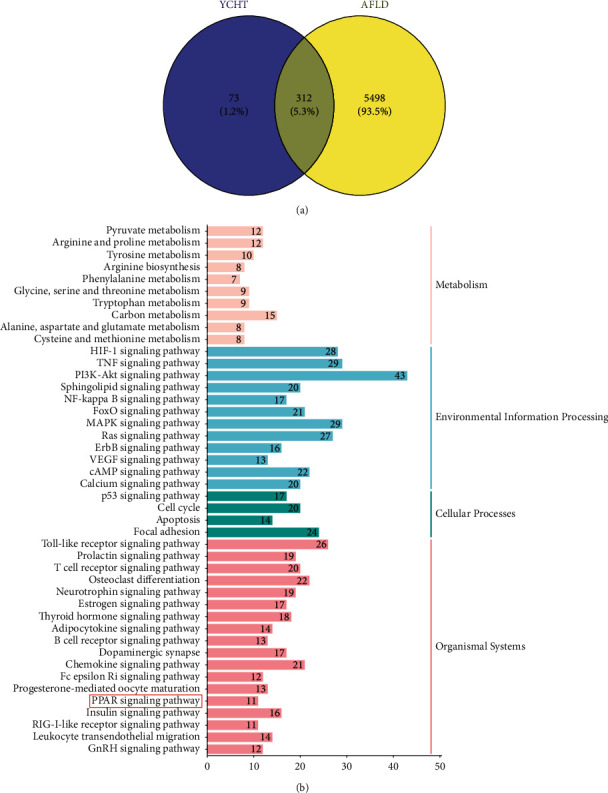
Network pharmacological analysis of Yin-Chen-Hao-Tang (YCHT) in the prevention and treatment of alcoholic fatty liver disease (AFLD). (a) Venn diagram of the mapping the target proteins set of YCHT and the disease genes set of AFLD. (b) KEGG pathway enrichment analysis of 312 potential therapeutic target proteins of YCHT on AFLD; the pathway enrichment results include PPAR signaling pathway (marked with a red square line). The bars show the number of genes in KEGG pathway terms, term *p* value < 0.001.

Through searching the GeneCards database, 5810 alcoholic fatty liver disease-related targets were obtained (please refer to the data of column “Gene Symbol” in Supplementary Material 2, which was the raw data retrieved from the GeneCards database). After intersecting 385 potential pharmacological targets of YCHT and 5810 alcoholic fatty liver disease-related targets, 312 potential therapeutic targets of YCHT on AFLD were obtained (Figure 1(a)).

The protein-protein interaction (PPI) analysis of 312 potential therapeutic targets was carried out by using the STRING database, and then the PPI data were imported into the software Cytoscape 3.8.0 for further degree value analysis. According to the degree value, the top 30 genes were INS, AKT1, IL6, TP53, CASP3, JUN, EGFR, MYC, STAT3, SRC, MAPK1, MAPK8, MMP9, PTGS2, CXCL8, IL1B, FOS, ESR1, TLR4, CCL2, CCND1, IL10, HSP90AA1, MMP2, CREB1, PPARG, ICAM1, IL4, NOS3, and RELA (please refer to the data of column “Degree” and corresponding column “Name” in Supplementary Material 3, which were the processing results of the Cytoscape-3.8.0 software). Among them, many inflammation-related genes such as IL6, IL1B, TLR4, CCL2, IL10, and IL4 were included, and also many lipids metabolism-related genes, such as STAT3, PPARG, and RELA, were included.

The KEGG enrichment analysis of 312 potential therapeutic targets was carried out by using DAVID online database. Inflammation-related pathways, such as the TNF signaling pathway, NF-kappa B signaling pathway, and Toll-like receptor signaling pathway, were obtained, and lipid metabolism-related pathways, such as the adipocytokine signaling pathway and PPAR signaling pathway, were obtained (Figure 1(b)).

### 3.2. YCHT Improved Ethanol-Induced Liver Injury and Steatosis in Mice

**Figure 2:**
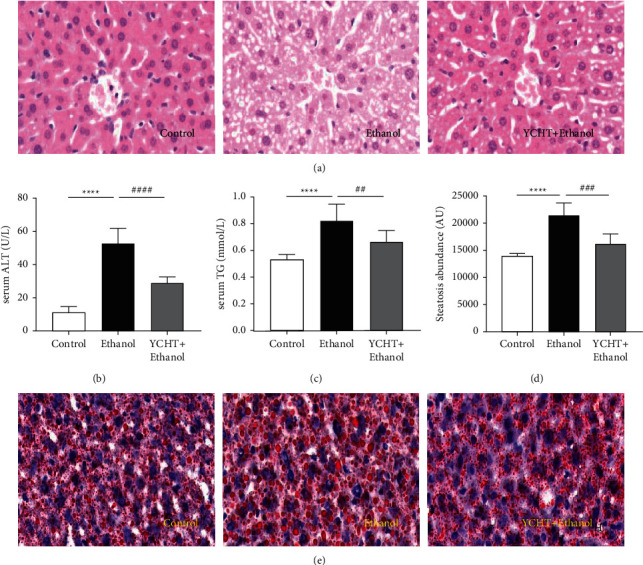
YCHT improved ethanol-induced liver injury and steatosis in mice. (a) Liver hematoxylin and eosin (HE) staining (×200). (b) Serum ALT levels. (c) Serum TG levels. (d) Semiquantitative analysis of Oil Red staining. (e) Liver Oil Red staining (×200). Results were presented as means ± SD (*n* = 8 mice/group).

To further illuminate the main targets and signaling pathways of YCHT in the prevention and treatment of AFLD, we established the mouse AFLD model and intervened with YCHT. Serum ALT and HE staining were used to evaluate the level of liver injury, and serum TG and Oil Red staining were used to measure the level of liver steatosis. As shown in Figures 2(b) and 2(c), serum ALT and TG in ethanol-fed mice were markedly increased after ten days of ethanol feedings (*p* < 0.001, *p* < 0.001). The livers in ethanol-fed mice exhibited widespread diffuse vesicular steatosis (Figure 2(a), HE staining), a large number of orange-red fat droplets in the cytoplasm (Figures 2(e) and 2(d), Oil Red staining and semi-quantitative), swelling hepatocytes, scattered hepatocyte apoptosis, blurred hepatocyte boundaries, disordered arrangement of hepatic cord, and narrowed hepatic sinusoidal space (Figure 2(a), HE staining). As expected, compared to ethanol-fed mice, serum ALT and TG in the YCHT-administrated mice were significantly decreased (*p* < 0.01, *p* < 0.01), and the ethanol-induced liver injury and steatosis were obviously improved in the YCHT-administrated mice (Figures 2(a) and 2(b)).

Data in Figures 2(b)–2(d) were all counted by one-way ANOVA followed by Bonferroni's multiple comparisons test to determine the statistical difference between two groups. Ethanol group versus control group, ^*∗∗∗∗*^*p* < 0.0001; YCHT group versus ethanol group, ^##^*p* < 0.01,  ^###^*p* < 0.001,  ^####^*p* < 0.0001.

### 3.3. Screening the Genes Differentially Expressed in Both YCHT Group (versus Ethanol Group) and Ethanol Group (versus Control Group) Based on RNA-Seq Transcriptomics

**Figure 3:**
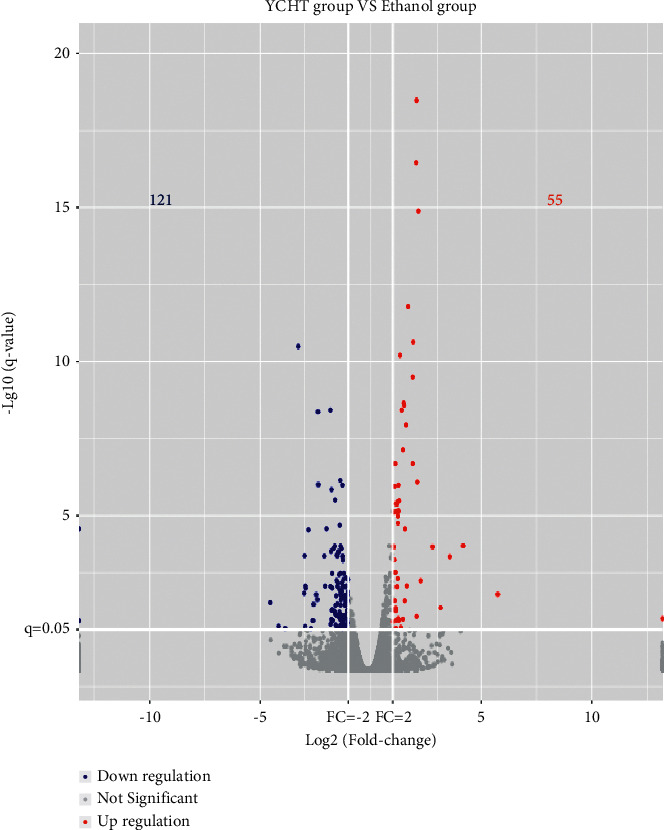
Volcano plot of differential expression genes of YCHT group versus ethanol group. Blue plots represent downregulated genes and red plots represent upregulated genes. FC: fold change.

Sifting with *q* value ≤0.05, FC ≥ 2, or FC ≤ 0.5, 1175 differential expression genes were obtained from 17226 genes in ethanol-treated mice compared with control mice, in which 817 genes were upregulated significantly and 358 genes were downregulated significantly (data were shown in the article to be published). 176 differential expression genes were obtained from 17517 genes in YCHT-administered mice compared with ethanol-treated mice, in which 55 genes were upregulated significantly and 121 genes were downregulated significantly (Figure 3). To elucidate the improvement mechanism of YCHT on alcohol-induced liver steatosis and injury, further differential genes screening had been carried out. The set of 176 differential expression genes from YCHT-administered mice compared with ethanol-treated mice was intersected with the set of 1175 differential expression genes from ethanol-treated mice compared with control mice. The screening results showed that the expression of 79 genes was upregulated by ethanol and reversed by YCHT (Table 3). At the same time, the expression of 42 genes was downregulated by ethanol and reversed by YCHT (Table 4). The results suggest that these genes may be related to the effect of YCHT on the alleviation of hepatic steatosis and liver injury.

### 3.4. KEGG Signaling Pathway Enrichment of the Differential Expression Genes

**Figure 4:**
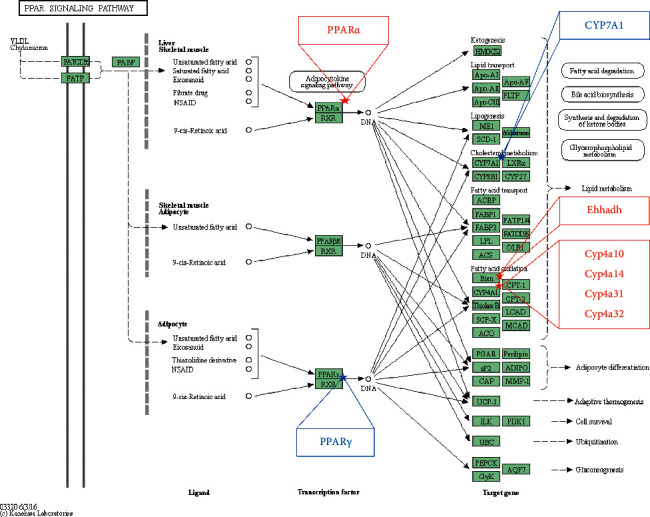
The enriched genes in The PPAR signaling pathway. Genes in the blue frame represent downregulated genes in the YHCT group compared with the ethanol group, and genes in the red frame represent upregulated genes in the YHCT group compared with the ethanol group.

To discover the key signaling pathway of YCHT in reducing hepatic steatosis and injury, the differential expressed genes in Tables 3 and 4 were further subjected to the DAVID online database for the KEEG signaling pathway enrichment. Enrichment results showed that the “PPAR signaling pathway” (*p* < 0.000018) was the most relevant pathway to the pharmacological effect of YCHT (Table 5). Seven genes, which were PPAR*γ*, CYP7A1, Ehhadh, Cyp4a10, Cyp4a14, Cyp4a31, and Cyp4a32, were found to be distributed in the PPAR signaling pathway (Figure 4).

The gene expression of peroxisome proliferator-activated receptor *γ* (PPAR*γ*), a transcription factor of fatty acid synthesis-related genes, was increased in the ethanol-fed mice and was decreased in the YCHT-administered mice. The expression of its downstream gene CYP7A1 had the same change tendency (Table 3). These data showed that YCHT could treat alcoholic fatty liver disease by reducing fatty acid and bile acid synthesis. At the same time, the gene expression of peroxisome proliferator-activated receptor *α* (PPAR*α*), a transcription factor of fatty acid *β*-oxidation related gene, and its downstream fatty acid oxidation related genes Ehhadh, Cyp4a10, Cyp4a14, Cyp4a31, and Cyp4a32 were all decreased in the ethanol-fed mice and were all increased in the YCHT-administered mice (Table 4, except PPAR*α* gene). Fold change (FC) of the PPAR*α* gene was 0.66 in the ethanol group compared with the control group, and it was FC = 1.58 in the YCHT group compared with the ethanol group (Table 6). Although the FC value of the PPAR*α* gene was out of the screening criteria (FC ≥ 2 or FC ≤ 0.5), its expression trend was consistent with its downstream genes, such as Ehhadh, Cyp4a10, Cyp4a14, Cyp4a31, and Cyp4a32. These data showed that YCHT could also treat alcoholic fatty liver disease by promoting fatty acid oxidation. In brief, the data suggested that the PPAR signaling pathway was the main potential pathway for YCHT to improve alcohol-induced hepatic steatosis in mice.

### 3.5. Verification of RNA-Seq Results by qRT-PCR

**Figure 5:**
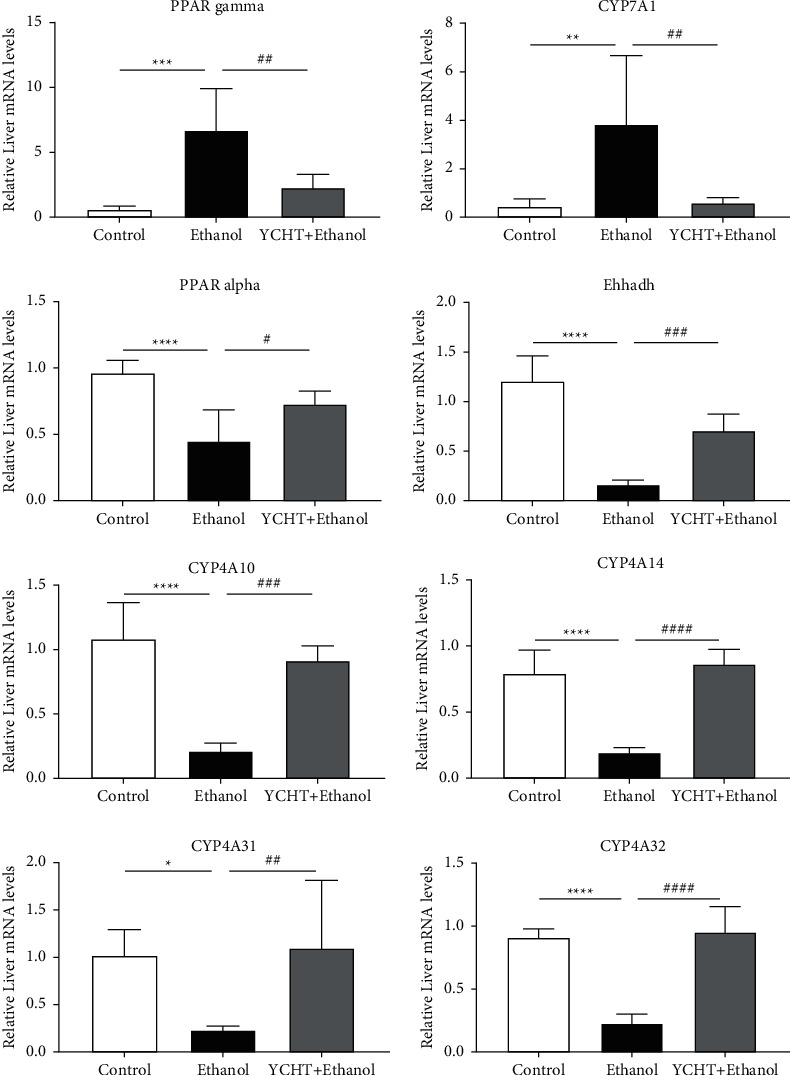
The relative mRNA expression levels of eight PPAR signaling pathway-related genes in mice liver. Results were presented as means ± SD (*n* = 3 mice/group).

To verify the accuracy of transcriptome analysis, eight genes on the PPAR signaling pathway were selected for quantitative reverse transcription-polymerase chain reaction (qRT-PCR) analysis. The results showed that the mRNA expression levels of PPAR signaling pathway-related genes verified by qRT-PCR were basically consistent with the RNA-seq samples, indicating that transcriptome analysis was credible (Figure 5 and Table 6).

Data in Figure 5 were all counted by one-way ANOVA followed by Bonferroni's multiple comparisons test to determine the statistical difference between two groups. Ethanol group versus control group, ^*∗*^*p* < 0.05,  ^*∗∗*^*p* < 0.01,  ^*∗∗∗*^*p* < 0.001,  ^*∗∗∗∗*^*p* < 0.0001; YCHT group versus ethanol group, ^#^*p* < 0.05,  ^##^*p* < 0.01,  ^###^*p* < 0.001,  ^####^*p* < 0.0001.

### 3.6. The Effects of YCHT on Protecting from Cell Injury Induced by Alcohol

**Figure 6:**
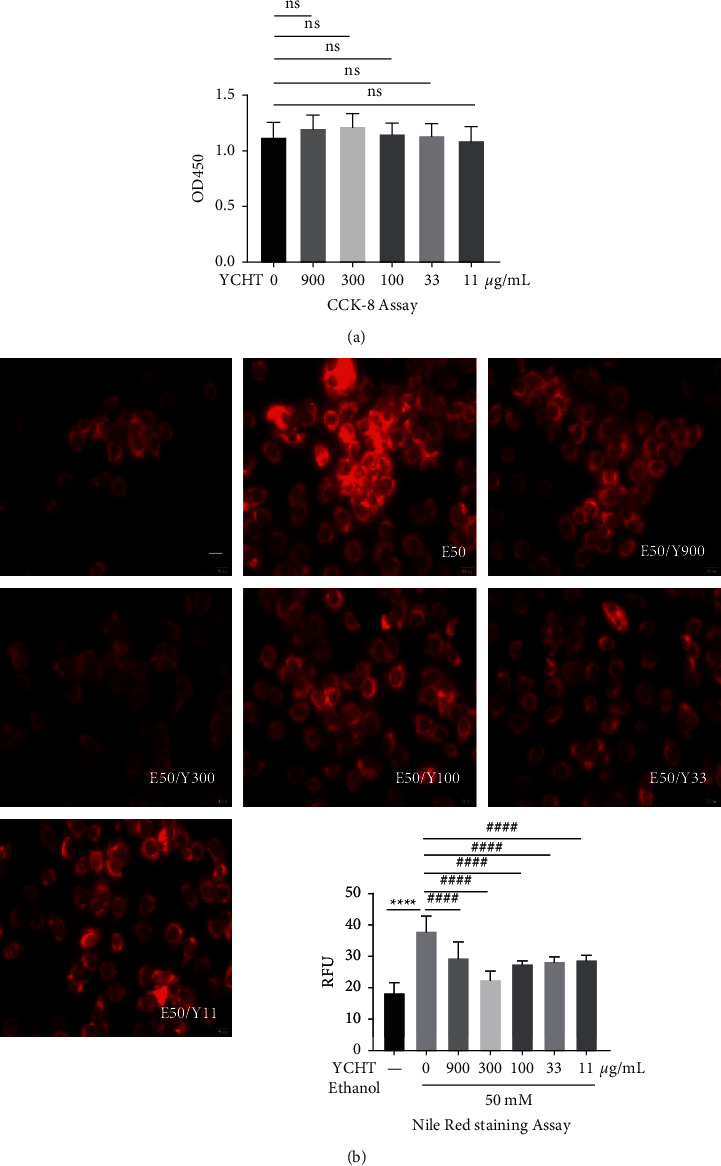
Cytotoxicity of YCHT on AML12 hepatocytes and the effect of YCHT on alcohol-induced AML12 hepatocytes steatosis. (a) The effect of different concentrations of YCHT on AML12 hepatocytes activity. (b) The effect of different concentrations of YCHT on the activity and fat production of AML12 hepatocytes stimulated by 50 mM alcohol. Results were presented as means ± SD (*n* = 3 replicates/group).

To investigate the effects of YCHT on cell injury induced by alcohol, the different concentrations of YCHT were used to treat AML12 cells with or without 50 mM alcohol for 12 h, and then cell activity was detected by CCK-8 and the lipid droplets in cells was observed by Nile Red fluorescent staining. It showed that YCHT at a concentration of 900 *μ*g/mL and below had no effect on cell activity and was not cytotoxic (Figure 6(a)). The results of Nile Red fluorescent staining suggested that 300 *μ*g/mL YCHT could obviously reduce the fat production of AML12 hepatocytes stimulated by 50 mM alcohol (Figure 6(b)). Therefore, in the subsequent pharmacological experiments, 300 *μ*g/mL was selected as the experimental concentration of YCHT.

Data in Figures 6(a) and 6(b) were all counted by one-way ANOVA followed by Bonferroni's multiple comparisons test to determine the statistical difference between two groups. Figure 6(a) shows different concentration YCHT groups versus control group, ns > 0.05. In Figure 6(b), ethanol group versus control group, ^*∗∗∗∗*^*p* < 0.0001; and different concentration YCHT groups versus ethanol group, ^####^*p* < 0.0001. Three separate experiments were performed with similar results. Pictures shown were from a single experiment. Data presented were the average of the three independent experiments.

### 3.7. YCHT Reduces Alcohol-Induced Steatosis of AML12 Hepatocytes by Inhibiting PPAR*γ* Expression and Promoting PPAR*α* Expression

**Figure 7:**
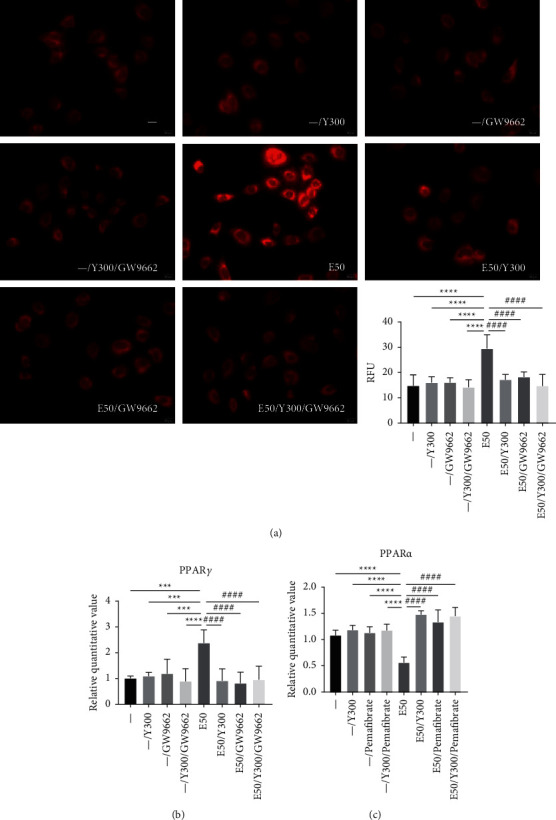
The effect of YCHT on alcohol-stimulated PPAR*γ* and PPAR*α* genes expression and steatosis in AML12 hepatocytes. (a) Nile Red staining. (b) The PPAR*γ* gene expression. (c) The PPAR*α* gene expression. Results were presented as means ± SD (*n* = 3 replicates/group).

Compared with the normal group, there was no significant difference in intracellular fat production in the YCHT, GW9662, and YCHT + GW9662 groups. The fat production in AML12 hepatocytes stimulated by alcohol was significantly increased. Compared with the alcohol group, the fat production in YCHT + alcohol, GW9662 + alcohol, YCHT + GW9662+alcohol group was significantly reduced, but there was no significant difference among YCHT + alcohol, GW9662+alcohol, and YCHT + GW9662 + alcohol group (Figure 7(a)).

Compared with the normal group, there was no significant difference in the expression of PPAR*γ* gene mRNA in the YCHT, GW9662, and YCHT + GW9662 group. The relative mRNA expression of the PPAR*γ* gene in the alcohol group was significantly increased and significantly reduced in the YCHT + alcohol group, but there was no significant difference between the YCHT + alcohol and GW9662 + alcohol group (Figure 7(b)). The above results indicated that YCHT could reduce the synthesis of fatty acids and bile acids by inhibiting the PPAR*γ* pathway.

Compared with the normal group, the relative mRNA expression of the PPAR*α* gene has no significant difference among the YCHT, pemafibrate, and YCHT + pemafibrate groups. After alcohol stimulating, the relative mRNA expression of the PPAR*α* gene was significantly reduced, while it increased after YCHT treatment. In addition, with the pemafibrate treatment, the relative mRNA expression of the PPAR*α* gene increased significantly, the same result as in the YCHT + pemafibrate group (Figure 7(c)). The above results indicated that YCHT could improve the oxidative decomposition of fatty acids by promoting the activation of the PPAR*α* pathway.

Data in Figures 7(a)–7(c) were all counted by one-way ANOVA followed by Bonferroni's multiple comparisons test to determine the statistical difference between two groups. Ethanol group versus control group, ^*∗∗∗∗*^*p* < 0.001 and ^*∗∗∗∗*^*p* < 0.0001; different concentration YCHT groups versus ethanol group, ^####^*p* < 0.0001. Three separate experiments were performed with similar results. Pictures shown were from a single experiment. Data presented were the average of the three independent experiments.

## 4. Discussion

Yin-Chen-Hao-Tang (YCHT) is one of the most famous hepatoprotective herbal formulas in China. YCHT and its modification have been widely used in the clinical treatment of jaundice and various types of liver diseases [10]. It is also used for alcoholic liver disease [4, 5]. YCHT had a good protective effect on serum enzymes and hepatocytes histological changes in acute [11] or chronic [7] rats liver injury induced by ethanol. It could reduce liver fat deposition and resist alcoholic hepatotoxicity in rats [7]. However, the molecular mechanism of YCHT against alcoholic fatty liver disease (AFLD) has not been clarified.

To explore the possible mechanism of YCHT in the prevention and treatment of AFLD, we first conducted a network pharmacological analysis of YCHT. Network pharmacology holds that the process of drugs in vivo is a complex action network, that is, the action process of “multicomponent, multitarget, and multipathway.” Our analysis results show that YCHT can exert the pharmacological effects of preventing and treating AFLD through multiple signal pathways such as inflammation-related pathways and lipid metabolism-related pathways. Inflammation-related pathways include the TNF signaling pathway, NF-kappa B signaling pathway, and Toll-like receptor signaling pathway, and lipid metabolism-related pathways include adipocytokine signaling pathway and PPAR signaling pathway. Because there are many signal pathways involved, we have carried out further research and exploration through experiments in vivo and in vitro, to obtain the most possible signal pathway and target of YCHT in the prevention and treatment of AFLD.

**Figure 8:**
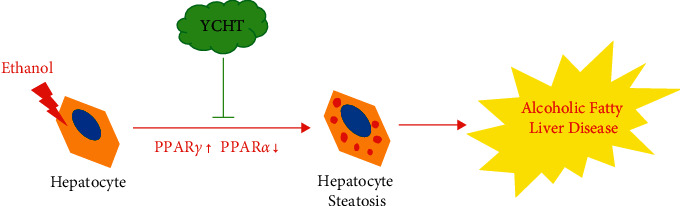
The prevention and treatment mechanism of YCHT on alcoholic fatty liver disease (AFLD). YCHT alleviated hepatocyte steatosis by reducing the expression of PPAR*γ,* which is related to the lipogenesis pathway, and increasing the expression of PPAR*α,* which is related to the lipolysis pathway, thereby improving AFLD.

In vivo experiments results showed that YCHT could significantly reduce mice liver injury and hepatic steatosis, and mice liver tissue RNA-seq transcriptome sequencing results showed that the mechanism of YCHT prevention and treatment of AFLD was most related to the PPAR-mediated fatty acid metabolism signaling pathway. Then, we confirmed that YCHT played a pharmacological role in inhibiting hepatocyte steatosis by downregulating PPAR*γ* and upregulating PPAR*α* gene expression through the hepatocyte AFLD model (Figure 8).

The main pathological feature of AFLD is excessive fat accumulation, namely, steatosis, in hepatocytes as a result of increased intracytoplasmic triglyceride formation [12]. PPAR signaling pathway plays an important role in the regulation of hepatic steatosis induced by alcohol [13]. Alcohol can activate the gene expression of PPAR*γ* [14] signaling pathway, which promotes the production of fat, thereby aggravating hepatic steatosis. On the contrary, alcohol can reduce the activity of the PPAR*α* signaling pathway, which decreases the fatty acids (FAs) *ß*-oxidation [15–17], thereby further promoting lipid accumulation in hepatocytes and ultimately leading to hepatic steatosis.

PPAR*γ*, a member of the nuclear hormone receptor superfamily which can regulate the expression of fatty acid synthesis-related genes, participates in lipid metabolism in metabolic tissues, such as liver and skeletal muscle [18]. The ethanol feeding induced liver fat accumulation and mRNA expression of hepatic PPAR*γ*2 in WT mice, which suggests that a high level of PPAR*γ*2 is a common driving force for fat accumulation induced by ethanol [19]. By specific knockout of the PPAR*γ* gene in mouse liver, it was found that alcohol-induced fat accumulation and liver injury in mouse liver were improved [14]. Cholesterol 7a-hydroxylase (CYP7A1) is the rate-limiting enzyme in the classic bile acid synthesis pathway, which plays a critical role in maintaining cholesterol and bile acid homeostasis [20]. PPAR*γ*/CYP7A1 pathway played an important role in the regulation of lipid accumulation and cholesterol metabolic transformation in L-02 human hepatic cells [21]. PPAR*γ* overexpression substantially increased the expression of the CYP7A1 gene [22]. Our RNA-seq data showed that alcohol significantly increased the gene expression of PPAR*γ* and CYP7A1, and YCHT markedly inhibited the expression of these two genes (Table 3). These results suggested that YCHT may reduce fatty acid and bile acid synthesis by reducing the gene expression of PPAR*γ* and CYP7A1 and finally improved AFLD. In cell experiments, we discovered that YCHT or GW9662 (PPAR*γ* inhibitor) could, respectively, inhibit PPAR*γ* gene expression and steatosis in ethanol-stimulated hepatocytes. The reduction of ethanol-stimulated hepatocyte steatosis by simultaneous administration of YCHT and GW9662 was almost the same as that of GW9662 alone; YCHT and GW9662 did not show synergistic inhibitory effect on ethanol-stimulated hepatocyte steatosis, indicating that they should play an antisteatosis role through the same signaling pathway. GW9662 alleviated ethanol-stimulated hepatocyte steatosis through inhibiting PPAR*γ* pathway, so YCHT also should exert its anti-ethanol-stimulated hepatocyte steatosis efficacy through PPAR*γ* pathway.

PPAR*α* is also a member of the nuclear hormone receptor superfamily. PPAR*α* can induce the expression of genes involved in the oxidation and transport of free fatty acids, including fatty acid *β*-oxidation pathways and membrane transporters, such as carnitine palmitoyltransferase 1 (CPT-1) and apolipoproteins [23]. Enzymes involved in fatty acid oxidation and regulated by PPAR*α* include acetyl-CoA carboxylase (ACC), fatty acid synthase (FAS), 3-hydroxy-3-methylglutaryl-CoA reductase (HMG-CoAR), cytochrome p4504 A (Cyp4a), and CPT-1 [24, 25]. Therefore, activation of these enzymes by PPAR*α* agonists can accelerate the degradation of various fatty acids, thus preventing and treating alcoholic liver disease [26]. PPAR*α* activity is positively regulated by NAD-dependent deacetylase sirtuin-1(SIRT1) in regulating lipid homeostasis. Ethanol-mediated SIRT1 inhibition and SIRT1 dysfunction may cause hyperacetylation of PPAR*α* in the liver. The disturbance of the PPAR*α* signaling pathway will reduce fatty acid oxidation and cause aggravated liver steatosis and inflammation [27]. Our RNA-seq data showed that alcohol could reduce the expression of the PPAR*α* gene and downstream fatty acid oxidation genes Ehhadh, Cyp4a10, Cyp4a14, Cyp4a31, and Cyp4a32. YCHT could increase the expression of these genes (Table 4). In cell experiments, we discovered that YCHT or pemafibrate (PPAR*α* agonist) could, respectively, promote PPAR*α* gene expression in ethanol-stimulated hepatocytes. The increase of PPAR*α* gene expression by simultaneous administration of YCHT and pemafibrate was almost the same as that of pemafibrate alone, and YCHT and pemafibrate did not show synergistic promotion effect on PPAR*α* gene expression, indicating that they might promote PPAR*α* gene expression through the same regulation mechanism. Taken together, these results suggested that YCHT promoted fatty acid oxidation by increasing the expression of PPAR*α* and finally improved AFLD.

## 5. Conclusions

YCHT reduces the expression of the PPAR*γ* gene, thereby inhibiting lipogenesis in the liver. Meanwhile, YCHT increases the expression of the PPAR*α* gene, thus promoting lipolysis in the liver and finally improving the alcohol-induced hepatic steatosis in mice. In brief, our results indicate that the PPAR signaling pathway is the most important potential targeted pathway for YCHT to improve alcoholic fatty liver disease (AFLD) (Figure 8).

## Figures and Tables

**Table 1:** Caloric profile of the diet.

	Control diet (TP4030C)	Ethanol diet (TP4030D)
Protein (kcal per liter)	180	180
Fat (kcal per liter)	350	350
Carbohydrate (kcal per liter)	470	190
Ethanol (kcal per liter)	0	280
Total (kcal per liter)	1,000	1,000

**Table 2:** Primer sequences used for PCR amplification.

Gene	Forward (5′ to 3′)	Reverse (5′ to 3′)
PPAR*γ*	TCGCTGATGCACTGCCTATG	GAGAGGTCCACAGAGCTGATT
CYP7A1	GGGATTGCTGTGGTAGTGAGG	GGTATGGAATCAACCCGTTGTC
PPAR*α*	AGAGCCCCATCTGTCCTCTC	ACTGGTAGTCTGCAAAACCAAA
Ehhadh	ACAGCGATACCAGAAGCCAG	TGGCAATCCGATAGTGACAGC
CYP4a10	TTCCCTGATGGACGCTCTTTA	GCAAACCTGGAAGGGTCAAAC
CYP4a14	GGAGCAATATACGAGTCCTGC	CAGAGTCCGCCATGATTTTGA
CYP4a31	CATCACCGCCCTTTCACTG	TCCCCCAGAACCATCGAGG
CYP4a32	ATGCCGACAATAACATGAAGGC	ACACGCTTACAATAGCCCAGG
GAPDH	TGACCTCAACTACATGGTCTACA	CTTCCCATTCTCGGCCTTG

**Table 3:** Fold change of differential expression genes upregulated by ethanol and downregulated by YCHT.

Gene name	FC (E/C)	FC (Y/E)	Gene name	FC (E/C)	FC (Y/E)
4933431E20Rik	2.82	0.41	Lmo2	2.23	0.30
Acot11	3.19	0.44	Lrat	2.22	0.43
Acot6	8.04	0.36	Lrtm1	5.87	0.45
Airn	6.64	0.28	Mid1ip1	2.31	0.23
Aldh3b3	2.35	0.39	Mmd2	6.52	0.31
Apol7a	2.09	0.49	Mtss1l	3.20	0.29
Arhgap24	2.34	0.39	Myh14	9.61	0.17
Arrdc4	2.90	0.39	Nes	2.31	0.33
Atp2b2	15.34	0.24	Npr3	3.61	0.40
Calcrl	2.02	0.39	Nptx1	13.54	0.12
Cbr3	2.82	0.18	Nr3c2	3.43	0.46
Cib2	2.15	0.45	Pakap	10.20	0.04
Col15a1	11.89	0.33	Phospho1	5.30	0.43
CYP7a1	6.19	0.19	Pparg	3.05	0.41
Dbp	2.43	0.34	Prss8	3.46	0.37
E030018B13Rik	2.37	0.32	Rasgrp3	2.08	0.39
Esrrg	2.46	0.40	Rgs12	3.33	0.41
Extl1	9.94	0.47	Rnf225	7.56	0.28
Fam198a	5.30	0.29	Robo1	5.95	0.42
Frmd8	3.61	0.39	RP23-392K24.6	57.54	0.13
Gatm	2.29	0.38	Samd4	7.56	0.19
Gbp6	2.29	0.35	Sirpa	4.87	0.34
Gdf10	3.65	0.48	Slc15a3	3.21	0.38
Gdf2	2.43	0.50	Slc1a4	4.14	0.42
Gm12265	6.27	0.29	Slc26a1	2.42	0.49
Gm20521	2.30	0.50	Slc26a6	2.42	0.46
Gm20708	12.58	0.07	Slc2a5	5.92	0.49
Gm35986	6.24	0.36	Slc34a2	42.56	0.13
Gm7694	4.15	0.39	Sorl1	3.26	0.39
Gnat1	13.64	0.37	Spata2l	3.04	0.42
Grin3b	11.34	0.30	Spsb2	2.05	0.46
Gstm4	8.47	0.49	Srxn1	9.75	0.41
Hgf	2.24	0.46	St6galnac4	5.63	0.34
Hmox1	3.01	0.32	Syt12	6.25	0.30
Il1b	3.83	0.14	Tgfbr2	5.78	0.45
Klf2	2.17	0.42	Tnfrsf11b	2.60	0.45
Klhdc7a	13.33	0.25	Tnfsf10	2.93	0.34
Lgr5	2.23	0.46	Tsc22d1	3.23	0.38
Lhfpl2	2.73	0.40	Ttc7	2.14	0.45
Lilr4b	3.39	0.43			

*Notes.* C: control group; E: ethanol group; Y: YCHT group; FC: fold change.

**Table 4:** Fold change of differential expression genes downregulated by ethanol and upregulated by YCHT.

Gene name	FC (E/C)	FC (Y/E)	Gene name	FC (E/C)	FC (Y/E)
8430408G22Rik	0.32	4.49	Gm43305	0.21	2.49
Acot1	0.16	2.74	Gm4952	0.46	2.09
Amotl2	0.33	2.22	Hes1	0.49	2.32
Apc	0.45	2.31	Il1r1	0.16	3.74
Arhgef37	0.38	2.74	Kpna1	0.24	4.19
Arntl	0.32	2.25	Map3k13	0.27	2.30
Cdkn1a	0.19	2.89	Nr0b2	0.22	6.26
Chrna2	0.32	3.02	Otud1	0.33	2.11
Clec2h	0.05	11.97	Pdk4	0.07	4.34
Clpx	0.36	2.33	Pfkfb1	0.41	2.14
CYP26a1	0.42	2.15	Ppm1k	0.46	2.26
CYP4a10	0.30	3.23	Ppp1r3c	0.41	2.18
CYP4a14	0.26	2.84	Rnf186	0.33	2.55
CYP4a31	0.28	2.37	Rnf24	0.37	2.26
CYP4a32	0.48	2.37	Scara5	0.12	8.89
D630039A03Rik	0.43	2.09	Sgk2	0.35	2.35
Ehhadh	0.18	2.80	Slc25a30	0.44	2.01
Elovl3	0.19	6.93	Smad9	0.14	2.91
Fam107a	0.05	4.84	Ttll8	0.20	3.09
Fam134b	0.34	2.62	Upp2	0.18	2.18
Fkbp5	0.42	2.08	Zbtb16	0.23	3.75

*Notes.* C: control group; E: ethanol group; Y: YCHT group; FC: fold change.

**Table 5:** KEGG signaling pathway enrichment.

Category	Term	Count	%	*p* value
KEGG PATHWAY	PPAR signaling pathway	7	6	0.000018
KEGG PATHWAY	Fatty acid degradation	5	4.3	0.00036
KEGG PATHWAY	Retinol metabolism	6	5.2	0.00038
KEGG PATHWAY	Inflammatory mediator regulation of TRP channels	6	5.2	0.0018
KEGG PATHWAY	Osteoclast differentiation	6	5.2	0.0018
KEGG PATHWAY	Arachidonic acid metabolism	5	4.3	0.0034
KEGG PATHWAY	Vascular smooth muscle contraction	5	4.3	0.012
KEGG PATHWAY	FoxO signaling pathway	5	4.3	0.014
KEGG PATHWAY	Transcriptional misregulation in cancer	5	4.3	0.028
KEGG PATHWAY	Metabolic pathways	15	12.9	0.051
KEGG PATHWAY	Pathways in cancer	7	6	0.057
KEGG PATHWAY	Metabolism of xenobiotics by cytochrome P450	3	2.6	0.074
KEGG PATHWAY	Cytokine-cytokine receptor interaction	5	4.3	0.089
KEGG PATHWAY	MAPK signaling pathway	5	4.3	0.099

**Table 6:** Fold change of gene expression obtained by RNA-seq and qRT-PCR.

Gene name	Method	FC (E/C)	FC (Y/E)
PPAR*γ*	RNA-seq	3.05	0.41
	qRT-PCR	11.67	0.34
CYP7a1	RNA-seq	6.19	0.19
	qRT-PCR	8.88	0.16
PPAR*α*	RNA-seq	0.66	1.58
	qRT-PCR	0.47	1.6
Ehhadh	RNA-seq	0.18	2.80
	qRT-PCR	0.14	4.28
CYP4a10	RNA-seq	0.30	3.23
	qRT-PCR	0.20	5.94
CYP4a14	RNA-seq	0.26	2.84
	qRT-PCR	0.24	4.47
CYP4a31	RNA-seq	0.28	2.37
	qRT-PCR	0.23	4.77
CYP4a32	RNA-seq	0.48	2.37
	qRT-PCR	0.25	4.17

*Notes.* C: control group; E: ethanol group; Y: YCHT group; FC: fold change.

## Data Availability

The data used to support the findings of this study are available from the corresponding author upon request.

## Abbreviations

AFLD: Alcoholic fatty liver disease
ALD: Alcoholic liver disease
ALT: Alanine aminotransferase
Cyp4a: Cytochrome p4504A
CYP7A1: Cholesterol 7a-hydroxylase
FC: Fold change
FDR: False discovery rate
FPKM: Fragments per kilobase of exon model per million mapped reads
KEGG: Kyoto encyclopedia of genes and genomes
PPAR*α*: Peroxisome proliferator-activated receptor *aα*
PPAR*γ*: Peroxisome proliferator-activated receptor *γ*
PPI: Protein-protein interaction
qPCR: Quantitative PCR
RNA-Seq: RNA sequencing
TG: triglyceride
TMM: Trimmed mean of *M* values
YCHT: Yin-Chen-Hao-Tang.

## Supplementary Materials

Supplementary Materials Supplementary Material 1-1: Herb Target-Artemisiae Scopariae Herba. Supplementary Material 1-2: Herb Target-Gardeniae Fructus. Supplementary Material 1-3: Herb Target-Radix Rhei Et Rhizome. Supplementary Material 2: AFLD-GeneCards-SearchResults. Supplementary Material 3: string_interactions.tsv default node.
