# Supplementary material for: The Study of Yin-Chen-Hao-Tang Preventing and Treating Alcoholic Fatty Liver Disease through PPAR Signaling Pathway Based on Network Pharmacology and RNA-Seq Transcriptomics
Source: Evid Based Complement Alternat Med. 2021 Dec 31;2021:8917993. doi: 10.1155/2021/8917993 (PMC8741355; doi:10.1155/2021/8917993)
Supplement: Supplementary Materials — Supplementary Material 1-1: Herb Target-Artemisiae Scopariae Herba. Supplementary Material 1-2: Herb Target-Gardeniae Fructus. Supplementary Material 1-3: Herb Target-Radix Rhei Et Rhizome. Supplementary Material 2: AFLD-GeneCards-SearchResults. Supplementary Material 3: string_interactions.tsv default node. [file 8917993.f1.zip › 8917993.f1/Supplementary material 1-1 Herb Target-Artemisiae Scopariae Herba.pdf]

| Mol ID | Molecule Name | Target Name | drugbank | Status |
|---|---|---|---|---|
| MOL000118 | Terpineol | M3 | 51 | |
| MOL000118 | Terpineol | M1 | 103 | |
| MOL000118 | (L)-alpha-Terpineol | Gamma-aminobutyric-acid receptor subunit alpha-2 | 423 | |
| MOL000118 | (L)-alpha-Terpineol | Gamma-aminobutyric-acid receptor subunit alpha-5 | 523 | |
| MOL000118 | (L)-alpha-Terpineol | Sodium-dependent noradrenaline transporter | 540 | |
| MOL000118 | (L)-alpha-Terpineol | Gamma-aminobutyric-acid receptor subunit alpha-3 | 580 | |
| MOL000118 | Terpineol | M2 | 617 | |
| MOL000118 | Terpineol | Alpha-1B adrenergic receptor | 632 | |
| MOL000118 | Terpineol | transporter | 713 | |
| MOL000118 | (L)-alpha-Terpineol | Gamma-aminobutyric-acid receptor subunit alpha-1 | 872 | |
| MOL000118 | (L)-alpha-Terpineol | Gamma-aminobutyric-acid receptor subunit alpha-6 | 841 | |
| MOL000118 | Terpineol | Prostaglandin G/H synthase 1 | 20 | |
| MOL000118 | (L)-alpha-Terpineol | Sodium channel protein type 5 subunit alpha | 220 | |
| MOL000118 | Terpineol | Coagulation factor X | 239 | |
| MOL000118 | Terpineol | Prostaglandin G/H synthase 2 | 290 | |
| MOL000118 | Terpineol | DNA topoisomerase 2-alpha | 817 | |
| MOL000118 | Terpineol | Heat shock protein HSP 90-alpha | 1939 | |
| MOL000118 | Terpineol | Nuclear receptor coactivator 2 | 6241 | |
| MOL000118 | (L)-alpha-Terpineol | Calcium-activated potassium channel subunit alpha 1 | 610 | |
| MOL000118 | Terpineol | Cytochrome P450-cam | 2298 | |
| MOL000118 | Terpineol | Alpha-1A adrenergic receptor | 556 | |
| MOL000118 | Terpineol | Ig gamma-1 chain C region | 4785 | |
| MOL000126 | (-)-nopinene | Gamma-aminobutyric-acid receptor subunit alpha-2 | 423 | |
| MOL000126 | (-)-nopinene | Gamma-aminobutyric-acid receptor subunit alpha-1 | 872 | |
| MOL000126 | (-)-nopinene | Cytochrome P450-cam | 2298 | |
| MOL000126 | (-)-nopinene | Prostaglandin G/H synthase 1 | 20 | |
| MOL000126 | (-)-nopinene | M3 | 51 | |
| MOL000126 | (-)-nopinene | M1 | 103 | |
| MOL000126 | (-)-nopinene | Prostaglandin G/H synthase 2 | 290 | |
| MOL000126 | (-)-nopinene | Retinoic acid receptor RXR-alpha | 459 | |
| MOL000126 | (-)-nopinene | Sodium-dependent noradrenaline transporter | 540 | |
| MOL000126 | (-)-nopinene | Gamma-aminobutyric-acid receptor subunit alpha-3 | 580 | |
| MOL000126 | (-)-nopinene | M2 | 617 | |
| MOL000126 | (-)-nopinene | Alpha-1B adrenergic receptor | 632 | |
| MOL000126 | (-)-nopinene | Nuclear receptor coactivator 2 | 6241 | |
| MOL000126 | (-)-nopinene | Gamma-aminobutyric-acid receptor subunit alpha-6 | 841 | |
| MOL000126 | (-)-nopinene | Alpha-2C adrenergic receptor | 378 | |
| MOL000126 | (-)-nopinene | Alpha-1A adrenergic receptor | 556 | |
| MOL000126 | (-)-nopinene | transporter | 713 | |

| | | | | |
|---|---|---|---|---|
| MOL000126 | (-)-nopinene | Amine oxidase [flavin-containing] A | 3941 | |
| MOL000126 | (-)-nopinene | Lysozyme | 2300 | |
| MOL000126 | (-)-nopinene | Gamma-aminobutyric-acid receptor subunit alpha-5 | 523 | |
| MOL000126 | (-)-nopinene | Neuronal acetylcholine receptor subunit alpha-2 | 813 | |
| MOL000172 | Furol | | | |
| MOL000172 | Furol | | | |
| MOL000172 | Furol | | | |
| MOL000172 | Furol | Lysozyme | 2300 | |
| MOL000172 | Furol | Nicotinate-nucleotide--dimethylbenzimidazole phosphoribosyltransferase | 2264 | |
| MOL001801 | salicylic acid | Prostaglandin G/H synthase 1 | 20 | |
| MOL001801 | salicylic acid | Prostaglandin G/H synthase 2 | 290 | |
| MOL001801 | salicylic acid | Amine oxidase [flavin-containing] B | 3939 | |
| MOL001801 | salicylic acid | | | |
| MOL001801 | salicylic acid | | | |
| MOL001801 | salicylic acid | Lysozyme | 2300 | |
| MOL001801 | salicylic acid | Nicotinate-nucleotide--dimethylbenzimidazole phosphoribosyltransferase | 2264 | |
| MOL001801 | salicylic acid | Trypsin-3 | 2886 | |
| MOL001801 | salicylic acid | Arachidonate 5-lipoxygenase | 275 | validated |
| MOL001801 | salicylic acid | | | |
| MOL001801 | salicylic acid | Chymotrypsinogen B | 6011 | |
| MOL001801 | salicylic acid | Transcription factor p65 | h001 | validated |
| MOL001801 | salicylic acid | Fatty acid synthase | 1295 | validated |
| MOL001801 | salicylic acid | Superoxide dismutase [Cu-Zn] | 4152 | validated |
| MOL001801 | salicylic acid | | | validated |
| MOL001801 | salicylic acid | Endothelin-1 | h001 | validated |
| MOL001801 | salicylic acid | Interleukin-4 | h001 | validated |
| MOL001801 | salicylic acid | Neutrophil cytosol factor 1 | h001 | validated |
| MOL001801 | salicylic acid | Serum paraoxonase/arylesterase 1 | 1198 | validated |
| MOL001801 | salicylic acid | Apolipoprotein A-I | h001 | validated |
| MOL001801 | salicylic acid | Plasminogen | 234 | validated |
| MOL001801 | salicylic acid | subunit | 1787 | validated |
| MOL001801 | salicylic acid | Ferritin, mitochondrial | h001 | validated |
| MOL001801 | salicylic acid | 6-phosphofructokinase, muscle type | h001 | validated |
| MOL001801 | salicylic acid | C1 | 139 | validated |
| MOL001801 | salicylic acid | Thyroid peroxidase | 563 | validated |
| MOL001801 | salicylic acid | Beta-glucuronidase | 1354 | validated |
| MOL001801 | salicylic acid | Hairy/enhancer-of-split related with YRPW motif protein 1 | h001 | validated |
| MOL001801 | salicylic acid | Prolow-density lipoprotein receptor-related protein 1 | h001 | validated |
| MOL001801 | salicylic acid | Tyrosine aminotransferase | 5493 | validated |
| MOL001801 | salicylic acid | Golgi-associated plant pathogenesis-related protein 1 | 5369 | validated |
| MOL001801 | salicylic acid | Membrane primary amine oxidase | h001 | validated |
| MOL001801 | salicylic acid | Telomeric repeat-binding factor 1 | h001 | validated |
| MOL001880 | OXL | Prostaglandin G/H synthase 1 | 20 | |

| | | | |
|---|---|---|---|
| MOL001880 | OXL | Nitric-oxide synthase, brain | 76 |
| MOL001880 | OXL | Procollagen-lysine,2-oxoglutarate 5-dioxygenase 1 | 97 |
| MOL001880 | OXL | mitochondrial | 147 |
| MOL001880 | OXL | Succinate semialdehyde dehydrogenase, mitochondrial | 170 |
| MOL001880 | OXL | 4-aminobutyrate aminotransferase, mitochondrial | 280 |
| MOL001880 | OXL | Prostaglandin G/H synthase 2 | 290 |
| MOL001880 | OXL | Serine hydroxymethyltransferase, mitochondrial | 321 |
| MOL001880 | OXL | DNA polymerase | 338 |
| MOL001880 | OXL | Glutamate [NMDA] receptor subunit zeta-1 | 401 |
| MOL001880 | OXL | Kynureninase | 435 |
| MOL001880 | OXL | Ornithine aminotransferase, mitochondrial | 472 |
| MOL001880 | OXL | Glycine receptor subunit alpha-1 | 482 |
| MOL001880 | OXL | Aspartate aminotransferase, cytoplasmic | 586 |
| MOL001880 | OXL | Glutamate [NMDA] receptor subunit epsilon-1 | 837 |
| MOL001880 | OXL | Gamma-aminobutyric-acid receptor subunit alpha-1 | 872 |
| MOL001880 | OXL | Proto-oncogene tyrosine-protein kinase Src | 933 |
| MOL001880 | OXL | Cathepsin D | 1243 |
| MOL001880 | OXL | Lactotransferrin | 1439 |
| MOL001880 | OXL | Macrophage metalloelastase | 2203 |
| MOL001880 | OXL | Leukotriene A-4 hydrolase | 3060 |
| MOL001880 | OXL | M-phase inducer phosphatase 2 | 3105 |
| MOL001880 | OXL | cytoplasmic | 3610 |
| MOL001880 | OXL | Cholinesterase | 3923 |
| MOL001880 | OXL | Xanthine dehydrogenase/oxidase | 3947 |
| MOL001880 | OXL | | |
| MOL001880 | OXL | | |
| MOL001880 | OXL | | |
| MOL001880 | OXL | | |
| MOL001880 | OXL | | |
| MOL001880 | OXL | | |
| MOL001880 | OXL | | |
| MOL001880 | OXL | | |
| MOL001880 | OXL | Monocarboxylate transporter 2 | 489 |
| MOL001880 | OXL | Cytochrome P450-cam | 2298 |
| MOL001880 | OXL | Aspartate aminotransferase | 2249 |
| MOL001880 | OXL | Beta-galactosidase | 2592 |
| MOL001880 | OXL | Ferrichrome-iron receptor | 2427 |
| MOL001880 | OXL | Histidinol dehydrogenase | 3191 |
| MOL001880 | OXL | Pyruvate dehydrogenase [cytochrome] | 845 |
| MOL001880 | OXL | Acetyl-CoA acetyltransferase | 2596 |
| MOL001880 | OXL | Formate dehydrogenase H | 2638 |
| MOL001880 | OXL | Formate acetyltransferase 1 | 3178 |

| | | | |
|---|---|---|---|
| MOL001880 | OXL | Adenylosuccinate synthetase | 2632 |
| MOL001880 | OXL | Succinate dehydrogenase flavoprotein subunit | 5725 |
| MOL001880 | OXL | Alanine racemase | 2453 |
| MOL001880 | OXL | Gag-Pol polyprotein | 2237 |
| MOL001880 | OXL | Methionine synthase | 2935 |
| MOL001880 | OXL | C4-dicarboxylate transport transcriptional regulatory protein dctD | 3336 |
| MOL001880 | OXL | D-alanyl-D-alanine carboxypeptidase | 2461 |
| MOL001880 | OXL | Isocitrate dehydrogenase [NADP] | 2286 |
| MOL001880 | OXL | Phosphoenolpyruvate carboxykinase [ATP] | 4794 |
| MOL001880 | OXL | Siroheme synthase | 4318 |
| MOL001880 | OXL | Aconitate hydratase 2 | 3553 |
| MOL001880 | OXL | Monomeric sarcosine oxidase | 2380 |
| MOL001880 | OXL | Bacillolysin | 2457 |
| MOL001880 | OXL | Dihydroxyacetone kinase | 2397 |
| MOL001880 | OXL | Haloalkane dehalogenase | 2281 |
| MOL001880 | OXL | dehydrogenase | 2881 |
| MOL001880 | OXL | Fumarate reductase flavoprotein subunit | 2709 |
| MOL001880 | OXL | 2-isopropylmalate synthase | 3179 |
| MOL001880 | OXL | 1-aminocyclopropane-1-carboxylate deaminase | 2518 |
| MOL001880 | OXL | Hydroxylamine reductase | 3274 |
| MOL001880 | OXL | Nicotinate-nucleotide--dimethylbenzimidazole phosphoribosyltransferase | 2264 |
| MOL001880 | OXL | Glucose--fructose oxidoreductase | 2826 |
| MOL001880 | OXL | Growth-inhibiting protein 18 | 3877 |
| MOL001880 | OXL | | |
| MOL001880 | OXL | | |
| MOL001880 | OXL | | |
| MOL001880 | OXL | Methylmalonyl-CoA carboxyltransferase 5S subunit | 2983 |
| MOL001880 | OXL | L-cysteine/cystine lyase C-DES | 4802 |
| MOL001880 | OXL | Malonamidase E2 | 4600 |
| MOL001880 | OXL | Serotransferrin | 566 |
| MOL001880 | OXL | Glutamate dehydrogenase 1, mitochondrial | 201 |
| MOL001880 | OXL | Aspartate aminotransferase, mitochondrial | 426 |
| MOL001880 | OXL | Pyruvate kinase isozymes M1/M2 | 98 |
| MOL001880 | OXL | Serine--pyruvate aminotransferase | 349 |
| MOL001880 | OXL | Monocarboxylate transporter 7 | 195 |
| MOL001880 | OXL | Alanine aminotransferase 1 | 735 |
| MOL001880 | OXL | Aldehyde dehydrogenase X, mitochondrial | 531 |
| MOL001880 | OXL | Succinate dehydrogenase [ubiquinone] flavoprotein subunit, | 197 |
| MOL001880 | OXL | Cystathionine gamma-lyase | 868 |

| | | | |
|---|---|---|---|
| MOL001880 | OXL | Serine hydroxymethyltransferase, cytosolic | 367 |
| MOL001880 | OXL | Betaine--homocysteine S-methyltransferase 1 | 941 |
| MOL001880 | OXL | Succinyl-CoA:3-ketoacid-coenzyme A transferase 2, mitochondrial | 4008 |
| MOL001880 | OXL | Alanine--glyoxylate aminotransferase 2, mitochondrial | 114 |
| MOL001880 | OXL | NADP-dependent malic enzyme | 666 |
| MOL001880 | OXL | Glutamate dehydrogenase 2, mitochondrial | 830 |
| MOL001880 | OXL | Alanyl-tRNA synthetase, cytoplasmic | 323 |
| MOL001880 | OXL | Glycine amidinotransferase, mitochondrial | 383 |
| MOL001880 | OXL | Calcium-transporting ATPase type 2C member 1 | 241 |
| MOL001880 | OXL | S-adenosylmethionine synthetase isoform type-1 | 453 |
| MOL001880 | OXL | Cysteine desulfurase, mitochondrial | 169 |
| MOL001880 | OXL | Alanine--glyoxylate aminotransferase 2-like 2 | 3899 |
| MOL001880 | OXL | Proton-coupled amino acid transporter 1 | 593 |
| MOL001880 | OXL | 1 | 3978 |
| MOL001880 | OXL | L-lactate dehydrogenase A chain | 473 |
| MOL001880 | OXL | Glycine receptor subunit alpha-2 | 820 |
| MOL001880 | OXL | 2-amino-3-ketobutyrate coenzyme A ligase, mitochondrial | 575 |
| MOL001880 | OXL | Pyruvate dehydrogenase E1 component subunit beta, mitochondrial | 110 |
| MOL001880 | OXL | Triosephosphate isomerase | 6346 |
| MOL001880 | OXL | Delta-1-pyrroline-5-carboxylate dehydrogenase, mitochondrial | 271 |
| MOL001880 | OXL | NAD-dependent malic enzyme, mitochondrial | 166 |
| MOL001880 | OXL | Glycine receptor subunit alpha-3 | 461 |
| MOL001880 | OXL | SHMT2 protein | 3884 |
| MOL001880 | OXL | NADP-dependent malic enzyme, mitochondrial | 363 |
| MOL001880 | OXL | L-lactate dehydrogenase B chain | 77 |
| MOL001880 | OXL | Trypsin-3 | 2886 |
| MOL001880 | OXL | Sigma factor sigB regulation protein rsbQ | 3490 |
| MOL001880 | OXL | aminotransferase | 2537 |
| MOL001880 | OXL | Aldose reductase | 822 |
| MOL001880 | OXL | Glycogen phosphorylase, muscle form | 1152 |
| MOL001880 | OXL | Fumarate hydratase class II | 3486 |
| MOL001880 | OXL | N-acetylneuraminate lyase | 3000 |
| MOL001880 | OXL | Phosphate-binding protein pstS precursor | 3694 |
| MOL001880 | OXL | Beta-amylase | 3365 |
| MOL001880 | OXL | Tyrosine-protein kinase transforming protein Src | 2556 |

| | | | | |
|---|---|---|---|---|
| MOL001880 | OXL | 2-hydroxy-6-oxo-7-methylocta-2,4-dienoate hydrolase | 4674 | |
| MOL001880 | OXL | Phosphotriesterase | 5742 | |
| MOL001880 | OXL | NADH-ubiquinone oxidoreductase 75 kDa subunit, mitochondrial | 548 | |
| MOL001880 | OXL | Gephyrin | 4532 | |
| MOL001880 | OXL | Succinyl-CoA:3-ketoacid-coenzyme A transferase 1, mitochondrial | 4003 | |
| MOL001880 | OXL | 2 | 4000 | |
| MOL001880 | OXL | Calcium-binding mitochondrial carrier protein Aralar2 | 513 | |
| MOL001880 | OXL | Succinyl-CoA ligase [ADP-forming] beta-chain, mitochondrial | 4009 | |
| MOL001999 | scoparone | Prostaglandin G/H synthase 1 | 20 | |
| MOL001999 | scoparone | Prothrombin | 54 | |
| MOL001999 | scoparone | M1 | 103 | |
| MOL001999 | scoparone | Prostaglandin G/H synthase 2 | 290 | |
| MOL001999 | scoparone | Beta-2 adrenergic receptor | 766 | |
| MOL001999 | scoparone | transporter | 824 | |
| MOL001999 | scoparone | Gamma-aminobutyric-acid receptor subunit alpha-1 | 872 | |
| MOL001999 | scoparone | Leukotriene A-4 hydrolase | 3060 | |
| MOL001999 | scoparone | Amine oxidase [flavin-containing] B | 3939 | |
| MOL001999 | scoparone | cAMP-dependent protein kinase catalytic subunit alpha | 6263 | |
| MOL001999 | scoparone | Glutamate receptor 2 | 921 | |
| MOL001999 | scoparone | cAMP-dependent protein kinase inhibitor alpha | 6264 | |
| MOL001999 | scoparone | Transcription factor p65 | h001 | validated |
| MOL001999 | scoparone | NF-kappa-B inhibitor alpha | h001 | validated |
| MOL001999 | scoparone | Small inducible cytokine A2 | 1649 | validated |
| MOL001999 | scoparone | Interleukin-8 | h001 | validated |
| MOL001999 | scoparone | Tyrosinase | h001 | validated |
| MOL001999 | scoparone | 5,6-dihydroxyindole-2-carboxylic acid oxidase | h001 | validated |
| MOL001999 | scoparone | L-dopachrome tautomerase | h001 | validated |
| MOL000207 | Methyleugenol | Prostaglandin G/H synthase 1 | 20 | |
| MOL000207 | Methyleugenol | D(1A) dopamine receptor | 23 | |
| MOL000207 | Methyleugenol | M3 | 51 | |
| MOL000207 | Methyleugenol | M1 | 103 | |
| MOL000207 | Methyleugenol | Beta-1 adrenergic receptor | 193 | |
| MOL000207 | Methyleugenol | Sodium channel protein type 5 subunit alpha | 220 | |
| MOL000207 | Methyleugenol | Prostaglandin G/H synthase 2 | 290 | |
| MOL000207 | Methyleugenol | Nitric-oxide synthase, endothelial | 291 | |
| MOL000207 | Methyleugenol | Alpha-2A adrenergic receptor | 318 | |
| MOL000207 | Methyleugenol | Alpha-2C adrenergic receptor | 378 | |
| MOL000207 | Methyleugenol | 5-hydroxytryptamine 2A receptor | 502 | |
| MOL000207 | Methyleugenol | Sodium-dependent noradrenaline transporter | 540 | |
| MOL000207 | Methyleugenol | Alpha-1A adrenergic receptor | 556 | |
| MOL000207 | Methyleugenol | M2 | 617 | |

| | | | | |
|---|---|---|---|---|
| MOL000207 | Methyleugenol | Alpha-2B adrenergic receptor | 629 | |
| MOL000207 | Methyleugenol | Alpha-1B adrenergic receptor | 632 | |
| MOL000207 | Methyleugenol | transporter | 713 | |
| MOL000207 | Methyleugenol | Beta-2 adrenergic receptor | 766 | |
| MOL000207 | Methyleugenol | Alpha-1D adrenergic receptor | 789 | |
| MOL000207 | Methyleugenol | Beta-lactamase | 2478 | |
| MOL000207 | Methyleugenol | Amine oxidase [flavin-containing] B | 3939 | |
| MOL000207 | Methyleugenol | Leukotriene A-4 hydrolase | 3060 | |
| MOL000207 | Methyleugenol | transporter | 824 | |
| MOL000207 | Methyleugenol | activator | 1074 | |
| MOL000207 | Methyleugenol | cAMP-dependent protein kinase inhibitor alpha | 6264 | |
| MOL000251 | Rhamnocitrin | Nitric oxide synthase, inducible | 7 | |
| MOL000251 | Rhamnocitrin | Prostaglandin G/H synthase 1 | 20 | |
| MOL000251 | Rhamnocitrin | Estrogen receptor | 136 | |
| MOL000251 | Rhamnocitrin | Androgen receptor | 146 | |
| MOL000251 | Rhamnocitrin | Peroxisome proliferator-activated receptor gamma | 238 | |
| MOL000251 | Rhamnocitrin | Prostaglandin G/H synthase 2 | 290 | |
| MOL000251 | Rhamnocitrin | Estrogen receptor beta | 869 | |
| MOL000251 | Rhamnocitrin | Dipeptidyl peptidase 4 | 952 | |
| MOL000251 | Rhamnocitrin | Mitogen-activated protein kinase 14 | 1540 | |
| MOL000251 | Rhamnocitrin | Glycogen synthase kinase-3 beta | 1721 | |
| MOL000251 | Rhamnocitrin | Heat shock protein HSP 90-alpha | 1939 | |
| MOL000251 | Rhamnocitrin | Cell division protein kinase 2 | 2240 | |
| MOL000251 | Rhamnocitrin | Phosphatidylinositol-4,5-bisphosphate 3-kinase catalytic subunit gamma isoform | 2404 | |
| MOL000251 | Rhamnocitrin | Chk1 | 5790 | |
| MOL000251 | Rhamnocitrin | cAMP-dependent protein kinase catalytic subunit alpha | 6263 | |
| MOL000251 | Rhamnocitrin | Trypsin-1 | 3176 | |
| MOL000251 | Rhamnocitrin | Calmodulin | 465 | |
| MOL000251 | Rhamnocitrin | Nuclear receptor coactivator 2 | 6241 | |
| MOL000251 | Rhamnocitrin | Aldose reductase | 822 | |
| MOL000254 | eugenol | Prostaglandin G/H synthase 1 | 20 | |
| MOL000254 | eugenol | D(1A) dopamine receptor | 23 | |
| MOL000254 | eugenol | M3 | 51 | |
| MOL000254 | eugenol | M1 | 103 | |
| MOL000254 | eugenol | Beta-1 adrenergic receptor | 193 | |
| MOL000254 | eugenol | Prostaglandin G/H synthase 2 | 290 | validated |
| MOL000254 | eugenol | Nitric-oxide synthase, endothelial | 291 | |
| MOL000254 | eugenol | Alpha-2A adrenergic receptor | 318 | |
| MOL000254 | eugenol | Alpha-2C adrenergic receptor | 378 | |
| MOL000254 | eugenol | Sodium-dependent noradrenaline transporter | 540 | |
| MOL000254 | eugenol | Alpha-1A adrenergic receptor | 556 | |
| MOL000254 | eugenol | M2 | 617 | |
| MOL000254 | eugenol | Alpha-1B adrenergic receptor | 632 | |
| MOL000254 | eugenol | transporter | 713 | |
| MOL000254 | eugenol | Beta-2 adrenergic receptor | 766 | |
| MOL000254 | eugenol | Beta-lactamase | 2478 | |

| | | | | |
|---|---|---|---|---|
| MOL000254 | eugenol | Amine oxidase [flavin-containing] B | 3939 | validated |
| MOL000254 | eugenol | Amine oxidase [flavin-containing] A | 3941 | validated |
| MOL000254 | eugenol | Lysozyme | 2300 | |
| MOL000254 | eugenol | Chymotrypsinogen B | 6011 | |
| MOL000254 | eugenol | Alpha-1D adrenergic receptor | 789 | |
| MOL000254 | eugenol | Leukotriene A-4 hydrolase | 3060 | |
| MOL000254 | eugenol | Alpha-2B adrenergic receptor | 629 | |
| MOL000254 | eugenol | activator | 1074 | |
| MOL000254 | eugenol | Thermolysin | 6285 | |
| MOL000254 | eugenol | Sodium channel protein type 5 subunit alpha | 220 | |
| MOL000254 | eugenol | Transcription factor p65 | h001 | validated |
| MOL000254 | eugenol | Cytochrome P450 1A1 | h001 | validated |
| MOL000254 | eugenol | Cytochrome P450 1B1 | h001 | validated |
| MOL000254 | eugenol | Arachidonate 5-lipoxygenase | 275 | validated |
| MOL000254 | eugenol | Aryl hydrocarbon receptor | 6220 | validated |
| MOL000254 | eugenol | Canalicular multispecific organic anion transporter 1 | h001 | validated |
| MOL000254 | eugenol | CD86 | 942 | validated |
| MOL000254 | eugenol | Serine/threonine-protein phosphatase 2B catalytic subunit | 6746 | validated |
| MOL000254 | eugenol | Pepsin A | 6231 | validated |
| MOL000254 | eugenol | Mucin-1 | h001 | validated |
| MOL000254 | eugenol | Quinone oxidoreductase | 2159 | validated |
| MOL000254 | eugenol | Calcium-transporting ATPase type 2C member 1 | 241 | validated |
| MOL000254 | eugenol | Short transient receptor potential channel 3 | h001 | validated |
| MOL000254 | eugenol | Transient receptor potential cation channel subfamily V member 3 | 5838 | validated |
| MOL000254 | eugenol | 2 | h001 | validated |
| MOL002818 | Piceol | | | |
| MOL002818 | Piceol | Lysozyme | 2300 | |
| MOL002818 | Piceol | Nicotinate-nucleotide--dimethylbenzimidazole phosphoribosyltransferase | 2264 | |
| MOL002818 | Piceol | | | |
| MOL000339 | Isoscopoletin | Prostaglandin G/H synthase 1 | 20 | |
| MOL000339 | Isoscopoletin | D(1A) dopamine receptor | 23 | |
| MOL000339 | Isoscopoletin | Prothrombin | 54 | |
| MOL000339 | Isoscopoletin | M1 | 103 | |
| MOL000339 | Isoscopoletin | Prostaglandin G/H synthase 2 | 290 | |
| MOL000339 | Isoscopoletin | Beta-2 adrenergic receptor | 766 | |
| MOL000339 | Isoscopoletin | Leukotriene A-4 hydrolase | 3060 | |
| MOL000339 | Isoscopoletin | Amine oxidase [flavin-containing] B | 3939 | |
| MOL000339 | Isoscopoletin | cAMP-dependent protein kinase catalytic subunit alpha | 6263 | |
| MOL000339 | Isoscopoletin | Glutamate receptor 2 | 921 | |
| MOL000339 | Isoscopoletin | cAMP-dependent protein kinase inhibitor alpha | 6264 | |
| MOL000339 | Isoscopoletin | Ribosyldihydronicotinamide dehydrogenase [quinone] | 243 | |

| | | | | |
|---|---|---|---|---|
| MOL000339 | Isoscopoletin | Queuine tRNA-ribosyltransferase | 2561 | |
| MOL000354 | isorhamnetin | Nitric oxide synthase, inducible | 7 | validated |
| MOL000354 | isorhamnetin | Prostaglandin G/H synthase 1 | 20 | |
| MOL000354 | isorhamnetin | Estrogen receptor | 136 | |
| MOL000354 | isorhamnetin | Androgen receptor | 146 | |
| MOL000354 | isorhamnetin | Peroxisome proliferator-activated receptor gamma | 238 | |
| MOL000354 | isorhamnetin | Prostaglandin G/H synthase 2 | 290 | |
| MOL000354 | isorhamnetin | Tyrosine-protein phosphatase non-receptor type 1 | 687 | |
| MOL000354 | isorhamnetin | Estrogen receptor beta | 869 | |
| MOL000354 | isorhamnetin | Dipeptidyl peptidase 4 | 952 | |
| MOL000354 | isorhamnetin | Mitogen-activated protein kinase 14 | 1540 | |
| MOL000354 | isorhamnetin | Glycogen synthase kinase-3 beta | 1721 | |
| MOL000354 | isorhamnetin | Heat shock protein HSP 90-alpha | 1939 | |
| MOL000354 | isorhamnetin | Cell division protein kinase 2 | 2240 | |
| MOL000354 | isorhamnetin | Phosphatidylinositol-4,5-bisphosphate 3-kinase catalytic subunit gamma isoform | 2404 | |
| MOL000354 | isorhamnetin | cAMP-dependent protein kinase catalytic subunit alpha | 6263 | |
| MOL000354 | isorhamnetin | Trypsin-1 | 3176 | |
| MOL000354 | isorhamnetin | Proto-oncogene serine/threonine-protein kinase Pim-1 | 2347 | |
| MOL000354 | isorhamnetin | Cyclin-A2 | 6235 | |
| MOL000354 | isorhamnetin | Nuclear receptor coactivator 2 | 6241 | |
| MOL000354 | isorhamnetin | Calmodulin | 465 | |
| MOL000354 | isorhamnetin | Glycogen phosphorylase, muscle form | 1152 | |
| MOL000354 | isorhamnetin | Peroxisome proliferator-activated receptor delta | 1502 | |
| MOL000354 | isorhamnetin | Chk1 | 5790 | |
| MOL000354 | isorhamnetin | Aldose reductase | 822 | |
| MOL000354 | isorhamnetin | Nuclear receptor coactivator 1 | 6228 | |
| MOL000354 | isorhamnetin | Coagulation factor VII | 369 | |
| MOL000354 | isorhamnetin | Prothrombin | 54 | |
| MOL000354 | isorhamnetin | Nitric-oxide synthase, endothelial | 291 | |
| MOL000354 | isorhamnetin | Acetylcholinesterase | 474 | |
| MOL000354 | isorhamnetin | Gamma-aminobutyric-acid receptor subunit alpha-1 | 872 | |
| MOL000354 | isorhamnetin | Amine oxidase [flavin-containing] B | 3939 | |
| MOL000354 | isorhamnetin | Glutamate receptor 2 | 921 | |
| MOL000354 | isorhamnetin | Cytochrome P450-cam | 2298 | |
| MOL000354 | isorhamnetin | Transcription factor p65 | h001 | validated |
| MOL000354 | isorhamnetin | Xanthine dehydrogenase/oxidase | 3947 | validated |
| MOL000354 | isorhamnetin | Neutrophil cytosol factor 1 | h001 | validated |
| MOL000354 | isorhamnetin | Oxidized low-density lipoprotein receptor 1 | h001 | validated |
| MOL000358 | beta-sitosterol | Progesterone receptor | 614 | |
| MOL000358 | beta-sitosterol | Nuclear receptor coactivator 2 | 6241 | |
| MOL000358 | beta-sitosterol | Prostaglandin G/H synthase 1 | 20 | |
| MOL000358 | beta-sitosterol | Prostaglandin G/H synthase 2 | 290 | |
| MOL000358 | beta-sitosterol | Heat shock protein HSP 90-alpha | 1939 | |

| | | | | |
|---|---|---|---|---|
| MOL000358 | beta-sitosterol | Phosphatidylinositol-4,5-bisphosphate 3-kinase catalytic subunit gamma isoform | 2404 | |
| MOL000358 | beta-sitosterol | Potassium voltage-gated channel subfamily H member 2 | 101 | |
| MOL000358 | beta-sitosterol | cAMP-dependent protein kinase catalytic subunit alpha | 6263 | |
| MOL000358 | beta-sitosterol | D(1A) dopamine receptor | 23 | |
| MOL000358 | beta-sitosterol | M3 | 51 | |
| MOL000358 | beta-sitosterol | M1 | 103 | |
| MOL000358 | beta-sitosterol | Sodium channel protein type 5 subunit alpha | 220 | |
| MOL000358 | beta-sitosterol | Gamma-aminobutyric-acid receptor subunit alpha-2 | 423 | |
| MOL000358 | beta-sitosterol | M4 | 450 | |
| MOL000358 | beta-sitosterol | cGMP-inhibited 3',5'-cyclic phosphodiesterase A | 485 | |
| MOL000358 | beta-sitosterol | 5-hydroxytryptamine 2A receptor | 502 | |
| MOL000358 | beta-sitosterol | Gamma-aminobutyric-acid receptor subunit alpha-5 | 523 | |
| MOL000358 | beta-sitosterol | Alpha-1A adrenergic receptor | 556 | |
| MOL000358 | beta-sitosterol | Gamma-aminobutyric-acid receptor subunit alpha-3 | 580 | |
| MOL000358 | beta-sitosterol | M2 | 617 | |
| MOL000358 | beta-sitosterol | Alpha-1B adrenergic receptor | 632 | |
| MOL000358 | beta-sitosterol | Beta-2 adrenergic receptor | 766 | |
| MOL000358 | beta-sitosterol | Neuronal acetylcholine receptor subunit alpha-2 | 813 | |
| MOL000358 | beta-sitosterol | transporter | 824 | |
| MOL000358 | beta-sitosterol | Mu-type opioid receptor | 847 | |
| MOL000358 | beta-sitosterol | Gamma-aminobutyric-acid receptor subunit alpha-1 | 872 | |
| MOL000358 | beta-sitosterol | Neuronal acetylcholine receptor subunit alpha-7 | 4095 | |
| MOL000358 | beta-sitosterol | Cytochrome P450-cam | 2298 | |
| MOL000358 | beta-sitosterol | Apoptosis regulator Bcl-2 | 273 | validated |
| MOL000358 | beta-sitosterol | Apoptosis regulator BAX | h001 | validated |
| MOL000358 | beta-sitosterol | Caspase-9 | h001 | validated |
| MOL000358 | beta-sitosterol | Transcription factor AP-1 | 1629 | validated |
| MOL000358 | beta-sitosterol | Caspase-3 | h001 | validated |
| MOL000358 | beta-sitosterol | Caspase-8 | h001 | validated |
| MOL000358 | beta-sitosterol | Protein kinase C alpha type | h001 | validated |
| MOL000358 | beta-sitosterol | Transforming growth factor beta-1 | h001 | validated |
| MOL000358 | beta-sitosterol | Serum paraoxonase/arylesterase 1 | 1198 | validated |
| MOL000358 | beta-sitosterol | Microtubule-associated protein 2 | 1852 | validated |
| MOL000036 | beta-caryophyllene | Prostaglandin G/H synthase 1 | 20 | |
| MOL000036 | beta-caryophyllene | M3 | 51 | |
| MOL000036 | beta-caryophyllene | M1 | 103 | |
| MOL000036 | beta-caryophyllene | Prostaglandin G/H synthase 2 | 290 | |
| MOL000036 | beta-caryophyllene | Gamma-aminobutyric-acid receptor subunit alpha-2 | 423 | |
| MOL000036 | beta-caryophyllene | Retinoic acid receptor RXR-alpha | 459 | |
| MOL000036 | beta-caryophyllene | M2 | 617 | |

| | | | | |
|---|---|---|---|---|
| MOL000036 | beta-caryophyllene | Alpha-1B adrenergic receptor | 632 | |
| MOL000036 | beta-caryophyllene | Neuronal acetylcholine receptor subunit alpha-2 | 813 | |
| MOL000036 | beta-caryophyllene | Gamma-aminobutyric-acid receptor subunit alpha-1 | 872 | |
| MOL000036 | beta-caryophyllene | Nuclear receptor coactivator 2 | 6241 | |
| MOL000036 | beta-caryophyllene | Gamma-aminobutyric-acid receptor subunit alpha-6 | 841 | |
| MOL000036 | beta-caryophyllene | Sodium-dependent noradrenaline transporter | 540 | |
| MOL000036 | beta-caryophyllene | Alpha-1A adrenergic receptor | 556 | |
| MOL000036 | beta-caryophyllene | transporter | 713 | |
| MOL000036 | beta-caryophyllene | Interleukin-6 | 1159 | validated |
| MOL000040 | Scopoletol | Prostaglandin G/H synthase 1 | 20 | |
| MOL000040 | Scopoletol | Prothrombin | 54 | |
| MOL000040 | Scopoletol | Ribosyldihydronicotinamide dehydrogenase [quinone] | 243 | |
| MOL000040 | Scopoletol | Prostaglandin G/H synthase 2 | 290 | |
| MOL000040 | Scopoletol | Carbonic anhydrase 1 | 295 | |
| MOL000040 | Scopoletol | Beta-2 adrenergic receptor | 766 | |
| MOL000040 | Scopoletol | Leukotriene A-4 hydrolase | 3060 | |
| MOL000040 | Scopoletol | Amine oxidase [flavin-containing] B | 3939 | |
| MOL000040 | Scopoletol | Glutamate receptor 2 | 921 | |
| MOL000040 | Scopoletol | cAMP-dependent protein kinase catalytic subunit alpha | 6263 | |
| MOL000040 | Scopoletol | Sodium-dependent noradrenaline transporter | 540 | |
| MOL000040 | Scopoletol | Queuine tRNA-ribosyltransferase | 2561 | |
| MOL000415 | rutin | DNA topoisomerase 2-alpha | 817 | |
| MOL000415 | rutin | Transcription factor p65 | h001 | validated |
| MOL000415 | rutin | Tumor necrosis factor | 777 | validated |
| MOL000415 | rutin | Interleukin-6 | 1159 | validated |
| MOL000415 | rutin | Caspase-3 | h001 | validated |
| MOL000415 | rutin | | | validated |
| MOL000415 | rutin | Superoxide dismutase [Cu-Zn] | 4152 | validated |
| MOL000415 | rutin | Interleukin-1 beta | 1654 | validated |
| MOL000415 | rutin | Interleukin-8 | h001 | validated |
| MOL000415 | rutin | Protein kinase C beta type | h001 | validated |
| MOL000415 | rutin | Arachidonate 5-lipoxygenase | 275 | validated |
| MOL000415 | rutin | 3-hydroxy-3-methylglutaryl-coenzyme A reductase | 3387 | validated |
| MOL000415 | rutin | Hyaluronan synthase 2 | h001 | validated |
| MOL000415 | rutin | Type I iodothyronine deiodinase | h001 | validated |
| MOL000415 | rutin | receptor | h001 | validated |
| MOL000415 | rutin | Insulin | 5961 | validated |
| MOL000415 | rutin | Low affinity immunoglobulin epsilon Fc receptor | h001 | validated |
| MOL000415 | rutin | Integrin beta-2 | 1630 | validated |
| MOL000415 | rutin | Thromboxane A2 receptor | 835 | validated |
| MOL000437 | Hirsutrin | DNA topoisomerase 2-alpha | 817 | |
| MOL000437 | Hirsutrin | Tyrosine-protein phosphatase non-receptor type 1 | 687 | |

| | | | |
|---|---|---|---|
| MOL000437 | Hirsutrin | Coagulation factor X | 239 |
| MOL004609 | Areapillin | Nitric oxide synthase, inducible | 7 |
| MOL004609 | Areapillin | Prothrombin | 54 |
| MOL004609 | Areapillin | Androgen receptor | 146 |
| MOL004609 | Areapillin | Sodium channel protein type 5 subunit alpha | 220 |
| MOL004609 | Areapillin | Coagulation factor X | 239 |
| MOL004609 | Areapillin | Prostaglandin G/H synthase 2 | 290 |
| MOL004609 | Areapillin | Coagulation factor VII | 369 |
| MOL004609 | Areapillin | Tyrosine-protein phosphatase non-receptor type 1 | 687 |
| MOL004609 | Areapillin | DNA topoisomerase 2-alpha | 817 |
| MOL004609 | Areapillin | Estrogen receptor beta | 869 |
| MOL004609 | Areapillin | Dipeptidyl peptidase 4 | 952 |
| MOL004609 | Areapillin | Heat shock protein HSP 90-alpha | 1939 |
| MOL004609 | Areapillin | Ig gamma-1 chain C region | 4785 |
| MOL004609 | Areapillin | Trypsin-1 | 3176 |
| MOL004609 | Areapillin | Nuclear receptor coactivator 2 | 6241 |
| MOL004609 | Areapillin | Nuclear receptor coactivator 1 | 6228 |
| MOL004609 | Areapillin | Calmodulin | 465 |
| MOL004617 | Ayapanin | Prostaglandin G/H synthase 1 | 20 |
| MOL004617 | Ayapanin | Prothrombin | 54 |
| MOL004617 | Ayapanin | Prostaglandin G/H synthase 2 | 290 |
| MOL004617 | Ayapanin | Alpha-2A adrenergic receptor | 318 |
| MOL004617 | Ayapanin | transporter | 713 |
| MOL004617 | Ayapanin | Beta-2 adrenergic receptor | 766 |
| MOL004617 | Ayapanin | transporter | 824 |
| MOL004617 | Ayapanin | Leukotriene A-4 hydrolase | 3060 |
| MOL004617 | Ayapanin | Amine oxidase [flavin-containing] B | 3939 |
| MOL004617 | Ayapanin | Amine oxidase [flavin-containing] A | 3941 |
| MOL004617 | Ayapanin | cAMP-dependent protein kinase catalytic subunit alpha | 6263 |
| MOL004617 | Ayapanin | M1 | 103 |
| MOL004617 | Ayapanin | Alpha-1A adrenergic receptor | 556 |
| MOL004617 | Ayapanin | Dipeptidyl peptidase 4 | 952 |
| MOL004617 | Ayapanin | cAMP-dependent protein kinase inhibitor alpha | 6264 |
| MOL004734 | Butal | Haloalkane dehalogenase | 2281 |
| MOL004734 | Butal | A2 | 6270 |
| MOL004734 | Butal | Trypsin-3 | 2886 |
| MOL005109 | Capillarin | Prostaglandin G/H synthase 1 | 20 |
| MOL005109 | Capillarin | Prostaglandin G/H synthase 2 | 290 |
| MOL005109 | Capillarin | Amine oxidase [flavin-containing] B | 3939 |
| MOL005109 | Capillarin | cAMP-dependent protein kinase catalytic subunit alpha | 6263 |
| MOL005573 | Genkwanin | Nitric oxide synthase, inducible | 7 |
| MOL005573 | Genkwanin | Prostaglandin G/H synthase 1 | 20 |
| MOL005573 | Genkwanin | Androgen receptor | 146 |
| MOL005573 | Genkwanin | Prostaglandin G/H synthase 2 | 290 |
| MOL005573 | Genkwanin | Retinoic acid receptor RXR-alpha | 459 |
| MOL005573 | Genkwanin | Estrogen receptor beta | 869 |
| MOL005573 | Genkwanin | Dipeptidyl peptidase 4 | 952 |

| | | | | |
|---|---|---|---|---|
| MOL005573 | Genkwanin | Heat shock protein HSP 90-alpha | 1939 | |
| MOL005573 | Genkwanin | cAMP-dependent protein kinase catalytic subunit alpha | 6263 | |
| MOL005573 | Genkwanin | Trypsin-1 | 3176 | |
| MOL005573 | Genkwanin | Nuclear receptor coactivator 2 | 6241 | |
| MOL005573 | Genkwanin | Nuclear receptor coactivator 1 | 6228 | |
| MOL005573 | Genkwanin | Calmodulin | 465 | |
| MOL005573 | Genkwanin | Chk1 | 5790 | |
| MOL000635 | vanillin | Amine oxidase [flavin-containing] B | 3939 | |
| MOL000635 | vanillin | Amine oxidase [flavin-containing] A | 3941 | |
| MOL000635 | vanillin | Lysozyme | 2300 | |
| MOL000635 | vanillin | Nicotinate-nucleotide--dimethylbenzimidazole phosphoribosyltransferase | 2264 | |
| MOL000635 | vanillin | Prostaglandin G/H synthase 2 | 290 | |
| MOL000635 | vanillin | Gamma-aminobutyric-acid receptor subunit alpha-1 | 872 | |
| MOL000635 | vanillin | | | |
| MOL000635 | vanillin | Cytochrome P450-cam | 2298 | |
| MOL000635 | vanillin | Matrix metalloproteinase-9 | h001 | validated |
| MOL000635 | vanillin | Mitogen-activated protein kinase 1 | 1176 | validated |
| MOL000635 | vanillin | Transcription factor AP-1 | 1629 | validated |
| MOL006589 | Artepillin C | Prostaglandin G/H synthase 1 | 20 | |
| MOL006589 | Artepillin C | M1 | 103 | |
| MOL006589 | Artepillin C | Estrogen receptor | 136 | |
| MOL006589 | Artepillin C | Peroxisome proliferator-activated receptor gamma | 238 | |
| MOL006589 | Artepillin C | Prostaglandin G/H synthase 2 | 290 | |
| MOL006589 | Artepillin C | cGMP-inhibited 3',5'-cyclic phosphodiesterase A | 485 | |
| MOL006589 | Artepillin C | Sodium-dependent noradrenaline transporter | 540 | |
| MOL006589 | Artepillin C | transporter | 713 | |
| MOL006589 | Artepillin C | Beta-2 adrenergic receptor | 766 | |
| MOL006589 | Artepillin C | Leukotriene A-4 hydrolase | 3060 | |
| MOL007260 | Isorhamnetin-3-mono-beta-D- | Coagulation factor X | 239 | |
| MOL007260 | Isorhamnetin-3-mono-beta-D- | DNA topoisomerase 2-alpha | 817 | |
| MOL007260 | Isorhamnetin-3-mono-beta-D- | Tyrosine-protein phosphatase non-receptor type 1 | 687 | |
| MOL007274 | Skrofulein | Nitric oxide synthase, inducible | 7 | |
| MOL007274 | Skrofulein | Prostaglandin G/H synthase 1 | 20 | |
| MOL007274 | Skrofulein | Sodium channel protein type 5 subunit alpha | 220 | |
| MOL007274 | Skrofulein | Prostaglandin G/H synthase 2 | 290 | |
| MOL007274 | Skrofulein | Dipeptidyl peptidase 4 | 952 | |
| MOL007274 | Skrofulein | Heat shock protein HSP 90-alpha | 1939 | |
| MOL007274 | Skrofulein | cAMP-dependent protein kinase catalytic subunit alpha | 6263 | |
| MOL007274 | Skrofulein | Trypsin-1 | 3176 | |
| MOL007274 | Skrofulein | Nuclear receptor coactivator 2 | 6241 | |

| | | | |
|---|---|---|---|
| MOL007274 | Skrofulein | Nuclear receptor coactivator 1 | 6228 |
| MOL007274 | Skrofulein | Calmodulin | 465 |
| MOL007405 | Cirsilineol | Nitric oxide synthase, inducible | 7 |
| MOL007405 | Cirsilineol | Potassium voltage-gated channel subfamily H member 2 | 101 |
| MOL007405 | Cirsilineol | Androgen receptor | 146 |
| MOL007405 | Cirsilineol | Sodium channel protein type 5 subunit alpha | 220 |
| MOL007405 | Cirsilineol | Coagulation factor X | 239 |
| MOL007405 | Cirsilineol | Prostaglandin G/H synthase 2 | 290 |
| MOL007405 | Cirsilineol | Nitric-oxide synthase, endothelial | 291 |
| MOL007405 | Cirsilineol | Coagulation factor VII | 369 |
| MOL007405 | Cirsilineol | DNA topoisomerase 2-alpha | 817 |
| MOL007405 | Cirsilineol | Estrogen receptor beta | 869 |
| MOL007405 | Cirsilineol | Dipeptidyl peptidase 4 | 952 |
| MOL007405 | Cirsilineol | Peroxisome proliferator-activated receptor delta | 1502 |
| MOL007405 | Cirsilineol | Mitogen-activated protein kinase 14 | 1540 |
| MOL007405 | Cirsilineol | Glycogen synthase kinase-3 beta | 1721 |
| MOL007405 | Cirsilineol | Heat shock protein HSP 90-alpha | 1939 |
| MOL007405 | Cirsilineol | Cell division protein kinase 2 | 2240 |
| MOL007405 | Cirsilineol | Chk1 | 5790 |
| MOL007405 | Cirsilineol | Trypsin-1 | 3176 |
| MOL007405 | Cirsilineol | Nuclear receptor coactivator 2 | 6241 |
| MOL007405 | Cirsilineol | Nuclear receptor coactivator 1 | 6228 |
| MOL007405 | Cirsilineol | Calcium-activated potassium channel subunit alpha 1 | 610 |
| MOL007405 | Cirsilineol | Calmodulin | 465 |
| MOL007405 | Cirsilineol | Prothrombin | 54 |
| MOL007405 | Cirsilineol | cAMP-dependent protein kinase catalytic subunit alpha | 6263 |
| MOL008039 | Isoarcapillin | Nitric oxide synthase, inducible | 7 |
| MOL008039 | Isoarcapillin | Prothrombin | 54 |
| MOL008039 | Isoarcapillin | Androgen receptor | 146 |
| MOL008039 | Isoarcapillin | Sodium channel protein type 5 subunit alpha | 220 |
| MOL008039 | Isoarcapillin | Coagulation factor X | 239 |
| MOL008039 | Isoarcapillin | Prostaglandin G/H synthase 2 | 290 |
| MOL008039 | Isoarcapillin | Coagulation factor VII | 369 |
| MOL008039 | Isoarcapillin | Tyrosine-protein phosphatase non-receptor type 1 | 687 |
| MOL008039 | Isoarcapillin | DNA topoisomerase 2-alpha | 817 |
| MOL008039 | Isoarcapillin | Estrogen receptor beta | 869 |
| MOL008039 | Isoarcapillin | Dipeptidyl peptidase 4 | 952 |
| MOL008039 | Isoarcapillin | Heat shock protein HSP 90-alpha | 1939 |
| MOL008039 | Isoarcapillin | Trypsin-1 | 3176 |
| MOL008039 | Isoarcapillin | Nuclear receptor coactivator 2 | 6241 |
| MOL008039 | Isoarcapillin | Calmodulin | 465 |
| MOL008040 | Eupalitin | Nitric oxide synthase, inducible | 7 |
| MOL008040 | Eupalitin | Prostaglandin G/H synthase 1 | 20 |
| MOL008040 | Eupalitin | Androgen receptor | 146 |

| | | | | |
|---|---|---|---|---|
| MOL008040 | Eupalitin | Sodium channel protein type 5 subunit alpha | 220 | |
| MOL008040 | Eupalitin | Prostaglandin G/H synthase 2 | 290 | |
| MOL008040 | Eupalitin | Estrogen receptor beta | 869 | |
| MOL008040 | Eupalitin | Dipeptidyl peptidase 4 | 952 | |
| MOL008040 | Eupalitin | Mitogen-activated protein kinase 14 | 1540 | |
| MOL008040 | Eupalitin | Glycogen synthase kinase-3 beta | 1721 | |
| MOL008040 | Eupalitin | Heat shock protein HSP 90-alpha | 1939 | |
| MOL008040 | Eupalitin | Cell division protein kinase 2 | 2240 | |
| MOL008040 | Eupalitin | Chk1 | 5790 | |
| MOL008040 | Eupalitin | Trypsin-1 | 3176 | |
| MOL008040 | Eupalitin | Calmodulin | 465 | |
| MOL008040 | Eupalitin | Prothrombin | 54 | |
| MOL008040 | Eupalitin | cAMP-dependent protein kinase catalytic subunit alpha | 6263 | |
| MOL008041 | Eupatolitin | Nitric oxide synthase, inducible | 7 | |
| MOL008041 | Eupatolitin | Androgen receptor | 146 | |
| MOL008041 | Eupatolitin | Sodium channel protein type 5 subunit alpha | 220 | |
| MOL008041 | Eupatolitin | Prostaglandin G/H synthase 2 | 290 | |
| MOL008041 | Eupatolitin | DNA topoisomerase 2-alpha | 817 | |
| MOL008041 | Eupatolitin | Estrogen receptor beta | 869 | |
| MOL008041 | Eupatolitin | Dipeptidyl peptidase 4 | 952 | |
| MOL008041 | Eupatolitin | Heat shock protein HSP 90-alpha | 1939 | |
| MOL008041 | Eupatolitin | Cell division protein kinase 2 | 2240 | |
| MOL008041 | Eupatolitin | Trypsin-1 | 3176 | |
| MOL008043 | capillarisin | Prostaglandin G/H synthase 1 | 20 | |
| MOL008043 | capillarisin | Prostaglandin G/H synthase 2 | 290 | |
| MOL008043 | capillarisin | Tyrosine-protein phosphatase non-receptor type 1 | 687 | |
| MOL008043 | capillarisin | Aldose reductase | 822 | |
| MOL008043 | capillarisin | Dipeptidyl peptidase 4 | 952 | |
| MOL008043 | capillarisin | Glycogen phosphorylase, muscle form | 1152 | |
| MOL008043 | capillarisin | Mitogen-activated protein kinase 14 | 1540 | |
| MOL008043 | capillarisin | Glycogen synthase kinase-3 beta | 1721 | |
| MOL008043 | capillarisin | Heat shock protein HSP 90-alpha | 1939 | |
| MOL008043 | capillarisin | Cell division protein kinase 2 | 2240 | |
| MOL008043 | capillarisin | Phosphatidylinositol-4,5-bisphosphate 3-kinase catalytic subunit gamma isoform | 2404 | |
| MOL008043 | capillarisin | Chk1 | 5790 | |
| MOL008043 | capillarisin | cAMP-dependent protein kinase catalytic subunit alpha | 6263 | |
| MOL008043 | capillarisin | Calmodulin | 465 | |
| MOL008043 | capillarisin | Matrix metalloproteinase-9 | h001 | validated |
| MOL008044 | Methylcapillarisin | Nitric oxide synthase, inducible | 7 | |
| MOL008044 | Methylcapillarisin | Prostaglandin G/H synthase 1 | 20 | |
| MOL008044 | Methylcapillarisin | Prothrombin | 54 | |
| MOL008044 | 7-Methylcapillarisin | Sodium channel protein type 5 subunit alpha | 220 | |
| MOL008044 | Methylcapillarisin | Prostaglandin G/H synthase 2 | 290 | |

| | | | |
|---|---|---|---|
| MOL008044 | 7-Methylcapillarisin | Tyrosine-protein phosphatase non-receptor type 1 | 687 |
| MOL008044 | Methylcapillarisin | Estrogen receptor beta | 869 |
| MOL008044 | Methylcapillarisin | Dipeptidyl peptidase 4 | 952 |
| MOL008044 | Methylcapillarisin | Heat shock protein HSP 90-alpha | 1939 |
| MOL008044 | Methylcapillarisin | Cell division protein kinase 2 | 2240 |
| MOL008044 | Methylcapillarisin | Chk1 | 5790 |
| MOL008044 | 7-Methylcapillarisin | cAMP-dependent protein kinase catalytic subunit alpha | 6263 |
| MOL008044 | Methylcapillarisin | Calmodulin | 465 |
| MOL008045 | 4'-Methylcapillarisin | Prothrombin | 54 |
| MOL008045 | 4'-Methylcapillarisin | Sodium channel protein type 5 subunit alpha | 220 |
| MOL008045 | 4'-Methylcapillarisin | Prostaglandin G/H synthase 2 | 290 |
| MOL008045 | 4'-Methylcapillarisin | Tyrosine-protein phosphatase non-receptor type 1 | 687 |
| MOL008045 | 4'-Methylcapillarisin | Dipeptidyl peptidase 4 | 952 |
| MOL008045 | 4'-Methylcapillarisin | Mitogen-activated protein kinase 14 | 1540 |
| MOL008045 | 4'-Methylcapillarisin | Glycogen synthase kinase-3 beta | 1721 |
| MOL008045 | 4'-Methylcapillarisin | Heat shock protein HSP 90-alpha | 1939 |
| MOL008045 | 4'-Methylcapillarisin | Cell division protein kinase 2 | 2240 |
| MOL008045 | 4'-Methylcapillarisin | cAMP-dependent protein kinase catalytic subunit alpha | 6263 |
| MOL008045 | 4'-Methylcapillarisin | Calmodulin | 465 |
| MOL008046 | Demethoxycapillarisin | Prostaglandin G/H synthase 1 | 20 |
| MOL008046 | Demethoxycapillarisin | Estrogen receptor | 136 |
| MOL008046 | Demethoxycapillarisin | Peroxisome proliferator-activated receptor gamma | 238 |
| MOL008046 | Demethoxycapillarisin | Prostaglandin G/H synthase 2 | 290 |
| MOL008046 | Demethoxycapillarisin | Tyrosine-protein phosphatase non-receptor type 1 | 687 |
| MOL008046 | Demethoxycapillarisin | Dipeptidyl peptidase 4 | 952 |
| MOL008046 | Demethoxycapillarisin | Mitogen-activated protein kinase 14 | 1540 |
| MOL008046 | Demethoxycapillarisin | Glycogen synthase kinase-3 beta | 1721 |
| MOL008046 | Demethoxycapillarisin | Heat shock protein HSP 90-alpha | 1939 |
| MOL008046 | Demethoxycapillarisin | Cell division protein kinase 2 | 2240 |

| | | | |
|---|---|---|---|
| MOL008046 | Demethoxycapillarisin | cAMP-dependent protein kinase catalytic subunit alpha | 6263 |
| MOL008046 | Demethoxycapillarisin | Aldose reductase | 822 |
| MOL008046 | Demethoxycapillarisin | Phosphatidylinositol-4,5-bisphosphate 3-kinase catalytic subunit gamma isoform | 2404 |
| MOL008047 | Artepillin A | Nitric oxide synthase, inducible | 7 |
| MOL008047 | Artepillin A | Prostaglandin G/H synthase 1 | 20 |
| MOL008047 | Artepillin A | Prothrombin | 54 |
| MOL008047 | Artepillin A | M1 | 103 |
| MOL008047 | Artepillin A | Estrogen receptor | 136 |
| MOL008047 | Artepillin A | Androgen receptor | 146 |
| MOL008047 | Artepillin A | Prostaglandin G/H synthase 2 | 290 |
| MOL008047 | Artepillin A | Nitric-oxide synthase, endothelial | 291 |
| MOL008047 | Artepillin A | cGMP-inhibited 3',5'-cyclic phosphodiesterase A | 485 |
| MOL008047 | Artepillin A | Alpha-1A adrenergic receptor | 556 |
| MOL008047 | Artepillin A | Beta-2 adrenergic receptor | 766 |
| MOL008047 | Artepillin A | Estrogen receptor beta | 869 |
| MOL008047 | Artepillin A | Dipeptidyl peptidase 4 | 952 |
| MOL008047 | Artepillin A | Glycogen synthase kinase-3 beta | 1721 |
| MOL008047 | Artepillin A | Cell division protein kinase 2 | 2240 |
| MOL008047 | Artepillin A | Beta-lactamase | 2478 |
| MOL008047 | Artepillin A | Trypsin-1 | 3176 |
| MOL008052 | Capillene | Amine oxidase [flavin-containing] B | 3939 |
| MOL008054 | Capillanol | Amine oxidase [flavin-containing] B | 3939 |
| MOL008054 | Capillanol | Amine oxidase [flavin-containing] A | 3941 |
| MOL008054 | Capillanol | cAMP-dependent protein kinase inhibitor alpha | 6264 |
| MOL008057 | Capillin | Prostaglandin G/H synthase 1 | 20 |
| MOL008057 | Capillin | Prostaglandin G/H synthase 2 | 290 |
| MOL008057 | Capillin | Amine oxidase [flavin-containing] B | 3939 |
| MOL008057 | Capillin | Amine oxidase [flavin-containing] A | 3941 |
| MOL000908 | beta-elemene | Prostaglandin G/H synthase 2 | 290 |
| MOL000908 | beta-elemene | Gamma-aminobutyric-acid receptor subunit alpha-2 | 423 |
| MOL000908 | beta-elemene | Retinoic acid receptor RXR-alpha | 459 |
| MOL000908 | beta-elemene | Sodium-dependent noradrenaline transporter | 540 |
| MOL000908 | beta-elemene | Gamma-aminobutyric-acid receptor subunit alpha-3 | 580 |
| MOL000908 | beta-elemene | M2 | 617 |
| MOL000908 | beta-elemene | Gamma-aminobutyric-acid receptor subunit alpha-1 | 872 |
| MOL000908 | beta-elemene | Gamma-aminobutyric-acid receptor subunit alpha-6 | 841 |
| MOL000908 | beta-elemene | Prostaglandin G/H synthase 1 | 20 |
| MOL000908 | beta-elemene | M3 | 51 |
| MOL000908 | beta-elemene | M1 | 103 |
| MOL000908 | beta-elemene | Alpha-1A adrenergic receptor | 556 |

| | | | | |
|---|---|---|---|---|
| MOL000908 | beta-elemene | Neuronal acetylcholine receptor subunit alpha-7 | 4095 | |
| MOL000908 | beta-elemene | Nuclear receptor coactivator 2 | 6241 | |
| MOL000908 | beta-elemene | Gamma-aminobutyric-acid receptor subunit alpha-5 | 523 | |
| MOL000908 | beta-elemene | Apoptosis regulator Bcl-2 | 273 | validated |
| MOL000908 | beta-elemene | Cyclin-dependent kinase inhibitor 1 | h001 | validated |
| MOL000908 | beta-elemene | Eukaryotic translation initiation factor 6 | h001 | validated |
| MOL000908 | beta-elemene | Retinoblastoma-associated protein | 3846 | validated |
| MOL000908 | beta-elemene | Cellular tumor antigen p53 | 5788 | validated |
| MOL000908 | beta-elemene | Telomerase protein component 1 | h001 | validated |
| MOL000908 | beta-elemene | Protein CBFA2T1 | h001 | validated |
| MOL000908 | beta-elemene | Cell division control protein 2 homolog | 1771 | validated |
| MOL000908 | beta-elemene | G2/mitotic-specific cyclin-B1 | h001 | validated |
| MOL000908 | beta-elemene | Transforming protein RhoA | h001 | validated |
| MOL000916 | Car-3-ene | Cytochrome P450-cam | 2298 | |
| MOL000916 | Car-3-ene | Prostaglandin G/H synthase 2 | 290 | |
| MOL000916 | Car-3-ene | Retinoic acid receptor RXR-alpha | 459 | |
| MOL000916 | Car-3-ene | Nuclear receptor coactivator 2 | 6241 | |
| MOL000098 | quercetin | Prostaglandin G/H synthase 1 | 20 | validated |
| MOL000098 | quercetin | Androgen receptor | 146 | validated |
| MOL000098 | quercetin | Peroxisome proliferator-activated receptor gamma | 238 | |
| MOL000098 | quercetin | Prostaglandin G/H synthase 2 | 290 | validated |
| MOL000098 | quercetin | Heat shock protein HSP 90-alpha | 1939 | |
| MOL000098 | quercetin | Phosphatidylinositol-4,5-bisphosphate 3-kinase catalytic subunit gamma isoform | 2404 | |
| MOL000098 | quercetin | Nuclear receptor coactivator 2 | 6241 | |
| MOL000098 | quercetin | Dipeptidyl peptidase 4 | 952 | |
| MOL000098 | quercetin | Aldose reductase | 822 | |
| MOL000098 | quercetin | Trypsin-1 | 3176 | |
| MOL000098 | quercetin | DNA topoisomerase 2-alpha | 817 | |
| MOL000098 | quercetin | Prothrombin | 54 | |
| MOL000098 | quercetin | Potassium voltage-gated channel subfamily H member 2 | 101 | |
| MOL000098 | quercetin | Sodium channel protein type 5 subunit alpha | 220 | |
| MOL000098 | quercetin | Coagulation factor X | 239 | |
| MOL000098 | quercetin | Beta-2 adrenergic receptor | 766 | |
| MOL000098 | quercetin | Stromelysin-1 | 1926 | |
| MOL000098 | quercetin | cAMP-dependent protein kinase catalytic subunit alpha | 6263 | |
| MOL000098 | quercetin | Coagulation factor VII | 369 | |
| MOL000098 | quercetin | Nitric-oxide synthase, endothelial | 291 | |
| MOL000098 | quercetin | Retinoic acid receptor RXR-alpha | 459 | |
| MOL000098 | quercetin | Acetylcholinesterase | 474 | |
| MOL000098 | quercetin | Gamma-aminobutyric-acid receptor subunit alpha-1 | 872 | |
| MOL000098 | quercetin | Amine oxidase [flavin-containing] B | 3939 | |

| | | | | |
|---|---|---|---|---|
| MOL000098 | quercetin | Transcription factor p65 | h001 | validated |
| MOL000098 | quercetin | Epidermal growth factor receptor | 844 | validated |
| MOL000098 | quercetin | RAC-alpha serine/threonine-protein kinase | h001 | validated |
| MOL000098 | quercetin | | | validated |
| MOL000098 | quercetin | G1/S-specific cyclin-D1 | h001 | validated |
| MOL000098 | quercetin | Apoptosis regulator Bcl-2 | 273 | validated |
| MOL000098 | quercetin | Bcl-2-like protein 1 | h001 | validated |
| MOL000098 | quercetin | Proto-oncogene c-Fos | h001 | validated |
| MOL000098 | quercetin | Cyclin-dependent kinase inhibitor 1 | h001 | validated |
| MOL000098 | quercetin | Eukaryotic translation initiation factor 6 | h001 | validated |
| MOL000098 | quercetin | Apoptosis regulator BAX | h001 | validated |
| MOL000098 | quercetin | Caspase-9 | h001 | validated |
| MOL000098 | quercetin | activator | 1074 | validated |
| MOL000098 | quercetin | 72 kDa type IV collagenase | 707 | validated |
| MOL000098 | quercetin | Matrix metalloproteinase-9 | h001 | validated |
| MOL000098 | quercetin | Mitogen-activated protein kinase 1 | 1176 | validated |
| MOL000098 | quercetin | Interleukin-10 | h001 | validated |
| MOL000098 | quercetin | Retinoblastoma-associated protein | 3846 | validated |
| MOL000098 | quercetin | Tumor necrosis factor | 777 | validated |
| MOL000098 | quercetin | Transcription factor AP-1 | 1629 | validated |
| MOL000098 | quercetin | Interleukin-6 | 1159 | validated |
| MOL000098 | quercetin | Cyclin-dependent kinase inhibitor 2A, isoforms 1/2/3 | h001 | validated |
| MOL000098 | quercetin | Activator of 90 kDa heat shock protein ATPase homolog 1 | h001 | validated |
| MOL000098 | quercetin | Caspase-3 | h001 | validated |
| MOL000098 | quercetin | Cellular tumor antigen p53 | 5788 | validated |
| MOL000098 | quercetin | ETS domain-containing protein Elk-1 | h001 | validated |
| MOL000098 | quercetin | NF-kappa-B inhibitor alpha | h001 | validated |
| MOL000098 | quercetin | Ornithine decarboxylase | 449 | validated |
| MOL000098 | quercetin | Xanthine dehydrogenase/oxidase | 3947 | validated |
| MOL000098 | quercetin | Caspase-8 | h001 | validated |
| MOL000098 | quercetin | DNA topoisomerase 1 | 3552 | validated |
| MOL000098 | quercetin | RAF proto-oncogene serine/threonine-protein kinase | h001 | validated |
| MOL000098 | quercetin | Superoxide dismutase [Cu-Zn] | 4152 | validated |
| MOL000098 | quercetin | Protein kinase C alpha type | h001 | validated |
| MOL000098 | quercetin | Interstitial collagenase | 1167 | validated |
| MOL000098 | quercetin | Hypoxia-inducible factor 1-alpha | h001 | validated |
| MOL000098 | quercetin | Signal transducer and activator of transcription 1-alpha/beta | h001 | validated |
| MOL000098 | quercetin | Protein CBFA2T1 | h001 | validated |
| MOL000098 | quercetin | Probable E3 ubiquitin-protein ligase HERC5 | h001 | validated |
| MOL000098 | quercetin | Cell division control protein 2 homolog | 1771 | validated |
| MOL000098 | quercetin | 78 kDa glucose-regulated protein | 1847 | validated |
| MOL000098 | quercetin | Receptor tyrosine-protein kinase erbB-2 | h001 | validated |

| | | | | |
|---|---|---|---|---|
| MOL000098 | quercetin | Peroxisome proliferator-activated receptor gamma | h001 | validated |
| MOL000098 | quercetin | Acetyl-CoA carboxylase 1 | 690 | validated |
| MOL000098 | quercetin | Heme oxygenase 1 | 3391 | validated |
| MOL000098 | quercetin | Cytochrome P450 3A4 | 4512 | validated |
| MOL000098 | quercetin | Caveolin-1 | h001 | validated |
| MOL000098 | quercetin | Myc proto-oncogene protein | h001 | validated |
| MOL000098 | quercetin | Tissue factor | 2139 | validated |
| MOL000098 | quercetin | Gap junction alpha-1 protein | 1827 | validated |
| MOL000098 | quercetin | Cytochrome P450 1A1 | h001 | validated |
| MOL000098 | quercetin | Intercellular adhesion molecule 1 | h001 | validated |
| MOL000098 | quercetin | Interleukin-1 beta | 1654 | validated |
| MOL000098 | quercetin | Small inducible cytokine A2 | 1649 | validated |
| MOL000098 | quercetin | E-selectin | 1756 | validated |
| MOL000098 | quercetin | Vascular cell adhesion protein 1 | 1908 | validated |
| MOL000098 | quercetin | subtype | 4131 | validated |
| MOL000098 | quercetin | Interleukin-8 | h001 | validated |
| MOL000098 | quercetin | Protein kinase C beta type | h001 | validated |
| MOL000098 | quercetin | Baculoviral IAP repeat-containing protein 5 | h001 | validated |
| MOL000098 | quercetin | Dual oxidase 2 | h001 | validated |
| MOL000098 | quercetin | Nitric oxide synthase, endothelial | h001 | validated |
| MOL000098 | quercetin | Heat shock protein beta-1 | h001 | validated |
| MOL000098 | quercetin | Transforming growth factor beta-1 | h001 | validated |
| MOL000098 | quercetin | Maltase-glucoamylase, intestinal | 929 | validated |
| MOL000098 | quercetin | Interleukin-2 | 1575 | validated |
| MOL000098 | quercetin | Nuclear receptor subfamily 1 group I member 2 | h001 | validated |
| MOL000098 | quercetin | Cytochrome P450 1B1 | h001 | validated |
| MOL000098 | quercetin | G2/mitotic-specific cyclin-B1 | h001 | validated |
| MOL000098 | quercetin | Tissue-type plasminogen activator | 1792 | validated |
| MOL000098 | quercetin | Thrombomodulin | 2021 | validated |
| MOL000098 | quercetin | Plasminogen activator inhibitor 1 | h001 | validated |
| MOL000098 | quercetin | Interferon gamma | 1253 | validated |
| MOL000098 | quercetin | Arachidonate 5-lipoxygenase | 275 | validated |
| MOL000098 | quercetin | Phosphatidylinositol-3,4,5-trisphosphate 3-phosphatase and dual-specificity protein | h001 | validated |
| MOL000098 | quercetin | Interleukin-1 alpha | h001 | validated |
| MOL000098 | quercetin | Myeloperoxidase | 1757 | validated |
| MOL000098 | quercetin | DNA topoisomerase 2-alpha | h001 | validated |
| MOL000098 | quercetin | Neutrophil cytosol factor 1 | h001 | validated |
| MOL000098 | quercetin | ATP-binding cassette sub-family G member 2 | h001 | validated |
| MOL000098 | quercetin | Hyaluronan synthase 2 | h001 | validated |
| MOL000098 | quercetin | Nuclear factor erythroid 2-related factor 2 | h001 | validated |
| MOL000098 | quercetin | NAD(P)H dehydrogenase [quinone] 1 | 2157 | validated |
| MOL000098 | quercetin | Poly [ADP-ribose] polymerase 1 | h001 | validated |
| MOL000098 | quercetin | Aryl hydrocarbon receptor | 6220 | validated |
| MOL000098 | quercetin | 26S proteasome non-ATPase regulatory subunit 3 | h001 | validated |

| | | | | |
|---|---|---|---|---|
| MOL000098 | quercetin | Solute carrier family 2, facilitated glucose transporter | h001 | validated |
| MOL000098 | quercetin | Collagen alpha-1(III) chain | 3853 | validated |
| MOL000098 | quercetin | DNA gyrase subunit B | 4150 | validated |
| MOL000098 | quercetin | C-X-C motif chemokine 11 | h001 | validated |
| MOL000098 | quercetin | C-X-C motif chemokine 2 | h001 | validated |
| MOL000098 | quercetin | DDB1- and CUL4-associated factor 5 | h001 | validated |
| MOL000098 | quercetin | Nuclear receptor subfamily 1 group I member 3 | h001 | validated |
| MOL000098 | quercetin | Chk2 | h001 | validated |
| MOL000098 | quercetin | Insulin receptor | 36 | validated |
| MOL000098 | quercetin | Claudin-4 | h001 | validated |
| MOL000098 | quercetin | Peroxisome proliferator-activated receptor alpha | h001 | validated |
| MOL000098 | quercetin | Peroxisome proliferator-activated receptor delta | h001 | validated |
| MOL000098 | quercetin | Heat shock factor protein 1 | h001 | validated |
| MOL000098 | quercetin | C-reactive protein | h001 | validated |
| MOL000098 | quercetin | C-X-C motif chemokine 10 | h001 | validated |
| MOL000098 | quercetin | Inhibitor of nuclear factor kappa-B kinase subunit alpha | h001 | validated |
| MOL000098 | quercetin | Osteopontin | h001 | validated |
| MOL000098 | quercetin | Runt-related transcription factor 2 | h001 | validated |
| MOL000098 | quercetin | Ras association domain-containing protein 1 | h001 | validated |
| MOL000098 | quercetin | Transcription factor E2F1 | h001 | validated |
| MOL000098 | quercetin | Transcription factor E2F2 | h001 | validated |
| MOL000098 | quercetin | Prostatic acid phosphatase | 1859 | validated |
| MOL000098 | quercetin | Cathepsin D | 1243 | validated |
| MOL000098 | quercetin | Insulin-like growth factor-binding protein 3 | h001 | validated |
| MOL000098 | quercetin | Insulin-like growth factor II | h001 | validated |
| MOL000098 | quercetin | CD40 ligand | h001 | validated |
| MOL000098 | quercetin | Interferon regulatory factor 1 | h001 | validated |
| MOL000098 | quercetin | Receptor tyrosine-protein kinase erbB-3 | h001 | validated |
| MOL000098 | quercetin | Serum paraoxonase/arylesterase 1 | 1198 | validated |
| MOL000098 | quercetin | Type I iodothyronine deiodinase | h001 | validated |
| MOL000098 | quercetin | enhancer 1 | h001 | validated |
| MOL000098 | quercetin | Puromycin-sensitive aminopeptidase | h001 | validated |
| MOL000098 | quercetin | Hexokinase-2 | h001 | validated |
| MOL000098 | quercetin | Homeobox protein Nkx-3.1 | h001 | validated |
| MOL000098 | quercetin | Ras GTPase-activating protein 1 | h001 | validated |
| MOL000098 | quercetin | Peroxidase C1A | h001 | validated |
| MOL000098 | quercetin | Glutathione S-transferase Mu 1 | 896 | validated |
| MOL000098 | quercetin | Glutathione S-transferase Mu 2 | 2165 | validated |
