# Supplementary material for: The Study of Yin-Chen-Hao-Tang Preventing and Treating Alcoholic Fatty Liver Disease through PPAR Signaling Pathway Based on Network Pharmacology and RNA-Seq Transcriptomics
Source: Evid Based Complement Alternat Med. 2021 Dec 31;2021:8917993. doi: 10.1155/2021/8917993 (PMC8741355; doi:10.1155/2021/8917993)
Supplement: Supplementary Materials — Supplementary Material 1-1: Herb Target-Artemisiae Scopariae Herba. Supplementary Material 1-2: Herb Target-Gardeniae Fructus. Supplementary Material 1-3: Herb Target-Radix Rhei Et Rhizome. Supplementary Material 2: AFLD-GeneCards-SearchResults. Supplementary Material 3: string_interactions.tsv default node. [file 8917993.f1.zip › 8917993.f1/Supplementary material 1-2 Herb Target--Gardeniae Fructus.pdf]

Mol ID	Molecule Name	Target Name	drugbank	Status
MOL001406	crocetin	Cytochrome P450-cam	2298	
MOL001406	crocetin	Muscarinic acetylcholine receptor M3	51	
MOL001406	crocetin	Muscarinic acetylcholine receptor M1	103	
MOL001406	crocetin	Gamma-aminobutyric-acid receptor subunit alpha-2	423	
MOL001406	crocetin	Gamma-aminobutyric-acid receptor subunit alpha-5	523	
MOL001406	crocetin	Alpha-1A adrenergic receptor	556	
MOL001406	crocetin	Gamma-aminobutyric-acid receptor subunit alpha-3	580	
MOL001406	crocetin	Muscarinic acetylcholine receptor M2	617	
MOL001406	crocetin	Alpha-1B adrenergic receptor	632	
MOL001406	crocetin	Gamma-aminobutyric-acid receptor subunit alpha-1	872	
MOL001406	crocetin	Ig gamma-1 chain C region	4785	
MOL001406	crocetin	Prostaglandin G/H synthase 2	290	
MOL001406	crocetin	Nuclear receptor coactivator 2	6241	
MOL001406	crocetin	Vascular cell adhesion protein 1	1908	validated
MOL001648	genipin	Prostaglandin G/H synthase 1	20	
MOL001648	genipin	Prostaglandin G/H synthase 2	290	
MOL001648	genipin	Carbonic anhydrase 2	357	
MOL001648	genipin	Gamma-aminobutyric-acid receptor subunit alpha-2	423	
MOL001648	genipin	Gamma-aminobutyric-acid receptor subunit alpha-1	872	
MOL001648	genipin	Thymidine kinase	2559	
MOL001648	genipin	Trypsin-1	3176	
MOL001648	genipin	Glutamate receptor 2	921	
MOL001648	genipin	Gamma-aminobutyric-acid receptor subunit alpha-6	841	
MOL001648	genipin	Canalicular multispecific organic anion transporter 1	h001	validated
MOL001941	Ammidin	Prothrombin	54	
MOL001941	Ammidin	Muscarinic acetylcholine receptor M1	103	
MOL001941	Ammidin	Prostaglandin G/H synthase 2	290	
MOL001941	Ammidin	Gamma-aminobutyric-acid receptor subunit alpha-1	872	
MOL001941	Ammidin	Dipeptidyl peptidase 4	952	

MOL001941	Ammidin	Phosphatidylinositol-4,5-bisphosphate 3-kinase catalytic subunit gamma isoform	2404	
MOL001941	Ammidin	cAMP-dependent protein kinase catalytic subunit alpha	6263	
MOL001941	Ammidin	Amine oxidase [flavin-containing] B	3939	
MOL000437	Hirsutrin	DNA topoisomerase 2-alpha	817	
MOL000437	Hirsutrin	Tyrosine-protein phosphatase non-receptor type 1	687	
MOL000437	Hirsutrin	Coagulation factor X	239	
MOL004556	crocin	Sphingomyelin phosphodiesterase 2	h001	validated
MOL004557	geniposide	Carbonic anhydrase 2	357	
MOL004557	geniposide	Apoptosis regulator Bcl-2	273	validated
MOL004557	geniposide	Heme oxygenase 1	3391	validated
MOL004557	geniposide	Neuromodulin	h001	validated
MOL004557	geniposide	Phospholipase B1, membrane-associated	h001	validated
MOL004557	geniposide	Glucagon	h001	validated
MOL004557	geniposide	Glutathione S-transferase Mu 1	896	validated
MOL004557	geniposide	Glutathione S-transferase Mu 2	2165	validated
MOL000415	rutin	DNA topoisomerase 2-alpha	817	
MOL000415	rutin	Transcription factor p65	h001	validated
MOL000415	rutin	Tumor necrosis factor	777	validated
MOL000415	rutin	Interleukin-6	1159	validated
MOL000415	rutin	Caspase-3	h001	validated
MOL000415	rutin			validated
MOL000415	rutin	Superoxide dismutase [Cu-Zn]	4152	validated
MOL000415	rutin	Interleukin-1 beta	1654	validated
MOL000415	rutin	Interleukin-8	h001	validated
MOL000415	rutin	Protein kinase C beta type	h001	validated
MOL000415	rutin	Arachidonate 5-lipoxygenase	275	validated
MOL000415	rutin	3-hydroxy-3-methylglutaryl-coenzyme A reductase	3387	validated
MOL000415	rutin	Hyaluronan synthase 2	h001	validated
MOL000415	rutin	Type I iodothyronine deiodinase	h001	validated
MOL000415	rutin	C5a anaphylatoxin chemotactic receptor	h001	validated
MOL000415	rutin	Insulin	5961	validated
MOL000415	rutin	Low affinity immunoglobulin epsilon Fc receptor	h001	validated
MOL000415	rutin	Integrin beta-2	1630	validated
MOL000415	rutin	Thromboxane A2 receptor	835	validated

MOL004559	(1S,4aS,5R,7S,7aS)-5,7-dihydroxy-7-methyl-1-[(2S,3R,4S,5S,6R)-3,4,5-trihydroxy-6-(hydroxymethyl)oxan-2-yl]oxy-4a,5,6,7a-tetrahydro-1H-cyclopenta[d]pyran-4-carboxylic acid	Carbonic anhydrase 2	357	
MOL004559	(1S,4aS,5R,7S,7aS)-5,7-dihydroxy-7-methyl-1-[(2S,3R,4S,5S,6R)-3,4,5-trihydroxy-6-(hydroxymethyl)oxan-2-yl]oxy-4a,5,6,7a-tetrahydro-1H-cyclopenta[d]pyran-4-carboxylic acid	Dipeptidyl peptidase 4	952	
MOL004560	SHANZHISIDE_qt	Trypsin-1	3176	
MOL004560	SHANZHISIDE_qt	Glutamate receptor 2	921	
MOL004561	Sudan III	Prothrombin	54	
MOL004561	Sudan III	Estrogen receptor	136	
MOL004561	Sudan III	Prostaglandin G/H synthase 2	290	
MOL004561	Sudan III	Coagulation factor VII	369	
MOL004561	Sudan III	Estrogen receptor beta	869	
MOL004561	Sudan III	Dipeptidyl peptidase 4	952	
MOL004561	Sudan III	Mitogen-activated protein kinase 14	1540	
MOL004561	Sudan III	Glycogen synthase kinase-3 beta	1721	
MOL004561	Sudan III	Mitogen-activated protein kinase 10	1836	
MOL004561	Sudan III	Cell division protein kinase 2	2240	
MOL004561	Sudan III	cAMP-dependent protein kinase catalytic subunit alpha	6263	
MOL004561	Sudan III	Proto-oncogene serine/threonine-protein kinase Pim-1	2347	
MOL004561	Sudan III	Cyclin-A2	6235	
MOL000511	ursolic acid	Urokinase-type plasminogen activator	1074	validated
MOL000511	ursolic acid	Cathepsin B	1233	validated
MOL000511	ursolic acid	Transcription factor p65	h001	validated
MOL000511	ursolic acid	Signal transducer and activator of transcription 3	h001	validated
MOL000511	ursolic acid			validated
MOL000511	ursolic acid	G1/S-specific cyclin-D1	h001	validated
MOL000511	ursolic acid	Apoptosis regulator Bcl-2	273	validated
MOL000511	ursolic acid	Bcl-2-like protein 1	h001	validated

MOL000511	ursolic acid	Proto-oncogene c-Fos	h001	validated
MOL000511	ursolic acid	Cyclin-dependent kinase inhibitor 1	h001	validated
MOL000511	ursolic acid	Apoptosis regulator BAX	h001	validated
MOL000511	ursolic acid	Caspase-9	h001	validated
MOL000511	ursolic acid	72 kDa type IV collagenase	707	validated
MOL000511	ursolic acid	Matrix metalloproteinase-9	h001	validated
MOL000511	ursolic acid	Cell division protein kinase 4	3959	validated
MOL000511	ursolic acid	Tumor necrosis factor	777	validated
MOL000511	ursolic acid	Transcription factor AP-1	1629	validated
MOL000511	ursolic acid	Interleukin-6	1159	validated
MOL000511	ursolic acid	Cell division protein kinase 6	3955	validated
MOL000511	ursolic acid	Caspase-3	h001	validated
MOL000511	ursolic acid	Cellular tumor antigen p53	5788	validated
MOL000511	ursolic acid	Mitogen-activated protein kinase 8	6292	validated
MOL000511	ursolic acid	Prostaglandin G/H synthase 2	290	validated
MOL000511	ursolic acid	NF-kappa-B inhibitor alpha	h001	validated
MOL000511	ursolic acid	Caspase-8	h001	validated
MOL000511	ursolic acid	Fatty acid synthase	1295	validated
MOL000511	ursolic acid	Interstitial collagenase	1167	validated
MOL000511	ursolic acid	Stromelysin-1	1926	validated
MOL000511	ursolic acid	Probable E3 ubiquitin-protein ligase HERC5	h001	validated
MOL000511	ursolic acid	Heparin-binding growth factor 2	1525	validated
MOL000511	ursolic acid	Intercellular adhesion molecule 1	h001	validated
MOL000511	ursolic acid	Interleukin-1 beta	1654	validated
MOL000511	ursolic acid	Cyclic AMP-responsive element-binding protein 1	h001	validated
MOL000511	ursolic acid	E-selectin	1756	validated
MOL000511	ursolic acid	Prostaglandin E2 receptor, EP3 subtype	4131	validated
MOL000511	ursolic acid	Prostaglandin G/H synthase 1	20	validated
MOL000511	ursolic acid	Induced myeloid leukemia cell differentiation protein Mcl-1	h001	validated
MOL000511	ursolic acid	Protein kinase C gamma type	h001	validated
MOL000511	ursolic acid	Cyclic AMP-dependent transcription factor ATF-2	h001	validated
MOL000511	ursolic acid	Granulocyte-macrophage colony-stimulating factor	h001	validated
MOL000511	ursolic acid	Platelet endothelial cell adhesion molecule	h001	validated
MOL000511	ursolic acid	C-Jun-amino-terminal kinase-interacting protein 2	h001	validated

MOL000511	ursolic acid	Baculoviral IAP repeat-containing protein 5	h001	validated
MOL000511	ursolic acid	Tyrosine-protein phosphatase non-receptor type 6	h001	validated
MOL000511	ursolic acid	Neuromodulin	h001	validated
MOL000511	ursolic acid	Dual oxidase 2	h001	validated
MOL000511	ursolic acid	Nitric oxide synthase, endothelial	h001	validated
MOL000511	ursolic acid	Tyrosine-protein phosphatase non-receptor type 1	h001	validated
MOL000511	ursolic acid	Phosphatidylinositol-3,4,5-trisphosphate 5-phosphatase 2	h001	validated
MOL000511	ursolic acid	Lipopolysaccharide-induced tumor necrosis factor-alpha factor	h001	validated
MOL000511	ursolic acid	G1/S-specific cyclin-D2	h001	validated
MOL000511	ursolic acid	Tumor necrosis factor ligand superfamily member 6	h001	validated
MOL000511	ursolic acid	Caspase-1	h001	validated
MOL000511	ursolic acid	Ectonucleotide pyrophosphatase/phosphodiesterase family member 7	h001	validated
MOL000098	quercetin	Prostaglandin G/H synthase 1	20	validated
MOL000098	quercetin	Androgen receptor	146	validated
MOL000098	quercetin	Peroxisome proliferator-activated receptor gamma	238	
MOL000098	quercetin	Prostaglandin G/H synthase 2	290	validated
MOL000098	quercetin	Heat shock protein HSP 90-alpha	1939	
MOL000098	quercetin	Phosphatidylinositol-4,5-bisphosphate 3-kinase catalytic subunit gamma isoform	2404	
MOL000098	quercetin	Nuclear receptor coactivator 2	6241	
MOL000098	quercetin	Dipeptidyl peptidase 4	952	
MOL000098	quercetin	Aldose reductase	822	
MOL000098	quercetin	Trypsin-1	3176	
MOL000098	quercetin	DNA topoisomerase 2-alpha	817	
MOL000098	quercetin	Prothrombin	54	
MOL000098	quercetin	Potassium voltage-gated channel subfamily H member 2	101	
MOL000098	quercetin	Sodium channel protein type 5 subunit alpha	220	
MOL000098	quercetin	Coagulation factor X	239	
MOL000098	quercetin	Beta-2 adrenergic receptor	766	
MOL000098	quercetin	Stromelysin-1	1926	

MOL000098	quercetin	cAMP-dependent protein kinase catalytic subunit alpha	6263	
MOL000098	quercetin	Coagulation factor VII	369	
MOL000098	quercetin	Nitric-oxide synthase, endothelial	291	
MOL000098	quercetin	Retinoic acid receptor RXR-alpha	459	
MOL000098	quercetin	Acetylcholinesterase	474	
MOL000098	quercetin	Gamma-aminobutyric-acid receptor subunit alpha-1	872	
MOL000098	quercetin	Amine oxidase [flavin-containing] B	3939	
MOL000098	quercetin	Transcription factor p65	h001	validated
MOL000098	quercetin	Epidermal growth factor receptor	844	validated
MOL000098	quercetin	RAC-alpha serine/threonine-protein kinase	h001	validated
MOL000098	quercetin			validated
MOL000098	quercetin	G1/S-specific cyclin-D1	h001	validated
MOL000098	quercetin	Apoptosis regulator Bcl-2	273	validated
MOL000098	quercetin	Bcl-2-like protein 1	h001	validated
MOL000098	quercetin	Proto-oncogene c-Fos	h001	validated
MOL000098	quercetin	Cyclin-dependent kinase inhibitor 1	h001	validated
MOL000098	quercetin	Eukaryotic translation initiation factor 6	h001	validated
MOL000098	quercetin	Apoptosis regulator BAX	h001	validated
MOL000098	quercetin	Caspase-9	h001	validated
MOL000098	quercetin	Urokinase-type plasminogen activator	1074	validated
MOL000098	quercetin	72 kDa type IV collagenase	707	validated
MOL000098	quercetin	Matrix metalloproteinase-9	h001	validated
MOL000098	quercetin	Mitogen-activated protein kinase 1	1176	validated
MOL000098	quercetin	Interleukin-10	h001	validated
MOL000098	quercetin	Retinoblastoma-associated protein	3846	validated
MOL000098	quercetin	Tumor necrosis factor	777	validated
MOL000098	quercetin	Transcription factor AP-1	1629	validated
MOL000098	quercetin	Interleukin-6	1159	validated
MOL000098	quercetin	Cyclin-dependent kinase inhibitor 2A, isoforms 1/2/3	h001	validated
MOL000098	quercetin	Activator of 90 kDa heat shock protein ATPase homolog 1	h001	validated
MOL000098	quercetin	Caspase-3	h001	validated
MOL000098	quercetin	Cellular tumor antigen p53	5788	validated
MOL000098	quercetin	ETS domain-containing protein Elk-1	h001	validated
MOL000098	quercetin	NF-kappa-B inhibitor alpha	h001	validated
MOL000098	quercetin	Ornithine decarboxylase	449	validated

MOL000098	quercetin	Xanthine dehydrogenase/oxidase	3947	validated
MOL000098	quercetin	Caspase-8	h001	validated
MOL000098	quercetin	DNA topoisomerase 1	3552	validated
MOL000098	quercetin	RAF proto-oncogene serine/threonine-protein kinase	h001	validated
MOL000098	quercetin	Superoxide dismutase [Cu-Zn]	4152	validated
MOL000098	quercetin	Protein kinase C alpha type	h001	validated
MOL000098	quercetin	Interstitial collagenase	1167	validated
MOL000098	quercetin	Hypoxia-inducible factor 1-alpha	h001	validated
MOL000098	quercetin	Signal transducer and activator of transcription 1-alpha/beta	h001	validated
MOL000098	quercetin	Protein CBFA2T1	h001	validated
MOL000098	quercetin	Probable E3 ubiquitin-protein ligase HERC5	h001	validated
MOL000098	quercetin	Cell division control protein 2 homolog	1771	validated
MOL000098	quercetin	78 kDa glucose-regulated protein	1847	validated
MOL000098	quercetin	Receptor tyrosine-protein kinase erbB-2	h001	validated
MOL000098	quercetin	Peroxisome proliferator-activated receptor gamma	h001	validated
MOL000098	quercetin	Acetyl-CoA carboxylase 1	690	validated
MOL000098	quercetin	Heme oxygenase 1	3391	validated
MOL000098	quercetin	Cytochrome P450 3A4	4512	validated
MOL000098	quercetin	Caveolin-1	h001	validated
MOL000098	quercetin	Myc proto-oncogene protein	h001	validated
MOL000098	quercetin	Tissue factor	2139	validated
MOL000098	quercetin	Gap junction alpha-1 protein	1827	validated
MOL000098	quercetin	Cytochrome P450 1A1	h001	validated
MOL000098	quercetin	Intercellular adhesion molecule 1	h001	validated
MOL000098	quercetin	Interleukin-1 beta	1654	validated
MOL000098	quercetin	Small inducible cytokine A2	1649	validated
MOL000098	quercetin	E-selectin	1756	validated
MOL000098	quercetin	Vascular cell adhesion protein 1	1908	validated
MOL000098	quercetin	Prostaglandin E2 receptor, EP3 subtype	4131	validated
MOL000098	quercetin	Interleukin-8	h001	validated
MOL000098	quercetin	Protein kinase C beta type	h001	validated
MOL000098	quercetin	Baculoviral IAP repeat-containing protein 5	h001	validated
MOL000098	quercetin	Dual oxidase 2	h001	validated
MOL000098	quercetin	Nitric oxide synthase, endothelial	h001	validated
MOL000098	quercetin	Heat shock protein beta-1	h001	validated

MOL000098	quercetin	Transforming growth factor beta-1	h001	validated
MOL000098	quercetin	Maltase-glucoamylase, intestinal	929	validated
MOL000098	quercetin	Interleukin-2	1575	validated
MOL000098	quercetin	Nuclear receptor subfamily 1 group I member 2	h001	validated
MOL000098	quercetin	Cytochrome P450 1B1	h001	validated
MOL000098	quercetin	G2/mitotic-specific cyclin-B1	h001	validated
MOL000098	quercetin	Tissue-type plasminogen activator	1792	validated
MOL000098	quercetin	Thrombomodulin	2021	validated
MOL000098	quercetin	Plasminogen activator inhibitor 1	h001	validated
MOL000098	quercetin	Interferon gamma	1253	validated
MOL000098	quercetin	Arachidonate 5-lipoxygenase	275	validated
MOL000098	quercetin	Phosphatidylinositol-3,4,5-trisphosphate 3-phosphatase and dual-specificity protein phosphatase PTEN	h001	validated
MOL000098	quercetin	Interleukin-1 alpha	h001	validated
MOL000098	quercetin	Myeloperoxidase	1757	validated
MOL000098	quercetin	DNA topoisomerase 2-alpha	h001	validated
MOL000098	quercetin	Neutrophil cytosol factor 1	h001	validated
MOL000098	quercetin	ATP-binding cassette sub-family G member 2	h001	validated
MOL000098	quercetin	Hyaluronan synthase 2	h001	validated
MOL000098	quercetin	Nuclear factor erythroid 2-related factor 2	h001	validated
MOL000098	quercetin	NAD(P)H dehydrogenase [quinone] 1	2157	validated
MOL000098	quercetin	Poly [ADP-ribose] polymerase 1	h001	validated
MOL000098	quercetin	Aryl hydrocarbon receptor	6220	validated
MOL000098	quercetin	26S proteasome non-ATPase regulatory subunit 3	h001	validated
MOL000098	quercetin	Solute carrier family 2, facilitated glucose transporter member 4	h001	validated
MOL000098	quercetin	Collagen alpha-1(III) chain	3853	validated
MOL000098	quercetin	DNA gyrase subunit B	4150	validated
MOL000098	quercetin	C-X-C motif chemokine 11	h001	validated
MOL000098	quercetin	C-X-C motif chemokine 2	h001	validated
MOL000098	quercetin	DDB1- and CUL4-associated factor 5	h001	validated
MOL000098	quercetin	Nuclear receptor subfamily 1 group I member 3	h001	validated
MOL000098	quercetin	Serine/threonine-protein kinase Chk2	h001	validated
MOL000098	quercetin	Insulin receptor	36	validated
MOL000098	quercetin	Claudin-4	h001	validated

MOL000098	quercetin	Peroxisome proliferator-activated receptor alpha	h001	validated
MOL000098	quercetin	Peroxisome proliferator-activated receptor delta	h001	validated
MOL000098	quercetin	Heat shock factor protein 1	h001	validated
MOL000098	quercetin	C-reactive protein	h001	validated
MOL000098	quercetin	C-X-C motif chemokine 10	h001	validated
MOL000098	quercetin	Inhibitor of nuclear factor kappa-B kinase subunit alpha	h001	validated
MOL000098	quercetin	Osteopontin	h001	validated
MOL000098	quercetin	Runt-related transcription factor 2	h001	validated
MOL000098	quercetin	Ras association domain-containing protein 1	h001	validated
MOL000098	quercetin	Transcription factor E2F1	h001	validated
MOL000098	quercetin	Transcription factor E2F2	h001	validated
MOL000098	quercetin	Prostatic acid phosphatase	1859	validated
MOL000098	quercetin	Cathepsin D	1243	validated
MOL000098	quercetin	Insulin-like growth factor-binding protein 3	h001	validated
MOL000098	quercetin	Insulin-like growth factor II	h001	validated
MOL000098	quercetin	CD40 ligand	h001	validated
MOL000098	quercetin	Interferon regulatory factor 1	h001	validated
MOL000098	quercetin	Receptor tyrosine-protein kinase erbB-3	h001	validated
MOL000098	quercetin	Serum paraoxonase/arylesterase 1	1198	validated
MOL000098	quercetin	Type I iodothyronine deiodinase	h001	validated
MOL000098	quercetin	Procollagen C-endopeptidase enhancer 1	h001	validated
MOL000098	quercetin	Puromycin-sensitive aminopeptidase	h001	validated
MOL000098	quercetin	Hexokinase-2	h001	validated
MOL000098	quercetin	Homeobox protein Nkx-3.1	h001	validated
MOL000098	quercetin	Ras GTPase-activating protein 1	h001	validated
MOL000098	quercetin	Peroxidase C1A	h001	validated
MOL000098	quercetin	Glutathione S-transferase Mu 1	896	validated
MOL000098	quercetin	Glutathione S-transferase Mu 2	2165	validated
MOL000003	MTL			
MOL000003	MTL	Cytochrome P450-cam	2298	
MOL000023	Hemo-sol	Prostaglandin G/H synthase 2	290	
MOL000023	Hemo-sol	Gamma-aminobutyric-acid receptor subunit alpha-1	872	
MOL000023	Hemo-sol			
MOL000023	Hemo-sol			

MOL000023	Hemo-sol	Cytochrome P450-cam	2298
MOL000023	Hemo-sol	Nuclear receptor coactivator 2	6241
MOL000023	Hemo-sol	Nuclear receptor coactivator 1	6228
MOL000023	Hemo-sol	Muscarinic acetylcholine receptor M2	617
MOL000023	Hemo-sol	Gamma-aminobutyric-acid receptor subunit alpha-2	423
MOL000023	Hemo-sol	Muscarinic acetylcholine receptor M1	103
MOL000023	Hemo-sol	Gamma-aminobutyric-acid receptor subunit alpha-5	523
MOL000023	Hemo-sol	Gamma-aminobutyric-acid receptor subunit alpha-3	580
MOL000023	Hemo-sol	Ig gamma-1 chain C region	4785
MOL000023	Hemo-sol	Gamma-aminobutyric-acid receptor subunit alpha-6	841
MOL000035	beta-Selinene	Prostaglandin G/H synthase 1	20
MOL000035	beta-Selinene	Muscarinic acetylcholine receptor M3	51
MOL000035	beta-Selinene	Muscarinic acetylcholine receptor M1	103
MOL000035	beta-Selinene	Prostaglandin G/H synthase 2	290
MOL000035	beta-Selinene	Gamma-aminobutyric-acid receptor subunit alpha-2	423
MOL000035	beta-Selinene	Retinoic acid receptor RXR-alpha	459
MOL000035	beta-Selinene	Sodium-dependent noradrenaline transporter	540
MOL000035	beta-Selinene	Gamma-aminobutyric-acid receptor subunit alpha-3	580
MOL000035	beta-Selinene	Muscarinic acetylcholine receptor M2	617
MOL000035	beta-Selinene	Alpha-1B adrenergic receptor	632
MOL000035	beta-Selinene	Gamma-aminobutyric-acid receptor subunit alpha-1	872
MOL000035	beta-Selinene	Nuclear receptor coactivator 2	6241
MOL000035	beta-Selinene	Gamma-aminobutyric-acid receptor subunit alpha-6	841
MOL000105	protocatechuic acid	Prostaglandin G/H synthase 1	20
MOL000105	protocatechuic acid	Arachidonate 5-lipoxygenase	275
MOL000105	protocatechuic acid	Prostaglandin G/H synthase 2	290
MOL000105	protocatechuic acid	Amine oxidase [flavin-containing] B	3939
MOL000105	protocatechuic acid	Lysozyme	2300

MOL000105	protocatechuic acid	Nicotinate-nucleotide--dimethylbenzimidazole phosphoribosyltransferase	2264	
MOL000105	protocatechuic acid			
MOL000105	protocatechuic acid	Trypsin-3	2886	
MOL000105	protocatechuic acid	Protein kinase C alpha type	h001	validated
MOL000105	protocatechuic acid	Protein kinase C beta type	h001	validated
MOL000105	protocatechuic acid	Protein kinase C gamma type	h001	validated
MOL000105	protocatechuic acid	Maltase-glucoamylase, intestinal	929	validated
MOL000105	protocatechuic acid	Protein kinase C zeta type	h001	validated
MOL000116	Nonanal			
MOL000116	Nonanal			
MOL000116	Nonanal	Gamma-aminobutyric-acid receptor subunit alpha-2	423	
MOL000116	Nonanal	Gamma-aminobutyric-acid receptor subunit alpha-1	872	
MOL000116	Nonanal	Cytochrome P450-cam	2298	
MOL000120	dec-2-enal			
MOL000120	dec-2-enal			
MOL000131	EIC	Prostaglandin G/H synthase 1	20	
MOL000131	EIC	Prostaglandin G/H synthase 2	290	
MOL000131	EIC	Retinoic acid receptor RXR-alpha	459	
MOL000131	EIC	Nuclear receptor coactivator 2	6241	
MOL000131	EIC	Lysozyme	2300	
MOL000131	EIC	Nicotinate-nucleotide--dimethylbenzimidazole phosphoribosyltransferase	2264	
MOL000131	EIC	Sodium-dependent noradrenaline transporter	540	
MOL000131	EIC	Ig gamma-1 chain C region	4785	
MOL000131	EIC	Gamma-aminobutyric-acid receptor subunit alpha-2	423	
MOL000131	EIC	Gamma-aminobutyric-acid receptor subunit alpha-1	872	
MOL000131	EIC	Cytochrome P450-cam	2298	
MOL000131	EIC	Transient receptor potential cation channel subfamily V member 1	1558	
MOL000131	EIC	Muscarinic acetylcholine receptor M1	103	
MOL000131	EIC	Muscarinic acetylcholine receptor M2	617	
MOL000131	EIC	Gamma-aminobutyric-acid receptor subunit alpha-6	841	
MOL000223	caffeic acid	Prostaglandin G/H synthase 1	20	validated
MOL000223	caffeic acid	Beta-1 adrenergic receptor	193	

MOL000223	caffeic acid	Prostaglandin G/H synthase 2	290	validated
MOL000223	caffeic acid	Alpha-2A adrenergic receptor	318	
MOL000223	caffeic acid	Alpha-2C adrenergic receptor	378	
MOL000223	caffeic acid	Beta-2 adrenergic receptor	766	
MOL000223	caffeic acid	Amine oxidase [flavin-containing] B	3939	
MOL000223	caffeic acid	Chymotrypsinogen B	6011	
MOL000223	caffeic acid	Sodium-dependent dopamine transporter	713	
MOL000223	caffeic acid	Aldose reductase	822	
MOL000223	caffeic acid	Urokinase-type plasminogen activator	1074	
MOL000223	caffeic acid	Amine oxidase [flavin-containing] A	3941	
MOL000223	caffeic acid	Lysozyme	2300	
MOL000223	caffeic acid	Alpha-1A adrenergic receptor	556	
MOL000223	caffeic acid	Alpha-1D adrenergic receptor	789	
MOL000223	caffeic acid	Tumor necrosis factor	777	validated
MOL000223	caffeic acid	Cytochrome P450 1A1	h001	validated
MOL000223	caffeic acid	Protein kinase C beta type	h001	validated
MOL000223	caffeic acid	Tyrosine-protein kinase BTK	2355	validated
MOL000223	caffeic acid	Glial fibrillary acidic protein	h001	validated
MOL000223	caffeic acid	Insulin-like growth factor II	h001	validated
MOL000223	caffeic acid	P-selectin	2058	validated
MOL000223	caffeic acid	Ras-related C3 botulinum toxin substrate 1	1882	validated
MOL000252	farnesol	Prostaglandin G/H synthase 2	290	
MOL000252	farnesol	Sodium-dependent noradrenaline transporter	540	
MOL000252	farnesol	Amine oxidase [flavin-containing] B	3939	
MOL000252	farnesol	Nuclear receptor coactivator 2	6241	
MOL000252	farnesol	Prostaglandin G/H synthase 1	20	
MOL000252	farnesol	Retinoic acid receptor RXR-alpha	459	
MOL000252	farnesol	Interleukin-6	1159	validated
MOL000252	farnesol	Caspase-3	h001	validated
MOL000252	farnesol	Involucrin	h001	validated
MOL000252	farnesol	Ras-specific guanine nucleotide-releasing factor 2	h001	validated
MOL000252	farnesol	Bcl-2 homologous antagonist/killer	h001	validated

MOL000252	farnesol	Toll-like receptor 4	4210	validated
MOL000252	farnesol	Lipoprotein lipase	266	validated
MOL000252	farnesol	3-hydroxy-3-methylglutaryl-coenzyme A reductase	3387	validated
MOL000252	farnesol	Peroxisome proliferator-activated receptor alpha	h001	validated
MOL000252	farnesol	Toll-like receptor 2	2090	validated
MOL000252	farnesol	Beta-defensin 2	h001	validated
MOL000252	farnesol	Protein HIRA	h001	validated
MOL000252	farnesol	SERTA domain-containing protein 3	h001	validated
MOL000252	farnesol	Progesterone receptor	614	validated
MOL000252	farnesol	Bile acid receptor	3233	validated
MOL000252	farnesol	Glutaminase liver isoform, mitochondrial	5	validated
MOL000252	farnesol	Ig delta chain C region	h001	validated
MOL000263	oleanolic acid	Caspase-9	h001	validated
MOL000263	oleanolic acid	Caspase-3	h001	validated
MOL000263	oleanolic acid	Heme oxygenase 1	3391	validated
MOL000263	oleanolic acid	Intercellular adhesion molecule 1	h001	validated
MOL000263	oleanolic acid	NAD(P)H dehydrogenase [quinone] 1	2157	validated
MOL000263	oleanolic acid	Pancreatic alpha-amylase	4513	validated
MOL000305	lauric acid	Prostaglandin G/H synthase 1	20	
MOL000305	lauric acid	Cholinesterase	3923	
MOL000305	lauric acid	Phospholipase A2	6500	
MOL000305	lauric acid			
MOL000305	lauric acid			
MOL000305	lauric acid	Ig gamma-1 chain C region	4785	
MOL000305	lauric acid	Ferrichrome-iron receptor	2427	
MOL000305	lauric acid	3-oxoacyl-[acyl-carrier-protein] synthase 1	889	
MOL000305	lauric acid	Prostaglandin G/H synthase 2	290	validated
MOL000305	lauric acid	Genome polyprotein	6521	
MOL000305	lauric acid	Transcription factor p65	h001	validated
MOL000305	lauric acid	RAC-alpha serine/threonine-protein kinase	h001	validated
MOL000305	lauric acid	Interleukin-6	1159	validated
MOL000305	lauric acid	Interleukin-8	h001	validated
MOL000305	lauric acid	Dual oxidase 2	h001	validated
MOL000305	lauric acid	T-lymphocyte activation antigen CD80	944	validated
MOL000305	lauric acid	T-lymphocyte activation antigen CD86	942	validated
MOL000305	lauric acid	Tumor necrosis factor receptor superfamily member 5	h001	validated
MOL000358	beta-sitosterol	Progesterone receptor	614	
MOL000358	beta-sitosterol	Nuclear receptor coactivator 2	6241	

MOL000358	beta-sitosterol	Prostaglandin G/H synthase 1	20	
MOL000358	beta-sitosterol	Prostaglandin G/H synthase 2	290	
MOL000358	beta-sitosterol	Heat shock protein HSP 90-alpha	1939	
MOL000358	beta-sitosterol	Phosphatidylinositol-4,5-bisphosphate 3-kinase catalytic subunit gamma isoform	2404	
MOL000358	beta-sitosterol	Potassium voltage-gated channel subfamily H member 2	101	
MOL000358	beta-sitosterol	cAMP-dependent protein kinase catalytic subunit alpha	6263	
MOL000358	beta-sitosterol	D(1A) dopamine receptor	23	
MOL000358	beta-sitosterol	Muscarinic acetylcholine receptor M3	51	
MOL000358	beta-sitosterol	Muscarinic acetylcholine receptor M1	103	
MOL000358	beta-sitosterol	Sodium channel protein type 5 subunit alpha	220	
MOL000358	beta-sitosterol	Gamma-aminobutyric-acid receptor subunit alpha-2	423	
MOL000358	beta-sitosterol	Muscarinic acetylcholine receptor M4	450	
MOL000358	beta-sitosterol	cGMP-inhibited 3',5'-cyclic phosphodiesterase A	485	
MOL000358	beta-sitosterol	5-hydroxytryptamine 2A receptor	502	
MOL000358	beta-sitosterol	Gamma-aminobutyric-acid receptor subunit alpha-5	523	
MOL000358	beta-sitosterol	Alpha-1A adrenergic receptor	556	
MOL000358	beta-sitosterol	Gamma-aminobutyric-acid receptor subunit alpha-3	580	
MOL000358	beta-sitosterol	Muscarinic acetylcholine receptor M2	617	
MOL000358	beta-sitosterol	Alpha-1B adrenergic receptor	632	
MOL000358	beta-sitosterol	Beta-2 adrenergic receptor	766	
MOL000358	beta-sitosterol	Neuronal acetylcholine receptor subunit alpha-2	813	
MOL000358	beta-sitosterol	Sodium-dependent serotonin transporter	824	
MOL000358	beta-sitosterol	Mu-type opioid receptor	847	
MOL000358	beta-sitosterol	Gamma-aminobutyric-acid receptor subunit alpha-1	872	
MOL000358	beta-sitosterol	Neuronal acetylcholine receptor subunit alpha-7	4095	
MOL000358	beta-sitosterol	Cytochrome P450-cam	2298	
MOL000358	beta-sitosterol	Apoptosis regulator Bcl-2	273	validated

MOL000358	beta-sitosterol	Apoptosis regulator BAX	h001	validated
MOL000358	beta-sitosterol	Caspase-9	h001	validated
MOL000358	beta-sitosterol	Transcription factor AP-1	1629	validated
MOL000358	beta-sitosterol	Caspase-3	h001	validated
MOL000358	beta-sitosterol	Caspase-8	h001	validated
MOL000358	beta-sitosterol	Protein kinase C alpha type	h001	validated
MOL000358	beta-sitosterol	Transforming growth factor beta-1	h001	validated
MOL000358	beta-sitosterol	Serum paraoxonase/arylesterase 1	1198	validated
MOL000358	beta-sitosterol	Microtubule-associated protein 2	1852	validated
MOL000365	syringaresinol	Coagulation factor X	239	
MOL000365	syringaresinol	Prostaglandin G/H synthase 2	290	
MOL000365	syringaresinol	DNA topoisomerase 2-alpha	817	
MOL000365	syringaresinol	Nuclear receptor coactivator 2	6241	
MOL000365	syringaresinol	Calmodulin	465	
MOL000365	syringaresinol	Potassium voltage-gated channel subfamily H member 2	101	
MOL000365	syringaresinol	Coagulation factor VII	369	
MOL000365	syringaresinol	Calcium-activated potassium channel subunit alpha 1	610	
MOL000365	syringaresinol	Heat shock protein HSP 90-alpha	1939	
MOL000422	kaempferol	Nitric oxide synthase, inducible	7	validated
MOL000422	kaempferol	Prostaglandin G/H synthase 1	20	
MOL000422	kaempferol	Androgen receptor	146	
MOL000422	kaempferol	Peroxisome proliferator-activated receptor gamma	238	
MOL000422	kaempferol	Prostaglandin G/H synthase 2	290	validated
MOL000422	kaempferol	Heat shock protein HSP 90-alpha	1939	
MOL000422	kaempferol	Phosphatidylinositol-4,5-bisphosphate 3-kinase catalytic subunit gamma isoform	2404	
MOL000422	kaempferol	cAMP-dependent protein kinase catalytic subunit alpha	6263	
MOL000422	kaempferol	Nuclear receptor coactivator 2	6241	
MOL000422	kaempferol	Dipeptidyl peptidase 4	952	
MOL000422	kaempferol	Trypsin-1	3176	
MOL000422	kaempferol	Progesterone receptor	614	
MOL000422	kaempferol	Prothrombin	54	
MOL000422	kaempferol	Muscarinic acetylcholine receptor M1	103	

MOL000422	kaempferol	Nitric-oxide synthase, endothelial	291	
MOL000422	kaempferol	Gamma-aminobutyric-acid receptor subunit alpha-2	423	
MOL000422	kaempferol	Acetylcholinesterase	474	
MOL000422	kaempferol	Sodium-dependent noradrenaline transporter	540	
MOL000422	kaempferol	Muscarinic acetylcholine receptor M2	617	
MOL000422	kaempferol	Alpha-1B adrenergic receptor	632	
MOL000422	kaempferol	Gamma-aminobutyric-acid receptor subunit alpha-1	872	
MOL000422	kaempferol	DNA topoisomerase 2-alpha	817	
MOL000422	kaempferol	Coagulation factor VII	369	
MOL000422	kaempferol	Calmodulin	465	
MOL000422	kaempferol	Transcription factor p65	h001	validated
MOL000422	kaempferol	Inhibitor of nuclear factor kappa-B kinase subunit beta	h001	validated
MOL000422	kaempferol	RAC-alpha serine/threonine-protein kinase	h001	validated
MOL000422	kaempferol	Apoptosis regulator Bcl-2	273	validated
MOL000422	kaempferol	Apoptosis regulator BAX	h001	validated
MOL000422	kaempferol	Tumor necrosis factor	777	validated
MOL000422	kaempferol	Transcription factor AP-1	1629	validated
MOL000422	kaempferol	Activator of 90 kDa heat shock protein ATPase homolog 1	h001	validated
MOL000422	kaempferol	Caspase-3	h001	validated
MOL000422	kaempferol	Mitogen-activated protein kinase 8	6292	validated
MOL000422	kaempferol	Xanthine dehydrogenase/oxidase	3947	validated
MOL000422	kaempferol	Interstitial collagenase	1167	validated
MOL000422	kaempferol	Signal transducer and activator of transcription 1-alpha/beta	h001	validated
MOL000422	kaempferol	Cell division control protein 2 homolog	1771	validated
MOL000422	kaempferol	Peroxisome proliferator-activated receptor gamma	h001	validated
MOL000422	kaempferol	Heme oxygenase 1	3391	validated
MOL000422	kaempferol	Cytochrome P450 3A4	4512	validated
MOL000422	kaempferol			validated
MOL000422	kaempferol	Cytochrome P450 1A1	h001	validated
MOL000422	kaempferol	Intercellular adhesion molecule 1	h001	validated
MOL000422	kaempferol	E-selectin	1756	validated
MOL000422	kaempferol	Vascular cell adhesion protein 1	1908	validated
MOL000422	kaempferol	Nuclear receptor subfamily 1 group I member 2	h001	validated
MOL000422	kaempferol	Cytochrome P450 1B1	h001	validated

MOL000422	kaempferol	Arachidonate 5-lipoxygenase	275	validated
MOL000422	kaempferol	Hyaluronan synthase 2	h001	validated
MOL000422	kaempferol	Aryl hydrocarbon receptor	6220	validated
MOL000422	kaempferol	26S proteasome non-ATPase regulatory subunit 3	h001	validated
MOL000422	kaempferol	Solute carrier family 2, facilitated glucose transporter member 4	h001	validated
MOL000422	kaempferol	Nuclear receptor subfamily 1 group I member 3	h001	validated
MOL000422	kaempferol	Insulin receptor	36	validated
MOL000422	kaempferol	Type I iodothyronine deiodinase	h001	validated
MOL000422	kaempferol	Serine/threonine-protein phosphatase 2B catalytic subunit alpha isoform	6746	validated
MOL000422	kaempferol	Peroxidase C1A	h001	validated
MOL000422	kaempferol	Glutathione S-transferase Mu 1	896	validated
MOL000422	kaempferol	Glutathione S-transferase Mu 2	2165	validated
MOL000422	kaempferol	Aldo-keto reductase family 1 member C3	650	validated
MOL000422	kaempferol	Antileukoproteinase	h001	validated
MOL000449	Stigmasterol	Progesterone receptor	614	
MOL000449	Stigmasterol	Mineralocorticoid receptor	737	
MOL000449	Stigmasterol	Nuclear receptor coactivator 2	6241	
MOL000449	Stigmasterol			
MOL000449	Stigmasterol	Ig gamma-1 chain C region	4785	
MOL000449	Stigmasterol	Retinoic acid receptor RXR-alpha	459	
MOL000449	Stigmasterol	Nuclear receptor coactivator 1	6228	
MOL000449	Stigmasterol	Prostaglandin G/H synthase 1	20	
MOL000449	Stigmasterol	Prostaglandin G/H synthase 2	290	
MOL000449	Stigmasterol	Alpha-2A adrenergic receptor	318	
MOL000449	Stigmasterol	Sodium-dependent noradrenaline transporter	540	
MOL000449	Stigmasterol	Sodium-dependent dopamine transporter	713	
MOL000449	Stigmasterol	Beta-2 adrenergic receptor	766	
MOL000449	Stigmasterol	Aldose reductase	822	
MOL000449	Stigmasterol	Urokinase-type plasminogen activator	1074	
MOL000449	Stigmasterol	Leukotriene A-4 hydrolase	3060	
MOL000449	Stigmasterol	Amine oxidase [flavin-containing] B	3939	
MOL000449	Stigmasterol	Amine oxidase [flavin-containing] A	3941	

MOL000449	Stigmasterol	cAMP-dependent protein kinase catalytic subunit alpha	6263	
MOL000449	Stigmasterol	Chymotrypsinogen B	6011	
MOL000449	Stigmasterol	Muscarinic acetylcholine receptor M3	51	
MOL000449	Stigmasterol	Muscarinic acetylcholine receptor M1	103	
MOL000449	Stigmasterol	Beta-1 adrenergic receptor	193	
MOL000449	Stigmasterol	Sodium channel protein type 5 subunit alpha	220	
MOL000449	Stigmasterol	5-hydroxytryptamine 2A receptor	502	
MOL000449	Stigmasterol	Alpha-1A adrenergic receptor	556	
MOL000449	Stigmasterol	Gamma-aminobutyric-acid receptor subunit alpha-3	580	
MOL000449	Stigmasterol	Muscarinic acetylcholine receptor M2	617	
MOL000449	Stigmasterol	Alpha-1B adrenergic receptor	632	
MOL000449	Stigmasterol	Gamma-aminobutyric-acid receptor subunit alpha-1	872	
MOL000449	Stigmasterol	Neuronal acetylcholine receptor subunit alpha-7	4095	
MOL000478	Eucarvone	Gamma-aminobutyric-acid receptor subunit alpha-2	423	
MOL000478	Eucarvone	Gamma-aminobutyric-acid receptor subunit alpha-5	523	
MOL000478	Eucarvone	Muscarinic acetylcholine receptor M2	617	
MOL000478	Eucarvone	Gamma-aminobutyric-acid receptor subunit alpha-1	872	
MOL000478	Eucarvone	Cytochrome P450-cam	2298	
MOL000478	Eucarvone	Glucocorticoid receptor	871	
MOL000666	hexanal	Tumor necrosis factor	777	validated
MOL000666	hexanal	Transcription factor AP-1	1629	validated
MOL000666	hexanal	Tissue factor	2139	validated
MOL000666	hexanal	Mitogen-activated protein kinase 10	1836	validated
MOL000666	hexanal	Abl interactor 1	h001	validated
MOL000666	hexanal	Platelet glycoprotein 4	h001	validated
MOL000668	PENTYLFURAN	Sodium-dependent noradrenaline transporter	540	
MOL000668	PENTYLFURAN	Alpha-1A adrenergic receptor	556	
MOL000675	oleic acid	Prostaglandin G/H synthase 1	20	
MOL000675	oleic acid	Nuclear receptor coactivator 2	6241	
MOL000675	oleic acid	Prostaglandin G/H synthase 2	290	
MOL000675	oleic acid			

MOL000675	oleic acid			
MOL000675	oleic acid			
MOL000675	oleic acid	Lysozyme	2300	
MOL000675	oleic acid	Nicotinate-nucleotide--dimethylbenzimidazole phosphoribosyltransferase	2264	
MOL000675	oleic acid	Trypsin-3	2886	
MOL000675	oleic acid	Retinoic acid receptor RXR-alpha	459	
MOL000675	oleic acid	Cytochrome P450-cam	2298	
MOL000675	oleic acid	Urokinase-type plasminogen activator	1074	validated
MOL000675	oleic acid	Superoxide dismutase [Cu-Zn]	4152	validated
MOL000675	oleic acid			validated
MOL000675	oleic acid	Telomerase protein component 1	h001	validated
MOL000675	oleic acid	Endothelin-1	h001	validated
MOL000675	oleic acid	Receptor tyrosine-protein kinase erbB-2	h001	validated
MOL000675	oleic acid	Peroxisome proliferator-activated receptor gamma	h001	validated
MOL000675	oleic acid	Lipoprotein lipase	266	validated
MOL000675	oleic acid	Neuromodulin	h001	validated
MOL000675	oleic acid	Plasminogen activator inhibitor 1	h001	validated
MOL000675	oleic acid	Brain-derived neurotrophic factor	h001	validated
MOL000675	oleic acid	3-hydroxy-3-methylglutaryl-coenzyme A reductase	3387	validated
MOL000675	oleic acid	Myeloperoxidase	1757	validated
MOL000675	oleic acid	Peroxisome proliferator-activated receptor alpha	h001	validated
MOL000675	oleic acid	Peroxisome proliferator-activated receptor delta	h001	validated
MOL000675	oleic acid	C-reactive protein	h001	validated
MOL000675	oleic acid	Serum paraoxonase/arylesterase 1	1198	validated
MOL000675	oleic acid	Insulin	5961	validated
MOL000675	oleic acid	Plasminogen	234	validated
MOL000675	oleic acid	Fatty acid-binding protein, liver	h001	validated
MOL000675	oleic acid	Retinol-binding protein 2	h001	validated
MOL000675	oleic acid	Glucagon	h001	validated
MOL000675	oleic acid	Glutamyl aminopeptidase	4020	validated
MOL000675	oleic acid	Mitochondrial uncoupling protein 2	h001	validated
MOL000675	oleic acid	Sterol O-acyltransferase 1	117	validated
MOL000675	oleic acid	Cholecystokinin	h001	validated
MOL000675	oleic acid	Cbp/p300-interacting transactivator 1	h001	validated
MOL000675	oleic acid	Pancreas/duodenum homeobox protein 1	h001	validated

MOL000675	oleic acid	Solute carrier family 2, facilitated glucose transporter member 2	h001	validated
MOL000675	oleic acid	Peptidyl-glycine alpha-amidating monooxygenase	3943	validated
MOL000675	oleic acid	Acyl-CoA desaturase	h001	validated
MOL000675	oleic acid	Mitochondrial uncoupling protein 3	h001	validated
MOL000675	oleic acid	Cholesteryl ester transfer protein	h001	validated
MOL000675	oleic acid	Peptide YY	h001	validated
MOL000675	oleic acid	Aspartyl aminopeptidase	4026	validated
MOL000675	oleic acid	Cell-death-related nuclease 7	h001	validated
MOL000723	trans-2,4-decadienal	Gamma-aminobutyric-acid receptor subunit alpha-2	423	
MOL000723	trans-2,4-decadienal	Gamma-aminobutyric-acid receptor subunit alpha-1	872	
MOL000723	trans-2,4-decadienal	Cytochrome P450-cam	2298	
MOL000723	trans-2,4-decadienal			
MOL000723	trans-2,4-decadienal	G1/S-specific cyclin-D1	h001	validated
MOL000723	trans-2,4-decadienal	Eukaryotic translation initiation factor 6	h001	validated
MOL000723	trans-2,4-decadienal	Retinoblastoma-associated protein	3846	validated
MOL000723	trans-2,4-decadienal	Cell division protein kinase 4	3959	validated
MOL000860	stearic acid	Prostaglandin G/H synthase 1	20	
MOL000860	stearic acid	Prostaglandin G/H synthase 2	290	
MOL000860	stearic acid	Retinoic acid receptor RXR-alpha	459	
MOL000860	stearic acid	Nuclear receptor coactivator 2	6241	
MOL000860	stearic acid	Ig gamma-1 chain C region	4785	
MOL000860	stearic acid	Transcription factor Sp1	h001	validated
MOL000860	stearic acid	Ectonucleotide pyrophosphatase/phosphodiesterase family member 7	h001	validated
MOL000874	paeonol	Prostaglandin G/H synthase 1	20	
MOL000874	paeonol	Beta-1 adrenergic receptor	193	
MOL000874	paeonol	Prostaglandin G/H synthase 2	290	
MOL000874	paeonol	Alpha-2A adrenergic receptor	318	
MOL000874	paeonol	Alpha-2C adrenergic receptor	378	
MOL000874	paeonol	Sodium-dependent noradrenaline transporter	540	
MOL000874	paeonol	Alpha-2B adrenergic receptor	629	

MOL000874	paeonol	Alpha-1B adrenergic receptor	632	
MOL000874	paeonol	Sodium-dependent dopamine transporter	713	
MOL000874	paeonol	Beta-2 adrenergic receptor	766	
MOL000874	paeonol	Alpha-1D adrenergic receptor	789	
MOL000874	paeonol	Beta-lactamase	2478	
MOL000874	paeonol	Amine oxidase [flavin-containing] B	3939	validated
MOL000874	paeonol	Amine oxidase [flavin-containing] A	3941	validated
MOL000874	paeonol	Chymotrypsinogen B	6011	
MOL000874	paeonol	Alpha-1A adrenergic receptor	556	
MOL000874	paeonol	Muscarinic acetylcholine receptor M1	103	
MOL000874	paeonol	Muscarinic acetylcholine receptor M2	617	
MOL000874	paeonol	Transcription factor p65	h001	validated
MOL000874	paeonol	RAC-alpha serine/threonine-protein kinase	h001	validated
MOL000874	paeonol	Apoptosis regulator Bcl-2	273	validated
MOL000874	paeonol	Apoptosis regulator BAX	h001	validated
MOL000874	paeonol	Mitogen-activated protein kinase 1	1176	validated
MOL000874	paeonol	Tumor necrosis factor	777	validated
MOL000874	paeonol	Activator of 90 kDa heat shock protein ATPase homolog 1	h001	validated
MOL000874	paeonol	NF-kappa-B inhibitor alpha	h001	validated
MOL000874	paeonol	Intercellular adhesion molecule 1	h001	validated
MOL000874	paeonol	Interleukin-2	1575	validated
MOL000874	paeonol	Tyrosinase	h001	validated
MOL000874	paeonol	Phosphatidylinositol-3,4,5-trisphosphate 3-phosphatase and dual-specificity protein phosphatase PTEN	h001	validated
MOL000879	methyl palmitate	Prostaglandin G/H synthase 1	20	
MOL000879	methyl palmitate	Nuclear receptor coactivator 2	6241	
MOL000879	methyl palmitate	Ig gamma-1 chain C region	4785	
MOL000879	methyl palmitate	Transcription factor p65	h001	validated
MOL000879	methyl palmitate	Interleukin-10	h001	validated
MOL000879	methyl palmitate	Tumor necrosis factor	777	validated
MOL000879	methyl palmitate	Interleukin-6	1159	validated
MOL000879	methyl palmitate	Prostaglandin G/H synthase 2	290	validated
MOL000879	methyl palmitate	Prostaglandin E2 receptor, EP3 subtype	4131	validated
MOL000896	13657-68-6	Mineralocorticoid receptor	737	

MOL000896	13657-68-6	Muscarinic acetylcholine receptor M1	103
MOL000896	13657-68-6	Gamma-aminobutyric-acid receptor subunit alpha-2	423
MOL000896	13657-68-6	Gamma-aminobutyric-acid receptor subunit alpha-5	523
MOL000896	13657-68-6	Gamma-aminobutyric-acid receptor subunit alpha-3	580
MOL000896	13657-68-6	Muscarinic acetylcholine receptor M2	617
MOL000896	13657-68-6	Gamma-aminobutyric-acid receptor subunit alpha-1	872
MOL000896	13657-68-6	Ig gamma-1 chain C region	4785
MOL000896	13657-68-6	Gamma-aminobutyric-acid receptor subunit alpha-6	841
MOL000910	Germacron	Gamma-aminobutyric-acid receptor subunit alpha-2	423
MOL000910	Germacron	Gamma-aminobutyric-acid receptor subunit alpha-1	872
MOL000910	Germacron	Cytochrome P450-cam	2298
MOL000910	Germacron	Prostaglandin G/H synthase 1	20
MOL000910	Germacron	Muscarinic acetylcholine receptor M1	103
MOL000910	Germacron	Prostaglandin G/H synthase 2	290
MOL000910	Germacron	Sodium-dependent noradrenaline transporter	540
MOL000910	Germacron	Glutamate receptor 2	921
MOL000920	LINALOOL (D)	Muscarinic acetylcholine receptor M1	103
MOL000920	LINALOOL (D)	Gamma-aminobutyric-acid receptor subunit alpha-2	423
MOL000920	LINALOOL (D)	Muscarinic acetylcholine receptor M2	617
MOL000920	LINALOOL (D)	Sodium-dependent noradrenaline transporter	540
MOL000920	LINALOOL (D)	Gamma-aminobutyric-acid receptor subunit alpha-5	523
MOL000920	LINALOOL (D)	Gamma-aminobutyric-acid receptor subunit alpha-3	580
MOL000920	LINALOOL (D)	Gamma-aminobutyric-acid receptor subunit alpha-6	841
MOL000920	LINALOOL (D)	Prostaglandin G/H synthase 2	290
MOL000920	LINALOOL (D)		
MOL000920	LINALOOL (D)	Gamma-aminobutyric-acid receptor subunit alpha-1	872
MOL000920	LINALOOL (D)	Lysozyme	2300
MOL000920	LINALOOL (D)	Nicotinate-nucleotide--dimethylbenzimidazole phosphoribosyltransferase	2264
MOL000920	LINALOOL (D)	Cytochrome P450-cam	2298

MOL000920	LINALOOL (D)	Prostaglandin G/H synthase 1	20	
MOL000920	LINALOOL (D)	Muscarinic acetylcholine receptor M3	51	
MOL000920	LINALOOL (D)	Beta-1 adrenergic receptor	193	
MOL000920	LINALOOL (D)	Alpha-2A adrenergic receptor	318	
MOL000920	LINALOOL (D)	Retinoic acid receptor RXR-alpha	459	
MOL000920	LINALOOL (D)	Alpha-1A adrenergic receptor	556	
MOL000920	LINALOOL (D)	Alpha-1B adrenergic receptor	632	
MOL000920	LINALOOL (D)	Sodium-dependent dopamine transporter	713	
MOL000920	LINALOOL (D)	Beta-2 adrenergic receptor	766	
MOL000920	LINALOOL (D)	Sodium-dependent serotonin transporter	824	
MOL000920	LINALOOL (D)	Leukotriene A-4 hydrolase	3060	
MOL000920	LINALOOL (D)	Amine oxidase [flavin-containing] B	3939	
MOL000920	LINALOOL (D)	cAMP-dependent protein kinase inhibitor alpha	6264	
MOL000971	Ethylpalmitate	Nuclear receptor coactivator 2	6241	
MOL001283	C09704	Prostaglandin G/H synthase 2	290	
MOL001283	C09704	Nuclear receptor coactivator 2	6241	
MOL001393	myristic acid	Prostaglandin G/H synthase 1	20	
MOL001393	myristic acid	Prostaglandin G/H synthase 2	290	
MOL001393	myristic acid	Cholinesterase	3923	
MOL001393	myristic acid	Phospholipase A2	6500	
MOL001393	myristic acid	Genome polyprotein	6521	
MOL001393	myristic acid	Ig gamma-1 chain C region	4785	
MOL001393	myristic acid	Ferrichrome-iron receptor	2427	
MOL001393	myristic acid	3-oxoacyl-[acyl-carrier-protein] synthase 1	889	
MOL001393	myristic acid	Nuclear receptor coactivator 2	6241	
MOL001393	myristic acid	Nuclear receptor coactivator 1	6228	
MOL001393	myristic acid	Phosphatidylcholine-sterol acyltransferase	h001	validated
MOL001417	OCTENAL			
MOL001417	OCTENAL			
MOL001417	OCTENAL	Trypsin-3	2886	
MOL001494	Mandenol	Prostaglandin G/H synthase 1	20	
MOL001494	Mandenol	Prostaglandin G/H synthase 2	290	

MOL001494	Mandenol	Nuclear receptor coactivator 2	6241
MOL001501	Daturic acid	Prostaglandin G/H synthase 1	20
MOL001501	Daturic acid	Ig gamma-1 chain C region	4785
MOL001501	Daturic acid	Nuclear receptor coactivator 2	6241
MOL001641	METHYL LINOLEATE	Prostaglandin G/H synthase 1	20
MOL001641	METHYL LINOLEATE	Prostaglandin G/H synthase 2	290
MOL001641	METHYL LINOLEATE	Nuclear receptor coactivator 2	6241
MOL001641	METHYL LINOLEATE	Retinoic acid receptor RXR-alpha	459
MOL001661	Scandoside methyl ester	Carbonic anhydrase 2	357
MOL001662	scandoside_qt	Prostaglandin G/H synthase 1	20
MOL001662	scandoside_qt	Prostaglandin G/H synthase 2	290
MOL001662	scandoside_qt	Carbonic anhydrase 2	357
MOL001662	scandoside_qt	Trypsin-1	3176
MOL001662	scandoside_qt	Glutamate receptor 2	921
MOL001666	Deacetyl asperulosidic acid methyl ester	Carbonic anhydrase 2	357
MOL001666	Deacetyl asperulosidic acid methyl ester	Trypsin-1	3176
MOL001667	deacetyl asperuloside acid_qt	Prostaglandin G/H synthase 1	20
MOL001667	deacetyl asperuloside acid_qt	Prostaglandin G/H synthase 2	290
MOL001667	deacetyl asperuloside acid_qt	Carbonic anhydrase 2	357
MOL001667	deacetyl asperuloside acid_qt	Trypsin-1	3176
MOL001667	deacetyl asperuloside acid_qt	Glutamate receptor 2	921
MOL001668	Geniposidic acid	Carbonic anhydrase 2	357
MOL001668	Geniposidic acid	Prothrombin	54
MOL001669	geniposidie acid_qt	Prostaglandin G/H synthase 2	290
MOL001669	geniposidie acid_qt	Carbonic anhydrase 2	357
MOL001669	geniposidie acid_qt	Gamma-aminobutyric-acid receptor subunit alpha-1	872
MOL001669	geniposidie acid_qt	Trypsin-1	3176
MOL001669	geniposidie acid_qt	Glutamate receptor 2	921
MOL001669	geniposidie acid_qt	Ig gamma-1 chain C region	4785
MOL001739	zoomaric acid	Prostaglandin G/H synthase 1	20
MOL001739	zoomaric acid	Prostaglandin G/H synthase 2	290
MOL001739	zoomaric acid	Nuclear receptor coactivator 2	6241

MOL001745	Methyl vaccenate	Nuclear receptor coactivator 2	6241	
MOL001850	Izoforon	Gamma-aminobutyric-acid receptor subunit alpha-2	423	
MOL001850	Izoforon	Gamma-aminobutyric-acid receptor subunit alpha-3	580	
MOL001850	Izoforon	Gamma-aminobutyric-acid receptor subunit alpha-1	872	
MOL001850	Izoforon	Cytochrome P450-cam	2298	
MOL001850	Izoforon	Gamma-aminobutyric-acid receptor subunit alpha-6	841	
MOL001942	isoimperatorin	Prostaglandin G/H synthase 2	290	validated
MOL002046	hexanoic acid			
MOL002046	hexanoic acid	Trypsin-3	2886	
MOL002046	hexanoic acid	Bacillolysin	2457	
MOL002046	hexanoic acid	Inositol-3-phosphate synthase 1	h001	validated
MOL002203	Excepar1 M-OL	Prostaglandin G/H synthase 1	20	
MOL002203	Excepar1 M-OL	Prostaglandin G/H synthase 2	290	
MOL002203	Excepar1 M-OL	Retinoic acid receptor RXR-alpha	459	
MOL002203	Excepar1 M-OL	Nuclear receptor coactivator 2	6241	
MOL002560	chrysin	Prostaglandin G/H synthase 1	20	
MOL002560	chrysin	Androgen receptor	146	
MOL002560	chrysin	Prostaglandin G/H synthase 2	290	validated
MOL002560	chrysin	cGMP-inhibited 3',5'-cyclic phosphodiesterase A	485	
MOL002560	chrysin	Sodium-dependent serotonin transporter	824	
MOL002560	chrysin	Gamma-aminobutyric-acid receptor subunit alpha-1	872	
MOL002560	chrysin	Dipeptidyl peptidase 4	952	
MOL002560	chrysin	Heat shock protein HSP 90-alpha	1939	
MOL002560	chrysin	Phosphatidylinositol-4,5-bisphosphate 3-kinase catalytic subunit gamma isoform	2404	
MOL002560	chrysin	Amine oxidase [flavin-containing] B	3939	
MOL002560	chrysin	cAMP-dependent protein kinase catalytic subunit alpha	6263	
MOL002560	chrysin	cAMP-dependent protein kinase inhibitor alpha	6264	
MOL002560	chrysin	Prothrombin	54	

MOL002560	chrysin	Cyclin-dependent kinase inhibitor 1	h001	validated
MOL002560	chrysin	Transforming growth factor beta-1	h001	validated
MOL002560	chrysin	Interleukin-4	h001	validated
MOL002560	chrysin	Cytochrome P450 19A1	h001	validated
MOL002560	chrysin	Interleukin-13	h001	validated
MOL002560	chrysin			validated
MOL002883	Ethyl oleate (NF)	Nuclear receptor coactivator 2	6241	
MOL003095	5-hydroxy-7-methoxy-2-(3,4,5-trimethoxyphenyl)chromone	Nitric oxide synthase, inducible	7	
MOL003095	5-hydroxy-7-methoxy-2-(3,4,5-trimethoxyphenyl)chromone	Prostaglandin G/H synthase 1	20	
MOL003095	5-hydroxy-7-methoxy-2-(3,4,5-trimethoxyphenyl)chromone	Prothrombin	54	
MOL003095	5-hydroxy-7-methoxy-2-(3,4,5-trimethoxyphenyl)chromone	Potassium voltage-gated channel subfamily H member 2	101	
MOL003095	5-hydroxy-7-methoxy-2-(3,4,5-trimethoxyphenyl)chromone	Estrogen receptor	136	
MOL003095	5-hydroxy-7-methoxy-2-(3,4,5-trimethoxyphenyl)chromone	Androgen receptor	146	
MOL003095	5-hydroxy-7-methoxy-2-(3,4,5-trimethoxyphenyl)chromone	Sodium channel protein type 5 subunit alpha	220	
MOL003095	5-hydroxy-7-methoxy-2-(3,4,5-trimethoxyphenyl)chromone	Peroxisome proliferator-activated receptor gamma	238	
MOL003095	5-hydroxy-7-methoxy-2-(3,4,5-trimethoxyphenyl)chromone	Coagulation factor X	239	
MOL003095	5-hydroxy-7-methoxy-2-(3,4,5-trimethoxyphenyl)chromone	Prostaglandin G/H synthase 2	290	
MOL003095	5-hydroxy-7-methoxy-2-(3,4,5-trimethoxyphenyl)chromone	Nitric-oxide synthase, endothelial	291	
MOL003095	5-hydroxy-7-methoxy-2-(3,4,5-trimethoxyphenyl)chromone	Voltage-dependent calcium channel subunit alpha-2/delta-1	762	
MOL003095	5-hydroxy-7-methoxy-2-(3,4,5-trimethoxyphenyl)chromone	DNA topoisomerase 2-alpha	817	
MOL003095	5-hydroxy-7-methoxy-2-(3,4,5-trimethoxyphenyl)chromone	Estrogen receptor beta	869	

MOL003095	5-hydroxy-7-methoxy-2-(3,4,5-trimethoxyphenyl)chromone	Dipeptidyl peptidase 4	952
MOL003095	5-hydroxy-7-methoxy-2-(3,4,5-trimethoxyphenyl)chromone	Mitogen-activated protein kinase 14	1540
MOL003095	5-hydroxy-7-methoxy-2-(3,4,5-trimethoxyphenyl)chromone	Glycogen synthase kinase-3 beta	1721
MOL003095	5-hydroxy-7-methoxy-2-(3,4,5-trimethoxyphenyl)chromone	Heat shock protein HSP 90-alpha	1939
MOL003095	5-hydroxy-7-methoxy-2-(3,4,5-trimethoxyphenyl)chromone	Serine/threonine-protein kinase Chk1	5790
MOL003095	5-hydroxy-7-methoxy-2-(3,4,5-trimethoxyphenyl)chromone	Trypsin-1	3176
MOL003095	5-hydroxy-7-methoxy-2-(3,4,5-trimethoxyphenyl)chromone	Nuclear receptor coactivator 2	6241
MOL003095	5-hydroxy-7-methoxy-2-(3,4,5-trimethoxyphenyl)chromone	Nuclear receptor coactivator 1	6228
MOL003095	5-hydroxy-7-methoxy-2-(3,4,5-trimethoxyphenyl)chromone	Calcium-activated potassium channel subunit alpha 1	610
MOL003095	5-hydroxy-7-methoxy-2-(3,4,5-trimethoxyphenyl)chromone	Calmodulin	465
MOL003095	5-hydroxy-7-methoxy-2-(3,4,5-trimethoxyphenyl)chromone	Beta-2 adrenergic receptor	766
MOL003095	5-hydroxy-7-methoxy-2-(3,4,5-trimethoxyphenyl)chromone	Beta-secretase 1	1406
MOL005840	PANA	Prostaglandin G/H synthase 1	20
MOL005840	PANA	Prostaglandin G/H synthase 2	290
MOL006219	Clorius	Lysozyme	2300
MOL006219	Clorius	Nicotinate-nucleotide--dimethylbenzimidazole phosphoribosyltransferase	2264
MOL006219	Clorius		

MOL007147	methyl (1S,4aS,5R,7S,7aS)-5,7-dihydroxy-7-methyl-1-[(2S,3R,4S,5S,6R)-3,4,5-trihydroxy-6-(hydroxymethyl)oxan-2-yl]oxy-4a,5,6,7a-tetrahydro-1H-cyclopenta[d]pyran-4-carboxylate	Carbonic anhydrase 2	357
MOL007147	methyl (1S,4aS,5R,7S,7aS)-5,7-dihydroxy-7-methyl-1-[(2S,3R,4S,5S,6R)-3,4,5-trihydroxy-6-(hydroxymethyl)oxan-2-yl]oxy-4a,5,6,7a-tetrahydro-1H-cyclopenta[d]pyran-4-carboxylate	Dipeptidyl peptidase 4	952
MOL007148	shanzhiside methyl ester_qt	Prostaglandin G/H synthase 2	290
MOL007148	shanzhiside methyl ester_qt	Carbonic anhydrase 2	357
MOL007148	shanzhiside methyl ester_qt	Trypsin-1	3176
MOL007148	shanzhiside methyl ester_qt	Glutamate receptor 2	921
MOL007244	3,4-di-o-caffeoylquinic acid	Coagulation factor X	239
MOL007244	3,4-di-o-caffeoylquinic acid	Tyrosine-protein phosphatase non-receptor type 1	687
MOL007245	3-Methylkempferol	Nitric oxide synthase, inducible	7
MOL007245	3-Methylkempferol	Prostaglandin G/H synthase 1	20
MOL007245	3-Methylkempferol	Androgen receptor	146
MOL007245	3-Methylkempferol	Prostaglandin G/H synthase 2	290
MOL007245	3-Methylkempferol	Dipeptidyl peptidase 4	952
MOL007245	3-Methylkempferol	Mitogen-activated protein kinase 14	1540
MOL007245	3-Methylkempferol	Glycogen synthase kinase-3 beta	1721
MOL007245	3-Methylkempferol	Heat shock protein HSP 90-alpha	1939
MOL007245	3-Methylkempferol	Cell division protein kinase 2	2240
MOL007245	3-Methylkempferol	Phosphatidylinositol-4,5-bisphosphate 3-kinase catalytic subunit gamma isoform	2404
MOL007245	3-Methylkempferol	cAMP-dependent protein kinase catalytic subunit alpha	6263
MOL009547	desacetyl asperulosidic acid	Carbonic anhydrase 2	357

MOL009548	desacetyl asperulosidic acid_qt	Prostaglandin G/H synthase 2	290
MOL009548	desacetyl asperulosidic acid_qt	Carbonic anhydrase 2	357
MOL009548	desacetyl asperulosidic acid_qt	Trypsin-1	3176
MOL009548	desacetyl asperulosidic acid_qt	Glutamate receptor 2	921
