# Supplementary material for: The Study of Yin-Chen-Hao-Tang Preventing and Treating Alcoholic Fatty Liver Disease through PPAR Signaling Pathway Based on Network Pharmacology and RNA-Seq Transcriptomics
Source: Evid Based Complement Alternat Med. 2021 Dec 31;2021:8917993. doi: 10.1155/2021/8917993 (PMC8741355; doi:10.1155/2021/8917993)
Supplement: Supplementary Materials — Supplementary Material 1-1: Herb Target-Artemisiae Scopariae Herba. Supplementary Material 1-2: Herb Target-Gardeniae Fructus. Supplementary Material 1-3: Herb Target-Radix Rhei Et Rhizome. Supplementary Material 2: AFLD-GeneCards-SearchResults. Supplementary Material 3: string_interactions.tsv default node. [file 8917993.f1.zip › 8917993.f1/Supplementary material 1-3 Herb Target-Radix Rhei Et Rhizome.pdf]

Mol ID	Molecule Name	Target Name	drugbank	Status
MOL001237	o-Acetyltoluene			
MOL001237	o-Acetyltoluene	Lysozyme	2300	
MOL001237	o-Acetyltoluene	Nicotinate-nucleotide--dimethylbenzimidazole phosphoribosyltransferase	2264	
MOL001237	o-Acetyltoluene	Glucocorticoid receptor	871	
MOL001301	cis-Zimtsaeure	Amine oxidase [flavin-containing] B	3939	
MOL001301	cis-Zimtsaeure	Amine oxidase [flavin-containing] A	3941	
MOL001301	cis-Zimtsaeure	Lysozyme	2300	
MOL001301	cis-Zimtsaeure	Chymotrypsinogen B	6011	
MOL001456	citric acid	Coagulation factor X	239	
MOL001456	citric acid	Prostaglandin G/H synthase 2	290	
MOL001456	citric acid	Aldose reductase	822	
MOL001456	citric acid	Proto-oncogene tyrosine-protein kinase Src	933	
MOL001456	citric acid	Cathepsin D	1243	
MOL001456	citric acid	Ig gamma-1 chain C region	4785	
MOL001456	citric acid	Nitric-oxide synthase, brain	76	
MOL001456	citric acid	Glutamate [NMDA] receptor subunit epsilon-1	837	
MOL001456	citric acid	Gamma-aminobutyric-acid receptor subunit alpha-1	872	
MOL001456	citric acid	Ferrichrome-iron receptor	2427	
MOL001456	citric acid	Glutamate receptor 2	921	
MOL001456	citric acid	Tyrosine-protein phosphatase non-receptor type 1	687	
MOL001456	citric acid	Bacillolysin	2457	
MOL001456	citric acid	Carboxypeptidase B	5579	
MOL001456	citric acid	Triosephosphate isomerase	6346	
MOL001456	citric acid	Glutamate receptor, ionotropic kainate 2	918	
MOL001456	citric acid	Histidine decarboxylase	3	validated
MOL001729	Crysophanol	Prostaglandin G/H synthase 1	20	
MOL001729	Crysophanol	Prostaglandin G/H synthase 2	290	
MOL001729	Crysophanol	Heat shock protein HSP 90-alpha	1939	
MOL001729	Crysophanol	Nuclear receptor coactivator 2	6241	
MOL001729	Crysophanol	Calmodulin	465	
MOL001729	Crysophanol	Sodium channel protein type 5 subunit alpha	220	
MOL001729	Crysophanol	cGMP-inhibited 3',5'-cyclic phosphodiesterase A	485	
MOL001729	Crysophanol	Gamma-aminobutyric-acid receptor subunit alpha-1	872	

MOL001729	Crysophanol	Phosphatidylinositol-4,5-bisphosphate 3-kinase catalytic subunit gamma isoform	2404
MOL001729	Crysophanol	cAMP-dependent protein kinase catalytic subunit alpha	6263
MOL001729	Crysophanol	Ig gamma-1 chain C region	4785
MOL001729	Crysophanol	cAMP-dependent protein kinase inhibitor alpha	6264
MOL001794	MAE	4-aminobutyrate aminotransferase, mitochondrial	280
MOL001794	MAE	Aspartate aminotransferase, cytoplasmic	586
MOL001794	MAE	Proto-oncogene tyrosine-protein kinase Src	933
MOL001794	MAE		
MOL001794	MAE		
MOL001794	MAE	Aspartate aminotransferase	2249
MOL001794	MAE	Ferrichrome-iron receptor	2427
MOL001794	MAE	Aromatic-amino-acid aminotransferase	2537
MOL001794	MAE	1-aminocyclopropane-1-carboxylate deaminase	2518
MOL001794	MAE	Growth-inhibiting protein 18	3877
MOL001794	MAE	Aspartate aminotransferase, mitochondrial	426
MOL001794	MAE	Triosephosphate isomerase	6346
MOL001794	MAE	Cholinesterase	3923
MOL001794	MAE	Cytochrome P450-cam	2298
MOL001794	MAE	Bacillolysin	2457
MOL001794	MAE	Nicotinate-nucleotide--dimethylbenzimidazole phosphoribosyltransferase	2264
MOL001794	MAE	NAD-dependent malic enzyme, mitochondrial	166
MOL001880	OXL	Prostaglandin G/H synthase 1	20
MOL001880	OXL	Nitric-oxide synthase, brain	76
MOL001880	OXL	Procollagen-lysine,2-oxoglutarate 5-dioxygenase 1	97
MOL001880	OXL	Aldehyde dehydrogenase, mitochondrial	147
MOL001880	OXL	Succinate semialdehyde dehydrogenase, mitochondrial	170
MOL001880	OXL	4-aminobutyrate aminotransferase, mitochondrial	280

MOL001880	OXL	Prostaglandin G/H synthase 2	290
MOL001880	OXL	Serine hydroxymethyltransferase, mitochondrial	321
MOL001880	OXL	DNA polymerase	338
MOL001880	OXL	Glutamate [NMDA] receptor subunit zeta-1	401
MOL001880	OXL	Kynureninase	435
MOL001880	OXL	Ornithine aminotransferase, mitochondrial	472
MOL001880	OXL	Glycine receptor subunit alpha-1	482
MOL001880	OXL	Aspartate aminotransferase, cytoplasmic	586
MOL001880	OXL	Glutamate [NMDA] receptor subunit epsilon-1	837
MOL001880	OXL	Gamma-aminobutyric-acid receptor subunit alpha-1	872
MOL001880	OXL	Proto-oncogene tyrosine-protein kinase Src	933
MOL001880	OXL	Cathepsin D	1243
MOL001880	OXL	Lactotransferrin	1439
MOL001880	OXL	Macrophage metalloelastase	2203
MOL001880	OXL	Leukotriene A-4 hydrolase	3060
MOL001880	OXL	M-phase inducer phosphatase 2	3105
MOL001880	OXL	Thioredoxin reductase 1, cytoplasmic	3610
MOL001880	OXL	Cholinesterase	3923
MOL001880	OXL	Xanthine dehydrogenase/oxidase	3947
MOL001880	OXL		
MOL001880	OXL		
MOL001880	OXL		
MOL001880	OXL		
MOL001880	OXL		
MOL001880	OXL		
MOL001880	OXL		
MOL001880	OXL		
MOL001880	OXL	Monocarboxylate transporter 2	489
MOL001880	OXL	Cytochrome P450-cam	2298
MOL001880	OXL	Aspartate aminotransferase	2249
MOL001880	OXL	Beta-galactosidase	2592
MOL001880	OXL	Ferrichrome-iron receptor	2427
MOL001880	OXL	Histidinol dehydrogenase	3191
MOL001880	OXL	Pyruvate dehydrogenase [cytochrome]	845
MOL001880	OXL	Acetyl-CoA acetyltransferase	2596
MOL001880	OXL	Formate dehydrogenase H	2638
MOL001880	OXL	Formate acetyltransferase 1	3178

MOL001880	OXL	Adenylosuccinate synthetase	2632
MOL001880	OXL	Succinate dehydrogenase flavoprotein subunit	5725
MOL001880	OXL	Alanine racemase	2453
MOL001880	OXL	Gag-Pol polyprotein	2237
MOL001880	OXL	Methionine synthase	2935
MOL001880	OXL	C4-dicarboxylate transport transcriptional regulatory protein dctD	3336
MOL001880	OXL	D-alanyl-D-alanine carboxypeptidase	2461
MOL001880	OXL	Isocitrate dehydrogenase [NADP]	2286
MOL001880	OXL	Phosphoenolpyruvate carboxykinase [ATP]	4794
MOL001880	OXL	Siroheme synthase	4318
MOL001880	OXL	Aconitate hydratase 2	3553
MOL001880	OXL	Monomeric sarcosine oxidase	2380
MOL001880	OXL	Bacillolysin	2457
MOL001880	OXL	Dihydroxyacetone kinase	2397
MOL001880	OXL	Haloalkane dehalogenase	2281
MOL001880	OXL	Gamma-aminobutyraldehyde dehydrogenase	2881
MOL001880	OXL	Fumarate reductase flavoprotein subunit	2709
MOL001880	OXL	2-isopropylmalate synthase	3179
MOL001880	OXL	1-aminocyclopropane-1-carboxylate deaminase	2518
MOL001880	OXL	Hydroxylamine reductase	3274
MOL001880	OXL	Nicotinate-nucleotide--dimethylbenzimidazole phosphoribosyltransferase	2264
MOL001880	OXL	Glucose--fructose oxidoreductase	2826
MOL001880	OXL	Growth-inhibiting protein 18	3877
MOL001880	OXL		
MOL001880	OXL		
MOL001880	OXL		
MOL001880	OXL	Methylmalonyl-CoA carboxyltransferase 5S subunit	2983
MOL001880	OXL	L-cysteine/cystine lyase C-DES	4802
MOL001880	OXL	Malonamidase E2	4600
MOL001880	OXL	Serotransferrin	566
MOL001880	OXL	Glutamate dehydrogenase 1, mitochondrial	201
MOL001880	OXL	Aspartate aminotransferase, mitochondrial	426
MOL001880	OXL	Pyruvate kinase isozymes M1/M2	98

MOL001880	OXL	Serine--pyruvate aminotransferase	349
MOL001880	OXL	Monocarboxylate transporter 7	195
MOL001880	OXL	Alanine aminotransferase 1	735
MOL001880	OXL	Aldehyde dehydrogenase X, mitochondrial	531
MOL001880	OXL	Succinate dehydrogenase [ubiquinone] flavoprotein subunit, mitochondrial	197
MOL001880	OXL	Cystathionine gamma-lyase	868
MOL001880	OXL	Serine hydroxymethyltransferase, cytosolic	367
MOL001880	OXL	Betaine--homocysteine S-methyltransferase 1	941
MOL001880	OXL	Succinyl-CoA:3-ketoacid-coenzyme A transferase 2, mitochondrial	4008
MOL001880	OXL	Alanine--glyoxylate aminotransferase 2, mitochondrial	114
MOL001880	OXL	NADP-dependent malic enzyme	666
MOL001880	OXL	Glutamate dehydrogenase 2, mitochondrial	830
MOL001880	OXL	Alanyl-tRNA synthetase, cytoplasmic	323
MOL001880	OXL	Glycine amidinotransferase, mitochondrial	383
MOL001880	OXL	Calcium-transporting ATPase type 2C member 1	241
MOL001880	OXL	S-adenosylmethionine synthetase isoform type-1	453
MOL001880	OXL	Cysteine desulfurase, mitochondrial	169
MOL001880	OXL	Alanine--glyoxylate aminotransferase 2-like 2	3899
MOL001880	OXL	Proton-coupled amino acid transporter 1	593
MOL001880	OXL	Adenylosuccinate synthetase isozyme 1	3978
MOL001880	OXL	L-lactate dehydrogenase A chain	473
MOL001880	OXL	Glycine receptor subunit alpha-2	820
MOL001880	OXL	2-amino-3-ketobutyrate coenzyme A ligase, mitochondrial	575
MOL001880	OXL	Pyruvate dehydrogenase E1 component subunit beta, mitochondrial	110
MOL001880	OXL	Triosephosphate isomerase	6346

MOL001880	OXL	Delta-1-pyrroline-5-carboxylate dehydrogenase, mitochondrial	271
MOL001880	OXL	NAD-dependent malic enzyme, mitochondrial	166
MOL001880	OXL	Glycine receptor subunit alpha-3	461
MOL001880	OXL	SHMT2 protein	3884
MOL001880	OXL	NADP-dependent malic enzyme, mitochondrial	363
MOL001880	OXL	L-lactate dehydrogenase B chain	77
MOL001880	OXL	Trypsin-3	2886
MOL001880	OXL	Sigma factor sigB regulation protein rsbQ	3490
MOL001880	OXL	Aromatic-amino-acid aminotransferase	2537
MOL001880	OXL	Aldose reductase	822
MOL001880	OXL	Glycogen phosphorylase, muscle form	1152
MOL001880	OXL	Fumarate hydratase class II	3486
MOL001880	OXL	N-acetylneuraminate lyase	3000
MOL001880	OXL	Phosphate-binding protein pstS precursor	3694
MOL001880	OXL	Beta-amylase	3365
MOL001880	OXL	Tyrosine-protein kinase transforming protein Src	2556
MOL001880	OXL	2-hydroxy-6-oxo-7-methylocta-2,4-dienoate hydrolase	4674
MOL001880	OXL	Phosphotriesterase	5742
MOL001880	OXL	NADH-ubiquinone oxidoreductase 75 kDa subunit, mitochondrial	548
MOL001880	OXL	Gephyrin	4532
MOL001880	OXL	Succinyl-CoA:3-ketoacid-coenzyme A transferase 1, mitochondrial	4003
MOL001880	OXL	Prolyl 4-hydroxylase subunit alpha-2	4000
MOL001880	OXL	Calcium-binding mitochondrial carrier protein Aralar2	513
MOL001880	OXL	Succinyl-CoA ligase [ADP-forming] beta-chain, mitochondrial	4009
MOL001986	β-sitosterol	Progesterone receptor	614
MOL001986	β-sitosterol	Nuclear receptor coactivator 2	6241
MOL001986	β-sitosterol	Mineralocorticoid receptor	737
MOL002230	(+)-Catechin-pentaacetate	Prothrombin	54
MOL002230	(+)-Catechin-pentaacetate	Coagulation factor X	239

MOL002230	(+)-Catechin-pentaacetate	Prostaglandin G/H synthase 2	290
MOL002231	(-)-Epicatechin-pentaacetate	Prothrombin	54
MOL002231	(-)-Epicatechin-pentaacetate	Coagulation factor X	239
MOL002231	(-)-Epicatechin-pentaacetate	Prostaglandin G/H synthase 2	290
MOL002231	(-)-Epicatechin-pentaacetate	Nuclear receptor coactivator 2	6241
MOL002232	2-Cinnamoyl-glucose	Beta-lactamase	2478
MOL002235	EUPATIN	Nitric oxide synthase, inducible	7
MOL002235	EUPATIN	Androgen receptor	146
MOL002235	EUPATIN	Coagulation factor X	239
MOL002235	EUPATIN	Prostaglandin G/H synthase 2	290
MOL002235	EUPATIN	Coagulation factor VII	369
MOL002235	EUPATIN	DNA topoisomerase 2-alpha	817
MOL002235	EUPATIN	Estrogen receptor beta	869
MOL002235	EUPATIN	Dipeptidyl peptidase 4	952
MOL002235	EUPATIN	Heat shock protein HSP 90-alpha	1939
MOL002235	EUPATIN	Trypsin-1	3176
MOL002235	EUPATIN	Nuclear receptor coactivator 2	6241
MOL002235	EUPATIN	Calmodulin	465
MOL002235	EUPATIN	Prothrombin	54
MOL002235	EUPATIN	Sodium channel protein type 5 subunit alpha	220
MOL002235	EUPATIN	Vascular endothelial growth factor receptor 2	407
MOL002235	EUPATIN	Peroxisome proliferator-activated receptor delta	1502
MOL002238	3-Hydroxy-25-norfriedel-3,1(10)-dien-2-one-30-oic acid	Mineralocorticoid receptor	737
MOL002240	5-Carboxy-7-hydroxy-2-methyl-benzopyran-gamma-	Prostaglandin G/H synthase 1	20
MOL002240	5-Carboxy-7-hydroxy-2-methyl-benzopyran-gamma-	Prothrombin	54
MOL002240	5-Carboxy-7-hydroxy-2-methyl-benzopyran-gamma-	Prostaglandin G/H synthase 2	290
MOL002240	5-Carboxy-7-hydroxy-2-methyl-benzopyran-gamma-	Carbonic anhydrase 4	592
MOL002240	5-Carboxy-7-hydroxy-2-methyl-benzopyran-gamma-	Dipeptidyl peptidase 4	952
MOL002240	5-Carboxy-7-hydroxy-2-methyl-benzopyran-gamma-	Heat shock protein HSP 90-alpha	1939
MOL002240	5-Carboxy-7-hydroxy-2-methyl-benzopyran-gamma-	Glutamate receptor 2	921
MOL002243	Anthraglycoside B	Coagulation factor X	239
MOL002243	Anthraglycoside B	DNA topoisomerase 2-alpha	817

MOL002244	Chrysophanol glucoside	Coagulation factor X	239	
MOL002244	Chrysophanol glucoside	Prostaglandin G/H synthase 2	290	
MOL002244	Chrysophanol glucoside	Coagulation factor VII	369	
MOL002244	Chrysophanol glucoside	DNA topoisomerase 2-alpha	817	
MOL002244	Chrysophanol glucoside	Calmodulin	465	
MOL002247	Emodin-6-glucoside	Coagulation factor X	239	
MOL002249	gallocatechin	Prostaglandin G/H synthase 1	20	
MOL002249	gallocatechin	Estrogen receptor	136	
MOL002249	gallocatechin	Prostaglandin G/H synthase 2	290	
MOL002249	gallocatechin	Heat shock protein HSP 90-alpha	1939	
MOL002249	gallocatechin	Nuclear receptor coactivator 2	6241	
MOL002249	gallocatechin	Matrix metalloproteinase-9	h001	validated
MOL002256	1,8-dihydroxy-3-methoxy-2,6-dimethyl-9,10-anthraquinone	Prostaglandin G/H synthase 1	20	
MOL002256	1,8-dihydroxy-3-methoxy-2,6-dimethyl-9,10-anthraquinone	Sodium channel protein type 5 subunit alpha	220	
MOL002256	1,8-dihydroxy-3-methoxy-2,6-dimethyl-9,10-anthraquinone	Coagulation factor X	239	
MOL002256	1,8-dihydroxy-3-methoxy-2,6-dimethyl-9,10-anthraquinone	Prostaglandin G/H synthase 2	290	
MOL002256	1,8-dihydroxy-3-methoxy-2,6-dimethyl-9,10-anthraquinone	Nitric-oxide synthase, endothelial	291	
MOL002256	1,8-dihydroxy-3-methoxy-2,6-dimethyl-9,10-anthraquinone	Coagulation factor VII	369	
MOL002256	1,8-dihydroxy-3-methoxy-2,6-dimethyl-9,10-anthraquinone	Retinoic acid receptor RXR-alpha	459	
MOL002256	1,8-dihydroxy-3-methoxy-2,6-dimethyl-9,10-anthraquinone	DNA topoisomerase 2-alpha	817	
MOL002256	1,8-dihydroxy-3-methoxy-2,6-dimethyl-9,10-anthraquinone	Heat shock protein HSP 90-alpha	1939	
MOL002256	1,8-dihydroxy-3-methoxy-2,6-dimethyl-9,10-anthraquinone	Phosphatidylinositol-4,5-bisphosphate 3-kinase catalytic subunit gamma isoform	2404	
MOL002256	1,8-dihydroxy-3-methoxy-2,6-dimethyl-9,10-anthraquinone	Ig gamma-1 chain C region	4785	

MOL002256	1,8-dihydroxy-3-methoxy-2,6-dimethyl-9,10-anthraquinone	Nuclear receptor coactivator 2	6241
MOL002256	1,8-dihydroxy-3-methoxy-2,6-dimethyl-9,10-anthraquinone	Nuclear receptor coactivator 1	6228
MOL002256	1,8-dihydroxy-3-methoxy-2,6-dimethyl-9,10-anthraquinone	Calmodulin	465
MOL002258	Physcion-9-O-beta-D-glucopyranoside_qt	Prostaglandin G/H synthase 1	20
MOL002258	Physcion-9-O-beta-D-glucopyranoside_qt	Prostaglandin G/H synthase 2	290
MOL002258	Physcion-9-O-beta-D-glucopyranoside_qt	Coagulation factor VII	369
MOL002258	Physcion-9-O-beta-D-glucopyranoside_qt	DNA topoisomerase 2-alpha	817
MOL002258	Physcion-9-O-beta-D-glucopyranoside_qt	Heat shock protein HSP 90-alpha	1939
MOL002258	Physcion-9-O-beta-D-glucopyranoside_qt	Phosphatidylinositol-4,5-bisphosphate 3-kinase catalytic subunit gamma isoform	2404
MOL002258	Physcion-9-O-beta-D-glucopyranoside_qt	Nuclear receptor coactivator 2	6241
MOL002258	Physcion-9-O-beta-D-glucopyranoside_qt	Nuclear receptor coactivator 1	6228
MOL002259	Physciondiglucoside	DNA topoisomerase 2-alpha	817
MOL002261	ZINC04081604	Retinoic acid receptor RXR-alpha	459
MOL002261	ZINC04081604	Progesterone receptor	614
MOL002261	ZINC04081604	Mineralocorticoid receptor	737
MOL002261	ZINC04081604	Nuclear receptor coactivator 2	6241
MOL002261	ZINC04081604	Nuclear receptor coactivator 1	6228
MOL002262	5-[(Z)-2-(3-hydroxy-4-methoxy-phenyl)vinyl]resorcinol	Prostaglandin G/H synthase 1	20
MOL002262	5-[(Z)-2-(3-hydroxy-4-methoxy-phenyl)vinyl]resorcinol	Estrogen receptor	136
MOL002262	5-[(Z)-2-(3-hydroxy-4-methoxy-phenyl)vinyl]resorcinol	Prostaglandin G/H synthase 2	290
MOL002262	5-[(Z)-2-(3-hydroxy-4-methoxy-phenyl)vinyl]resorcinol	Retinoic acid receptor RXR-alpha	459
MOL002262	5-[(Z)-2-(3-hydroxy-4-methoxy-phenyl)vinyl]resorcinol	Beta-2 adrenergic receptor	766

MOL002262	5-[(Z)-2-(3-hydroxy-4-methoxy-phenyl)vinyl]resorcinol	Heat shock protein HSP 90-alpha	1939	
MOL002262	5-[(Z)-2-(3-hydroxy-4-methoxy-phenyl)vinyl]resorcinol	Amine oxidase [flavin-containing] B	3939	
MOL002262	5-[(Z)-2-(3-hydroxy-4-methoxy-phenyl)vinyl]resorcinol	cAMP-dependent protein kinase catalytic subunit alpha	6263	
MOL002262	5-[(Z)-2-(3-hydroxy-4-methoxy-phenyl)vinyl]resorcinol	Nuclear receptor coactivator 2	6241	
MOL002262	5-[(Z)-2-(3-hydroxy-4-methoxy-phenyl)vinyl]resorcinol	cAMP-dependent protein kinase inhibitor alpha	6264	
MOL002267	Rhein diglucoside	DNA topoisomerase 2-alpha	817	
MOL002268	rhein	Prostaglandin G/H synthase 1	20	
MOL002268	rhein	Prostaglandin G/H synthase 2	290	
MOL002268	rhein	Heat shock protein HSP 90-alpha	1939	
MOL002268	rhein	Phosphatidylinositol-4,5-bisphosphate 3-kinase catalytic subunit gamma isoform	2404	
MOL002268	rhein	Nuclear receptor coactivator 2	6241	
MOL002268	rhein	Aldose reductase	822	
MOL002268	rhein	Transcription factor AP-1	1629	validated
MOL002270	Rheinoside A_qt	Carbonic anhydrase 2	357	
MOL002279	Serotonin	Prostaglandin G/H synthase 1	20	
MOL002279	Serotonin	D(1A) dopamine receptor	23	
MOL002279	Serotonin	Muscarinic acetylcholine receptor M3	51	
MOL002279	Serotonin	Muscarinic acetylcholine receptor M1	103	
MOL002279	Serotonin	Beta-1 adrenergic receptor	193	
MOL002279	Serotonin	Sodium channel protein type 5 subunit alpha	220	
MOL002279	Serotonin	Prostaglandin G/H synthase 2	290	
MOL002279	Serotonin	Alpha-2C adrenergic receptor	378	
MOL002279	Serotonin	Alpha-1A adrenergic receptor	556	
MOL002279	Serotonin	Alpha-1B adrenergic receptor	632	
MOL002279	Serotonin	Sodium-dependent dopamine transporter	713	
MOL002279	Serotonin	Beta-2 adrenergic receptor	766	
MOL002279	Serotonin	Leukotriene A-4 hydrolase	3060	

MOL002279	Serotonin	Amine oxidase [flavin-containing] B	3939
MOL002279	Serotonin	cAMP-dependent protein kinase catalytic subunit alpha	6263
MOL002279	Serotonin	cAMP-dependent protein kinase inhibitor alpha	6264
MOL002280	Torachrysone-8-O-beta-D-(6'-oxayl)-glucoside	DNA topoisomerase 2-alpha	817
MOL002281	Toralactone	Nitric oxide synthase, inducible	7
MOL002281	Toralactone	Prostaglandin G/H synthase 1	20
MOL002281	Toralactone	Estrogen receptor	136
MOL002281	Toralactone	Prostaglandin G/H synthase 2	290
MOL002281	Toralactone	Estrogen receptor beta	869
MOL002281	Toralactone	Heat shock protein HSP 90-alpha	1939
MOL002281	Toralactone	Phosphatidylinositol-4,5-bisphosphate 3-kinase catalytic subunit gamma isoform	2404
MOL002281	Toralactone	Serine/threonine-protein kinase Chk1	5790
MOL002281	Toralactone	cAMP-dependent protein kinase catalytic subunit alpha	6263
MOL002283	[(2R,3S,4S,5R,6S)-6-[4-[(Z)-2-(3,5-dihydroxyphenyl)ethenyl]phenoxy]-3,4,5-trihydroxyoxan-2-yl]methyl 3,4,5-	Tyrosine-protein phosphatase non-receptor type 1	687
MOL002284	PIT	Prostaglandin G/H synthase 1	20
MOL002284	PIT	Prostaglandin G/H synthase 2	290
MOL002284	PIT	Amine oxidase [flavin-containing] B	3939
MOL002284	PIT	cAMP-dependent protein kinase catalytic subunit alpha	6263
MOL002285	1-O-Galloyl-glycerol	Prostaglandin G/H synthase 2	290
MOL002286	laccaic acid D	Prostaglandin G/H synthase 1	20
MOL002286	laccaic acid D	Prostaglandin G/H synthase 2	290
MOL002286	laccaic acid D	Phosphatidylinositol-4,5-bisphosphate 3-kinase catalytic subunit gamma isoform	2404

MOL002288	Emodin-1-O-beta-D-glucopyranoside	DNA topoisomerase 2-alpha	817	
MOL002295	cinnamic acid	Prostaglandin G/H synthase 1	20	
MOL002295	cinnamic acid	Prostaglandin G/H synthase 2	290	
MOL002295	cinnamic acid	Amine oxidase [flavin-containing] B	3939	
MOL002295	cinnamic acid	Amine oxidase [flavin-containing] A	3941	
MOL002295	cinnamic acid	Lysozyme	2300	
MOL002295	cinnamic acid	Bacillolysin	2457	
MOL002295	cinnamic acid	Maltase-glucoamylase, intestinal	929	validated
MOL002295	cinnamic acid	Trans-cinnamate 4-monooxygenase	h001	validated
MOL002295	cinnamic acid	Tissue factor	2139	validated
MOL002297	Daucosterol_qt	Progesterone receptor	614	
MOL002297	Daucosterol_qt	Nuclear receptor coactivator 2	6241	
MOL002299	DMR	4-aminobutyrate aminotransferase, mitochondrial	280	
MOL002299	DMR	Aspartate aminotransferase, cytoplasmic	586	
MOL002299	DMR	Tyrosine-protein phosphatase non-receptor type 1	687	
MOL002299	DMR	Aldose reductase	822	
MOL002299	DMR	Gamma-aminobutyric-acid receptor subunit alpha-1	872	
MOL002299	DMR	Cathepsin D	1243	
MOL002299	DMR			
MOL002299	DMR			
MOL002299	DMR	Aspartate aminotransferase	2249	
MOL002299	DMR	Ferrichrome-iron receptor	2427	
MOL002299	DMR	Adenylosuccinate synthetase	2632	
MOL002299	DMR	Bacillolysin	2457	
MOL002299	DMR	1-aminocyclopropane-1-carboxylate deaminase	2518	
MOL002299	DMR	Growth-inhibiting protein 18	3877	
MOL002299	DMR	Aspartate aminotransferase, mitochondrial	426	
MOL002299	DMR	S-adenosylmethionine synthetase isoform type-2	334	
MOL002299	DMR	Prolyl 3-hydroxylase 3	3951	
MOL002299	DMR	S-adenosylmethionine synthetase isoform type-1	453	
MOL002299	DMR	Triosephosphate isomerase	6346	
MOL002299	DMR	NAD-dependent malic enzyme, mitochondrial	166	

MOL002299	DMR	Proto-oncogene tyrosine-protein kinase Src	933
MOL002299	DMR	Cholinesterase	3923
MOL002299	DMR	Cytochrome P450-cam	2298
MOL002299	DMR	Formate acetyltransferase 1	3178
MOL002299	DMR	Nicotinate-nucleotide--dimethylbenzimidazole phosphoribosyltransferase	2264
MOL002299	DMR	Adenylosuccinate synthetase isozyme 1	3978
MOL002300	10beta-Hydroxy-6beta-isobutyrylfuranoeremophilane	Muscarinic acetylcholine receptor M3	51
MOL002300	10beta-Hydroxy-6beta-isobutyrylfuranoeremophilane	Prothrombin	54
MOL002300	10beta-Hydroxy-6beta-isobutyrylfuranoeremophilane	Muscarinic acetylcholine receptor M1	103
MOL002300	10beta-Hydroxy-6beta-isobutyrylfuranoeremophilane	Prostaglandin G/H synthase 2	290
MOL002300	10beta-Hydroxy-6beta-isobutyrylfuranoeremophilane	Gamma-aminobutyric-acid receptor subunit alpha-1	872
MOL002300	10beta-Hydroxy-6beta-isobutyrylfuranoeremophilane	Dipeptidyl peptidase 4	952
MOL002300	10beta-Hydroxy-6beta-isobutyrylfuranoeremophilane	Neuronal acetylcholine receptor subunit alpha-7	4095
MOL002301	DLA	Prostaglandin G/H synthase 1	20
MOL002301	DLA	Nitric-oxide synthase, brain	76
MOL002301	DLA	Succinate semialdehyde dehydrogenase, mitochondrial	170
MOL002301	DLA	4-aminobutyrate aminotransferase, mitochondrial	280
MOL002301	DLA	DNA polymerase	338
MOL002301	DLA	Glutamate [NMDA] receptor subunit zeta-1	401
MOL002301	DLA	Kynureninase	435
MOL002301	DLA	Glycine receptor subunit alpha-1	482
MOL002301	DLA	Aspartate aminotransferase, cytoplasmic	586
MOL002301	DLA	Glutamate receptor 1	705
MOL002301	DLA	Branched-chain-amino-acid aminotransferase, mitochondrial	795

MOL002301	DLA	Gamma-aminobutyric-acid receptor subunit alpha-1	872
MOL002301	DLA	Proto-oncogene tyrosine-protein kinase Src	933
MOL002301	DLA	Glycogen phosphorylase, muscle form	1152
MOL002301	DLA	Cathepsin D	1243
MOL002301	DLA	Neutrophil collagenase	2154
MOL002301	DLA	Macrophage metalloelastase	2203
MOL002301	DLA	UDP-glucose 4-epimerase	2762
MOL002301	DLA	M-phase inducer phosphatase 2	3105
MOL002301	DLA	Thioredoxin reductase 1, cytoplasmic	3610
MOL002301	DLA	Cholinesterase	3923
MOL002301	DLA	Xanthine dehydrogenase/oxidase	3947
MOL002301	DLA	Chymotrypsin-like elastase family member 1	6260
MOL002301	DLA		
MOL002301	DLA		
MOL002301	DLA		
MOL002301	DLA		
MOL002301	DLA		
MOL002301	DLA		
MOL002301	DLA		
MOL002301	DLA		
MOL002301	DLA	Phosphonoacetaldehyde hydrolase	5363
MOL002301	DLA	Cytochrome P450-cam	2298
MOL002301	DLA	Aspartate aminotransferase	2249
MOL002301	DLA	Beta-galactosidase	2592
MOL002301	DLA	Fumarate hydratase class II	3486
MOL002301	DLA	Ferrichrome-iron receptor	2427
MOL002301	DLA	Histidinol dehydrogenase	3191
MOL002301	DLA	Acetyl-CoA acetyltransferase	2596
MOL002301	DLA	Formate acetyltransferase 1	3178
MOL002301	DLA	Adenylosuccinate synthetase	2632
MOL002301	DLA	Beta-lactamase SHV-1 precursor	3687
MOL002301	DLA	Beta-lactamase SHV-1	6691
MOL002301	DLA	Alanine racemase	2453
MOL002301	DLA	Gag-Pol polyprotein	2237
MOL002301	DLA	D-alanyl-D-alanine carboxypeptidase	2461
MOL002301	DLA	Monomeric sarcosine oxidase	2380
MOL002301	DLA	Bacillolysin	2457
MOL002301	DLA	Dihydroxyacetone kinase	2397
MOL002301	DLA	Haloalkane dehalogenase	2281
MOL002301	DLA	Gamma-aminobutyraldehyde dehydrogenase	2881
MOL002301	DLA	2-isopropylmalate synthase	3179

MOL002301	DLA	2-hydroxy-6-oxo-7-methylocta-2,4-dienoate hydrolase	4674
MOL002301	DLA	1-aminocyclopropane-1-carboxylate deaminase	2518
MOL002301	DLA	Nicotinate-nucleotide--dimethylbenzimidazole phosphoribosyltransferase	2264
MOL002301	DLA	Glucose--fructose oxidoreductase	2826
MOL002301	DLA	Growth-inhibiting protein 18	3877
MOL002301	DLA	Methylmalonyl-CoA carboxyltransferase 5S subunit	2983
MOL002301	DLA	Phosphotriesterase	5742
MOL002301	DLA	Malonamidase E2	4600
MOL002301	DLA	Coagulation factor XIII A chain	6
MOL002301	DLA	Aspartate aminotransferase, mitochondrial	426
MOL002301	DLA	Pyruvate kinase isozymes M1/M2	98
MOL002301	DLA	Serine--pyruvate aminotransferase	349
MOL002301	DLA	Alanine aminotransferase 1	735
MOL002301	DLA	Cystathionine gamma-lyase	868
MOL002301	DLA	Glycine N-methyltransferase	411
MOL002301	DLA	Choline dehydrogenase, mitochondrial	700
MOL002301	DLA	Alanine--glyoxylate aminotransferase 2, mitochondrial	114
MOL002301	DLA	Gephyrin	4532
MOL002301	DLA	NADP-dependent malic enzyme	666
MOL002301	DLA	Alanyl-tRNA synthetase, cytoplasmic	323
MOL002301	DLA	Glycine amidinotransferase, mitochondrial	383
MOL002301	DLA	Cysteine desulfurase, mitochondrial	169
MOL002301	DLA	Alanine--glyoxylate aminotransferase 2-like 2	3899
MOL002301	DLA	Proton-coupled amino acid transporter 1	593
MOL002301	DLA	Adenylosuccinate synthetase isozyme 1	3978
MOL002301	DLA	L-lactate dehydrogenase A chain	473
MOL002301	DLA	2-amino-3-ketobutyrate coenzyme A ligase, mitochondrial	575
MOL002301	DLA	Triosephosphate isomerase	6346

MOL002301	DLA	NAD-dependent malic enzyme, mitochondrial	166
MOL002301	DLA	5-aminolevulinate synthase, erythroid-specific, mitochondrial	1088
MOL002301	DLA	Calcium-binding mitochondrial carrier protein Aralar2	513
MOL002301	DLA	NADP-dependent malic enzyme, mitochondrial	363
MOL002301	DLA	Trypsin-3	2886
MOL002301	DLA	Prostaglandin G/H synthase 2	290
MOL002301	DLA	Urease alpha subunit	370
MOL002301	DLA	Ornithine aminotransferase, mitochondrial	472
MOL002301	DLA	Pyruvate dehydrogenase [cytochrome]	845
MOL002301	DLA	Beta-lactamase SHV-2 precursor	3701
MOL002301	DLA	Alpha-ketoglutarate-dependent taurine dioxygenase	2529
MOL002301	DLA	Betaine--homocysteine S-methyltransferase 1	941
MOL002301	DLA	Pyruvate dehydrogenase E1 component subunit beta, mitochondrial	110
MOL002301	DLA	SHMT2 protein	3884
MOL002301	DLA	Methylmalonyl-CoA carboxyltransferase 12S subunit	3240
MOL002301	DLA	L-cysteine/cystine lyase C-DES	4802
MOL002301	DLA	Peroxisomal sarcosine oxidase	4061
MOL002301	DLA	L-lactate dehydrogenase B chain	77
MOL002302	RHAPONTIN	Prostaglandin G/H synthase 2	290
MOL000346	succinic acid	4-aminobutyrate aminotransferase, mitochondrial	280
MOL000346	succinic acid	Aspartate aminotransferase, cytoplasmic	586
MOL000346	succinic acid	Branched-chain-amino-acid aminotransferase, mitochondrial	795
MOL000346	succinic acid	Aldose reductase	822
MOL000346	succinic acid	Proto-oncogene tyrosine-protein kinase Src	933
MOL000346	succinic acid	Cathepsin D	1243

MOL000346	succinic acid	M-phase inducer phosphatase 2	3105
MOL000346	succinic acid	Cholinesterase	3923
MOL000346	succinic acid		
MOL000346	succinic acid		
MOL000346	succinic acid		
MOL000346	succinic acid	Cytochrome P450-cam	2298
MOL000346	succinic acid	Aspartate aminotransferase	2249
MOL000346	succinic acid	Ferrichrome-iron receptor	2427
MOL000346	succinic acid	Monomeric sarcosine oxidase	2380
MOL000346	succinic acid	Bacillolysin	2457
MOL000346	succinic acid	Aromatic-amino-acid aminotransferase	2537
MOL000346	succinic acid	1-aminocyclopropane-1-carboxylate deaminase	2518
MOL000346	succinic acid	Growth-inhibiting protein 18	3877
MOL000346	succinic acid	Aspartate aminotransferase, mitochondrial	426
MOL000346	succinic acid	S-adenosylmethionine synthetase isoform type-1	453
MOL000346	succinic acid	Triosephosphate isomerase	6346
MOL000346	succinic acid	NAD-dependent malic enzyme, mitochondrial	166
MOL000346	succinic acid	NADP-dependent malic enzyme, mitochondrial	363
MOL000346	succinic acid	Trypsin-3	2886
MOL000346	succinic acid	Nitric-oxide synthase, brain	76
MOL000346	succinic acid	Succinate semialdehyde dehydrogenase, mitochondrial	170
MOL000346	succinic acid	Ornithine aminotransferase, mitochondrial	472
MOL000346	succinic acid	Glycine receptor subunit alpha-1	482
MOL000346	succinic acid	Tyrosine-protein phosphatase non-receptor type 1	687
MOL000346	succinic acid	Gamma-aminobutyric-acid receptor subunit alpha-1	872
MOL000346	succinic acid	Thioredoxin reductase 1, cytoplasmic	3610
MOL000346	succinic acid	cAMP-dependent protein kinase catalytic subunit alpha	6263
MOL000346	succinic acid	Acetyl-CoA acetyltransferase	2596
MOL000346	succinic acid	Formate acetyltransferase 1	3178
MOL000346	succinic acid	Adenylosuccinate synthetase	2632
MOL000346	succinic acid	Glucarate dehydratase	3708
MOL000346	succinic acid	D-alanyl-D-alanine carboxypeptidase	2461

MOL000346	succinic acid	Deacetoxycephalosporin C synthetase	3334	
MOL000346	succinic acid	Malate dehydrogenase	2329	
MOL000346	succinic acid	Fumarate reductase flavoprotein subunit	2709	
MOL000346	succinic acid	2-isopropylmalate synthase	3179	
MOL000346	succinic acid	Nicotinate-nucleotide--dimethylbenzimidazole phosphoribosyltransferase	2264	
MOL000346	succinic acid	Malonamidase E2	4600	
MOL000346	succinic acid	Glutamate dehydrogenase 1, mitochondrial	201	
MOL000346	succinic acid	Ornithine carbamoyltransferase, mitochondrial	336	
MOL000346	succinic acid	Pyruvate kinase isozymes M1/M2	98	
MOL000346	succinic acid	Serine--pyruvate aminotransferase	349	
MOL000346	succinic acid	Oxidoreductase	1410	
MOL000346	succinic acid	Succinate dehydrogenase [ubiquinone] flavoprotein subunit, mitochondrial	197	
MOL000346	succinic acid	S-adenosylmethionine synthetase isoform type-2	334	
MOL000346	succinic acid	Prolyl 3-hydroxylase 1	3945	
MOL000346	succinic acid	Prolyl 3-hydroxylase 3	3951	
MOL000346	succinic acid	Succinyl-CoA:3-ketoacid-coenzyme A transferase 2, mitochondrial	4008	
MOL000346	succinic acid	Mitochondrial dicarboxylate carrier	4010	
MOL000346	succinic acid	Prolyl 4-hydroxylase subunit alpha-2	4000	
MOL000346	succinic acid	Succinyl-CoA ligase [ADP-forming] beta-chain, mitochondrial	4009	
MOL000346	succinic acid	Haloalkane dehalogenase	2281	
MOL000346	succinic acid	Glucose--fructose oxidoreductase	2826	
MOL000346	succinic acid	Caspase-3	h001	validated
MOL000358	beta-sitosterol	Progesterone receptor	614	
MOL000358	beta-sitosterol	Nuclear receptor coactivator 2	6241	
MOL000358	beta-sitosterol	Prostaglandin G/H synthase 1	20	
MOL000358	beta-sitosterol	Prostaglandin G/H synthase 2	290	
MOL000358	beta-sitosterol	Heat shock protein HSP 90-alpha	1939	

MOL000358	beta-sitosterol	Phosphatidylinositol-4,5-bisphosphate 3-kinase catalytic subunit gamma isoform	2404	
MOL000358	beta-sitosterol	Potassium voltage-gated channel subfamily H member 2	101	
MOL000358	beta-sitosterol	cAMP-dependent protein kinase catalytic subunit alpha	6263	
MOL000358	beta-sitosterol	D(1A) dopamine receptor	23	
MOL000358	beta-sitosterol	Muscarinic acetylcholine receptor M3	51	
MOL000358	beta-sitosterol	Muscarinic acetylcholine receptor M1	103	
MOL000358	beta-sitosterol	Sodium channel protein type 5 subunit alpha	220	
MOL000358	beta-sitosterol	Gamma-aminobutyric-acid receptor subunit alpha-2	423	
MOL000358	beta-sitosterol	Muscarinic acetylcholine receptor M4	450	
MOL000358	beta-sitosterol	cGMP-inhibited 3',5'-cyclic phosphodiesterase A	485	
MOL000358	beta-sitosterol	5-hydroxytryptamine 2A receptor	502	
MOL000358	beta-sitosterol	Gamma-aminobutyric-acid receptor subunit alpha-5	523	
MOL000358	beta-sitosterol	Alpha-1A adrenergic receptor	556	
MOL000358	beta-sitosterol	Gamma-aminobutyric-acid receptor subunit alpha-3	580	
MOL000358	beta-sitosterol	Muscarinic acetylcholine receptor M2	617	
MOL000358	beta-sitosterol	Alpha-1B adrenergic receptor	632	
MOL000358	beta-sitosterol	Beta-2 adrenergic receptor	766	
MOL000358	beta-sitosterol	Neuronal acetylcholine receptor subunit alpha-2	813	
MOL000358	beta-sitosterol	Sodium-dependent serotonin transporter	824	
MOL000358	beta-sitosterol	Mu-type opioid receptor	847	
MOL000358	beta-sitosterol	Gamma-aminobutyric-acid receptor subunit alpha-1	872	
MOL000358	beta-sitosterol	Neuronal acetylcholine receptor subunit alpha-7	4095	
MOL000358	beta-sitosterol	Cytochrome P450-cam	2298	
MOL000358	beta-sitosterol	Apoptosis regulator Bcl-2	273	validated
MOL000358	beta-sitosterol	Apoptosis regulator BAX	h001	validated
MOL000358	beta-sitosterol	Caspase-9	h001	validated
MOL000358	beta-sitosterol	Transcription factor AP-1	1629	validated
MOL000358	beta-sitosterol	Caspase-3	h001	validated
MOL000358	beta-sitosterol	Caspase-8	h001	validated
MOL000358	beta-sitosterol	Protein kinase C alpha type	h001	validated

MOL000358	beta-sitosterol	Transforming growth factor beta-1	h001	validated
MOL000358	beta-sitosterol	Serum paraoxonase/arylesterase 1	1198	validated
MOL000358	beta-sitosterol	Microtubule-associated protein 2	1852	validated
MOL000471	aloe-emodin	Prostaglandin G/H synthase 1	20	
MOL000471	aloe-emodin	Prostaglandin G/H synthase 2	290	
MOL000471	aloe-emodin	Heat shock protein HSP 90-alpha	1939	
MOL000471	aloe-emodin	Phosphatidylinositol-4,5-bisphosphate 3-kinase catalytic subunit gamma isoform	2404	
MOL000471	aloe-emodin	cAMP-dependent protein kinase catalytic subunit alpha	6263	
MOL000471	aloe-emodin	Nuclear receptor coactivator 2	6241	
MOL000471	aloe-emodin	cAMP-dependent protein kinase inhibitor alpha	6264	
MOL000471	aloe-emodin	Aldose reductase	822	
MOL000471	aloe-emodin	Ig gamma-1 chain C region	4785	
MOL000471	aloe-emodin	Cyclin-dependent kinase inhibitor 1	h001	validated
MOL000471	aloe-emodin	Eukaryotic translation initiation factor 6	h001	validated
MOL000471	aloe-emodin	Apoptosis regulator BAX	h001	validated
MOL000471	aloe-emodin	Tumor necrosis factor	777	validated
MOL000471	aloe-emodin	Caspase-3	h001	validated
MOL000471	aloe-emodin	Cellular tumor antigen p53	5788	validated
MOL000471	aloe-emodin	Fatty acid synthase	1295	validated
MOL000471	aloe-emodin	Protein kinase C alpha type	h001	validated
MOL000471	aloe-emodin	Protein kinase C epsilon type	h001	validated
MOL000471	aloe-emodin	Cell division control protein 2 homolog	1771	validated
MOL000471	aloe-emodin	Proliferating cell nuclear antigen	h001	validated
MOL000471	aloe-emodin	Myc proto-oncogene protein	h001	validated
MOL000471	aloe-emodin	Interleukin-1 beta	1654	validated
MOL000471	aloe-emodin	Protein kinase C delta type	h001	validated
MOL000471	aloe-emodin	G2/mitotic-specific cyclin-B1	h001	validated
MOL000472	emodin	Prostaglandin G/H synthase 1	20	
MOL000472	emodin	Prostaglandin G/H synthase 2	290	
MOL000472	emodin	Coagulation factor VII	369	
MOL000472	emodin	Heat shock protein HSP 90-alpha	1939	

MOL000472	emodin	Phosphatidylinositol-4,5-bisphosphate 3-kinase catalytic subunit gamma isoform	2404	
MOL000472	emodin	cAMP-dependent protein kinase catalytic subunit alpha	6263	
MOL000472	emodin	Ig gamma-1 chain C region	4785	
MOL000472	emodin	Coagulation factor X	239	
MOL000472	emodin	Vascular endothelial growth factor receptor 2	407	validated
MOL000472	emodin	DNA topoisomerase 2-alpha	817	
MOL000472	emodin	Nuclear receptor coactivator 2	6241	
MOL000472	emodin	Nuclear receptor coactivator 1	6228	
MOL000472	emodin	Calmodulin	465	
MOL000472	emodin	Cyclin-dependent kinase inhibitor 1	h001	validated
MOL000472	emodin	Vascular endothelial growth factor receptor 1	32	validated
MOL000472	emodin	Matrix metalloproteinase-9	h001	validated
MOL000472	emodin			validated
MOL000472	emodin	Tumor necrosis factor	777	validated
MOL000472	emodin	Caspase-3	h001	validated
MOL000472	emodin	Cellular tumor antigen p53	5788	validated
MOL000472	emodin	Protein kinase C epsilon type	h001	validated
MOL000472	emodin	Interstitial collagenase	1167	validated
MOL000472	emodin	Peroxisome proliferator-activated receptor gamma	h001	validated
MOL000472	emodin	Myc proto-oncogene protein	h001	validated
MOL000472	emodin	Cytochrome P450 1A1	h001	validated
MOL000472	emodin	Interleukin-1 beta	1654	validated
MOL000472	emodin	Protein kinase C delta type	h001	validated
MOL000472	emodin	Granulocyte-macrophage colony-stimulating factor	h001	validated
MOL000472	emodin	Transforming growth factor beta-1	h001	validated
MOL000472	emodin	Actin, aortic smooth muscle	h001	validated
MOL000472	emodin	Amine oxidase [flavin-containing] B	3939	validated
MOL000472	emodin	Tyrosine-protein kinase BTK	2355	validated
MOL000472	emodin	Solute carrier family 2, facilitated glucose transporter member 4	h001	validated
MOL000472	emodin	Vascular endothelial growth factor receptor 3	26	validated
MOL000472	emodin	Solute carrier family 2, facilitated glucose transporter member 1	h001	validated
MOL000476	Physcion	Prostaglandin G/H synthase 1	20	

MOL000476	Physcion	Sodium channel protein type 5 subunit alpha	220	
MOL000476	Physcion	Prostaglandin G/H synthase 2	290	
MOL000476	Physcion	Nitric-oxide synthase, endothelial	291	
MOL000476	Physcion	Coagulation factor VII	369	
MOL000476	Physcion	DNA topoisomerase 2-alpha	817	
MOL000476	Physcion	Heat shock protein HSP 90-alpha	1939	
MOL000476	Physcion	Phosphatidylinositol-4,5-bisphosphate 3-kinase catalytic subunit gamma isoform	2404	
MOL000476	Physcion	Beta-lactamase	2478	
MOL000476	Physcion	Ig gamma-1 chain C region	4785	
MOL000476	Physcion	Nuclear receptor coactivator 2	6241	
MOL000476	Physcion	Nuclear receptor coactivator 1	6228	
MOL000476	Physcion	cAMP-dependent protein kinase inhibitor alpha	6264	
MOL000476	Physcion	Calmodulin	465	
MOL000476	Physcion	cAMP-dependent protein kinase catalytic subunit alpha	6263	
MOL000476	Physcion	Coagulation factor X	239	
MOL000476	Physcion	Retinoic acid receptor RXR-alpha	459	
MOL000513	3,4,5-trihydroxybenzoic acid	Prostaglandin G/H synthase 1	20	
MOL000513	3,4,5-trihydroxybenzoic acid	Prostaglandin G/H synthase 2	290	
MOL000513	3,4,5-trihydroxybenzoic acid	Amine oxidase [flavin-containing] B	3939	
MOL000513	3,4,5-trihydroxybenzoic acid	Progesterone receptor	614	
MOL000513	3,4,5-trihydroxybenzoic acid	Tyrosine-protein phosphatase non-receptor type 1	687	
MOL000513	3,4,5-trihydroxybenzoic acid	DNA topoisomerase 2-alpha	817	
MOL000513	3,4,5-trihydroxybenzoic acid	Heat shock protein HSP 90-alpha	1939	
MOL000513	3,4,5-trihydroxybenzoic acid	Phosphatidylinositol-4,5-bisphosphate 3-kinase catalytic subunit gamma isoform	2404	
MOL000513	3,4,5-trihydroxybenzoic acid	Caspase-9	h001	validated
MOL000513	3,4,5-trihydroxybenzoic acid	Caspase-3	h001	validated

MOL000513	3,4,5-trihydroxybenzoic acid	Cellular tumor antigen p53	5788	validated
MOL000513	3,4,5-trihydroxybenzoic acid	Fatty acid synthase	1295	validated
MOL000513	3,4,5-trihydroxybenzoic acid	Tumor necrosis factor ligand superfamily member 6	h001	validated
MOL000513	3,4,5-trihydroxybenzoic acid	Microsomal glutathione S-transferase 1	4032	validated
MOL000513	3,4,5-trihydroxybenzoic acid	Cytochrome P450 3A43	h001	validated
MOL000096	(-)-catechin	Prostaglandin G/H synthase 1	20	
MOL000096	(-)-catechin	Estrogen receptor	136	
MOL000096	(-)-catechin	Prostaglandin G/H synthase 2	290	
MOL000096	(-)-catechin	Heat shock protein HSP 90-alpha	1939	
MOL000096	(-)-catechin	Beta-lactamase	2478	
MOL000096	(-)-catechin	cAMP-dependent protein kinase catalytic subunit alpha	6263	
MOL000096	(-)-catechin	Nuclear receptor coactivator 2	6241	
MOL000096	(-)-catechin	Calmodulin	465	
MOL000096	(-)-catechin	Fatty acid synthase	1295	validated
MOL000096	(-)-catechin	Peroxisome proliferator-activated receptor gamma	h001	validated
MOL000096	(-)-catechin	Krueppel-like factor 7	h001	validated
