# Supplementary material for: The Study of Yin-Chen-Hao-Tang Preventing and Treating Alcoholic Fatty Liver Disease through PPAR Signaling Pathway Based on Network Pharmacology and RNA-Seq Transcriptomics
Source: Evid Based Complement Alternat Med. 2021 Dec 31;2021:8917993. doi: 10.1155/2021/8917993 (PMC8741355; doi:10.1155/2021/8917993)
Supplement: Supplementary Materials — Supplementary Material 1-1: Herb Target-Artemisiae Scopariae Herba. Supplementary Material 1-2: Herb Target-Gardeniae Fructus. Supplementary Material 1-3: Herb Target-Radix Rhei Et Rhizome. Supplementary Material 2: AFLD-GeneCards-SearchResults. Supplementary Material 3: string_interactions.tsv default node. [file 8917993.f1.zip › 8917993.f1/Supplementary material 2-AFLD-GeneCards-SearchResults.pdf]

Gene Symbol	Description	Category	Gifts	GC Id	Relevance score	GeneCards Link
PKD1	Polycystin 1, Transient Receptor Potential Channel Interacting	Protein Coding	46	GC16M003057	119.5238	https://www.genecards.org/cgi-bin/carddisp.pl?gene=PKD1
IL6	Interleukin 6	Protein Coding	50	GC07P022725	101.6283	https://www.genecards.org/cgi-bin/carddisp.pl?gene=IL6
TNF	Tumor Necrosis Factor	Protein Coding	51	GC06P055202	101.4095	https://www.genecards.org/cgi-bin/carddisp.pl?gene=TNF
PNPLA3	Patatin Like Phospholipase Domain Containing 3	Protein Coding	41	GC22P043923	100.9991	https://www.genecards.org/cgi-bin/carddisp.pl?gene=PNPLA3
APOE	Apolipoprotein E	Protein Coding	51	GC19P044906	99.73659	https://www.genecards.org/cgi-bin/carddisp.pl?gene=APOE
TP53	Tumor Protein P53	Protein Coding	54	GC17M007661	98.49773	https://www.genecards.org/cgi-bin/carddisp.pl?gene=TP53
PRMT7	Protein Arginine Methyltransferase 7	Protein Coding	45	GC16P068363	94.93203	https://www.genecards.org/cgi-bin/carddisp.pl?gene=PRMT7
PKHD1	PKHD1 Ciliary IPT Domain Containing Fibrocystin/Polyductin	Protein Coding	38	GC06M051588	94.50678	https://www.genecards.org/cgi-bin/carddisp.pl?gene=PKHD1
HFE	Homeostatic Iron Regulator	Protein Coding	44	GC06P026087	93.73489	https://www.genecards.org/cgi-bin/carddisp.pl?gene=HFE
PKD2	Polycystin 2, Transient Receptor Potential Cation Channel	Protein Coding	47	GC04P088007	90.89617	https://www.genecards.org/cgi-bin/carddisp.pl?gene=PKD2
ADH1C	Alcohol Dehydrogenase 1C (Class I), Gamma Polypeptide	Protein Coding	43	GC04M099336	89.8614	https://www.genecards.org/cgi-bin/carddisp.pl?gene=ADH1C
MIR122	MicroRNA 122	RNA Gene	21	GC18P058451	89.85336	https://www.genecards.org/cgi-bin/carddisp.pl?gene=MIR122
ALB	Albumin	Protein Coding	50	GC04P073397	88.7054	https://www.genecards.org/cgi-bin/carddisp.pl?gene=ALB
INS	Insulin	Protein Coding	48	GC11M002159	87.77376	https://www.genecards.org/cgi-bin/carddisp.pl?gene=INS
ALDH2	Aldehyde Dehydrogenase 2 Family Member	Protein Coding	51	GC12P111766	87.5976	https://www.genecards.org/cgi-bin/carddisp.pl?gene=ALDH2
ADH1B	Alcohol Dehydrogenase 1B (Class I), Beta Polypeptide	Protein Coding	44	GC04M099304	85.45002	https://www.genecards.org/cgi-bin/carddisp.pl?gene=ADH1B

IL10	Interleukin 10	Protein Coding	48	GC01M206767	83.81952	https://www.genecards.org/cgi-bin/carddisp.pl?gene=IL10
PPARG	Peroxisome Proliferator Activated Receptor Gamma	Protein Coding	53	GC03P012287	79.53107	https://www.genecards.org/cgi-bin/carddisp.pl?gene=PPARG
SEC63	SEC63 Homolog, Protein Translocation Regulator	Protein Coding	44	GC06M107867	79.07613	https://www.genecards.org/cgi-bin/carddisp.pl?gene=SEC63
SLC17A5	Solute Carrier Family 17 Member 5	Protein Coding	45	GC06M073593	78.82408	https://www.genecards.org/cgi-bin/carddisp.pl?gene=SLC17A5
HADHA	Hydroxyacyl-CoA Dehydrogenase Trifunctional Multienzyme Complex Subunit Alpha	Protein Coding	47	GC02M026190	78.51592	https://www.genecards.org/cgi-bin/carddisp.pl?gene=HADHA
GBA	Glucosylceramidase Beta	Protein Coding	48	GC01M155234	76.74422	https://www.genecards.org/cgi-bin/carddisp.pl?gene=GBA
TGFB1	Transforming Growth Factor Beta 1	Protein Coding	52	GC19M041301	76.22275	https://www.genecards.org/cgi-bin/carddisp.pl?gene=TGFB1
CYP2E1	Cytochrome P450 Family 2 Subfamily E Member 1	Protein Coding	46	GC10P133520	75.4827	https://www.genecards.org/cgi-bin/carddisp.pl?gene=CYP2E1
GPT	Glutamic--Pyruvic Transaminase	Protein Coding	43	GC08P144502	73.7998	https://www.genecards.org/cgi-bin/carddisp.pl?gene=GPT
LMNA	Lamin A/C	Protein Coding	48	GC01P156082	72.939	https://www.genecards.org/cgi-bin/carddisp.pl?gene=LMNA
PRKCSH	Protein Kinase C Substrate 80K-H	Protein Coding	44	GC19P011435	72.54431	https://www.genecards.org/cgi-bin/carddisp.pl?gene=PRKCSH
NPC1	NPC Intracellular Cholesterol Transporter 1	Protein Coding	47	GC18M023506	72.08344	https://www.genecards.org/cgi-bin/carddisp.pl?gene=NPC1
MARS1	Methionyl-TRNA Synthetase 1	Protein Coding	36	GC12P057476	71.26236	https://www.genecards.org/cgi-bin/carddisp.pl?gene=MARS1
FABP1	Fatty Acid Binding Protein 1	Protein Coding	44	GC02M088122	71.22694	https://www.genecards.org/cgi-bin/carddisp.pl?gene=FABP1
MIR21	MicroRNA 21	RNA Gene	24	GC17P059841	70.34363	https://www.genecards.org/cgi-bin/carddisp.pl?gene=MIR21
SNCA	Synuclein Alpha	Protein Coding	50	GC04M089724	69.74733	https://www.genecards.org/cgi-bin/carddisp.pl?gene=SNCA
SERPINA1	Serpin Family A Member 1	Protein Coding	50	GC14M094376	69.68977	https://www.genecards.org/cgi-bin/carddisp.pl?gene=SERPINA1
IL1B	Interleukin 1 Beta	Protein Coding	49	GC02M112829	68.79674	https://www.genecards.org/cgi-bin/carddisp.pl?gene=IL1B

CTNNB1	Catenin Beta 1	Protein Coding	54	GC03P041236	68.70273	https://www.genecards.org/cgi-bin/carddisp.pl?gene=CTNNB1
APC	APC Regulator Of WNT Signaling Pathway	Protein Coding	49	GC05P112707	68.14858	https://www.genecards.org/cgi-bin/carddisp.pl?gene=APC
SMPD1	Sphingomyelin Phosphodiesterase 1	Protein Coding	48	GC11P006390	68.0873	https://www.genecards.org/cgi-bin/carddisp.pl?gene=SMPD1
FAS	Fas Cell Surface Death Receptor	Protein Coding	51	GC10P088969	68.04547	https://www.genecards.org/cgi-bin/carddisp.pl?gene=FAS
GAA	Alpha Glucosidase	Protein Coding	48	GC17P080101	67.76856	https://www.genecards.org/cgi-bin/carddisp.pl?gene=GAA
FASN	Fatty Acid Synthase	Protein Coding	50	GC17M082078	66.77557	https://www.genecards.org/cgi-bin/carddisp.pl?gene=FASN
ATP7B	ATPase Copper Transporting Beta	Protein Coding	48	GC13M051930	66.68053	https://www.genecards.org/cgi-bin/carddisp.pl?gene=ATP7B
PIK3CA	Phosphatidylinositol-4,5-Bisphosphate 3-Kinase Catalytic Subunit Alpha	Protein Coding	53	GC03P179148	66.29842	https://www.genecards.org/cgi-bin/carddisp.pl?gene=PIK3CA
TSC2	TSC Complex Subunit 2	Protein Coding	50	GC16P005579	65.77246	https://www.genecards.org/cgi-bin/carddisp.pl?gene=TSC2
GGT1	Gamma-Glutamyltransferase 1	Protein Coding	48	GC22P024583	65.13819	https://www.genecards.org/cgi-bin/carddisp.pl?gene=GGT1
APOB	Apolipoprotein B	Protein Coding	46	GC02M020956	64.52831	https://www.genecards.org/cgi-bin/carddisp.pl?gene=APOB
APOA1	Apolipoprotein A1	Protein Coding	49	GC11M116835	64.36289	https://www.genecards.org/cgi-bin/carddisp.pl?gene=APOA1
PPARA	Peroxisome Proliferator Activated Receptor Alpha	Protein Coding	47	GC22P046150	64.11192	https://www.genecards.org/cgi-bin/carddisp.pl?gene=PPARA
GANAB	Glucosidase II Alpha Subunit	Protein Coding	45	GC11M069251	63.74379	https://www.genecards.org/cgi-bin/carddisp.pl?gene=GANAB
MIR34A	MicroRNA 34a	RNA Gene	22	GC01M009151	63.60819	https://www.genecards.org/cgi-bin/carddisp.pl?gene=MIR34A
ABCB11	ATP Binding Cassette Subfamily B Member 11	Protein Coding	47	GC02M168922	63.43503	https://www.genecards.org/cgi-bin/carddisp.pl?gene=ABCB11
CRP	C-Reactive Protein	Protein Coding	47	GC01M159719	62.44139	https://www.genecards.org/cgi-bin/carddisp.pl?gene=CRP
LRP5	LDL Receptor Related Protein 5	Protein Coding	49	GC11P068298	62.32442	https://www.genecards.org/cgi-bin/carddisp.pl?gene=LRP5
CPT2	Carnitine Palmitoyltransferase 2	Protein Coding	50	GC01P053196	61.98357	https://www.genecards.org/cgi-bin/carddisp.pl?gene=CPT2
SQSTM1	Sequestosome 1	Protein Coding	49	GC05P179806	61.87291	https://www.genecards.org/cgi-bin/carddisp.pl?gene=SQSTM1

APP	Amyloid Beta Precursor Protein	Protein Coding	52	GC21M025880	61.60009	https://www.genecards.org/cgi-bin/carddisp.pl?gene=APP
F2	Coagulation Factor II, Thrombin	Protein Coding	49	GC11P046720	61.5752	https://www.genecards.org/cgi-bin/carddisp.pl?gene=F2
ADIPOQ	Adiponectin, C1Q And Collagen Domain Containing	Protein Coding	45	GC03P186842	61.53323	https://www.genecards.org/cgi-bin/carddisp.pl?gene=ADIPOQ
PSEN1	Presenilin 1	Protein Coding	52	GC14P073136	61.30258	https://www.genecards.org/cgi-bin/carddisp.pl?gene=PSEN1
TLR4	Toll Like Receptor 4	Protein Coding	51	GC09P117704	61.23509	https://www.genecards.org/cgi-bin/carddisp.pl?gene=TLR4
CFTR	CF Transmembrane Conductance Regulator	Protein Coding	52	GC07P117287	61.18417	https://www.genecards.org/cgi-bin/carddisp.pl?gene=CFTR
PTEN	Phosphatase And Tensin Homolog	Protein Coding	53	GC10P087863	60.41596	https://www.genecards.org/cgi-bin/carddisp.pl?gene=PTEN
KRAS	KRAS Proto-Oncogene, GTPase	Protein Coding	52	GC12M025204	60.32327	https://www.genecards.org/cgi-bin/carddisp.pl?gene=KRAS
ABCA1	ATP Binding Cassette Subfamily A Member 1	Protein Coding	48	GC09M104781	60.32098	https://www.genecards.org/cgi-bin/carddisp.pl?gene=ABCA1
NOD2	Nucleotide Binding Oligomerization Domain Containing 2	Protein Coding	48	GC16P050693	60.2951	https://www.genecards.org/cgi-bin/carddisp.pl?gene=NOD2
KRT18	Keratin 18	Protein Coding	49	GC12P052948	60.13712	https://www.genecards.org/cgi-bin/carddisp.pl?gene=KRT18
CPT1A	Carnitine Palmitoyltransferase 1A	Protein Coding	50	GC11M068754	60.10578	https://www.genecards.org/cgi-bin/carddisp.pl?gene=CPT1A
MFN2	Mitofusin 2	Protein Coding	48	GC01P011980	59.97812	https://www.genecards.org/cgi-bin/carddisp.pl?gene=MFN2
CXCL8	C-X-C Motif Chemokine Ligand 8	Protein Coding	42	GC04P073740	59.72943	https://www.genecards.org/cgi-bin/carddisp.pl?gene=CXCL8
NAFLD1	Fatty Liver Disease 1, Susceptiblity To	Genetic Locus	1	GC22U901005	59.54738	https://www.genecards.org/cgi-bin/carddisp.pl?gene=NAFLD1
LPL	Lipoprotein Lipase	Protein Coding	50	GC08P019901	59.30823	https://www.genecards.org/cgi-bin/carddisp.pl?gene=LPL
AKT1	AKT Serine/Threonine Kinase 1	Protein Coding	54	GC14M104769	59.26334	https://www.genecards.org/cgi-bin/carddisp.pl?gene=AKT1
G6PC1	Glucose-6-Phosphatase Catalytic Subunit 1	Protein Coding	36	GC17P044746	59.18401	https://www.genecards.org/cgi-bin/carddisp.pl?gene=G6PC1

MIR17	MicroRNA 17	RNA Gene	21	GC13P091350	59.14647	https://www.genecards.org/cgi-bin/carddisp.pl?gene=MIR17
NR1H4	Nuclear Receptor Subfamily 1 Group H Member 4	Protein Coding	49	GC12P100473	57.76836	https://www.genecards.org/cgi-bin/carddisp.pl?gene=NR1H4
LIPA	Lipase A, Lysosomal Acid Type	Protein Coding	48	GC10M089213	57.73469	https://www.genecards.org/cgi-bin/carddisp.pl?gene=LIPA
MIR155	MicroRNA 155	RNA Gene	19	GC21P025560	57.37925	https://www.genecards.org/cgi-bin/carddisp.pl?gene=MIR155
POLG	DNA Polymerase Gamma, Catalytic Subunit	Protein Coding	46	GC15M089365	57.32057	https://www.genecards.org/cgi-bin/carddisp.pl?gene=POLG
ACE	Angiotensin I Converting Enzyme	Protein Coding	50	GC17P063477	57.08172	https://www.genecards.org/cgi-bin/carddisp.pl?gene=ACE
MIR10B	MicroRNA 10b	RNA Gene	21	GC02P176150	56.82254	https://www.genecards.org/cgi-bin/carddisp.pl?gene=MIR10B
LEP	Leptin	Protein Coding	48	GC07P128241	56.78732	https://www.genecards.org/cgi-bin/carddisp.pl?gene=LEP
HNF4A	Hepatocyte Nuclear Factor 4 Alpha	Protein Coding	51	GC20P044355	56.78619	https://www.genecards.org/cgi-bin/carddisp.pl?gene=HNF4A
MPZ	Myelin Protein Zero	Protein Coding	44	GC01M161304	56.69935	https://www.genecards.org/cgi-bin/carddisp.pl?gene=MPZ
VWF	Von Willebrand Factor	Protein Coding	49	GC12M005917	56.60173	https://www.genecards.org/cgi-bin/carddisp.pl?gene=VWF
CTLA4	Cytotoxic T-Lymphocyte Associated Protein 4	Protein Coding	46	GC02P203867	56.58133	https://www.genecards.org/cgi-bin/carddisp.pl?gene=CTLA4
LRRK2	Leucine Rich Repeat Kinase 2	Protein Coding	50	GC12P040196	55.73726	https://www.genecards.org/cgi-bin/carddisp.pl?gene=LRRK2
PRKN	Parkin RBR E3 Ubiquitin Protein Ligase	Protein Coding	40	GC06M161348	55.72185	https://www.genecards.org/cgi-bin/carddisp.pl?gene=PRKN
STAT3	Signal Transducer And Activator Of Transcription 3	Protein Coding	53	GC17M042313	55.53656	https://www.genecards.org/cgi-bin/carddisp.pl?gene=STAT3
MTHFR	Methylenetetrahydrofolate Reductase	Protein Coding	49	GC01M011785	55.03107	https://www.genecards.org/cgi-bin/carddisp.pl?gene=MTHFR
IFNG	Interferon Gamma	Protein Coding	48	GC12M068154	54.84436	https://www.genecards.org/cgi-bin/carddisp.pl?gene=IFNG
PHKA2	Phosphorylase Kinase Regulatory Subunit Alpha 2	Protein Coding	46	GC0XM018892	54.79215	https://www.genecards.org/cgi-bin/carddisp.pl?gene=PHKA2

ACADM	Acyl-CoA Dehydrogenase Medium Chain	Protein Coding	47	GC01P075724	54.48516	https://www.genecards.org/cgi-bin/carddisp.pl?gene=ACADM
HADHB	Hydroxyacyl-CoA Dehydrogenase Trifunctional Multienzyme Complex Subunit Beta	Protein Coding	48	GC02P026243	54.44437	https://www.genecards.org/cgi-bin/carddisp.pl?gene=HADHB
PSAP	Prosaposin	Protein Coding	47	GC10M071816	54.37947	https://www.genecards.org/cgi-bin/carddisp.pl?gene=PSAP
RET	Ret Proto-Oncogene	Protein Coding	54	GC10P043081	54.16558	https://www.genecards.org/cgi-bin/carddisp.pl?gene=RET
CD36	CD36 Molecule	Protein Coding	49	GC07P080369	54.08582	https://www.genecards.org/cgi-bin/carddisp.pl?gene=CD36
HLA-DRB1	Major Histocompatibility Complex, Class II, DR Beta 1	Protein Coding	48	GC06M032578	54.01691	https://www.genecards.org/cgi-bin/carddisp.pl?gene=HLA-DRB1
AGL	Amylo-Alpha-1, 6-Glucosidase, 4-Alpha-Glucanotransferase	Protein Coding	45	GC01P099850	53.58754	https://www.genecards.org/cgi-bin/carddisp.pl?gene=AGL
TERT	Telomerase Reverse Transcriptase	Protein Coding	52	GC05M001253	53.33697	https://www.genecards.org/cgi-bin/carddisp.pl?gene=TERT
VCP	Valosin Containing Protein	Protein Coding	50	GC09M035056	53.28416	https://www.genecards.org/cgi-bin/carddisp.pl?gene=VCP
INSR	Insulin Receptor	Protein Coding	54	GC19M007112	53.28331	https://www.genecards.org/cgi-bin/carddisp.pl?gene=INSR
CASP8	Caspase 8	Protein Coding	52	GC02P201233	53.08566	https://www.genecards.org/cgi-bin/carddisp.pl?gene=CASP8
MIR146A	MicroRNA 146a	RNA Gene	22	GC05P160485	53.00844	https://www.genecards.org/cgi-bin/carddisp.pl?gene=MIR146A
NOS3	Nitric Oxide Synthase 3	Protein Coding	52	GC07P150990	52.36965	https://www.genecards.org/cgi-bin/carddisp.pl?gene=NOS3
PRNP	Prion Protein	Protein Coding	47	GC20P004686	52.19698	https://www.genecards.org/cgi-bin/carddisp.pl?gene=PRNP
PYGL	Glycogen Phosphorylase L	Protein Coding	49	GC14M050857	52.19163	https://www.genecards.org/cgi-bin/carddisp.pl?gene=PYGL
MTTP	Microsomal Triglyceride Transfer Protein	Protein Coding	45	GC04P099563	52.15165	https://www.genecards.org/cgi-bin/carddisp.pl?gene=MTTP
FAR1	Fatty Acyl-CoA Reductase 1	Protein Coding	43	GC11P013668	51.87154	https://www.genecards.org/cgi-bin/carddisp.pl?gene=FAR1

SREBF1	Sterol Regulatory Element Binding Transcription Factor 1	Protein Coding	46	GC17M017810	51.58896	https://www.genecards.org/cgi-bin/carddisp.pl?gene=SREBF1
EGF	Epidermal Growth Factor	Protein Coding	51	GC04P109912	51.46554	https://www.genecards.org/cgi-bin/carddisp.pl?gene=EGF
TSC1	TSC Complex Subunit 1	Protein Coding	48	GC09M132891	51.2425	https://www.genecards.org/cgi-bin/carddisp.pl?gene=TSC1
EGFR	Epidermal Growth Factor Receptor	Protein Coding	54	GC07P055019	51.1895	https://www.genecards.org/cgi-bin/carddisp.pl?gene=EGFR
HNF1A	HNF1 Homeobox A	Protein Coding	46	GC12P120978	50.89312	https://www.genecards.org/cgi-bin/carddisp.pl?gene=HNF1A
ACOX1	Acyl-CoA Oxidase 1	Protein Coding	47	GC17M075941	50.42818	https://www.genecards.org/cgi-bin/carddisp.pl?gene=ACOX1
GBE1	1,4-Alpha-Glucan Branching Enzyme 1	Protein Coding	45	GC03M081489	50.25403	https://www.genecards.org/cgi-bin/carddisp.pl?gene=GBE1
TRPV4	Transient Receptor Potential Cation Channel Subfamily V Member 4	Protein Coding	50	GC12M109783	50.16294	https://www.genecards.org/cgi-bin/carddisp.pl?gene=TRPV4
DRD2	Dopamine Receptor D2	Protein Coding	51	GC11M113409	50.1232	https://www.genecards.org/cgi-bin/carddisp.pl?gene=DRD2
MAPT	Microtubule Associated Protein Tau	Protein Coding	51	GC17P045894	49.91683	https://www.genecards.org/cgi-bin/carddisp.pl?gene=MAPT
FABP4	Fatty Acid Binding Protein 4	Protein Coding	45	GC08M081478	49.90357	https://www.genecards.org/cgi-bin/carddisp.pl?gene=FABP4
TTR	Transthyretin	Protein Coding	50	GC18P031557	49.79599	https://www.genecards.org/cgi-bin/carddisp.pl?gene=TTR
AFP	Alpha Fetoprotein	Protein Coding	46	GC04P073431	49.74606	https://www.genecards.org/cgi-bin/carddisp.pl?gene=AFP
AKR1A1	Aldo-Keto Reductase Family 1 Member A1	Protein Coding	45	GC01P045550	49.6741	https://www.genecards.org/cgi-bin/carddisp.pl?gene=AKR1A1
GJB1	Gap Junction Protein Beta 1	Protein Coding	48	GC0XP071212	49.54929	https://www.genecards.org/cgi-bin/carddisp.pl?gene=GJB1
FABP2	Fatty Acid Binding Protein 2	Protein Coding	43	GC04M119317	49.53653	https://www.genecards.org/cgi-bin/carddisp.pl?gene=FABP2
CYP3A4	Cytochrome P450 Family 3 Subfamily A Member 4	Protein Coding	51	GC07M099759	49.52491	https://www.genecards.org/cgi-bin/carddisp.pl?gene=CYP3A4
LOC106627981	GBA Recombination Region	Biological Region	2	GC01P155233	49.46788	https://www.genecards.org/cgi-bin/carddisp.pl?gene=LOC106627981
SOD1	Superoxide Dismutase 1	Protein Coding	52	GC21P031659	49.46487	https://www.genecards.org/cgi-bin/carddisp.pl?gene=SOD1

IGF1	Insulin Like Growth Factor 1	Protein Coding	50	GC12M102395	49.23305	https://www.genecards.org/cgi-bin/carddisp.pl?gene=IGF1
NR1H3	Nuclear Receptor Subfamily 1 Group H Member 3	Protein Coding	48	GC11P047248	49.15171	https://www.genecards.org/cgi-bin/carddisp.pl?gene=NR1H3
SERPINE1	Serpin Family E Member 1	Protein Coding	51	GC07P101127	48.93843	https://www.genecards.org/cgi-bin/carddisp.pl?gene=SERPINE1
ABCA4	ATP Binding Cassette Subfamily A Member 4	Protein Coding	45	GC01M093992	48.93377	https://www.genecards.org/cgi-bin/carddisp.pl?gene=ABCA4
RBP4	Retinol Binding Protein 4	Protein Coding	45	GC10M093591	48.77829	https://www.genecards.org/cgi-bin/carddisp.pl?gene=RBP4
CYBB	Cytochrome B-245 Beta Chain	Protein Coding	47	GC0XP037780	48.75913	https://www.genecards.org/cgi-bin/carddisp.pl?gene=CYBB
KRT8	Keratin 8	Protein Coding	48	GC12M052897	48.65049	https://www.genecards.org/cgi-bin/carddisp.pl?gene=KRT8
ABCB4	ATP Binding Cassette Subfamily B Member 4	Protein Coding	47	GC07M087401	48.33384	https://www.genecards.org/cgi-bin/carddisp.pl?gene=ABCB4
GLA	Galactosidase Alpha	Protein Coding	49	GC0XM101393	48.26838	https://www.genecards.org/cgi-bin/carddisp.pl?gene=GLA
TLR2	Toll Like Receptor 2	Protein Coding	52	GC04P153684	48.07245	https://www.genecards.org/cgi-bin/carddisp.pl?gene=TLR2
BRAF	B-Raf Proto-Oncogene, Serine/Threonine Kinase	Protein Coding	54	GC07M140717	47.7293	https://www.genecards.org/cgi-bin/carddisp.pl?gene=BRAF
ELOVL4	ELOVL Fatty Acid Elongase 4	Protein Coding	46	GC06M079914	47.67517	https://www.genecards.org/cgi-bin/carddisp.pl?gene=ELOVL4
GDAP1	Ganglioside Induced Differentiation Associated Protein 1	Protein Coding	43	GC08P074315	47.34433	https://www.genecards.org/cgi-bin/carddisp.pl?gene=GDAP1
PON1	Paraoxonase 1	Protein Coding	46	GC07M095297	47.30381	https://www.genecards.org/cgi-bin/carddisp.pl?gene=PON1
TRMU	TRNA Mitochondrial 2-Thiouridylase	Protein Coding	40	GC22P046330	47.26027	https://www.genecards.org/cgi-bin/carddisp.pl?gene=TRMU
BDNF	Brain Derived Neurotrophic Factor	Protein Coding	48	GC11M027654	47.24964	https://www.genecards.org/cgi-bin/carddisp.pl?gene=BDNF
SLC25A13	Solute Carrier Family 25 Member 13	Protein Coding	47	GC07M096120	47.23321	https://www.genecards.org/cgi-bin/carddisp.pl?gene=SLC25A13
GYS2	Glycogen Synthase 2	Protein Coding	44	GC12M021531	46.79419	https://www.genecards.org/cgi-bin/carddisp.pl?gene=GYS2

CCL2	C-C Motif Chemokine Ligand 2	Protein Coding	49	GC17P034255	46.73888	https://www.genecards.org/cgi-bin/carddisp.pl?gene=CCL2
HLA-DQB1	Major Histocompatibility Complex, Class II, DQ Beta 1	Protein Coding	43	GC06M046939	46.662	https://www.genecards.org/cgi-bin/carddisp.pl?gene=HLA-DQB1
CASP3	Caspase 3	Protein Coding	51	GC04M184627	46.52601	https://www.genecards.org/cgi-bin/carddisp.pl?gene=CASP3
TF	Transferrin	Protein Coding	50	GC03P133666	46.46587	https://www.genecards.org/cgi-bin/carddisp.pl?gene=TF
NEFL	Neurofilament Light Chain	Protein Coding	47	GC08M024950	46.25078	https://www.genecards.org/cgi-bin/carddisp.pl?gene=NEFL
IL1A	Interleukin 1 Alpha	Protein Coding	44	GC02M112773	46.23853	https://www.genecards.org/cgi-bin/carddisp.pl?gene=IL1A
ABCD1	ATP Binding Cassette Subfamily D Member 1	Protein Coding	47	GC0XP153724	46.18417	https://www.genecards.org/cgi-bin/carddisp.pl?gene=ABCD1
RYR1	Ryanodine Receptor 1	Protein Coding	47	GC19P040452	46.17769	https://www.genecards.org/cgi-bin/carddisp.pl?gene=RYR1
HMOX1	Heme Oxygenase 1	Protein Coding	53	GC22P035380	46.1692	https://www.genecards.org/cgi-bin/carddisp.pl?gene=HMOX1
MIR29A	MicroRNA 29a	RNA Gene	22	GC07M130876	46.14713	https://www.genecards.org/cgi-bin/carddisp.pl?gene=MIR29A
MET	MET Proto-Oncogene, Receptor Tyrosine Kinase	Protein Coding	54	GC07P116672	46.14655	https://www.genecards.org/cgi-bin/carddisp.pl?gene=MET
HNF1B	HNF1 Homeobox B	Protein Coding	44	GC17M037686	45.97813	https://www.genecards.org/cgi-bin/carddisp.pl?gene=HNF1B
NBAS	NBAS Subunit Of NRZ Tethering Complex	Protein Coding	40	GC02M014998	45.46504	https://www.genecards.org/cgi-bin/carddisp.pl?gene=NBAS
MIR15A	MicroRNA 15a	RNA Gene	16	GC13M050049	45.45919	https://www.genecards.org/cgi-bin/carddisp.pl?gene=MIR15A
MIR27A	MicroRNA 27a	RNA Gene	22	GC19M014109	45.27167	https://www.genecards.org/cgi-bin/carddisp.pl?gene=MIR27A
PEX6	Peroxisomal Biogenesis Factor 6	Protein Coding	43	GC06M042963	45.21988	https://www.genecards.org/cgi-bin/carddisp.pl?gene=PEX6
NPC2	NPC Intracellular Cholesterol Transporter 2	Protein Coding	42	GC14M074476	45.16733	https://www.genecards.org/cgi-bin/carddisp.pl?gene=NPC2
MPO	Myeloperoxidase	Protein Coding	51	GC17M058269	45.08788	https://www.genecards.org/cgi-bin/carddisp.pl?gene=MPO
LDLR	Low Density Lipoprotein Receptor	Protein Coding	50	GC19P011091	45.07767	https://www.genecards.org/cgi-bin/carddisp.pl?gene=LDLR
ADH4	Alcohol Dehydrogenase 4 (Class II), Pi Polypeptide	Protein Coding	45	GC04M099123	44.93248	https://www.genecards.org/cgi-bin/carddisp.pl?gene=ADH4

IRS1	Insulin Receptor Substrate 1	Protein Coding	48	GC02M226731	44.90195	https://www.genecards.org/cgi-bin/carddisp.pl?gene=IRS1
VEGFA	Vascular Endothelial Growth Factor A	Protein Coding	49	GC06P043770	44.88901	https://www.genecards.org/cgi-bin/carddisp.pl?gene=VEGFA
HBB	Hemoglobin Subunit Beta	Protein Coding	46	GC11M005434	44.88221	https://www.genecards.org/cgi-bin/carddisp.pl?gene=HBB
SH3TC2	SH3 Domain And Tetratricopeptide Repeats 2	Protein Coding	38	GC05M148923	44.60126	https://www.genecards.org/cgi-bin/carddisp.pl?gene=SH3TC2
JAK2	Janus Kinase 2	Protein Coding	54	GC09P004985	44.59028	https://www.genecards.org/cgi-bin/carddisp.pl?gene=JAK2
GFAP	Glial Fibrillary Acidic Protein	Protein Coding	48	GC17M044905	44.53488	https://www.genecards.org/cgi-bin/carddisp.pl?gene=GFAP
BRCA2	BRCA2 DNA Repair Associated	Protein Coding	50	GC13P032315	44.44099	https://www.genecards.org/cgi-bin/carddisp.pl?gene=BRCA2
FBN1	Fibrillin 1	Protein Coding	47	GC15M048408	44.03257	https://www.genecards.org/cgi-bin/carddisp.pl?gene=FBN1
LEPR	Leptin Receptor	Protein Coding	50	GC01P065421	43.93019	https://www.genecards.org/cgi-bin/carddisp.pl?gene=LEPR
CEP290	Centrosomal Protein 290	Protein Coding	41	GC12M088049	43.91979	https://www.genecards.org/cgi-bin/carddisp.pl?gene=CEP290
MIR200B	MicroRNA 200b	RNA Gene	20	GC01P001167	43.83263	https://www.genecards.org/cgi-bin/carddisp.pl?gene=MIR200B
TNFRSF1A	TNF Receptor Superfamily Member 1A	Protein Coding	50	GC12M006328	43.65583	https://www.genecards.org/cgi-bin/carddisp.pl?gene=TNFRSF1A
PHKG2	Phosphorylase Kinase Catalytic Subunit Gamma 2	Protein Coding	47	GC16P032474	43.65182	https://www.genecards.org/cgi-bin/carddisp.pl?gene=PHKG2
UGT1A1	UDP Glucuronosyltransferase Family 1 Member A1	Protein Coding	49	GC02P233760	43.62955	https://www.genecards.org/cgi-bin/carddisp.pl?gene=UGT1A1
HTR2A	5-Hydroxytryptamine Receptor 2A	Protein Coding	48	GC13M046831	43.61702	https://www.genecards.org/cgi-bin/carddisp.pl?gene=HTR2A
MIR143	MicroRNA 143	RNA Gene	23	GC05P149410	43.47362	https://www.genecards.org/cgi-bin/carddisp.pl?gene=MIR143
ACADVL	Acyl-CoA Dehydrogenase Very Long Chain	Protein Coding	46	GC17P007219	43.4342	https://www.genecards.org/cgi-bin/carddisp.pl?gene=ACADVL
COMT	Catechol-O-Methyltransferase	Protein Coding	52	GC22P019941	43.25294	https://www.genecards.org/cgi-bin/carddisp.pl?gene=COMT
HLA-B	Major Histocompatibility Complex, Class I, B	Protein Coding	46	GC06M046832	43.1871	https://www.genecards.org/cgi-bin/carddisp.pl?gene=HLA-B

FADS1	Fatty Acid Desaturase 1	Protein Coding	45	GC11M061799	43.11945	https://www.genecards.org/cgi-bin/carddisp.pl?gene=FADS1
GARS1	Glycyl-TRNA Synthetase 1	Protein Coding	37	GC07P030580	43.09009	https://www.genecards.org/cgi-bin/carddisp.pl?gene=GARS1
MIR203A	MicroRNA 203a	RNA Gene	19	GC14P106054	43.05531	https://www.genecards.org/cgi-bin/carddisp.pl?gene=MIR203A
MIR223	MicroRNA 223	RNA Gene	21	GC0XP066018	43.05186	https://www.genecards.org/cgi-bin/carddisp.pl?gene=MIR223
HSD17B10	Hydroxysteroid 17-Beta Dehydrogenase 10	Protein Coding	46	GC0XM053431	43.02921	https://www.genecards.org/cgi-bin/carddisp.pl?gene=HSD17B10
MMP1	Matrix Metallopeptidase 1	Protein Coding	52	GC11M102810	43.01691	https://www.genecards.org/cgi-bin/carddisp.pl?gene=MMP1
ADH5	Alcohol Dehydrogenase 5 (Class III), Chi Polypeptide	Protein Coding	47	GC04M099070	42.67286	https://www.genecards.org/cgi-bin/carddisp.pl?gene=ADH5
SOD2	Superoxide Dismutase 2	Protein Coding	51	GC06M159669	42.65597	https://www.genecards.org/cgi-bin/carddisp.pl?gene=SOD2
MMP9	Matrix Metallopeptidase 9	Protein Coding	54	GC20P046008	42.55257	https://www.genecards.org/cgi-bin/carddisp.pl?gene=MMP9
RETN	Resistin	Protein Coding	44	GC19P007669	42.46697	https://www.genecards.org/cgi-bin/carddisp.pl?gene=RETN
MT-ND1	Mitochondrially Encoded NADH:Ubiquinone Oxidoreductase Core Subunit 1	Protein Coding	33	GCMTP003309	42.44855	https://www.genecards.org/cgi-bin/carddisp.pl?gene=MT-ND1
PARK7	Parkinsonism Associated Deglycase	Protein Coding	47	GC01P008012	42.37277	https://www.genecards.org/cgi-bin/carddisp.pl?gene=PARK7
BSCL2	BSCL2 Lipid Droplet Biogenesis Associated, Seipin	Protein Coding	43	GC11M069260	42.28667	https://www.genecards.org/cgi-bin/carddisp.pl?gene=BSCL2
PPARGC1A	PPARG Coactivator 1 Alpha	Protein Coding	47	GC04M023755	42.28547	https://www.genecards.org/cgi-bin/carddisp.pl?gene=PPARGC1A
AR	Androgen Receptor	Protein Coding	53	GC0XP067544	42.14416	https://www.genecards.org/cgi-bin/carddisp.pl?gene=AR
CAT	Catalase	Protein Coding	50	GC11P034460	42.04203	https://www.genecards.org/cgi-bin/carddisp.pl?gene=CAT
PMP22	Peripheral Myelin Protein 22	Protein Coding	41	GC17M015229	41.85723	https://www.genecards.org/cgi-bin/carddisp.pl?gene=PMP22
SCD	Stearoyl-CoA Desaturase	Protein Coding	49	GC10P100347	41.82539	https://www.genecards.org/cgi-bin/carddisp.pl?gene=SCD
FABP3	Fatty Acid Binding Protein 3	Protein Coding	44	GC01M031365	41.78497	https://www.genecards.org/cgi-bin/carddisp.pl?gene=FABP3

PEX5	Peroxisomal Biogenesis Factor 5	Protein Coding	43	GC12P011881	41.78151	https://www.genecards.org/cgi-bin/carddisp.pl?gene=PEX5
MIR106B	MicroRNA 106b	RNA Gene	22	GC07M100472	41.65577	https://www.genecards.org/cgi-bin/carddisp.pl?gene=MIR106B
SMAD4	SMAD Family Member 4	Protein Coding	51	GC18P051028	41.64323	https://www.genecards.org/cgi-bin/carddisp.pl?gene=SMAD4
HSPB1	Heat Shock Protein Family B (Small) Member 1	Protein Coding	52	GC07P076302	41.62627	https://www.genecards.org/cgi-bin/carddisp.pl?gene=HSPB1
IL18	Interleukin 18	Protein Coding	44	GC11M112143	41.53468	https://www.genecards.org/cgi-bin/carddisp.pl?gene=IL18
MIR22	MicroRNA 22	RNA Gene	20	GC17M001713	41.40516	https://www.genecards.org/cgi-bin/carddisp.pl?gene=MIR22
PEX1	Peroxisomal Biogenesis Factor 1	Protein Coding	45	GC07M092487	41.33879	https://www.genecards.org/cgi-bin/carddisp.pl?gene=PEX1
CP	Ceruloplasmin	Protein Coding	48	GC03M149162	41.33826	https://www.genecards.org/cgi-bin/carddisp.pl?gene=CP
NAGLU	N-Acetyl-Alpha-Glucosaminidase	Protein Coding	43	GC17P042537	41.32879	https://www.genecards.org/cgi-bin/carddisp.pl?gene=NAGLU
ERBB2	Erb-B2 Receptor Tyrosine Kinase 2	Protein Coding	54	GC17P039687	41.31289	https://www.genecards.org/cgi-bin/carddisp.pl?gene=ERBB2
HAMP	Hepcidin Antimicrobial Peptide	Protein Coding	44	GC19P040344	41.29496	https://www.genecards.org/cgi-bin/carddisp.pl?gene=HAMP
CYP1A1	Cytochrome P450 Family 1 Subfamily A Member 1	Protein Coding	48	GC15M074719	41.27938	https://www.genecards.org/cgi-bin/carddisp.pl?gene=CYP1A1
ABHD5	Abhydrolase Domain Containing 5, Lysophosphatidic Acid Acyltransferase	Protein Coding	45	GC03P043707	41.19246	https://www.genecards.org/cgi-bin/carddisp.pl?gene=ABHD5
NOTCH1	Notch Receptor 1	Protein Coding	51	GC09M136705	41.00478	https://www.genecards.org/cgi-bin/carddisp.pl?gene=NOTCH1
SERPINA3	Serpin Family A Member 3	Protein Coding	44	GC14P094612	40.99447	https://www.genecards.org/cgi-bin/carddisp.pl?gene=SERPINA3
ADH1A	Alcohol Dehydrogenase 1A (Class I), Alpha Polypeptide	Protein Coding	43	GC04M099276	40.94531	https://www.genecards.org/cgi-bin/carddisp.pl?gene=ADH1A
AARS1	Alanyl-TRNA Synthetase 1	Protein Coding	37	GC16M070343	40.93534	https://www.genecards.org/cgi-bin/carddisp.pl?gene=AARS1
HSD17B4	Hydroxysteroid 17-Beta Dehydrogenase 4	Protein Coding	47	GC05P119452	40.9006	https://www.genecards.org/cgi-bin/carddisp.pl?gene=HSD17B4

JAG1	Jagged Canonical Notch Ligand 1	Protein Coding	51	GC20M010637	40.87424	https://www.genecards.org/cgi-bin/carddisp.pl?gene=JAG1
FAAH	Fatty Acid Amide Hydrolase	Protein Coding	48	GC01P046394	40.82689	https://www.genecards.org/cgi-bin/carddisp.pl?gene=FAAH
HTT	Huntingtin	Protein Coding	44	GC04P003041	40.73323	https://www.genecards.org/cgi-bin/carddisp.pl?gene=HTT
FADS2	Fatty Acid Desaturase 2	Protein Coding	45	GC11P061792	40.73115	https://www.genecards.org/cgi-bin/carddisp.pl?gene=FADS2
GSTM1	Glutathione S-Transferase Mu 1	Protein Coding	43	GC01P109687	40.72124	https://www.genecards.org/cgi-bin/carddisp.pl?gene=GSTM1
MIR126	MicroRNA 126	RNA Gene	22	GC09P136670	40.57684	https://www.genecards.org/cgi-bin/carddisp.pl?gene=MIR126
LIPC	Lipase C, Hepatic Type	Protein Coding	46	GC15P058410	40.48891	https://www.genecards.org/cgi-bin/carddisp.pl?gene=LIPC
PSEN2	Presenilin 2	Protein Coding	50	GC01P226870	40.48117	https://www.genecards.org/cgi-bin/carddisp.pl?gene=PSEN2
GABRA2	Gamma-Aminobutyric Acid Type A Receptor Subunit Alpha2	Protein Coding	47	GC04M046243	40.41587	https://www.genecards.org/cgi-bin/carddisp.pl?gene=GABRA2
IGF2	Insulin Like Growth Factor 2	Protein Coding	48	GC11M002130	40.3887	https://www.genecards.org/cgi-bin/carddisp.pl?gene=IGF2
ICAM1	Intercellular Adhesion Molecule 1	Protein Coding	51	GC19P010270	40.3847	https://www.genecards.org/cgi-bin/carddisp.pl?gene=ICAM1
SLC6A4	Solute Carrier Family 6 Member 4	Protein Coding	48	GC17M030194	40.3251	https://www.genecards.org/cgi-bin/carddisp.pl?gene=SLC6A4
FA2H	Fatty Acid 2-Hydroxylase	Protein Coding	45	GC16M074712	40.22961	https://www.genecards.org/cgi-bin/carddisp.pl?gene=FA2H
IL2	Interleukin 2	Protein Coding	45	GC04M122451	40.22802	https://www.genecards.org/cgi-bin/carddisp.pl?gene=IL2
CCND1	Cyclin D1	Protein Coding	52	GC11P069641	40.22279	https://www.genecards.org/cgi-bin/carddisp.pl?gene=CCND1
HGF	Hepatocyte Growth Factor	Protein Coding	52	GC07M081699	40.17495	https://www.genecards.org/cgi-bin/carddisp.pl?gene=HGF
MIR20A	MicroRNA 20a	RNA Gene	19	GC13P091444	40.15267	https://www.genecards.org/cgi-bin/carddisp.pl?gene=MIR20A
GDNF	Glial Cell Derived Neurotrophic Factor	Protein Coding	48	GC05M037812	40.03215	https://www.genecards.org/cgi-bin/carddisp.pl?gene=GDNF
BRCA1	BRCA1 DNA Repair Associated	Protein Coding	51	GC17M043044	40.025	https://www.genecards.org/cgi-bin/carddisp.pl?gene=BRCA1
IL1RN	Interleukin 1 Receptor Antagonist	Protein Coding	48	GC02P116694	39.97456	https://www.genecards.org/cgi-bin/carddisp.pl?gene=IL1RN
ESR1	Estrogen Receptor 1	Protein Coding	53	GC06P151656	39.92862	https://www.genecards.org/cgi-bin/carddisp.pl?gene=ESR1

FASLG	Fas Ligand	Protein Coding	48	GC01P172628	39.92387	https://www.genecards.org/cgi-bin/carddisp.pl?gene=FASLG
ADH7	Alcohol Dehydrogenase 7 (Class IV), Mu Or Sigma Polypeptide	Protein Coding	45	GC04M099412	39.8848	https://www.genecards.org/cgi-bin/carddisp.pl?gene=ADH7
ABCB1	ATP Binding Cassette Subfamily B Member 1	Protein Coding	52	GC07M087504	39.84828	https://www.genecards.org/cgi-bin/carddisp.pl?gene=ABCB1
FARSB	Phenylalanyl-TRNA Synthetase Subunit Beta	Protein Coding	45	GC02M222570	39.83929	https://www.genecards.org/cgi-bin/carddisp.pl?gene=FARSB
PRX	Periaxin	Protein Coding	40	GC19M040393	39.81201	https://www.genecards.org/cgi-bin/carddisp.pl?gene=PRX
CC2D2A	Coiled-Coil And C2 Domain Containing 2A	Protein Coding	38	GC04P015471	39.7771	https://www.genecards.org/cgi-bin/carddisp.pl?gene=CC2D2A
F5	Coagulation Factor V	Protein Coding	45	GC01M169511	39.71928	https://www.genecards.org/cgi-bin/carddisp.pl?gene=F5
MAPK1	Mitogen-Activated Protein Kinase 1	Protein Coding	52	GC22M021754	39.71365	https://www.genecards.org/cgi-bin/carddisp.pl?gene=MAPK1
YARS1	Tyrosyl-TRNA Synthetase 1	Protein Coding	37	GC01M032776	39.64629	https://www.genecards.org/cgi-bin/carddisp.pl?gene=YARS1
DNM2	Dynamin 2	Protein Coding	50	GC19P010718	39.50795	https://www.genecards.org/cgi-bin/carddisp.pl?gene=DNM2
HADH	Hydroxyacyl-CoA Dehydrogenase	Protein Coding	47	GC04P107989	39.49352	https://www.genecards.org/cgi-bin/carddisp.pl?gene=HADH
IGF2R	Insulin Like Growth Factor 2 Receptor	Protein Coding	46	GC06P159969	39.49142	https://www.genecards.org/cgi-bin/carddisp.pl?gene=IGF2R
UCP2	Uncoupling Protein 2	Protein Coding	46	GC11M073974	39.47613	https://www.genecards.org/cgi-bin/carddisp.pl?gene=UCP2
PINK1	PTEN Induced Kinase 1	Protein Coding	48	GC01P020634	39.441	https://www.genecards.org/cgi-bin/carddisp.pl?gene=PINK1
IGHMBP2	Immunoglobulin Mu DNA Binding Protein 2	Protein Coding	42	GC11P068903	39.37398	https://www.genecards.org/cgi-bin/carddisp.pl?gene=IGHMBP2
ALDH3A2	Aldehyde Dehydrogenase 3 Family Member A2	Protein Coding	45	GC17P019648	39.27642	https://www.genecards.org/cgi-bin/carddisp.pl?gene=ALDH3A2
DYNC1H1	Dynein Cytoplasmic 1 Heavy Chain 1	Protein Coding	45	GC14P106032	39.27556	https://www.genecards.org/cgi-bin/carddisp.pl?gene=DYNC1H1
TMEM67	Transmembrane Protein 67	Protein Coding	38	GC08P093754	39.24709	https://www.genecards.org/cgi-bin/carddisp.pl?gene=TMEM67
HEXA	Hexosaminidase Subunit Alpha	Protein Coding	46	GC15M072340	39.15423	https://www.genecards.org/cgi-bin/carddisp.pl?gene=HEXA

PTGS2	Prostaglandin-Endoperoxide Synthase 2	Protein Coding	49	GC01M186640	39.11338	https://www.genecards.org/cgi-bin/carddisp.pl?gene=PTGS2
PLA2G6	Phospholipase A2 Group VI	Protein Coding	48	GC22M049636	39.06322	https://www.genecards.org/cgi-bin/carddisp.pl?gene=PLA2G6
CAV1	Caveolin 1	Protein Coding	48	GC07P116524	39.05238	https://www.genecards.org/cgi-bin/carddisp.pl?gene=CAV1
FIG4	FIG4 Phosphoinositide 5-Phosphatase	Protein Coding	44	GC06P109691	39.04974	https://www.genecards.org/cgi-bin/carddisp.pl?gene=FIG4
MIR221	MicroRNA 221	RNA Gene	20	GC0XM045746	38.90244	https://www.genecards.org/cgi-bin/carddisp.pl?gene=MIR221
MTOR	Mechanistic Target Of Rapamycin Kinase	Protein Coding	54	GC01M011106	38.86319	https://www.genecards.org/cgi-bin/carddisp.pl?gene=MTOR
ATP8B1	ATPase Phospholipid Transporting 8B1	Protein Coding	40	GC18M057646	38.82219	https://www.genecards.org/cgi-bin/carddisp.pl?gene=ATP8B1
CFH	Complement Factor H	Protein Coding	46	GC01P196621	38.72898	https://www.genecards.org/cgi-bin/carddisp.pl?gene=CFH
SPP1	Secreted Phosphoprotein 1	Protein Coding	47	GC04P087975	38.65092	https://www.genecards.org/cgi-bin/carddisp.pl?gene=SPP1
ABCC2	ATP Binding Cassette Subfamily C Member 2	Protein Coding	48	GC10P099782	38.58338	https://www.genecards.org/cgi-bin/carddisp.pl?gene=ABCC2
CYBA	Cytochrome B-245 Alpha Chain	Protein Coding	48	GC16M088643	38.52029	https://www.genecards.org/cgi-bin/carddisp.pl?gene=CYBA
ASAH1	N-Acylsphingosine Amidohydrolase 1	Protein Coding	47	GC08M018055	38.43977	https://www.genecards.org/cgi-bin/carddisp.pl?gene=ASAH1
VHL	Von Hippel-Lindau Tumor Suppressor	Protein Coding	47	GC03P011015	38.43132	https://www.genecards.org/cgi-bin/carddisp.pl?gene=VHL
IL4	Interleukin 4	Protein Coding	46	GC05P132673	38.40767	https://www.genecards.org/cgi-bin/carddisp.pl?gene=IL4
MAPK8	Mitogen-Activated Protein Kinase 8	Protein Coding	51	GC10P048306	38.08849	https://www.genecards.org/cgi-bin/carddisp.pl?gene=MAPK8
CDH1	Cadherin 1	Protein Coding	51	GC16P068737	38.05853	https://www.genecards.org/cgi-bin/carddisp.pl?gene=CDH1
LCAT	Lecithin-Cholesterol Acyltransferase	Protein Coding	47	GC16M067939	38.00154	https://www.genecards.org/cgi-bin/carddisp.pl?gene=LCAT
CYP1A2	Cytochrome P450 Family 1 Subfamily A Member 2	Protein Coding	46	GC15P074748	37.88988	https://www.genecards.org/cgi-bin/carddisp.pl?gene=CYP1A2

PTPN11	Protein Tyrosine Phosphatase Non-Receptor Type 11	Protein Coding	54	GC12P112418	37.85031	https://www.genecards.org/cgi-bin/carddisp.pl?gene=PTPN11
PYGM	Glycogen Phosphorylase, Muscle Associated	Protein Coding	47	GC11M064746	37.7818	https://www.genecards.org/cgi-bin/carddisp.pl?gene=PYGM
F9	Coagulation Factor IX	Protein Coding	47	GC0XP139530	37.73995	https://www.genecards.org/cgi-bin/carddisp.pl?gene=F9
HMGCR	3-Hydroxy-3-Methylglutaryl-CoA Reductase	Protein Coding	47	GC05P075336	37.69389	https://www.genecards.org/cgi-bin/carddisp.pl?gene=HMGCR
NFKB1	Nuclear Factor Kappa B Subunit 1	Protein Coding	52	GC04P102501	37.68575	https://www.genecards.org/cgi-bin/carddisp.pl?gene=NFKB1
PHYH	Phytanoyl-CoA 2-Hydroxylase	Protein Coding	46	GC10M013277	37.60526	https://www.genecards.org/cgi-bin/carddisp.pl?gene=PHYH
PNPLA2	Patatin Like Phospholipase Domain Containing 2	Protein Coding	45	GC11P000994	37.59045	https://www.genecards.org/cgi-bin/carddisp.pl?gene=PNPLA2
CYP27A1	Cytochrome P450 Family 27 Subfamily A Member 1	Protein Coding	48	GC02P218781	37.35265	https://www.genecards.org/cgi-bin/carddisp.pl?gene=CYP27A1
CDKN2A	Cyclin Dependent Kinase Inhibitor 2A	Protein Coding	52	GC09M021967	37.34846	https://www.genecards.org/cgi-bin/carddisp.pl?gene=CDKN2A
APOC3	Apolipoprotein C3	Protein Coding	44	GC11P116829	37.31222	https://www.genecards.org/cgi-bin/carddisp.pl?gene=APOC3
MUC1	Mucin 1, Cell Surface Associated	Protein Coding	47	GC01M155185	37.22253	https://www.genecards.org/cgi-bin/carddisp.pl?gene=MUC1
STAT1	Signal Transducer And Activator Of Transcription 1	Protein Coding	54	GC02M190908	37.14822	https://www.genecards.org/cgi-bin/carddisp.pl?gene=STAT1
COL2A1	Collagen Type II Alpha 1 Chain	Protein Coding	49	GC12M047972	37.08184	https://www.genecards.org/cgi-bin/carddisp.pl?gene=COL2A1
GSTP1	Glutathione S-Transferase Pi 1	Protein Coding	51	GC11P067583	36.93613	https://www.genecards.org/cgi-bin/carddisp.pl?gene=GSTP1
KIT	KIT Proto-Oncogene, Receptor Tyrosine Kinase	Protein Coding	53	GC04P054657	36.91124	https://www.genecards.org/cgi-bin/carddisp.pl?gene=KIT
ACACA	Acetyl-CoA Carboxylase Alpha	Protein Coding	50	GC17M037084	36.90222	https://www.genecards.org/cgi-bin/carddisp.pl?gene=ACACA

SLC6A3	Solute Carrier Family 6 Member 3	Protein Coding	50	GC05M001392	36.87577	https://www.genecards.org/cgi-bin/carddisp.pl?gene=SLC6A3
MIR222	MicroRNA 222	RNA Gene	21	GC0XM045747	36.82392	https://www.genecards.org/cgi-bin/carddisp.pl?gene=MIR222
GRIN2B	Glutamate Ionotropic Receptor NMDA Type Subunit 2B	Protein Coding	51	GC12M013437	36.73241	https://www.genecards.org/cgi-bin/carddisp.pl?gene=GRIN2B
PROM1	Prominin 1	Protein Coding	45	GC04M015965	36.68137	https://www.genecards.org/cgi-bin/carddisp.pl?gene=PROM1
MLXIPL	MLX Interacting Protein Like	Protein Coding	43	GC07M073593	36.64859	https://www.genecards.org/cgi-bin/carddisp.pl?gene=MLXIPL
SLC22A5	Solute Carrier Family 22 Member 5	Protein Coding	47	GC05P132369	36.60142	https://www.genecards.org/cgi-bin/carddisp.pl?gene=SLC22A5
BCS1L	BCS1 Homolog, Ubiquinol-Cytochrome C Reductase Complex Chaperone	Protein Coding	45	GC02P218658	36.50166	https://www.genecards.org/cgi-bin/carddisp.pl?gene=BCS1L
RELA	RELA Proto-Oncogene, NF-KB Subunit	Protein Coding	51	GC11M065653	36.49992	https://www.genecards.org/cgi-bin/carddisp.pl?gene=RELA
CYP2C9	Cytochrome P450 Family 2 Subfamily C Member 9	Protein Coding	50	GC10P094938	36.45664	https://www.genecards.org/cgi-bin/carddisp.pl?gene=CYP2C9
IRS2	Insulin Receptor Substrate 2	Protein Coding	45	GC13M109752	36.43042	https://www.genecards.org/cgi-bin/carddisp.pl?gene=IRS2
ALG8	ALG8 Alpha-1,3-Glucosyltransferase	Protein Coding	44	GC11M078100	36.41171	https://www.genecards.org/cgi-bin/carddisp.pl?gene=ALG8
MT-ATP6	Mitochondrially Encoded ATP Synthase Membrane Subunit 6	Protein Coding	33	GCMTP008531	36.40771	https://www.genecards.org/cgi-bin/carddisp.pl?gene=MT-ATP6
PLP1	Proteolipid Protein 1	Protein Coding	44	GC0XP103773	36.40392	https://www.genecards.org/cgi-bin/carddisp.pl?gene=PLP1
DNAJB11	DnaJ Heat Shock Protein Family (Hsp40) Member B11	Protein Coding	41	GC03P186567	36.38556	https://www.genecards.org/cgi-bin/carddisp.pl?gene=DNAJB11
REN	Renin	Protein Coding	48	GC01M204154	36.35252	https://www.genecards.org/cgi-bin/carddisp.pl?gene=REN
EHHADH	Enoyl-CoA Hydratase And 3-Hydroxyacyl CoA Dehydrogenase	Protein Coding	45	GC03M185190	36.2691	https://www.genecards.org/cgi-bin/carddisp.pl?gene=EHHADH

AGT	Angiotensinogen	Protein Coding	50	GC01M230702	36.25179	https://www.genecards.org/cgi-bin/carddisp.pl?gene=AGT
KIF1B	Kinesin Family Member 1B	Protein Coding	45	GC01P010210	36.22093	https://www.genecards.org/cgi-bin/carddisp.pl?gene=KIF1B
EDNRB	Endothelin Receptor Type B	Protein Coding	50	GC13M077895	36.21944	https://www.genecards.org/cgi-bin/carddisp.pl?gene=EDNRB
PEX19	Peroxisomal Biogenesis Factor 19	Protein Coding	43	GC01M160276	36.19999	https://www.genecards.org/cgi-bin/carddisp.pl?gene=PEX19
HRAS	HRas Proto-Oncogene, GTPase	Protein Coding	52	GC11M001078	36.19028	https://www.genecards.org/cgi-bin/carddisp.pl?gene=HRAS
CYP2D6	Cytochrome P450 Family 2 Subfamily D Member 6	Protein Coding	49	GC22M042126	36.18971	https://www.genecards.org/cgi-bin/carddisp.pl?gene=CYP2D6
CYP2C19	Cytochrome P450 Family 2 Subfamily C Member 19	Protein Coding	48	GC10P094762	36.17167	https://www.genecards.org/cgi-bin/carddisp.pl?gene=CYP2C19
HP	Haptoglobin	Protein Coding	45	GC16P072089	36.10358	https://www.genecards.org/cgi-bin/carddisp.pl?gene=HP
TTN	Titin	Protein Coding	48	GC02M178525	36.07557	https://www.genecards.org/cgi-bin/carddisp.pl?gene=TTN
MME	Membrane Metalloendopeptidase	Protein Coding	51	GC03P155024	35.78926	https://www.genecards.org/cgi-bin/carddisp.pl?gene=MME
SLC2A1	Solute Carrier Family 2 Member 1	Protein Coding	53	GC01M042925	35.76385	https://www.genecards.org/cgi-bin/carddisp.pl?gene=SLC2A1
SDHB	Succinate Dehydrogenase Complex Iron Sulfur Subunit B	Protein Coding	48	GC01M017238	35.74825	https://www.genecards.org/cgi-bin/carddisp.pl?gene=SDHB
PCSK9	Proprotein Convertase Subtilisin/Kexin Type 9	Protein Coding	51	GC01P055039	35.70944	https://www.genecards.org/cgi-bin/carddisp.pl?gene=PCSK9
TGFBR2	Transforming Growth Factor Beta Receptor 2	Protein Coding	51	GC03P030623	35.63598	https://www.genecards.org/cgi-bin/carddisp.pl?gene=TGFBR2
MYC	MYC Proto-Oncogene, BHLH Transcription Factor	Protein Coding	51	GC08P127735	35.60102	https://www.genecards.org/cgi-bin/carddisp.pl?gene=MYC
SBF2	SET Binding Factor 2	Protein Coding	40	GC11M009789	35.58323	https://www.genecards.org/cgi-bin/carddisp.pl?gene=SBF2
PEX16	Peroxisomal Biogenesis Factor 16	Protein Coding	39	GC11M068965	35.58163	https://www.genecards.org/cgi-bin/carddisp.pl?gene=PEX16
ANO5	Anoctamin 5	Protein Coding	38	GC11P021799	35.56927	https://www.genecards.org/cgi-bin/carddisp.pl?gene=ANO5

CETP	Cholesteryl Ester Transfer Protein	Protein Coding	47	GC16P056961	35.55682	https://www.genecards.org/cgi-bin/carddisp.pl?gene=CETP
ENG	Endoglin	Protein Coding	47	GC09M127815	35.49092	https://www.genecards.org/cgi-bin/carddisp.pl?gene=ENG
NCF2	Neutrophil Cytosolic Factor 2	Protein Coding	48	GC01M183555	35.40974	https://www.genecards.org/cgi-bin/carddisp.pl?gene=NCF2
ELOVL6	ELOVL Fatty Acid Elongase 6	Protein Coding	41	GC04M110045	35.40345	https://www.genecards.org/cgi-bin/carddisp.pl?gene=ELOVL6
TIMP1	TIMP Metallopeptidase Inhibitor 1	Protein Coding	45	GC0XP047583	35.38219	https://www.genecards.org/cgi-bin/carddisp.pl?gene=TIMP1
PRKAG2	Protein Kinase AMP-Activated Non-Catalytic Subunit Gamma 2	Protein Coding	51	GC07M151556	35.34998	https://www.genecards.org/cgi-bin/carddisp.pl?gene=PRKAG2
SIRT1	Sirtuin 1	Protein Coding	50	GC10P067884	35.17909	https://www.genecards.org/cgi-bin/carddisp.pl?gene=SIRT1
BCKDHB	Branched Chain Keto Acid Dehydrogenase E1 Subunit Beta	Protein Coding	44	GC06P080106	35.14046	https://www.genecards.org/cgi-bin/carddisp.pl?gene=BCKDHB
LAMP2	Lysosomal Associated Membrane Protein 2	Protein Coding	45	GC0XM120426	35.11869	https://www.genecards.org/cgi-bin/carddisp.pl?gene=LAMP2
JUN	Jun Proto-Oncogene, AP-1 Transcription Factor Subunit	Protein Coding	50	GC01M058780	35.06437	https://www.genecards.org/cgi-bin/carddisp.pl?gene=JUN
APBB1	Amyloid Beta Precursor Protein Binding Family B Member 1	Protein Coding	44	GC11M006396	35.02167	https://www.genecards.org/cgi-bin/carddisp.pl?gene=APBB1
ACADS	Acyl-CoA Dehydrogenase Short Chain	Protein Coding	47	GC12P120922	34.95808	https://www.genecards.org/cgi-bin/carddisp.pl?gene=ACADS
ABCG5	ATP Binding Cassette Subfamily G Member 5	Protein Coding	45	GC02M043806	34.94785	https://www.genecards.org/cgi-bin/carddisp.pl?gene=ABCG5
MIR93	MicroRNA 93	RNA Gene	20	GC07M100467	34.85299	https://www.genecards.org/cgi-bin/carddisp.pl?gene=MIR93
MMP2	Matrix Metallopeptidase 2	Protein Coding	54	GC16P055390	34.79349	https://www.genecards.org/cgi-bin/carddisp.pl?gene=MMP2
MIR181A1	MicroRNA 181a-1	RNA Gene	19	GC01M198860	34.78139	https://www.genecards.org/cgi-bin/carddisp.pl?gene=MIR181A1
UMOD	Uromodulin	Protein Coding	43	GC16M020344	34.75622	https://www.genecards.org/cgi-bin/carddisp.pl?gene=UMOD

NF1	Neurofibromin 1	Protein Coding	50	GC17P031094	34.75281	https://www.genecards.org/cgi-bin/carddisp.pl?gene=NF1
PLAU	Plasminogen Activator, Urokinase	Protein Coding	52	GC10P073909	34.50766	https://www.genecards.org/cgi-bin/carddisp.pl?gene=PLAU
MKS1	MKS Transition Zone Complex Subunit 1	Protein Coding	41	GC17M058205	34.48266	https://www.genecards.org/cgi-bin/carddisp.pl?gene=MKS1
NOS2	Nitric Oxide Synthase 2	Protein Coding	50	GC17M027756	34.44846	https://www.genecards.org/cgi-bin/carddisp.pl?gene=NOS2
FOS	Fos Proto-Oncogene, AP-1 Transcription Factor Subunit	Protein Coding	51	GC14P075278	34.42918	https://www.genecards.org/cgi-bin/carddisp.pl?gene=FOS
DGAT1	Diacylglycerol O-Acyltransferase 1	Protein Coding	47	GC08M144316	34.414	https://www.genecards.org/cgi-bin/carddisp.pl?gene=DGAT1
MIR132	MicroRNA 132	RNA Gene	21	GC17M002049	34.38493	https://www.genecards.org/cgi-bin/carddisp.pl?gene=MIR132
CCR6	C-C Motif Chemokine Receptor 6	Protein Coding	45	GC06P167111	34.2535	https://www.genecards.org/cgi-bin/carddisp.pl?gene=CCR6
SPINK1	Serine Peptidase Inhibitor Kazal Type 1	Protein Coding	44	GC05M147825	34.21467	https://www.genecards.org/cgi-bin/carddisp.pl?gene=SPINK1
SURF1	SURF1 Cytochrome C Oxidase Assembly Factor	Protein Coding	44	GC09M133351	34.18819	https://www.genecards.org/cgi-bin/carddisp.pl?gene=SURF1
CYCS	Cytochrome C, Somatic	Protein Coding	49	GC07M025118	34.18287	https://www.genecards.org/cgi-bin/carddisp.pl?gene=CYCS
EGR2	Early Growth Response 2	Protein Coding	44	GC10M062811	34.15501	https://www.genecards.org/cgi-bin/carddisp.pl?gene=EGR2
HLA-DQA1	Major Histocompatibility Complex, Class II, DQ Alpha 1	Protein Coding	43	GC06P055231	34.0709	https://www.genecards.org/cgi-bin/carddisp.pl?gene=HLA-DQA1
GATA4	GATA Binding Protein 4	Protein Coding	49	GC08P011676	34.07054	https://www.genecards.org/cgi-bin/carddisp.pl?gene=GATA4
AXIN1	Axin 1	Protein Coding	47	GC16M000287	33.70123	https://www.genecards.org/cgi-bin/carddisp.pl?gene=AXIN1
COL4A1	Collagen Type IV Alpha 1 Chain	Protein Coding	49	GC13M110148	33.69635	https://www.genecards.org/cgi-bin/carddisp.pl?gene=COL4A1
ADH6	Alcohol Dehydrogenase 6 (Class V)	Protein Coding	41	GC04M099202	33.63883	https://www.genecards.org/cgi-bin/carddisp.pl?gene=ADH6
NPHP3	Nephrocystin 3	Protein Coding	39	GC03M132683	33.61153	https://www.genecards.org/cgi-bin/carddisp.pl?gene=NPHP3
NDRG1	N-Myc Downstream Regulated 1	Protein Coding	45	GC08M133237	33.60076	https://www.genecards.org/cgi-bin/carddisp.pl?gene=NDRG1

APOA5	Apolipoprotein A5	Protein Coding	45	GC11M116789	33.56956	https://www.genecards.org/cgi-bin/carddisp.pl?gene=APOA5
CDKN3	Cyclin Dependent Kinase Inhibitor 3	Protein Coding	43	GC14P054398	33.56842	https://www.genecards.org/cgi-bin/carddisp.pl?gene=CDKN3
NR1H2	Nuclear Receptor Subfamily 1 Group H Member 2	Protein Coding	49	GC19P050329	33.50757	https://www.genecards.org/cgi-bin/carddisp.pl?gene=NR1H2
TNFRSF11A	TNF Receptor Superfamily Member 11a	Protein Coding	46	GC18P062325	33.47861	https://www.genecards.org/cgi-bin/carddisp.pl?gene=TNFRSF11A
ATM	ATM Serine/Threonine Kinase	Protein Coding	54	GC11P108222	33.46486	https://www.genecards.org/cgi-bin/carddisp.pl?gene=ATM
MIR127	MicroRNA 127	RNA Gene	20	GC14P105976	33.43264	https://www.genecards.org/cgi-bin/carddisp.pl?gene=MIR127
XBP1	X-Box Binding Protein 1	Protein Coding	46	GC22M028794	33.30799	https://www.genecards.org/cgi-bin/carddisp.pl?gene=XBP1
CASR	Calcium Sensing Receptor	Protein Coding	51	GC03P122183	33.3078	https://www.genecards.org/cgi-bin/carddisp.pl?gene=CASR
FGFR1	Fibroblast Growth Factor Receptor 1	Protein Coding	55	GC08M038400	33.30003	https://www.genecards.org/cgi-bin/carddisp.pl?gene=FGFR1
IL17A	Interleukin 17A	Protein Coding	43	GC06P052186	33.21131	https://www.genecards.org/cgi-bin/carddisp.pl?gene=IL17A
POMC	Proopiomelanocortin	Protein Coding	48	GC02M025160	33.12635	https://www.genecards.org/cgi-bin/carddisp.pl?gene=POMC
CTSD	Cathepsin D	Protein Coding	52	GC11M001752	33.0695	https://www.genecards.org/cgi-bin/carddisp.pl?gene=CTSD
FABP5	Fatty Acid Binding Protein 5	Protein Coding	42	GC08P081282	33.02143	https://www.genecards.org/cgi-bin/carddisp.pl?gene=FABP5
GATA3	GATA Binding Protein 3	Protein Coding	50	GC10P008045	33.00939	https://www.genecards.org/cgi-bin/carddisp.pl?gene=GATA3
TNFRSF11B	TNF Receptor Superfamily Member 11b	Protein Coding	48	GC08M118923	32.85939	https://www.genecards.org/cgi-bin/carddisp.pl?gene=TNFRSF11B
PEX14	Peroxisomal Biogenesis Factor 14	Protein Coding	44	GC01P010472	32.81486	https://www.genecards.org/cgi-bin/carddisp.pl?gene=PEX14
TWNK	Twinkle MtDNA Helicase	Protein Coding	34	GC10P100993	32.79261	https://www.genecards.org/cgi-bin/carddisp.pl?gene=TWNK
FABP12	Fatty Acid Binding Protein 12	Protein Coding	32	GC08M081524	32.77424	https://www.genecards.org/cgi-bin/carddisp.pl?gene=FABP12
GALC	Galactosylceramidase	Protein Coding	44	GC14M087837	32.75415	https://www.genecards.org/cgi-bin/carddisp.pl?gene=GALC
DBT	Dihydrolipoamide Branched Chain Transacylase E2	Protein Coding	43	GC01M100186	32.73995	https://www.genecards.org/cgi-bin/carddisp.pl?gene=DBT

MT-CO1	Mitochondrially Encoded Cytochrome C Oxidase I	Protein Coding	34	GCMTP005906	32.7396	https://www.genecards.org/cgi-bin/carddisp.pl?gene=MT-CO1
FOXP3	Forkhead Box P3	Protein Coding	47	GC0XM049250	32.71606	https://www.genecards.org/cgi-bin/carddisp.pl?gene=FOXP3
MIR195	MicroRNA 195	RNA Gene	19	GC17M007018	32.6982	https://www.genecards.org/cgi-bin/carddisp.pl?gene=MIR195
CES1	Carboxylesterase 1	Protein Coding	47	GC16M055836	32.63636	https://www.genecards.org/cgi-bin/carddisp.pl?gene=CES1
GHRL	Ghrelin And Obestatin Prepropeptide	Protein Coding	44	GC03M010285	32.62742	https://www.genecards.org/cgi-bin/carddisp.pl?gene=GHRL
MEFV	MEFV Innate Immuity Regulator, Pyrin	Protein Coding	44	GC16M003757	32.57311	https://www.genecards.org/cgi-bin/carddisp.pl?gene=MEFV
GCK	Glucokinase	Protein Coding	51	GC07M044154	32.55552	https://www.genecards.org/cgi-bin/carddisp.pl?gene=GCK
ABCG8	ATP Binding Cassette Subfamily G Member 8	Protein Coding	43	GC02P043828	32.51627	https://www.genecards.org/cgi-bin/carddisp.pl?gene=ABCG8
ADIPOR1	Adiponectin Receptor 1	Protein Coding	45	GC01M202940	32.46071	https://www.genecards.org/cgi-bin/carddisp.pl?gene=ADIPOR1
ACSL4	Acyl-CoA Synthetase Long Chain Family Member 4	Protein Coding	45	GC0XM109624	32.41319	https://www.genecards.org/cgi-bin/carddisp.pl?gene=ACSL4
AGTR1	Angiotensin II Receptor Type 1	Protein Coding	51	GC03P148697	32.31946	https://www.genecards.org/cgi-bin/carddisp.pl?gene=AGTR1
ASPA	Aspartoacylase	Protein Coding	45	GC17P003472	32.30996	https://www.genecards.org/cgi-bin/carddisp.pl?gene=ASPA
LITAF	Lipopolysaccharide Induced TNF Factor	Protein Coding	45	GC16M011547	32.28507	https://www.genecards.org/cgi-bin/carddisp.pl?gene=LITAF
SOX10	SRY-Box Transcription Factor 10	Protein Coding	46	GC22M049686	32.20561	https://www.genecards.org/cgi-bin/carddisp.pl?gene=SOX10
NRAS	NRAS Proto-Oncogene, GTPase	Protein Coding	50	GC01M114704	32.20041	https://www.genecards.org/cgi-bin/carddisp.pl?gene=NRAS
DLD	Dihydrolipoamide Dehydrogenase	Protein Coding	51	GC07P107890	32.18312	https://www.genecards.org/cgi-bin/carddisp.pl?gene=DLD
IFNA1	Interferon Alpha 1	Protein Coding	40	GC09P021522	32.13412	https://www.genecards.org/cgi-bin/carddisp.pl?gene=IFNA1
MIR18A	MicroRNA 18a	RNA Gene	18	GC13P091464	32.11747	https://www.genecards.org/cgi-bin/carddisp.pl?gene=MIR18A
EPO	Erythropoietin	Protein Coding	41	GC07P100720	32.09436	https://www.genecards.org/cgi-bin/carddisp.pl?gene=EPO
MIR29C	MicroRNA 29c	RNA Gene	18	GC01M207802	32.09364	https://www.genecards.org/cgi-bin/carddisp.pl?gene=MIR29C
VDR	Vitamin D Receptor	Protein Coding	52	GC12M047841	32.08536	https://www.genecards.org/cgi-bin/carddisp.pl?gene=VDR

MT-CYB	Mitochondrially Encoded Cytochrome B	Protein Coding	32	GCMTP014749	32.04627	https://www.genecards.org/cgi-bin/carddisp.pl?gene=MT-CYB
TARDBP	TAR DNA Binding Protein	Protein Coding	47	GC01P011013	32.02393	https://www.genecards.org/cgi-bin/carddisp.pl?gene=TARDBP
GNAS	GNAS Complex Locus	Protein Coding	51	GC20P058839	31.99583	https://www.genecards.org/cgi-bin/carddisp.pl?gene=GNAS
ADAM17	ADAM Metallopeptidase Domain 17	Protein Coding	51	GC02M009488	31.8755	https://www.genecards.org/cgi-bin/carddisp.pl?gene=ADAM17
IL2RA	Interleukin 2 Receptor Subunit Alpha	Protein Coding	51	GC10M006010	31.87153	https://www.genecards.org/cgi-bin/carddisp.pl?gene=IL2RA
SCN5A	Sodium Voltage-Gated Channel Alpha Subunit 5	Protein Coding	51	GC03M038549	31.8436	https://www.genecards.org/cgi-bin/carddisp.pl?gene=SCN5A
CD4	CD4 Molecule	Protein Coding	50	GC12P006786	31.80013	https://www.genecards.org/cgi-bin/carddisp.pl?gene=CD4
CYP2A6	Cytochrome P450 Family 2 Subfamily A Member 6	Protein Coding	48	GC19M040843	31.79175	https://www.genecards.org/cgi-bin/carddisp.pl?gene=CYP2A6
HEXB	Hexosaminidase Subunit Beta	Protein Coding	48	GC05P074640	31.7232	https://www.genecards.org/cgi-bin/carddisp.pl?gene=HEXB
ADIPOR2	Adiponectin Receptor 2	Protein Coding	43	GC12P001670	31.68838	https://www.genecards.org/cgi-bin/carddisp.pl?gene=ADIPOR2
DDIT3	DNA Damage Inducible Transcript 3	Protein Coding	46	GC12M057516	31.68555	https://www.genecards.org/cgi-bin/carddisp.pl?gene=DDIT3
SST	Somatostatin	Protein Coding	43	GC03M187668	31.66714	https://www.genecards.org/cgi-bin/carddisp.pl?gene=SST
MIR25	MicroRNA 25	RNA Gene	20	GC07M100093	31.64085	https://www.genecards.org/cgi-bin/carddisp.pl?gene=MIR25
WDR19	WD Repeat Domain 19	Protein Coding	38	GC04P039184	31.60878	https://www.genecards.org/cgi-bin/carddisp.pl?gene=WDR19
SLC40A1	Solute Carrier Family 40 Member 1	Protein Coding	47	GC02M189560	31.59604	https://www.genecards.org/cgi-bin/carddisp.pl?gene=SLC40A1
PIK3R1	Phosphoinositide-3-Kinase Regulatory Subunit 1	Protein Coding	51	GC05P068215	31.52237	https://www.genecards.org/cgi-bin/carddisp.pl?gene=PIK3R1
FAR2	Fatty Acyl-CoA Reductase 2	Protein Coding	40	GC12P029145	31.47194	https://www.genecards.org/cgi-bin/carddisp.pl?gene=FAR2
MIR145	MicroRNA 145	RNA Gene	22	GC05P149430	31.41011	https://www.genecards.org/cgi-bin/carddisp.pl?gene=MIR145
NCF1	Neutrophil Cytosolic Factor 1	Protein Coding	49	GC07P074773	31.40522	https://www.genecards.org/cgi-bin/carddisp.pl?gene=NCF1
MPV17	Mitochondrial Inner Membrane Protein MPV17	Protein Coding	42	GC02M027309	31.37327	https://www.genecards.org/cgi-bin/carddisp.pl?gene=MPV17
INF2	Inverted Formin 2	Protein Coding	41	GC14P106060	31.37173	https://www.genecards.org/cgi-bin/carddisp.pl?gene=INF2

MT-TL1	Mitochondrially Encoded TRNA-Leu (UUA/G) 1	RNA Gene	15	GCMTP003232	31.30286	https://www.genecards.org/cgi-bin/carddisp.pl?gene=MT-TL1
MIR140	MicroRNA 140	RNA Gene	22	GC16P069934	31.29485	https://www.genecards.org/cgi-bin/carddisp.pl?gene=MIR140
COL1A1	Collagen Type I Alpha 1 Chain	Protein Coding	51	GC17M050183	31.25345	https://www.genecards.org/cgi-bin/carddisp.pl?gene=COL1A1
ENPP1	Ectonucleotide Pyrophosphatase/Phosphodiesterase 1	Protein Coding	48	GC06P131808	31.24789	https://www.genecards.org/cgi-bin/carddisp.pl?gene=ENPP1
SLCO1B1	Solute Carrier Organic Anion Transporter Family Member 1B1	Protein Coding	48	GC12P021132	31.18969	https://www.genecards.org/cgi-bin/carddisp.pl?gene=SLCO1B1
MIR107	MicroRNA 107	RNA Gene	19	GC10M089600	31.17746	https://www.genecards.org/cgi-bin/carddisp.pl?gene=MIR107
MBL2	Mannose Binding Lectin 2	Protein Coding	48	GC10M052760	31.10712	https://www.genecards.org/cgi-bin/carddisp.pl?gene=MBL2
SCP2	Sterol Carrier Protein 2	Protein Coding	48	GC01P052927	31.07189	https://www.genecards.org/cgi-bin/carddisp.pl?gene=SCP2
NDUFS1	NADH:Ubiquinone Oxidoreductase Core Subunit S1	Protein Coding	47	GC02M206114	31.05576	https://www.genecards.org/cgi-bin/carddisp.pl?gene=NDUFS1
MIR210	MicroRNA 210	RNA Gene	21	GC11M001082	31.01629	https://www.genecards.org/cgi-bin/carddisp.pl?gene=MIR210
ALPL	Alkaline Phosphatase, Biomineralization Associated	Protein Coding	51	GC01P021508	30.99582	https://www.genecards.org/cgi-bin/carddisp.pl?gene=ALPL
SYNJ1	Synaptojanin 1	Protein Coding	46	GC21M032628	30.95378	https://www.genecards.org/cgi-bin/carddisp.pl?gene=SYNJ1
CLN3	CLN3 Lysosomal/Endosomal Transmembrane Protein, Battenin	Protein Coding	45	GC16M028466	30.94835	https://www.genecards.org/cgi-bin/carddisp.pl?gene=CLN3
CAV3	Caveolin 3	Protein Coding	44	GC03P008733	30.93867	https://www.genecards.org/cgi-bin/carddisp.pl?gene=CAV3
GUSB	Glucuronidase Beta	Protein Coding	48	GC07M065960	30.9182	https://www.genecards.org/cgi-bin/carddisp.pl?gene=GUSB
CD40LG	CD40 Ligand	Protein Coding	48	GC0XP136649	30.87214	https://www.genecards.org/cgi-bin/carddisp.pl?gene=CD40LG
PEMT	Phosphatidylethanolamine N-Methyltransferase	Protein Coding	41	GC17M017506	30.86824	https://www.genecards.org/cgi-bin/carddisp.pl?gene=PEMT

HSD3B7	Hydroxy-Delta-5-Steroid Dehydrogenase, 3 Beta- And Steroid Delta-Isomerase 7	Protein Coding	43	GC16P030985	30.8208	https://www.genecards.org/cgi-bin/carddisp.pl?gene=HSD3B7
TH	Tyrosine Hydroxylase	Protein Coding	52	GC11M002163	30.81452	https://www.genecards.org/cgi-bin/carddisp.pl?gene=TH
EDN3	Endothelin 3	Protein Coding	47	GC20P059300	30.8041	https://www.genecards.org/cgi-bin/carddisp.pl?gene=EDN3
SLC27A2	Solute Carrier Family 27 Member 2	Protein Coding	46	GC15P050182	30.7966	https://www.genecards.org/cgi-bin/carddisp.pl?gene=SLC27A2
MTMR2	Myotubularin Related Protein 2	Protein Coding	44	GC11M095822	30.78401	https://www.genecards.org/cgi-bin/carddisp.pl?gene=MTMR2
MIR181B1	MicroRNA 181b-1	RNA Gene	19	GC01M198858	30.75272	https://www.genecards.org/cgi-bin/carddisp.pl?gene=MIR181B1
GCKR	Glucokinase Regulator	Protein Coding	42	GC02P027496	30.66492	https://www.genecards.org/cgi-bin/carddisp.pl?gene=GCKR
IL12RB1	Interleukin 12 Receptor Subunit Beta 1	Protein Coding	45	GC19M018058	30.66179	https://www.genecards.org/cgi-bin/carddisp.pl?gene=IL12RB1
SMAD3	SMAD Family Member 3	Protein Coding	50	GC15P067063	30.63283	https://www.genecards.org/cgi-bin/carddisp.pl?gene=SMAD3
SDHD	Succinate Dehydrogenase Complex Subunit D	Protein Coding	45	GC11P112087	30.61225	https://www.genecards.org/cgi-bin/carddisp.pl?gene=SDHD
HSPB8	Heat Shock Protein Family B (Small) Member 8	Protein Coding	45	GC12P119173	30.57958	https://www.genecards.org/cgi-bin/carddisp.pl?gene=HSPB8
MIR23B	MicroRNA 23b	RNA Gene	19	GC09P095085	30.56204	https://www.genecards.org/cgi-bin/carddisp.pl?gene=MIR23B
TFR2	Transferrin Receptor 2	Protein Coding	45	GC07M100620	30.52137	https://www.genecards.org/cgi-bin/carddisp.pl?gene=TFR2
SERPINC1	Serpin Family C Member 1	Protein Coding	49	GC01M174251	30.48973	https://www.genecards.org/cgi-bin/carddisp.pl?gene=SERPINC1
AKT2	AKT Serine/Threonine Kinase 2	Protein Coding	54	GC19M040230	30.47733	https://www.genecards.org/cgi-bin/carddisp.pl?gene=AKT2
MAOA	Monoamine Oxidase A	Protein Coding	51	GC0XP043654	30.41586	https://www.genecards.org/cgi-bin/carddisp.pl?gene=MAOA
TJP2	Tight Junction Protein 2	Protein Coding	47	GC09P069121	30.40238	https://www.genecards.org/cgi-bin/carddisp.pl?gene=TJP2
PMM2	Phosphomannomutase 2	Protein Coding	47	GC16P008788	30.3838	https://www.genecards.org/cgi-bin/carddisp.pl?gene=PMM2
CHAT	Choline O-Acetyltransferase	Protein Coding	48	GC10P049609	30.38092	https://www.genecards.org/cgi-bin/carddisp.pl?gene=CHAT
GYS1	Glycogen Synthase 1	Protein Coding	50	GC19M048970	30.36652	https://www.genecards.org/cgi-bin/carddisp.pl?gene=GYS1
AHSG	Alpha 2-HS Glycoprotein	Protein Coding	44	GC03P186612	30.36555	https://www.genecards.org/cgi-bin/carddisp.pl?gene=AHSG
PRKCD	Protein Kinase C Delta	Protein Coding	54	GC03P053156	30.35056	https://www.genecards.org/cgi-bin/carddisp.pl?gene=PRKCD

AKR1D1	Aldo-Keto Reductase Family 1 Member D1	Protein Coding	47	GC07P138003	30.34717	https://www.genecards.org/cgi-bin/carddisp.pl?gene=AKR1D1
MSH2	MutS Homolog 2	Protein Coding	49	GC02P047402	30.34285	https://www.genecards.org/cgi-bin/carddisp.pl?gene=MSH2
GFER	Growth Factor, Augmenter Of Liver Regeneration	Protein Coding	45	GC16P001984	30.30947	https://www.genecards.org/cgi-bin/carddisp.pl?gene=GFER
GCG	Glucagon	Protein Coding	41	GC02M162142	30.19323	https://www.genecards.org/cgi-bin/carddisp.pl?gene=GCG
ATP7A	ATPase Copper Transporting Alpha	Protein Coding	47	GC0XP077943	30.17986	https://www.genecards.org/cgi-bin/carddisp.pl?gene=ATP7A
RPGRIP1L	RPGRIP1 Like	Protein Coding	39	GC16M053597	30.1636	https://www.genecards.org/cgi-bin/carddisp.pl?gene=RPGRIP1L
ADA	Adenosine Deaminase	Protein Coding	52	GC20M044620	30.14722	https://www.genecards.org/cgi-bin/carddisp.pl?gene=ADA
PHKB	Phosphorylase Kinase Regulatory Subunit Beta	Protein Coding	46	GC16P047436	30.13322	https://www.genecards.org/cgi-bin/carddisp.pl?gene=PHKB
OFD1	OFD1 Centriole And Centriolar Satellite Protein	Protein Coding	43	GC0XP013734	30.11156	https://www.genecards.org/cgi-bin/carddisp.pl?gene=OFD1
ETFDH	Electron Transfer Flavoprotein Dehydrogenase	Protein Coding	45	GC04P158672	30.08014	https://www.genecards.org/cgi-bin/carddisp.pl?gene=ETFDH
CCR5	C-C Motif Chemokine Receptor 5	Protein Coding	47	GC03P046383	30.06822	https://www.genecards.org/cgi-bin/carddisp.pl?gene=CCR5
AMACR	Alpha-Methylacyl-CoA Racemase	Protein Coding	46	GC05M033986	30.05449	https://www.genecards.org/cgi-bin/carddisp.pl?gene=AMACR
MIR196A2	MicroRNA 196a-2	RNA Gene	21	GC12P054368	30.04873	https://www.genecards.org/cgi-bin/carddisp.pl?gene=MIR196A2
LIPE	Lipase E, Hormone Sensitive Type	Protein Coding	48	GC19M042401	30.04714	https://www.genecards.org/cgi-bin/carddisp.pl?gene=LIPE
IFT172	Intraflagellar Transport 172	Protein Coding	40	GC02M027537	30.01382	https://www.genecards.org/cgi-bin/carddisp.pl?gene=IFT172
MIR31	MicroRNA 31	RNA Gene	20	GC09M021513	29.99636	https://www.genecards.org/cgi-bin/carddisp.pl?gene=MIR31
NPY	Neuropeptide Y	Protein Coding	46	GC07P024290	29.94777	https://www.genecards.org/cgi-bin/carddisp.pl?gene=NPY
MIR23A	MicroRNA 23a	RNA Gene	20	GC19M014110	29.90722	https://www.genecards.org/cgi-bin/carddisp.pl?gene=MIR23A
NPHP1	Nephrocystin 1	Protein Coding	44	GC02M110122	29.85468	https://www.genecards.org/cgi-bin/carddisp.pl?gene=NPHP1
ARSA	Arylsulfatase A	Protein Coding	47	GC22M050622	29.83828	https://www.genecards.org/cgi-bin/carddisp.pl?gene=ARSA

MAP2K1	Mitogen-Activated Protein Kinase Kinase 1	Protein Coding	54	GC15P066386	29.82568	https://www.genecards.org/cgi-bin/carddisp.pl?gene=MAP2K1
ADAMTS13	ADAM Metallopeptidase With Thrombospondin Type 1 Motif 13	Protein Coding	47	GC09P133414	29.77298	https://www.genecards.org/cgi-bin/carddisp.pl?gene=ADAMTS13
MT-ND5	Mitochondrially Encoded NADH:Ubiquinone Oxidoreductase Core Subunit 5	Protein Coding	33	GCMTP012339	29.76902	https://www.genecards.org/cgi-bin/carddisp.pl?gene=MT-ND5
FGFR2	Fibroblast Growth Factor Receptor 2	Protein Coding	54	GC10M121478	29.76437	https://www.genecards.org/cgi-bin/carddisp.pl?gene=FGFR2
PRKAR1A	Protein Kinase CAMP-Dependent Type I Regulatory Subunit Alpha	Protein Coding	52	GC17P068545	29.75539	https://www.genecards.org/cgi-bin/carddisp.pl?gene=PRKAR1A
HIF1A	Hypoxia Inducible Factor 1 Subunit Alpha	Protein Coding	48	GC14P061695	29.73709	https://www.genecards.org/cgi-bin/carddisp.pl?gene=HIF1A
CNR1	Cannabinoid Receptor 1	Protein Coding	47	GC06M088139	29.72316	https://www.genecards.org/cgi-bin/carddisp.pl?gene=CNR1
PLIN2	Perilipin 2	Protein Coding	44	GC09M019127	29.71279	https://www.genecards.org/cgi-bin/carddisp.pl?gene=PLIN2
GM2A	GM2 Ganglioside Activator	Protein Coding	43	GC05P151229	29.71018	https://www.genecards.org/cgi-bin/carddisp.pl?gene=GM2A
INPP5E	Inositol Polyphosphate-5-Phosphatase E	Protein Coding	42	GC09M136428	29.64631	https://www.genecards.org/cgi-bin/carddisp.pl?gene=INPP5E
JUP	Junction Plakoglobin	Protein Coding	48	GC17M041754	29.63376	https://www.genecards.org/cgi-bin/carddisp.pl?gene=JUP
XIAP	X-Linked Inhibitor Of Apoptosis	Protein Coding	50	GC0XP123859	29.61671	https://www.genecards.org/cgi-bin/carddisp.pl?gene=XIAP
IL13	Interleukin 13	Protein Coding	45	GC05P132656	29.61028	https://www.genecards.org/cgi-bin/carddisp.pl?gene=IL13
ERCC6	ERCC Excision Repair 6, Chromatin Remodeling Factor	Protein Coding	46	GC10M049454	29.59432	https://www.genecards.org/cgi-bin/carddisp.pl?gene=ERCC6
ABCB7	ATP Binding Cassette Subfamily B Member 7	Protein Coding	44	GC0XM075053	29.55732	https://www.genecards.org/cgi-bin/carddisp.pl?gene=ABCB7

CEBPA	CCAAT Enhancer Binding Protein Alpha	Protein Coding	48	GC19M033299	29.52411	https://www.genecards.org/cgi-bin/carddisp.pl?gene=CEBPA
SLC2A4	Solute Carrier Family 2 Member 4	Protein Coding	47	GC17P009071	29.49243	https://www.genecards.org/cgi-bin/carddisp.pl?gene=SLC2A4
MYH7	Myosin Heavy Chain 7	Protein Coding	48	GC14M023412	29.45399	https://www.genecards.org/cgi-bin/carddisp.pl?gene=MYH7
HNRNPA1	Heterogeneous Nuclear Ribonucleoprotein A1	Protein Coding	47	GC12P054280	29.43881	https://www.genecards.org/cgi-bin/carddisp.pl?gene=HNRNPA1
FGD4	FYVE, RhoGEF And PH Domain Containing 4	Protein Coding	43	GC12P032407	29.41738	https://www.genecards.org/cgi-bin/carddisp.pl?gene=FGD4
NLRP3	NLR Family Pyrin Domain Containing 3	Protein Coding	48	GC01P247415	29.38379	https://www.genecards.org/cgi-bin/carddisp.pl?gene=NLRP3
SLC27A1	Solute Carrier Family 27 Member 1	Protein Coding	41	GC19P026639	29.37304	https://www.genecards.org/cgi-bin/carddisp.pl?gene=SLC27A1
GRN	Granulin Precursor	Protein Coding	47	GC17P044345	29.34554	https://www.genecards.org/cgi-bin/carddisp.pl?gene=GRN
EDN1	Endothelin 1	Protein Coding	48	GC06P012256	29.34463	https://www.genecards.org/cgi-bin/carddisp.pl?gene=EDN1
NOTCH2	Notch Receptor 2	Protein Coding	50	GC01M119911	29.34434	https://www.genecards.org/cgi-bin/carddisp.pl?gene=NOTCH2
GAPDH	Glyceraldehyde-3-Phosphate Dehydrogenase	Protein Coding	48	GC12P011841	29.33832	https://www.genecards.org/cgi-bin/carddisp.pl?gene=GAPDH
F3	Coagulation Factor III, Tissue Factor	Protein Coding	45	GC01M094530	29.33734	https://www.genecards.org/cgi-bin/carddisp.pl?gene=F3
C3	Complement C3	Protein Coding	48	GC19M006677	29.31971	https://www.genecards.org/cgi-bin/carddisp.pl?gene=C3
BMP6	Bone Morphogenetic Protein 6	Protein Coding	44	GC06P007726	29.30725	https://www.genecards.org/cgi-bin/carddisp.pl?gene=BMP6
SLC2A2	Solute Carrier Family 2 Member 2	Protein Coding	50	GC03M170996	29.28117	https://www.genecards.org/cgi-bin/carddisp.pl?gene=SLC2A2
G6PD	Glucose-6-Phosphate Dehydrogenase	Protein Coding	51	GC0XM154531	29.21536	https://www.genecards.org/cgi-bin/carddisp.pl?gene=G6PD
HSPD1	Heat Shock Protein Family D (Hsp60) Member 1	Protein Coding	48	GC02M197486	29.21293	https://www.genecards.org/cgi-bin/carddisp.pl?gene=HSPD1

ALMS1	ALMS1 Centrosome And Basal Body Associated Protein	Protein Coding	42	GC02P073385	29.20957	https://www.genecards.org/cgi-bin/carddisp.pl?gene=ALMS1
ARG1	Arginase 1	Protein Coding	50	GC06P131473	29.12553	https://www.genecards.org/cgi-bin/carddisp.pl?gene=ARG1
SLC27A4	Solute Carrier Family 27 Member 4	Protein Coding	47	GC09P128340	29.11403	https://www.genecards.org/cgi-bin/carddisp.pl?gene=SLC27A4
IL23R	Interleukin 23 Receptor	Protein Coding	45	GC01P067138	29.03369	https://www.genecards.org/cgi-bin/carddisp.pl?gene=IL23R
ELANE	Elastase, Neutrophil Expressed	Protein Coding	47	GC19P000855	29.01259	https://www.genecards.org/cgi-bin/carddisp.pl?gene=ELANE
LBR	Lamin B Receptor	Protein Coding	48	GC01M225401	28.98354	https://www.genecards.org/cgi-bin/carddisp.pl?gene=LBR
IGF1R	Insulin Like Growth Factor 1 Receptor	Protein Coding	55	GC15P098648	28.96224	https://www.genecards.org/cgi-bin/carddisp.pl?gene=IGF1R
RLBP1	Retinaldehyde Binding Protein 1	Protein Coding	45	GC15M089209	28.95952	https://www.genecards.org/cgi-bin/carddisp.pl?gene=RLBP1
ASS1	Argininosuccinate Synthase 1	Protein Coding	49	GC09P130444	28.95013	https://www.genecards.org/cgi-bin/carddisp.pl?gene=ASS1
MIR191	MicroRNA 191	RNA Gene	20	GC03M049438	28.94748	https://www.genecards.org/cgi-bin/carddisp.pl?gene=MIR191
ALDH9A1	Aldehyde Dehydrogenase 9 Family Member A1	Protein Coding	42	GC01M165670	28.94373	https://www.genecards.org/cgi-bin/carddisp.pl?gene=ALDH9A1
THBD	Thrombomodulin	Protein Coding	44	GC20M023026	28.87011	https://www.genecards.org/cgi-bin/carddisp.pl?gene=THBD
AHI1	Abelson Helper Integration Site 1	Protein Coding	42	GC06M135283	28.86435	https://www.genecards.org/cgi-bin/carddisp.pl?gene=AHI1
B2M	Beta-2-Microglobulin	Protein Coding	49	GC15P044711	28.82696	https://www.genecards.org/cgi-bin/carddisp.pl?gene=B2M
ELOVL5	ELOVL Fatty Acid Elongase 5	Protein Coding	44	GC06M053240	28.80394	https://www.genecards.org/cgi-bin/carddisp.pl?gene=ELOVL5
WT1	WT1 Transcription Factor	Protein Coding	50	GC11M032365	28.78292	https://www.genecards.org/cgi-bin/carddisp.pl?gene=WT1
PEX11B	Peroxisomal Biogenesis Factor 11 Beta	Protein Coding	41	GC01M145911	28.73597	https://www.genecards.org/cgi-bin/carddisp.pl?gene=PEX11B
IL6R	Interleukin 6 Receptor	Protein Coding	50	GC01P154405	28.71971	https://www.genecards.org/cgi-bin/carddisp.pl?gene=IL6R
ACP5	Acid Phosphatase 5, Tartrate Resistant	Protein Coding	47	GC19M011574	28.65755	https://www.genecards.org/cgi-bin/carddisp.pl?gene=ACP5
MECP2	Methyl-CpG Binding Protein 2	Protein Coding	46	GC0XM154021	28.62125	https://www.genecards.org/cgi-bin/carddisp.pl?gene=MECP2

MT-ND2	Mitochondrially Encoded NADH:Ubiquinone Oxidoreductase Core Subunit 2	Protein Coding	34	GCMTP004472	28.61034	https://www.genecards.org/cgi-bin/carddisp.pl?gene=MT-ND2
BAX	BCL2 Associated X, Apoptosis Regulator	Protein Coding	49	GC19P048954	28.60721	https://www.genecards.org/cgi-bin/carddisp.pl?gene=BAX
DSP	Desmoplakin	Protein Coding	50	GC06P007541	28.60315	https://www.genecards.org/cgi-bin/carddisp.pl?gene=DSP
ABCC8	ATP Binding Cassette Subfamily C Member 8	Protein Coding	47	GC11M017392	28.55995	https://www.genecards.org/cgi-bin/carddisp.pl?gene=ABCC8
NCF4	Neutrophil Cytosolic Factor 4	Protein Coding	49	GC22P036860	28.54453	https://www.genecards.org/cgi-bin/carddisp.pl?gene=NCF4
MIR181A2	MicroRNA 181a-2	RNA Gene	20	GC09P124692	28.54213	https://www.genecards.org/cgi-bin/carddisp.pl?gene=MIR181A2
MIR200A	MicroRNA 200a	RNA Gene	22	GC01P002022	28.52721	https://www.genecards.org/cgi-bin/carddisp.pl?gene=MIR200A
CREB1	CAMP Responsive Element Binding Protein 1	Protein Coding	50	GC02P207529	28.51627	https://www.genecards.org/cgi-bin/carddisp.pl?gene=CREB1
UGT1A7	UDP Glucuronosyltransferase Family 1 Member A7	Protein Coding	39	GC02P233681	28.5161	https://www.genecards.org/cgi-bin/carddisp.pl?gene=UGT1A7
SCARB2	Scavenger Receptor Class B Member 2	Protein Coding	44	GC04M076158	28.49587	https://www.genecards.org/cgi-bin/carddisp.pl?gene=SCARB2
MIR224	MicroRNA 224	RNA Gene	17	GC0XM151958	28.47194	https://www.genecards.org/cgi-bin/carddisp.pl?gene=MIR224
ACTA2	Actin Alpha 2, Smooth Muscle	Protein Coding	49	GC10M088935	28.45015	https://www.genecards.org/cgi-bin/carddisp.pl?gene=ACTA2
LARS1	Leucyl-TRNA Synthetase 1	Protein Coding	37	GC05M146114	28.44983	https://www.genecards.org/cgi-bin/carddisp.pl?gene=LARS1
TGFB2	Transforming Growth Factor Beta 2	Protein Coding	50	GC01P218345	28.41203	https://www.genecards.org/cgi-bin/carddisp.pl?gene=TGFB2
LPA	Lipoprotein(A)	Protein Coding	42	GC06M160531	28.40335	https://www.genecards.org/cgi-bin/carddisp.pl?gene=LPA
MIR199A1	MicroRNA 199a-1	RNA Gene	19	GC19M010817	28.39847	https://www.genecards.org/cgi-bin/carddisp.pl?gene=MIR199A1
H2AC18	H2A Clustered Histone 18	Protein Coding	26	GC01M150124	28.3431	https://www.genecards.org/cgi-bin/carddisp.pl?gene=H2AC18
VIM	Vimentin	Protein Coding	51	GC10P017227	28.3147	https://www.genecards.org/cgi-bin/carddisp.pl?gene=VIM
MMP3	Matrix Metallopeptidase 3	Protein Coding	51	GC11M102835	28.29143	https://www.genecards.org/cgi-bin/carddisp.pl?gene=MMP3

COX5A	Cytochrome C Oxidase Subunit 5A	Protein Coding	45	GC15M074919	28.26572	https://www.genecards.org/cgi-bin/carddisp.pl?gene=COX5A
GJA1	Gap Junction Protein Alpha 1	Protein Coding	51	GC06P121436	28.25968	https://www.genecards.org/cgi-bin/carddisp.pl?gene=GJA1
ELN	Elastin	Protein Coding	45	GC07P074027	28.20924	https://www.genecards.org/cgi-bin/carddisp.pl?gene=ELN
GRIN2A	Glutamate Ionotropic Receptor NMDA Type Subunit 2A	Protein Coding	52	GC16M009753	28.19079	https://www.genecards.org/cgi-bin/carddisp.pl?gene=GRIN2A
TM6SF2	Transmembrane 6 Superfamily Member 2	Protein Coding	29	GC19M019264	28.19012	https://www.genecards.org/cgi-bin/carddisp.pl?gene=TM6SF2
FCGR2A	Fc Fragment Of IgG Receptor IIa	Protein Coding	46	GC01P161505	28.18043	https://www.genecards.org/cgi-bin/carddisp.pl?gene=FCGR2A
FN1	Fibronectin 1	Protein Coding	50	GC02M215360	28.16509	https://www.genecards.org/cgi-bin/carddisp.pl?gene=FN1
RRM2B	Ribonucleotide Reductase Regulatory TP53 Inducible Subunit M2B	Protein Coding	50	GC08M102204	28.14182	https://www.genecards.org/cgi-bin/carddisp.pl?gene=RRM2B
IDUA	Alpha-L-Iduronidase	Protein Coding	43	GC04P000986	28.08902	https://www.genecards.org/cgi-bin/carddisp.pl?gene=IDUA
TBK1	TANK Binding Kinase 1	Protein Coding	50	GC12P064451	28.04251	https://www.genecards.org/cgi-bin/carddisp.pl?gene=TBK1
CST3	Cystatin C	Protein Coding	45	GC20M023627	28.04056	https://www.genecards.org/cgi-bin/carddisp.pl?gene=CST3
DMD	Dystrophin	Protein Coding	47	GC0XM031097	27.96534	https://www.genecards.org/cgi-bin/carddisp.pl?gene=DMD
HJV	Hemojuvelin BMP Co-Receptor	Protein Coding	36	GC01M146018	27.94221	https://www.genecards.org/cgi-bin/carddisp.pl?gene=HJV
MT-CO3	Mitochondrially Encoded Cytochrome C Oxidase III	Protein Coding	31	GCMTP009209	27.86255	https://www.genecards.org/cgi-bin/carddisp.pl?gene=MT-CO3
SMARCA4	SWI/SNF Related, Matrix Associated, Actin Dependent Regulator Of Chromatin, Subfamily A, Member 4	Protein Coding	50	GC19P010932	27.85229	https://www.genecards.org/cgi-bin/carddisp.pl?gene=SMARCA4
CREBBP	CREB Binding Protein	Protein Coding	52	GC16M003745	27.79096	https://www.genecards.org/cgi-bin/carddisp.pl?gene=CREBBP
NR4A2	Nuclear Receptor Subfamily 4 Group A Member 2	Protein Coding	48	GC02M156324	27.7746	https://www.genecards.org/cgi-bin/carddisp.pl?gene=NR4A2

PRKAB1	Protein Kinase AMP-Activated Non-Catalytic Subunit Beta 1	Protein Coding	48	GC12P119632	27.74568	https://www.genecards.org/cgi-bin/carddisp.pl?gene=PRKAB1
CYP7A1	Cytochrome P450 Family 7 Subfamily A Member 1	Protein Coding	44	GC08M058476	27.70166	https://www.genecards.org/cgi-bin/carddisp.pl?gene=CYP7A1
NDUFS4	NADH:Ubiquinone Oxidoreductase Subunit S4	Protein Coding	45	GC05P053560	27.68033	https://www.genecards.org/cgi-bin/carddisp.pl?gene=NDUFS4
ABCD3	ATP Binding Cassette Subfamily D Member 3	Protein Coding	45	GC01P094418	27.6318	https://www.genecards.org/cgi-bin/carddisp.pl?gene=ABCD3
MAP3K5	Mitogen-Activated Protein Kinase Kinase Kinase 5	Protein Coding	48	GC06M136557	27.62798	https://www.genecards.org/cgi-bin/carddisp.pl?gene=MAP3K5
ETFA	Electron Transfer Flavoprotein Subunit Alpha	Protein Coding	46	GC15M076215	27.62755	https://www.genecards.org/cgi-bin/carddisp.pl?gene=ETFA
HNRNPA2B1	Heterogeneous Nuclear Ribonucleoprotein A2/B1	Protein Coding	47	GC07M026174	27.6245	https://www.genecards.org/cgi-bin/carddisp.pl?gene=HNRNPA2B1
ACACB	Acetyl-CoA Carboxylase Beta	Protein Coding	47	GC12P109116	27.58549	https://www.genecards.org/cgi-bin/carddisp.pl?gene=ACACB
TNFRSF1B	TNF Receptor Superfamily Member 1B	Protein Coding	48	GC01P012167	27.55905	https://www.genecards.org/cgi-bin/carddisp.pl?gene=TNFRSF1B
PDGFRL	Platelet Derived Growth Factor Receptor Like	Protein Coding	43	GC08P017576	27.55731	https://www.genecards.org/cgi-bin/carddisp.pl?gene=PDGFRL
BCHE	Butyrylcholinesterase	Protein Coding	49	GC03M165772	27.54298	https://www.genecards.org/cgi-bin/carddisp.pl?gene=BCHE
ACTB	Actin Beta	Protein Coding	50	GC07M005527	27.54268	https://www.genecards.org/cgi-bin/carddisp.pl?gene=ACTB
DGUOK	Deoxyguanosine Kinase	Protein Coding	45	GC02P073926	27.45197	https://www.genecards.org/cgi-bin/carddisp.pl?gene=DGUOK
ATXN3	Ataxin 3	Protein Coding	45	GC14M095796	27.44978	https://www.genecards.org/cgi-bin/carddisp.pl?gene=ATXN3
C9orf72	C9orf72-SMCR8 Complex Subunit	Protein Coding	42	GC09M027588	27.4369	https://www.genecards.org/cgi-bin/carddisp.pl?gene=C9orf72
CD14	CD14 Molecule	Protein Coding	45	GC05M140631	27.43073	https://www.genecards.org/cgi-bin/carddisp.pl?gene=CD14
BMP2	Bone Morphogenetic Protein 2	Protein Coding	47	GC20P006696	27.42911	https://www.genecards.org/cgi-bin/carddisp.pl?gene=BMP2

BEST1	Bestrophin 1	Protein Coding	44	GC11P061949	27.41586	https://www.genecards.org/cgi-bin/carddisp.pl?gene=BEST1
GSK3B	Glycogen Synthase Kinase 3 Beta	Protein Coding	51	GC03M119821	27.39133	https://www.genecards.org/cgi-bin/carddisp.pl?gene=GSK3B
CCL5	C-C Motif Chemokine Ligand 5	Protein Coding	44	GC17M035871	27.36868	https://www.genecards.org/cgi-bin/carddisp.pl?gene=CCL5
BCL2	BCL2 Apoptosis Regulator	Protein Coding	51	GC18M063123	27.29396	https://www.genecards.org/cgi-bin/carddisp.pl?gene=BCL2
NAT2	N-Acetyltransferase 2	Protein Coding	44	GC08P018391	27.22961	https://www.genecards.org/cgi-bin/carddisp.pl?gene=NAT2
PEX7	Peroxisomal Biogenesis Factor 7	Protein Coding	44	GC06P136822	27.22859	https://www.genecards.org/cgi-bin/carddisp.pl?gene=PEX7
PCK2	Phosphoenolpyruvate Carboxykinase 2, Mitochondrial	Protein Coding	48	GC14P024094	27.22156	https://www.genecards.org/cgi-bin/carddisp.pl?gene=PCK2
HARS1	Histidyl-TRNA Synthetase 1	Protein Coding	37	GC05M140673	27.17268	https://www.genecards.org/cgi-bin/carddisp.pl?gene=HARS1
ALDOB	Aldolase, Fructose-Bisphosphate B	Protein Coding	46	GC09M101420	27.15658	https://www.genecards.org/cgi-bin/carddisp.pl?gene=ALDOB
SELE	Selectin E	Protein Coding	44	GC01M169722	27.12234	https://www.genecards.org/cgi-bin/carddisp.pl?gene=SELE
MIR30A	MicroRNA 30a	RNA Gene	20	GC06M071403	27.09053	https://www.genecards.org/cgi-bin/carddisp.pl?gene=MIR30A
FGFR3	Fibroblast Growth Factor Receptor 3	Protein Coding	55	GC04P001795	27.07513	https://www.genecards.org/cgi-bin/carddisp.pl?gene=FGFR3
SLC19A3	Solute Carrier Family 19 Member 3	Protein Coding	47	GC02M227685	27.04611	https://www.genecards.org/cgi-bin/carddisp.pl?gene=SLC19A3
RAF1	Raf-1 Proto-Oncogene, Serine/Threonine Kinase	Protein Coding	54	GC03M012583	27.03859	https://www.genecards.org/cgi-bin/carddisp.pl?gene=RAF1
GLB1	Galactosidase Beta 1	Protein Coding	48	GC03M032963	27.02466	https://www.genecards.org/cgi-bin/carddisp.pl?gene=GLB1
NKX2-5	NK2 Homeobox 5	Protein Coding	45	GC05M173232	27.02188	https://www.genecards.org/cgi-bin/carddisp.pl?gene=NKX2-5
GSTT1	Glutathione S-Transferase Theta 1	Protein Coding	33	GC22Mi00270	27.01846	https://www.genecards.org/cgi-bin/carddisp.pl?gene=GSTT1
MSH6	MutS Homolog 6	Protein Coding	51	GC02P047695	27.00141	https://www.genecards.org/cgi-bin/carddisp.pl?gene=MSH6
KARS1	Lysyl-TRNA Synthetase 1	Protein Coding	38	GC16M075628	26.97801	https://www.genecards.org/cgi-bin/carddisp.pl?gene=KARS1
CXCL12	C-X-C Motif Chemokine Ligand 12	Protein Coding	46	GC10M044294	26.92284	https://www.genecards.org/cgi-bin/carddisp.pl?gene=CXCL12
NPPB	Natriuretic Peptide B	Protein Coding	44	GC01M011858	26.91472	https://www.genecards.org/cgi-bin/carddisp.pl?gene=NPPB

PRKAA1	Protein Kinase AMP-Activated Catalytic Subunit Alpha 1	Protein Coding	48	GC05M040759	26.90591	https://www.genecards.org/cgi-bin/carddisp.pl?gene=PRKAA1
ATP2A2	ATPase Sarcoplasmic/Endoplasmic Reticulum Ca2+ Transporting 2	Protein Coding	52	GC12P110280	26.90004	https://www.genecards.org/cgi-bin/carddisp.pl?gene=ATP2A2
OPRM1	Opioid Receptor Mu 1	Protein Coding	49	GC06P154075	26.89049	https://www.genecards.org/cgi-bin/carddisp.pl?gene=OPRM1
KCNQ1	Potassium Voltage-Gated Channel Subfamily Q Member 1	Protein Coding	50	GC11P002444	26.87739	https://www.genecards.org/cgi-bin/carddisp.pl?gene=KCNQ1
MIR125A	MicroRNA 125a	RNA Gene	21	GC19P051720	26.86593	https://www.genecards.org/cgi-bin/carddisp.pl?gene=MIR125A
ALPP	Alkaline Phosphatase, Placental	Protein Coding	47	GC02P232378	26.85051	https://www.genecards.org/cgi-bin/carddisp.pl?gene=ALPP
FADS3	Fatty Acid Desaturase 3	Protein Coding	37	GC11M061873	26.83002	https://www.genecards.org/cgi-bin/carddisp.pl?gene=FADS3
HLA-A	Major Histocompatibility Complex, Class I, A	Protein Coding	48	GC06P055161	26.8138	https://www.genecards.org/cgi-bin/carddisp.pl?gene=HLA-A
FABP6	Fatty Acid Binding Protein 6	Protein Coding	43	GC05P160187	26.81321	https://www.genecards.org/cgi-bin/carddisp.pl?gene=FABP6
SOCS3	Suppressor Of Cytokine Signaling 3	Protein Coding	45	GC17M078356	26.81011	https://www.genecards.org/cgi-bin/carddisp.pl?gene=SOCS3
CCN2	Cellular Communication Network Factor 2	Protein Coding	40	GC06M131948	26.80043	https://www.genecards.org/cgi-bin/carddisp.pl?gene=CCN2
PRPH2	Peripherin 2	Protein Coding	39	GC06M047116	26.73267	https://www.genecards.org/cgi-bin/carddisp.pl?gene=PRPH2
GPC3	Glypican 3	Protein Coding	46	GC0XM133535	26.71111	https://www.genecards.org/cgi-bin/carddisp.pl?gene=GPC3
FAH	Fumarylacetoacetate Hydrolase	Protein Coding	47	GC15P080152	26.70848	https://www.genecards.org/cgi-bin/carddisp.pl?gene=FAH
DPP4	Dipeptidyl Peptidase 4	Protein Coding	50	GC02M161992	26.70602	https://www.genecards.org/cgi-bin/carddisp.pl?gene=DPP4
PTPRC	Protein Tyrosine Phosphatase Receptor Type C	Protein Coding	51	GC01P198607	26.60435	https://www.genecards.org/cgi-bin/carddisp.pl?gene=PTPRC
MEN1	Menin 1	Protein Coding	47	GC11M064803	26.59502	https://www.genecards.org/cgi-bin/carddisp.pl?gene=MEN1
FLNA	Filamin A	Protein Coding	50	GC0XM154348	26.5132	https://www.genecards.org/cgi-bin/carddisp.pl?gene=FLNA

PON2	Paraoxonase 2	Protein Coding	44	GC07M095404	26.49184	https://www.genecards.org/cgi-bin/carddisp.pl?gene=PON2
CDKN1A	Cyclin Dependent Kinase Inhibitor 1A	Protein Coding	50	GC06P055348	26.4918	https://www.genecards.org/cgi-bin/carddisp.pl?gene=CDKN1A
SCT	Secretin	Protein Coding	35	GC11M000626	26.48512	https://www.genecards.org/cgi-bin/carddisp.pl?gene=SCT
CFI	Complement Factor I	Protein Coding	47	GC04M109732	26.47642	https://www.genecards.org/cgi-bin/carddisp.pl?gene=CFI
NGF	Nerve Growth Factor	Protein Coding	51	GC01M115285	26.4562	https://www.genecards.org/cgi-bin/carddisp.pl?gene=NGF
PDGFRB	Platelet Derived Growth Factor Receptor Beta	Protein Coding	55	GC05M150113	26.45407	https://www.genecards.org/cgi-bin/carddisp.pl?gene=PDGFRB
PKLR	Pyruvate Kinase L/R	Protein Coding	48	GC01M155289	26.42625	https://www.genecards.org/cgi-bin/carddisp.pl?gene=PKLR
RUNX1	RUNX Family Transcription Factor 1	Protein Coding	49	GC21M034787	26.42223	https://www.genecards.org/cgi-bin/carddisp.pl?gene=RUNX1
JAK1	Janus Kinase 1	Protein Coding	54	GC01M064833	26.41035	https://www.genecards.org/cgi-bin/carddisp.pl?gene=JAK1
MMACHC	Metabolism Of Cobalamin Associated C	Protein Coding	44	GC01P045500	26.35614	https://www.genecards.org/cgi-bin/carddisp.pl?gene=MMACHC
GPD1	Glycerol-3-Phosphate Dehydrogenase 1	Protein Coding	46	GC12P050105	26.32545	https://www.genecards.org/cgi-bin/carddisp.pl?gene=GPD1
FGF21	Fibroblast Growth Factor 21	Protein Coding	40	GC19P048781	26.31933	https://www.genecards.org/cgi-bin/carddisp.pl?gene=FGF21
MAPK14	Mitogen-Activated Protein Kinase 14	Protein Coding	52	GC06P055339	26.3193	https://www.genecards.org/cgi-bin/carddisp.pl?gene=MAPK14
RAB7A	RAB7A, Member RAS Oncogene Family	Protein Coding	49	GC03P131442	26.28293	https://www.genecards.org/cgi-bin/carddisp.pl?gene=RAB7A
CYP7B1	Cytochrome P450 Family 7 Subfamily B Member 1	Protein Coding	46	GC08M064587	26.24104	https://www.genecards.org/cgi-bin/carddisp.pl?gene=CYP7B1
ALOX5	Arachidonate 5-Lipoxygenase	Protein Coding	50	GC10P045374	26.23742	https://www.genecards.org/cgi-bin/carddisp.pl?gene=ALOX5
THPO	Thrombopoietin	Protein Coding	43	GC03M184371	26.23381	https://www.genecards.org/cgi-bin/carddisp.pl?gene=THPO
F7	Coagulation Factor VII	Protein Coding	48	GC13P113105	26.22255	https://www.genecards.org/cgi-bin/carddisp.pl?gene=F7
ACAD9	Acyl-CoA Dehydrogenase Family Member 9	Protein Coding	44	GC03P131450	26.1841	https://www.genecards.org/cgi-bin/carddisp.pl?gene=ACAD9
IL5	Interleukin 5	Protein Coding	45	GC05M132541	26.13515	https://www.genecards.org/cgi-bin/carddisp.pl?gene=IL5

IKBKB	Inhibitor Of Nuclear Factor Kappa B Kinase Subunit Beta	Protein Coding	53	GC08P042271	26.09329	https://www.genecards.org/cgi-bin/carddisp.pl?gene=IKBKB
NAGA	Alpha-N-Acetylgalactosaminidase	Protein Coding	44	GC22M042058	26.08938	https://www.genecards.org/cgi-bin/carddisp.pl?gene=NAGA
TGFB3	Transforming Growth Factor Beta 3	Protein Coding	47	GC14M075958	26.07246	https://www.genecards.org/cgi-bin/carddisp.pl?gene=TGFB3
VCAM1	Vascular Cell Adhesion Molecule 1	Protein Coding	46	GC01P100719	26.07201	https://www.genecards.org/cgi-bin/carddisp.pl?gene=VCAM1
NHLRC1	NHL Repeat Containing E3 Ubiquitin Protein Ligase 1	Protein Coding	41	GC06M018120	26.04428	https://www.genecards.org/cgi-bin/carddisp.pl?gene=NHLRC1
EP300	E1A Binding Protein P300	Protein Coding	51	GC22P041091	26.03346	https://www.genecards.org/cgi-bin/carddisp.pl?gene=EP300
PDCD1	Programmed Cell Death 1	Protein Coding	48	GC02M241849	25.99944	https://www.genecards.org/cgi-bin/carddisp.pl?gene=PDCD1
IL1R1	Interleukin 1 Receptor Type 1	Protein Coding	46	GC02P102136	25.99226	https://www.genecards.org/cgi-bin/carddisp.pl?gene=IL1R1
GATA6	GATA Binding Protein 6	Protein Coding	48	GC18P022169	25.97951	https://www.genecards.org/cgi-bin/carddisp.pl?gene=GATA6
GJB2	Gap Junction Protein Beta 2	Protein Coding	47	GC13M020187	25.95803	https://www.genecards.org/cgi-bin/carddisp.pl?gene=GJB2
MT-ND4	Mitochondrially Encoded NADH:Ubiquinone Oxidoreductase Core Subunit 4	Protein Coding	33	GCMTP010762	25.91657	https://www.genecards.org/cgi-bin/carddisp.pl?gene=MT-ND4
PLA2G2A	Phospholipase A2 Group IIA	Protein Coding	48	GC01M019975	25.91336	https://www.genecards.org/cgi-bin/carddisp.pl?gene=PLA2G2A
SDHA	Succinate Dehydrogenase Complex Flavoprotein Subunit A	Protein Coding	48	GC05P000208	25.90883	https://www.genecards.org/cgi-bin/carddisp.pl?gene=SDHA
CEL	Carboxyl Ester Lipase	Protein Coding	47	GC09P133061	25.87259	https://www.genecards.org/cgi-bin/carddisp.pl?gene=CEL
PRF1	Perforin 1	Protein Coding	45	GC10M070597	25.84634	https://www.genecards.org/cgi-bin/carddisp.pl?gene=PRF1
CD79A	CD79a Molecule	Protein Coding	47	GC19P041877	25.84297	https://www.genecards.org/cgi-bin/carddisp.pl?gene=CD79A
AWAT2	Acyl-CoA Wax Alcohol Acyltransferase 2	Protein Coding	33	GC0XM070040	25.84177	https://www.genecards.org/cgi-bin/carddisp.pl?gene=AWAT2
BGLAP	Bone Gamma-Carboxyglutamate Protein	Protein Coding	41	GC01P156242	25.82944	https://www.genecards.org/cgi-bin/carddisp.pl?gene=BGLAP

UGT1A6	UDP Glucuronosyltransferase Family 1 Member A6	Protein Coding	42	GC02P233691	25.82225	https://www.genecards.org/cgi-bin/carddisp.pl?gene=UGT1A6
CBS	Cystathionine Beta-Synthase	Protein Coding	49	GC21M043053	25.80807	https://www.genecards.org/cgi-bin/carddisp.pl?gene=CBS
KCNJ11	Potassium Inwardly Rectifying Channel Subfamily J Member 11	Protein Coding	48	GC11M017385	25.79145	https://www.genecards.org/cgi-bin/carddisp.pl?gene=KCNJ11
MLH1	MutL Homolog 1	Protein Coding	49	GC03P036993	25.77318	https://www.genecards.org/cgi-bin/carddisp.pl?gene=MLH1
SFTPC	Surfactant Protein C	Protein Coding	44	GC08P022156	25.76713	https://www.genecards.org/cgi-bin/carddisp.pl?gene=SFTPC
ITGAM	Integrin Subunit Alpha M	Protein Coding	47	GC16P032553	25.75382	https://www.genecards.org/cgi-bin/carddisp.pl?gene=ITGAM
MT-ND3	Mitochondrially Encoded NADH:Ubiquinone Oxidoreductase Core Subunit 3	Protein Coding	33	GCMTP010061	25.74694	https://www.genecards.org/cgi-bin/carddisp.pl?gene=MT-ND3
NFE2L2	Nuclear Factor, Erythroid 2 Like 2	Protein Coding	50	GC02M177227	25.7454	https://www.genecards.org/cgi-bin/carddisp.pl?gene=NFE2L2
CDKN1B	Cyclin Dependent Kinase Inhibitor 1B	Protein Coding	48	GC12P012722	25.70904	https://www.genecards.org/cgi-bin/carddisp.pl?gene=CDKN1B
NFKBIA	NFKB Inhibitor Alpha	Protein Coding	51	GC14M035401	25.69777	https://www.genecards.org/cgi-bin/carddisp.pl?gene=NFKBIA
H19	H19 Imprinted Maternally Expressed Transcript	RNA Gene	28	GC11M001995	25.68446	https://www.genecards.org/cgi-bin/carddisp.pl?gene=H19
NDUFV1	NADH:Ubiquinone Oxidoreductase Core Subunit V1	Protein Coding	47	GC11P067670	25.68073	https://www.genecards.org/cgi-bin/carddisp.pl?gene=NDUFV1
MT-ND6	Mitochondrially Encoded NADH:Ubiquinone Oxidoreductase Core Subunit 6	Protein Coding	33	GCMTM014151	25.66519	https://www.genecards.org/cgi-bin/carddisp.pl?gene=MT-ND6
IFNA2	Interferon Alpha 2	Protein Coding	41	GC09M021384	25.65508	https://www.genecards.org/cgi-bin/carddisp.pl?gene=IFNA2
KRT7	Keratin 7	Protein Coding	43	GC12P052232	25.65355	https://www.genecards.org/cgi-bin/carddisp.pl?gene=KRT7

OTC	Ornithine Transcarbamylase	Protein Coding	48	GC0XP038353	25.65215	https://www.genecards.org/cgi-bin/carddisp.pl?gene=OTC
NR1I2	Nuclear Receptor Subfamily 1 Group I Member 2	Protein Coding	47	GC03P119780	25.61819	https://www.genecards.org/cgi-bin/carddisp.pl?gene=NR1I2
SHH	Sonic Hedgehog Signaling Molecule	Protein Coding	50	GC07M155799	25.60373	https://www.genecards.org/cgi-bin/carddisp.pl?gene=SHH
KRT19	Keratin 19	Protein Coding	46	GC17M041523	25.59081	https://www.genecards.org/cgi-bin/carddisp.pl?gene=KRT19
COL4A5	Collagen Type IV Alpha 5 Chain	Protein Coding	44	GC0XP108439	25.56473	https://www.genecards.org/cgi-bin/carddisp.pl?gene=COL4A5
PRSS1	Serine Protease 1	Protein Coding	47	GC07P145775	25.55352	https://www.genecards.org/cgi-bin/carddisp.pl?gene=PRSS1
AGPAT2	1-Acylglycerol-3-Phosphate O-Acyltransferase 2	Protein Coding	47	GC09M136673	25.49581	https://www.genecards.org/cgi-bin/carddisp.pl?gene=AGPAT2
FGF2	Fibroblast Growth Factor 2	Protein Coding	48	GC04P122826	25.4317	https://www.genecards.org/cgi-bin/carddisp.pl?gene=FGF2
CCK	Cholecystokinin	Protein Coding	43	GC03M042274	25.3374	https://www.genecards.org/cgi-bin/carddisp.pl?gene=CCK
CD34	CD34 Molecule	Protein Coding	44	GC01M207880	25.33303	https://www.genecards.org/cgi-bin/carddisp.pl?gene=CD34
ADRB2	Adrenoceptor Beta 2	Protein Coding	50	GC05P148825	25.30973	https://www.genecards.org/cgi-bin/carddisp.pl?gene=ADRB2
ATF4	Activating Transcription Factor 4	Protein Coding	47	GC22P039547	25.30847	https://www.genecards.org/cgi-bin/carddisp.pl?gene=ATF4
SAG	S-Antigen Visual Arrestin	Protein Coding	45	GC02P233328	25.30161	https://www.genecards.org/cgi-bin/carddisp.pl?gene=SAG
C4A	Complement C4A (Rodgers Blood Group)	Protein Coding	44	GC06P055224	25.29449	https://www.genecards.org/cgi-bin/carddisp.pl?gene=C4A
XDH	Xanthine Dehydrogenase	Protein Coding	47	GC02M031334	25.29173	https://www.genecards.org/cgi-bin/carddisp.pl?gene=XDH
A2M	Alpha-2-Macroglobulin	Protein Coding	47	GC12M009067	25.28257	https://www.genecards.org/cgi-bin/carddisp.pl?gene=A2M
PMS2	PMS1 Homolog 2, Mismatch Repair System Component	Protein Coding	49	GC07M005973	25.28156	https://www.genecards.org/cgi-bin/carddisp.pl?gene=PMS2
OCRL	OCRL Inositol Polyphosphate-5-Phosphatase	Protein Coding	45	GC0XP129539	25.27095	https://www.genecards.org/cgi-bin/carddisp.pl?gene=OCRL

FGF23	Fibroblast Growth Factor 23	Protein Coding	46	GC12M004368	25.26514	https://www.genecards.org/cgi-bin/carddisp.pl?gene=FGF23
ALDH1A1	Aldehyde Dehydrogenase 1 Family Member A1	Protein Coding	48	GC09M072900	25.25164	https://www.genecards.org/cgi-bin/carddisp.pl?gene=ALDH1A1
COL7A1	Collagen Type VII Alpha 1 Chain	Protein Coding	45	GC03M048564	25.24354	https://www.genecards.org/cgi-bin/carddisp.pl?gene=COL7A1
TLR5	Toll Like Receptor 5	Protein Coding	48	GC01M223132	25.23612	https://www.genecards.org/cgi-bin/carddisp.pl?gene=TLR5
BMPR2	Bone Morphogenetic Protein Receptor Type 2	Protein Coding	50	GC02P202376	25.21325	https://www.genecards.org/cgi-bin/carddisp.pl?gene=BMPR2
RHO	Rhodopsin	Protein Coding	48	GC03P131457	25.18714	https://www.genecards.org/cgi-bin/carddisp.pl?gene=RHO
GLUL	Glutamate-Ammonia Ligase	Protein Coding	50	GC01M182378	25.17989	https://www.genecards.org/cgi-bin/carddisp.pl?gene=GLUL
KRT5	Keratin 5	Protein Coding	48	GC12M052514	25.13056	https://www.genecards.org/cgi-bin/carddisp.pl?gene=KRT5
ABL1	ABL Proto-Oncogene 1, Non-Receptor Tyrosine Kinase	Protein Coding	53	GC09P130713	25.09013	https://www.genecards.org/cgi-bin/carddisp.pl?gene=ABL1
CD28	CD28 Molecule	Protein Coding	47	GC02P203706	25.08379	https://www.genecards.org/cgi-bin/carddisp.pl?gene=CD28
NAMPT	Nicotinamide Phosphoribosyltransferase	Protein Coding	48	GC07M106248	25.07551	https://www.genecards.org/cgi-bin/carddisp.pl?gene=NAMPT
HSPA4	Heat Shock Protein Family A (Hsp70) Member 4	Protein Coding	43	GC05P133051	25.06934	https://www.genecards.org/cgi-bin/carddisp.pl?gene=HSPA4
MIR200C	MicroRNA 200c	RNA Gene	21	GC12P011871	25.04626	https://www.genecards.org/cgi-bin/carddisp.pl?gene=MIR200C
ATRX	ATRX Chromatin Remodeler	Protein Coding	47	GC0XM077504	25.04457	https://www.genecards.org/cgi-bin/carddisp.pl?gene=ATRX
FGA	Fibrinogen Alpha Chain	Protein Coding	48	GC04M154583	25.01811	https://www.genecards.org/cgi-bin/carddisp.pl?gene=FGA
TREM2	Triggering Receptor Expressed On Myeloid Cells 2	Protein Coding	43	GC06M047080	25.00278	https://www.genecards.org/cgi-bin/carddisp.pl?gene=TREM2
UROD	Uroporphyrinogen Decarboxylase	Protein Coding	46	GC01P045023	24.99612	https://www.genecards.org/cgi-bin/carddisp.pl?gene=UROD
FTL	Ferritin Light Chain	Protein Coding	48	GC19P048965	24.99425	https://www.genecards.org/cgi-bin/carddisp.pl?gene=FTL
SMN1	Survival Of Motor Neuron 1, Telomeric	Protein Coding	42	GC05P070924	24.97404	https://www.genecards.org/cgi-bin/carddisp.pl?gene=SMN1

STK11	Serine/Threonine Kinase 11	Protein Coding	50	GC19P001177	24.973	https://www.genecards.org/cgi-bin/carddisp.pl?gene=STK11
IGFBP1	Insulin Like Growth Factor Binding Protein 1	Protein Coding	44	GC07P046838	24.9713	https://www.genecards.org/cgi-bin/carddisp.pl?gene=IGFBP1
FKRP	Fukutin Related Protein	Protein Coding	41	GC19P046746	24.95414	https://www.genecards.org/cgi-bin/carddisp.pl?gene=FKRP
TFRC	Transferrin Receptor	Protein Coding	49	GC03M196027	24.94923	https://www.genecards.org/cgi-bin/carddisp.pl?gene=TFRC
GNE	Glucosamine (UDP-N-Acetyl)-2-Epimerase/N-Acetylmannosamine Kinase	Protein Coding	44	GC09M036214	24.94873	https://www.genecards.org/cgi-bin/carddisp.pl?gene=GNE
CYP19A1	Cytochrome P450 Family 19 Subfamily A Member 1	Protein Coding	50	GC15M051208	24.94394	https://www.genecards.org/cgi-bin/carddisp.pl?gene=CYP19A1
IGFBP3	Insulin Like Growth Factor Binding Protein 3	Protein Coding	45	GC07M045912	24.93682	https://www.genecards.org/cgi-bin/carddisp.pl?gene=IGFBP3
CTSB	Cathepsin B	Protein Coding	51	GC08M011842	24.92962	https://www.genecards.org/cgi-bin/carddisp.pl?gene=CTSB
AVP	Arginine Vasopressin	Protein Coding	47	GC20M003082	24.90945	https://www.genecards.org/cgi-bin/carddisp.pl?gene=AVP
FFAR4	Free Fatty Acid Receptor 4	Protein Coding	39	GC10P093566	24.85429	https://www.genecards.org/cgi-bin/carddisp.pl?gene=FFAR4
SLPI	Secretory Leukocyte Peptidase Inhibitor	Protein Coding	40	GC20M045252	24.80633	https://www.genecards.org/cgi-bin/carddisp.pl?gene=SLPI
MTUS1	Microtubule Associated Scaffold Protein 1	Protein Coding	37	GC08M017643	24.78032	https://www.genecards.org/cgi-bin/carddisp.pl?gene=MTUS1
SOS1	SOS Ras/Rac Guanine Nucleotide Exchange Factor 1	Protein Coding	49	GC02M038981	24.74988	https://www.genecards.org/cgi-bin/carddisp.pl?gene=SOS1
AIRE	Autoimmune Regulator	Protein Coding	45	GC21P044285	24.74964	https://www.genecards.org/cgi-bin/carddisp.pl?gene=AIRE
ERCC2	ERCC Excision Repair 2, TFIIH Core Complex Helicase Subunit	Protein Coding	48	GC19M045349	24.74029	https://www.genecards.org/cgi-bin/carddisp.pl?gene=ERCC2
TMEM216	Transmembrane Protein 216	Protein Coding	36	GC11P061391	24.72112	https://www.genecards.org/cgi-bin/carddisp.pl?gene=TMEM216
TG	Thyroglobulin	Protein Coding	43	GC08P132866	24.70642	https://www.genecards.org/cgi-bin/carddisp.pl?gene=TG
MIRLET7D	MicroRNA Let-7d	RNA Gene	21	GC09P094178	24.70107	https://www.genecards.org/cgi-bin/carddisp.pl?gene=MIRLET7D

TLR3	Toll Like Receptor 3	Protein Coding	52	GC04P186059	24.66375	https://www.genecards.org/cgi-bin/carddisp.pl?gene=TLR3
CLU	Clusterin	Protein Coding	47	GC08M027596	24.64778	https://www.genecards.org/cgi-bin/carddisp.pl?gene=CLU
TFAM	Transcription Factor A, Mitochondrial	Protein Coding	45	GC10P058385	24.63551	https://www.genecards.org/cgi-bin/carddisp.pl?gene=TFAM
FFAR2	Free Fatty Acid Receptor 2	Protein Coding	41	GC19P040347	24.5946	https://www.genecards.org/cgi-bin/carddisp.pl?gene=FFAR2
UGT1A8	UDP Glucuronosyltransferase Family 1 Member A8	Protein Coding	36	GC02P233618	24.59324	https://www.genecards.org/cgi-bin/carddisp.pl?gene=UGT1A8
FECH	Ferrochelatase	Protein Coding	47	GC18M057544	24.57081	https://www.genecards.org/cgi-bin/carddisp.pl?gene=FECH
PAH	Phenylalanine Hydroxylase	Protein Coding	49	GC12M102836	24.5406	https://www.genecards.org/cgi-bin/carddisp.pl?gene=PAH
CSTB	Cystatin B	Protein Coding	47	GC21M043772	24.53864	https://www.genecards.org/cgi-bin/carddisp.pl?gene=CSTB
SREBF2	Sterol Regulatory Element Binding Transcription Factor 2	Protein Coding	43	GC22P041833	24.51161	https://www.genecards.org/cgi-bin/carddisp.pl?gene=SREBF2
AIFM1	Apoptosis Inducing Factor Mitochondria Associated 1	Protein Coding	50	GC0XM130129	24.51155	https://www.genecards.org/cgi-bin/carddisp.pl?gene=AIFM1
TBP	TATA-Box Binding Protein	Protein Coding	49	GC06P170554	24.50735	https://www.genecards.org/cgi-bin/carddisp.pl?gene=TBP
SRC	SRC Proto-Oncogene, Non-Receptor Tyrosine Kinase	Protein Coding	52	GC20P037344	24.50633	https://www.genecards.org/cgi-bin/carddisp.pl?gene=SRC
POMGNT1	Protein O-Linked Mannose N-Acetylglucosaminyltransferase 1 (Beta 1,2-)	Protein Coding	45	GC01M046188	24.46222	https://www.genecards.org/cgi-bin/carddisp.pl?gene=POMGNT1
ACOX2	Acyl-CoA Oxidase 2	Protein Coding	44	GC03M058490	24.44621	https://www.genecards.org/cgi-bin/carddisp.pl?gene=ACOX2
CHEK2	Checkpoint Kinase 2	Protein Coding	54	GC22M028687	24.44594	https://www.genecards.org/cgi-bin/carddisp.pl?gene=CHEK2
RXRA	Retinoid X Receptor Alpha	Protein Coding	51	GC09P134317	24.36722	https://www.genecards.org/cgi-bin/carddisp.pl?gene=RXRA
ITCH	Itchy E3 Ubiquitin Protein Ligase	Protein Coding	46	GC20P034363	24.35444	https://www.genecards.org/cgi-bin/carddisp.pl?gene=ITCH

UGT1A9	UDP Glucuronosyltransferase Family 1 Member A9	Protein Coding	44	GC02P233671	24.33155	https://www.genecards.org/cgi-bin/carddisp.pl?gene=UGT1A9
ACTA1	Actin Alpha 1, Skeletal Muscle	Protein Coding	48	GC01M229431	24.2884	https://www.genecards.org/cgi-bin/carddisp.pl?gene=ACTA1
DRD4	Dopamine Receptor D4	Protein Coding	47	GC11P000979	24.28248	https://www.genecards.org/cgi-bin/carddisp.pl?gene=DRD4
COL4A4	Collagen Type IV Alpha 4 Chain	Protein Coding	44	GC02M226971	24.27524	https://www.genecards.org/cgi-bin/carddisp.pl?gene=COL4A4
CXCL10	C-X-C Motif Chemokine Ligand 10	Protein Coding	45	GC04M076021	24.26254	https://www.genecards.org/cgi-bin/carddisp.pl?gene=CXCL10
TBX5	T-Box Transcription Factor 5	Protein Coding	47	GC12M114353	24.25562	https://www.genecards.org/cgi-bin/carddisp.pl?gene=TBX5
MALAT1	Metastasis Associated Lung Adenocarcinoma Transcript 1	RNA Gene	23	GC11P066225	24.24067	https://www.genecards.org/cgi-bin/carddisp.pl?gene=MALAT1
IFIH1	Interferon Induced With Helicase C Domain 1	Protein Coding	48	GC02M162267	24.21241	https://www.genecards.org/cgi-bin/carddisp.pl?gene=IFIH1
RB1	RB Transcriptional Corepressor 1	Protein Coding	49	GC13P048303	24.19917	https://www.genecards.org/cgi-bin/carddisp.pl?gene=RB1
CYP2B6	Cytochrome P450 Family 2 Subfamily B Member 6	Protein Coding	48	GC19P040991	24.1731	https://www.genecards.org/cgi-bin/carddisp.pl?gene=CYP2B6
EIF2S1	Eukaryotic Translation Initiation Factor 2 Subunit Alpha	Protein Coding	45	GC14P067359	24.17068	https://www.genecards.org/cgi-bin/carddisp.pl?gene=EIF2S1
TGFBR1	Transforming Growth Factor Beta Receptor 1	Protein Coding	54	GC09P099104	24.16077	https://www.genecards.org/cgi-bin/carddisp.pl?gene=TGFBR1
MIRLET7C	MicroRNA Let-7c	RNA Gene	22	GC21P016551	24.12523	https://www.genecards.org/cgi-bin/carddisp.pl?gene=MIRLET7C
EPCAM	Epithelial Cell Adhesion Molecule	Protein Coding	48	GC02P047345	24.09324	https://www.genecards.org/cgi-bin/carddisp.pl?gene=EPCAM
STAT5B	Signal Transducer And Activator Of Transcription 5B	Protein Coding	50	GC17M042199	24.09065	https://www.genecards.org/cgi-bin/carddisp.pl?gene=STAT5B
TYR	Tyrosinase	Protein Coding	48	GC11P089177	24.0699	https://www.genecards.org/cgi-bin/carddisp.pl?gene=TYR

DNMT1	DNA Methyltransferase 1	Protein Coding	51	GC19M010133	24.05653	https://www.genecards.org/cgi-bin/carddisp.pl?gene=DNMT1
CYP1B1	Cytochrome P450 Family 1 Subfamily B Member 1	Protein Coding	49	GC02M038066	24.00853	https://www.genecards.org/cgi-bin/carddisp.pl?gene=CYP1B1
BBS10	Bardet-Biedl Syndrome 10	Protein Coding	41	GC12M076344	23.99728	https://www.genecards.org/cgi-bin/carddisp.pl?gene=BBS10
MTM1	Myotubularin 1	Protein Coding	45	GC0XP150562	23.9802	https://www.genecards.org/cgi-bin/carddisp.pl?gene=MTM1
CRH	Corticotropin Releasing Hormone	Protein Coding	44	GC08M066176	23.97061	https://www.genecards.org/cgi-bin/carddisp.pl?gene=CRH
GJC2	Gap Junction Protein Gamma 2	Protein Coding	42	GC01P228471	23.95737	https://www.genecards.org/cgi-bin/carddisp.pl?gene=GJC2
DBH	Dopamine Beta-Hydroxylase	Protein Coding	51	GC09P133636	23.94764	https://www.genecards.org/cgi-bin/carddisp.pl?gene=DBH
SPTLC1	Serine Palmitoyltransferase Long Chain Base Subunit 1	Protein Coding	47	GC09M092002	23.94386	https://www.genecards.org/cgi-bin/carddisp.pl?gene=SPTLC1
IDH1	Isocitrate Dehydrogenase (NADP(+)) 1	Protein Coding	53	GC02M208236	23.92354	https://www.genecards.org/cgi-bin/carddisp.pl?gene=IDH1
CD40	CD40 Molecule	Protein Coding	48	GC20P046118	23.91717	https://www.genecards.org/cgi-bin/carddisp.pl?gene=CD40
STAT4	Signal Transducer And Activator Of Transcription 4	Protein Coding	47	GC02M191029	23.90696	https://www.genecards.org/cgi-bin/carddisp.pl?gene=STAT4
CXCR4	C-X-C Motif Chemokine Receptor 4	Protein Coding	52	GC02M136114	23.90555	https://www.genecards.org/cgi-bin/carddisp.pl?gene=CXCR4
BBS1	Bardet-Biedl Syndrome 1	Protein Coding	38	GC11P066772	23.9034	https://www.genecards.org/cgi-bin/carddisp.pl?gene=BBS1
KL	Klotho	Protein Coding	46	GC13P033016	23.86306	https://www.genecards.org/cgi-bin/carddisp.pl?gene=KL
SDHC	Succinate Dehydrogenase Complex Subunit C	Protein Coding	45	GC01P161314	23.86123	https://www.genecards.org/cgi-bin/carddisp.pl?gene=SDHC
ERCC1	ERCC Excision Repair 1, Endonuclease Non-Catalytic Subunit	Protein Coding	47	GC19M046972	23.84486	https://www.genecards.org/cgi-bin/carddisp.pl?gene=ERCC1
PRODH	Proline Dehydrogenase 1	Protein Coding	47	GC22M018912	23.80731	https://www.genecards.org/cgi-bin/carddisp.pl?gene=PRODH
PRTN3	Proteinase 3	Protein Coding	45	GC19P000840	23.80523	https://www.genecards.org/cgi-bin/carddisp.pl?gene=PRTN3
MIR141	MicroRNA 141	RNA Gene	22	GC12P011872	23.80027	https://www.genecards.org/cgi-bin/carddisp.pl?gene=MIR141

MAOB	Monoamine Oxidase B	Protein Coding	44	GC0XM043766	23.80009	https://www.genecards.org/cgi-bin/carddisp.pl?gene=MAOB
CYP4A11	Cytochrome P450 Family 4 Subfamily A Member 11	Protein Coding	44	GC01M046929	23.78612	https://www.genecards.org/cgi-bin/carddisp.pl?gene=CYP4A11
SLC25A4	Solute Carrier Family 25 Member 4	Protein Coding	50	GC04P185143	23.78164	https://www.genecards.org/cgi-bin/carddisp.pl?gene=SLC25A4
MT-CO2	Mitochondrially Encoded Cytochrome C Oxidase II	Protein Coding	34	GCMTP007587	23.76284	https://www.genecards.org/cgi-bin/carddisp.pl?gene=MT-CO2
EPHX1	Epoxide Hydrolase 1	Protein Coding	48	GC01P225810	23.75412	https://www.genecards.org/cgi-bin/carddisp.pl?gene=EPHX1
TNFSF10	TNF Superfamily Member 10	Protein Coding	47	GC03M172505	23.74385	https://www.genecards.org/cgi-bin/carddisp.pl?gene=TNFSF10
DCTN1	Dynactin Subunit 1	Protein Coding	47	GC02M074361	23.73948	https://www.genecards.org/cgi-bin/carddisp.pl?gene=DCTN1
UGT1A	UDP Glucuronosyltransferase Family 1 Member A Complex Locus	Genetic Locus	10	GC02P233592	23.72621	https://www.genecards.org/cgi-bin/carddisp.pl?gene=UGT1A
TLR9	Toll Like Receptor 9	Protein Coding	45	GC03M052222	23.72428	https://www.genecards.org/cgi-bin/carddisp.pl?gene=TLR9
SELP	Selectin P	Protein Coding	46	GC01M169558	23.72334	https://www.genecards.org/cgi-bin/carddisp.pl?gene=SELP
MAPK3	Mitogen-Activated Protein Kinase 3	Protein Coding	50	GC16M031188	23.71683	https://www.genecards.org/cgi-bin/carddisp.pl?gene=MAPK3
FUS	FUS RNA Binding Protein	Protein Coding	45	GC16P031180	23.71544	https://www.genecards.org/cgi-bin/carddisp.pl?gene=FUS
CAPN3	Calpain 3	Protein Coding	48	GC15P042359	23.69723	https://www.genecards.org/cgi-bin/carddisp.pl?gene=CAPN3
SHBG	Sex Hormone Binding Globulin	Protein Coding	41	GC17P007613	23.66805	https://www.genecards.org/cgi-bin/carddisp.pl?gene=SHBG
IL12B	Interleukin 12B	Protein Coding	44	GC05M159314	23.66675	https://www.genecards.org/cgi-bin/carddisp.pl?gene=IL12B
CALCA	Calcitonin Related Polypeptide Alpha	Protein Coding	43	GC11M014945	23.66144	https://www.genecards.org/cgi-bin/carddisp.pl?gene=CALCA
TYMP	Thymidine Phosphorylase	Protein Coding	47	GC22M050525	23.65818	https://www.genecards.org/cgi-bin/carddisp.pl?gene=TYMP
SEPSECS	Sep (O-Phosphoserine) TRNA:Sec (Selenocysteine) TRNA Synthase	Protein Coding	44	GC04M025121	23.65126	https://www.genecards.org/cgi-bin/carddisp.pl?gene=SEPSECS
MDM2	MDM2 Proto-Oncogene	Protein Coding	53	GC12P068808	23.63696	https://www.genecards.org/cgi-bin/carddisp.pl?gene=MDM2

CALR	Calreticulin	Protein Coding	51	GC19P012938	23.61307	https://www.genecards.org/cgi-bin/carddisp.pl?gene=CALR
TNFSF11	TNF Superfamily Member 11	Protein Coding	48	GC13P042562	23.60391	https://www.genecards.org/cgi-bin/carddisp.pl?gene=TNFSF11
TMEM231	Transmembrane Protein 231	Protein Coding	37	GC16M075536	23.59649	https://www.genecards.org/cgi-bin/carddisp.pl?gene=TMEM231
NPPA	Natriuretic Peptide A	Protein Coding	47	GC01M011846	23.58355	https://www.genecards.org/cgi-bin/carddisp.pl?gene=NPPA
ACVRL1	Activin A Receptor Like Type 1	Protein Coding	50	GC12P051906	23.57769	https://www.genecards.org/cgi-bin/carddisp.pl?gene=ACVRL1
MIR29B1	MicroRNA 29b-1	RNA Gene	20	GC07M130877	23.56023	https://www.genecards.org/cgi-bin/carddisp.pl?gene=MIR29B1
TPP1	Tripeptidyl Peptidase 1	Protein Coding	43	GC11M006620	23.55012	https://www.genecards.org/cgi-bin/carddisp.pl?gene=TPP1
PDGFB	Platelet Derived Growth Factor Subunit B	Protein Coding	50	GC22M049027	23.53149	https://www.genecards.org/cgi-bin/carddisp.pl?gene=PDGFB
NTRK1	Neurotrophic Receptor Tyrosine Kinase 1	Protein Coding	50	GC01P156815	23.51118	https://www.genecards.org/cgi-bin/carddisp.pl?gene=NTRK1
GATA1	GATA Binding Protein 1	Protein Coding	47	GC0XP048786	23.48822	https://www.genecards.org/cgi-bin/carddisp.pl?gene=GATA1
LCN2	Lipocalin 2	Protein Coding	44	GC09P128149	23.47865	https://www.genecards.org/cgi-bin/carddisp.pl?gene=LCN2
SCO2	Synthesis Of Cytochrome C Oxidase 2	Protein Coding	45	GC22M050523	23.47234	https://www.genecards.org/cgi-bin/carddisp.pl?gene=SCO2
MIR24-2	MicroRNA 24-2	RNA Gene	18	GC19M014108	23.46977	https://www.genecards.org/cgi-bin/carddisp.pl?gene=MIR24-2
S100B	S100 Calcium Binding Protein B	Protein Coding	46	GC21M048326	23.43947	https://www.genecards.org/cgi-bin/carddisp.pl?gene=S100B
ERBB4	Erb-B2 Receptor Tyrosine Kinase 4	Protein Coding	55	GC02M211375	23.41989	https://www.genecards.org/cgi-bin/carddisp.pl?gene=ERBB4
MYBPC3	Myosin Binding Protein C3	Protein Coding	47	GC11M068997	23.41675	https://www.genecards.org/cgi-bin/carddisp.pl?gene=MYBPC3
MVK	Mevalonate Kinase	Protein Coding	50	GC12P109573	23.37072	https://www.genecards.org/cgi-bin/carddisp.pl?gene=MVK
MYH9	Myosin Heavy Chain 9	Protein Coding	50	GC22M036281	23.35895	https://www.genecards.org/cgi-bin/carddisp.pl?gene=MYH9
HBA2	Hemoglobin Subunit Alpha 2	Protein Coding	43	GC16P005491	23.35552	https://www.genecards.org/cgi-bin/carddisp.pl?gene=HBA2
DGAT2	Diacylglycerol O-Acyltransferase 2	Protein Coding	44	GC11P075759	23.35301	https://www.genecards.org/cgi-bin/carddisp.pl?gene=DGAT2

POLG2	DNA Polymerase Gamma 2, Accessory Subunit	Protein Coding	42	GC17M064477	23.35107	https://www.genecards.org/cgi-bin/carddisp.pl?gene=POLG2
DES	Desmin	Protein Coding	48	GC02P219418	23.33959	https://www.genecards.org/cgi-bin/carddisp.pl?gene=DES
KDR	Kinase Insert Domain Receptor	Protein Coding	54	GC04M055078	23.3303	https://www.genecards.org/cgi-bin/carddisp.pl?gene=KDR
MIR16-1	MicroRNA 16-1	RNA Gene	22	GC13M050048	23.32462	https://www.genecards.org/cgi-bin/carddisp.pl?gene=MIR16-1
BTNL2	Butyrophilin Like 2	Protein Coding	39	GC06M032393	23.31023	https://www.genecards.org/cgi-bin/carddisp.pl?gene=BTNL2
NR3C1	Nuclear Receptor Subfamily 3 Group C Member 1	Protein Coding	51	GC05M143277	23.27912	https://www.genecards.org/cgi-bin/carddisp.pl?gene=NR3C1
TERC	Telomerase RNA Component	RNA Gene	29	GC03M169765	23.27304	https://www.genecards.org/cgi-bin/carddisp.pl?gene=TERC
GH1	Growth Hormone 1	Protein Coding	44	GC17M063917	23.26996	https://www.genecards.org/cgi-bin/carddisp.pl?gene=GH1
SPG11	SPG11 Vesicle Trafficking Associated, Spatacsin	Protein Coding	40	GC15M044562	23.25724	https://www.genecards.org/cgi-bin/carddisp.pl?gene=SPG11
MIR148A	MicroRNA 148a	RNA Gene	19	GC07M025993	23.2564	https://www.genecards.org/cgi-bin/carddisp.pl?gene=MIR148A
ATP13A2	ATPase Cation Transporting 13A2	Protein Coding	43	GC01M016985	23.23734	https://www.genecards.org/cgi-bin/carddisp.pl?gene=ATP13A2
PTCH1	Patched 1	Protein Coding	51	GC09M095442	23.2351	https://www.genecards.org/cgi-bin/carddisp.pl?gene=PTCH1
HSPA5	Heat Shock Protein Family A (Hsp70) Member 5	Protein Coding	48	GC09M125234	23.22696	https://www.genecards.org/cgi-bin/carddisp.pl?gene=HSPA5
TREX1	Three Prime Repair Exonuclease 1	Protein Coding	43	GC03P048466	23.19012	https://www.genecards.org/cgi-bin/carddisp.pl?gene=TREX1
DRD1	Dopamine Receptor D1	Protein Coding	45	GC05M175440	23.13916	https://www.genecards.org/cgi-bin/carddisp.pl?gene=DRD1
CD44	CD44 Molecule (Indian Blood Group)	Protein Coding	48	GC11P035139	23.0711	https://www.genecards.org/cgi-bin/carddisp.pl?gene=CD44
MT-TK	Mitochondrially Encoded TRNA-Lys (AAA/G)	RNA Gene	14	GCMTP008297	23.04232	https://www.genecards.org/cgi-bin/carddisp.pl?gene=MT-TK
ZEB2	Zinc Finger E-Box Binding Homeobox 2	Protein Coding	48	GC02M144384	23.03422	https://www.genecards.org/cgi-bin/carddisp.pl?gene=ZEB2
PANK2	Pantothenate Kinase 2	Protein Coding	44	GC20P003887	23.02143	https://www.genecards.org/cgi-bin/carddisp.pl?gene=PANK2

CACNA1A	Calcium Voltage-Gated Channel Subunit Alpha1 A	Protein Coding	47	GC19M013206	22.98386	https://www.genecards.org/cgi-bin/carddisp.pl?gene=CACNA1A
NEFH	Neurofilament Heavy Chain	Protein Coding	47	GC22P029480	22.97906	https://www.genecards.org/cgi-bin/carddisp.pl?gene=NEFH
PLAT	Plasminogen Activator, Tissue Type	Protein Coding	50	GC08M042174	22.97392	https://www.genecards.org/cgi-bin/carddisp.pl?gene=PLAT
PRKACA	Protein Kinase CAMP-Activated Catalytic Subunit Alpha	Protein Coding	52	GC19M014129	22.9361	https://www.genecards.org/cgi-bin/carddisp.pl?gene=PRKACA
PRL	Prolactin	Protein Coding	44	GC06M022287	22.92311	https://www.genecards.org/cgi-bin/carddisp.pl?gene=PRL
PAX2	Paired Box 2	Protein Coding	47	GC10P100735	22.90901	https://www.genecards.org/cgi-bin/carddisp.pl?gene=PAX2
CSF3	Colony Stimulating Factor 3	Protein Coding	40	GC17P040015	22.90438	https://www.genecards.org/cgi-bin/carddisp.pl?gene=CSF3
MIR182	MicroRNA 182	RNA Gene	20	GC07M129770	22.82952	https://www.genecards.org/cgi-bin/carddisp.pl?gene=MIR182
LRAT	Lecithin Retinol Acyltransferase	Protein Coding	45	GC04P154626	22.82145	https://www.genecards.org/cgi-bin/carddisp.pl?gene=LRAT
NTRK2	Neurotrophic Receptor Tyrosine Kinase 2	Protein Coding	53	GC09P084668	22.81317	https://www.genecards.org/cgi-bin/carddisp.pl?gene=NTRK2
MAT1A	Methionine Adenosyltransferase 1A	Protein Coding	48	GC10M080271	22.8094	https://www.genecards.org/cgi-bin/carddisp.pl?gene=MAT1A
PON3	Paraoxonase 3	Protein Coding	45	GC07M095359	22.79139	https://www.genecards.org/cgi-bin/carddisp.pl?gene=PON3
ARSB	Arylsulfatase B	Protein Coding	44	GC05M078777	22.73584	https://www.genecards.org/cgi-bin/carddisp.pl?gene=ARSB
RAD51	RAD51 Recombinase	Protein Coding	53	GC15P040694	22.67805	https://www.genecards.org/cgi-bin/carddisp.pl?gene=RAD51
HMBS	Hydroxymethylbilane Synthase	Protein Coding	45	GC11P119084	22.67586	https://www.genecards.org/cgi-bin/carddisp.pl?gene=HMBS
ADAM10	ADAM Metallopeptidase Domain 10	Protein Coding	53	GC15M058588	22.65889	https://www.genecards.org/cgi-bin/carddisp.pl?gene=ADAM10
APOH	Apolipoprotein H	Protein Coding	44	GC17M066212	22.65094	https://www.genecards.org/cgi-bin/carddisp.pl?gene=APOH
MYH6	Myosin Heavy Chain 6	Protein Coding	47	GC14M023380	22.64859	https://www.genecards.org/cgi-bin/carddisp.pl?gene=MYH6
MTR	5-Methyltetrahydrofolate-Homocysteine Methyltransferase	Protein Coding	47	GC01P236795	22.6412	https://www.genecards.org/cgi-bin/carddisp.pl?gene=MTR
EDAR	Ectodysplasin A Receptor	Protein Coding	42	GC02M108894	22.63719	https://www.genecards.org/cgi-bin/carddisp.pl?gene=EDAR

MIR130A	MicroRNA 130a	RNA Gene	20	GC11P057641	22.59392	https://www.genecards.org/cgi-bin/carddisp.pl?gene=MIR130A
GP1BA	Glycoprotein Ib Platelet Subunit Alpha	Protein Coding	46	GC17P004932	22.59148	https://www.genecards.org/cgi-bin/carddisp.pl?gene=GP1BA
MSR1	Macrophage Scavenger Receptor 1	Protein Coding	47	GC08M016107	22.57181	https://www.genecards.org/cgi-bin/carddisp.pl?gene=MSR1
DRD3	Dopamine Receptor D3	Protein Coding	45	GC03M114128	22.5685	https://www.genecards.org/cgi-bin/carddisp.pl?gene=DRD3
PRKCQ	Protein Kinase C Theta	Protein Coding	50	GC10M006393	22.53702	https://www.genecards.org/cgi-bin/carddisp.pl?gene=PRKCQ
ADA2	Adenosine Deaminase 2	Protein Coding	34	GC22M017179	22.52611	https://www.genecards.org/cgi-bin/carddisp.pl?gene=ADA2
HLA-DPB1	Major Histocompatibility Complex, Class II, DP Beta 1	Protein Coding	44	GC06P055237	22.52042	https://www.genecards.org/cgi-bin/carddisp.pl?gene=HLA-DPB1
SGSH	N-Sulfoglucosamine Sulfohydrolase	Protein Coding	45	GC17M080206	22.50916	https://www.genecards.org/cgi-bin/carddisp.pl?gene=SGSH
PIK3C2A	Phosphatidylinositol-4-Phosphate 3-Kinase Catalytic Subunit Type 2 Alpha	Protein Coding	48	GC11M017273	22.50707	https://www.genecards.org/cgi-bin/carddisp.pl?gene=PIK3C2A
GCH1	GTP Cyclohydrolase 1	Protein Coding	48	GC14M054842	22.45965	https://www.genecards.org/cgi-bin/carddisp.pl?gene=GCH1
KMT2A	Lysine Methyltransferase 2A	Protein Coding	45	GC11P118436	22.44756	https://www.genecards.org/cgi-bin/carddisp.pl?gene=KMT2A
APOC2	Apolipoprotein C2	Protein Coding	45	GC19P044945	22.44405	https://www.genecards.org/cgi-bin/carddisp.pl?gene=APOC2
LTA	Lymphotoxin Alpha	Protein Coding	43	GC06P055200	22.43643	https://www.genecards.org/cgi-bin/carddisp.pl?gene=LTA
UGT1A4	UDP Glucuronosyltransferase Family 1 Member A4	Protein Coding	42	GC02P233718	22.43235	https://www.genecards.org/cgi-bin/carddisp.pl?gene=UGT1A4
LRP6	LDL Receptor Related Protein 6	Protein Coding	49	GC12M015714	22.42312	https://www.genecards.org/cgi-bin/carddisp.pl?gene=LRP6
HTRA2	HtrA Serine Peptidase 2	Protein Coding	47	GC02P074529	22.38318	https://www.genecards.org/cgi-bin/carddisp.pl?gene=HTRA2
MPL	MPL Proto-Oncogene, Thrombopoietin Receptor	Protein Coding	48	GC01P043337	22.36273	https://www.genecards.org/cgi-bin/carddisp.pl?gene=MPL
RARRES2	Retinoic Acid Receptor Responder 2	Protein Coding	40	GC07M150333	22.33598	https://www.genecards.org/cgi-bin/carddisp.pl?gene=RARRES2

ALK	ALK Receptor Tyrosine Kinase	Protein Coding	52	GC02M029190	22.33488	https://www.genecards.org/cgi-bin/carddisp.pl?gene=ALK
ACHE	Acetylcholinesterase (Cartwright Blood Group)	Protein Coding	47	GC07M100889	22.33347	https://www.genecards.org/cgi-bin/carddisp.pl?gene=ACHE
GIGYF2	GRB10 Interacting GYF Protein 2	Protein Coding	40	GC02P232698	22.31904	https://www.genecards.org/cgi-bin/carddisp.pl?gene=GIGYF2
APOL1	Apolipoprotein L1	Protein Coding	43	GC22P036253	22.31812	https://www.genecards.org/cgi-bin/carddisp.pl?gene=APOL1
SPARC	Secreted Protein Acidic And Cysteine Rich	Protein Coding	51	GC05M151661	22.31486	https://www.genecards.org/cgi-bin/carddisp.pl?gene=SPARC
DNAJB2	DnaJ Heat Shock Protein Family (Hsp40) Member B2	Protein Coding	44	GC02P219279	22.30968	https://www.genecards.org/cgi-bin/carddisp.pl?gene=DNAJB2
TET2	Tet Methylcytosine Dioxygenase 2	Protein Coding	45	GC04P105145	22.30623	https://www.genecards.org/cgi-bin/carddisp.pl?gene=TET2
PLG	Plasminogen	Protein Coding	49	GC06P160702	22.30028	https://www.genecards.org/cgi-bin/carddisp.pl?gene=PLG
FLT1	Fms Related Receptor Tyrosine Kinase 1	Protein Coding	51	GC13M028300	22.28722	https://www.genecards.org/cgi-bin/carddisp.pl?gene=FLT1
GABRG2	Gamma-Aminobutyric Acid Type A Receptor Subunit Gamma2	Protein Coding	48	GC05P162000	22.27747	https://www.genecards.org/cgi-bin/carddisp.pl?gene=GABRG2
GLUD1	Glutamate Dehydrogenase 1	Protein Coding	50	GC10M087050	22.2685	https://www.genecards.org/cgi-bin/carddisp.pl?gene=GLUD1
CRAT	Carnitine O-Acetyltransferase	Protein Coding	45	GC09M129094	22.26841	https://www.genecards.org/cgi-bin/carddisp.pl?gene=CRAT
EIF2AK4	Eukaryotic Translation Initiation Factor 2 Alpha Kinase 4	Protein Coding	45	GC15P039934	22.25947	https://www.genecards.org/cgi-bin/carddisp.pl?gene=EIF2AK4
MIF	Macrophage Migration Inhibitory Factor	Protein Coding	50	GC22P023894	22.24175	https://www.genecards.org/cgi-bin/carddisp.pl?gene=MIF
PRKAA2	Protein Kinase AMP-Activated Catalytic Subunit Alpha 2	Protein Coding	51	GC01P056645	22.22001	https://www.genecards.org/cgi-bin/carddisp.pl?gene=PRKAA2

PCK1	Phosphoenolpyruvate Carboxykinase 1	Protein Coding	49	GC20P057561	22.20303	https://www.genecards.org/cgi-bin/carddisp.pl?gene=PCK1
NQO1	NAD(P)H Quinone Dehydrogenase 1	Protein Coding	50	GC16M069706	22.19145	https://www.genecards.org/cgi-bin/carddisp.pl?gene=NQO1
ECE1	Endothelin Converting Enzyme 1	Protein Coding	48	GC01M021217	22.18433	https://www.genecards.org/cgi-bin/carddisp.pl?gene=ECE1
NDUFS2	NADH:Ubiquinone Oxidoreductase Core Subunit S2	Protein Coding	45	GC01P161197	22.11997	https://www.genecards.org/cgi-bin/carddisp.pl?gene=NDUFS2
GHR	Growth Hormone Receptor	Protein Coding	47	GC05P042429	22.09077	https://www.genecards.org/cgi-bin/carddisp.pl?gene=GHR
SP1	Sp1 Transcription Factor	Protein Coding	45	GC12P053380	22.08446	https://www.genecards.org/cgi-bin/carddisp.pl?gene=SP1
AWAT1	Acyl-CoA Wax Alcohol Acyltransferase 1	Protein Coding	29	GC0XP070234	22.06117	https://www.genecards.org/cgi-bin/carddisp.pl?gene=AWAT1
NR1I3	Nuclear Receptor Subfamily 1 Group I Member 3	Protein Coding	45	GC01M161229	22.04448	https://www.genecards.org/cgi-bin/carddisp.pl?gene=NR1I3
BICC1	BicC Family RNA Binding Protein 1	Protein Coding	37	GC10P058513	22.02353	https://www.genecards.org/cgi-bin/carddisp.pl?gene=BICC1
TCF7L2	Transcription Factor 7 Like 2	Protein Coding	46	GC10P112950	22.01611	https://www.genecards.org/cgi-bin/carddisp.pl?gene=TCF7L2
FADD	Fas Associated Via Death Domain	Protein Coding	48	GC11P070203	21.99869	https://www.genecards.org/cgi-bin/carddisp.pl?gene=FADD
NOTCH3	Notch Receptor 3	Protein Coding	50	GC19M015159	21.98598	https://www.genecards.org/cgi-bin/carddisp.pl?gene=NOTCH3
HLA-C	Major Histocompatibility Complex, Class I, C	Protein Coding	45	GC06M046831	21.98008	https://www.genecards.org/cgi-bin/carddisp.pl?gene=HLA-C
UGT1A10	UDP Glucuronosyltransferase Family 1 Member A10	Protein Coding	41	GC02P233636	21.94478	https://www.genecards.org/cgi-bin/carddisp.pl?gene=UGT1A10
AFG3L2	AFG3 Like Matrix AAA Peptidase Subunit 2	Protein Coding	45	GC18M012328	21.93707	https://www.genecards.org/cgi-bin/carddisp.pl?gene=AFG3L2
SCARB1	Scavenger Receptor Class B Member 1	Protein Coding	46	GC12M124776	21.90098	https://www.genecards.org/cgi-bin/carddisp.pl?gene=SCARB1

CHIT1	Chitinase 1	Protein Coding	44	GC01M203181	21.90074	https://www.genecards.org/cgi-bin/carddisp.pl?gene=CHIT1
PKD2L1	Polycystin 2 Like 1, Transient Receptor Potential Cation Channel	Protein Coding	42	GC10M100288	21.89248	https://www.genecards.org/cgi-bin/carddisp.pl?gene=PKD2L1
GUCY2D	Guanylate Cyclase 2D, Retinal	Protein Coding	44	GC17P008002	21.86941	https://www.genecards.org/cgi-bin/carddisp.pl?gene=GUCY2D
APOA2	Apolipoprotein A2	Protein Coding	44	GC01M161222	21.85445	https://www.genecards.org/cgi-bin/carddisp.pl?gene=APOA2
SBF1	SET Binding Factor 1	Protein Coding	42	GC22M050445	21.82764	https://www.genecards.org/cgi-bin/carddisp.pl?gene=SBF1
IFNB1	Interferon Beta 1	Protein Coding	42	GC09M021077	21.81886	https://www.genecards.org/cgi-bin/carddisp.pl?gene=IFNB1
CASP1	Caspase 1	Protein Coding	51	GC11M105025	21.81465	https://www.genecards.org/cgi-bin/carddisp.pl?gene=CASP1
RPE65	Retinoid Isomerohydrolase RPE65	Protein Coding	45	GC01M068428	21.80959	https://www.genecards.org/cgi-bin/carddisp.pl?gene=RPE65
LRP1	LDL Receptor Related Protein 1	Protein Coding	48	GC12P057128	21.8083	https://www.genecards.org/cgi-bin/carddisp.pl?gene=LRP1
HBA1	Hemoglobin Subunit Alpha 1	Protein Coding	43	GC16P005490	21.80419	https://www.genecards.org/cgi-bin/carddisp.pl?gene=HBA1
PPIG	Peptidylprolyl Isomerase G	Protein Coding	43	GC02P169584	21.77139	https://www.genecards.org/cgi-bin/carddisp.pl?gene=PPIG
ALAD	Aminolevulinate Dehydratase	Protein Coding	47	GC09M113386	21.76044	https://www.genecards.org/cgi-bin/carddisp.pl?gene=ALAD
CASP9	Caspase 9	Protein Coding	48	GC01M015491	21.74611	https://www.genecards.org/cgi-bin/carddisp.pl?gene=CASP9
NDUFS7	NADH:Ubiquinone Oxidoreductase Core Subunit S7	Protein Coding	47	GC19P001635	21.72116	https://www.genecards.org/cgi-bin/carddisp.pl?gene=NDUFS7
CDK4	Cyclin Dependent Kinase 4	Protein Coding	54	GC12M057743	21.71355	https://www.genecards.org/cgi-bin/carddisp.pl?gene=CDK4
TCTN2	Tectonic Family Member 2	Protein Coding	39	GC12P123671	21.71339	https://www.genecards.org/cgi-bin/carddisp.pl?gene=TCTN2
CSF2	Colony Stimulating Factor 2	Protein Coding	44	GC05P132073	21.71247	https://www.genecards.org/cgi-bin/carddisp.pl?gene=CSF2
SMAD6	SMAD Family Member 6	Protein Coding	47	GC15P066702	21.67286	https://www.genecards.org/cgi-bin/carddisp.pl?gene=SMAD6
CLCN7	Chloride Voltage-Gated Channel 7	Protein Coding	46	GC16M001444	21.67075	https://www.genecards.org/cgi-bin/carddisp.pl?gene=CLCN7
AGPS	Alkylglycerone Phosphate Synthase	Protein Coding	44	GC02P177392	21.66693	https://www.genecards.org/cgi-bin/carddisp.pl?gene=AGPS

SP140	SP140 Nuclear Body Protein	Protein Coding	38	GC02P230203	21.65835	https://www.genecards.org/cgi-bin/carddisp.pl?gene=SP140
IDH2	Isocitrate Dehydrogenase (NADP(+)) 2	Protein Coding	52	GC15M090083	21.64223	https://www.genecards.org/cgi-bin/carddisp.pl?gene=IDH2
CTNS	Cystinosin, Lysosomal Cystine Transporter	Protein Coding	45	GC17P003636	21.61856	https://www.genecards.org/cgi-bin/carddisp.pl?gene=CTNS
AKR1B1	Aldo-Keto Reductase Family 1 Member B	Protein Coding	48	GC07M134442	21.61324	https://www.genecards.org/cgi-bin/carddisp.pl?gene=AKR1B1
COG2	Component Of Oligomeric Golgi Complex 2	Protein Coding	41	GC01P230642	21.60097	https://www.genecards.org/cgi-bin/carddisp.pl?gene=COG2
RIPK1	Receptor Interacting Serine/Threonine Kinase 1	Protein Coding	50	GC06P003073	21.59178	https://www.genecards.org/cgi-bin/carddisp.pl?gene=RIPK1
FLG	Filaggrin	Protein Coding	40	GC01M152274	21.5907	https://www.genecards.org/cgi-bin/carddisp.pl?gene=FLG
COL1A2	Collagen Type I Alpha 2 Chain	Protein Coding	48	GC07P094394	21.56881	https://www.genecards.org/cgi-bin/carddisp.pl?gene=COL1A2
MIR183	MicroRNA 183	RNA Gene	18	GC07M129798	21.55194	https://www.genecards.org/cgi-bin/carddisp.pl?gene=MIR183
CRB1	Crumbs Cell Polarity Complex Component 1	Protein Coding	44	GC01P197170	21.55091	https://www.genecards.org/cgi-bin/carddisp.pl?gene=CRB1
NR5A2	Nuclear Receptor Subfamily 5 Group A Member 2	Protein Coding	47	GC01P199996	21.54375	https://www.genecards.org/cgi-bin/carddisp.pl?gene=NR5A2
MIR144	MicroRNA 144	RNA Gene	17	GC17M031086	21.51498	https://www.genecards.org/cgi-bin/carddisp.pl?gene=MIR144
MMUT	Methylmalonyl-CoA Mutase	Protein Coding	36	GC06M049430	21.50561	https://www.genecards.org/cgi-bin/carddisp.pl?gene=MMUT
UGT1A3	UDP Glucuronosyltransferase Family 1 Member A3	Protein Coding	39	GC02P233729	21.49641	https://www.genecards.org/cgi-bin/carddisp.pl?gene=UGT1A3
CD8A	CD8a Molecule	Protein Coding	47	GC02M086784	21.49401	https://www.genecards.org/cgi-bin/carddisp.pl?gene=CD8A
MIR205	MicroRNA 205	RNA Gene	20	GC01P209493	21.49146	https://www.genecards.org/cgi-bin/carddisp.pl?gene=MIR205
PPARD	Peroxisome Proliferator Activated Receptor Delta	Protein Coding	48	GC06P055324	21.48622	https://www.genecards.org/cgi-bin/carddisp.pl?gene=PPARD
CPOX	Coproporphyrinogen Oxidase	Protein Coding	44	GC03M098576	21.45315	https://www.genecards.org/cgi-bin/carddisp.pl?gene=CPOX

ARID1B	AT-Rich Interaction Domain 1B	Protein Coding	45	GC06P156777	21.44675	https://www.genecards.org/cgi-bin/carddisp.pl?gene=ARID1B
CYP2C8	Cytochrome P450 Family 2 Subfamily C Member 8	Protein Coding	49	GC10M095038	21.43716	https://www.genecards.org/cgi-bin/carddisp.pl?gene=CYP2C8
EDNRA	Endothelin Receptor Type A	Protein Coding	50	GC04P147480	21.39463	https://www.genecards.org/cgi-bin/carddisp.pl?gene=EDNRA
SBDS	SBDS Ribosome Maturation Factor	Protein Coding	43	GC07M066987	21.38954	https://www.genecards.org/cgi-bin/carddisp.pl?gene=SBDS
NDUFAF6	NADH:Ubiquinone Oxidoreductase Complex Assembly Factor 6	Protein Coding	35	GC08P094895	21.36459	https://www.genecards.org/cgi-bin/carddisp.pl?gene=NDUFAF6
PIK3CG	Phosphatidylinositol-4,5-Bisphosphate 3-Kinase Catalytic Subunit Gamma	Protein Coding	49	GC07P106865	21.32251	https://www.genecards.org/cgi-bin/carddisp.pl?gene=PIK3CG
MIR33A	MicroRNA 33a	RNA Gene	19	GC22P041900	21.31961	https://www.genecards.org/cgi-bin/carddisp.pl?gene=MIR33A
MST1	Macrophage Stimulating 1	Protein Coding	46	GC03M049683	21.31592	https://www.genecards.org/cgi-bin/carddisp.pl?gene=MST1
CPS1	Carbamoyl-Phosphate Synthase 1	Protein Coding	46	GC02P210477	21.30848	https://www.genecards.org/cgi-bin/carddisp.pl?gene=CPS1
TTN-AS1	TTN Antisense RNA 1	RNA Gene	16	GC02P178521	21.30586	https://www.genecards.org/cgi-bin/carddisp.pl?gene=TTN-AS1
SYP	Synaptophysin	Protein Coding	44	GC0XM049187	21.29917	https://www.genecards.org/cgi-bin/carddisp.pl?gene=SYP
PC	Pyruvate Carboxylase	Protein Coding	48	GC11M066848	21.28424	https://www.genecards.org/cgi-bin/carddisp.pl?gene=PC
NDUFV2	NADH:Ubiquinone Oxidoreductase Core Subunit V2	Protein Coding	45	GC18P009092	21.2616	https://www.genecards.org/cgi-bin/carddisp.pl?gene=NDUFV2
CD46	CD46 Molecule	Protein Coding	47	GC01P207752	21.24768	https://www.genecards.org/cgi-bin/carddisp.pl?gene=CD46
KITLG	KIT Ligand	Protein Coding	45	GC12M088492	21.23805	https://www.genecards.org/cgi-bin/carddisp.pl?gene=KITLG
ABCC6	ATP Binding Cassette Subfamily C Member 6	Protein Coding	47	GC16M016148	21.2084	https://www.genecards.org/cgi-bin/carddisp.pl?gene=ABCC6
CDKN2B	Cyclin Dependent Kinase Inhibitor 2B	Protein Coding	48	GC09M022002	21.20182	https://www.genecards.org/cgi-bin/carddisp.pl?gene=CDKN2B

GYG1	Glycogenin 1	Protein Coding	47	GC03P148991	21.19632	https://www.genecards.org/cgi-bin/carddisp.pl?gene=GYG1
COX6A1	Cytochrome C Oxidase Subunit 6A1	Protein Coding	45	GC12P120439	21.15471	https://www.genecards.org/cgi-bin/carddisp.pl?gene=COX6A1
CACNA1C	Calcium Voltage-Gated Channel Subunit Alpha1 C	Protein Coding	50	GC12P001970	21.14977	https://www.genecards.org/cgi-bin/carddisp.pl?gene=CACNA1C
ERN1	Endoplasmic Reticulum To Nucleus Signaling 1	Protein Coding	46	GC17M064039	21.14885	https://www.genecards.org/cgi-bin/carddisp.pl?gene=ERN1
LOX	Lysyl Oxidase	Protein Coding	45	GC05M122063	21.14855	https://www.genecards.org/cgi-bin/carddisp.pl?gene=LOX
IFNGR1	Interferon Gamma Receptor 1	Protein Coding	50	GC06M137197	21.12041	https://www.genecards.org/cgi-bin/carddisp.pl?gene=IFNGR1
GALNS	Galactosamine (N-Acetyl)-6-Sulfatase	Protein Coding	45	GC16M088813	21.07434	https://www.genecards.org/cgi-bin/carddisp.pl?gene=GALNS
MIR451A	MicroRNA 451a	RNA Gene	18	GC17M028861	21.06853	https://www.genecards.org/cgi-bin/carddisp.pl?gene=MIR451A
GLE1	GLE1 RNA Export Mediator	Protein Coding	41	GC09P128504	21.05136	https://www.genecards.org/cgi-bin/carddisp.pl?gene=GLE1
EIF2AK3	Eukaryotic Translation Initiation Factor 2 Alpha Kinase 3	Protein Coding	50	GC02M088556	21.03918	https://www.genecards.org/cgi-bin/carddisp.pl?gene=EIF2AK3
PLA2G4A	Phospholipase A2 Group IVA	Protein Coding	50	GC01P186798	21.03371	https://www.genecards.org/cgi-bin/carddisp.pl?gene=PLA2G4A
GSR	Glutathione-Disulfide Reductase	Protein Coding	50	GC08M030678	21.03178	https://www.genecards.org/cgi-bin/carddisp.pl?gene=GSR
CRYAA	Crystallin Alpha A	Protein Coding	45	GC21P043169	20.98034	https://www.genecards.org/cgi-bin/carddisp.pl?gene=CRYAA
CYP3A5	Cytochrome P450 Family 3 Subfamily A Member 5	Protein Coding	48	GC07M099648	20.96791	https://www.genecards.org/cgi-bin/carddisp.pl?gene=CYP3A5
B9D1	B9 Domain Containing 1	Protein Coding	37	GC17M019334	20.95131	https://www.genecards.org/cgi-bin/carddisp.pl?gene=B9D1
PLA2G7	Phospholipase A2 Group VII	Protein Coding	50	GC06M047382	20.93696	https://www.genecards.org/cgi-bin/carddisp.pl?gene=PLA2G7
NBN	Nibrin	Protein Coding	48	GC08M089933	20.93391	https://www.genecards.org/cgi-bin/carddisp.pl?gene=NBN
MIR30E	MicroRNA 30e	RNA Gene	21	GC01P040754	20.91616	https://www.genecards.org/cgi-bin/carddisp.pl?gene=MIR30E
TNNI3	Troponin I3, Cardiac Type	Protein Coding	50	GC19M055151	20.88725	https://www.genecards.org/cgi-bin/carddisp.pl?gene=TNNI3
IL2RB	Interleukin 2 Receptor Subunit Beta	Protein Coding	49	GC22M037125	20.87809	https://www.genecards.org/cgi-bin/carddisp.pl?gene=IL2RB
CDH2	Cadherin 2	Protein Coding	53	GC18M027950	20.8751	https://www.genecards.org/cgi-bin/carddisp.pl?gene=CDH2

ITGB3	Integrin Subunit Beta 3	Protein Coding	50	GC17P047254	20.85393	https://www.genecards.org/cgi-bin/carddisp.pl?gene=ITGB3
NDUFB9	NADH:Ubiquinone Oxidoreductase Subunit B9	Protein Coding	45	GC08P124539	20.8491	https://www.genecards.org/cgi-bin/carddisp.pl?gene=NDUFB9
SLC10A1	Solute Carrier Family 10 Member 1	Protein Coding	45	GC14M069775	20.82361	https://www.genecards.org/cgi-bin/carddisp.pl?gene=SLC10A1
CR2	Complement C3d Receptor 2	Protein Coding	45	GC01P207454	20.82146	https://www.genecards.org/cgi-bin/carddisp.pl?gene=CR2
SCO1	Synthesis Of Cytochrome C Oxidase 1	Protein Coding	47	GC17M010672	20.80253	https://www.genecards.org/cgi-bin/carddisp.pl?gene=SCO1
TNFAIP3	TNF Alpha Induced Protein 3	Protein Coding	48	GC06P137866	20.7878	https://www.genecards.org/cgi-bin/carddisp.pl?gene=TNFAIP3
DYRK1A	Dual Specificity Tyrosine Phosphorylation Regulated Kinase 1A	Protein Coding	51	GC21P037365	20.78073	https://www.genecards.org/cgi-bin/carddisp.pl?gene=DYRK1A
PAX6	Paired Box 6	Protein Coding	48	GC11M031784	20.77972	https://www.genecards.org/cgi-bin/carddisp.pl?gene=PAX6
CX3CR1	C-X3-C Motif Chemokine Receptor 1	Protein Coding	45	GC03M039279	20.74883	https://www.genecards.org/cgi-bin/carddisp.pl?gene=CX3CR1
PECAM1	Platelet And Endothelial Cell Adhesion Molecule 1	Protein Coding	41	GC17M064319	20.73357	https://www.genecards.org/cgi-bin/carddisp.pl?gene=PECAM1
MYD88	MYD88 Innate Immune Signal Transduction Adaptor	Protein Coding	50	GC03P038139	20.71233	https://www.genecards.org/cgi-bin/carddisp.pl?gene=MYD88
FGF19	Fibroblast Growth Factor 19	Protein Coding	44	GC11M070382	20.71029	https://www.genecards.org/cgi-bin/carddisp.pl?gene=FGF19
CEBPB	CCAAT Enhancer Binding Protein Beta	Protein Coding	44	GC20P050190	20.70691	https://www.genecards.org/cgi-bin/carddisp.pl?gene=CEBPB
BCL2L1	BCL2 Like 1	Protein Coding	47	GC20M031664	20.70552	https://www.genecards.org/cgi-bin/carddisp.pl?gene=BCL2L1
SLC18A2	Solute Carrier Family 18 Member A2	Protein Coding	48	GC10P117241	20.69474	https://www.genecards.org/cgi-bin/carddisp.pl?gene=SLC18A2
RUNX2	RUNX Family Transcription Factor 2	Protein Coding	48	GC06P055427	20.67907	https://www.genecards.org/cgi-bin/carddisp.pl?gene=RUNX2
IL18BP	Interleukin 18 Binding Protein	Protein Coding	40	GC11P071998	20.65721	https://www.genecards.org/cgi-bin/carddisp.pl?gene=IL18BP
ALDH3A1	Aldehyde Dehydrogenase 3 Family Member A1	Protein Coding	44	GC17M019737	20.61052	https://www.genecards.org/cgi-bin/carddisp.pl?gene=ALDH3A1

FLT3	Fms Related Receptor Tyrosine Kinase 3	Protein Coding	53	GC13M028003	20.57567	https://www.genecards.org/cgi-bin/carddisp.pl?gene=FLT3
LGALS3	Galectin 3	Protein Coding	45	GC14P055124	20.56834	https://www.genecards.org/cgi-bin/carddisp.pl?gene=LGALS3
ASL	Argininosuccinate Lyase	Protein Coding	47	GC07P066075	20.55681	https://www.genecards.org/cgi-bin/carddisp.pl?gene=ASL
RPS6KB1	Ribosomal Protein S6 Kinase B1	Protein Coding	50	GC17P059893	20.55362	https://www.genecards.org/cgi-bin/carddisp.pl?gene=RPS6KB1
IL7R	Interleukin 7 Receptor	Protein Coding	47	GC05P035852	20.5457	https://www.genecards.org/cgi-bin/carddisp.pl?gene=IL7R
CDH23	Cadherin Related 23	Protein Coding	43	GC10P071396	20.53548	https://www.genecards.org/cgi-bin/carddisp.pl?gene=CDH23
FYN	FYN Proto-Oncogene, Src Family Tyrosine Kinase	Protein Coding	48	GC06M111660	20.52723	https://www.genecards.org/cgi-bin/carddisp.pl?gene=FYN
ENO2	Enolase 2	Protein Coding	48	GC12P006913	20.5169	https://www.genecards.org/cgi-bin/carddisp.pl?gene=ENO2
FFAR3	Free Fatty Acid Receptor 3	Protein Coding	38	GC19P040640	20.49132	https://www.genecards.org/cgi-bin/carddisp.pl?gene=FFAR3
NDUFS3	NADH:Ubiquinone Oxidoreductase Core Subunit S3	Protein Coding	48	GC11P047567	20.49101	https://www.genecards.org/cgi-bin/carddisp.pl?gene=NDUFS3
TIMP2	TIMP Metallopeptidase Inhibitor 2	Protein Coding	44	GC17M078852	20.49062	https://www.genecards.org/cgi-bin/carddisp.pl?gene=TIMP2
IL21	Interleukin 21	Protein Coding	43	GC04M122612	20.47485	https://www.genecards.org/cgi-bin/carddisp.pl?gene=IL21
PDYN	Prodynorphin	Protein Coding	44	GC20M001978	20.46194	https://www.genecards.org/cgi-bin/carddisp.pl?gene=PDYN
ACTC1	Actin Alpha Cardiac Muscle 1	Protein Coding	43	GC15M034788	20.45549	https://www.genecards.org/cgi-bin/carddisp.pl?gene=ACTC1
SMARCB1	SWI/SNF Related, Matrix Associated, Actin Dependent Regulator Of Chromatin, Subfamily B, Member 1	Protein Coding	46	GC22P023786	20.43601	https://www.genecards.org/cgi-bin/carddisp.pl?gene=SMARCB1
COL4A3	Collagen Type IV Alpha 3 Chain	Protein Coding	45	GC02P227164	20.41813	https://www.genecards.org/cgi-bin/carddisp.pl?gene=COL4A3
CHMP2B	Charged Multivesicular Body Protein 2B	Protein Coding	45	GC03P087227	20.4057	https://www.genecards.org/cgi-bin/carddisp.pl?gene=CHMP2B

SLC4A1	Solute Carrier Family 4 Member 1 (Diego Blood Group)	Protein Coding	48	GC17M044355	20.39626	https://www.genecards.org/cgi-bin/carddisp.pl?gene=SLC4A1
FLNC	Filamin C	Protein Coding	45	GC07P128830	20.38033	https://www.genecards.org/cgi-bin/carddisp.pl?gene=FLNC
MIRLET7E	MicroRNA Let-7e	RNA Gene	20	GC19P051718	20.36852	https://www.genecards.org/cgi-bin/carddisp.pl?gene=MIRLET7E
MEG3	Maternally Expressed 3	RNA Gene	30	GC14P106296	20.36535	https://www.genecards.org/cgi-bin/carddisp.pl?gene=MEG3
PARP1	Poly(ADP-Ribose) Polymerase 1	Protein Coding	50	GC01M226360	20.35945	https://www.genecards.org/cgi-bin/carddisp.pl?gene=PARP1
NPM1	Nucleophosmin 1	Protein Coding	50	GC05P171387	20.34415	https://www.genecards.org/cgi-bin/carddisp.pl?gene=NPM1
CR1	Complement C3b/C4b Receptor 1 (Knops Blood Group)	Protein Coding	45	GC01P207496	20.33232	https://www.genecards.org/cgi-bin/carddisp.pl?gene=CR1
NCAM1	Neural Cell Adhesion Molecule 1	Protein Coding	47	GC11P112961	20.31113	https://www.genecards.org/cgi-bin/carddisp.pl?gene=NCAM1
ABCA7	ATP Binding Cassette Subfamily A Member 7	Protein Coding	44	GC19P001040	20.30239	https://www.genecards.org/cgi-bin/carddisp.pl?gene=ABCA7
COL3A1	Collagen Type III Alpha 1 Chain	Protein Coding	47	GC02P188974	20.26769	https://www.genecards.org/cgi-bin/carddisp.pl?gene=COL3A1
CHGA	Chromogranin A	Protein Coding	43	GC14P092925	20.25076	https://www.genecards.org/cgi-bin/carddisp.pl?gene=CHGA
DNM1L	Dynamin 1 Like	Protein Coding	48	GC12P032679	20.20063	https://www.genecards.org/cgi-bin/carddisp.pl?gene=DNM1L
PALB2	Partner And Localizer Of BRCA2	Protein Coding	43	GC16M023603	20.19271	https://www.genecards.org/cgi-bin/carddisp.pl?gene=PALB2
CCL3	C-C Motif Chemokine Ligand 3	Protein Coding	40	GC17M036088	20.1804	https://www.genecards.org/cgi-bin/carddisp.pl?gene=CCL3
F13A1	Coagulation Factor XIII A Chain	Protein Coding	47	GC06M006144	20.17239	https://www.genecards.org/cgi-bin/carddisp.pl?gene=F13A1
ZAP70	Zeta Chain Of T Cell Receptor Associated Protein Kinase 70	Protein Coding	52	GC02P097696	20.15912	https://www.genecards.org/cgi-bin/carddisp.pl?gene=ZAP70
ICOSLG	Inducible T Cell Costimulator Ligand	Protein Coding	39	GC21M044222	20.159	https://www.genecards.org/cgi-bin/carddisp.pl?gene=ICOSLG
SERPINF2	Serpin Family F Member 2	Protein Coding	44	GC17P001742	20.15118	https://www.genecards.org/cgi-bin/carddisp.pl?gene=SERPINF2
MB	Myoglobin	Protein Coding	44	GC22M035606	20.14612	https://www.genecards.org/cgi-bin/carddisp.pl?gene=MB

ABCG2	ATP Binding Cassette Subfamily G Member 2 (Junior Blood Group)	Protein Coding	51	GC04M088090	20.13269	https://www.genecards.org/cgi-bin/carddisp.pl?gene=ABCG2
MIR9-1	MicroRNA 9-1	RNA Gene	20	GC01M156420	20.12142	https://www.genecards.org/cgi-bin/carddisp.pl?gene=MIR9-1
GC	GC Vitamin D Binding Protein	Protein Coding	44	GC04M071741	20.12044	https://www.genecards.org/cgi-bin/carddisp.pl?gene=GC
EIF4G1	Eukaryotic Translation Initiation Factor 4 Gamma 1	Protein Coding	47	GC03P184314	20.10441	https://www.genecards.org/cgi-bin/carddisp.pl?gene=EIF4G1
PHKA1	Phosphorylase Kinase Regulatory Subunit Alpha 1	Protein Coding	44	GC0XM072578	20.09604	https://www.genecards.org/cgi-bin/carddisp.pl?gene=PHKA1
ADHFE1	Alcohol Dehydrogenase Iron Containing 1	Protein Coding	38	GC08P066432	20.08615	https://www.genecards.org/cgi-bin/carddisp.pl?gene=ADHFE1
COPA	COPI Coat Complex Subunit Alpha	Protein Coding	43	GC01M160288	20.08255	https://www.genecards.org/cgi-bin/carddisp.pl?gene=COPA
PDX1	Pancreatic And Duodenal Homeobox 1	Protein Coding	48	GC13P027921	20.07712	https://www.genecards.org/cgi-bin/carddisp.pl?gene=PDX1
TNNT2	Troponin T2, Cardiac Type	Protein Coding	50	GC01M201359	20.04509	https://www.genecards.org/cgi-bin/carddisp.pl?gene=TNNT2
MIR204	MicroRNA 204	RNA Gene	21	GC09M070809	20.04273	https://www.genecards.org/cgi-bin/carddisp.pl?gene=MIR204
CD19	CD19 Molecule	Protein Coding	50	GC16P032270	20.03776	https://www.genecards.org/cgi-bin/carddisp.pl?gene=CD19
SLC1A2	Solute Carrier Family 1 Member 2	Protein Coding	50	GC11M035252	20.03393	https://www.genecards.org/cgi-bin/carddisp.pl?gene=SLC1A2
USH2A	Usherin	Protein Coding	38	GC01M215622	19.99977	https://www.genecards.org/cgi-bin/carddisp.pl?gene=USH2A
SORD	Sorbitol Dehydrogenase	Protein Coding	47	GC15P045023	19.99626	https://www.genecards.org/cgi-bin/carddisp.pl?gene=SORD
PGM1	Phosphoglucomutase 1	Protein Coding	49	GC01P063593	19.99465	https://www.genecards.org/cgi-bin/carddisp.pl?gene=PGM1
MYH11	Myosin Heavy Chain 11	Protein Coding	46	GC16M015704	19.99353	https://www.genecards.org/cgi-bin/carddisp.pl?gene=MYH11
TSPO	Translocator Protein	Protein Coding	44	GC22P043151	19.97203	https://www.genecards.org/cgi-bin/carddisp.pl?gene=TSPO
CSF1	Colony Stimulating Factor 1	Protein Coding	44	GC01P109911	19.95462	https://www.genecards.org/cgi-bin/carddisp.pl?gene=CSF1
RARB	Retinoic Acid Receptor Beta	Protein Coding	51	GC03P024830	19.95306	https://www.genecards.org/cgi-bin/carddisp.pl?gene=RARB

KCNH2	Potassium Voltage-Gated Channel Subfamily H Member 2	Protein Coding	50	GC07M150944	19.94983	https://www.genecards.org/cgi-bin/carddisp.pl?gene=KCNH2
ITGB1	Integrin Subunit Beta 1	Protein Coding	51	GC10M032890	19.94729	https://www.genecards.org/cgi-bin/carddisp.pl?gene=ITGB1
KNG1	Kininogen 1	Protein Coding	45	GC03P186717	19.92728	https://www.genecards.org/cgi-bin/carddisp.pl?gene=KNG1
GNAQ	G Protein Subunit Alpha Q	Protein Coding	50	GC09M077716	19.92713	https://www.genecards.org/cgi-bin/carddisp.pl?gene=GNAQ
CDK1	Cyclin Dependent Kinase 1	Protein Coding	47	GC10P060772	19.92036	https://www.genecards.org/cgi-bin/carddisp.pl?gene=CDK1
SCN4A	Sodium Voltage-Gated Channel Alpha Subunit 4	Protein Coding	45	GC17M063938	19.90688	https://www.genecards.org/cgi-bin/carddisp.pl?gene=SCN4A
SULT2A1	Sulfotransferase Family 2A Member 1	Protein Coding	45	GC19M047870	19.89643	https://www.genecards.org/cgi-bin/carddisp.pl?gene=SULT2A1
PINK1-AS	PINK1 Antisense RNA	RNA Gene	13	GC01M020642	19.8783	https://www.genecards.org/cgi-bin/carddisp.pl?gene=PINK1-AS
BBS4	Bardet-Biedl Syndrome 4	Protein Coding	42	GC15P072686	19.87511	https://www.genecards.org/cgi-bin/carddisp.pl?gene=BBS4
MIR429	MicroRNA 429	RNA Gene	19	GC01P002023	19.87298	https://www.genecards.org/cgi-bin/carddisp.pl?gene=MIR429
IKBKG	Inhibitor Of Nuclear Factor Kappa B Kinase Regulatory Subunit Gamma	Protein Coding	48	GC0XP154541	19.86198	https://www.genecards.org/cgi-bin/carddisp.pl?gene=IKBKG
MIR26A1	MicroRNA 26a-1	RNA Gene	20	GC03P037969	19.85808	https://www.genecards.org/cgi-bin/carddisp.pl?gene=MIR26A1
ANXA5	Annexin A5	Protein Coding	47	GC04M121667	19.85589	https://www.genecards.org/cgi-bin/carddisp.pl?gene=ANXA5
SBF2-AS1	SBF2 Antisense RNA 1	RNA Gene	16	GC11P009758	19.83401	https://www.genecards.org/cgi-bin/carddisp.pl?gene=SBF2-AS1
UROS	Uroporphyrinogen III Synthase	Protein Coding	43	GC10M125784	19.82992	https://www.genecards.org/cgi-bin/carddisp.pl?gene=UROS
TULP1	TUB Like Protein 1	Protein Coding	43	GC06M047024	19.82913	https://www.genecards.org/cgi-bin/carddisp.pl?gene=TULP1
YAP1	Yes1 Associated Transcriptional Regulator	Protein Coding	48	GC11P102110	19.82795	https://www.genecards.org/cgi-bin/carddisp.pl?gene=YAP1
RAG2	Recombination Activating 2	Protein Coding	43	GC11M036575	19.8225	https://www.genecards.org/cgi-bin/carddisp.pl?gene=RAG2
L1CAM	L1 Cell Adhesion Molecule	Protein Coding	47	GC0XM153864	19.81722	https://www.genecards.org/cgi-bin/carddisp.pl?gene=L1CAM

UQCRB	Ubiquinol-Cytochrome C Reductase Binding Protein	Protein Coding	44	GC08M096225	19.80413	https://www.genecards.org/cgi-bin/carddisp.pl?gene=UQCRB
RPS27A	Ribosomal Protein S27a	Protein Coding	44	GC02P055231	19.78513	https://www.genecards.org/cgi-bin/carddisp.pl?gene=RPS27A
NEB	Nebulin	Protein Coding	41	GC02M151485	19.77995	https://www.genecards.org/cgi-bin/carddisp.pl?gene=NEB
ERCC4	ERCC Excision Repair 4, Endonuclease Catalytic Subunit	Protein Coding	46	GC16P013920	19.76361	https://www.genecards.org/cgi-bin/carddisp.pl?gene=ERCC4
SPG7	SPG7 Matrix AAA Peptidase Subunit, Paraplegin	Protein Coding	44	GC16P089790	19.76343	https://www.genecards.org/cgi-bin/carddisp.pl?gene=SPG7
MIR483	MicroRNA 483	RNA Gene	18	GC11M002219	19.75251	https://www.genecards.org/cgi-bin/carddisp.pl?gene=MIR483
LIVAR	Liver Cell Viability Associated LncRNA	RNA Gene	10	GC18M070336	19.73477	https://www.genecards.org/cgi-bin/carddisp.pl?gene=LIVAR
PRPS1	Phosphoribosyl Pyrophosphate Synthetase 1	Protein Coding	47	GC0XP107628	19.71732	https://www.genecards.org/cgi-bin/carddisp.pl?gene=PRPS1
PNKP	Polynucleotide Kinase 3'-Phosphatase	Protein Coding	46	GC19M049861	19.70959	https://www.genecards.org/cgi-bin/carddisp.pl?gene=PNKP
EZH2	Enhancer Of Zeste 2 Polycomb Repressive Complex 2 Subunit	Protein Coding	54	GC07M148807	19.70585	https://www.genecards.org/cgi-bin/carddisp.pl?gene=EZH2
ALDOA	Aldolase, Fructose-Bisphosphate A	Protein Coding	49	GC16P030064	19.70424	https://www.genecards.org/cgi-bin/carddisp.pl?gene=ALDOA
RAG1	Recombination Activating 1	Protein Coding	46	GC11P036520	19.68536	https://www.genecards.org/cgi-bin/carddisp.pl?gene=RAG1
MKKS	MKKS Centrosomal Shuttling Protein	Protein Coding	40	GC20M010424	19.68426	https://www.genecards.org/cgi-bin/carddisp.pl?gene=MKKS
TRIM2	Tripartite Motif Containing 2	Protein Coding	41	GC04P153152	19.6751	https://www.genecards.org/cgi-bin/carddisp.pl?gene=TRIM2
MIR34C	MicroRNA 34c	RNA Gene	20	GC11P111591	19.65926	https://www.genecards.org/cgi-bin/carddisp.pl?gene=MIR34C
MFSD2A	Major Facilitator Superfamily Domain Containing 2A	Protein Coding	39	GC01P039955	19.64758	https://www.genecards.org/cgi-bin/carddisp.pl?gene=MFSD2A
MBP	Myelin Basic Protein	Protein Coding	45	GC18M076978	19.6423	https://www.genecards.org/cgi-bin/carddisp.pl?gene=MBP

GNMT	Glycine N-Methyltransferase	Protein Coding	46	GC06P042960	19.63456	https://www.genecards.org/cgi-bin/carddisp.pl?gene=GNMT
PDGFRA	Platelet Derived Growth Factor Receptor Alpha	Protein Coding	55	GC04P054229	19.63359	https://www.genecards.org/cgi-bin/carddisp.pl?gene=PDGFRA
DDC	Dopa Decarboxylase	Protein Coding	53	GC07M050458	19.62644	https://www.genecards.org/cgi-bin/carddisp.pl?gene=DDC
ACSL3	Acyl-CoA Synthetase Long Chain Family Member 3	Protein Coding	41	GC02P222860	19.61269	https://www.genecards.org/cgi-bin/carddisp.pl?gene=ACSL3
CHD7	Chromodomain Helicase DNA Binding Protein 7	Protein Coding	46	GC08P060678	19.59666	https://www.genecards.org/cgi-bin/carddisp.pl?gene=CHD7
CYP2U1	Cytochrome P450 Family 2 Subfamily U Member 1	Protein Coding	44	GC04P107931	19.59149	https://www.genecards.org/cgi-bin/carddisp.pl?gene=CYP2U1
CHUK	Component Of Inhibitor Of Nuclear Factor Kappa B Kinase Complex	Protein Coding	52	GC10M100188	19.56647	https://www.genecards.org/cgi-bin/carddisp.pl?gene=CHUK
PPA2	Inorganic Pyrophosphatase 2	Protein Coding	42	GC04M105369	19.56451	https://www.genecards.org/cgi-bin/carddisp.pl?gene=PPA2
HBG2	Hemoglobin Subunit Gamma 2	Protein Coding	43	GC11M005442	19.55492	https://www.genecards.org/cgi-bin/carddisp.pl?gene=HBG2
MKI67	Marker Of Proliferation Ki-67	Protein Coding	45	GC10M128096	19.5453	https://www.genecards.org/cgi-bin/carddisp.pl?gene=MKI67
FXN	Frataxin	Protein Coding	47	GC09P069035	19.51641	https://www.genecards.org/cgi-bin/carddisp.pl?gene=FXN
CCL11	C-C Motif Chemokine Ligand 11	Protein Coding	44	GC17P034285	19.51405	https://www.genecards.org/cgi-bin/carddisp.pl?gene=CCL11
PRKCA	Protein Kinase C Alpha	Protein Coding	52	GC17P066302	19.50887	https://www.genecards.org/cgi-bin/carddisp.pl?gene=PRKCA
RAC1	Rac Family Small GTPase 1	Protein Coding	50	GC07P006377	19.50507	https://www.genecards.org/cgi-bin/carddisp.pl?gene=RAC1
LRPPRC	Leucine Rich Pentatricopeptide Repeat Containing	Protein Coding	43	GC02M043850	19.50468	https://www.genecards.org/cgi-bin/carddisp.pl?gene=LRPPRC
RYR2	Ryanodine Receptor 2	Protein Coding	48	GC01P237042	19.48267	https://www.genecards.org/cgi-bin/carddisp.pl?gene=RYR2
ADCY10	Adenylate Cyclase 10	Protein Coding	45	GC01M167809	19.4616	https://www.genecards.org/cgi-bin/carddisp.pl?gene=ADCY10
PGR	Progesterone Receptor	Protein Coding	51	GC11M101030	19.45818	https://www.genecards.org/cgi-bin/carddisp.pl?gene=PGR

TPH1	Tryptophan Hydroxylase 1	Protein Coding	45	GC11M018040	19.42734	https://www.genecards.org/cgi-bin/carddisp.pl?gene=TPH1
SERPINA6	Serpin Family A Member 6	Protein Coding	44	GC14M095823	19.42633	https://www.genecards.org/cgi-bin/carddisp.pl?gene=SERPINA6
SCN1A	Sodium Voltage-Gated Channel Alpha Subunit 1	Protein Coding	47	GC02M165989	19.41956	https://www.genecards.org/cgi-bin/carddisp.pl?gene=SCN1A
NEU1	Neuraminidase 1	Protein Coding	45	GC06M031857	19.41008	https://www.genecards.org/cgi-bin/carddisp.pl?gene=NEU1
LDHA	Lactate Dehydrogenase A	Protein Coding	52	GC11P018394	19.39824	https://www.genecards.org/cgi-bin/carddisp.pl?gene=LDHA
BBS12	Bardet-Biedl Syndrome 12	Protein Coding	34	GC04P122702	19.37847	https://www.genecards.org/cgi-bin/carddisp.pl?gene=BBS12
FAAH2	Fatty Acid Amide Hydrolase 2	Protein Coding	37	GC0XP057121	19.3499	https://www.genecards.org/cgi-bin/carddisp.pl?gene=FAAH2
PMP2	Peripheral Myelin Protein 2	Protein Coding	43	GC08M081440	19.3472	https://www.genecards.org/cgi-bin/carddisp.pl?gene=PMP2
SELL	Selectin L	Protein Coding	43	GC01M169690	19.34113	https://www.genecards.org/cgi-bin/carddisp.pl?gene=SELL
MIR27B	MicroRNA 27b	RNA Gene	20	GC09P095097	19.33519	https://www.genecards.org/cgi-bin/carddisp.pl?gene=MIR27B
ITPR1	Inositol 1,4,5-Trisphosphate Receptor Type 1	Protein Coding	49	GC03P004486	19.32337	https://www.genecards.org/cgi-bin/carddisp.pl?gene=ITPR1
ITGB2	Integrin Subunit Beta 2	Protein Coding	51	GC21M044885	19.31633	https://www.genecards.org/cgi-bin/carddisp.pl?gene=ITGB2
RHOA	Ras Homolog Family Member A	Protein Coding	48	GC03M049359	19.31543	https://www.genecards.org/cgi-bin/carddisp.pl?gene=RHOA
UCP3	Uncoupling Protein 3	Protein Coding	44	GC11M074000	19.30833	https://www.genecards.org/cgi-bin/carddisp.pl?gene=UCP3
SCN2A	Sodium Voltage-Gated Channel Alpha Subunit 2	Protein Coding	48	GC02P165194	19.29945	https://www.genecards.org/cgi-bin/carddisp.pl?gene=SCN2A
PNPLA6	Patatin Like Phospholipase Domain Containing 6	Protein Coding	45	GC19P007534	19.26455	https://www.genecards.org/cgi-bin/carddisp.pl?gene=PNPLA6
SYK	Spleen Associated Tyrosine Kinase	Protein Coding	51	GC09P091373	19.26337	https://www.genecards.org/cgi-bin/carddisp.pl?gene=SYK
HSP90AA1	Heat Shock Protein 90 Alpha Family Class A Member 1	Protein Coding	48	GC14M102080	19.26155	https://www.genecards.org/cgi-bin/carddisp.pl?gene=HSP90AA1
MIR192	MicroRNA 192	RNA Gene	22	GC11M064891	19.24969	https://www.genecards.org/cgi-bin/carddisp.pl?gene=MIR192
SORL1	Sortilin Related Receptor 1	Protein Coding	45	GC11P121452	19.24726	https://www.genecards.org/cgi-bin/carddisp.pl?gene=SORL1

ERBB3	Erb-B2 Receptor Tyrosine Kinase 3	Protein Coding	54	GC12P056376	19.24438	https://www.genecards.org/cgi-bin/carddisp.pl?gene=ERBB3
EPRS1	Glutamyl-Prolyl-TRNA Synthetase 1	Protein Coding	37	GC01M219969	19.23979	https://www.genecards.org/cgi-bin/carddisp.pl?gene=EPRS1
IL15	Interleukin 15	Protein Coding	41	GC04P141636	19.22407	https://www.genecards.org/cgi-bin/carddisp.pl?gene=IL15
CYB5R3	Cytochrome B5 Reductase 3	Protein Coding	45	GC22M042617	19.22358	https://www.genecards.org/cgi-bin/carddisp.pl?gene=CYB5R3
SOX9	SRY-Box Transcription Factor 9	Protein Coding	48	GC17P072121	19.21017	https://www.genecards.org/cgi-bin/carddisp.pl?gene=SOX9
F10	Coagulation Factor X	Protein Coding	50	GC13P113122	19.20737	https://www.genecards.org/cgi-bin/carddisp.pl?gene=F10
MMP14	Matrix Metallopeptidase 14	Protein Coding	52	GC14P026302	19.17437	https://www.genecards.org/cgi-bin/carddisp.pl?gene=MMP14
ITGA2B	Integrin Subunit Alpha 2b	Protein Coding	51	GC17M044442	19.16889	https://www.genecards.org/cgi-bin/carddisp.pl?gene=ITGA2B
DKK1	Dickkopf WNT Signaling Pathway Inhibitor 1	Protein Coding	45	GC10P052314	19.16834	https://www.genecards.org/cgi-bin/carddisp.pl?gene=DKK1
WFS1	Wolframin ER Transmembrane Glycoprotein	Protein Coding	46	GC04P006260	19.16253	https://www.genecards.org/cgi-bin/carddisp.pl?gene=WFS1
TGFA	Transforming Growth Factor Alpha	Protein Coding	46	GC02M070447	19.1429	https://www.genecards.org/cgi-bin/carddisp.pl?gene=TGFA
ADAR	Adenosine Deaminase RNA Specific	Protein Coding	45	GC01M154582	19.1289	https://www.genecards.org/cgi-bin/carddisp.pl?gene=ADAR
PTGS1	Prostaglandin-Endoperoxide Synthase 1	Protein Coding	47	GC09P122370	19.1281	https://www.genecards.org/cgi-bin/carddisp.pl?gene=PTGS1
IDS	Iduronate 2-Sulfatase	Protein Coding	49	GC0XM149476	19.12573	https://www.genecards.org/cgi-bin/carddisp.pl?gene=IDS
POLR1C	RNA Polymerase I And III Subunit C	Protein Coding	44	GC06P055413	19.11817	https://www.genecards.org/cgi-bin/carddisp.pl?gene=POLR1C
BIRC5	Baculoviral IAP Repeat Containing 5	Protein Coding	47	GC17P078214	19.07029	https://www.genecards.org/cgi-bin/carddisp.pl?gene=BIRC5
DLAT	Dihydrolipoamide S-Acetyltransferase	Protein Coding	47	GC11P112024	19.02916	https://www.genecards.org/cgi-bin/carddisp.pl?gene=DLAT
CDKN1C	Cyclin Dependent Kinase Inhibitor 1C	Protein Coding	47	GC11M002894	19.02737	https://www.genecards.org/cgi-bin/carddisp.pl?gene=CDKN1C

CLCN1	Chloride Voltage-Gated Channel 1	Protein Coding	45	GC07P143316	19.02526	https://www.genecards.org/cgi-bin/carddisp.pl?gene=CLCN1
LTF	Lactotransferrin	Protein Coding	44	GC03M046435	18.99238	https://www.genecards.org/cgi-bin/carddisp.pl?gene=LTF
DNAH8	Dynein Axonemal Heavy Chain 8	Protein Coding	38	GC06P055369	18.96644	https://www.genecards.org/cgi-bin/carddisp.pl?gene=DNAH8
GALK1	Galactokinase 1	Protein Coding	48	GC17M075751	18.94658	https://www.genecards.org/cgi-bin/carddisp.pl?gene=GALK1
FLAD1	Flavin Adenine Dinucleotide Synthetase 1	Protein Coding	41	GC01P154983	18.92473	https://www.genecards.org/cgi-bin/carddisp.pl?gene=FLAD1
GNB1	G Protein Subunit Beta 1	Protein Coding	47	GC01M001785	18.91256	https://www.genecards.org/cgi-bin/carddisp.pl?gene=GNB1
PHOX2B	Paired Like Homeobox 2B	Protein Coding	44	GC04M041746	18.90218	https://www.genecards.org/cgi-bin/carddisp.pl?gene=PHOX2B
SLC22A4	Solute Carrier Family 22 Member 4	Protein Coding	44	GC05P132294	18.90059	https://www.genecards.org/cgi-bin/carddisp.pl?gene=SLC22A4
AGXT	Alanine--Glyoxylate And Serine--Pyruvate Aminotransferase	Protein Coding	46	GC02P240868	18.89698	https://www.genecards.org/cgi-bin/carddisp.pl?gene=AGXT
SOST	Sclerostin	Protein Coding	44	GC17M043753	18.88508	https://www.genecards.org/cgi-bin/carddisp.pl?gene=SOST
GOT2	Glutamic-Oxaloacetic Transaminase 2	Protein Coding	47	GC16M058707	18.87727	https://www.genecards.org/cgi-bin/carddisp.pl?gene=GOT2
HCRT	Hypocretin Neuropeptide Precursor	Protein Coding	41	GC17M042185	18.87497	https://www.genecards.org/cgi-bin/carddisp.pl?gene=HCRT
TALDO1	Transaldolase 1	Protein Coding	47	GC11P000775	18.86015	https://www.genecards.org/cgi-bin/carddisp.pl?gene=TALDO1
RARA	Retinoic Acid Receptor Alpha	Protein Coding	51	GC17P040309	18.84603	https://www.genecards.org/cgi-bin/carddisp.pl?gene=RARA
AGER	Advanced Glycosylation End-Product Specific Receptor	Protein Coding	45	GC06M032180	18.83654	https://www.genecards.org/cgi-bin/carddisp.pl?gene=AGER
NSD1	Nuclear Receptor Binding SET Domain Protein 1	Protein Coding	44	GC05P177134	18.8358	https://www.genecards.org/cgi-bin/carddisp.pl?gene=NSD1
FMR1	FMRP Translational Regulator 1	Protein Coding	45	GC0XP147925	18.83535	https://www.genecards.org/cgi-bin/carddisp.pl?gene=FMR1
GAL	Galanin And GMAP Prepropeptide	Protein Coding	45	GC11P068684	18.82381	https://www.genecards.org/cgi-bin/carddisp.pl?gene=GAL

C19orf12	Chromosome 19 Open Reading Frame 12	Protein Coding	37	GC19M029699	18.81699	https://www.genecards.org/cgi-bin/carddisp.pl?gene=C19orf12
GPX1	Glutathione Peroxidase 1	Protein Coding	48	GC03M049486	18.81093	https://www.genecards.org/cgi-bin/carddisp.pl?gene=GPX1
B9D2	B9 Domain Containing 2	Protein Coding	40	GC19M041354	18.80832	https://www.genecards.org/cgi-bin/carddisp.pl?gene=B9D2
LMNB1	Lamin B1	Protein Coding	48	GC05P126776	18.80138	https://www.genecards.org/cgi-bin/carddisp.pl?gene=LMNB1
AGK	Acylglycerol Kinase	Protein Coding	43	GC07P141551	18.77034	https://www.genecards.org/cgi-bin/carddisp.pl?gene=AGK
MIR151A	MicroRNA 151a	RNA Gene	18	GC08M140733	18.76171	https://www.genecards.org/cgi-bin/carddisp.pl?gene=MIR151A
TEK	TEK Receptor Tyrosine Kinase	Protein Coding	51	GC09P027109	18.74609	https://www.genecards.org/cgi-bin/carddisp.pl?gene=TEK
FTH1	Ferritin Heavy Chain 1	Protein Coding	51	GC11M061959	18.7335	https://www.genecards.org/cgi-bin/carddisp.pl?gene=FTH1
HSD11B1	Hydroxysteroid 11-Beta Dehydrogenase 1	Protein Coding	50	GC01P209686	18.72218	https://www.genecards.org/cgi-bin/carddisp.pl?gene=HSD11B1
NDUFAF2	NADH:Ubiquinone Oxidoreductase Complex Assembly Factor 2	Protein Coding	41	GC05P060945	18.71305	https://www.genecards.org/cgi-bin/carddisp.pl?gene=NDUFAF2
CFTR-AS1	CFTR Antisense RNA 1	RNA Gene	10	GC07M117561	18.71105	https://www.genecards.org/cgi-bin/carddisp.pl?gene=CFTR-AS1
EPHX2	Epoxide Hydrolase 2	Protein Coding	48	GC08P027490	18.69537	https://www.genecards.org/cgi-bin/carddisp.pl?gene=EPHX2
ESR2	Estrogen Receptor 2	Protein Coding	50	GC14M064084	18.67936	https://www.genecards.org/cgi-bin/carddisp.pl?gene=ESR2
XRCC1	X-Ray Repair Cross Complementing 1	Protein Coding	44	GC19M043543	18.67051	https://www.genecards.org/cgi-bin/carddisp.pl?gene=XRCC1
GALE	UDP-Galactose-4-Epimerase	Protein Coding	45	GC01M023795	18.66944	https://www.genecards.org/cgi-bin/carddisp.pl?gene=GALE
SMC1A	Structural Maintenance Of Chromosomes 1A	Protein Coding	47	GC0XM053374	18.66214	https://www.genecards.org/cgi-bin/carddisp.pl?gene=SMC1A
SAA1	Serum Amyloid A1	Protein Coding	43	GC11P018267	18.66112	https://www.genecards.org/cgi-bin/carddisp.pl?gene=SAA1
NRG1	Neuregulin 1	Protein Coding	47	GC08P031639	18.65294	https://www.genecards.org/cgi-bin/carddisp.pl?gene=NRG1
ZMPSTE24	Zinc Metallopeptidase STE24	Protein Coding	43	GC01P040258	18.63879	https://www.genecards.org/cgi-bin/carddisp.pl?gene=ZMPSTE24
CFHR5	Complement Factor H Related 5	Protein Coding	40	GC01P196977	18.63254	https://www.genecards.org/cgi-bin/carddisp.pl?gene=CFHR5

SCN9A	Sodium Voltage-Gated Channel Alpha Subunit 9	Protein Coding	48	GC02M166195	18.63041	https://www.genecards.org/cgi-bin/carddisp.pl?gene=SCN9A
MIR98	MicroRNA 98	RNA Gene	17	GC0XM053595	18.60065	https://www.genecards.org/cgi-bin/carddisp.pl?gene=MIR98
MIR373	MicroRNA 373	RNA Gene	20	GC19P056408	18.59049	https://www.genecards.org/cgi-bin/carddisp.pl?gene=MIR373
HMGB1	High Mobility Group Box 1	Protein Coding	45	GC13M030456	18.58014	https://www.genecards.org/cgi-bin/carddisp.pl?gene=HMGB1
FGFR4	Fibroblast Growth Factor Receptor 4	Protein Coding	52	GC05P177086	18.52946	https://www.genecards.org/cgi-bin/carddisp.pl?gene=FGFR4
DNAJC13	DnaJ Heat Shock Protein Family (Hsp40) Member C13	Protein Coding	38	GC03P132417	18.52785	https://www.genecards.org/cgi-bin/carddisp.pl?gene=DNAJC13
NDUFA1	NADH:Ubiquinone Oxidoreductase Subunit A1	Protein Coding	45	GC0XP119871	18.52526	https://www.genecards.org/cgi-bin/carddisp.pl?gene=NDUFA1
CRHR1	Corticotropin Releasing Hormone Receptor 1	Protein Coding	46	GC17P045784	18.52266	https://www.genecards.org/cgi-bin/carddisp.pl?gene=CRHR1
CTRC	Chymotrypsin C	Protein Coding	44	GC01P015438	18.50513	https://www.genecards.org/cgi-bin/carddisp.pl?gene=CTRC
REST	RE1 Silencing Transcription Factor	Protein Coding	45	GC04P056907	18.49587	https://www.genecards.org/cgi-bin/carddisp.pl?gene=REST
PITX2	Paired Like Homeodomain 2	Protein Coding	48	GC04M110617	18.4945	https://www.genecards.org/cgi-bin/carddisp.pl?gene=PITX2
MAPK10	Mitogen-Activated Protein Kinase 10	Protein Coding	52	GC04M085990	18.47836	https://www.genecards.org/cgi-bin/carddisp.pl?gene=MAPK10
GAD1	Glutamate Decarboxylase 1	Protein Coding	52	GC02P170813	18.4657	https://www.genecards.org/cgi-bin/carddisp.pl?gene=GAD1
WNT1	Wnt Family Member 1	Protein Coding	46	GC12P049053	18.45898	https://www.genecards.org/cgi-bin/carddisp.pl?gene=WNT1
CDK2	Cyclin Dependent Kinase 2	Protein Coding	52	GC12P055966	18.4469	https://www.genecards.org/cgi-bin/carddisp.pl?gene=CDK2
MYLK	Myosin Light Chain Kinase	Protein Coding	53	GC03M123610	18.44142	https://www.genecards.org/cgi-bin/carddisp.pl?gene=MYLK
BTD	Biotinidase	Protein Coding	45	GC03P015967	18.43469	https://www.genecards.org/cgi-bin/carddisp.pl?gene=BTD
NPC1L1	NPC1 Like Intracellular Cholesterol Transporter 1	Protein Coding	44	GC07M044512	18.41949	https://www.genecards.org/cgi-bin/carddisp.pl?gene=NPC1L1
S100A9	S100 Calcium Binding Protein A9	Protein Coding	43	GC01P153357	18.40723	https://www.genecards.org/cgi-bin/carddisp.pl?gene=S100A9

CIITA	Class II Major Histocompatibility Complex Transactivator	Protein Coding	45	GC16P010879	18.406	https://www.genecards.org/cgi-bin/carddisp.pl?gene=CIITA
SDCCAG8	SHH Signaling And Ciliogenesis Regulator SDCCAG8	Protein Coding	43	GC01P243255	18.3744	https://www.genecards.org/cgi-bin/carddisp.pl?gene=SDCCAG8
CEACAM5	CEA Cell Adhesion Molecule 5	Protein Coding	42	GC19P041709	18.37294	https://www.genecards.org/cgi-bin/carddisp.pl?gene=CEACAM5
IL3	Interleukin 3	Protein Coding	44	GC05P132060	18.36293	https://www.genecards.org/cgi-bin/carddisp.pl?gene=IL3
ANGPT2	Angiopoietin 2	Protein Coding	46	GC08M006499	18.36282	https://www.genecards.org/cgi-bin/carddisp.pl?gene=ANGPT2
MIR214	MicroRNA 214	RNA Gene	20	GC01M172234	18.35883	https://www.genecards.org/cgi-bin/carddisp.pl?gene=MIR214
LRRC56	Leucine Rich Repeat Containing 56	Protein Coding	36	GC11P000518	18.32882	https://www.genecards.org/cgi-bin/carddisp.pl?gene=LRRC56
MIR30D	MicroRNA 30d	RNA Gene	17	GC08M134804	18.32839	https://www.genecards.org/cgi-bin/carddisp.pl?gene=MIR30D
MMP13	Matrix Metallopeptidase 13	Protein Coding	51	GC11M102942	18.32537	https://www.genecards.org/cgi-bin/carddisp.pl?gene=MMP13
RAD51C	RAD51 Paralog C	Protein Coding	44	GC17P058692	18.31807	https://www.genecards.org/cgi-bin/carddisp.pl?gene=RAD51C
GAST	Gastrin	Protein Coding	40	GC17P041712	18.31575	https://www.genecards.org/cgi-bin/carddisp.pl?gene=GAST
RELN	Reelin	Protein Coding	44	GC07M103471	18.31478	https://www.genecards.org/cgi-bin/carddisp.pl?gene=RELN
SETX	Senataxin	Protein Coding	41	GC09M132261	18.29539	https://www.genecards.org/cgi-bin/carddisp.pl?gene=SETX
SMN2	Survival Of Motor Neuron 2, Centromeric	Protein Coding	43	GC05P070049	18.29088	https://www.genecards.org/cgi-bin/carddisp.pl?gene=SMN2
NGLY1	N-Glycanase 1	Protein Coding	45	GC03M025718	18.28975	https://www.genecards.org/cgi-bin/carddisp.pl?gene=NGLY1
IRF1	Interferon Regulatory Factor 1	Protein Coding	47	GC05M132440	18.28387	https://www.genecards.org/cgi-bin/carddisp.pl?gene=IRF1
AARS2	Alanyl-TRNA Synthetase 2, Mitochondrial	Protein Coding	43	GC06M044297	18.27469	https://www.genecards.org/cgi-bin/carddisp.pl?gene=AARS2
C4B	Complement C4B (Chido Blood Group)	Protein Coding	44	GC06P032014	18.26848	https://www.genecards.org/cgi-bin/carddisp.pl?gene=C4B
GLI3	GLI Family Zinc Finger 3	Protein Coding	50	GC07M041960	18.24593	https://www.genecards.org/cgi-bin/carddisp.pl?gene=GLI3
CACNA1S	Calcium Voltage-Gated Channel Subunit Alpha1 S	Protein Coding	49	GC01M201008	18.2353	https://www.genecards.org/cgi-bin/carddisp.pl?gene=CACNA1S

CHCHD10	Coiled-Coil-Helix-Coiled-Coil-Helix Domain Containing 10	Protein Coding	40	GC22M023765	18.2297	https://www.genecards.org/cgi-bin/carddisp.pl?gene=CHCHD10
CACNA1F	Calcium Voltage-Gated Channel Subunit Alpha1 F	Protein Coding	45	GC0XM049205	18.22608	https://www.genecards.org/cgi-bin/carddisp.pl?gene=CACNA1F
SKIV2L	Ski2 Like RNA Helicase	Protein Coding	44	GC06P055223	18.22394	https://www.genecards.org/cgi-bin/carddisp.pl?gene=SKIV2L
STXBP1	Syntaxin Binding Protein 1	Protein Coding	49	GC09P127582	18.22083	https://www.genecards.org/cgi-bin/carddisp.pl?gene=STXBP1
KHK	Ketohexokinase	Protein Coding	45	GC02P027086	18.21322	https://www.genecards.org/cgi-bin/carddisp.pl?gene=KHK
NOS1	Nitric Oxide Synthase 1	Protein Coding	50	GC12M117208	18.19965	https://www.genecards.org/cgi-bin/carddisp.pl?gene=NOS1
BTK	Bruton Tyrosine Kinase	Protein Coding	54	GC0XM101349	18.19194	https://www.genecards.org/cgi-bin/carddisp.pl?gene=BTK
SIRT3	Sirtuin 3	Protein Coding	48	GC11M000215	18.18283	https://www.genecards.org/cgi-bin/carddisp.pl?gene=SIRT3
MIR96	MicroRNA 96	RNA Gene	20	GC07M129774	18.18232	https://www.genecards.org/cgi-bin/carddisp.pl?gene=MIR96
WDR45	WD Repeat Domain 45	Protein Coding	41	GC0XM049074	18.17897	https://www.genecards.org/cgi-bin/carddisp.pl?gene=WDR45
CD27	CD27 Molecule	Protein Coding	45	GC12P011824	18.15331	https://www.genecards.org/cgi-bin/carddisp.pl?gene=CD27
AKR1C4	Aldo-Keto Reductase Family 1 Member C4	Protein Coding	48	GC10P005195	18.15305	https://www.genecards.org/cgi-bin/carddisp.pl?gene=AKR1C4
CLDN1	Claudin 1	Protein Coding	48	GC03M190305	18.14766	https://www.genecards.org/cgi-bin/carddisp.pl?gene=CLDN1
AHR	Aryl Hydrocarbon Receptor	Protein Coding	49	GC07P016916	18.14008	https://www.genecards.org/cgi-bin/carddisp.pl?gene=AHR
PLCB1	Phospholipase C Beta 1	Protein Coding	49	GC20P008061	18.10563	https://www.genecards.org/cgi-bin/carddisp.pl?gene=PLCB1
PPOX	Protoporphyrinogen Oxidase	Protein Coding	44	GC01P161184	18.09037	https://www.genecards.org/cgi-bin/carddisp.pl?gene=PPOX
UCP1	Uncoupling Protein 1	Protein Coding	45	GC04M140559	18.08976	https://www.genecards.org/cgi-bin/carddisp.pl?gene=UCP1
HTR1A	5-Hydroxytryptamine Receptor 1A	Protein Coding	48	GC05M063960	18.08189	https://www.genecards.org/cgi-bin/carddisp.pl?gene=HTR1A
ADK	Adenosine Kinase	Protein Coding	52	GC10P074152	18.07587	https://www.genecards.org/cgi-bin/carddisp.pl?gene=ADK
ARL13B	ADP Ribosylation Factor Like GTPase 13B	Protein Coding	38	GC03P093980	18.07421	https://www.genecards.org/cgi-bin/carddisp.pl?gene=ARL13B
TMPRSS6	Transmembrane Serine Protease 6	Protein Coding	44	GC22M037066	18.06333	https://www.genecards.org/cgi-bin/carddisp.pl?gene=TMPRSS6

IKZF1	IKAROS Family Zinc Finger 1	Protein Coding	48	GC07P050303	18.0478	https://www.genecards.org/cgi-bin/carddisp.pl?gene=IKZF1
VIP	Vasoactive Intestinal Peptide	Protein Coding	45	GC06P152750	18.04681	https://www.genecards.org/cgi-bin/carddisp.pl?gene=VIP
CD274	CD274 Molecule	Protein Coding	45	GC09P005450	18.04519	https://www.genecards.org/cgi-bin/carddisp.pl?gene=CD274
ANO10	Anoctamin 10	Protein Coding	41	GC03M043355	18.04361	https://www.genecards.org/cgi-bin/carddisp.pl?gene=ANO10
SMAD2	SMAD Family Member 2	Protein Coding	48	GC18M047809	18.0278	https://www.genecards.org/cgi-bin/carddisp.pl?gene=SMAD2
GRK1	G Protein-Coupled Receptor Kinase 1	Protein Coding	43	GC13P113645	18.01824	https://www.genecards.org/cgi-bin/carddisp.pl?gene=GRK1
NDUFA13	NADH:Ubiquinone Oxidoreductase Subunit A13	Protein Coding	45	GC19P019515	18.01547	https://www.genecards.org/cgi-bin/carddisp.pl?gene=NDUFA13
ITGAL	Integrin Subunit Alpha L	Protein Coding	47	GC16P030472	18.00021	https://www.genecards.org/cgi-bin/carddisp.pl?gene=ITGAL
MIR150	MicroRNA 150	RNA Gene	22	GC19M049500	17.99328	https://www.genecards.org/cgi-bin/carddisp.pl?gene=MIR150
FKTN	Fukutin	Protein Coding	40	GC09P105558	17.9832	https://www.genecards.org/cgi-bin/carddisp.pl?gene=FKTN
SHC1	SHC Adaptor Protein 1	Protein Coding	46	GC01M154962	17.97852	https://www.genecards.org/cgi-bin/carddisp.pl?gene=SHC1
FTO	FTO Alpha-Ketoglutarate Dependent Dioxygenase	Protein Coding	45	GC16P053737	17.97195	https://www.genecards.org/cgi-bin/carddisp.pl?gene=FTO
HPRT1	Hypoxanthine Phosphoribosyltransferase 1	Protein Coding	49	GC0XP134460	17.96763	https://www.genecards.org/cgi-bin/carddisp.pl?gene=HPRT1
PODXL	Podocalyxin Like	Protein Coding	43	GC07M131500	17.93267	https://www.genecards.org/cgi-bin/carddisp.pl?gene=PODXL
HDAC9	Histone Deacetylase 9	Protein Coding	47	GC07P018086	17.93263	https://www.genecards.org/cgi-bin/carddisp.pl?gene=HDAC9
DPYD	Dihydropyrimidine Dehydrogenase	Protein Coding	53	GC01M097015	17.93047	https://www.genecards.org/cgi-bin/carddisp.pl?gene=DPYD
PIK3CD	Phosphatidylinositol-4,5-Bisphosphate 3-Kinase Catalytic Subunit Delta	Protein Coding	54	GC01P009629	17.92698	https://www.genecards.org/cgi-bin/carddisp.pl?gene=PIK3CD
ABCA3	ATP Binding Cassette Subfamily A Member 3	Protein Coding	49	GC16M002275	17.92265	https://www.genecards.org/cgi-bin/carddisp.pl?gene=ABCA3
COL11A1	Collagen Type XI Alpha 1 Chain	Protein Coding	43	GC01M102876	17.91344	https://www.genecards.org/cgi-bin/carddisp.pl?gene=COL11A1
PTH	Parathyroid Hormone	Protein Coding	47	GC11M013492	17.90086	https://www.genecards.org/cgi-bin/carddisp.pl?gene=PTH

NR3C2	Nuclear Receptor Subfamily 3 Group C Member 2	Protein Coding	48	GC04M148078	17.8857	https://www.genecards.org/cgi-bin/carddisp.pl?gene=NR3C2
MMP7	Matrix Metallopeptidase 7	Protein Coding	49	GC11M102425	17.88334	https://www.genecards.org/cgi-bin/carddisp.pl?gene=MMP7
MITF	Melanocyte Inducing Transcription Factor	Protein Coding	48	GC03P069788	17.84032	https://www.genecards.org/cgi-bin/carddisp.pl?gene=MITF
ADRB3	Adrenoceptor Beta 3	Protein Coding	46	GC08M037962	17.83694	https://www.genecards.org/cgi-bin/carddisp.pl?gene=ADRB3
PSTPIP1	Proline-Serine-Threonine Phosphatase Interacting Protein 1	Protein Coding	46	GC15P076993	17.8344	https://www.genecards.org/cgi-bin/carddisp.pl?gene=PSTPIP1
CYP17A1	Cytochrome P450 Family 17 Subfamily A Member 1	Protein Coding	50	GC10M102830	17.82563	https://www.genecards.org/cgi-bin/carddisp.pl?gene=CYP17A1
HTR3A	5-Hydroxytryptamine Receptor 3A	Protein Coding	47	GC11P113974	17.82027	https://www.genecards.org/cgi-bin/carddisp.pl?gene=HTR3A
DDX3X	DEAD-Box Helicase 3 X-Linked	Protein Coding	48	GC0XP041333	17.81497	https://www.genecards.org/cgi-bin/carddisp.pl?gene=DDX3X
DNMT3B	DNA Methyltransferase 3 Beta	Protein Coding	51	GC20P032762	17.81486	https://www.genecards.org/cgi-bin/carddisp.pl?gene=DNMT3B
HK1	Hexokinase 1	Protein Coding	51	GC10P069269	17.80375	https://www.genecards.org/cgi-bin/carddisp.pl?gene=HK1
UGT1A5	UDP Glucuronosyltransferase Family 1 Member A5	Protein Coding	34	GC02P233712	17.79956	https://www.genecards.org/cgi-bin/carddisp.pl?gene=UGT1A5
TRPV1	Transient Receptor Potential Cation Channel Subfamily V Member 1	Protein Coding	47	GC17M003565	17.78683	https://www.genecards.org/cgi-bin/carddisp.pl?gene=TRPV1
SI	Sucrase-Isomaltase	Protein Coding	44	GC03M164978	17.77207	https://www.genecards.org/cgi-bin/carddisp.pl?gene=SI
LACC1	Laccase Domain Containing 1	Protein Coding	35	GC13P043879	17.77165	https://www.genecards.org/cgi-bin/carddisp.pl?gene=LACC1
BACE1	Beta-Secretase 1	Protein Coding	47	GC11M117285	17.76959	https://www.genecards.org/cgi-bin/carddisp.pl?gene=BACE1
GNB3	G Protein Subunit Beta 3	Protein Coding	48	GC12P006839	17.76518	https://www.genecards.org/cgi-bin/carddisp.pl?gene=GNB3

ETV6	ETS Variant Transcription Factor 6	Protein Coding	47	GC12P011649	17.73456	https://www.genecards.org/cgi-bin/carddisp.pl?gene=ETV6
SGPL1	Sphingosine-1-Phosphate Lyase 1	Protein Coding	45	GC10P070815	17.73369	https://www.genecards.org/cgi-bin/carddisp.pl?gene=SGPL1
ABCC3	ATP Binding Cassette Subfamily C Member 3	Protein Coding	47	GC17P050634	17.72028	https://www.genecards.org/cgi-bin/carddisp.pl?gene=ABCC3
SLC25A15	Solute Carrier Family 25 Member 15	Protein Coding	44	GC13P040789	17.70816	https://www.genecards.org/cgi-bin/carddisp.pl?gene=SLC25A15
LBP	Lipopolysaccharide Binding Protein	Protein Coding	44	GC20P038346	17.69856	https://www.genecards.org/cgi-bin/carddisp.pl?gene=LBP
ACTG1	Actin Gamma 1	Protein Coding	50	GC17M081509	17.69749	https://www.genecards.org/cgi-bin/carddisp.pl?gene=ACTG1
KRT14	Keratin 14	Protein Coding	48	GC17M041582	17.68269	https://www.genecards.org/cgi-bin/carddisp.pl?gene=KRT14
DVL2	Dishevelled Segment Polarity Protein 2	Protein Coding	47	GC17M007225	17.6784	https://www.genecards.org/cgi-bin/carddisp.pl?gene=DVL2
DECR1	2,4-Dienoyl-CoA Reductase 1	Protein Coding	42	GC08P090001	17.6648	https://www.genecards.org/cgi-bin/carddisp.pl?gene=DECR1
CES2	Carboxylesterase 2	Protein Coding	44	GC16P066934	17.66127	https://www.genecards.org/cgi-bin/carddisp.pl?gene=CES2
HLA-G	Major Histocompatibility Complex, Class I, G	Protein Coding	45	GC06P055150	17.65405	https://www.genecards.org/cgi-bin/carddisp.pl?gene=HLA-G
EVC2	EvC Ciliary Complex Subunit 2	Protein Coding	40	GC04M005534	17.65048	https://www.genecards.org/cgi-bin/carddisp.pl?gene=EVC2
MIR142	MicroRNA 142	RNA Gene	20	GC17M058331	17.63076	https://www.genecards.org/cgi-bin/carddisp.pl?gene=MIR142
CCR1	C-C Motif Chemokine Receptor 1	Protein Coding	45	GC03M046218	17.62608	https://www.genecards.org/cgi-bin/carddisp.pl?gene=CCR1
HSPA8	Heat Shock Protein Family A (Hsp70) Member 8	Protein Coding	48	GC11M123057	17.62122	https://www.genecards.org/cgi-bin/carddisp.pl?gene=HSPA8
CPT1B	Carnitine Palmitoyltransferase 1B	Protein Coding	45	GC22M050569	17.61032	https://www.genecards.org/cgi-bin/carddisp.pl?gene=CPT1B
SPAST	Spastin	Protein Coding	40	GC02P032063	17.58852	https://www.genecards.org/cgi-bin/carddisp.pl?gene=SPAST
CBL	Cbl Proto-Oncogene	Protein Coding	51	GC11P119206	17.58418	https://www.genecards.org/cgi-bin/carddisp.pl?gene=CBL
PRKG1	Protein Kinase CGMP-Dependent 1	Protein Coding	51	GC10P050991	17.57796	https://www.genecards.org/cgi-bin/carddisp.pl?gene=PRKG1

EARS2	Glutamyl-TRNA Synthetase 2, Mitochondrial	Protein Coding	43	GC16M023527	17.57607	https://www.genecards.org/cgi-bin/carddisp.pl?gene=EARS2
ZFYVE26	Zinc Finger FYVE-Type Containing 26	Protein Coding	39	GC14M067727	17.56585	https://www.genecards.org/cgi-bin/carddisp.pl?gene=ZFYVE26
MIR378A	MicroRNA 378a	RNA Gene	19	GC05P149732	17.55978	https://www.genecards.org/cgi-bin/carddisp.pl?gene=MIR378A
PHB	Prohibitin	Protein Coding	48	GC17M049404	17.55864	https://www.genecards.org/cgi-bin/carddisp.pl?gene=PHB
MUC5B	Mucin 5B, Oligomeric Mucus/Gel-Forming	Protein Coding	42	GC11P001244	17.55026	https://www.genecards.org/cgi-bin/carddisp.pl?gene=MUC5B
LIG4	DNA Ligase 4	Protein Coding	48	GC13M108207	17.5399	https://www.genecards.org/cgi-bin/carddisp.pl?gene=LIG4
ASXL1	ASXL Transcriptional Regulator 1	Protein Coding	43	GC20P032364	17.53126	https://www.genecards.org/cgi-bin/carddisp.pl?gene=ASXL1
DARS2	Aspartyl-TRNA Synthetase 2, Mitochondrial	Protein Coding	43	GC01P173824	17.52016	https://www.genecards.org/cgi-bin/carddisp.pl?gene=DARS2
SCN1B	Sodium Voltage-Gated Channel Beta Subunit 1	Protein Coding	45	GC19P035030	17.5088	https://www.genecards.org/cgi-bin/carddisp.pl?gene=SCN1B
MGMT	O-6-Methylguanine-DNA Methyltransferase	Protein Coding	50	GC10P129467	17.50516	https://www.genecards.org/cgi-bin/carddisp.pl?gene=MGMT
ACAN	Aggrecan	Protein Coding	46	GC15P088813	17.49879	https://www.genecards.org/cgi-bin/carddisp.pl?gene=ACAN
IL22	Interleukin 22	Protein Coding	42	GC12M068248	17.49619	https://www.genecards.org/cgi-bin/carddisp.pl?gene=IL22
HSPG2	Heparan Sulfate Proteoglycan 2	Protein Coding	46	GC01M021822	17.49487	https://www.genecards.org/cgi-bin/carddisp.pl?gene=HSPG2
MIR328	MicroRNA 328	RNA Gene	18	GC16M067203	17.4909	https://www.genecards.org/cgi-bin/carddisp.pl?gene=MIR328
DNMT3A	DNA Methyltransferase 3 Alpha	Protein Coding	52	GC02M025228	17.49035	https://www.genecards.org/cgi-bin/carddisp.pl?gene=DNMT3A
LEPQTL1	Leptin, Serum Levels Of	Genetic Locus	2	GC02U903086	17.46852	https://www.genecards.org/cgi-bin/carddisp.pl?gene=LEPQTL1
DEGS1	Delta 4-Desaturase, Sphingolipid 1	Protein Coding	41	GC01P224175	17.44939	https://www.genecards.org/cgi-bin/carddisp.pl?gene=DEGS1
TPO	Thyroid Peroxidase	Protein Coding	49	GC02P001374	17.43083	https://www.genecards.org/cgi-bin/carddisp.pl?gene=TPO
RDH12	Retinol Dehydrogenase 12	Protein Coding	45	GC14P067701	17.42397	https://www.genecards.org/cgi-bin/carddisp.pl?gene=RDH12
TAT	Tyrosine Aminotransferase	Protein Coding	45	GC16M071565	17.42278	https://www.genecards.org/cgi-bin/carddisp.pl?gene=TAT

TFAP2A	Transcription Factor AP-2 Alpha	Protein Coding	48	GC06M010393	17.41299	https://www.genecards.org/cgi-bin/carddisp.pl?gene=TFAP2A
LOC110806263	TERT 5' Regulatory Region	Biological Region	2	GC05P001294	17.39179	https://www.genecards.org/cgi-bin/carddisp.pl?gene=LOC110806263
SERPINF1	Serpin Family F Member 1	Protein Coding	45	GC17P001761	17.36952	https://www.genecards.org/cgi-bin/carddisp.pl?gene=SERPINF1
ACSF3	Acyl-CoA Synthetase Family Member 3	Protein Coding	43	GC16P089088	17.36868	https://www.genecards.org/cgi-bin/carddisp.pl?gene=ACSF3
CDK5	Cyclin Dependent Kinase 5	Protein Coding	54	GC07M151053	17.36576	https://www.genecards.org/cgi-bin/carddisp.pl?gene=CDK5
KCNQ1OT1	KCNQ1 Opposite Strand/Antisense Transcript 1	RNA Gene	26	GC11M002661	17.36142	https://www.genecards.org/cgi-bin/carddisp.pl?gene=KCNQ1OT1
NDUFS8	NADH:Ubiquinone Oxidoreductase Core Subunit S8	Protein Coding	46	GC11P068030	17.34398	https://www.genecards.org/cgi-bin/carddisp.pl?gene=NDUFS8
NR0B2	Nuclear Receptor Subfamily 0 Group B Member 2	Protein Coding	44	GC01M026922	17.3346	https://www.genecards.org/cgi-bin/carddisp.pl?gene=NR0B2
KMT2D	Lysine Methyltransferase 2D	Protein Coding	42	GC12M049018	17.33155	https://www.genecards.org/cgi-bin/carddisp.pl?gene=KMT2D
IFNL3	Interferon Lambda 3	Protein Coding	35	GC19M039243	17.31583	https://www.genecards.org/cgi-bin/carddisp.pl?gene=IFNL3
FANCC	FA Complementation Group C	Protein Coding	48	GC09M095099	17.31461	https://www.genecards.org/cgi-bin/carddisp.pl?gene=FANCC
APOC1	Apolipoprotein C1	Protein Coding	41	GC19P044914	17.29979	https://www.genecards.org/cgi-bin/carddisp.pl?gene=APOC1
RTEL1	Regulator Of Telomere Elongation Helicase 1	Protein Coding	41	GC20P063658	17.26829	https://www.genecards.org/cgi-bin/carddisp.pl?gene=RTEL1
HBG1	Hemoglobin Subunit Gamma 1	Protein Coding	43	GC11M005431	17.26441	https://www.genecards.org/cgi-bin/carddisp.pl?gene=HBG1
CDKN2B-AS1	CDKN2B Antisense RNA 1	RNA Gene	22	GC09P021994	17.25854	https://www.genecards.org/cgi-bin/carddisp.pl?gene=CDKN2B-AS1
GRB2	Growth Factor Receptor Bound Protein 2	Protein Coding	49	GC17M075318	17.24964	https://www.genecards.org/cgi-bin/carddisp.pl?gene=GRB2
SLC27A3	Solute Carrier Family 27 Member 3	Protein Coding	40	GC01P153786	17.23686	https://www.genecards.org/cgi-bin/carddisp.pl?gene=SLC27A3

RBP1	Retinol Binding Protein 1	Protein Coding	40	GC03M139517	17.22531	https://www.genecards.org/cgi-bin/carddisp.pl?gene=RBP1
PLAUR	Plasminogen Activator, Urokinase Receptor	Protein Coding	45	GC19M043646	17.22167	https://www.genecards.org/cgi-bin/carddisp.pl?gene=PLAUR
EMD	Emerin	Protein Coding	45	GC0XP154379	17.18898	https://www.genecards.org/cgi-bin/carddisp.pl?gene=EMD
FLT4	Fms Related Receptor Tyrosine Kinase 4	Protein Coding	52	GC05M180607	17.17457	https://www.genecards.org/cgi-bin/carddisp.pl?gene=FLT4
PCNA	Proliferating Cell Nuclear Antigen	Protein Coding	52	GC20M005114	17.17124	https://www.genecards.org/cgi-bin/carddisp.pl?gene=PCNA
ADM	Adrenomedullin	Protein Coding	45	GC11P010304	17.15399	https://www.genecards.org/cgi-bin/carddisp.pl?gene=ADM
SOX2	SRY-Box Transcription Factor 2	Protein Coding	48	GC03P181711	17.14379	https://www.genecards.org/cgi-bin/carddisp.pl?gene=SOX2
CALB2	Calbindin 2	Protein Coding	41	GC16P071392	17.14216	https://www.genecards.org/cgi-bin/carddisp.pl?gene=CALB2
FBP1	Fructose-Bisphosphatase 1	Protein Coding	50	GC09M094603	17.13457	https://www.genecards.org/cgi-bin/carddisp.pl?gene=FBP1
XRCC2	X-Ray Repair Cross Complementing 2	Protein Coding	43	GC07M152644	17.10191	https://www.genecards.org/cgi-bin/carddisp.pl?gene=XRCC2
LOC111674472	DNase I Hypersensitive Sites In Introns 16 And 17a Of CFTR	Biological Region	2	GC07P117607	17.10055	https://www.genecards.org/cgi-bin/carddisp.pl?gene=LOC111674472
KCNJ5	Potassium Inwardly Rectifying Channel Subfamily J Member 5	Protein Coding	48	GC11P128891	17.08913	https://www.genecards.org/cgi-bin/carddisp.pl?gene=KCNJ5
BLK	BLK Proto-Oncogene, Src Family Tyrosine Kinase	Protein Coding	51	GC08P011486	17.08165	https://www.genecards.org/cgi-bin/carddisp.pl?gene=BLK
DNASE1	Deoxyribonuclease 1	Protein Coding	43	GC16P003611	17.08039	https://www.genecards.org/cgi-bin/carddisp.pl?gene=DNASE1
AKT3	AKT Serine/Threonine Kinase 3	Protein Coding	54	GC01M243488	17.07912	https://www.genecards.org/cgi-bin/carddisp.pl?gene=AKT3
GATA2	GATA Binding Protein 2	Protein Coding	48	GC03M128479	17.0775	https://www.genecards.org/cgi-bin/carddisp.pl?gene=GATA2
MT-TE	Mitochondrially Encoded TRNA-Glu (GAA/G)	RNA Gene	13	GCMTM012350	17.06599	https://www.genecards.org/cgi-bin/carddisp.pl?gene=MT-TE

SFTPB	Surfactant Protein B	Protein Coding	44	GC02M085657	17.06023	https://www.genecards.org/cgi-bin/carddisp.pl?gene=SFTPB
PNLIP	Pancreatic Lipase	Protein Coding	48	GC10P116545	17.04584	https://www.genecards.org/cgi-bin/carddisp.pl?gene=PNLIP
CXCR3	C-X-C Motif Chemokine Receptor 3	Protein Coding	45	GC0XM071615	17.03813	https://www.genecards.org/cgi-bin/carddisp.pl?gene=CXCR3
BLVRA	Biliverdin Reductase A	Protein Coding	45	GC07P043758	17.02469	https://www.genecards.org/cgi-bin/carddisp.pl?gene=BLVRA
PLTP	Phospholipid Transfer Protein	Protein Coding	44	GC20M045898	17.01707	https://www.genecards.org/cgi-bin/carddisp.pl?gene=PLTP
WWOX	WW Domain Containing Oxidoreductase	Protein Coding	48	GC16P078099	17.01404	https://www.genecards.org/cgi-bin/carddisp.pl?gene=WWOX
FUCA1	Alpha-L-Fucosidase 1	Protein Coding	47	GC01M023845	16.9952	https://www.genecards.org/cgi-bin/carddisp.pl?gene=FUCA1
TSHR	Thyroid Stimulating Hormone Receptor	Protein Coding	48	GC14P080954	16.98258	https://www.genecards.org/cgi-bin/carddisp.pl?gene=TSHR
CFHR1	Complement Factor H Related 1	Protein Coding	42	GC01P196822	16.97022	https://www.genecards.org/cgi-bin/carddisp.pl?gene=CFHR1
ATP2C1	ATPase Secretory Pathway Ca2+ Transporting 1	Protein Coding	45	GC03P130850	16.96741	https://www.genecards.org/cgi-bin/carddisp.pl?gene=ATP2C1
GRM5	Glutamate Metabotropic Receptor 5	Protein Coding	48	GC11M088504	16.96287	https://www.genecards.org/cgi-bin/carddisp.pl?gene=GRM5
BMP4	Bone Morphogenetic Protein 4	Protein Coding	50	GC14M053949	16.94471	https://www.genecards.org/cgi-bin/carddisp.pl?gene=BMP4
DKC1	Dyskerin Pseudouridine Synthase 1	Protein Coding	48	GC0XP154762	16.93802	https://www.genecards.org/cgi-bin/carddisp.pl?gene=DKC1
SLC12A3	Solute Carrier Family 12 Member 3	Protein Coding	47	GC16P056865	16.92522	https://www.genecards.org/cgi-bin/carddisp.pl?gene=SLC12A3
SOCS1	Suppressor Of Cytokine Signaling 1	Protein Coding	44	GC16M011255	16.91556	https://www.genecards.org/cgi-bin/carddisp.pl?gene=SOCS1
JPH3	Junctophilin 3	Protein Coding	42	GC16P087601	16.88008	https://www.genecards.org/cgi-bin/carddisp.pl?gene=JPH3
PRDX1	Peroxiredoxin 1	Protein Coding	50	GC01M045511	16.84415	https://www.genecards.org/cgi-bin/carddisp.pl?gene=PRDX1
NQO2	N-Ribosyldihydronicotinamide:Quinone Reductase 2	Protein Coding	46	GC06P003014	16.83867	https://www.genecards.org/cgi-bin/carddisp.pl?gene=NQO2
HDAC1	Histone Deacetylase 1	Protein Coding	50	GC01P032292	16.8233	https://www.genecards.org/cgi-bin/carddisp.pl?gene=HDAC1
BECN1	Beclin 1	Protein Coding	47	GC17M042810	16.81464	https://www.genecards.org/cgi-bin/carddisp.pl?gene=BECN1

CYB5A	Cytochrome B5 Type A	Protein Coding	45	GC18M074250	16.79537	https://www.genecards.org/cgi-bin/carddisp.pl?gene=CYB5A
ASGR2	Asialoglycoprotein Receptor 2	Protein Coding	41	GC17M007101	16.7761	https://www.genecards.org/cgi-bin/carddisp.pl?gene=ASGR2
MUC5AC	Mucin 5AC, Oligomeric Mucus/Gel-Forming	Protein Coding	39	GC11P001151	16.76953	https://www.genecards.org/cgi-bin/carddisp.pl?gene=MUC5AC
PECR	Peroxisomal Trans-2-Enoyl-CoA Reductase	Protein Coding	43	GC02M215996	16.7628	https://www.genecards.org/cgi-bin/carddisp.pl?gene=PECR
POMT1	Protein O-Mannosyltransferase 1	Protein Coding	47	GC09P131502	16.75833	https://www.genecards.org/cgi-bin/carddisp.pl?gene=POMT1
MT-TS1	Mitochondrially Encoded TRNA-Ser (UCN) 1	RNA Gene	15	GCMTM005917	16.75518	https://www.genecards.org/cgi-bin/carddisp.pl?gene=MT-TS1
MYCN	MYCN Proto-Oncogene, BHLH Transcription Factor	Protein Coding	47	GC02P015949	16.74515	https://www.genecards.org/cgi-bin/carddisp.pl?gene=MYCN
AIP	Aryl Hydrocarbon Receptor Interacting Protein	Protein Coding	45	GC11P067468	16.7333	https://www.genecards.org/cgi-bin/carddisp.pl?gene=AIP
CFB	Complement Factor B	Protein Coding	46	GC06P031945	16.72658	https://www.genecards.org/cgi-bin/carddisp.pl?gene=CFB
NTS	Neurotensin	Protein Coding	41	GC12P085876	16.70058	https://www.genecards.org/cgi-bin/carddisp.pl?gene=NTS
FNDC5	Fibronectin Type III Domain Containing 5	Protein Coding	34	GC01M032866	16.69098	https://www.genecards.org/cgi-bin/carddisp.pl?gene=FNDC5
GZMB	Granzyme B	Protein Coding	46	GC14M024630	16.68339	https://www.genecards.org/cgi-bin/carddisp.pl?gene=GZMB
U2AF1	U2 Small Nuclear RNA Auxiliary Factor 1	Protein Coding	44	GC21M043092	16.68231	https://www.genecards.org/cgi-bin/carddisp.pl?gene=U2AF1
TWIST1	Twist Family BHLH Transcription Factor 1	Protein Coding	47	GC07M019020	16.68091	https://www.genecards.org/cgi-bin/carddisp.pl?gene=TWIST1
PIK3R2	Phosphoinositide-3-Kinase Regulatory Subunit 2	Protein Coding	50	GC19P018153	16.66394	https://www.genecards.org/cgi-bin/carddisp.pl?gene=PIK3R2
GSS	Glutathione Synthetase	Protein Coding	47	GC20M034928	16.64843	https://www.genecards.org/cgi-bin/carddisp.pl?gene=GSS

NPHS2	NPHS2 Stomatin Family Member, Podocin	Protein Coding	42	GC01M179519	16.62767	https://www.genecards.org/cgi-bin/carddisp.pl?gene=NPHS2
BRIP1	BRCA1 Interacting Helicase 1	Protein Coding	48	GC17M061679	16.60106	https://www.genecards.org/cgi-bin/carddisp.pl?gene=BRIP1
HOTAIR	HOX Transcript Antisense RNA	RNA Gene	26	GC12M053962	16.59629	https://www.genecards.org/cgi-bin/carddisp.pl?gene=HOTAIR
SERPINH1	Serpin Family H Member 1	Protein Coding	46	GC11P075562	16.57393	https://www.genecards.org/cgi-bin/carddisp.pl?gene=SERPINH1
CXCL1	C-X-C Motif Chemokine Ligand 1	Protein Coding	43	GC04P073869	16.56734	https://www.genecards.org/cgi-bin/carddisp.pl?gene=CXCL1
TINF2	TERF1 Interacting Nuclear Factor 2	Protein Coding	42	GC14M024234	16.55839	https://www.genecards.org/cgi-bin/carddisp.pl?gene=TINF2
AQP1	Aquaporin 1 (Colton Blood Group)	Protein Coding	47	GC07P030911	16.5561	https://www.genecards.org/cgi-bin/carddisp.pl?gene=AQP1
FZD4	Frizzled Class Receptor 4	Protein Coding	51	GC11M086945	16.54615	https://www.genecards.org/cgi-bin/carddisp.pl?gene=FZD4
HTR2C	5-Hydroxytryptamine Receptor 2C	Protein Coding	48	GC0XP114584	16.51718	https://www.genecards.org/cgi-bin/carddisp.pl?gene=HTR2C
CA2	Carbonic Anhydrase 2	Protein Coding	52	GC08P085463	16.51351	https://www.genecards.org/cgi-bin/carddisp.pl?gene=CA2
STAT6	Signal Transducer And Activator Of Transcription 6	Protein Coding	50	GC12M057095	16.50465	https://www.genecards.org/cgi-bin/carddisp.pl?gene=STAT6
FGB	Fibrinogen Beta Chain	Protein Coding	47	GC04P154572	16.48997	https://www.genecards.org/cgi-bin/carddisp.pl?gene=FGB
SLC10A2	Solute Carrier Family 10 Member 2	Protein Coding	43	GC13M103043	16.4813	https://www.genecards.org/cgi-bin/carddisp.pl?gene=SLC10A2
GLS	Glutaminase	Protein Coding	48	GC02P190880	16.46054	https://www.genecards.org/cgi-bin/carddisp.pl?gene=GLS
EIF2AK2	Eukaryotic Translation Initiation Factor 2 Alpha Kinase 2	Protein Coding	46	GC02M037099	16.45991	https://www.genecards.org/cgi-bin/carddisp.pl?gene=EIF2AK2
TKT	Transketolase	Protein Coding	48	GC03M053224	16.45034	https://www.genecards.org/cgi-bin/carddisp.pl?gene=TKT
SUMF1	Sulfatase Modifying Factor 1	Protein Coding	43	GC03M003700	16.44326	https://www.genecards.org/cgi-bin/carddisp.pl?gene=SUMF1

KCNJ10	Potassium Inwardly Rectifying Channel Subfamily J Member 10	Protein Coding	45	GC01M159998	16.42751	https://www.genecards.org/cgi-bin/carddisp.pl?gene=KCNJ10
FH	Fumarate Hydratase	Protein Coding	46	GC01M241499	16.41443	https://www.genecards.org/cgi-bin/carddisp.pl?gene=FH
CS	Citrate Synthase	Protein Coding	45	GC12M056271	16.41187	https://www.genecards.org/cgi-bin/carddisp.pl?gene=CS
ADD1	Adducin 1	Protein Coding	45	GC04P002855	16.4056	https://www.genecards.org/cgi-bin/carddisp.pl?gene=ADD1
TBC1D24	TBC1 Domain Family Member 24	Protein Coding	38	GC16P002475	16.40266	https://www.genecards.org/cgi-bin/carddisp.pl?gene=TBC1D24
MFSD8	Major Facilitator Superfamily Domain Containing 8	Protein Coding	37	GC04M127917	16.39691	https://www.genecards.org/cgi-bin/carddisp.pl?gene=MFSD8
C5	Complement C5	Protein Coding	47	GC09M120952	16.39404	https://www.genecards.org/cgi-bin/carddisp.pl?gene=C5
KIAA0586	KIAA0586	Protein Coding	36	GC14P058427	16.39266	https://www.genecards.org/cgi-bin/carddisp.pl?gene=KIAA0586
CCNA2	Cyclin A2	Protein Coding	46	GC04M121816	16.38851	https://www.genecards.org/cgi-bin/carddisp.pl?gene=CCNA2
PF4	Platelet Factor 4	Protein Coding	42	GC04M073980	16.38232	https://www.genecards.org/cgi-bin/carddisp.pl?gene=PF4
ALDH1B1	Aldehyde Dehydrogenase 1 Family Member B1	Protein Coding	45	GC09P038392	16.37377	https://www.genecards.org/cgi-bin/carddisp.pl?gene=ALDH1B1
DHFR	Dihydrofolate Reductase	Protein Coding	50	GC05M080626	16.36003	https://www.genecards.org/cgi-bin/carddisp.pl?gene=DHFR
PLIN1	Perilipin 1	Protein Coding	45	GC15M089664	16.35218	https://www.genecards.org/cgi-bin/carddisp.pl?gene=PLIN1
THBS1	Thrombospondin 1	Protein Coding	45	GC15P039581	16.34905	https://www.genecards.org/cgi-bin/carddisp.pl?gene=THBS1
AHCY	Adenosylhomocysteinase	Protein Coding	51	GC20M034341	16.34795	https://www.genecards.org/cgi-bin/carddisp.pl?gene=AHCY
CDC42	Cell Division Cycle 42	Protein Coding	51	GC01P022112	16.34657	https://www.genecards.org/cgi-bin/carddisp.pl?gene=CDC42
ATP6AP2	ATPase H+ Transporting Accessory Protein 2	Protein Coding	45	GC0XP040582	16.3449	https://www.genecards.org/cgi-bin/carddisp.pl?gene=ATP6AP2
LMBRD1	LMBR1 Domain Containing 1	Protein Coding	39	GC06M069675	16.33275	https://www.genecards.org/cgi-bin/carddisp.pl?gene=LMBRD1
ACTN4	Actinin Alpha 4	Protein Coding	47	GC19P038647	16.31758	https://www.genecards.org/cgi-bin/carddisp.pl?gene=ACTN4
ANGPTL3	Angiopoietin Like 3	Protein Coding	46	GC01P062597	16.31535	https://www.genecards.org/cgi-bin/carddisp.pl?gene=ANGPTL3
MECR	Mitochondrial Trans-2-Enoyl-CoA Reductase	Protein Coding	44	GC01M029192	16.31058	https://www.genecards.org/cgi-bin/carddisp.pl?gene=MECR

IFT122	Intraflagellar Transport 122 Protein	Protein Coding	40	GC03P129440	16.3056	https://www.genecards.org/cgi-bin/carddisp.pl?gene=IFT122
PTPN3	Tyrosine Phosphatase Non-Receptor Type 3	Protein Coding	45	GC09M109375	16.29969	https://www.genecards.org/cgi-bin/carddisp.pl?gene=PTPN3
IL7	Interleukin 7	Protein Coding	43	GC08M078689	16.29952	https://www.genecards.org/cgi-bin/carddisp.pl?gene=IL7
CYP27B1	Cytochrome P450 Family 27 Subfamily B Member 1	Protein Coding	48	GC12M057757	16.29617	https://www.genecards.org/cgi-bin/carddisp.pl?gene=CYP27B1
STAR	Steroidogenic Acute Regulatory Protein	Protein Coding	47	GC08M038145	16.29576	https://www.genecards.org/cgi-bin/carddisp.pl?gene=STAR
ODC1	Ornithine Decarboxylase 1	Protein Coding	49	GC02M010432	16.29564	https://www.genecards.org/cgi-bin/carddisp.pl?gene=ODC1
TTPA	Alpha Tocopherol Transfer Protein	Protein Coding	41	GC08M063048	16.29455	https://www.genecards.org/cgi-bin/carddisp.pl?gene=TTPA
MYO7A	Myosin VIIA	Protein Coding	43	GC11P077128	16.28915	https://www.genecards.org/cgi-bin/carddisp.pl?gene=MYO7A
CHI3L1	Chitinase 3 Like 1	Protein Coding	44	GC01M203148	16.28605	https://www.genecards.org/cgi-bin/carddisp.pl?gene=CHI3L1
KCNV2	Potassium Voltage-Gated Channel Modifier Subfamily V Member 2	Protein Coding	41	GC09P002717	16.28587	https://www.genecards.org/cgi-bin/carddisp.pl?gene=KCNV2
SMARCA2	SWI/SNF Related, Matrix Associated, Actin Dependent Regulator Of Chromatin, Subfamily A, Member 2	Protein Coding	50	GC09P001980	16.28386	https://www.genecards.org/cgi-bin/carddisp.pl?gene=SMARCA2
IRGM	Immunity Related GTPase M	Protein Coding	38	GC05P150846	16.27772	https://www.genecards.org/cgi-bin/carddisp.pl?gene=IRGM
SIL1	SIL1 Nucleotide Exchange Factor	Protein Coding	41	GC05M138957	16.26973	https://www.genecards.org/cgi-bin/carddisp.pl?gene=SIL1
COX4I1	Cytochrome C Oxidase Subunit 4I1	Protein Coding	47	GC16P085798	16.25666	https://www.genecards.org/cgi-bin/carddisp.pl?gene=COX4I1
VPS13B	Vacuolar Protein Sorting 13 Homolog B	Protein Coding	40	GC08P099011	16.25064	https://www.genecards.org/cgi-bin/carddisp.pl?gene=VPS13B
CD163	CD163 Molecule	Protein Coding	43	GC12M007471	16.23718	https://www.genecards.org/cgi-bin/carddisp.pl?gene=CD163

NDUFA6	NADH:Ubiquinone Oxidoreductase Subunit A6	Protein Coding	45	GC22M042085	16.23557	https://www.genecards.org/cgi-bin/carddisp.pl?gene=NDUFA6
S100A8	S100 Calcium Binding Protein A8	Protein Coding	43	GC01M153391	16.23479	https://www.genecards.org/cgi-bin/carddisp.pl?gene=S100A8
COL4A2	Collagen Type IV Alpha 2 Chain	Protein Coding	45	GC13P110305	16.2328	https://www.genecards.org/cgi-bin/carddisp.pl?gene=COL4A2
CCNB1	Cyclin B1	Protein Coding	47	GC05P069167	16.22871	https://www.genecards.org/cgi-bin/carddisp.pl?gene=CCNB1
TNFRSF10B	TNF Receptor Superfamily Member 10b	Protein Coding	51	GC08M023020	16.2237	https://www.genecards.org/cgi-bin/carddisp.pl?gene=TNFRSF10B
PEPD	Peptidase D	Protein Coding	46	GC19M033386	16.21912	https://www.genecards.org/cgi-bin/carddisp.pl?gene=PEPD
CAMTA1	Calmodulin Binding Transcription Activator 1	Protein Coding	41	GC01P006806	16.20734	https://www.genecards.org/cgi-bin/carddisp.pl?gene=CAMTA1
CHKA	Choline Kinase Alpha	Protein Coding	42	GC11M068052	16.20527	https://www.genecards.org/cgi-bin/carddisp.pl?gene=CHKA
DAO	D-Amino Acid Oxidase	Protein Coding	45	GC12P108859	16.20326	https://www.genecards.org/cgi-bin/carddisp.pl?gene=DAO
LOC110806262	Solute Carrier Family 6 Member 4 Gene Promoter	Biological Region	1	GC17P030235	16.19947	https://www.genecards.org/cgi-bin/carddisp.pl?gene=LOC110806262
ATP1A3	ATPase Na+/K+ Transporting Subunit Alpha 3	Protein Coding	48	GC19M041966	16.18552	https://www.genecards.org/cgi-bin/carddisp.pl?gene=ATP1A3
GNB4	G Protein Subunit Beta 4	Protein Coding	43	GC03M179397	16.16672	https://www.genecards.org/cgi-bin/carddisp.pl?gene=GNB4
HDAC8	Histone Deacetylase 8	Protein Coding	48	GC0XM072329	16.14522	https://www.genecards.org/cgi-bin/carddisp.pl?gene=HDAC8
BBS2	Bardet-Biedl Syndrome 2	Protein Coding	42	GC16M056467	16.13379	https://www.genecards.org/cgi-bin/carddisp.pl?gene=BBS2
ADNP	Activity Dependent Neuroprotector Homeobox	Protein Coding	43	GC20M050888	16.12299	https://www.genecards.org/cgi-bin/carddisp.pl?gene=ADNP
FOXO1	Forkhead Box O1	Protein Coding	48	GC13M040555	16.10978	https://www.genecards.org/cgi-bin/carddisp.pl?gene=FOXO1
FHIT	Fragile Histidine Triad Diadenosine Triphosphatase	Protein Coding	45	GC03M059747	16.10194	https://www.genecards.org/cgi-bin/carddisp.pl?gene=FHIT
PLCG1	Phospholipase C Gamma 1	Protein Coding	49	GC20P041136	16.09266	https://www.genecards.org/cgi-bin/carddisp.pl?gene=PLCG1
IRF8	Interferon Regulatory Factor 8	Protein Coding	47	GC16P085898	16.08925	https://www.genecards.org/cgi-bin/carddisp.pl?gene=IRF8

DYSF	Dysferlin	Protein Coding	44	GC02P071453	16.0834	https://www.genecards.org/cgi-bin/carddisp.pl?gene=DYSF
ANGPT1	Angiopoietin 1	Protein Coding	46	GC08M107246	16.08221	https://www.genecards.org/cgi-bin/carddisp.pl?gene=ANGPT1
OGG1	8-Oxoguanine DNA Glycosylase	Protein Coding	47	GC03P009751	16.08128	https://www.genecards.org/cgi-bin/carddisp.pl?gene=OGG1
RNASEH2B	Ribonuclease H2 Subunit B	Protein Coding	37	GC13P050909	16.06469	https://www.genecards.org/cgi-bin/carddisp.pl?gene=RNASEH2B
LTBP3	Latent Transforming Growth Factor Beta Binding Protein 3	Protein Coding	42	GC11M065538	16.06443	https://www.genecards.org/cgi-bin/carddisp.pl?gene=LTBP3
SLC39A8	Solute Carrier Family 39 Member 8	Protein Coding	44	GC04M102252	16.0611	https://www.genecards.org/cgi-bin/carddisp.pl?gene=SLC39A8
NDUFA10	NADH:Ubiquinone Oxidoreductase Subunit A10	Protein Coding	45	GC02M239893	16.05857	https://www.genecards.org/cgi-bin/carddisp.pl?gene=NDUFA10
BBS7	Bardet-Biedl Syndrome 7	Protein Coding	38	GC04M121824	16.05673	https://www.genecards.org/cgi-bin/carddisp.pl?gene=BBS7
GHRH	Growth Hormone Releasing Hormone	Protein Coding	40	GC20M037251	16.04934	https://www.genecards.org/cgi-bin/carddisp.pl?gene=GHRH
APOA4	Apolipoprotein A4	Protein Coding	43	GC11M116820	16.04388	https://www.genecards.org/cgi-bin/carddisp.pl?gene=APOA4
BAK1	BCL2 Antagonist/Killer 1	Protein Coding	45	GC06M033572	16.03828	https://www.genecards.org/cgi-bin/carddisp.pl?gene=BAK1
ERCC8	ERCC Excision Repair 8, CSA Ubiquitin Ligase Complex Subunit	Protein Coding	43	GC05M060874	16.02978	https://www.genecards.org/cgi-bin/carddisp.pl?gene=ERCC8
TP63	Tumor Protein P63	Protein Coding	49	GC03P189598	16.01756	https://www.genecards.org/cgi-bin/carddisp.pl?gene=TP63
SNAI2	Snail Family Transcriptional Repressor 2	Protein Coding	45	GC08M048917	16.01589	https://www.genecards.org/cgi-bin/carddisp.pl?gene=SNAI2
SELENON	Selenoprotein N	Protein Coding	33	GC01P025800	16.00738	https://www.genecards.org/cgi-bin/carddisp.pl?gene=SELENON
JAK3	Janus Kinase 3	Protein Coding	52	GC19M017824	16.00489	https://www.genecards.org/cgi-bin/carddisp.pl?gene=JAK3
CNTNAP2	Contactin Associated Protein 2	Protein Coding	44	GC07P146116	16.00147	https://www.genecards.org/cgi-bin/carddisp.pl?gene=CNTNAP2
FHL1	Four And A Half LIM Domains 1	Protein Coding	47	GC0XP136146	15.99871	https://www.genecards.org/cgi-bin/carddisp.pl?gene=FHL1
WRAP53	WD Repeat Containing Antisense To TP53	Protein Coding	41	GC17P009114	15.99333	https://www.genecards.org/cgi-bin/carddisp.pl?gene=WRAP53

ABCG1	ATP Binding Cassette Subfamily G Member 1	Protein Coding	44	GC21P042199	15.96082	https://www.genecards.org/cgi-bin/carddisp.pl?gene=ABCG1
M6PR	Mannose-6-Phosphate Receptor, Cation Dependent	Protein Coding	44	GC12M008969	15.95886	https://www.genecards.org/cgi-bin/carddisp.pl?gene=M6PR
FABP5P3	Fatty Acid Binding Protein 5 Pseudogene 3	Pseudogene	19	GC07P152436	15.95772	https://www.genecards.org/cgi-bin/carddisp.pl?gene=FABP5P3
SETD2	SET Domain Containing 2, Histone Lysine Methyltransferase	Protein Coding	47	GC03M047033	15.9547	https://www.genecards.org/cgi-bin/carddisp.pl?gene=SETD2
GDF15	Growth Differentiation Factor 15	Protein Coding	43	GC19P026656	15.94497	https://www.genecards.org/cgi-bin/carddisp.pl?gene=GDF15
NDUFB11	NADH:Ubiquinone Oxidoreductase Subunit B11	Protein Coding	40	GC0XM047142	15.94305	https://www.genecards.org/cgi-bin/carddisp.pl?gene=NDUFB11
AKR1C2	Aldo-Keto Reductase Family 1 Member C2	Protein Coding	47	GC10M004987	15.9403	https://www.genecards.org/cgi-bin/carddisp.pl?gene=AKR1C2
PTH1R	Parathyroid Hormone 1 Receptor	Protein Coding	50	GC03P046877	15.93879	https://www.genecards.org/cgi-bin/carddisp.pl?gene=PTH1R
MT-TF	Mitochondrially Encoded TRNA-Phe (UUU/C)	RNA Gene	14	GCMTP000003	15.93401	https://www.genecards.org/cgi-bin/carddisp.pl?gene=MT-TF
POR	Cytochrome P450 Oxidoreductase	Protein Coding	50	GC07P075899	15.924	https://www.genecards.org/cgi-bin/carddisp.pl?gene=POR
MUTYH	MutY DNA Glycosylase	Protein Coding	45	GC01M045329	15.91155	https://www.genecards.org/cgi-bin/carddisp.pl?gene=MUTYH
CRX	Cone-Rod Homeobox	Protein Coding	44	GC19P047819	15.90081	https://www.genecards.org/cgi-bin/carddisp.pl?gene=CRX
CD55	CD55 Molecule (Cromer Blood Group)	Protein Coding	48	GC01P207321	15.89428	https://www.genecards.org/cgi-bin/carddisp.pl?gene=CD55
GDF6	Growth Differentiation Factor 6	Protein Coding	43	GC08M096142	15.89301	https://www.genecards.org/cgi-bin/carddisp.pl?gene=GDF6
NDUFB8	NADH:Ubiquinone Oxidoreductase Subunit B8	Protein Coding	43	GC10M100523	15.89291	https://www.genecards.org/cgi-bin/carddisp.pl?gene=NDUFB8
TRIM32	Tripartite Motif Containing 32	Protein Coding	44	GC09P116687	15.89095	https://www.genecards.org/cgi-bin/carddisp.pl?gene=TRIM32

ITGA4	Integrin Subunit Alpha 4	Protein Coding	49	GC02P181456	15.87939	https://www.genecards.org/cgi-bin/carddisp.pl?gene=ITGA4
SPTLC2	Serine Palmitoyltransferase Long Chain Base Subunit 2	Protein Coding	48	GC14M077505	15.87024	https://www.genecards.org/cgi-bin/carddisp.pl?gene=SPTLC2
BCR	BCR Activator Of RhoGEF And GTPase	Protein Coding	52	GC22P023179	15.86954	https://www.genecards.org/cgi-bin/carddisp.pl?gene=BCR
CNTNAP1	Contactin Associated Protein 1	Protein Coding	43	GC17P042680	15.86062	https://www.genecards.org/cgi-bin/carddisp.pl?gene=CNTNAP1
CDC73	Cell Division Cycle 73	Protein Coding	45	GC01P193121	15.85806	https://www.genecards.org/cgi-bin/carddisp.pl?gene=CDC73
SNCB	Synuclein Beta	Protein Coding	44	GC05M176620	15.85333	https://www.genecards.org/cgi-bin/carddisp.pl?gene=SNCB
MIR320A	MicroRNA 320a	RNA Gene	19	GC08M022249	15.84664	https://www.genecards.org/cgi-bin/carddisp.pl?gene=MIR320A
TYK2	Tyrosine Kinase 2	Protein Coding	53	GC19M010350	15.83986	https://www.genecards.org/cgi-bin/carddisp.pl?gene=TYK2
ITGA3	Integrin Subunit Alpha 3	Protein Coding	47	GC17P050055	15.83595	https://www.genecards.org/cgi-bin/carddisp.pl?gene=ITGA3
FLI1	Fli-1 Proto-Oncogene, ETS Transcription Factor	Protein Coding	49	GC11P128686	15.80927	https://www.genecards.org/cgi-bin/carddisp.pl?gene=FLI1
TGIF1	TGFB Induced Factor Homeobox 1	Protein Coding	47	GC18P003411	15.80026	https://www.genecards.org/cgi-bin/carddisp.pl?gene=TGIF1
MIRLET7B	MicroRNA Let-7b	RNA Gene	20	GC22P046119	15.79724	https://www.genecards.org/cgi-bin/carddisp.pl?gene=MIRLET7B
HTRA1	HtrA Serine Peptidase 1	Protein Coding	44	GC10P122461	15.78774	https://www.genecards.org/cgi-bin/carddisp.pl?gene=HTRA1
OPA1	OPA1 Mitochondrial Dynamin Like GTPase	Protein Coding	45	GC03P193594	15.78479	https://www.genecards.org/cgi-bin/carddisp.pl?gene=OPA1
UBE3A	Ubiquitin Protein Ligase E3A	Protein Coding	48	GC15M025333	15.76962	https://www.genecards.org/cgi-bin/carddisp.pl?gene=UBE3A
DICER1	Dicer 1, Ribonuclease III	Protein Coding	48	GC14M095086	15.76591	https://www.genecards.org/cgi-bin/carddisp.pl?gene=DICER1
PCCA	Propionyl-CoA Carboxylase Subunit Alpha	Protein Coding	48	GC13P100089	15.75922	https://www.genecards.org/cgi-bin/carddisp.pl?gene=PCCA
PVALB	Parvalbumin	Protein Coding	40	GC22M036800	15.75431	https://www.genecards.org/cgi-bin/carddisp.pl?gene=PVALB
MYOT	Myotilin	Protein Coding	42	GC05P137867	15.75248	https://www.genecards.org/cgi-bin/carddisp.pl?gene=MYOT
KEAP1	Kelch Like ECH Associated Protein 1	Protein Coding	48	GC19M010486	15.74058	https://www.genecards.org/cgi-bin/carddisp.pl?gene=KEAP1

IRF3	Interferon Regulatory Factor 3	Protein Coding	47	GC19M049659	15.73547	https://www.genecards.org/cgi-bin/carddisp.pl?gene=IRF3
CD80	CD80 Molecule	Protein Coding	42	GC03M119524	15.73169	https://www.genecards.org/cgi-bin/carddisp.pl?gene=CD80
ANXA2	Annexin A2	Protein Coding	49	GC15M060347	15.72817	https://www.genecards.org/cgi-bin/carddisp.pl?gene=ANXA2
CASP10	Caspase 10	Protein Coding	48	GC02P201182	15.72532	https://www.genecards.org/cgi-bin/carddisp.pl?gene=CASP10
CLCNKB	Chloride Voltage-Gated Channel Kb	Protein Coding	44	GC01P016043	15.72475	https://www.genecards.org/cgi-bin/carddisp.pl?gene=CLCNKB
UGT2B7	UDP Glucuronosyltransferase Family 2 Member B7	Protein Coding	45	GC04P069051	15.71338	https://www.genecards.org/cgi-bin/carddisp.pl?gene=UGT2B7
ALOX12	Arachidonate 12-Lipoxygenase, 12S Type	Protein Coding	44	GC17P006995	15.71093	https://www.genecards.org/cgi-bin/carddisp.pl?gene=ALOX12
AOX1	Aldehyde Oxidase 1	Protein Coding	45	GC02P200585	15.70968	https://www.genecards.org/cgi-bin/carddisp.pl?gene=AOX1
SATB2	SATB Homeobox 2	Protein Coding	45	GC02M199269	15.68483	https://www.genecards.org/cgi-bin/carddisp.pl?gene=SATB2
NDUFS6	NADH:Ubiquinone Oxidoreductase Subunit S6	Protein Coding	45	GC05P001801	15.68081	https://www.genecards.org/cgi-bin/carddisp.pl?gene=NDUFS6
GOLM1	Golgi Membrane Protein 1	Protein Coding	39	GC09M086026	15.67516	https://www.genecards.org/cgi-bin/carddisp.pl?gene=GOLM1
NGFR	Nerve Growth Factor Receptor	Protein Coding	46	GC17P049495	15.67087	https://www.genecards.org/cgi-bin/carddisp.pl?gene=NGFR
ENO1	Enolase 1	Protein Coding	48	GC01M008861	15.64779	https://www.genecards.org/cgi-bin/carddisp.pl?gene=ENO1
MATR3	Matrin 3	Protein Coding	43	GC05P139274	15.64753	https://www.genecards.org/cgi-bin/carddisp.pl?gene=MATR3
CASP7	Caspase 7	Protein Coding	50	GC10P113679	15.64046	https://www.genecards.org/cgi-bin/carddisp.pl?gene=CASP7
BHMT	Betaine--Homocysteine S-Methyltransferase	Protein Coding	43	GC05P079111	15.63945	https://www.genecards.org/cgi-bin/carddisp.pl?gene=BHMT
ATP1A1	ATPase Na+/K+ Transporting Subunit Alpha 1	Protein Coding	51	GC01P116372	15.6326	https://www.genecards.org/cgi-bin/carddisp.pl?gene=ATP1A1
PCCB	Propionyl-CoA Carboxylase Subunit Beta	Protein Coding	48	GC03P136250	15.62269	https://www.genecards.org/cgi-bin/carddisp.pl?gene=PCCB
IARS2	Isoleucyl-TRNA Synthetase 2, Mitochondrial	Protein Coding	42	GC01P220094	15.61557	https://www.genecards.org/cgi-bin/carddisp.pl?gene=IARS2

XRCC3	X-Ray Repair Cross Complementing 3	Protein Coding	43	GC14M103697	15.61494	https://www.genecards.org/cgi-bin/carddisp.pl?gene=XRCC3
ZEB1	Zinc Finger E-Box Binding Homeobox 1	Protein Coding	49	GC10P031318	15.61462	https://www.genecards.org/cgi-bin/carddisp.pl?gene=ZEB1
VAPB	VAMP Associated Protein B And C	Protein Coding	46	GC20P058389	15.61244	https://www.genecards.org/cgi-bin/carddisp.pl?gene=VAPB
FCGR3A	Fc Fragment Of IgG Receptor IIIa	Protein Coding	45	GC01M161541	15.60458	https://www.genecards.org/cgi-bin/carddisp.pl?gene=FCGR3A
MIR149	MicroRNA 149	RNA Gene	20	GC02P240456	15.60336	https://www.genecards.org/cgi-bin/carddisp.pl?gene=MIR149
CHRNA7	Cholinergic Receptor Nicotinic Alpha 7 Subunit	Protein Coding	46	GC15P031923	15.59157	https://www.genecards.org/cgi-bin/carddisp.pl?gene=CHRNA7
LIPG	Lipase G, Endothelial Type	Protein Coding	44	GC18P049560	15.5748	https://www.genecards.org/cgi-bin/carddisp.pl?gene=LIPG
MIR424	MicroRNA 424	RNA Gene	16	GC0XM134651	15.56528	https://www.genecards.org/cgi-bin/carddisp.pl?gene=MIR424
HSPA1A	Heat Shock Protein Family A (Hsp70) Member 1A	Protein Coding	44	GC06P055219	15.56333	https://www.genecards.org/cgi-bin/carddisp.pl?gene=HSPA1A
MERTK	MER Proto-Oncogene, Tyrosine Kinase	Protein Coding	51	GC02P111898	15.56089	https://www.genecards.org/cgi-bin/carddisp.pl?gene=MERTK
HDAC6	Histone Deacetylase 6	Protein Coding	52	GC0XP048801	15.55583	https://www.genecards.org/cgi-bin/carddisp.pl?gene=HDAC6
LOC111674475	CFTR Intron 11 Enhancer	Biological Region	2	GC07P117587	15.54835	https://www.genecards.org/cgi-bin/carddisp.pl?gene=LOC111674475
GABRD	Gamma-Aminobutyric Acid Type A Receptor Subunit Delta	Protein Coding	46	GC01P002019	15.53534	https://www.genecards.org/cgi-bin/carddisp.pl?gene=GABRD
CCL20	C-C Motif Chemokine Ligand 20	Protein Coding	44	GC02P227814	15.53394	https://www.genecards.org/cgi-bin/carddisp.pl?gene=CCL20
SFTPD	Surfactant Protein D	Protein Coding	44	GC10M079937	15.52863	https://www.genecards.org/cgi-bin/carddisp.pl?gene=SFTPD
RNASEH2C	Ribonuclease H2 Subunit C	Protein Coding	40	GC11M065714	15.52587	https://www.genecards.org/cgi-bin/carddisp.pl?gene=RNASEH2C
ANKK1	Ankyrin Repeat And Kinase Domain Containing 1	Protein Coding	38	GC11P113387	15.52338	https://www.genecards.org/cgi-bin/carddisp.pl?gene=ANKK1
TKFC	Triokinase And FMN Cyclase	Protein Coding	40	GC11P061334	15.50475	https://www.genecards.org/cgi-bin/carddisp.pl?gene=TKFC

RNASEH2A	Ribonuclease H2 Subunit A	Protein Coding	44	GC19P012896	15.49743	https://www.genecards.org/cgi-bin/carddisp.pl?gene=RNASEH2A
SAMHD1	SAM And HD Domain Containing Deoxynucleoside Triphosphate Triphosphohydrolase 1	Protein Coding	43	GC20M036890	15.49395	https://www.genecards.org/cgi-bin/carddisp.pl?gene=SAMHD1
P2RY12	Purinergic Receptor P2Y12	Protein Coding	49	GC03M151336	15.48017	https://www.genecards.org/cgi-bin/carddisp.pl?gene=P2RY12
NDUFB3	NADH:Ubiquinone Oxidoreductase Subunit B3	Protein Coding	43	GC02P201071	15.47583	https://www.genecards.org/cgi-bin/carddisp.pl?gene=NDUFB3
SPR	Sepiapterin Reductase	Protein Coding	51	GC02P072850	15.45502	https://www.genecards.org/cgi-bin/carddisp.pl?gene=SPR
TGM2	Transglutaminase 2	Protein Coding	48	GC20M038127	15.44772	https://www.genecards.org/cgi-bin/carddisp.pl?gene=TGM2
LAMA2	Laminin Subunit Alpha 2	Protein Coding	44	GC06P128863	15.4388	https://www.genecards.org/cgi-bin/carddisp.pl?gene=LAMA2
IL11	Interleukin 11	Protein Coding	41	GC19M055364	15.43434	https://www.genecards.org/cgi-bin/carddisp.pl?gene=IL11
ACOX3	Acyl-CoA Oxidase 3, Pristanoyl	Protein Coding	41	GC04M008380	15.42329	https://www.genecards.org/cgi-bin/carddisp.pl?gene=ACOX3
NKX2-1	NK2 Homeobox 1	Protein Coding	47	GC14M036516	15.4176	https://www.genecards.org/cgi-bin/carddisp.pl?gene=NKX2-1
NDUFA12	NADH:Ubiquinone Oxidoreductase Subunit A12	Protein Coding	45	GC12M094898	15.39772	https://www.genecards.org/cgi-bin/carddisp.pl?gene=NDUFA12
STX1A	Syntaxin 1A	Protein Coding	48	GC07M073700	15.39687	https://www.genecards.org/cgi-bin/carddisp.pl?gene=STX1A
DVL1	Dishevelled Segment Polarity Protein 1	Protein Coding	48	GC01M001335	15.39504	https://www.genecards.org/cgi-bin/carddisp.pl?gene=DVL1
HPGD	15-Hydroxyprostaglandin Dehydrogenase	Protein Coding	48	GC04M174490	15.39483	https://www.genecards.org/cgi-bin/carddisp.pl?gene=HPGD
ONECUT1	One Cut Homeobox 1	Protein Coding	41	GC15M067272	15.38384	https://www.genecards.org/cgi-bin/carddisp.pl?gene=ONECUT1
LIFR	LIF Receptor Subunit Alpha	Protein Coding	47	GC05M038475	15.38278	https://www.genecards.org/cgi-bin/carddisp.pl?gene=LIFR
ATRIP	ATR Interacting Protein	Protein Coding	43	GC03P048449	15.37511	https://www.genecards.org/cgi-bin/carddisp.pl?gene=ATRIP

SMARCAL1	SWI/SNF Related, Matrix Associated, Actin Dependent Regulator Of Chromatin, Subfamily A Like 1	Protein Coding	44	GC02P216412	15.37228	https://www.genecards.org/cgi-bin/carddisp.pl?gene=SMARCAL1
ISG15	ISG15 Ubiquitin Like Modifier	Protein Coding	47	GC01P001001	15.36656	https://www.genecards.org/cgi-bin/carddisp.pl?gene=ISG15
FANCD2	FA Complementation Group D2	Protein Coding	47	GC03P010026	15.36095	https://www.genecards.org/cgi-bin/carddisp.pl?gene=FANCD2
H6PD	Hexose-6-Phosphate Dehydrogenase/Glucose 1-Dehydrogenase	Protein Coding	43	GC01P009234	15.35358	https://www.genecards.org/cgi-bin/carddisp.pl?gene=H6PD
PROS1	Protein S	Protein Coding	48	GC03M093873	15.35192	https://www.genecards.org/cgi-bin/carddisp.pl?gene=PROS1
SMAD7	SMAD Family Member 7	Protein Coding	44	GC18M048919	15.34348	https://www.genecards.org/cgi-bin/carddisp.pl?gene=SMAD7
MGLL	Monoglyceride Lipase	Protein Coding	46	GC03M127689	15.33552	https://www.genecards.org/cgi-bin/carddisp.pl?gene=MGLL
EGR1	Early Growth Response 1	Protein Coding	44	GC05P138465	15.3355	https://www.genecards.org/cgi-bin/carddisp.pl?gene=EGR1
FDFT1	Farnesyl-Diphosphate Farnesyltransferase 1	Protein Coding	46	GC08P011795	15.3271	https://www.genecards.org/cgi-bin/carddisp.pl?gene=FDFT1
ADCYAP1	Adenylate Cyclase Activating Polypeptide 1	Protein Coding	43	GC18P000895	15.32689	https://www.genecards.org/cgi-bin/carddisp.pl?gene=ADCYAP1
ETS1	ETS Proto-Oncogene 1, Transcription Factor	Protein Coding	49	GC11M128458	15.3215	https://www.genecards.org/cgi-bin/carddisp.pl?gene=ETS1
TNFRSF10A	TNF Receptor Superfamily Member 10a	Protein Coding	45	GC08M023190	15.30898	https://www.genecards.org/cgi-bin/carddisp.pl?gene=TNFRSF10A
STAT5A	Signal Transducer And Activator Of Transcription 5A	Protein Coding	47	GC17P042287	15.30695	https://www.genecards.org/cgi-bin/carddisp.pl?gene=STAT5A
KIAA0319L	KIAA0319 Like	Protein Coding	37	GC01M035433	15.29306	https://www.genecards.org/cgi-bin/carddisp.pl?gene=KIAA0319L
DLG4	Discs Large MAGUK Scaffold Protein 4	Protein Coding	48	GC17M007189	15.28131	https://www.genecards.org/cgi-bin/carddisp.pl?gene=DLG4

OXA1L	OXA1L Mitochondrial Inner Membrane Protein	Protein Coding	40	GC14P022766	15.26388	https://www.genecards.org/cgi-bin/carddisp.pl?gene=OXA1L
SLC16A2	Solute Carrier Family 16 Member 2	Protein Coding	46	GC0XP074439	15.26246	https://www.genecards.org/cgi-bin/carddisp.pl?gene=SLC16A2
PTK2	Protein Tyrosine Kinase 2	Protein Coding	48	GC08M140657	15.26065	https://www.genecards.org/cgi-bin/carddisp.pl?gene=PTK2
ABCC1	ATP Binding Cassette Subfamily C Member 1	Protein Coding	50	GC16P015949	15.2595	https://www.genecards.org/cgi-bin/carddisp.pl?gene=ABCC1
CSF1R	Colony Stimulating Factor 1 Receptor	Protein Coding	52	GC05M150053	15.25608	https://www.genecards.org/cgi-bin/carddisp.pl?gene=CSF1R
GAD2	Glutamate Decarboxylase 2	Protein Coding	47	GC10P026216	15.25606	https://www.genecards.org/cgi-bin/carddisp.pl?gene=GAD2
ARV1	ARV1 Homolog, Fatty Acid Homeostasis Modulator	Protein Coding	37	GC01P230978	15.24654	https://www.genecards.org/cgi-bin/carddisp.pl?gene=ARV1
BMPR1A	Bone Morphogenetic Protein Receptor Type 1A	Protein Coding	51	GC10P086756	15.24649	https://www.genecards.org/cgi-bin/carddisp.pl?gene=BMPR1A
TJP1	Tight Junction Protein 1	Protein Coding	44	GC15M029699	15.22886	https://www.genecards.org/cgi-bin/carddisp.pl?gene=TJP1
NTF3	Neurotrophin 3	Protein Coding	43	GC12P005432	15.22458	https://www.genecards.org/cgi-bin/carddisp.pl?gene=NTF3
CTC1	CST Telomere Replication Complex Component 1	Protein Coding	35	GC17M009204	15.21308	https://www.genecards.org/cgi-bin/carddisp.pl?gene=CTC1
COX6B1	Cytochrome C Oxidase Subunit 6B1	Protein Coding	43	GC19P040358	15.21075	https://www.genecards.org/cgi-bin/carddisp.pl?gene=COX6B1
MIR499A	MicroRNA 499a	RNA Gene	21	GC20P034990	15.19535	https://www.genecards.org/cgi-bin/carddisp.pl?gene=MIR499A
APTX	Aprataxin	Protein Coding	45	GC09M032886	15.18989	https://www.genecards.org/cgi-bin/carddisp.pl?gene=APTX
GCLC	Glutamate-Cysteine Ligase Catalytic Subunit	Protein Coding	44	GC06M053497	15.1827	https://www.genecards.org/cgi-bin/carddisp.pl?gene=GCLC
LOC113664106	CFTR Intron 2 DNase I Hypersensitive Site	Biological Region	2	GC07P117508	15.17766	https://www.genecards.org/cgi-bin/carddisp.pl?gene=LOC113664106

COL18A1	Collagen Type XVIII Alpha 1 Chain	Protein Coding	45	GC21P045405	15.17525	https://www.genecards.org/cgi-bin/carddisp.pl?gene=COL18A1
CDKL5	Cyclin Dependent Kinase Like 5	Protein Coding	44	GC0XP018425	15.17344	https://www.genecards.org/cgi-bin/carddisp.pl?gene=CDKL5
UBE2L3	Ubiquitin Conjugating Enzyme E2 L3	Protein Coding	46	GC22P021549	15.1722	https://www.genecards.org/cgi-bin/carddisp.pl?gene=UBE2L3
RB1CC1	RB1 Inducible Coiled-Coil 1	Protein Coding	43	GC08M052622	15.16649	https://www.genecards.org/cgi-bin/carddisp.pl?gene=RB1CC1
DHCR7	7-Dehydrocholesterol Reductase	Protein Coding	47	GC11M071428	15.15968	https://www.genecards.org/cgi-bin/carddisp.pl?gene=DHCR7
MIR342	MicroRNA 342	RNA Gene	19	GC14P100109	15.14464	https://www.genecards.org/cgi-bin/carddisp.pl?gene=MIR342
MEF2A	Myocyte Enhancer Factor 2A	Protein Coding	48	GC15P099565	15.14463	https://www.genecards.org/cgi-bin/carddisp.pl?gene=MEF2A
MIR185	MicroRNA 185	RNA Gene	21	GC22P020106	15.1385	https://www.genecards.org/cgi-bin/carddisp.pl?gene=MIR185
UBD	Ubiquitin D	Protein Coding	39	GC06M046741	15.11981	https://www.genecards.org/cgi-bin/carddisp.pl?gene=UBD
NEAT1	Nuclear Paraspeckle Assembly Transcript 1	RNA Gene	23	GC11P066222	15.11348	https://www.genecards.org/cgi-bin/carddisp.pl?gene=NEAT1
PGAM2	Phosphoglycerate Mutase 2	Protein Coding	45	GC07M044062	15.10423	https://www.genecards.org/cgi-bin/carddisp.pl?gene=PGAM2
PTRH2	Peptidyl-TRNA Hydrolase 2	Protein Coding	45	GC17M059674	15.09198	https://www.genecards.org/cgi-bin/carddisp.pl?gene=PTRH2
TBCE	Tubulin Folding Cofactor E	Protein Coding	39	GC01P235357	15.0892	https://www.genecards.org/cgi-bin/carddisp.pl?gene=TBCE
DAG1	Dystroglycan 1	Protein Coding	47	GC03P049482	15.08203	https://www.genecards.org/cgi-bin/carddisp.pl?gene=DAG1
MC4R	Melanocortin 4 Receptor	Protein Coding	47	GC18M060371	15.08177	https://www.genecards.org/cgi-bin/carddisp.pl?gene=MC4R
AIPL1	Aryl Hydrocarbon Receptor Interacting Protein Like 1	Protein Coding	43	GC17M006393	15.0814	https://www.genecards.org/cgi-bin/carddisp.pl?gene=AIPL1
COX8A	Cytochrome C Oxidase Subunit 8A	Protein Coding	41	GC11P063994	15.06893	https://www.genecards.org/cgi-bin/carddisp.pl?gene=COX8A
MIR486-1	MicroRNA 486-1	RNA Gene	16	GC08M041660	15.06324	https://www.genecards.org/cgi-bin/carddisp.pl?gene=MIR486-1
WDPCP	WD Repeat Containing Planar Cell Polarity Effector	Protein Coding	37	GC02M063121	15.05699	https://www.genecards.org/cgi-bin/carddisp.pl?gene=WDPCP

SYNE1	Spectrin Repeat Containing Nuclear Envelope Protein 1	Protein Coding	41	GC06M152121	15.05679	https://www.genecards.org/cgi-bin/carddisp.pl?gene=SYNE1
MIR15B	MicroRNA 15b	RNA Gene	19	GC03P160404	15.04995	https://www.genecards.org/cgi-bin/carddisp.pl?gene=MIR15B
STIM1	Stromal Interaction Molecule 1	Protein Coding	49	GC11P003855	15.04688	https://www.genecards.org/cgi-bin/carddisp.pl?gene=STIM1
CYP4F2	Cytochrome P450 Family 4 Subfamily F Member 2	Protein Coding	45	GC19M015878	15.04514	https://www.genecards.org/cgi-bin/carddisp.pl?gene=CYP4F2
UQCRC2	Ubiquinol-Cytochrome C Reductase Core Protein 2	Protein Coding	47	GC16P021963	15.035	https://www.genecards.org/cgi-bin/carddisp.pl?gene=UQCRC2
CDC25A	Cell Division Cycle 25A	Protein Coding	48	GC03M048173	15.0292	https://www.genecards.org/cgi-bin/carddisp.pl?gene=CDC25A
HPD	4-Hydroxyphenylpyruvate Dioxygenase	Protein Coding	46	GC12M121839	15.02463	https://www.genecards.org/cgi-bin/carddisp.pl?gene=HPD
CD68	CD68 Molecule	Protein Coding	40	GC17P007579	15.0206	https://www.genecards.org/cgi-bin/carddisp.pl?gene=CD68
ERCC3	ERCC Excision Repair 3, TFIIH Core Complex Helicase Subunit	Protein Coding	48	GC02M127257	15.01849	https://www.genecards.org/cgi-bin/carddisp.pl?gene=ERCC3
TANGO2	Transport And Golgi Organization 2 Homolog	Protein Coding	36	GC22P020017	15.0146	https://www.genecards.org/cgi-bin/carddisp.pl?gene=TANGO2
MED12	Mediator Complex Subunit 12	Protein Coding	45	GC0XP071118	14.99786	https://www.genecards.org/cgi-bin/carddisp.pl?gene=MED12
UNC13D	Unc-13 Homolog D	Protein Coding	44	GC17M075827	14.99652	https://www.genecards.org/cgi-bin/carddisp.pl?gene=UNC13D
IRAK1	Interleukin 1 Receptor Associated Kinase 1	Protein Coding	50	GC0XM154010	14.98808	https://www.genecards.org/cgi-bin/carddisp.pl?gene=IRAK1
CYP11B2	Cytochrome P450 Family 11 Subfamily B Member 2	Protein Coding	48	GC08M142910	14.97955	https://www.genecards.org/cgi-bin/carddisp.pl?gene=CYP11B2
GRIA2	Glutamate Ionotropic Receptor AMPA Type Subunit 2	Protein Coding	51	GC04P157204	14.97819	https://www.genecards.org/cgi-bin/carddisp.pl?gene=GRIA2
GSN	Gelsolin	Protein Coding	49	GC09P121201	14.95962	https://www.genecards.org/cgi-bin/carddisp.pl?gene=GSN
BBS9	Bardet-Biedl Syndrome 9	Protein Coding	39	GC07P033112	14.95851	https://www.genecards.org/cgi-bin/carddisp.pl?gene=BBS9

GK	Glycerol Kinase	Protein Coding	48	GC0XP030747	14.95562	https://www.genecards.org/cgi-bin/carddisp.pl?gene=GK
FARSA	Phenylalanyl-TRNA Synthetase Subunit Alpha	Protein Coding	41	GC19M012922	14.95508	https://www.genecards.org/cgi-bin/carddisp.pl?gene=FARSA
CHRM3	Cholinergic Receptor Muscarinic 3	Protein Coding	50	GC01P239386	14.94667	https://www.genecards.org/cgi-bin/carddisp.pl?gene=CHRM3
VEGFC	Vascular Endothelial Growth Factor C	Protein Coding	48	GC04M176683	14.9419	https://www.genecards.org/cgi-bin/carddisp.pl?gene=VEGFC
TGM1	Transglutaminase 1	Protein Coding	46	GC14M024249	14.93787	https://www.genecards.org/cgi-bin/carddisp.pl?gene=TGM1
AURKA	Aurora Kinase A	Protein Coding	52	GC20M056370	14.93218	https://www.genecards.org/cgi-bin/carddisp.pl?gene=AURKA
MTFMT	Mitochondrial Methionyl-TRNA Formyltransferase	Protein Coding	42	GC15M065001	14.9236	https://www.genecards.org/cgi-bin/carddisp.pl?gene=MTFMT
SERPINA7	Serpin Family A Member 7	Protein Coding	40	GC0XM106032	14.91298	https://www.genecards.org/cgi-bin/carddisp.pl?gene=SERPINA7
ACSS2	Acyl-CoA Synthetase Short Chain Family Member 2	Protein Coding	44	GC20P034873	14.90542	https://www.genecards.org/cgi-bin/carddisp.pl?gene=ACSS2
IMPG2	Interphotoreceptor Matrix Proteoglycan 2	Protein Coding	38	GC03M101222	14.90423	https://www.genecards.org/cgi-bin/carddisp.pl?gene=IMPG2
ARID1A	AT-Rich Interaction Domain 1A	Protein Coding	45	GC01P026693	14.89883	https://www.genecards.org/cgi-bin/carddisp.pl?gene=ARID1A
TXN	Thioredoxin	Protein Coding	46	GC09M110243	14.89429	https://www.genecards.org/cgi-bin/carddisp.pl?gene=TXN
MGAM	Maltase-Glucoamylase	Protein Coding	42	GC07P145925	14.89125	https://www.genecards.org/cgi-bin/carddisp.pl?gene=MGAM
CREB3L3	CAMP Responsive Element Binding Protein 3 Like 3	Protein Coding	40	GC19P004153	14.88233	https://www.genecards.org/cgi-bin/carddisp.pl?gene=CREB3L3
TNFSF12	TNF Superfamily Member 12	Protein Coding	41	GC17P009104	14.87695	https://www.genecards.org/cgi-bin/carddisp.pl?gene=TNFSF12
AXIN2	Axin 2	Protein Coding	49	GC17M065528	14.87438	https://www.genecards.org/cgi-bin/carddisp.pl?gene=AXIN2
SIGMAR1	Sigma Non-Opioid Intracellular Receptor 1	Protein Coding	48	GC09M034634	14.87434	https://www.genecards.org/cgi-bin/carddisp.pl?gene=SIGMAR1
SNAP29	Synaptosome Associated Protein 29	Protein Coding	43	GC22P020859	14.87272	https://www.genecards.org/cgi-bin/carddisp.pl?gene=SNAP29

TYROBP	Transmembrane Immune Signaling Adaptor TYROBP	Protein Coding	44	GC19M035904	14.86237	https://www.genecards.org/cgi-bin/carddisp.pl?gene=TYROBP
PDE5A	Phosphodiesterase 5A	Protein Coding	45	GC04M119494	14.862	https://www.genecards.org/cgi-bin/carddisp.pl?gene=PDE5A
KCTD17	Potassium Channel Tetramerization Domain Containing 17	Protein Coding	40	GC22P037051	14.85376	https://www.genecards.org/cgi-bin/carddisp.pl?gene=KCTD17
PRDX6	Peroxiredoxin 6	Protein Coding	47	GC01P173477	14.83703	https://www.genecards.org/cgi-bin/carddisp.pl?gene=PRDX6
PRSS2	Serine Protease 2	Protein Coding	41	GC07P145798	14.82199	https://www.genecards.org/cgi-bin/carddisp.pl?gene=PRSS2
MCL1	MCL1 Apoptosis Regulator, BCL2 Family Member	Protein Coding	48	GC01M150561	14.80123	https://www.genecards.org/cgi-bin/carddisp.pl?gene=MCL1
ACSM2B	Acyl-CoA Synthetase Medium Chain Family Member 2B	Protein Coding	36	GC16M020547	14.79	https://www.genecards.org/cgi-bin/carddisp.pl?gene=ACSM2B
IGHE	Immunoglobulin Heavy Constant Epsilon	Protein Coding	26	GC14M110308	14.78862	https://www.genecards.org/cgi-bin/carddisp.pl?gene=IGHE
HSD17B12	Hydroxysteroid 17-Beta Dehydrogenase 12	Protein Coding	41	GC11P043660	14.78369	https://www.genecards.org/cgi-bin/carddisp.pl?gene=HSD17B12
IFNAR2	Interferon Alpha And Beta Receptor Subunit 2	Protein Coding	49	GC21P033229	14.78071	https://www.genecards.org/cgi-bin/carddisp.pl?gene=IFNAR2
ANKLE2	Ankyrin Repeat And LEM Domain Containing 2	Protein Coding	39	GC12M132725	14.76638	https://www.genecards.org/cgi-bin/carddisp.pl?gene=ANKLE2
PML	PML Nuclear Body Scaffold	Protein Coding	46	GC15P073994	14.76556	https://www.genecards.org/cgi-bin/carddisp.pl?gene=PML
PSMB4	Proteasome 20S Subunit Beta 4	Protein Coding	46	GC01P151372	14.75507	https://www.genecards.org/cgi-bin/carddisp.pl?gene=PSMB4
NUBPL	Nucleotide Binding Protein Like	Protein Coding	40	GC14P031489	14.7522	https://www.genecards.org/cgi-bin/carddisp.pl?gene=NUBPL
SLC29A3	Solute Carrier Family 29 Member 3	Protein Coding	44	GC10P071320	14.75152	https://www.genecards.org/cgi-bin/carddisp.pl?gene=SLC29A3
NRXN1	Neurexin 1	Protein Coding	48	GC02M049918	14.7514	https://www.genecards.org/cgi-bin/carddisp.pl?gene=NRXN1
TMEM199	Transmembrane Protein 199	Protein Coding	34	GC17P029324	14.74572	https://www.genecards.org/cgi-bin/carddisp.pl?gene=TMEM199

HTR1B	5-Hydroxytryptamine Receptor 1B	Protein Coding	45	GC06M077478	14.74036	https://www.genecards.org/cgi-bin/carddisp.pl?gene=HTR1B
SC5D	Sterol-C5-Desaturase	Protein Coding	40	GC11P121292	14.73855	https://www.genecards.org/cgi-bin/carddisp.pl?gene=SC5D
GABBR1	Gamma-Aminobutyric Acid Type B Receptor Subunit 1	Protein Coding	48	GC06M029555	14.73711	https://www.genecards.org/cgi-bin/carddisp.pl?gene=GABBR1
PRKD1	Protein Kinase D1	Protein Coding	51	GC14M029576	14.73232	https://www.genecards.org/cgi-bin/carddisp.pl?gene=PRKD1
RAB3GAP1	RAB3 GTPase Activating Protein Catalytic Subunit 1	Protein Coding	41	GC02P135052	14.72537	https://www.genecards.org/cgi-bin/carddisp.pl?gene=RAB3GAP1
CLEC7A	C-Type Lectin Domain Containing 7A	Protein Coding	45	GC12M015684	14.7238	https://www.genecards.org/cgi-bin/carddisp.pl?gene=CLEC7A
KLF6	Kruppel Like Factor 6	Protein Coding	44	GC10M003779	14.71518	https://www.genecards.org/cgi-bin/carddisp.pl?gene=KLF6
KMT2B	Lysine Methyltransferase 2B	Protein Coding	39	GC19P040361	14.69971	https://www.genecards.org/cgi-bin/carddisp.pl?gene=KMT2B
ITGA2	Integrin Subunit Alpha 2	Protein Coding	47	GC05P052989	14.69604	https://www.genecards.org/cgi-bin/carddisp.pl?gene=ITGA2
RDH5	Retinol Dehydrogenase 5	Protein Coding	47	GC12P055720	14.67124	https://www.genecards.org/cgi-bin/carddisp.pl?gene=RDH5
PFN1	Profilin 1	Protein Coding	48	GC17M004945	14.66519	https://www.genecards.org/cgi-bin/carddisp.pl?gene=PFN1
AUTS2	Activator Of Transcription And Developmental Regulator AUTS2	Protein Coding	40	GC07P069598	14.66234	https://www.genecards.org/cgi-bin/carddisp.pl?gene=AUTS2
DNAH11	Dynein Axonemal Heavy Chain 11	Protein Coding	42	GC07P021543	14.65823	https://www.genecards.org/cgi-bin/carddisp.pl?gene=DNAH11
MT-ND4L	Mitochondrially Encoded NADH:Ubiquinone Oxidoreductase Core Subunit 4L	Protein Coding	29	GCMTP010472	14.65598	https://www.genecards.org/cgi-bin/carddisp.pl?gene=MT-ND4L
RBP3	Retinol Binding Protein 3	Protein Coding	41	GC10P047348	14.65096	https://www.genecards.org/cgi-bin/carddisp.pl?gene=RBP3
UBQLN2	Ubiquilin 2	Protein Coding	44	GC0XP056563	14.63818	https://www.genecards.org/cgi-bin/carddisp.pl?gene=UBQLN2
MIR196A1	MicroRNA 196a-1	RNA Gene	20	GC17M048632	14.63727	https://www.genecards.org/cgi-bin/carddisp.pl?gene=MIR196A1

LOC113633877	CFTR Intron 19 DNase I Hypersensitive Site	Biological Region	2	GC07P117639	14.63089	https://www.genecards.org/cgi-bin/carddisp.pl?gene=LOC113633877
IL6ST	Interleukin 6 Cytokine Family Signal Transducer	Protein Coding	47	GC05M055935	14.59988	https://www.genecards.org/cgi-bin/carddisp.pl?gene=IL6ST
TMEM237	Transmembrane Protein 237	Protein Coding	37	GC02M201620	14.59057	https://www.genecards.org/cgi-bin/carddisp.pl?gene=TMEM237
MECOM	MDS1 And EVI1 Complex Locus	Protein Coding	48	GC03M169083	14.58373	https://www.genecards.org/cgi-bin/carddisp.pl?gene=MECOM
BSG	Basigin (Ok Blood Group)	Protein Coding	44	GC19P000571	14.58112	https://www.genecards.org/cgi-bin/carddisp.pl?gene=BSG
GNPAT	Glyceronephosphate O-Acyltransferase	Protein Coding	46	GC01P231241	14.57617	https://www.genecards.org/cgi-bin/carddisp.pl?gene=GNPAT
MIR146B	MicroRNA 146b	RNA Gene	19	GC10P102436	14.57183	https://www.genecards.org/cgi-bin/carddisp.pl?gene=MIR146B
ALS2	Alsin Rho Guanine Nucleotide Exchange Factor ALS2	Protein Coding	45	GC02M201701	14.57145	https://www.genecards.org/cgi-bin/carddisp.pl?gene=ALS2
LRP2	LDL Receptor Related Protein 2	Protein Coding	47	GC02M169127	14.5696	https://www.genecards.org/cgi-bin/carddisp.pl?gene=LRP2
HCFC1	Host Cell Factor C1	Protein Coding	47	GC0XM153947	14.56435	https://www.genecards.org/cgi-bin/carddisp.pl?gene=HCFC1
PYY	Peptide YY	Protein Coding	43	GC17M043952	14.56344	https://www.genecards.org/cgi-bin/carddisp.pl?gene=PYY
TCTN1	Tectonic Family Member 1	Protein Coding	37	GC12P110614	14.55394	https://www.genecards.org/cgi-bin/carddisp.pl?gene=TCTN1
TRB	T Cell Receptor Beta Locus	Protein Coding	17	GC07P146007	14.55346	https://www.genecards.org/cgi-bin/carddisp.pl?gene=TRB
TMEM106B	Transmembrane Protein 106B	Protein Coding	39	GC07P012218	14.54716	https://www.genecards.org/cgi-bin/carddisp.pl?gene=TMEM106B
GPC1	Glypican 1	Protein Coding	44	GC02P240435	14.53975	https://www.genecards.org/cgi-bin/carddisp.pl?gene=GPC1
GSTA1	Glutathione S-Transferase Alpha 1	Protein Coding	43	GC06M052791	14.53854	https://www.genecards.org/cgi-bin/carddisp.pl?gene=GSTA1
SOAT2	Sterol O-Acyltransferase 2	Protein Coding	44	GC12P053103	14.53415	https://www.genecards.org/cgi-bin/carddisp.pl?gene=SOAT2
APOD	Apolipoprotein D	Protein Coding	43	GC03M195568	14.5337	https://www.genecards.org/cgi-bin/carddisp.pl?gene=APOD
SDC1	Syndecan 1	Protein Coding	44	GC02M020200	14.53241	https://www.genecards.org/cgi-bin/carddisp.pl?gene=SDC1
LAMP1	Lysosomal Associated Membrane Protein 1	Protein Coding	43	GC13P113297	14.52898	https://www.genecards.org/cgi-bin/carddisp.pl?gene=LAMP1

ACAT1	Acetyl-CoA Acetyltransferase 1	Protein Coding	50	GC11P108121	14.51504	https://www.genecards.org/cgi-bin/carddisp.pl?gene=ACAT1
MAP2	Microtubule Associated Protein 2	Protein Coding	43	GC02P209424	14.49306	https://www.genecards.org/cgi-bin/carddisp.pl?gene=MAP2
SNAI1	Snail Family Transcriptional Repressor 1	Protein Coding	45	GC20P049982	14.49049	https://www.genecards.org/cgi-bin/carddisp.pl?gene=SNAI1
MIR24-1	MicroRNA 24-1	RNA Gene	19	GC09P095086	14.48898	https://www.genecards.org/cgi-bin/carddisp.pl?gene=MIR24-1
CDK6	Cyclin Dependent Kinase 6	Protein Coding	54	GC07M092604	14.48369	https://www.genecards.org/cgi-bin/carddisp.pl?gene=CDK6
GCGR	Glucagon Receptor	Protein Coding	48	GC17P081804	14.47223	https://www.genecards.org/cgi-bin/carddisp.pl?gene=GCGR
MIR106A	MicroRNA 106a	RNA Gene	18	GC0XM134219	14.46877	https://www.genecards.org/cgi-bin/carddisp.pl?gene=MIR106A
KIF7	Kinesin Family Member 7	Protein Coding	40	GC15M089608	14.4617	https://www.genecards.org/cgi-bin/carddisp.pl?gene=KIF7
MT-TT	Mitochondrially Encoded TRNA-Thr (ACN)	RNA Gene	15	GCMTP015890	14.45234	https://www.genecards.org/cgi-bin/carddisp.pl?gene=MT-TT
TOMM40	Translocase Of Outer Mitochondrial Membrane 40	Protein Coding	41	GC19P044890	14.43404	https://www.genecards.org/cgi-bin/carddisp.pl?gene=TOMM40
VKORC1	Vitamin K Epoxide Reductase Complex Subunit 1	Protein Coding	47	GC16M031116	14.41591	https://www.genecards.org/cgi-bin/carddisp.pl?gene=VKORC1
HLA-DPA1	Major Histocompatibility Complex, Class II, DP Alpha 1	Protein Coding	41	GC06M033064	14.41436	https://www.genecards.org/cgi-bin/carddisp.pl?gene=HLA-DPA1
MOGS	Mannosyl-Oligosaccharide Glucosidase	Protein Coding	43	GC02M074461	14.40893	https://www.genecards.org/cgi-bin/carddisp.pl?gene=MOGS
TBL1XR1	TBL1X Receptor 1	Protein Coding	45	GC03M177019	14.40362	https://www.genecards.org/cgi-bin/carddisp.pl?gene=TBL1XR1
SULT2B1	Sulfotransferase Family 2B Member 1	Protein Coding	46	GC19P048552	14.40354	https://www.genecards.org/cgi-bin/carddisp.pl?gene=SULT2B1
VCL	Vinculin	Protein Coding	48	GC10P073995	14.40218	https://www.genecards.org/cgi-bin/carddisp.pl?gene=VCL
STEAP3	STEAP3 Metalloreductase	Protein Coding	45	GC02P119222	14.40119	https://www.genecards.org/cgi-bin/carddisp.pl?gene=STEAP3
DGKE	Diacylglycerol Kinase Epsilon	Protein Coding	49	GC17P056834	14.39715	https://www.genecards.org/cgi-bin/carddisp.pl?gene=DGKE

PAFAH1B1	Platelet Activating Factor Acetylhydrolase 1b Regulatory Subunit 1	Protein Coding	45	GC17P002593	14.39711	https://www.genecards.org/cgi-bin/carddisp.pl?gene=PAFAH1B1
BCL10	BCL10 Immune Signaling Adaptor	Protein Coding	45	GC01M085265	14.39367	https://www.genecards.org/cgi-bin/carddisp.pl?gene=BCL10
TUBB4A	Tubulin Beta 4A Class IVa	Protein Coding	45	GC19M006496	14.39009	https://www.genecards.org/cgi-bin/carddisp.pl?gene=TUBB4A
HEPACAM	Hepatic And Glial Cell Adhesion Molecule	Protein Coding	40	GC11M124919	14.38429	https://www.genecards.org/cgi-bin/carddisp.pl?gene=HEPACAM
CXCL2	C-X-C Motif Chemokine Ligand 2	Protein Coding	41	GC04M074097	14.37961	https://www.genecards.org/cgi-bin/carddisp.pl?gene=CXCL2
NME1	NME/NM23 Nucleoside Diphosphate Kinase 1	Protein Coding	47	GC17P051154	14.37709	https://www.genecards.org/cgi-bin/carddisp.pl?gene=NME1
TACR1	Tachykinin Receptor 1	Protein Coding	47	GC02M075010	14.37669	https://www.genecards.org/cgi-bin/carddisp.pl?gene=TACR1
MIR199B	MicroRNA 199b	RNA Gene	19	GC09M128244	14.37488	https://www.genecards.org/cgi-bin/carddisp.pl?gene=MIR199B
PIK3CB	Phosphatidylinositol-4,5-Bisphosphate 3-Kinase Catalytic Subunit Beta	Protein Coding	50	GC03M138652	14.36272	https://www.genecards.org/cgi-bin/carddisp.pl?gene=PIK3CB
ALOX15	Arachidonate 15-Lipoxygenase	Protein Coding	45	GC17M004630	14.33671	https://www.genecards.org/cgi-bin/carddisp.pl?gene=ALOX15
HRH2	Histamine Receptor H2	Protein Coding	45	GC05P175659	14.32616	https://www.genecards.org/cgi-bin/carddisp.pl?gene=HRH2
TNFRSF13B	TNF Receptor Superfamily Member 13B	Protein Coding	47	GC17M016929	14.3109	https://www.genecards.org/cgi-bin/carddisp.pl?gene=TNFRSF13B
FOXO3	Forkhead Box O3	Protein Coding	44	GC06P108559	14.30285	https://www.genecards.org/cgi-bin/carddisp.pl?gene=FOXO3
HACL1	2-Hydroxyacyl-CoA Lyase 1	Protein Coding	40	GC03M016952	14.30033	https://www.genecards.org/cgi-bin/carddisp.pl?gene=HACL1
TAC1	Tachykinin Precursor 1	Protein Coding	44	GC07P097731	14.29236	https://www.genecards.org/cgi-bin/carddisp.pl?gene=TAC1
EWSR1	EWS RNA Binding Protein 1	Protein Coding	44	GC22P029269	14.28753	https://www.genecards.org/cgi-bin/carddisp.pl?gene=EWSR1
MYH14	Myosin Heavy Chain 14	Protein Coding	46	GC19P050192	14.2849	https://www.genecards.org/cgi-bin/carddisp.pl?gene=MYH14
LMNB2	Lamin B2	Protein Coding	44	GC19M002470	14.27617	https://www.genecards.org/cgi-bin/carddisp.pl?gene=LMNB2

CYP21A2	Cytochrome P450 Family 21 Subfamily A Member 2	Protein Coding	45	GC06P055225	14.27437	https://www.genecards.org/cgi-bin/carddisp.pl?gene=CYP21A2
IL17F	Interleukin 17F	Protein Coding	42	GC06M052209	14.27191	https://www.genecards.org/cgi-bin/carddisp.pl?gene=IL17F
CPA1	Carboxypeptidase A1	Protein Coding	45	GC07P130380	14.26514	https://www.genecards.org/cgi-bin/carddisp.pl?gene=CPA1
IL1RAPL2	Interleukin 1 Receptor Accessory Protein Like 2	Protein Coding	38	GC0XP104566	14.26404	https://www.genecards.org/cgi-bin/carddisp.pl?gene=IL1RAPL2
KLK3	Kallikrein Related Peptidase 3	Protein Coding	47	GC19P050854	14.25673	https://www.genecards.org/cgi-bin/carddisp.pl?gene=KLK3
FOXG1	Forkhead Box G1	Protein Coding	44	GC14P028766	14.25183	https://www.genecards.org/cgi-bin/carddisp.pl?gene=FOXG1
KCNMA1	Potassium Calcium-Activated Channel Subfamily M Alpha 1	Protein Coding	50	GC10M076869	14.24044	https://www.genecards.org/cgi-bin/carddisp.pl?gene=KCNMA1
IDO1	Indoleamine 2,3-Dioxygenase 1	Protein Coding	45	GC08P039891	14.23615	https://www.genecards.org/cgi-bin/carddisp.pl?gene=IDO1
WNT5A	Wnt Family Member 5A	Protein Coding	50	GC03M055465	14.23408	https://www.genecards.org/cgi-bin/carddisp.pl?gene=WNT5A
CCL4	C-C Motif Chemokine Ligand 4	Protein Coding	41	GC17P036103	14.23141	https://www.genecards.org/cgi-bin/carddisp.pl?gene=CCL4
DPAGT1	Dolichyl-Phosphate N-Acetylglucosaminephosphotransferase 1	Protein Coding	45	GC11M119096	14.20544	https://www.genecards.org/cgi-bin/carddisp.pl?gene=DPAGT1
SEC23B	SEC23 Homolog B, COPII Coat Complex Component	Protein Coding	44	GC20P018507	14.20014	https://www.genecards.org/cgi-bin/carddisp.pl?gene=SEC23B
ALPI	Alkaline Phosphatase, Intestinal	Protein Coding	44	GC02P232456	14.19633	https://www.genecards.org/cgi-bin/carddisp.pl?gene=ALPI
BBS5	Bardet-Biedl Syndrome 5	Protein Coding	39	GC02P169480	14.18223	https://www.genecards.org/cgi-bin/carddisp.pl?gene=BBS5
MIR335	MicroRNA 335	RNA Gene	18	GC07P130496	14.1812	https://www.genecards.org/cgi-bin/carddisp.pl?gene=MIR335
MIR125B1	MicroRNA 125b-1	RNA Gene	21	GC11M122100	14.17307	https://www.genecards.org/cgi-bin/carddisp.pl?gene=MIR125B1
TK2	Thymidine Kinase 2	Protein Coding	42	GC16M066508	14.16266	https://www.genecards.org/cgi-bin/carddisp.pl?gene=TK2
SLC22A18	Solute Carrier Family 22 Member 18	Protein Coding	43	GC11P002899	14.15041	https://www.genecards.org/cgi-bin/carddisp.pl?gene=SLC22A18
HMGA1	High Mobility Group AT-Hook 1	Protein Coding	47	GC06P055309	14.14295	https://www.genecards.org/cgi-bin/carddisp.pl?gene=HMGA1

SULT1A1	Sulfotransferase Family 1A Member 1	Protein Coding	43	GC16M028606	14.13631	https://www.genecards.org/cgi-bin/carddisp.pl?gene=SULT1A1
CYP11A1	Cytochrome P450 Family 11 Subfamily A Member 1	Protein Coding	50	GC15M074337	14.13366	https://www.genecards.org/cgi-bin/carddisp.pl?gene=CYP11A1
CACNA1D	Calcium Voltage-Gated Channel Subunit Alpha1 D	Protein Coding	48	GC03P053328	14.1296	https://www.genecards.org/cgi-bin/carddisp.pl?gene=CACNA1D
NEUROD1	Neuronal Differentiation 1	Protein Coding	44	GC02M181673	14.12438	https://www.genecards.org/cgi-bin/carddisp.pl?gene=NEUROD1
TPM2	Tropomyosin 2	Protein Coding	46	GC09M035672	14.12218	https://www.genecards.org/cgi-bin/carddisp.pl?gene=TPM2
CLOCK	Clock Circadian Regulator	Protein Coding	44	GC04M055427	14.12197	https://www.genecards.org/cgi-bin/carddisp.pl?gene=CLOCK
FGF8	Fibroblast Growth Factor 8	Protein Coding	48	GC10M101770	14.12045	https://www.genecards.org/cgi-bin/carddisp.pl?gene=FGF8
PPP3CA	Protein Phosphatase 3 Catalytic Subunit Alpha	Protein Coding	54	GC04M101024	14.11228	https://www.genecards.org/cgi-bin/carddisp.pl?gene=PPP3CA
BRF1	BRF1 RNA Polymerase III Transcription Initiation Factor Subunit	Protein Coding	41	GC14M105345	14.10909	https://www.genecards.org/cgi-bin/carddisp.pl?gene=BRF1
MMAB	Metabolism Of Cobalamin Associated B	Protein Coding	46	GC12M109553	14.10659	https://www.genecards.org/cgi-bin/carddisp.pl?gene=MMAB
PHEX	Phosphate Regulating Endopeptidase Homolog X-Linked	Protein Coding	41	GC0XP022032	14.10429	https://www.genecards.org/cgi-bin/carddisp.pl?gene=PHEX
MT-TV	Mitochondrially Encoded TRNA-Val (GUN)	RNA Gene	14	GCMTP001605	14.09835	https://www.genecards.org/cgi-bin/carddisp.pl?gene=MT-TV
CD59	CD59 Molecule (CD59 Blood Group)	Protein Coding	47	GC11M033704	14.09563	https://www.genecards.org/cgi-bin/carddisp.pl?gene=CD59
HERC2	HECT And RLD Domain Containing E3 Ubiquitin Protein Ligase 2	Protein Coding	45	GC15M028111	14.09394	https://www.genecards.org/cgi-bin/carddisp.pl?gene=HERC2

PDP1	Pyruvate Dehydrogenase Phosphatase Catalytic Subunit 1	Protein Coding	47	GC08P093857	14.09057	https://www.genecards.org/cgi-bin/carddisp.pl?gene=PDP1
ACP1	Acid Phosphatase 1	Protein Coding	44	GC02P000254	14.08999	https://www.genecards.org/cgi-bin/carddisp.pl?gene=ACP1
IAH1	Isoamyl Acetate Hydrolyzing Esterase 1 (Putative)	Protein Coding	34	GC02P009473	14.08892	https://www.genecards.org/cgi-bin/carddisp.pl?gene=IAH1
COL5A1	Collagen Type V Alpha 1 Chain	Protein Coding	46	GC09P134641	14.08258	https://www.genecards.org/cgi-bin/carddisp.pl?gene=COL5A1
GLP1R	Glucagon Like Peptide 1 Receptor	Protein Coding	47	GC06P039048	14.07604	https://www.genecards.org/cgi-bin/carddisp.pl?gene=GLP1R
VLDLR	Very Low Density Lipoprotein Receptor	Protein Coding	50	GC09P002611	14.07447	https://www.genecards.org/cgi-bin/carddisp.pl?gene=VLDLR
MDH2	Malate Dehydrogenase 2	Protein Coding	49	GC07P076048	14.07031	https://www.genecards.org/cgi-bin/carddisp.pl?gene=MDH2
RPS6KA3	Ribosomal Protein S6 Kinase A3	Protein Coding	53	GC0XM020149	14.06671	https://www.genecards.org/cgi-bin/carddisp.pl?gene=RPS6KA3
ACTN2	Actinin Alpha 2	Protein Coding	48	GC01P236686	14.06519	https://www.genecards.org/cgi-bin/carddisp.pl?gene=ACTN2
SULT1A3	Sulfotransferase Family 1A Member 3	Protein Coding	38	GC16P030199	14.0619	https://www.genecards.org/cgi-bin/carddisp.pl?gene=SULT1A3
FBLN5	Fibulin 5	Protein Coding	45	GC14M091869	14.05901	https://www.genecards.org/cgi-bin/carddisp.pl?gene=FBLN5
TFF3	Trefoil Factor 3	Protein Coding	41	GC21M042311	14.04886	https://www.genecards.org/cgi-bin/carddisp.pl?gene=TFF3
TUBB	Tubulin Beta Class I	Protein Coding	50	GC06P055181	14.04616	https://www.genecards.org/cgi-bin/carddisp.pl?gene=TUBB
TTC7A	Tetratricopeptide Repeat Domain 7A	Protein Coding	38	GC02P046906	14.0451	https://www.genecards.org/cgi-bin/carddisp.pl?gene=TTC7A
NRP1	Neuropilin 1	Protein Coding	47	GC10M033177	14.03739	https://www.genecards.org/cgi-bin/carddisp.pl?gene=NRP1
EPAS1	Endothelial PAS Domain Protein 1	Protein Coding	50	GC02P046293	14.03069	https://www.genecards.org/cgi-bin/carddisp.pl?gene=EPAS1
ATF6	Activating Transcription Factor 6	Protein Coding	48	GC01P161766	14.02761	https://www.genecards.org/cgi-bin/carddisp.pl?gene=ATF6
PLOD1	Procollagen-Lysine,2-Oxoglutarate 5-Dioxygenase 1	Protein Coding	43	GC01P011934	14.01457	https://www.genecards.org/cgi-bin/carddisp.pl?gene=PLOD1
GMPPB	GDP-Mannose Pyrophosphorylase B	Protein Coding	44	GC03M049716	14.01319	https://www.genecards.org/cgi-bin/carddisp.pl?gene=GMPPB

MIR324	MicroRNA 324	RNA Gene	18	GC17M007223	14.00825	https://www.genecards.org/cgi-bin/carddisp.pl?gene=MIR324
TECR	Trans-2,3-Enoyl-CoA Reductase	Protein Coding	44	GC19P014504	14.00636	https://www.genecards.org/cgi-bin/carddisp.pl?gene=TECR
HHEX	Hematopoietically Expressed Homeobox	Protein Coding	43	GC10P092689	14.00541	https://www.genecards.org/cgi-bin/carddisp.pl?gene=HHEX
PTPN1	Protein Tyrosine Phosphatase Non-Receptor Type 1	Protein Coding	51	GC20P050510	14.00476	https://www.genecards.org/cgi-bin/carddisp.pl?gene=PTPN1
CLCN2	Chloride Voltage-Gated Channel 2	Protein Coding	47	GC03M184346	14.00351	https://www.genecards.org/cgi-bin/carddisp.pl?gene=CLCN2
IMPDH1	Inosine Monophosphate Dehydrogenase 1	Protein Coding	50	GC07M128392	13.99338	https://www.genecards.org/cgi-bin/carddisp.pl?gene=IMPDH1
APEX1	Apurinic/Apyrimidinic Endodeoxyribonuclease 1	Protein Coding	45	GC14P020455	13.98484	https://www.genecards.org/cgi-bin/carddisp.pl?gene=APEX1
TXNRD2	Thioredoxin Reductase 2	Protein Coding	48	GC22M019863	13.98362	https://www.genecards.org/cgi-bin/carddisp.pl?gene=TXNRD2
DBI	Diazepam Binding Inhibitor, Acyl-CoA Binding Protein	Protein Coding	47	GC02P119366	13.96494	https://www.genecards.org/cgi-bin/carddisp.pl?gene=DBI
MIR331	MicroRNA 331	RNA Gene	18	GC12P095308	13.96247	https://www.genecards.org/cgi-bin/carddisp.pl?gene=MIR331
BAG3	BAG Cochaperone 3	Protein Coding	45	GC10P119651	13.95862	https://www.genecards.org/cgi-bin/carddisp.pl?gene=BAG3
ATN1	Atrophin 1	Protein Coding	44	GC12P011868	13.94733	https://www.genecards.org/cgi-bin/carddisp.pl?gene=ATN1
COG7	Component Of Oligomeric Golgi Complex 7	Protein Coding	39	GC16M023388	13.94407	https://www.genecards.org/cgi-bin/carddisp.pl?gene=COG7
LPIN2	Lipin 2	Protein Coding	43	GC18M002916	13.94319	https://www.genecards.org/cgi-bin/carddisp.pl?gene=LPIN2
EYS	Eyes Shut Homolog	Protein Coding	36	GC06M063719	13.9251	https://www.genecards.org/cgi-bin/carddisp.pl?gene=EYS
MYB	MYB Proto-Oncogene, Transcription Factor	Protein Coding	51	GC06P135180	13.92469	https://www.genecards.org/cgi-bin/carddisp.pl?gene=MYB
ABCD2	ATP Binding Cassette Subfamily D Member 2	Protein Coding	41	GC12M039530	13.92256	https://www.genecards.org/cgi-bin/carddisp.pl?gene=ABCD2
BMP7	Bone Morphogenetic Protein 7	Protein Coding	46	GC20M057168	13.91285	https://www.genecards.org/cgi-bin/carddisp.pl?gene=BMP7

CCR2	C-C Motif Chemokine Receptor 2	Protein Coding	45	GC03P046356	13.89845	https://www.genecards.org/cgi-bin/carddisp.pl?gene=CCR2
CELA2A	Chymotrypsin Like Elastase 2A	Protein Coding	39	GC01P015456	13.89703	https://www.genecards.org/cgi-bin/carddisp.pl?gene=CELA2A
OTX2	Orthodenticle Homeobox 2	Protein Coding	47	GC14M056799	13.89702	https://www.genecards.org/cgi-bin/carddisp.pl?gene=OTX2
FOXM1	Forkhead Box M1	Protein Coding	45	GC12M002857	13.8897	https://www.genecards.org/cgi-bin/carddisp.pl?gene=FOXM1
PIEZO1	Piezo Type Mechanosensitive Ion Channel Component 1	Protein Coding	38	GC16M088715	13.87765	https://www.genecards.org/cgi-bin/carddisp.pl?gene=PIEZO1
EPOR	Erythropoietin Receptor	Protein Coding	48	GC19M011377	13.87609	https://www.genecards.org/cgi-bin/carddisp.pl?gene=EPOR
ABCB6	ATP Binding Cassette Subfamily B Member 6 (Langereis Blood Group)	Protein Coding	47	GC02M219209	13.87128	https://www.genecards.org/cgi-bin/carddisp.pl?gene=ABCB6
MIR124-1	MicroRNA 124-1	RNA Gene	21	GC08M009903	13.86133	https://www.genecards.org/cgi-bin/carddisp.pl?gene=MIR124-1
GRM1	Glutamate Metabotropic Receptor 1	Protein Coding	51	GC06P145973	13.85019	https://www.genecards.org/cgi-bin/carddisp.pl?gene=GRM1
GPX3	Glutathione Peroxidase 3	Protein Coding	44	GC05P150997	13.84768	https://www.genecards.org/cgi-bin/carddisp.pl?gene=GPX3
PKP2	Plakophilin 2	Protein Coding	46	GC12M032790	13.84009	https://www.genecards.org/cgi-bin/carddisp.pl?gene=PKP2
MMP8	Matrix Metallopeptidase 8	Protein Coding	48	GC11M102617	13.83777	https://www.genecards.org/cgi-bin/carddisp.pl?gene=MMP8
SLC13A5	Solute Carrier Family 13 Member 5	Protein Coding	43	GC17M006684	13.83693	https://www.genecards.org/cgi-bin/carddisp.pl?gene=SLC13A5
RASSF1	Ras Association Domain Family Member 1	Protein Coding	45	GC03M050329	13.83044	https://www.genecards.org/cgi-bin/carddisp.pl?gene=RASSF1
TIMP3	TIMP Metallopeptidase Inhibitor 3	Protein Coding	46	GC22P032800	13.81891	https://www.genecards.org/cgi-bin/carddisp.pl?gene=TIMP3
TYMS	Thymidylate Synthetase	Protein Coding	48	GC18P000657	13.81405	https://www.genecards.org/cgi-bin/carddisp.pl?gene=TYMS
CXCR1	C-X-C Motif Chemokine Receptor 1	Protein Coding	44	GC02M218162	13.80201	https://www.genecards.org/cgi-bin/carddisp.pl?gene=CXCR1
TLR1	Toll Like Receptor 1	Protein Coding	48	GC04M038793	13.80146	https://www.genecards.org/cgi-bin/carddisp.pl?gene=TLR1
PROC	Protein C, Inactivator Of Coagulation Factors Va And VIIIa	Protein Coding	50	GC02P127418	13.79554	https://www.genecards.org/cgi-bin/carddisp.pl?gene=PROC

COX15	Cytochrome C Oxidase Assembly Homolog COX15	Protein Coding	43	GC10M099696	13.79495	https://www.genecards.org/cgi-bin/carddisp.pl?gene=COX15
BAD	BCL2 Associated Agonist Of Cell Death	Protein Coding	47	GC11M069329	13.78966	https://www.genecards.org/cgi-bin/carddisp.pl?gene=BAD
PRKCB	Protein Kinase C Beta	Protein Coding	48	GC16P023967	13.78564	https://www.genecards.org/cgi-bin/carddisp.pl?gene=PRKCB
PTGDS	Prostaglandin D2 Synthase	Protein Coding	45	GC09P137005	13.78054	https://www.genecards.org/cgi-bin/carddisp.pl?gene=PTGDS
VDAC1	Voltage Dependent Anion Channel 1	Protein Coding	47	GC05M133975	13.77833	https://www.genecards.org/cgi-bin/carddisp.pl?gene=VDAC1
OSM	Oncostatin M	Protein Coding	43	GC22M030262	13.76358	https://www.genecards.org/cgi-bin/carddisp.pl?gene=OSM
SOAT1	Sterol O-Acyltransferase 1	Protein Coding	45	GC01P179262	13.76268	https://www.genecards.org/cgi-bin/carddisp.pl?gene=SOAT1
IL17RA	Interleukin 17 Receptor A	Protein Coding	45	GC22P017116	13.74624	https://www.genecards.org/cgi-bin/carddisp.pl?gene=IL17RA
IGFBP2	Insulin Like Growth Factor Binding Protein 2	Protein Coding	44	GC02P216632	13.74202	https://www.genecards.org/cgi-bin/carddisp.pl?gene=IGFBP2
MMP12	Matrix Metallopeptidase 12	Protein Coding	45	GC11M102862	13.73298	https://www.genecards.org/cgi-bin/carddisp.pl?gene=MMP12
TAF1	TATA-Box Binding Protein Associated Factor 1	Protein Coding	46	GC0XP071366	13.72882	https://www.genecards.org/cgi-bin/carddisp.pl?gene=TAF1
KIAA0753	KIAA0753	Protein Coding	34	GC17M006578	13.72798	https://www.genecards.org/cgi-bin/carddisp.pl?gene=KIAA0753
PRDM16	PR/SET Domain 16	Protein Coding	45	GC01P003068	13.72771	https://www.genecards.org/cgi-bin/carddisp.pl?gene=PRDM16
RASGRP1	RAS Guanyl Releasing Protein 1	Protein Coding	48	GC15M038488	13.72265	https://www.genecards.org/cgi-bin/carddisp.pl?gene=RASGRP1
C1S	Complement C1s	Protein Coding	47	GC12P011876	13.71999	https://www.genecards.org/cgi-bin/carddisp.pl?gene=C1S
BPTF	Bromodomain PHD Finger Transcription Factor	Protein Coding	40	GC17P067825	13.70828	https://www.genecards.org/cgi-bin/carddisp.pl?gene=BPTF
PTHLH	Parathyroid Hormone Like Hormone	Protein Coding	46	GC12M027959	13.70041	https://www.genecards.org/cgi-bin/carddisp.pl?gene=PTHLH
CASK	Calcium/Calmodulin Dependent Serine Protein Kinase	Protein Coding	49	GC0XM041514	13.69434	https://www.genecards.org/cgi-bin/carddisp.pl?gene=CASK

CCKAR	Cholecystokinin A Receptor	Protein Coding	47	GC04M026483	13.68479	https://www.genecards.org/cgi-bin/carddisp.pl?gene=CCKAR
SUOX	Sulfite Oxidase	Protein Coding	46	GC12P055997	13.67192	https://www.genecards.org/cgi-bin/carddisp.pl?gene=SUOX
AKR1B10	Aldo-Keto Reductase Family 1 Member B10	Protein Coding	44	GC07P134527	13.6697	https://www.genecards.org/cgi-bin/carddisp.pl?gene=AKR1B10
HAVCR1	Hepatitis A Virus Cellular Receptor 1	Protein Coding	42	GC05M157028	13.65866	https://www.genecards.org/cgi-bin/carddisp.pl?gene=HAVCR1
POLD1	DNA Polymerase Delta 1, Catalytic Subunit	Protein Coding	47	GC19P050385	13.6574	https://www.genecards.org/cgi-bin/carddisp.pl?gene=POLD1
TPI1	Triosephosphate Isomerase 1	Protein Coding	48	GC12P011857	13.65639	https://www.genecards.org/cgi-bin/carddisp.pl?gene=TPI1
DDX58	DExD/H-Box Helicase 58	Protein Coding	47	GC09M032455	13.6557	https://www.genecards.org/cgi-bin/carddisp.pl?gene=DDX58
CASP2	Caspase 2	Protein Coding	50	GC07P145813	13.6534	https://www.genecards.org/cgi-bin/carddisp.pl?gene=CASP2
HK2	Hexokinase 2	Protein Coding	48	GC02P074833	13.6505	https://www.genecards.org/cgi-bin/carddisp.pl?gene=HK2
TMEM138	Transmembrane Protein 138	Protein Coding	37	GC11P061362	13.64693	https://www.genecards.org/cgi-bin/carddisp.pl?gene=TMEM138
PLD3	Phospholipase D Family Member 3	Protein Coding	43	GC19P040348	13.62637	https://www.genecards.org/cgi-bin/carddisp.pl?gene=PLD3
PDHA1	Pyruvate Dehydrogenase E1 Subunit Alpha 1	Protein Coding	50	GC0XP019343	13.6236	https://www.genecards.org/cgi-bin/carddisp.pl?gene=PDHA1
PDE4A	Phosphodiesterase 4A	Protein Coding	45	GC19P010416	13.61808	https://www.genecards.org/cgi-bin/carddisp.pl?gene=PDE4A
CD86	CD86 Molecule	Protein Coding	44	GC03P122055	13.61566	https://www.genecards.org/cgi-bin/carddisp.pl?gene=CD86
IL4R	Interleukin 4 Receptor	Protein Coding	48	GC16P027325	13.6084	https://www.genecards.org/cgi-bin/carddisp.pl?gene=IL4R
FAM13A	Family With Sequence Similarity 13 Member A	Protein Coding	38	GC04M088725	13.58992	https://www.genecards.org/cgi-bin/carddisp.pl?gene=FAM13A
GNRH1	Gonadotropin Releasing Hormone 1	Protein Coding	43	GC08M025419	13.58939	https://www.genecards.org/cgi-bin/carddisp.pl?gene=GNRH1
WNT3A	Wnt Family Member 3A	Protein Coding	48	GC01P228466	13.57946	https://www.genecards.org/cgi-bin/carddisp.pl?gene=WNT3A
ALDH5A1	Aldehyde Dehydrogenase 5 Family Member A1	Protein Coding	47	GC06P024494	13.5575	https://www.genecards.org/cgi-bin/carddisp.pl?gene=ALDH5A1
POSTN	Periostin	Protein Coding	44	GC13M037562	13.55501	https://www.genecards.org/cgi-bin/carddisp.pl?gene=POSTN
TTC8	Tetratricopeptide Repeat Domain 8	Protein Coding	40	GC14P089293	13.54434	https://www.genecards.org/cgi-bin/carddisp.pl?gene=TTC8

ECI2	Enoyl-CoA Delta Isomerase 2	Protein Coding	41	GC06M004115	13.54339	https://www.genecards.org/cgi-bin/carddisp.pl?gene=ECI2
COL11A2	Collagen Type XI Alpha 2 Chain	Protein Coding	45	GC06M033162	13.53173	https://www.genecards.org/cgi-bin/carddisp.pl?gene=COL11A2
SLC26A4	Solute Carrier Family 26 Member 4	Protein Coding	44	GC07P107660	13.51274	https://www.genecards.org/cgi-bin/carddisp.pl?gene=SLC26A4
PPBP	Pro-Platelet Basic Protein	Protein Coding	44	GC04M073986	13.49522	https://www.genecards.org/cgi-bin/carddisp.pl?gene=PPBP
ALDH18A1	Aldehyde Dehydrogenase 18 Family Member A1	Protein Coding	46	GC10M095605	13.49513	https://www.genecards.org/cgi-bin/carddisp.pl?gene=ALDH18A1
KCNJ1	Potassium Inwardly Rectifying Channel Subfamily J Member 1	Protein Coding	48	GC11M128741	13.49502	https://www.genecards.org/cgi-bin/carddisp.pl?gene=KCNJ1
TSFM	Ts Translation Elongation Factor, Mitochondrial	Protein Coding	44	GC12P057778	13.49161	https://www.genecards.org/cgi-bin/carddisp.pl?gene=TSFM
ANGPTL8	Angiopoietin Like 8	Protein Coding	29	GC19P011238	13.48359	https://www.genecards.org/cgi-bin/carddisp.pl?gene=ANGPTL8
OGDH	Oxoglutarate Dehydrogenase	Protein Coding	46	GC07P044606	13.48221	https://www.genecards.org/cgi-bin/carddisp.pl?gene=OGDH
FOXA2	Forkhead Box A2	Protein Coding	45	GC20M022581	13.48104	https://www.genecards.org/cgi-bin/carddisp.pl?gene=FOXA2
FOXP1	Forkhead Box P1	Protein Coding	46	GC03M070926	13.4762	https://www.genecards.org/cgi-bin/carddisp.pl?gene=FOXP1
MSTN	Myostatin	Protein Coding	45	GC02M190055	13.47489	https://www.genecards.org/cgi-bin/carddisp.pl?gene=MSTN
CTSK	Cathepsin K	Protein Coding	50	GC01M150837	13.46922	https://www.genecards.org/cgi-bin/carddisp.pl?gene=CTSK
CPLANE1	Ciliogenesis And Planar Polarity Effector 1	Protein Coding	27	GC05M037084	13.45301	https://www.genecards.org/cgi-bin/carddisp.pl?gene=CPLANE1
IL18R1	Interleukin 18 Receptor 1	Protein Coding	43	GC02P102311	13.44806	https://www.genecards.org/cgi-bin/carddisp.pl?gene=IL18R1
NAGS	N-Acetylglutamate Synthase	Protein Coding	43	GC17P044004	13.43669	https://www.genecards.org/cgi-bin/carddisp.pl?gene=NAGS
SLC52A3	Solute Carrier Family 52 Member 3	Protein Coding	39	GC20M000741	13.42792	https://www.genecards.org/cgi-bin/carddisp.pl?gene=SLC52A3
RHD	Rh Blood Group D Antigen	Protein Coding	41	GC01P025272	13.41545	https://www.genecards.org/cgi-bin/carddisp.pl?gene=RHD
E2F1	E2F Transcription Factor 1	Protein Coding	44	GC20M033675	13.39812	https://www.genecards.org/cgi-bin/carddisp.pl?gene=E2F1
NF2	Neurofibromin 2	Protein Coding	49	GC22P029603	13.3971	https://www.genecards.org/cgi-bin/carddisp.pl?gene=NF2

MRE11	MRE11 Homolog, Double Strand Break Repair Nuclease	Protein Coding	42	GC11M094875	13.39581	https://www.genecards.org/cgi-bin/carddisp.pl?gene=MRE11
CEP104	Centrosomal Protein 104	Protein Coding	36	GC01M003812	13.39383	https://www.genecards.org/cgi-bin/carddisp.pl?gene=CEP104
EZR	Ezrin	Protein Coding	46	GC06M158765	13.38914	https://www.genecards.org/cgi-bin/carddisp.pl?gene=EZR
TRPC6	Transient Receptor Potential Cation Channel Subfamily C Member 6	Protein Coding	49	GC11M101451	13.38856	https://www.genecards.org/cgi-bin/carddisp.pl?gene=TRPC6
CLCNKA	Chloride Voltage-Gated Channel Ka	Protein Coding	41	GC01P016018	13.38671	https://www.genecards.org/cgi-bin/carddisp.pl?gene=CLCNKA
CANX	Calnexin	Protein Coding	45	GC05P179678	13.38223	https://www.genecards.org/cgi-bin/carddisp.pl?gene=CANX
MIRLET7A1	MicroRNA Let-7a-1	RNA Gene	21	GC09P094175	13.37969	https://www.genecards.org/cgi-bin/carddisp.pl?gene=MIRLET7A1
COL6A1	Collagen Type VI Alpha 1 Chain	Protein Coding	44	GC21P045981	13.37753	https://www.genecards.org/cgi-bin/carddisp.pl?gene=COL6A1
RNU4ATAC	RNA, U4atac Small Nuclear (U12-Dependent Splicing)	RNA Gene	20	GC02P121539	13.37517	https://www.genecards.org/cgi-bin/carddisp.pl?gene=RNU4ATAC
IL33	Interleukin 33	Protein Coding	40	GC09P006206	13.36874	https://www.genecards.org/cgi-bin/carddisp.pl?gene=IL33
ADSL	Adenylosuccinate Lyase	Protein Coding	48	GC22P040346	13.35884	https://www.genecards.org/cgi-bin/carddisp.pl?gene=ADSL
CHPT1	Choline Phosphotransferase 1	Protein Coding	40	GC12P101696	13.34262	https://www.genecards.org/cgi-bin/carddisp.pl?gene=CHPT1
MEF2C	Myocyte Enhancer Factor 2C	Protein Coding	50	GC05M088718	13.34108	https://www.genecards.org/cgi-bin/carddisp.pl?gene=MEF2C
HAVCR2	Hepatitis A Virus Cellular Receptor 2	Protein Coding	44	GC05M157063	13.34015	https://www.genecards.org/cgi-bin/carddisp.pl?gene=HAVCR2
HGD	Homogentisate 1,2-Dioxygenase	Protein Coding	45	GC03M120628	13.3281	https://www.genecards.org/cgi-bin/carddisp.pl?gene=HGD
NDUFAB1	NADH:Ubiquinone Oxidoreductase Subunit AB1	Protein Coding	41	GC16M023582	13.32784	https://www.genecards.org/cgi-bin/carddisp.pl?gene=NDUFAB1
GLI1	GLI Family Zinc Finger 1	Protein Coding	48	GC12P057460	13.3216	https://www.genecards.org/cgi-bin/carddisp.pl?gene=GLI1
SLC7A7	Solute Carrier Family 7 Member 7	Protein Coding	47	GC14M022773	13.31963	https://www.genecards.org/cgi-bin/carddisp.pl?gene=SLC7A7

UGCG	UDP-Glucose Ceramide Glucosyltransferase	Protein Coding	43	GC09P111896	13.31046	https://www.genecards.org/cgi-bin/carddisp.pl?gene=UGCG
ANPEP	Alanyl Aminopeptidase, Membrane	Protein Coding	50	GC15M089784	13.30516	https://www.genecards.org/cgi-bin/carddisp.pl?gene=ANPEP
MBOAT7	Membrane Bound O-Acyltransferase Domain Containing 7	Protein Coding	41	GC19M054173	13.30096	https://www.genecards.org/cgi-bin/carddisp.pl?gene=MBOAT7
HPX	Hemopexin	Protein Coding	40	GC11M006435	13.30056	https://www.genecards.org/cgi-bin/carddisp.pl?gene=HPX
CEP41	Centrosomal Protein 41	Protein Coding	39	GC07M130393	13.29879	https://www.genecards.org/cgi-bin/carddisp.pl?gene=CEP41
DMPK	DM1 Protein Kinase	Protein Coding	49	GC19M045769	13.28736	https://www.genecards.org/cgi-bin/carddisp.pl?gene=DMPK
NUP107	Nucleoporin 107	Protein Coding	43	GC12P068686	13.28543	https://www.genecards.org/cgi-bin/carddisp.pl?gene=NUP107
PKM	Pyruvate Kinase M1/2	Protein Coding	46	GC15M072199	13.28074	https://www.genecards.org/cgi-bin/carddisp.pl?gene=PKM
ALOX5AP	Arachidonate 5-Lipoxygenase Activating Protein	Protein Coding	45	GC13P030713	13.27863	https://www.genecards.org/cgi-bin/carddisp.pl?gene=ALOX5AP
PICALM	Phosphatidylinositol Binding Clathrin Assembly Protein	Protein Coding	44	GC11M085957	13.27776	https://www.genecards.org/cgi-bin/carddisp.pl?gene=PICALM
DHRS2	Dehydrogenase/Reductase 2	Protein Coding	40	GC14P026312	13.27757	https://www.genecards.org/cgi-bin/carddisp.pl?gene=DHRS2
GNA11	G Protein Subunit Alpha 11	Protein Coding	48	GC19P003094	13.27677	https://www.genecards.org/cgi-bin/carddisp.pl?gene=GNA11
POU5F1	POU Class 5 Homeobox 1	Protein Coding	47	GC06M046826	13.27455	https://www.genecards.org/cgi-bin/carddisp.pl?gene=POU5F1
DHX30	DExH-Box Helicase 30	Protein Coding	41	GC03P047802	13.26659	https://www.genecards.org/cgi-bin/carddisp.pl?gene=DHX30
NDUFA11	NADH:Ubiquinone Oxidoreductase Subunit A11	Protein Coding	38	GC19M005891	13.26033	https://www.genecards.org/cgi-bin/carddisp.pl?gene=NDUFA11
RARS1	Arginyl-TRNA Synthetase 1	Protein Coding	37	GC05P168487	13.24706	https://www.genecards.org/cgi-bin/carddisp.pl?gene=RARS1
LYST	Lysosomal Trafficking Regulator	Protein Coding	38	GC01M235661	13.2379	https://www.genecards.org/cgi-bin/carddisp.pl?gene=LYST
AAAS	Aladin WD Repeat Nucleoporin	Protein Coding	42	GC12M053307	13.23345	https://www.genecards.org/cgi-bin/carddisp.pl?gene=AAAS
VRK1	VRK Serine/Threonine Kinase 1	Protein Coding	49	GC14P096797	13.2327	https://www.genecards.org/cgi-bin/carddisp.pl?gene=VRK1
CYC1	Cytochrome C1	Protein Coding	46	GC08P144095	13.22562	https://www.genecards.org/cgi-bin/carddisp.pl?gene=CYC1

NDE1	NudE Neurodevelopment Protein 1	Protein Coding	44	GC16P015661	13.21717	https://www.genecards.org/cgi-bin/carddisp.pl?gene=NDE1
MIR152	MicroRNA 152	RNA Gene	20	GC17M048037	13.21089	https://www.genecards.org/cgi-bin/carddisp.pl?gene=MIR152
GH-LCR	Growth Hormone Locus Control Region	Biological Region	2	GC17P063917	13.20954	https://www.genecards.org/cgi-bin/carddisp.pl?gene=GH-LCR
DHDDS	Dehydrodolichyl Diphosphate Synthase Subunit	Protein Coding	43	GC01P026432	13.20563	https://www.genecards.org/cgi-bin/carddisp.pl?gene=DHDDS
CCKBR	Cholecystokinin B Receptor	Protein Coding	45	GC11P006259	13.20471	https://www.genecards.org/cgi-bin/carddisp.pl?gene=CCKBR
RAB27A	RAB27A, Member RAS Oncogene Family	Protein Coding	49	GC15M055202	13.1928	https://www.genecards.org/cgi-bin/carddisp.pl?gene=RAB27A
OLR1	Oxidized Low Density Lipoprotein Receptor 1	Protein Coding	44	GC12M015685	13.18879	https://www.genecards.org/cgi-bin/carddisp.pl?gene=OLR1
XRCC4	X-Ray Repair Cross Complementing 4	Protein Coding	44	GC05P083077	13.18428	https://www.genecards.org/cgi-bin/carddisp.pl?gene=XRCC4
MAG	Myelin Associated Glycoprotein	Protein Coding	46	GC19P035292	13.173	https://www.genecards.org/cgi-bin/carddisp.pl?gene=MAG
YY1AP1	YY1 Associated Protein 1	Protein Coding	39	GC01M155659	13.17136	https://www.genecards.org/cgi-bin/carddisp.pl?gene=YY1AP1
CNTF	Ciliary Neurotrophic Factor	Protein Coding	42	GC11P058622	13.16641	https://www.genecards.org/cgi-bin/carddisp.pl?gene=CNTF
ASAH2	N-Acylsphingosine Amidohydrolase 2	Protein Coding	42	GC10M050182	13.16553	https://www.genecards.org/cgi-bin/carddisp.pl?gene=ASAH2
TFEB	Transcription Factor EB	Protein Coding	43	GC06M047093	13.15278	https://www.genecards.org/cgi-bin/carddisp.pl?gene=TFEB
CSNK2A1	Casein Kinase 2 Alpha 1	Protein Coding	52	GC20M000472	13.13254	https://www.genecards.org/cgi-bin/carddisp.pl?gene=CSNK2A1
ANXA1	Annexin A1	Protein Coding	50	GC09P073151	13.1302	https://www.genecards.org/cgi-bin/carddisp.pl?gene=ANXA1
MIR139	MicroRNA 139	RNA Gene	19	GC11M072615	13.12156	https://www.genecards.org/cgi-bin/carddisp.pl?gene=MIR139
TCAP	Titin-Cap	Protein Coding	43	GC17P039664	13.12141	https://www.genecards.org/cgi-bin/carddisp.pl?gene=TCAP
FLVCR1	FLVCR Heme Transporter 1	Protein Coding	41	GC01P212858	13.12076	https://www.genecards.org/cgi-bin/carddisp.pl?gene=FLVCR1
FURIN	Furin, Paired Basic Amino Acid Cleaving Enzyme	Protein Coding	47	GC15P090868	13.11329	https://www.genecards.org/cgi-bin/carddisp.pl?gene=FURIN

NFKB2	Nuclear Factor Kappa B Subunit 2	Protein Coding	52	GC10P102394	13.10839	https://www.genecards.org/cgi-bin/carddisp.pl?gene=NFKB2
LIF	LIF Interleukin 6 Family Cytokine	Protein Coding	43	GC22M030240	13.10428	https://www.genecards.org/cgi-bin/carddisp.pl?gene=LIF
HDAC2	Histone Deacetylase 2	Protein Coding	52	GC06M113933	13.0973	https://www.genecards.org/cgi-bin/carddisp.pl?gene=HDAC2
ADCY5	Adenylate Cyclase 5	Protein Coding	49	GC03M123282	13.09327	https://www.genecards.org/cgi-bin/carddisp.pl?gene=ADCY5
IMPG1	Interphotoreceptor Matrix Proteoglycan 1	Protein Coding	40	GC06M075921	13.08588	https://www.genecards.org/cgi-bin/carddisp.pl?gene=IMPG1
LZTR1	Leucine Zipper Like Transcription Regulator 1	Protein Coding	43	GC22P020996	13.08452	https://www.genecards.org/cgi-bin/carddisp.pl?gene=LZTR1
GPC4	Glypican 4	Protein Coding	46	GC0XM133300	13.07306	https://www.genecards.org/cgi-bin/carddisp.pl?gene=GPC4
SRD5A2	Steroid 5 Alpha-Reductase 2	Protein Coding	43	GC02M031522	13.07273	https://www.genecards.org/cgi-bin/carddisp.pl?gene=SRD5A2
NDUFA9	NADH:Ubiquinone Oxidoreductase Subunit A9	Protein Coding	44	GC12P004649	13.06477	https://www.genecards.org/cgi-bin/carddisp.pl?gene=NDUFA9
AGTR2	Angiotensin II Receptor Type 2	Protein Coding	44	GC0XP116170	13.06456	https://www.genecards.org/cgi-bin/carddisp.pl?gene=AGTR2
WNT4	Wnt Family Member 4	Protein Coding	48	GC01M022190	13.06435	https://www.genecards.org/cgi-bin/carddisp.pl?gene=WNT4
PAX4	Paired Box 4	Protein Coding	44	GC07M127610	13.06254	https://www.genecards.org/cgi-bin/carddisp.pl?gene=PAX4
ERLIN1	ER Lipid Raft Associated 1	Protein Coding	43	GC10M100150	13.05514	https://www.genecards.org/cgi-bin/carddisp.pl?gene=ERLIN1
HNRNPK	Heterogeneous Nuclear Ribonucleoprotein K	Protein Coding	47	GC09M086093	13.05486	https://www.genecards.org/cgi-bin/carddisp.pl?gene=HNRNPK
VTN	Vitronectin	Protein Coding	44	GC17M031079	13.05256	https://www.genecards.org/cgi-bin/carddisp.pl?gene=VTN
CIDEC	Cell Death Inducing DFFA Like Effector C	Protein Coding	42	GC03M009866	13.04613	https://www.genecards.org/cgi-bin/carddisp.pl?gene=CIDEC
NDUFA2	NADH:Ubiquinone Oxidoreductase Subunit A2	Protein Coding	43	GC05M140653	13.02415	https://www.genecards.org/cgi-bin/carddisp.pl?gene=NDUFA2
CSF3R	Colony Stimulating Factor 3 Receptor	Protein Coding	47	GC01M036466	13.021	https://www.genecards.org/cgi-bin/carddisp.pl?gene=CSF3R
P4HB	Prolyl 4-Hydroxylase Subunit Beta	Protein Coding	50	GC17M081843	13.02099	https://www.genecards.org/cgi-bin/carddisp.pl?gene=P4HB

PMPCA	Peptidase, Mitochondrial Processing Subunit Alpha	Protein Coding	44	GC09P136410	13.01141	https://www.genecards.org/cgi-bin/carddisp.pl?gene=PMPCA
BDKRB2	Bradykinin Receptor B2	Protein Coding	45	GC14P096205	13.01027	https://www.genecards.org/cgi-bin/carddisp.pl?gene=BDKRB2
ATRIP-TREX1	ATRIP-TREX1 Readthrough	RNA Gene	5	GC03P048450	13.00792	https://www.genecards.org/cgi-bin/carddisp.pl?gene=ATRIP-TREX1
FANCM	FA Complementation Group M	Protein Coding	43	GC14P045135	13.00647	https://www.genecards.org/cgi-bin/carddisp.pl?gene=FANCM
NCSTN	Nicastrin	Protein Coding	47	GC01P160343	12.99393	https://www.genecards.org/cgi-bin/carddisp.pl?gene=NCSTN
MAPK9	Mitogen-Activated Protein Kinase 9	Protein Coding	49	GC05M180234	12.9919	https://www.genecards.org/cgi-bin/carddisp.pl?gene=MAPK9
LDLRAP1	Low Density Lipoprotein Receptor Adaptor Protein 1	Protein Coding	44	GC01P025543	12.98189	https://www.genecards.org/cgi-bin/carddisp.pl?gene=LDLRAP1
HNMT	Histamine N-Methyltransferase	Protein Coding	46	GC02P137964	12.98167	https://www.genecards.org/cgi-bin/carddisp.pl?gene=HNMT
ACE2	Angiotensin Converting Enzyme 2	Protein Coding	49	GC0XM015494	12.97091	https://www.genecards.org/cgi-bin/carddisp.pl?gene=ACE2
WNK1	WNK Lysine Deficient Protein Kinase 1	Protein Coding	47	GC12P000733	12.96705	https://www.genecards.org/cgi-bin/carddisp.pl?gene=WNK1
DLK1	Delta Like Non-Canonical Notch Ligand 1	Protein Coding	45	GC14P106102	12.9622	https://www.genecards.org/cgi-bin/carddisp.pl?gene=DLK1
GLI2	GLI Family Zinc Finger 2	Protein Coding	48	GC02P120735	12.95878	https://www.genecards.org/cgi-bin/carddisp.pl?gene=GLI2
OXT	Oxytocin/Neurophysin I Prepropeptide	Protein Coding	41	GC20P003068	12.95798	https://www.genecards.org/cgi-bin/carddisp.pl?gene=OXT
GP1BB	Glycoprotein Ib Platelet Subunit Beta	Protein Coding	43	GC22P020083	12.94585	https://www.genecards.org/cgi-bin/carddisp.pl?gene=GP1BB
AQP4	Aquaporin 4	Protein Coding	47	GC18M026852	12.94447	https://www.genecards.org/cgi-bin/carddisp.pl?gene=AQP4
ORAI1	ORAI Calcium Release-Activated Calcium Modulator 1	Protein Coding	44	GC12P123580	12.92715	https://www.genecards.org/cgi-bin/carddisp.pl?gene=ORAI1
MIR99A	MicroRNA 99a	RNA Gene	21	GC21P016539	12.92301	https://www.genecards.org/cgi-bin/carddisp.pl?gene=MIR99A
GLS2	Glutaminase 2	Protein Coding	43	GC12M056470	12.91247	https://www.genecards.org/cgi-bin/carddisp.pl?gene=GLS2

DPM1	Dolichyl-Phosphate Mannosyltransferase Subunit 1, Catalytic	Protein Coding	45	GC20M050934	12.90042	https://www.genecards.org/cgi-bin/carddisp.pl?gene=DPM1
IL2RG	Interleukin 2 Receptor Subunit Gamma	Protein Coding	48	GC0XM071108	12.89767	https://www.genecards.org/cgi-bin/carddisp.pl?gene=IL2RG
AGA	Aspartylglucosaminidase	Protein Coding	45	GC04M177430	12.89751	https://www.genecards.org/cgi-bin/carddisp.pl?gene=AGA
EBF3	EBF Transcription Factor 3	Protein Coding	41	GC10M129835	12.87708	https://www.genecards.org/cgi-bin/carddisp.pl?gene=EBF3
CTCF	CCCTC-Binding Factor	Protein Coding	47	GC16P067563	12.87645	https://www.genecards.org/cgi-bin/carddisp.pl?gene=CTCF
AREG	Amphiregulin	Protein Coding	43	GC04P074445	12.87519	https://www.genecards.org/cgi-bin/carddisp.pl?gene=AREG
TRAF6	TNF Receptor Associated Factor 6	Protein Coding	47	GC11M036467	12.8637	https://www.genecards.org/cgi-bin/carddisp.pl?gene=TRAF6
MIR197	MicroRNA 197	RNA Gene	19	GC01P109549	12.85798	https://www.genecards.org/cgi-bin/carddisp.pl?gene=MIR197
TPM3	Tropomyosin 3	Protein Coding	47	GC01M154127	12.85281	https://www.genecards.org/cgi-bin/carddisp.pl?gene=TPM3
DHRS4	Dehydrogenase/Reductase 4	Protein Coding	45	GC14P023953	12.84587	https://www.genecards.org/cgi-bin/carddisp.pl?gene=DHRS4
IL23A	Interleukin 23 Subunit Alpha	Protein Coding	40	GC12P056440	12.84121	https://www.genecards.org/cgi-bin/carddisp.pl?gene=IL23A
KLF11	Kruppel Like Factor 11	Protein Coding	40	GC02P010044	12.8358	https://www.genecards.org/cgi-bin/carddisp.pl?gene=KLF11
TPM1	Tropomyosin 1	Protein Coding	50	GC15P078910	12.83545	https://www.genecards.org/cgi-bin/carddisp.pl?gene=TPM1
USP9X	Ubiquitin Specific Peptidase 9 X-Linked	Protein Coding	48	GC0XP041085	12.83245	https://www.genecards.org/cgi-bin/carddisp.pl?gene=USP9X
RARS2	Arginyl-TRNA Synthetase 2, Mitochondrial	Protein Coding	43	GC06M087514	12.82485	https://www.genecards.org/cgi-bin/carddisp.pl?gene=RARS2
SHPK	Sedoheptulokinase	Protein Coding	38	GC17M003608	12.8208	https://www.genecards.org/cgi-bin/carddisp.pl?gene=SHPK
PLCE1	Phospholipase C Epsilon 1	Protein Coding	44	GC10P093993	12.81417	https://www.genecards.org/cgi-bin/carddisp.pl?gene=PLCE1
APAF1	Apoptotic Peptidase Activating Factor 1	Protein Coding	47	GC12P098645	12.81352	https://www.genecards.org/cgi-bin/carddisp.pl?gene=APAF1
EIF4E	Eukaryotic Translation Initiation Factor 4E	Protein Coding	50	GC04M098879	12.81149	https://www.genecards.org/cgi-bin/carddisp.pl?gene=EIF4E
CUBN	Cubilin	Protein Coding	47	GC10M016824	12.80985	https://www.genecards.org/cgi-bin/carddisp.pl?gene=CUBN
CYP4F3	Cytochrome P450 Family 4 Subfamily F Member 3	Protein Coding	44	GC19P015640	12.80623	https://www.genecards.org/cgi-bin/carddisp.pl?gene=CYP4F3

LPIN1	Lipin 1	Protein Coding	48	GC02P011677	12.79837	https://www.genecards.org/cgi-bin/carddisp.pl?gene=LPIN1
MPI	Mannose Phosphate Isomerase	Protein Coding	45	GC15P074890	12.79825	https://www.genecards.org/cgi-bin/carddisp.pl?gene=MPI
PLCG2	Phospholipase C Gamma 2	Protein Coding	52	GC16P081773	12.79702	https://www.genecards.org/cgi-bin/carddisp.pl?gene=PLCG2
KCNJ2	Potassium Inwardly Rectifying Channel Subfamily J Member 2	Protein Coding	49	GC17P070168	12.79509	https://www.genecards.org/cgi-bin/carddisp.pl?gene=KCNJ2
ANKH	ANKH Inorganic Pyrophosphate Transport Regulator	Protein Coding	41	GC05M014706	12.79484	https://www.genecards.org/cgi-bin/carddisp.pl?gene=ANKH
POLR3A	RNA Polymerase III Subunit A	Protein Coding	44	GC10M077969	12.78629	https://www.genecards.org/cgi-bin/carddisp.pl?gene=POLR3A
FAT4	FAT Atypical Cadherin 4	Protein Coding	40	GC04P125315	12.7849	https://www.genecards.org/cgi-bin/carddisp.pl?gene=FAT4
KLLN	Killin, P53 Regulated DNA Replication Inhibitor	Protein Coding	30	GC10M087860	12.77884	https://www.genecards.org/cgi-bin/carddisp.pl?gene=KLLN
CAPN1	Calpain 1	Protein Coding	50	GC11P065300	12.77332	https://www.genecards.org/cgi-bin/carddisp.pl?gene=CAPN1
PIP5K1C	Phosphatidylinositol-4-Phosphate 5-Kinase Type 1 Gamma	Protein Coding	49	GC19M003631	12.77129	https://www.genecards.org/cgi-bin/carddisp.pl?gene=PIP5K1C
UBC	Ubiquitin C	Protein Coding	44	GC12M124911	12.76761	https://www.genecards.org/cgi-bin/carddisp.pl?gene=UBC
KRIT1	KRIT1 Ankyrin Repeat Containing	Protein Coding	43	GC07M092198	12.76535	https://www.genecards.org/cgi-bin/carddisp.pl?gene=KRIT1
CFLAR	CASP8 And FADD Like Apoptosis Regulator	Protein Coding	47	GC02P201117	12.76239	https://www.genecards.org/cgi-bin/carddisp.pl?gene=CFLAR
IDE	Insulin Degrading Enzyme	Protein Coding	49	GC10M092451	12.75746	https://www.genecards.org/cgi-bin/carddisp.pl?gene=IDE
CRYAB	Crystallin Alpha B	Protein Coding	45	GC11M111908	12.75703	https://www.genecards.org/cgi-bin/carddisp.pl?gene=CRYAB
NIPBL	NIPBL Cohesin Loading Factor	Protein Coding	41	GC05P036876	12.75626	https://www.genecards.org/cgi-bin/carddisp.pl?gene=NIPBL
CTNNA1	Catenin Alpha 1	Protein Coding	48	GC05P138613	12.75517	https://www.genecards.org/cgi-bin/carddisp.pl?gene=CTNNA1
CISH	Cytokine Inducible SH2 Containing Protein	Protein Coding	46	GC03M050618	12.73907	https://www.genecards.org/cgi-bin/carddisp.pl?gene=CISH

DSG2	Desmoglein 2	Protein Coding	47	GC18P031498	12.73005	https://www.genecards.org/cgi-bin/carddisp.pl?gene=DSG2
BLVRB	Biliverdin Reductase B	Protein Coding	41	GC19M040447	12.72987	https://www.genecards.org/cgi-bin/carddisp.pl?gene=BLVRB
AKR1C3	Aldo-Keto Reductase Family 1 Member C3	Protein Coding	47	GC10P005035	12.72881	https://www.genecards.org/cgi-bin/carddisp.pl?gene=AKR1C3
SCD5	Stearoyl-CoA Desaturase 5	Protein Coding	42	GC04M082629	12.72282	https://www.genecards.org/cgi-bin/carddisp.pl?gene=SCD5
ARPC1B	Actin Related Protein 2/3 Complex Subunit 1B	Protein Coding	44	GC07P099374	12.70459	https://www.genecards.org/cgi-bin/carddisp.pl?gene=ARPC1B
CLTC	Clathrin Heavy Chain	Protein Coding	47	GC17P059619	12.70436	https://www.genecards.org/cgi-bin/carddisp.pl?gene=CLTC
NR0B1	Nuclear Receptor Subfamily 0 Group B Member 1	Protein Coding	47	GC0XM030304	12.69775	https://www.genecards.org/cgi-bin/carddisp.pl?gene=NR0B1
GPBAR1	G Protein-Coupled Bile Acid Receptor 1	Protein Coding	40	GC02P218259	12.69684	https://www.genecards.org/cgi-bin/carddisp.pl?gene=GPBAR1
RUNX3	RUNX Family Transcription Factor 3	Protein Coding	44	GC01M024899	12.69664	https://www.genecards.org/cgi-bin/carddisp.pl?gene=RUNX3
SPTBN2	Spectrin Beta, Non-Erythrocytic 2	Protein Coding	43	GC11M069493	12.68712	https://www.genecards.org/cgi-bin/carddisp.pl?gene=SPTBN2
COL6A2	Collagen Type VI Alpha 2 Chain	Protein Coding	44	GC21P046098	12.67904	https://www.genecards.org/cgi-bin/carddisp.pl?gene=COL6A2
PTX3	Pentraxin 3	Protein Coding	43	GC03P157436	12.67726	https://www.genecards.org/cgi-bin/carddisp.pl?gene=PTX3
COQ8B	Coenzyme Q8B	Protein Coding	33	GC19M046773	12.67634	https://www.genecards.org/cgi-bin/carddisp.pl?gene=COQ8B
PER2	Period Circadian Regulator 2	Protein Coding	44	GC02M238244	12.6759	https://www.genecards.org/cgi-bin/carddisp.pl?gene=PER2
NR5A1	Nuclear Receptor Subfamily 5 Group A Member 1	Protein Coding	51	GC09M124481	12.67102	https://www.genecards.org/cgi-bin/carddisp.pl?gene=NR5A1
GSK3A	Glycogen Synthase Kinase 3 Alpha	Protein Coding	50	GC19M046852	12.66287	https://www.genecards.org/cgi-bin/carddisp.pl?gene=GSK3A
OPA3	Outer Mitochondrial Membrane Lipid Metabolism Regulator OPA3	Protein Coding	40	GC19M045527	12.66028	https://www.genecards.org/cgi-bin/carddisp.pl?gene=OPA3
SF3B1	Splicing Factor 3b Subunit 1	Protein Coding	45	GC02M197393	12.65619	https://www.genecards.org/cgi-bin/carddisp.pl?gene=SF3B1

PRKCE	Protein Kinase C Epsilon	Protein Coding	50	GC02P045651	12.65428	https://www.genecards.org/cgi-bin/carddisp.pl?gene=PRKCE
BID	BH3 Interacting Domain Death Agonist	Protein Coding	46	GC22M017734	12.63587	https://www.genecards.org/cgi-bin/carddisp.pl?gene=BID
BCL6	BCL6 Transcription Repressor	Protein Coding	45	GC03M187721	12.63439	https://www.genecards.org/cgi-bin/carddisp.pl?gene=BCL6
PAX5	Paired Box 5	Protein Coding	47	GC09M036828	12.62249	https://www.genecards.org/cgi-bin/carddisp.pl?gene=PAX5
CTH	Cystathionine Gamma-Lyase	Protein Coding	50	GC01P070411	12.61815	https://www.genecards.org/cgi-bin/carddisp.pl?gene=CTH
WASHC5	WASH Complex Subunit 5	Protein Coding	32	GC08M129230	12.61027	https://www.genecards.org/cgi-bin/carddisp.pl?gene=WASHC5
TICAM1	Toll Like Receptor Adaptor Molecule 1	Protein Coding	45	GC19M004815	12.6025	https://www.genecards.org/cgi-bin/carddisp.pl?gene=TICAM1
DSC2	Desmocollin 2	Protein Coding	46	GC18M031058	12.59691	https://www.genecards.org/cgi-bin/carddisp.pl?gene=DSC2
EIF4EBP1	Eukaryotic Translation Initiation Factor 4E Binding Protein 1	Protein Coding	47	GC08P038032	12.58926	https://www.genecards.org/cgi-bin/carddisp.pl?gene=EIF4EBP1
TUBA4A	Tubulin Alpha 4a	Protein Coding	46	GC02M219249	12.58491	https://www.genecards.org/cgi-bin/carddisp.pl?gene=TUBA4A
FOXC2	Forkhead Box C2	Protein Coding	45	GC16P086570	12.58415	https://www.genecards.org/cgi-bin/carddisp.pl?gene=FOXC2
MIR338	MicroRNA 338	RNA Gene	17	GC17M081126	12.58185	https://www.genecards.org/cgi-bin/carddisp.pl?gene=MIR338
CXCR2	C-X-C Motif Chemokine Receptor 2	Protein Coding	48	GC02P218125	12.57916	https://www.genecards.org/cgi-bin/carddisp.pl?gene=CXCR2
SLC12A6	Solute Carrier Family 12 Member 6	Protein Coding	49	GC15M034229	12.57227	https://www.genecards.org/cgi-bin/carddisp.pl?gene=SLC12A6
MIR154	MicroRNA 154	RNA Gene	18	GC14P106303	12.56909	https://www.genecards.org/cgi-bin/carddisp.pl?gene=MIR154
DHCR24	24-Dehydrocholesterol Reductase	Protein Coding	46	GC01M054849	12.56117	https://www.genecards.org/cgi-bin/carddisp.pl?gene=DHCR24
CPQ	Carboxypeptidase Q	Protein Coding	34	GC08P096645	12.55549	https://www.genecards.org/cgi-bin/carddisp.pl?gene=CPQ
FLNB	Filamin B	Protein Coding	47	GC03P058008	12.54686	https://www.genecards.org/cgi-bin/carddisp.pl?gene=FLNB
IAPP	Islet Amyloid Polypeptide	Protein Coding	42	GC12P021354	12.53185	https://www.genecards.org/cgi-bin/carddisp.pl?gene=IAPP
GPAM	Glycerol-3-Phosphate Acyltransferase, Mitochondrial	Protein Coding	43	GC10M112148	12.4958	https://www.genecards.org/cgi-bin/carddisp.pl?gene=GPAM

CHRM2	Cholinergic Receptor Muscarinic 2	Protein Coding	49	GC07P136868	12.48891	https://www.genecards.org/cgi-bin/carddisp.pl?gene=CHRM2
MYO5A	Myosin VA	Protein Coding	45	GC15M067802	12.48175	https://www.genecards.org/cgi-bin/carddisp.pl?gene=MYO5A
SLC1A3	Solute Carrier Family 1 Member 3	Protein Coding	51	GC05P036620	12.47509	https://www.genecards.org/cgi-bin/carddisp.pl?gene=SLC1A3
SLC34A3	Solute Carrier Family 34 Member 3	Protein Coding	42	GC09P137230	12.47387	https://www.genecards.org/cgi-bin/carddisp.pl?gene=SLC34A3
IARS1	Isoleucyl-TRNA Synthetase 1	Protein Coding	36	GC09M092211	12.47056	https://www.genecards.org/cgi-bin/carddisp.pl?gene=IARS1
UBB	Ubiquitin B	Protein Coding	44	GC17P016380	12.46593	https://www.genecards.org/cgi-bin/carddisp.pl?gene=UBB
DCN	Decorin	Protein Coding	47	GC12M091140	12.45364	https://www.genecards.org/cgi-bin/carddisp.pl?gene=DCN
MICA	MHC Class I Polypeptide-Related Sequence A	Protein Coding	39	GC06P031399	12.45189	https://www.genecards.org/cgi-bin/carddisp.pl?gene=MICA
CD69	CD69 Molecule	Protein Coding	41	GC12M015678	12.44717	https://www.genecards.org/cgi-bin/carddisp.pl?gene=CD69
CTSG	Cathepsin G	Protein Coding	45	GC14M024573	12.42989	https://www.genecards.org/cgi-bin/carddisp.pl?gene=CTSG
ASCL1	Achaete-Scute Family BHLH Transcription Factor 1	Protein Coding	45	GC12P102957	12.41847	https://www.genecards.org/cgi-bin/carddisp.pl?gene=ASCL1
COG6	Component Of Oligomeric Golgi Complex 6	Protein Coding	38	GC13P039655	12.40854	https://www.genecards.org/cgi-bin/carddisp.pl?gene=COG6
USP8	Ubiquitin Specific Peptidase 8	Protein Coding	49	GC15P050424	12.40557	https://www.genecards.org/cgi-bin/carddisp.pl?gene=USP8
FMO3	Flavin Containing Dimethylaniline Monoxygenase 3	Protein Coding	47	GC01P171090	12.4014	https://www.genecards.org/cgi-bin/carddisp.pl?gene=FMO3
BUB1	BUB1 Mitotic Checkpoint Serine/Threonine Kinase	Protein Coding	50	GC02M110637	12.39558	https://www.genecards.org/cgi-bin/carddisp.pl?gene=BUB1
MGP	Matrix Gla Protein	Protein Coding	43	GC12M015756	12.39504	https://www.genecards.org/cgi-bin/carddisp.pl?gene=MGP
WNT3	Wnt Family Member 3	Protein Coding	47	GC17M046762	12.39017	https://www.genecards.org/cgi-bin/carddisp.pl?gene=WNT3
KMT2C	Lysine Methyltransferase 2C	Protein Coding	42	GC07M152134	12.38634	https://www.genecards.org/cgi-bin/carddisp.pl?gene=KMT2C
AMH	Anti-Mullerian Hormone	Protein Coding	44	GC19P002251	12.3841	https://www.genecards.org/cgi-bin/carddisp.pl?gene=AMH
TP73	Tumor Protein P73	Protein Coding	46	GC01P003652	12.38259	https://www.genecards.org/cgi-bin/carddisp.pl?gene=TP73

BIRC3	Baculoviral IAP Repeat Containing 3	Protein Coding	47	GC11P102317	12.36399	https://www.genecards.org/cgi-bin/carddisp.pl?gene=BIRC3
C11orf65	Chromosome 11 Open Reading Frame 65	Protein Coding	31	GC11M108308	12.36275	https://www.genecards.org/cgi-bin/carddisp.pl?gene=C11orf65
ARL6	ADP Ribosylation Factor Like GTPase 6	Protein Coding	43	GC03P097764	12.35234	https://www.genecards.org/cgi-bin/carddisp.pl?gene=ARL6
RBPJ	Recombination Signal Binding Protein For Immunoglobulin Kappa J Region	Protein Coding	48	GC04P026165	12.34941	https://www.genecards.org/cgi-bin/carddisp.pl?gene=RBPJ
CD2AP	CD2 Associated Protein	Protein Coding	44	GC06P055437	12.34787	https://www.genecards.org/cgi-bin/carddisp.pl?gene=CD2AP
PVT1	Pvt1 Oncogene	RNA Gene	24	GC08P127822	12.34716	https://www.genecards.org/cgi-bin/carddisp.pl?gene=PVT1
GABRA3	Gamma-Aminobutyric Acid Type A Receptor Subunit Alpha3	Protein Coding	45	GC0XM152166	12.34649	https://www.genecards.org/cgi-bin/carddisp.pl?gene=GABRA3
CERS1	Ceramide Synthase 1	Protein Coding	41	GC19M018868	12.338	https://www.genecards.org/cgi-bin/carddisp.pl?gene=CERS1
RAD51D	RAD51 Paralog D	Protein Coding	41	GC17M035092	12.32848	https://www.genecards.org/cgi-bin/carddisp.pl?gene=RAD51D
ATP5F1E	ATP Synthase F1 Subunit Epsilon	Protein Coding	32	GC20M059026	12.31955	https://www.genecards.org/cgi-bin/carddisp.pl?gene=ATP5F1E
ADAMTS4	ADAM Metallopeptidase With Thrombospondin Type 1 Motif 4	Protein Coding	43	GC01M161184	12.31253	https://www.genecards.org/cgi-bin/carddisp.pl?gene=ADAMTS4
GRHPR	Glyoxylate And Hydroxypyruvate Reductase	Protein Coding	47	GC09P037412	12.3089	https://www.genecards.org/cgi-bin/carddisp.pl?gene=GRHPR
TBX20	T-Box Transcription Factor 20	Protein Coding	43	GC07M035237	12.30676	https://www.genecards.org/cgi-bin/carddisp.pl?gene=TBX20
GJB3	Gap Junction Protein Beta 3	Protein Coding	45	GC01P034781	12.30407	https://www.genecards.org/cgi-bin/carddisp.pl?gene=GJB3
BPI	Bactericidal Permeability Increasing Protein	Protein Coding	42	GC20P038304	12.30249	https://www.genecards.org/cgi-bin/carddisp.pl?gene=BPI

MBTPS2	Membrane Bound Transcription Factor Peptidase, Site 2	Protein Coding	44	GC0XP021839	12.30023	https://www.genecards.org/cgi-bin/carddisp.pl?gene=MBTPS2
MIR26B	MicroRNA 26b	RNA Gene	21	GC02P218402	12.29175	https://www.genecards.org/cgi-bin/carddisp.pl?gene=MIR26B
ICOS	Inducible T Cell Costimulator	Protein Coding	43	GC02P203937	12.28773	https://www.genecards.org/cgi-bin/carddisp.pl?gene=ICOS
STUB1	STIP1 Homology And U-Box Containing Protein 1	Protein Coding	46	GC16P005516	12.28294	https://www.genecards.org/cgi-bin/carddisp.pl?gene=STUB1
MIR212	MicroRNA 212	RNA Gene	19	GC17M002050	12.27748	https://www.genecards.org/cgi-bin/carddisp.pl?gene=MIR212
TLR7	Toll Like Receptor 7	Protein Coding	48	GC0XP012867	12.27539	https://www.genecards.org/cgi-bin/carddisp.pl?gene=TLR7
CXCL9	C-X-C Motif Chemokine Ligand 9	Protein Coding	39	GC04M076001	12.27432	https://www.genecards.org/cgi-bin/carddisp.pl?gene=CXCL9
MX1	MX Dynamin Like GTPase 1	Protein Coding	43	GC21P041420	12.27297	https://www.genecards.org/cgi-bin/carddisp.pl?gene=MX1
HBEGF	Heparin Binding EGF Like Growth Factor	Protein Coding	44	GC05M140332	12.27253	https://www.genecards.org/cgi-bin/carddisp.pl?gene=HBEGF
GAS5	Growth Arrest Specific 5	RNA Gene	23	GC01M173947	12.26382	https://www.genecards.org/cgi-bin/carddisp.pl?gene=GAS5
RPL5	Ribosomal Protein L5	Protein Coding	48	GC01P092832	12.26073	https://www.genecards.org/cgi-bin/carddisp.pl?gene=RPL5
PHGDH	Phosphoglycerate Dehydrogenase	Protein Coding	50	GC01P119660	12.24876	https://www.genecards.org/cgi-bin/carddisp.pl?gene=PHGDH
MOG	Myelin Oligodendrocyte Glycoprotein	Protein Coding	46	GC06P055140	12.23808	https://www.genecards.org/cgi-bin/carddisp.pl?gene=MOG
GRP	Gastrin Releasing Peptide	Protein Coding	41	GC18P059220	12.23281	https://www.genecards.org/cgi-bin/carddisp.pl?gene=GRP
COX20	Cytochrome C Oxidase Assembly Factor COX20	Protein Coding	38	GC01P244839	12.2285	https://www.genecards.org/cgi-bin/carddisp.pl?gene=COX20
FOLH1	Folate Hydrolase 1	Protein Coding	47	GC11M069030	12.21309	https://www.genecards.org/cgi-bin/carddisp.pl?gene=FOLH1
JMJD1C	Jumonji Domain Containing 1C	Protein Coding	41	GC10M063167	12.2083	https://www.genecards.org/cgi-bin/carddisp.pl?gene=JMJD1C
MT-TW	Mitochondrially Encoded TRNA-Trp (UGA/G)	RNA Gene	13	GCMTP005514	12.19986	https://www.genecards.org/cgi-bin/carddisp.pl?gene=MT-TW

JPH1	Junctophilin 1	Protein Coding	39	GC08M074234	12.194	https://www.genecards.org/cgi-bin/carddisp.pl?gene=JPH1
RETREG1	Reticulophagy Regulator 1	Protein Coding	31	GC05M016472	12.19262	https://www.genecards.org/cgi-bin/carddisp.pl?gene=RETREG1
LNCARSR	LncRNA Regulator Of Akt Signaling Associated With HCC And RCC	RNA Gene	11	GC09M079507	12.19117	https://www.genecards.org/cgi-bin/carddisp.pl?gene=LNCARSR
BARD1	BRCA1 Associated RING Domain 1	Protein Coding	47	GC02M214725	12.19033	https://www.genecards.org/cgi-bin/carddisp.pl?gene=BARD1
APPL1	Adaptor Protein, Phosphotyrosine Interacting With PH Domain And Leucine Zipper 1	Protein Coding	46	GC03P057227	12.18964	https://www.genecards.org/cgi-bin/carddisp.pl?gene=APPL1
CYP2J2	Cytochrome P450 Family 2 Subfamily J Member 2	Protein Coding	45	GC01M059893	12.18659	https://www.genecards.org/cgi-bin/carddisp.pl?gene=CYP2J2
HUWE1	HECT, UBA And WWE Domain Containing E3 Ubiquitin Protein Ligase 1	Protein Coding	44	GC0XM053532	12.18521	https://www.genecards.org/cgi-bin/carddisp.pl?gene=HUWE1
SLC25A19	Solute Carrier Family 25 Member 19	Protein Coding	44	GC17M075273	12.18481	https://www.genecards.org/cgi-bin/carddisp.pl?gene=SLC25A19
TUBA1B	Tubulin Alpha 1b	Protein Coding	43	GC12M049127	12.18077	https://www.genecards.org/cgi-bin/carddisp.pl?gene=TUBA1B
PPM1D	Protein Phosphatase, Mg2+/Mn2+ Dependent 1D	Protein Coding	48	GC17P060600	12.17867	https://www.genecards.org/cgi-bin/carddisp.pl?gene=PPM1D
GREM1	Gremlin 1, DAN Family BMP Antagonist	Protein Coding	44	GC15P033128	12.17686	https://www.genecards.org/cgi-bin/carddisp.pl?gene=GREM1
LIMK1	LIM Domain Kinase 1	Protein Coding	50	GC07P074082	12.1734	https://www.genecards.org/cgi-bin/carddisp.pl?gene=LIMK1
GABBR2	Gamma-Aminobutyric Acid Type B Receptor Subunit 2	Protein Coding	49	GC09M098288	12.16758	https://www.genecards.org/cgi-bin/carddisp.pl?gene=GABBR2
CNR2	Cannabinoid Receptor 2	Protein Coding	46	GC01M023870	12.16039	https://www.genecards.org/cgi-bin/carddisp.pl?gene=CNR2
CAPN10	Calpain 10	Protein Coding	42	GC02P240586	12.15099	https://www.genecards.org/cgi-bin/carddisp.pl?gene=CAPN10

CAMK2G	Calcium/Calmodulin Dependent Protein Kinase II Gamma	Protein Coding	48	GC10M073812	12.15047	https://www.genecards.org/cgi-bin/carddisp.pl?gene=CAMK2G
CALM1	Calmodulin 1	Protein Coding	45	GC14P090396	12.14745	https://www.genecards.org/cgi-bin/carddisp.pl?gene=CALM1
BLOC1S1	Biogenesis Of Lysosomal Organelles Complex 1 Subunit 1	Protein Coding	36	GC12P055765	12.14541	https://www.genecards.org/cgi-bin/carddisp.pl?gene=BLOC1S1
COX6A2	Cytochrome C Oxidase Subunit 6A2	Protein Coding	40	GC16M031439	12.13695	https://www.genecards.org/cgi-bin/carddisp.pl?gene=COX6A2
SUCLA2	Succinate-CoA Ligase ADP-Forming Subunit Beta	Protein Coding	47	GC13M047745	12.13319	https://www.genecards.org/cgi-bin/carddisp.pl?gene=SUCLA2
TRAF3	TNF Receptor Associated Factor 3	Protein Coding	48	GC14P106036	12.12416	https://www.genecards.org/cgi-bin/carddisp.pl?gene=TRAF3
AASS	Aminoadipate-Semialdehyde Synthase	Protein Coding	45	GC07M122073	12.11934	https://www.genecards.org/cgi-bin/carddisp.pl?gene=AASS
F2R	Coagulation Factor II Thrombin Receptor	Protein Coding	47	GC05P076716	12.11281	https://www.genecards.org/cgi-bin/carddisp.pl?gene=F2R
HDAC4	Histone Deacetylase 4	Protein Coding	52	GC02M239048	12.10809	https://www.genecards.org/cgi-bin/carddisp.pl?gene=HDAC4
LECT2	Leukocyte Cell Derived Chemotaxin 2	Protein Coding	37	GC05M135922	12.10747	https://www.genecards.org/cgi-bin/carddisp.pl?gene=LECT2
ATF2	Activating Transcription Factor 2	Protein Coding	47	GC02M175072	12.10292	https://www.genecards.org/cgi-bin/carddisp.pl?gene=ATF2
BCL2L11	BCL2 Like 11	Protein Coding	45	GC02P111119	12.10106	https://www.genecards.org/cgi-bin/carddisp.pl?gene=BCL2L11
ALDH7A1	Aldehyde Dehydrogenase 7 Family Member A1	Protein Coding	48	GC05M126541	12.09786	https://www.genecards.org/cgi-bin/carddisp.pl?gene=ALDH7A1
KANSL1	KAT8 Regulatory NSL Complex Subunit 1	Protein Coding	39	GC17M046031	12.08863	https://www.genecards.org/cgi-bin/carddisp.pl?gene=KANSL1
CTTN	Cortactin	Protein Coding	44	GC11P070398	12.08824	https://www.genecards.org/cgi-bin/carddisp.pl?gene=CTTN
SPTAN1	Spectrin Alpha, Non-Erythrocytic 1	Protein Coding	48	GC09P128552	12.0822	https://www.genecards.org/cgi-bin/carddisp.pl?gene=SPTAN1
SCAP	SREBF Chaperone	Protein Coding	41	GC03M047413	12.07995	https://www.genecards.org/cgi-bin/carddisp.pl?gene=SCAP
BGN	Biglycan	Protein Coding	43	GC0XP153494	12.07963	https://www.genecards.org/cgi-bin/carddisp.pl?gene=BGN
MIR193A	MicroRNA 193a	RNA Gene	18	GC17P031559	12.07841	https://www.genecards.org/cgi-bin/carddisp.pl?gene=MIR193A

SLC25A1	Solute Carrier Family 25 Member 1	Protein Coding	47	GC22M019177	12.06457	https://www.genecards.org/cgi-bin/carddisp.pl?gene=SLC25A1
DEPDC5	DEP Domain Containing 5, GATOR1 Subcomplex Subunit	Protein Coding	40	GC22P031753	12.06069	https://www.genecards.org/cgi-bin/carddisp.pl?gene=DEPDC5
IRF7	Interferon Regulatory Factor 7	Protein Coding	47	GC11M000612	12.05842	https://www.genecards.org/cgi-bin/carddisp.pl?gene=IRF7
TMEM165	Transmembrane Protein 165	Protein Coding	39	GC04P055395	12.05321	https://www.genecards.org/cgi-bin/carddisp.pl?gene=TMEM165
VCAN	Versican	Protein Coding	47	GC05P083471	12.05259	https://www.genecards.org/cgi-bin/carddisp.pl?gene=VCAN
VPS13A	Vacuolar Protein Sorting 13 Homolog A	Protein Coding	41	GC09P077177	12.04441	https://www.genecards.org/cgi-bin/carddisp.pl?gene=VPS13A
GYPA	Glycophorin A (MNS Blood Group)	Protein Coding	45	GC04M144109	12.04391	https://www.genecards.org/cgi-bin/carddisp.pl?gene=GYPA
HAX1	HCLS1 Associated Protein X-1	Protein Coding	43	GC01P154273	12.02726	https://www.genecards.org/cgi-bin/carddisp.pl?gene=HAX1
IGF2BP2	Insulin Like Growth Factor 2 MRNA Binding Protein 2	Protein Coding	44	GC03M185643	12.01956	https://www.genecards.org/cgi-bin/carddisp.pl?gene=IGF2BP2
SGCB	Sarcoglycan Beta	Protein Coding	41	GC04M052019	12.01912	https://www.genecards.org/cgi-bin/carddisp.pl?gene=SGCB
COMP	Cartilage Oligomeric Matrix Protein	Protein Coding	48	GC19M018783	12.01571	https://www.genecards.org/cgi-bin/carddisp.pl?gene=COMP
MYOD1	Myogenic Differentiation 1	Protein Coding	47	GC11P017741	12.01547	https://www.genecards.org/cgi-bin/carddisp.pl?gene=MYOD1
PTK2B	Protein Tyrosine Kinase 2 Beta	Protein Coding	50	GC08P027311	12.00551	https://www.genecards.org/cgi-bin/carddisp.pl?gene=PTK2B
ALOXE3	Arachidonate Lipoxygenase 3	Protein Coding	43	GC17M009179	12.00467	https://www.genecards.org/cgi-bin/carddisp.pl?gene=ALOXE3
SKI	SKI Proto-Oncogene	Protein Coding	45	GC01P002228	12.00082	https://www.genecards.org/cgi-bin/carddisp.pl?gene=SKI
TMEM43	Transmembrane Protein 43	Protein Coding	41	GC03P014124	11.99548	https://www.genecards.org/cgi-bin/carddisp.pl?gene=TMEM43
SLC2A9	Solute Carrier Family 2 Member 9	Protein Coding	46	GC04M009772	11.9935	https://www.genecards.org/cgi-bin/carddisp.pl?gene=SLC2A9
WRN	WRN RecQ Like Helicase	Protein Coding	45	GC08P031033	11.99336	https://www.genecards.org/cgi-bin/carddisp.pl?gene=WRN

MTHFD1	Methylenetetrahydrofolate Dehydrogenase, Cyclohydrolase And Formyltetrahydrofolate Synthetase 1	Protein Coding	45	GC14P064388	11.99127	https://www.genecards.org/cgi-bin/carddisp.pl?gene=MTHFD1
PCNT	Pericentrin	Protein Coding	42	GC21P046324	11.98215	https://www.genecards.org/cgi-bin/carddisp.pl?gene=PCNT
ACO1	Aconitase 1	Protein Coding	45	GC09P032374	11.98141	https://www.genecards.org/cgi-bin/carddisp.pl?gene=ACO1
GLDC	Glycine Decarboxylase	Protein Coding	48	GC09M006522	11.97592	https://www.genecards.org/cgi-bin/carddisp.pl?gene=GLDC
TRMT10C	TRNA Methyltransferase 10C, Mitochondrial RNase P Subunit	Protein Coding	36	GC03P101561	11.97461	https://www.genecards.org/cgi-bin/carddisp.pl?gene=TRMT10C
DOCK6	Dedicator Of Cytokinesis 6	Protein Coding	40	GC19M011199	11.97164	https://www.genecards.org/cgi-bin/carddisp.pl?gene=DOCK6
ATPAF2	ATP Synthase Mitochondrial F1 Complex Assembly Factor 2	Protein Coding	38	GC17M017977	11.97006	https://www.genecards.org/cgi-bin/carddisp.pl?gene=ATPAF2
PLA2G5	Phospholipase A2 Group V	Protein Coding	44	GC01P020028	11.96537	https://www.genecards.org/cgi-bin/carddisp.pl?gene=PLA2G5
NDUFB10	NADH:Ubiquinone Oxidoreductase Subunit B10	Protein Coding	44	GC16P005573	11.96009	https://www.genecards.org/cgi-bin/carddisp.pl?gene=NDUFB10
EPG5	Ectopic P-Granules Autophagy Protein 5 Homolog	Protein Coding	37	GC18M045800	11.95972	https://www.genecards.org/cgi-bin/carddisp.pl?gene=EPG5
CNP	2',3'-Cyclic Nucleotide 3' Phosphodiesterase	Protein Coding	44	GC17P041966	11.95895	https://www.genecards.org/cgi-bin/carddisp.pl?gene=CNP
OPRD1	Opioid Receptor Delta 1	Protein Coding	45	GC01P028812	11.95273	https://www.genecards.org/cgi-bin/carddisp.pl?gene=OPRD1
CCDC47	Coiled-Coil Domain Containing 47	Protein Coding	36	GC17M063745	11.94775	https://www.genecards.org/cgi-bin/carddisp.pl?gene=CCDC47
HAO1	Hydroxyacid Oxidase 1	Protein Coding	44	GC20M007863	11.93902	https://www.genecards.org/cgi-bin/carddisp.pl?gene=HAO1
FGL1	Fibrinogen Like 1	Protein Coding	41	GC08M017864	11.93853	https://www.genecards.org/cgi-bin/carddisp.pl?gene=FGL1
CDK8	Cyclin Dependent Kinase 8	Protein Coding	48	GC13P026254	11.93615	https://www.genecards.org/cgi-bin/carddisp.pl?gene=CDK8
DNM1	Dynamin 1	Protein Coding	51	GC09P128191	11.92584	https://www.genecards.org/cgi-bin/carddisp.pl?gene=DNM1

BIN1	Bridging Integrator 1	Protein Coding	47	GC02M127048	11.92172	https://www.genecards.org/cgi-bin/carddisp.pl?gene=BIN1
LIPF	Lipase F, Gastric Type	Protein Coding	42	GC10P088664	11.91782	https://www.genecards.org/cgi-bin/carddisp.pl?gene=LIPF
FGF7	Fibroblast Growth Factor 7	Protein Coding	43	GC15P049423	11.91234	https://www.genecards.org/cgi-bin/carddisp.pl?gene=FGF7
LIN28B	Lin-28 Homolog B	Protein Coding	43	GC06P104943	11.91173	https://www.genecards.org/cgi-bin/carddisp.pl?gene=LIN28B
PCYT1A	Phosphate Cytidylyltransferase 1A, Choline	Protein Coding	47	GC03M196214	11.88505	https://www.genecards.org/cgi-bin/carddisp.pl?gene=PCYT1A
MC2R	Melanocortin 2 Receptor	Protein Coding	48	GC18M019097	11.87764	https://www.genecards.org/cgi-bin/carddisp.pl?gene=MC2R
HNRNPU	Heterogeneous Nuclear Ribonucleoprotein U	Protein Coding	43	GC01M244844	11.86412	https://www.genecards.org/cgi-bin/carddisp.pl?gene=HNRNPU
TYRP1	Tyrosinase Related Protein 1	Protein Coding	47	GC09P012683	11.86264	https://www.genecards.org/cgi-bin/carddisp.pl?gene=TYRP1
NUS1	NUS1 Dehydrodolichyl Diphosphate Synthase Subunit	Protein Coding	40	GC06P117675	11.85999	https://www.genecards.org/cgi-bin/carddisp.pl?gene=NUS1
KAT6B	Lysine Acetyltransferase 6B	Protein Coding	43	GC10P074840	11.85551	https://www.genecards.org/cgi-bin/carddisp.pl?gene=KAT6B
CD38	CD38 Molecule	Protein Coding	45	GC04P015779	11.85372	https://www.genecards.org/cgi-bin/carddisp.pl?gene=CD38
EEF1A2	Eukaryotic Translation Elongation Factor 1 Alpha 2	Protein Coding	47	GC20M063488	11.85248	https://www.genecards.org/cgi-bin/carddisp.pl?gene=EEF1A2
BBIP1	BBSome Interacting Protein 1	Protein Coding	32	GC10M110898	11.85005	https://www.genecards.org/cgi-bin/carddisp.pl?gene=BBIP1
TAFAZZIN	Tafazzin, Phospholipid-Lysophospholipid Transacylase	Protein Coding	34	GC0XP154413	11.84665	https://www.genecards.org/cgi-bin/carddisp.pl?gene=TAFAZZIN
VPS11	VPS11 Core Subunit Of CORVET And HOPS Complexes	Protein Coding	42	GC11P119067	11.83394	https://www.genecards.org/cgi-bin/carddisp.pl?gene=VPS11
PLEK	Pleckstrin	Protein Coding	40	GC02P068365	11.82782	https://www.genecards.org/cgi-bin/carddisp.pl?gene=PLEK
SLC6A8	Solute Carrier Family 6 Member 8	Protein Coding	46	GC0XP153688	11.82304	https://www.genecards.org/cgi-bin/carddisp.pl?gene=SLC6A8
MIR128-2	MicroRNA 128-2	RNA Gene	20	GC03P035750	11.82282	https://www.genecards.org/cgi-bin/carddisp.pl?gene=MIR128-2

PTPA	Protein Phosphatase 2 Phosphatase Activator	Protein Coding	36	GC09P129111	11.82228	https://www.genecards.org/cgi-bin/carddisp.pl?gene=PTPA
TAP2	Transporter 2, ATP Binding Cassette Subfamily B Member	Protein Coding	45	GC06M032821	11.81733	https://www.genecards.org/cgi-bin/carddisp.pl?gene=TAP2
RIF1	Replication Timing Regulatory Factor 1	Protein Coding	40	GC02P151409	11.81404	https://www.genecards.org/cgi-bin/carddisp.pl?gene=RIF1
NCOR1	Nuclear Receptor Corepressor 1	Protein Coding	44	GC17M016029	11.80752	https://www.genecards.org/cgi-bin/carddisp.pl?gene=NCOR1
TNXB	Tenascin XB	Protein Coding	44	GC06M046900	11.80141	https://www.genecards.org/cgi-bin/carddisp.pl?gene=TNXB
ARF1	ADP Ribosylation Factor 1	Protein Coding	48	GC01P228082	11.80075	https://www.genecards.org/cgi-bin/carddisp.pl?gene=ARF1
THRA	Thyroid Hormone Receptor Alpha	Protein Coding	50	GC17P040058	11.79574	https://www.genecards.org/cgi-bin/carddisp.pl?gene=THRA
ATL1	Atlastin GTPase 1	Protein Coding	42	GC14P050532	11.79336	https://www.genecards.org/cgi-bin/carddisp.pl?gene=ATL1
TIA1	TIA1 Cytotoxic Granule Associated RNA Binding Protein	Protein Coding	44	GC02M070209	11.79236	https://www.genecards.org/cgi-bin/carddisp.pl?gene=TIA1
ZNF423	Zinc Finger Protein 423	Protein Coding	40	GC16M049487	11.77463	https://www.genecards.org/cgi-bin/carddisp.pl?gene=ZNF423
IL16	Interleukin 16	Protein Coding	43	GC15P081159	11.77269	https://www.genecards.org/cgi-bin/carddisp.pl?gene=IL16
FGF1	Fibroblast Growth Factor 1	Protein Coding	48	GC05M142555	11.76774	https://www.genecards.org/cgi-bin/carddisp.pl?gene=FGF1
KRT20	Keratin 20	Protein Coding	43	GC17M040875	11.75627	https://www.genecards.org/cgi-bin/carddisp.pl?gene=KRT20
KRT16	Keratin 16	Protein Coding	45	GC17M041609	11.75171	https://www.genecards.org/cgi-bin/carddisp.pl?gene=KRT16
MAP2K2	Mitogen-Activated Protein Kinase Kinase 2	Protein Coding	54	GC19M004090	11.74303	https://www.genecards.org/cgi-bin/carddisp.pl?gene=MAP2K2
BCAP31	B Cell Receptor Associated Protein 31	Protein Coding	41	GC0XM153701	11.71626	https://www.genecards.org/cgi-bin/carddisp.pl?gene=BCAP31
PALLD	Palladin, Cytoskeletal Associated Protein	Protein Coding	44	GC04P168497	11.71264	https://www.genecards.org/cgi-bin/carddisp.pl?gene=PALLD
STS	Steroid Sulfatase	Protein Coding	45	GC0XP007146	11.70275	https://www.genecards.org/cgi-bin/carddisp.pl?gene=STS

MYL2	Myosin Light Chain 2	Protein Coding	50	GC12M110910	11.69952	https://www.genecards.org/cgi-bin/carddisp.pl?gene=MYL2
PRDX5	Peroxiredoxin 5	Protein Coding	47	GC11P064317	11.69821	https://www.genecards.org/cgi-bin/carddisp.pl?gene=PRDX5
MCCC1	Methylcrotonyl-CoA Carboxylase Subunit 1	Protein Coding	44	GC03M183015	11.69649	https://www.genecards.org/cgi-bin/carddisp.pl?gene=MCCC1
GMPPA	GDP-Mannose Pyrophosphorylase A	Protein Coding	43	GC02P219498	11.69284	https://www.genecards.org/cgi-bin/carddisp.pl?gene=GMPPA
TUBB3	Tubulin Beta 3 Class III	Protein Coding	50	GC16P089919	11.68356	https://www.genecards.org/cgi-bin/carddisp.pl?gene=TUBB3
PPP2R1A	Protein Phosphatase 2 Scaffold Subunit Aalpha	Protein Coding	48	GC19P052189	11.67043	https://www.genecards.org/cgi-bin/carddisp.pl?gene=PPP2R1A
MIRLET7A3	MicroRNA Let-7a-3	RNA Gene	19	GC22P046112	11.65926	https://www.genecards.org/cgi-bin/carddisp.pl?gene=MIRLET7A3
BAP1	BRCA1 Associated Protein 1	Protein Coding	47	GC03M052401	11.65662	https://www.genecards.org/cgi-bin/carddisp.pl?gene=BAP1
CRPPA	CDP-L-Ribitol Pyrophosphorylase A	Protein Coding	30	GC07M016088	11.65179	https://www.genecards.org/cgi-bin/carddisp.pl?gene=CRPPA
CYP4F11	Cytochrome P450 Family 4 Subfamily F Member 11	Protein Coding	40	GC19M015912	11.65042	https://www.genecards.org/cgi-bin/carddisp.pl?gene=CYP4F11
GSTM3	Glutathione S-Transferase Mu 3	Protein Coding	46	GC01M109733	11.64908	https://www.genecards.org/cgi-bin/carddisp.pl?gene=GSTM3
LORICRIN	Loricrin Cornified Envelope Precursor Protein	Protein Coding	28	GC01P153262	11.64103	https://www.genecards.org/cgi-bin/carddisp.pl?gene=LORICRIN
TNFSF4	TNF Superfamily Member 4	Protein Coding	41	GC01M173183	11.63899	https://www.genecards.org/cgi-bin/carddisp.pl?gene=TNFSF4
DOCK7	Dedicator Of Cytokinesis 7	Protein Coding	42	GC01M062454	11.63472	https://www.genecards.org/cgi-bin/carddisp.pl?gene=DOCK7
PDE4D	Phosphodiesterase 4D	Protein Coding	49	GC05M058969	11.62312	https://www.genecards.org/cgi-bin/carddisp.pl?gene=PDE4D
ZFHX3	Zinc Finger Homeobox 3	Protein Coding	42	GC16M072782	11.61782	https://www.genecards.org/cgi-bin/carddisp.pl?gene=ZFHX3
ADRB1	Adrenoceptor Beta 1	Protein Coding	49	GC10P114044	11.61673	https://www.genecards.org/cgi-bin/carddisp.pl?gene=ADRB1
RAI1	Retinoic Acid Induced 1	Protein Coding	40	GC17P017682	11.6143	https://www.genecards.org/cgi-bin/carddisp.pl?gene=RAI1
SOD3	Superoxide Dismutase 3	Protein Coding	41	GC04P024798	11.61317	https://www.genecards.org/cgi-bin/carddisp.pl?gene=SOD3
FRZB	Frizzled Related Protein	Protein Coding	43	GC02M182833	11.60966	https://www.genecards.org/cgi-bin/carddisp.pl?gene=FRZB

LEF1	Lymphoid Enhancer Binding Factor 1	Protein Coding	48	GC04M108047	11.60907	https://www.genecards.org/cgi-bin/carddisp.pl?gene=LEF1
SUCLG1	Succinate-CoA Ligase GDP/ADP-Forming Subunit Alpha	Protein Coding	46	GC02M084423	11.60468	https://www.genecards.org/cgi-bin/carddisp.pl?gene=SUCLG1
CFP	Complement Factor Properdin	Protein Coding	44	GC0XM047624	11.60401	https://www.genecards.org/cgi-bin/carddisp.pl?gene=CFP
CD33	CD33 Molecule	Protein Coding	44	GC19P051212	11.5978	https://www.genecards.org/cgi-bin/carddisp.pl?gene=CD33
NOX4	NADPH Oxidase 4	Protein Coding	43	GC11M089324	11.59755	https://www.genecards.org/cgi-bin/carddisp.pl?gene=NOX4
SLC9A3	Solute Carrier Family 9 Member A3	Protein Coding	47	GC05M000472	11.59178	https://www.genecards.org/cgi-bin/carddisp.pl?gene=SLC9A3
AMPD1	Adenosine Monophosphate Deaminase 1	Protein Coding	46	GC01M114673	11.59108	https://www.genecards.org/cgi-bin/carddisp.pl?gene=AMPD1
MAN1B1	Mannosidase Alpha Class 1B Member 1	Protein Coding	47	GC09P137086	11.58369	https://www.genecards.org/cgi-bin/carddisp.pl?gene=MAN1B1
MIR193B	MicroRNA 193b	RNA Gene	20	GC16P014307	11.58105	https://www.genecards.org/cgi-bin/carddisp.pl?gene=MIR193B
SLC22A1	Solute Carrier Family 22 Member 1	Protein Coding	44	GC06P160121	11.58076	https://www.genecards.org/cgi-bin/carddisp.pl?gene=SLC22A1
FOXC1	Forkhead Box C1	Protein Coding	43	GC06P001610	11.58016	https://www.genecards.org/cgi-bin/carddisp.pl?gene=FOXC1
RAD54L	RAD54 Like	Protein Coding	47	GC01P046278	11.57112	https://www.genecards.org/cgi-bin/carddisp.pl?gene=RAD54L
COX7A2	Cytochrome C Oxidase Subunit 7A2	Protein Coding	39	GC06M075237	11.56741	https://www.genecards.org/cgi-bin/carddisp.pl?gene=COX7A2
DSPP	Dentin Sialophosphoprotein	Protein Coding	38	GC04P087608	11.56214	https://www.genecards.org/cgi-bin/carddisp.pl?gene=DSPP
ANGPTL4	Angiopoietin Like 4	Protein Coding	46	GC19P008363	11.55891	https://www.genecards.org/cgi-bin/carddisp.pl?gene=ANGPTL4
CYP24A1	Cytochrome P450 Family 24 Subfamily A Member 1	Protein Coding	48	GC20M054153	11.55513	https://www.genecards.org/cgi-bin/carddisp.pl?gene=CYP24A1
P2RX7	Purinergic Receptor P2X 7	Protein Coding	46	GC12P123575	11.55426	https://www.genecards.org/cgi-bin/carddisp.pl?gene=P2RX7
HLA-DRB5	Major Histocompatibility Complex, Class II, DR Beta 5	Protein Coding	41	GC06M046926	11.55396	https://www.genecards.org/cgi-bin/carddisp.pl?gene=HLA-DRB5

FEN1	Flap Structure-Specific Endonuclease 1	Protein Coding	47	GC11P061793	11.55333	https://www.genecards.org/cgi-bin/carddisp.pl?gene=FEN1
PTP4A1	Protein Tyrosine Phosphatase 4A1	Protein Coding	42	GC06P063521	11.54701	https://www.genecards.org/cgi-bin/carddisp.pl?gene=PTP4A1
SOX6	SRY-Box Transcription Factor 6	Protein Coding	44	GC11M015949	11.53486	https://www.genecards.org/cgi-bin/carddisp.pl?gene=SOX6
UQCRFS1	Ubiquinol-Cytochrome C Reductase, Rieske Iron-Sulfur Polypeptide 1	Protein Coding	47	GC19M029205	11.53317	https://www.genecards.org/cgi-bin/carddisp.pl?gene=UQCRFS1
ERCC5	ERCC Excision Repair 5, Endonuclease	Protein Coding	45	GC13P102845	11.52764	https://www.genecards.org/cgi-bin/carddisp.pl?gene=ERCC5
FDPS	Farnesyl Diphosphate Synthase	Protein Coding	46	GC01P155308	11.52526	https://www.genecards.org/cgi-bin/carddisp.pl?gene=FDPS
ACLY	ATP Citrate Lyase	Protein Coding	47	GC17M041866	11.51321	https://www.genecards.org/cgi-bin/carddisp.pl?gene=ACLY
USF1	Upstream Transcription Factor 1	Protein Coding	44	GC01M161039	11.50987	https://www.genecards.org/cgi-bin/carddisp.pl?gene=USF1
ITIH4	Inter-Alpha-Trypsin Inhibitor Heavy Chain 4	Protein Coding	43	GC03M052812	11.4992	https://www.genecards.org/cgi-bin/carddisp.pl?gene=ITIH4
USP18	Ubiquitin Specific Peptidase 18	Protein Coding	41	GC22P018149	11.49372	https://www.genecards.org/cgi-bin/carddisp.pl?gene=USP18
COL12A1	Collagen Type XII Alpha 1 Chain	Protein Coding	42	GC06M075084	11.49302	https://www.genecards.org/cgi-bin/carddisp.pl?gene=COL12A1
HSD11B2	Hydroxysteroid 11-Beta Dehydrogenase 2	Protein Coding	47	GC16P067433	11.4722	https://www.genecards.org/cgi-bin/carddisp.pl?gene=HSD11B2
VANGL2	VANGL Planar Cell Polarity Protein 2	Protein Coding	44	GC01P160400	11.47197	https://www.genecards.org/cgi-bin/carddisp.pl?gene=VANGL2
DCLRE1C	DNA Cross-Link Repair 1C	Protein Coding	45	GC10M014897	11.47091	https://www.genecards.org/cgi-bin/carddisp.pl?gene=DCLRE1C
UBQLN4	Ubiquilin 4	Protein Coding	40	GC01M156033	11.46719	https://www.genecards.org/cgi-bin/carddisp.pl?gene=UBQLN4
CEACAM1	CEA Cell Adhesion Molecule 1	Protein Coding	43	GC19M042507	11.46713	https://www.genecards.org/cgi-bin/carddisp.pl?gene=CEACAM1
CCL17	C-C Motif Chemokine Ligand 17	Protein Coding	38	GC16P057403	11.46223	https://www.genecards.org/cgi-bin/carddisp.pl?gene=CCL17
PRKCG	Protein Kinase C Gamma	Protein Coding	52	GC19P053879	11.45537	https://www.genecards.org/cgi-bin/carddisp.pl?gene=PRKCG

CAMP	Cathelicidin Antimicrobial Peptide	Protein Coding	42	GC03P048342	11.45532	https://www.genecards.org/cgi-bin/carddisp.pl?gene=CAMP
STING1	Stimulator Of Interferon Response CGAMP Interactor 1	Protein Coding	34	GC05M139476	11.44921	https://www.genecards.org/cgi-bin/carddisp.pl?gene=STING1
AOC3	Amine Oxidase Copper Containing 3	Protein Coding	44	GC17P042851	11.44919	https://www.genecards.org/cgi-bin/carddisp.pl?gene=AOC3
KCNE1	Potassium Voltage-Gated Channel Subfamily E Regulatory Subunit 1	Protein Coding	45	GC21M034446	11.43752	https://www.genecards.org/cgi-bin/carddisp.pl?gene=KCNE1
CDH13	Cadherin 13	Protein Coding	44	GC16P082626	11.43136	https://www.genecards.org/cgi-bin/carddisp.pl?gene=CDH13
BMP1	Bone Morphogenetic Protein 1	Protein Coding	47	GC08P022164	11.42709	https://www.genecards.org/cgi-bin/carddisp.pl?gene=BMP1
PRKCZ	Protein Kinase C Zeta	Protein Coding	49	GC01P002050	11.42618	https://www.genecards.org/cgi-bin/carddisp.pl?gene=PRKCZ
PAX3	Paired Box 3	Protein Coding	48	GC02M222199	11.42553	https://www.genecards.org/cgi-bin/carddisp.pl?gene=PAX3
TUG1	Taurine Up-Regulated 1	RNA Gene	23	GC22P030969	11.41941	https://www.genecards.org/cgi-bin/carddisp.pl?gene=TUG1
RHAG	Rh Associated Glycoprotein	Protein Coding	45	GC06M049605	11.41684	https://www.genecards.org/cgi-bin/carddisp.pl?gene=RHAG
NADK2	NAD Kinase 2, Mitochondrial	Protein Coding	38	GC05M036194	11.41531	https://www.genecards.org/cgi-bin/carddisp.pl?gene=NADK2
ARX	Aristaless Related Homeobox	Protein Coding	43	GC0XM025003	11.41329	https://www.genecards.org/cgi-bin/carddisp.pl?gene=ARX
CGA	Glycoprotein Hormones, Alpha Polypeptide	Protein Coding	43	GC06M087085	11.40474	https://www.genecards.org/cgi-bin/carddisp.pl?gene=CGA
RERE	Arginine-Glutamic Acid Dipeptide Repeats	Protein Coding	42	GC01M008364	11.40442	https://www.genecards.org/cgi-bin/carddisp.pl?gene=RERE
ENPP2	Ectonucleotide Pyrophosphatase/Phosphodiesterase 2	Protein Coding	44	GC08M119556	11.40085	https://www.genecards.org/cgi-bin/carddisp.pl?gene=ENPP2
CD9	CD9 Molecule	Protein Coding	44	GC12P011813	11.40051	https://www.genecards.org/cgi-bin/carddisp.pl?gene=CD9
ADAMTSL1	ADAMTS Like 1	Protein Coding	42	GC09P017906	11.39662	https://www.genecards.org/cgi-bin/carddisp.pl?gene=ADAMTSL1
TRIP13	Thyroid Hormone Receptor Interactor 13	Protein Coding	41	GC05P000892	11.38438	https://www.genecards.org/cgi-bin/carddisp.pl?gene=TRIP13

IQSEC2	IQ Motif And Sec7 Domain ArfGEF 2	Protein Coding	38	GC0XM053225	11.37843	https://www.genecards.org/cgi-bin/carddisp.pl?gene=IQSEC2
XRCC5	X-Ray Repair Cross Complementing 5	Protein Coding	45	GC02P216107	11.36979	https://www.genecards.org/cgi-bin/carddisp.pl?gene=XRCC5
REG3A	Regenerating Family Member 3 Alpha	Protein Coding	40	GC02M079157	11.36031	https://www.genecards.org/cgi-bin/carddisp.pl?gene=REG3A
MEGF10	Multiple EGF Like Domains 10	Protein Coding	41	GC05P127230	11.3457	https://www.genecards.org/cgi-bin/carddisp.pl?gene=MEGF10
SNCG	Synuclein Gamma	Protein Coding	42	GC10P086957	11.34427	https://www.genecards.org/cgi-bin/carddisp.pl?gene=SNCG
BLM	BLM RecQ Like Helicase	Protein Coding	50	GC15P090717	11.34175	https://www.genecards.org/cgi-bin/carddisp.pl?gene=BLM
HRH1	Histamine Receptor H1	Protein Coding	46	GC03P011113	11.3393	https://www.genecards.org/cgi-bin/carddisp.pl?gene=HRH1
MT-TS2	Mitochondrially Encoded TRNA-Ser (AGU/C) 2	RNA Gene	13	GCMTP012215	11.3355	https://www.genecards.org/cgi-bin/carddisp.pl?gene=MT-TS2
DDB2	Damage Specific DNA Binding Protein 2	Protein Coding	47	GC11P047237	11.33326	https://www.genecards.org/cgi-bin/carddisp.pl?gene=DDB2
HLA-DRA	Major Histocompatibility Complex, Class II, DR Alpha	Protein Coding	48	GC06P032439	11.33316	https://www.genecards.org/cgi-bin/carddisp.pl?gene=HLA-DRA
XPA	XPA, DNA Damage Recognition And Repair Factor	Protein Coding	47	GC09M097654	11.33177	https://www.genecards.org/cgi-bin/carddisp.pl?gene=XPA
PLA2G10	Phospholipase A2 Group X	Protein Coding	44	GC16M014672	11.32912	https://www.genecards.org/cgi-bin/carddisp.pl?gene=PLA2G10
PGF	Placental Growth Factor	Protein Coding	44	GC14M074941	11.32776	https://www.genecards.org/cgi-bin/carddisp.pl?gene=PGF
GOT1	Glutamic-Oxaloacetic Transaminase 1	Protein Coding	47	GC10M099396	11.32726	https://www.genecards.org/cgi-bin/carddisp.pl?gene=GOT1
CSNK1A1	Casein Kinase 1 Alpha 1	Protein Coding	50	GC05M149492	11.32133	https://www.genecards.org/cgi-bin/carddisp.pl?gene=CSNK1A1
PLCB4	Phospholipase C Beta 4	Protein Coding	48	GC20P009024	11.31773	https://www.genecards.org/cgi-bin/carddisp.pl?gene=PLCB4
ZADH2	Zinc Binding Alcohol Dehydrogenase Domain Containing 2	Protein Coding	38	GC18M075195	11.31729	https://www.genecards.org/cgi-bin/carddisp.pl?gene=ZADH2
NAT1	N-Acetyltransferase 1	Protein Coding	45	GC08P018179	11.31726	https://www.genecards.org/cgi-bin/carddisp.pl?gene=NAT1

HYOU1	Hypoxia Up-Regulated 1	Protein Coding	44	GC11M119045	11.30558	https://www.genecards.org/cgi-bin/carddisp.pl?gene=HYOU1
CUL4B	Cullin 4B	Protein Coding	44	GC0XM120524	11.28912	https://www.genecards.org/cgi-bin/carddisp.pl?gene=CUL4B
HPSE	Heparanase	Protein Coding	45	GC04M083292	11.28895	https://www.genecards.org/cgi-bin/carddisp.pl?gene=HPSE
DTNBP1	Dystrobrevin Binding Protein 1	Protein Coding	42	GC06M015470	11.27631	https://www.genecards.org/cgi-bin/carddisp.pl?gene=DTNBP1
ASGR1	Asialoglycoprotein Receptor 1	Protein Coding	41	GC17M007173	11.27588	https://www.genecards.org/cgi-bin/carddisp.pl?gene=ASGR1
PRPF8	Pre-MRNA Processing Factor 8	Protein Coding	42	GC17M001650	11.27551	https://www.genecards.org/cgi-bin/carddisp.pl?gene=PRPF8
NOTCH4	Notch Receptor 4	Protein Coding	45	GC06M046911	11.27038	https://www.genecards.org/cgi-bin/carddisp.pl?gene=NOTCH4
EHMT1	Euchromatic Histone Lysine Methyltransferase 1	Protein Coding	46	GC09P137618	11.25163	https://www.genecards.org/cgi-bin/carddisp.pl?gene=EHMT1
CERT1	Ceramide Transporter 1	Protein Coding	34	GC05M075356	11.2511	https://www.genecards.org/cgi-bin/carddisp.pl?gene=CERT1
NCOA3	Nuclear Receptor Coactivator 3	Protein Coding	47	GC20P047501	11.24948	https://www.genecards.org/cgi-bin/carddisp.pl?gene=NCOA3
MATN3	Matrilin 3	Protein Coding	43	GC02M019992	11.2486	https://www.genecards.org/cgi-bin/carddisp.pl?gene=MATN3
ARNTL	Aryl Hydrocarbon Receptor Nuclear Translocator Like	Protein Coding	43	GC11P013276	11.24733	https://www.genecards.org/cgi-bin/carddisp.pl?gene=ARNTL
AP1B1	Adaptor Related Protein Complex 1 Subunit Beta 1	Protein Coding	44	GC22M029327	11.24632	https://www.genecards.org/cgi-bin/carddisp.pl?gene=AP1B1
FBXW7	F-Box And WD Repeat Domain Containing 7	Protein Coding	45	GC04M152321	11.2374	https://www.genecards.org/cgi-bin/carddisp.pl?gene=FBXW7
MRPS7	Mitochondrial Ribosomal Protein S7	Protein Coding	39	GC17P075262	11.23643	https://www.genecards.org/cgi-bin/carddisp.pl?gene=MRPS7
MIP	Major Intrinsic Protein Of Lens Fiber	Protein Coding	42	GC12M056449	11.23009	https://www.genecards.org/cgi-bin/carddisp.pl?gene=MIP
PIK3R3	Phosphoinositide-3-Kinase Regulatory Subunit 3	Protein Coding	44	GC01M046041	11.22481	https://www.genecards.org/cgi-bin/carddisp.pl?gene=PIK3R3
IFT52	Intraflagellar Transport 52	Protein Coding	36	GC20P043590	11.22094	https://www.genecards.org/cgi-bin/carddisp.pl?gene=IFT52

PIK3C3	Phosphatidylinositol 3-Kinase Catalytic Subunit Type 3	Protein Coding	50	GC18P041955	11.21018	https://www.genecards.org/cgi-bin/carddisp.pl?gene=PIK3C3
ATP6V0A2	ATPase H+ Transporting V0 Subunit A2	Protein Coding	45	GC12P123712	11.20102	https://www.genecards.org/cgi-bin/carddisp.pl?gene=ATP6V0A2
FOXF1	Forkhead Box F1	Protein Coding	43	GC16P086510	11.20051	https://www.genecards.org/cgi-bin/carddisp.pl?gene=FOXF1
NUP133	Nucleoporin 133	Protein Coding	41	GC01M229441	11.19899	https://www.genecards.org/cgi-bin/carddisp.pl?gene=NUP133
SLC19A2	Solute Carrier Family 19 Member 2	Protein Coding	46	GC01M169463	11.19438	https://www.genecards.org/cgi-bin/carddisp.pl?gene=SLC19A2
GHSR	Growth Hormone Secretagogue Receptor	Protein Coding	48	GC03M172443	11.18972	https://www.genecards.org/cgi-bin/carddisp.pl?gene=GHSR
MIR95	MicroRNA 95	RNA Gene	16	GC04M008007	11.18044	https://www.genecards.org/cgi-bin/carddisp.pl?gene=MIR95
TRIM21	Tripartite Motif Containing 21	Protein Coding	43	GC11M004384	11.17753	https://www.genecards.org/cgi-bin/carddisp.pl?gene=TRIM21
SERPINB3	Serpin Family B Member 3	Protein Coding	43	GC18M063655	11.17457	https://www.genecards.org/cgi-bin/carddisp.pl?gene=SERPINB3
HSP90B1	Heat Shock Protein 90 Beta Family Member 1	Protein Coding	47	GC12P103930	11.16833	https://www.genecards.org/cgi-bin/carddisp.pl?gene=HSP90B1
LCK	LCK Proto-Oncogene, Src Family Tyrosine Kinase	Protein Coding	53	GC01P032251	11.16592	https://www.genecards.org/cgi-bin/carddisp.pl?gene=LCK
NTRK3	Neurotrophic Receptor Tyrosine Kinase 3	Protein Coding	52	GC15M087859	11.16512	https://www.genecards.org/cgi-bin/carddisp.pl?gene=NTRK3
SEMA3A	Semaphorin 3A	Protein Coding	45	GC07M083955	11.1553	https://www.genecards.org/cgi-bin/carddisp.pl?gene=SEMA3A
MAP3K7	Mitogen-Activated Protein Kinase Kinase Kinase 7	Protein Coding	51	GC06M090513	11.15236	https://www.genecards.org/cgi-bin/carddisp.pl?gene=MAP3K7
IFNAR1	Interferon Alpha And Beta Receptor Subunit 1	Protein Coding	45	GC21P033324	11.14013	https://www.genecards.org/cgi-bin/carddisp.pl?gene=IFNAR1
MIR128-1	MicroRNA 128-1	RNA Gene	19	GC02P135665	11.13795	https://www.genecards.org/cgi-bin/carddisp.pl?gene=MIR128-1
CUL3	Cullin 3	Protein Coding	47	GC02M224470	11.13085	https://www.genecards.org/cgi-bin/carddisp.pl?gene=CUL3
ELP1	Elongator Acetyltransferase Complex Subunit 1	Protein Coding	34	GC09M108868	11.12138	https://www.genecards.org/cgi-bin/carddisp.pl?gene=ELP1

SRF	Serum Response Factor	Protein Coding	42	GC06P043171	11.11334	https://www.genecards.org/cgi-bin/carddisp.pl?gene=SRF
PNMT	Phenylethanolamine N-Methyltransferase	Protein Coding	45	GC17P039667	11.10462	https://www.genecards.org/cgi-bin/carddisp.pl?gene=PNMT
RRAS2	RAS Related 2	Protein Coding	47	GC11M014299	11.10217	https://www.genecards.org/cgi-bin/carddisp.pl?gene=RRAS2
ID2	Inhibitor Of DNA Binding 2	Protein Coding	45	GC02P008678	11.08912	https://www.genecards.org/cgi-bin/carddisp.pl?gene=ID2
TFF1	Trefoil Factor 1	Protein Coding	45	GC21M042362	11.08742	https://www.genecards.org/cgi-bin/carddisp.pl?gene=TFF1
NMNAT1	Nicotinamide Nucleotide Adenylyltransferase 1	Protein Coding	49	GC01P009944	11.07916	https://www.genecards.org/cgi-bin/carddisp.pl?gene=NMNAT1
BCOR	BCL6 Corepressor	Protein Coding	43	GC0XM040049	11.07503	https://www.genecards.org/cgi-bin/carddisp.pl?gene=BCOR
SFRP4	Secreted Frizzled Related Protein 4	Protein Coding	44	GC07M037912	11.06559	https://www.genecards.org/cgi-bin/carddisp.pl?gene=SFRP4
TOP2A	DNA Topoisomerase II Alpha	Protein Coding	51	GC17M040388	11.05639	https://www.genecards.org/cgi-bin/carddisp.pl?gene=TOP2A
RYR3	Ryanodine Receptor 3	Protein Coding	42	GC15P033310	11.05561	https://www.genecards.org/cgi-bin/carddisp.pl?gene=RYR3
SLC35A2	Solute Carrier Family 35 Member A2	Protein Coding	41	GC0XM048903	11.05312	https://www.genecards.org/cgi-bin/carddisp.pl?gene=SLC35A2
LAMB1	Laminin Subunit Beta 1	Protein Coding	48	GC07M107923	11.04553	https://www.genecards.org/cgi-bin/carddisp.pl?gene=LAMB1
PACS1	Phosphofurin Acidic Cluster Sorting Protein 1	Protein Coding	41	GC11P066320	11.04384	https://www.genecards.org/cgi-bin/carddisp.pl?gene=PACS1
AIMP1	Aminoacyl TRNA Synthetase Complex Interacting Multifunctional Protein 1	Protein Coding	43	GC04P106315	11.04274	https://www.genecards.org/cgi-bin/carddisp.pl?gene=AIMP1
TRH	Thyrotropin Releasing Hormone	Protein Coding	44	GC03P129974	11.03993	https://www.genecards.org/cgi-bin/carddisp.pl?gene=TRH
MYPN	Myopalladin	Protein Coding	41	GC10P068106	11.02614	https://www.genecards.org/cgi-bin/carddisp.pl?gene=MYPN
RTN4	Reticulon 4	Protein Coding	45	GC02M054934	11.01538	https://www.genecards.org/cgi-bin/carddisp.pl?gene=RTN4
ARID2	AT-Rich Interaction Domain 2	Protein Coding	42	GC12P045729	11.01487	https://www.genecards.org/cgi-bin/carddisp.pl?gene=ARID2
FGF3	Fibroblast Growth Factor 3	Protein Coding	44	GC11M070491	11.01358	https://www.genecards.org/cgi-bin/carddisp.pl?gene=FGF3

EPHB4	EPH Receptor B4	Protein Coding	52	GC07M100803	11.01281	https://www.genecards.org/cgi-bin/carddisp.pl?gene=EPHB4
ITGA6	Integrin Subunit Alpha 6	Protein Coding	50	GC02P172427	11.00565	https://www.genecards.org/cgi-bin/carddisp.pl?gene=ITGA6
SMARCE1	SWI/SNF Related, Matrix Associated, Actin Dependent Regulator Of Chromatin, Subfamily E, Member 1	Protein Coding	46	GC17M040624	10.99075	https://www.genecards.org/cgi-bin/carddisp.pl?gene=SMARCE1
SEPTIN9	Septin 9	Protein Coding	36	GC17P077282	10.98312	https://www.genecards.org/cgi-bin/carddisp.pl?gene=SEPTIN9
SNAP25	Synaptosome Associated Protein 25	Protein Coding	51	GC20P010218	10.975	https://www.genecards.org/cgi-bin/carddisp.pl?gene=SNAP25
TLR6	Toll Like Receptor 6	Protein Coding	44	GC04M038828	10.96395	https://www.genecards.org/cgi-bin/carddisp.pl?gene=TLR6
MIR30C1	MicroRNA 30c-1	RNA Gene	22	GC01P040757	10.96354	https://www.genecards.org/cgi-bin/carddisp.pl?gene=MIR30C1
RORC	RAR Related Orphan Receptor C	Protein Coding	46	GC01M151806	10.96266	https://www.genecards.org/cgi-bin/carddisp.pl?gene=RORC
RMRP	RNA Component Of Mitochondrial RNA Processing Endoribonuclease	RNA Gene	26	GC09M035655	10.95343	https://www.genecards.org/cgi-bin/carddisp.pl?gene=RMRP
CCR3	C-C Motif Chemokine Receptor 3	Protein Coding	47	GC03P046163	10.94798	https://www.genecards.org/cgi-bin/carddisp.pl?gene=CCR3
GP6	Glycoprotein VI Platelet	Protein Coding	45	GC19M055013	10.93406	https://www.genecards.org/cgi-bin/carddisp.pl?gene=GP6
CACNA1H	Calcium Voltage-Gated Channel Subunit Alpha1 H	Protein Coding	51	GC16P001153	10.92992	https://www.genecards.org/cgi-bin/carddisp.pl?gene=CACNA1H
LONP1	Lon Peptidase 1, Mitochondrial	Protein Coding	44	GC19M005691	10.9264	https://www.genecards.org/cgi-bin/carddisp.pl?gene=LONP1
WNT7A	Wnt Family Member 7A	Protein Coding	50	GC03M016916	10.92104	https://www.genecards.org/cgi-bin/carddisp.pl?gene=WNT7A
CLPB	Caseinolytic Mitochondrial Matrix Peptidase Chaperone Subunit B	Protein Coding	44	GC11M072286	10.91575	https://www.genecards.org/cgi-bin/carddisp.pl?gene=CLPB

NSMCE2	NSE2 (MMS21) Homolog, SMC5-SMC6 Complex SUMO Ligase	Protein Coding	39	GC08P125091	10.91563	https://www.genecards.org/cgi-bin/carddisp.pl?gene=NSMCE2
COL6A3	Collagen Type VI Alpha 3 Chain	Protein Coding	44	GC02M237324	10.91505	https://www.genecards.org/cgi-bin/carddisp.pl?gene=COL6A3
ACAA2	Acetyl-CoA Acyltransferase 2	Protein Coding	44	GC18M049782	10.91199	https://www.genecards.org/cgi-bin/carddisp.pl?gene=ACAA2
SRSF2	Serine And Arginine Rich Splicing Factor 2	Protein Coding	41	GC17M076734	10.90971	https://www.genecards.org/cgi-bin/carddisp.pl?gene=SRSF2
ALG3	ALG3 Alpha-1,3-Mannosyltransferase	Protein Coding	42	GC03M184244	10.90968	https://www.genecards.org/cgi-bin/carddisp.pl?gene=ALG3
SPRTN	SprT-Like N-Terminal Domain	Protein Coding	36	GC01P231337	10.89466	https://www.genecards.org/cgi-bin/carddisp.pl?gene=SPRTN
CCR7	C-C Motif Chemokine Receptor 7	Protein Coding	45	GC17M040556	10.88799	https://www.genecards.org/cgi-bin/carddisp.pl?gene=CCR7
EXT1	Exostosin Glycosyltransferase 1	Protein Coding	50	GC08M117798	10.87894	https://www.genecards.org/cgi-bin/carddisp.pl?gene=EXT1
PRLR	Prolactin Receptor	Protein Coding	48	GC05M035048	10.87375	https://www.genecards.org/cgi-bin/carddisp.pl?gene=PRLR
GAP43	Growth Associated Protein 43	Protein Coding	44	GC03P115623	10.87299	https://www.genecards.org/cgi-bin/carddisp.pl?gene=GAP43
GPX4	Glutathione Peroxidase 4	Protein Coding	48	GC19P001103	10.87004	https://www.genecards.org/cgi-bin/carddisp.pl?gene=GPX4
KAT6A	Lysine Acetyltransferase 6A	Protein Coding	44	GC08M041929	10.86986	https://www.genecards.org/cgi-bin/carddisp.pl?gene=KAT6A
SET	SET Nuclear Proto-Oncogene	Protein Coding	47	GC09P128684	10.85428	https://www.genecards.org/cgi-bin/carddisp.pl?gene=SET
CD247	CD247 Molecule	Protein Coding	50	GC01M167399	10.84979	https://www.genecards.org/cgi-bin/carddisp.pl?gene=CD247
ATXN10	Ataxin 10	Protein Coding	43	GC22P045673	10.84245	https://www.genecards.org/cgi-bin/carddisp.pl?gene=ATXN10
UQCRQ	Ubiquinol-Cytochrome C Reductase Complex III Subunit VII	Protein Coding	42	GC05P132866	10.83769	https://www.genecards.org/cgi-bin/carddisp.pl?gene=UQCRQ
CCL18	C-C Motif Chemokine Ligand 18	Protein Coding	37	GC17P036064	10.83279	https://www.genecards.org/cgi-bin/carddisp.pl?gene=CCL18
LYRM4	LYR Motif Containing 4	Protein Coding	38	GC06M005032	10.83076	https://www.genecards.org/cgi-bin/carddisp.pl?gene=LYRM4

RAP1A	RAP1A, Member Of RAS Oncogene Family	Protein Coding	46	GC01P111542	10.82726	https://www.genecards.org/cgi-bin/carddisp.pl?gene=RAP1A
DCAF8	DDB1 And CUL4 Associated Factor 8	Protein Coding	38	GC01M160215	10.8264	https://www.genecards.org/cgi-bin/carddisp.pl?gene=DCAF8
UFD1	Ubiquitin Recognition Factor In ER Associated Degradation 1	Protein Coding	36	GC22M019450	10.82486	https://www.genecards.org/cgi-bin/carddisp.pl?gene=UFD1
JAZF1	JAZF Zinc Finger 1	Protein Coding	39	GC07M027830	10.82095	https://www.genecards.org/cgi-bin/carddisp.pl?gene=JAZF1
AMBP	Alpha-1-Microglobulin/Bikunin Precursor	Protein Coding	43	GC09M114060	10.81432	https://www.genecards.org/cgi-bin/carddisp.pl?gene=AMBP
RHOBTB2	Rho Related BTB Domain Containing 2	Protein Coding	40	GC08P022987	10.8103	https://www.genecards.org/cgi-bin/carddisp.pl?gene=RHOBTB2
RORA	RAR Related Orphan Receptor A	Protein Coding	50	GC15M060488	10.801	https://www.genecards.org/cgi-bin/carddisp.pl?gene=RORA
SCN11A	Sodium Voltage-Gated Channel Alpha Subunit 11	Protein Coding	42	GC03M038862	10.80071	https://www.genecards.org/cgi-bin/carddisp.pl?gene=SCN11A
CDX2	Caudal Type Homeobox 2	Protein Coding	44	GC13M027962	10.79582	https://www.genecards.org/cgi-bin/carddisp.pl?gene=CDX2
TNIP1	TNFAIP3 Interacting Protein 1	Protein Coding	42	GC05M151029	10.79447	https://www.genecards.org/cgi-bin/carddisp.pl?gene=TNIP1
MIR423	MicroRNA 423	RNA Gene	18	GC17P030117	10.79375	https://www.genecards.org/cgi-bin/carddisp.pl?gene=MIR423
LALBA	Lactalbumin Alpha	Protein Coding	40	GC12M048567	10.78378	https://www.genecards.org/cgi-bin/carddisp.pl?gene=LALBA
MIR30B	MicroRNA 30b	RNA Gene	20	GC08M134800	10.78068	https://www.genecards.org/cgi-bin/carddisp.pl?gene=MIR30B
PIP	Prolactin Induced Protein	Protein Coding	38	GC07P143132	10.77745	https://www.genecards.org/cgi-bin/carddisp.pl?gene=PIP
PNPO	Pyridoxamine 5'-Phosphate Oxidase	Protein Coding	46	GC17P047941	10.7726	https://www.genecards.org/cgi-bin/carddisp.pl?gene=PNPO
ITGAX	Integrin Subunit Alpha X	Protein Coding	45	GC16P032567	10.76672	https://www.genecards.org/cgi-bin/carddisp.pl?gene=ITGAX
ANK2	Ankyrin 2	Protein Coding	41	GC04P112706	10.76567	https://www.genecards.org/cgi-bin/carddisp.pl?gene=ANK2
SCAPER	S-Phase Cyclin A Associated Protein In The ER	Protein Coding	36	GC15M076347	10.7656	https://www.genecards.org/cgi-bin/carddisp.pl?gene=SCAPER

DACT1	Dishevelled Binding Antagonist Of Beta Catenin 1	Protein Coding	40	GC14P058633	10.76391	https://www.genecards.org/cgi-bin/carddisp.pl?gene=DACT1
PDGFA	Platelet Derived Growth Factor Subunit A	Protein Coding	44	GC07M000497	10.7553	https://www.genecards.org/cgi-bin/carddisp.pl?gene=PDGFA
MASP2	MBL Associated Serine Protease 2	Protein Coding	47	GC01M011026	10.75466	https://www.genecards.org/cgi-bin/carddisp.pl?gene=MASP2
ELAVL1	ELAV Like RNA Binding Protein 1	Protein Coding	43	GC19M007958	10.74929	https://www.genecards.org/cgi-bin/carddisp.pl?gene=ELAVL1
PIGR	Polymeric Immunoglobulin Receptor	Protein Coding	41	GC01M206928	10.74318	https://www.genecards.org/cgi-bin/carddisp.pl?gene=PIGR
SLC9A9	Solute Carrier Family 9 Member A9	Protein Coding	40	GC03M143265	10.74085	https://www.genecards.org/cgi-bin/carddisp.pl?gene=SLC9A9
GRIK3	Glutamate Ionotropic Receptor Kainate Type Subunit 3	Protein Coding	43	GC01M036795	10.73142	https://www.genecards.org/cgi-bin/carddisp.pl?gene=GRIK3
CPB2	Carboxypeptidase B2	Protein Coding	45	GC13M046053	10.72004	https://www.genecards.org/cgi-bin/carddisp.pl?gene=CPB2
JCAD	Junctional Cadherin 5 Associated	Protein Coding	26	GC10M030012	10.71963	https://www.genecards.org/cgi-bin/carddisp.pl?gene=JCAD
GFPT1	Glutamine--Fructose-6-Phosphate Transaminase 1	Protein Coding	47	GC02M069283	10.71841	https://www.genecards.org/cgi-bin/carddisp.pl?gene=GFPT1
PDK4	Pyruvate Dehydrogenase Kinase 4	Protein Coding	47	GC07M095583	10.70357	https://www.genecards.org/cgi-bin/carddisp.pl?gene=PDK4
ZFAT	Zinc Finger And AT-Hook Domain Containing	Protein Coding	37	GC08M134477	10.69573	https://www.genecards.org/cgi-bin/carddisp.pl?gene=ZFAT
CLRN1	Clarin 1	Protein Coding	35	GC03M150926	10.69383	https://www.genecards.org/cgi-bin/carddisp.pl?gene=CLRN1
THBS2	Thrombospondin 2	Protein Coding	46	GC06M169215	10.6914	https://www.genecards.org/cgi-bin/carddisp.pl?gene=THBS2
MBD5	Methyl-CpG Binding Domain Protein 5	Protein Coding	37	GC02P148021	10.68962	https://www.genecards.org/cgi-bin/carddisp.pl?gene=MBD5
STT3B	STT3 Oligosaccharyltransferase Complex Catalytic Subunit B	Protein Coding	44	GC03P031550	10.68569	https://www.genecards.org/cgi-bin/carddisp.pl?gene=STT3B

EPX	Eosinophil Peroxidase	Protein Coding	44	GC17P058192	10.67831	https://www.genecards.org/cgi-bin/carddisp.pl?gene=EPX
KCNA2	Potassium Voltage-Gated Channel Subfamily A Member 2	Protein Coding	46	GC01M110519	10.67739	https://www.genecards.org/cgi-bin/carddisp.pl?gene=KCNA2
STX3	Syntaxin 3	Protein Coding	43	GC11P059713	10.6753	https://www.genecards.org/cgi-bin/carddisp.pl?gene=STX3
RPL11	Ribosomal Protein L11	Protein Coding	49	GC01P023691	10.6748	https://www.genecards.org/cgi-bin/carddisp.pl?gene=RPL11
MAP1B	Microtubule Associated Protein 1B	Protein Coding	44	GC05P072107	10.67167	https://www.genecards.org/cgi-bin/carddisp.pl?gene=MAP1B
CERKL	Ceramide Kinase Like	Protein Coding	40	GC02M181536	10.66726	https://www.genecards.org/cgi-bin/carddisp.pl?gene=CERKL
LPCAT3	Lysophosphatidylcholine Acyltransferase 3	Protein Coding	36	GC12M006976	10.6657	https://www.genecards.org/cgi-bin/carddisp.pl?gene=LPCAT3
TCOF1	Treacle Ribosome Biogenesis Factor 1	Protein Coding	43	GC05P150358	10.6608	https://www.genecards.org/cgi-bin/carddisp.pl?gene=TCOF1
ROM1	Retinal Outer Segment Membrane Protein 1	Protein Coding	44	GC11P062611	10.65982	https://www.genecards.org/cgi-bin/carddisp.pl?gene=ROM1
YY1	YY1 Transcription Factor	Protein Coding	48	GC14P100238	10.65179	https://www.genecards.org/cgi-bin/carddisp.pl?gene=YY1
DHRS9	Dehydrogenase/Reductase 9	Protein Coding	40	GC02P169064	10.65149	https://www.genecards.org/cgi-bin/carddisp.pl?gene=DHRS9
MSBP1	Minisatellite Binding Protein 1	Protein Coding	5	GC00U990213	10.64589	https://www.genecards.org/cgi-bin/carddisp.pl?gene=MSBP1
SORT1	Sortilin 1	Protein Coding	44	GC01M109310	10.64058	https://www.genecards.org/cgi-bin/carddisp.pl?gene=SORT1
TRPA1	Transient Receptor Potential Cation Channel Subfamily A Member 1	Protein Coding	46	GC08M072019	10.63908	https://www.genecards.org/cgi-bin/carddisp.pl?gene=TRPA1
RPS19	Ribosomal Protein S19	Protein Coding	49	GC19P041859	10.63476	https://www.genecards.org/cgi-bin/carddisp.pl?gene=RPS19
HSPB2	Heat Shock Protein Family B (Small) Member 2	Protein Coding	41	GC11P111913	10.63035	https://www.genecards.org/cgi-bin/carddisp.pl?gene=HSPB2
GLIS3	GLIS Family Zinc Finger 3	Protein Coding	40	GC09M003816	10.62803	https://www.genecards.org/cgi-bin/carddisp.pl?gene=GLIS3
ITGA8	Integrin Subunit Alpha 8	Protein Coding	43	GC10M015513	10.62797	https://www.genecards.org/cgi-bin/carddisp.pl?gene=ITGA8

EIF2B1	Eukaryotic Translation Initiation Factor 2B Subunit Alpha	Protein Coding	44	GC12M123620	10.61639	https://www.genecards.org/cgi-bin/carddisp.pl?gene=EIF2B1
DISC1	DISC1 Scaffold Protein	Protein Coding	44	GC01P231626	10.61204	https://www.genecards.org/cgi-bin/carddisp.pl?gene=DISC1
RECQL4	RecQ Like Helicase 4	Protein Coding	43	GC08M144512	10.60959	https://www.genecards.org/cgi-bin/carddisp.pl?gene=RECQL4
CRABP1	Cellular Retinoic Acid Binding Protein 1	Protein Coding	42	GC15P078340	10.60447	https://www.genecards.org/cgi-bin/carddisp.pl?gene=CRABP1
CCND2	Cyclin D2	Protein Coding	50	GC12P011795	10.60138	https://www.genecards.org/cgi-bin/carddisp.pl?gene=CCND2
GJA5	Gap Junction Protein Alpha 5	Protein Coding	46	GC01M147756	10.59994	https://www.genecards.org/cgi-bin/carddisp.pl?gene=GJA5
CTBP1	C-Terminal Binding Protein 1	Protein Coding	48	GC04M001211	10.58987	https://www.genecards.org/cgi-bin/carddisp.pl?gene=CTBP1
PGK1	Phosphoglycerate Kinase 1	Protein Coding	49	GC0XP077944	10.58953	https://www.genecards.org/cgi-bin/carddisp.pl?gene=PGK1
TCTN3	Tectonic Family Member 3	Protein Coding	40	GC10M095663	10.5846	https://www.genecards.org/cgi-bin/carddisp.pl?gene=TCTN3
SLCO1A2	Solute Carrier Organic Anion Transporter Family Member 1A2	Protein Coding	40	GC12M021264	10.58215	https://www.genecards.org/cgi-bin/carddisp.pl?gene=SLCO1A2
B3GALNT2	Beta-1,3-N-Acetylgalactosaminyltransferase 2	Protein Coding	41	GC01M235440	10.57964	https://www.genecards.org/cgi-bin/carddisp.pl?gene=B3GALNT2
SETD5	SET Domain Containing 5	Protein Coding	38	GC03P009402	10.57862	https://www.genecards.org/cgi-bin/carddisp.pl?gene=SETD5
CD1D	CD1d Molecule	Protein Coding	43	GC01P158178	10.577	https://www.genecards.org/cgi-bin/carddisp.pl?gene=CD1D
FAR2P1	Fatty Acyl-CoA Reductase 2 Pseudogene 1	Pseudogene	13	GC02M130012	10.57271	https://www.genecards.org/cgi-bin/carddisp.pl?gene=FAR2P1
KRT10	Keratin 10	Protein Coding	43	GC17M040818	10.56855	https://www.genecards.org/cgi-bin/carddisp.pl?gene=KRT10
NNMT	Nicotinamide N-Methyltransferase	Protein Coding	46	GC11P114257	10.56772	https://www.genecards.org/cgi-bin/carddisp.pl?gene=NNMT
PLA2G1B	Phospholipase A2 Group IB	Protein Coding	47	GC12M120322	10.56747	https://www.genecards.org/cgi-bin/carddisp.pl?gene=PLA2G1B
MIR187	MicroRNA 187	RNA Gene	18	GC18M035904	10.56692	https://www.genecards.org/cgi-bin/carddisp.pl?gene=MIR187
TRAPPC9	Trafficking Protein Particle Complex Subunit 9	Protein Coding	39	GC08M139728	10.56671	https://www.genecards.org/cgi-bin/carddisp.pl?gene=TRAPPC9

LCT	Lactase	Protein Coding	43	GC02M135787	10.56483	https://www.genecards.org/cgi-bin/carddisp.pl?gene=LCT
RASA2	RAS P21 Protein Activator 2	Protein Coding	41	GC03P141487	10.56102	https://www.genecards.org/cgi-bin/carddisp.pl?gene=RASA2
SALL1	Spalt Like Transcription Factor 1	Protein Coding	45	GC16M051135	10.55348	https://www.genecards.org/cgi-bin/carddisp.pl?gene=SALL1
KRT17	Keratin 17	Protein Coding	47	GC17M041619	10.55346	https://www.genecards.org/cgi-bin/carddisp.pl?gene=KRT17
ZBTB20	Zinc Finger And BTB Domain Containing 20	Protein Coding	41	GC03M114315	10.54197	https://www.genecards.org/cgi-bin/carddisp.pl?gene=ZBTB20
OAT	Ornithine Aminotransferase	Protein Coding	48	GC10M124397	10.53657	https://www.genecards.org/cgi-bin/carddisp.pl?gene=OAT
DEFB4A	Defensin Beta 4A	Protein Coding	37	GC08P007895	10.53605	https://www.genecards.org/cgi-bin/carddisp.pl?gene=DEFB4A
DIO2	Iodothyronine Deiodinase 2	Protein Coding	41	GC14M080197	10.53469	https://www.genecards.org/cgi-bin/carddisp.pl?gene=DIO2
EYA1	EYA Transcriptional Coactivator And Phosphatase 1	Protein Coding	46	GC08M071210	10.53276	https://www.genecards.org/cgi-bin/carddisp.pl?gene=EYA1
AHSP	Alpha Hemoglobin Stabilizing Protein	Protein Coding	36	GC16P031527	10.53246	https://www.genecards.org/cgi-bin/carddisp.pl?gene=AHSP
PQBP1	Polyglutamine Binding Protein 1	Protein Coding	41	GC0XP048890	10.53089	https://www.genecards.org/cgi-bin/carddisp.pl?gene=PQBP1
PPP1R1B	Protein Phosphatase 1 Regulatory Inhibitor Subunit 1B	Protein Coding	44	GC17P039626	10.52589	https://www.genecards.org/cgi-bin/carddisp.pl?gene=PPP1R1B
FANCA	FA Complementation Group A	Protein Coding	49	GC16M089758	10.52587	https://www.genecards.org/cgi-bin/carddisp.pl?gene=FANCA
OGT	O-Linked N-Acetylglucosamine (GlcNAc) Transferase	Protein Coding	45	GC0XP071534	10.52515	https://www.genecards.org/cgi-bin/carddisp.pl?gene=OGT
CHRM1	Cholinergic Receptor Muscarinic 1	Protein Coding	47	GC11M069277	10.51278	https://www.genecards.org/cgi-bin/carddisp.pl?gene=CHRM1
ATP12A	ATPase H+/K+ Transporting Non-Gastric Alpha2 Subunit	Protein Coding	44	GC13P024680	10.51264	https://www.genecards.org/cgi-bin/carddisp.pl?gene=ATP12A

GABRA4	Gamma-Aminobutyric Acid Type A Receptor Subunit Alpha4	Protein Coding	45	GC04M046836	10.51147	https://www.genecards.org/cgi-bin/carddisp.pl?gene=GABRA4
MAP3K1	Mitogen-Activated Protein Kinase Kinase Kinase 1	Protein Coding	50	GC05P056815	10.50859	https://www.genecards.org/cgi-bin/carddisp.pl?gene=MAP3K1
THY1	Thy-1 Cell Surface Antigen	Protein Coding	43	GC11M119417	10.50677	https://www.genecards.org/cgi-bin/carddisp.pl?gene=THY1
CYGB	Cytoglobin	Protein Coding	39	GC17M076527	10.49993	https://www.genecards.org/cgi-bin/carddisp.pl?gene=CYGB
TOP1	DNA Topoisomerase I	Protein Coding	49	GC20P041028	10.49678	https://www.genecards.org/cgi-bin/carddisp.pl?gene=TOP1
SLC9A1	Solute Carrier Family 9 Member A1	Protein Coding	51	GC01M027109	10.48584	https://www.genecards.org/cgi-bin/carddisp.pl?gene=SLC9A1
RSF1	Remodeling And Spacing Factor 1	Protein Coding	36	GC11M077659	10.48472	https://www.genecards.org/cgi-bin/carddisp.pl?gene=RSF1
XPO1	Exportin 1	Protein Coding	47	GC02M061445	10.48443	https://www.genecards.org/cgi-bin/carddisp.pl?gene=XPO1
SLC39A14	Solute Carrier Family 39 Member 14	Protein Coding	44	GC08P022367	10.48276	https://www.genecards.org/cgi-bin/carddisp.pl?gene=SLC39A14
C5AR1	Complement C5a Receptor 1	Protein Coding	44	GC19P047290	10.48059	https://www.genecards.org/cgi-bin/carddisp.pl?gene=C5AR1
PROCR	Protein C Receptor	Protein Coding	43	GC20P035233	10.47434	https://www.genecards.org/cgi-bin/carddisp.pl?gene=PROCR
GSTA2	Glutathione S-Transferase Alpha 2	Protein Coding	42	GC06M052750	10.47274	https://www.genecards.org/cgi-bin/carddisp.pl?gene=GSTA2
NPR2	Natriuretic Peptide Receptor 2	Protein Coding	48	GC09P035782	10.47216	https://www.genecards.org/cgi-bin/carddisp.pl?gene=NPR2
ENTPD1	Ectonucleoside Triphosphate Diphosphohydrolase 1	Protein Coding	47	GC10P095711	10.4704	https://www.genecards.org/cgi-bin/carddisp.pl?gene=ENTPD1
DIABLO	Diablo IAP-Binding Mitochondrial Protein	Protein Coding	48	GC12M122208	10.46952	https://www.genecards.org/cgi-bin/carddisp.pl?gene=DIABLO
ACOT11	Acyl-CoA Thioesterase 11	Protein Coding	40	GC01P054542	10.46532	https://www.genecards.org/cgi-bin/carddisp.pl?gene=ACOT11
THRB	Thyroid Hormone Receptor Beta	Protein Coding	51	GC03M024117	10.46254	https://www.genecards.org/cgi-bin/carddisp.pl?gene=THRB
SEC61B	SEC61 Translocon Subunit Beta	Protein Coding	38	GC09P099222	10.4441	https://www.genecards.org/cgi-bin/carddisp.pl?gene=SEC61B

GNAO1	G Protein Subunit Alpha O1	Protein Coding	49	GC16P056231	10.44294	https://www.genecards.org/cgi-bin/carddisp.pl?gene=GNAO1
LPP	LIM Domain Containing Preferred Translocation Partner In Lipoma	Protein Coding	44	GC03P188153	10.44177	https://www.genecards.org/cgi-bin/carddisp.pl?gene=LPP
ACADSB	Acyl-CoA Dehydrogenase Short/Branched Chain	Protein Coding	47	GC10P123008	10.44067	https://www.genecards.org/cgi-bin/carddisp.pl?gene=ACADSB
SLC9A3R1	SLC9A3 Regulator 1	Protein Coding	46	GC17P074749	10.43063	https://www.genecards.org/cgi-bin/carddisp.pl?gene=SLC9A3R1
CDH3	Cadherin 3	Protein Coding	48	GC16P068637	10.41786	https://www.genecards.org/cgi-bin/carddisp.pl?gene=CDH3
SLC52A1	Solute Carrier Family 52 Member 1	Protein Coding	38	GC17M005032	10.41418	https://www.genecards.org/cgi-bin/carddisp.pl?gene=SLC52A1
ACP2	Acid Phosphatase 2, Lysosomal	Protein Coding	43	GC11M068994	10.41345	https://www.genecards.org/cgi-bin/carddisp.pl?gene=ACP2
ARSH	Arylsulfatase Family Member H	Protein Coding	34	GC0XP003006	10.41248	https://www.genecards.org/cgi-bin/carddisp.pl?gene=ARSH
NTF4	Neurotrophin 4	Protein Coding	44	GC19M049098	10.41016	https://www.genecards.org/cgi-bin/carddisp.pl?gene=NTF4
GGCX	Gamma-Glutamyl Carboxylase	Protein Coding	47	GC02M085544	10.40775	https://www.genecards.org/cgi-bin/carddisp.pl?gene=GGCX
ECM1	Extracellular Matrix Protein 1	Protein Coding	44	GC01P150508	10.40176	https://www.genecards.org/cgi-bin/carddisp.pl?gene=ECM1
SLC52A2	Solute Carrier Family 52 Member 2	Protein Coding	38	GC08P144333	10.39993	https://www.genecards.org/cgi-bin/carddisp.pl?gene=SLC52A2
SOS2	SOS Ras/Rho Guanine Nucleotide Exchange Factor 2	Protein Coding	46	GC14M050117	10.39744	https://www.genecards.org/cgi-bin/carddisp.pl?gene=SOS2
GOSR2	Golgi SNAP Receptor Complex Member 2	Protein Coding	44	GC17P046924	10.38933	https://www.genecards.org/cgi-bin/carddisp.pl?gene=GOSR2
PLD1	Phospholipase D1	Protein Coding	49	GC03M171600	10.37018	https://www.genecards.org/cgi-bin/carddisp.pl?gene=PLD1
ADORA1	Adenosine A1 Receptor	Protein Coding	48	GC01P203090	10.36107	https://www.genecards.org/cgi-bin/carddisp.pl?gene=ADORA1

ABO	ABO, Alpha 1-3-N-Acetylgalactosaminyltransferase And Alpha 1-3-Galactosyltransferase	Protein Coding	38	GC09M133250	10.35333	https://www.genecards.org/cgi-bin/carddisp.pl?gene=ABO
CTSL	Cathepsin L	Protein Coding	46	GC09P087725	10.35112	https://www.genecards.org/cgi-bin/carddisp.pl?gene=CTSL
PPP1CB	Protein Phosphatase 1 Catalytic Subunit Beta	Protein Coding	47	GC02P028752	10.34946	https://www.genecards.org/cgi-bin/carddisp.pl?gene=PPP1CB
CFL1	Cofilin 1	Protein Coding	47	GC11M065823	10.33877	https://www.genecards.org/cgi-bin/carddisp.pl?gene=CFL1
COX4I2	Cytochrome C Oxidase Subunit 4I2	Protein Coding	43	GC20P031637	10.33445	https://www.genecards.org/cgi-bin/carddisp.pl?gene=COX4I2
DVL3	Dishevelled Segment Polarity Protein 3	Protein Coding	47	GC03P184155	10.32727	https://www.genecards.org/cgi-bin/carddisp.pl?gene=DVL3
MAVS	Mitochondrial Antiviral Signaling Protein	Protein Coding	41	GC20P003850	10.31971	https://www.genecards.org/cgi-bin/carddisp.pl?gene=MAVS
FKBP5	FKBP Prolyl Isomerase 5	Protein Coding	47	GC06M047025	10.31944	https://www.genecards.org/cgi-bin/carddisp.pl?gene=FKBP5
AGRN	Agrin	Protein Coding	45	GC01P001020	10.31876	https://www.genecards.org/cgi-bin/carddisp.pl?gene=AGRN
NFIB	Nuclear Factor I B	Protein Coding	44	GC09M014077	10.31113	https://www.genecards.org/cgi-bin/carddisp.pl?gene=NFIB
ITPKC	Inositol-Trisphosphate 3-Kinase C	Protein Coding	44	GC19P041025	10.30676	https://www.genecards.org/cgi-bin/carddisp.pl?gene=ITPKC
BLZF1	Basic Leucine Zipper Nuclear Factor 1	Protein Coding	40	GC01P169367	10.30349	https://www.genecards.org/cgi-bin/carddisp.pl?gene=BLZF1
KCNK4	Potassium Two Pore Domain Channel Subfamily K Member 4	Protein Coding	42	GC11P064292	10.30004	https://www.genecards.org/cgi-bin/carddisp.pl?gene=KCNK4
FOXA1	Forkhead Box A1	Protein Coding	43	GC14M037589	10.29737	https://www.genecards.org/cgi-bin/carddisp.pl?gene=FOXA1
IRF2BP2	Interferon Regulatory Factor 2 Binding Protein 2	Protein Coding	36	GC01M234604	10.2962	https://www.genecards.org/cgi-bin/carddisp.pl?gene=IRF2BP2
HSD17B13	Hydroxysteroid 17-Beta Dehydrogenase 13	Protein Coding	37	GC04M087303	10.29436	https://www.genecards.org/cgi-bin/carddisp.pl?gene=HSD17B13
HDAC3	Histone Deacetylase 3	Protein Coding	50	GC05M141583	10.29344	https://www.genecards.org/cgi-bin/carddisp.pl?gene=HDAC3

VPS33A	VPS33A Core Subunit Of CORVET And HOPS Complexes	Protein Coding	41	GC12M122229	10.28881	https://www.genecards.org/cgi-bin/carddisp.pl?gene=VPS33A
DLST	Dihydrolipoamide S-Succinyltransferase	Protein Coding	47	GC14P074881	10.28112	https://www.genecards.org/cgi-bin/carddisp.pl?gene=DLST
ATR	ATR Serine/Threonine Kinase	Protein Coding	52	GC03M142449	10.27822	https://www.genecards.org/cgi-bin/carddisp.pl?gene=ATR
GJB6	Gap Junction Protein Beta 6	Protein Coding	45	GC13M020221	10.27575	https://www.genecards.org/cgi-bin/carddisp.pl?gene=GJB6
ABHD12	Abhydrolase Domain Containing 12, Lysophospholipase	Protein Coding	41	GC20M025294	10.27471	https://www.genecards.org/cgi-bin/carddisp.pl?gene=ABHD12
PCBD1	Pterin-4 Alpha-Carbinolamine Dehydratase 1	Protein Coding	46	GC10M070882	10.2719	https://www.genecards.org/cgi-bin/carddisp.pl?gene=PCBD1
PRKDC	Protein Kinase, DNA-Activated, Catalytic Subunit	Protein Coding	50	GC08M047773	10.27054	https://www.genecards.org/cgi-bin/carddisp.pl?gene=PRKDC
COL14A1	Collagen Type XIV Alpha 1 Chain	Protein Coding	43	GC08P120070	10.26869	https://www.genecards.org/cgi-bin/carddisp.pl?gene=COL14A1
CRABP2	Cellular Retinoic Acid Binding Protein 2	Protein Coding	44	GC01M156701	10.26667	https://www.genecards.org/cgi-bin/carddisp.pl?gene=CRABP2
EVC	EvC Ciliary Complex Subunit 1	Protein Coding	38	GC04P005712	10.26524	https://www.genecards.org/cgi-bin/carddisp.pl?gene=EVC
MIR186	MicroRNA 186	RNA Gene	18	GC01M071067	10.26039	https://www.genecards.org/cgi-bin/carddisp.pl?gene=MIR186
CTNNA3	Catenin Alpha 3	Protein Coding	40	GC10M065912	10.25986	https://www.genecards.org/cgi-bin/carddisp.pl?gene=CTNNA3
ANK1	Ankyrin 1	Protein Coding	43	GC08M041653	10.25131	https://www.genecards.org/cgi-bin/carddisp.pl?gene=ANK1
LGALS4	Galectin 4	Protein Coding	38	GC19M046731	10.24517	https://www.genecards.org/cgi-bin/carddisp.pl?gene=LGALS4
XRCC6	X-Ray Repair Cross Complementing 6	Protein Coding	47	GC22P041622	10.2426	https://www.genecards.org/cgi-bin/carddisp.pl?gene=XRCC6
NOX1	NADPH Oxidase 1	Protein Coding	42	GC0XM100843	10.22913	https://www.genecards.org/cgi-bin/carddisp.pl?gene=NOX1
DAXX	Death Domain Associated Protein	Protein Coding	44	GC06M033318	10.2246	https://www.genecards.org/cgi-bin/carddisp.pl?gene=DAXX

PGAP3	Post-GPI Attachment To Proteins Phospholipase 3	Protein Coding	38	GC17M039676	10.21666	https://www.genecards.org/cgi-bin/carddisp.pl?gene=PGAP3
CCNE1	Cyclin E1	Protein Coding	49	GC19P029811	10.21604	https://www.genecards.org/cgi-bin/carddisp.pl?gene=CCNE1
WWTR1	WW Domain Containing Transcription Regulator 1	Protein Coding	43	GC03M149517	10.20921	https://www.genecards.org/cgi-bin/carddisp.pl?gene=WWTR1
ATP4A	ATPase H+/K+ Transporting Subunit Alpha	Protein Coding	42	GC19M047080	10.2085	https://www.genecards.org/cgi-bin/carddisp.pl?gene=ATP4A
MIR130B	MicroRNA 130b	RNA Gene	17	GC22P027021	10.19444	https://www.genecards.org/cgi-bin/carddisp.pl?gene=MIR130B
GRIK2	Glutamate Ionotropic Receptor Kainate Type Subunit 2	Protein Coding	48	GC06P101181	10.18756	https://www.genecards.org/cgi-bin/carddisp.pl?gene=GRIK2
LPO	Lactoperoxidase	Protein Coding	39	GC17P058218	10.18184	https://www.genecards.org/cgi-bin/carddisp.pl?gene=LPO
PTGIS	Prostaglandin I2 Synthase	Protein Coding	48	GC20M049503	10.17269	https://www.genecards.org/cgi-bin/carddisp.pl?gene=PTGIS
PORCN	Porcupine O-Acyltransferase	Protein Coding	41	GC0XP048514	10.16844	https://www.genecards.org/cgi-bin/carddisp.pl?gene=PORCN
CD82	CD82 Molecule	Protein Coding	43	GC11P044586	10.16836	https://www.genecards.org/cgi-bin/carddisp.pl?gene=CD82
SPG21	SPG21 Abhydrolase Domain Containing, Maspardin	Protein Coding	40	GC15M064963	10.1648	https://www.genecards.org/cgi-bin/carddisp.pl?gene=SPG21
IL18RAP	Interleukin 18 Receptor Accessory Protein	Protein Coding	39	GC02P102418	10.16384	https://www.genecards.org/cgi-bin/carddisp.pl?gene=IL18RAP
COLGALT1	Collagen Beta(1-O)Galactosyltransferase 1	Protein Coding	37	GC19P017555	10.16361	https://www.genecards.org/cgi-bin/carddisp.pl?gene=COLGALT1
TMPO	Thymopoietin	Protein Coding	45	GC12P098515	10.15447	https://www.genecards.org/cgi-bin/carddisp.pl?gene=TMPO
CAST	Calpastatin	Protein Coding	46	GC05P096525	10.15332	https://www.genecards.org/cgi-bin/carddisp.pl?gene=CAST
AIF1	Allograft Inflammatory Factor 1	Protein Coding	40	GC06P055201	10.15277	https://www.genecards.org/cgi-bin/carddisp.pl?gene=AIF1
KCNC3	Potassium Voltage-Gated Channel Subfamily C Member 3	Protein Coding	44	GC19M050322	10.15228	https://www.genecards.org/cgi-bin/carddisp.pl?gene=KCNC3

NAA10	N-Alpha-Acetyltransferase 10, NatA Catalytic Subunit	Protein Coding	43	GC0XM153929	10.14086	https://www.genecards.org/cgi-bin/carddisp.pl?gene=NAA10
SOX2-OT	SOX2 Overlapping Transcript	RNA Gene	22	GC03P180989	10.12576	https://www.genecards.org/cgi-bin/carddisp.pl?gene=SOX2-OT
SLC9A6	Solute Carrier Family 9 Member A6	Protein Coding	47	GC0XP135985	10.12346	https://www.genecards.org/cgi-bin/carddisp.pl?gene=SLC9A6
HBB-LCR	Beta-Globin Locus Control Region	Biological Region	5	GC11P005270	10.12127	https://www.genecards.org/cgi-bin/carddisp.pl?gene=HBB-LCR
KCND3	Potassium Voltage-Gated Channel Subfamily D Member 3	Protein Coding	47	GC01M111770	10.12	https://www.genecards.org/cgi-bin/carddisp.pl?gene=KCND3
IVL	Involucrin	Protein Coding	38	GC01P152881	10.11668	https://www.genecards.org/cgi-bin/carddisp.pl?gene=IVL
CNBP	CCHC-Type Zinc Finger Nucleic Acid Binding Protein	Protein Coding	43	GC03M129167	10.11429	https://www.genecards.org/cgi-bin/carddisp.pl?gene=CNBP
XPC	XPC Complex Subunit, DNA Damage Recognition And Repair Factor	Protein Coding	46	GC03M016919	10.10494	https://www.genecards.org/cgi-bin/carddisp.pl?gene=XPC
POLR2A	RNA Polymerase II Subunit A	Protein Coding	45	GC17P009102	10.10481	https://www.genecards.org/cgi-bin/carddisp.pl?gene=POLR2A
ITGB4	Integrin Subunit Beta 4	Protein Coding	49	GC17P075721	10.10428	https://www.genecards.org/cgi-bin/carddisp.pl?gene=ITGB4
KCNJ6	Potassium Inwardly Rectifying Channel Subfamily J Member 6	Protein Coding	48	GC21M037607	10.10393	https://www.genecards.org/cgi-bin/carddisp.pl?gene=KCNJ6
HRG	Histidine Rich Glycoprotein	Protein Coding	43	GC03P186660	10.10212	https://www.genecards.org/cgi-bin/carddisp.pl?gene=HRG
YWHAQ	Tyrosine 3-Monooxygenase/Tryptophan 5-Monooxygenase Activation Protein Theta	Protein Coding	48	GC02M009583	10.0923	https://www.genecards.org/cgi-bin/carddisp.pl?gene=YWHAQ
ASPM	Assembly Factor For Spindle Microtubules	Protein Coding	39	GC01M197084	10.0893	https://www.genecards.org/cgi-bin/carddisp.pl?gene=ASPM

SLC25A12	Solute Carrier Family 25 Member 12	Protein Coding	47	GC02M171783	10.08722	https://www.genecards.org/cgi-bin/carddisp.pl?gene=SLC25A12
TARS1	Threonyl-TRNA Synthetase 1	Protein Coding	37	GC05P033441	10.08653	https://www.genecards.org/cgi-bin/carddisp.pl?gene=TARS1
PREPL	Prolyl Endopeptidase Like	Protein Coding	42	GC02M044281	10.08015	https://www.genecards.org/cgi-bin/carddisp.pl?gene=PREPL
MYOG	Myogenin	Protein Coding	40	GC01M203083	10.07588	https://www.genecards.org/cgi-bin/carddisp.pl?gene=MYOG
LAMC2	Laminin Subunit Gamma 2	Protein Coding	47	GC01P183186	10.0587	https://www.genecards.org/cgi-bin/carddisp.pl?gene=LAMC2
AXL	AXL Receptor Tyrosine Kinase	Protein Coding	52	GC19P041219	10.05224	https://www.genecards.org/cgi-bin/carddisp.pl?gene=AXL
MAP2K4	Mitogen-Activated Protein Kinase Kinase 4	Protein Coding	47	GC17P012020	10.04863	https://www.genecards.org/cgi-bin/carddisp.pl?gene=MAP2K4
CASQ1	Calsequestrin 1	Protein Coding	44	GC01P160190	10.04662	https://www.genecards.org/cgi-bin/carddisp.pl?gene=CASQ1
RAB7B	RAB7B, Member RAS Oncogene Family	Protein Coding	32	GC01M205976	10.04332	https://www.genecards.org/cgi-bin/carddisp.pl?gene=RAB7B
NOG	Noggin	Protein Coding	47	GC17P056593	10.04042	https://www.genecards.org/cgi-bin/carddisp.pl?gene=NOG
ACO2	Aconitase 2	Protein Coding	48	GC22P041448	10.034	https://www.genecards.org/cgi-bin/carddisp.pl?gene=ACO2
NES	Nestin	Protein Coding	40	GC01M156668	10.02803	https://www.genecards.org/cgi-bin/carddisp.pl?gene=NES
CYP4B1	Cytochrome P450 Family 4 Subfamily B Member 1	Protein Coding	44	GC01P046757	10.02289	https://www.genecards.org/cgi-bin/carddisp.pl?gene=CYP4B1
SRD5A1	Steroid 5 Alpha-Reductase 1	Protein Coding	45	GC05P006633	10.01725	https://www.genecards.org/cgi-bin/carddisp.pl?gene=SRD5A1
ADPRH	ADP-Ribosylarginine Hydrolase	Protein Coding	37	GC03P119579	10.0169	https://www.genecards.org/cgi-bin/carddisp.pl?gene=ADPRH
CDH5	Cadherin 5	Protein Coding	48	GC16P066366	10.00973	https://www.genecards.org/cgi-bin/carddisp.pl?gene=CDH5
GP9	Glycoprotein IX Platelet	Protein Coding	47	GC03P131453	10.00558	https://www.genecards.org/cgi-bin/carddisp.pl?gene=GP9
VARS1	Valyl-TRNA Synthetase 1	Protein Coding	37	GC06M046896	10.00223	https://www.genecards.org/cgi-bin/carddisp.pl?gene=VARS1
EEA1	Early Endosome Antigen 1	Protein Coding	44	GC12M092770	9.99829	https://www.genecards.org/cgi-bin/carddisp.pl?gene=EEA1
HIP1	Huntingtin Interacting Protein 1	Protein Coding	43	GC07M075533	9.997711	https://www.genecards.org/cgi-bin/carddisp.pl?gene=HIP1
GJB4	Gap Junction Protein Beta 4	Protein Coding	41	GC01P034759	9.995314	https://www.genecards.org/cgi-bin/carddisp.pl?gene=GJB4

VARS2	Valyl-TRNA Synthetase 2, Mitochondrial	Protein Coding	43	GC06P055186	9.991434	https://www.genecards.org/cgi-bin/carddisp.pl?gene=VARS2
WNT8B	Wnt Family Member 8B	Protein Coding	42	GC10P100463	9.984997	https://www.genecards.org/cgi-bin/carddisp.pl?gene=WNT8B
SGCD	Sarcoglycan Delta	Protein Coding	46	GC05P155686	9.983204	https://www.genecards.org/cgi-bin/carddisp.pl?gene=SGCD
EPHB2	EPH Receptor B2	Protein Coding	52	GC01P022710	9.982726	https://www.genecards.org/cgi-bin/carddisp.pl?gene=EPHB2
CYLD	CYLD Lysine 63 Deubiquitinase	Protein Coding	50	GC16P050742	9.977121	https://www.genecards.org/cgi-bin/carddisp.pl?gene=CYLD
RNASEL	Ribonuclease L	Protein Coding	45	GC01M182542	9.974077	https://www.genecards.org/cgi-bin/carddisp.pl?gene=RNASEL
TBX4	T-Box Transcription Factor 4	Protein Coding	42	GC17P061451	9.961285	https://www.genecards.org/cgi-bin/carddisp.pl?gene=TBX4
S100A12	S100 Calcium Binding Protein A12	Protein Coding	40	GC01M153373	9.958586	https://www.genecards.org/cgi-bin/carddisp.pl?gene=S100A12
MSX1	Msh Homeobox 1	Protein Coding	46	GC04P004861	9.957418	https://www.genecards.org/cgi-bin/carddisp.pl?gene=MSX1
CCND3	Cyclin D3	Protein Coding	48	GC06M041934	9.956216	https://www.genecards.org/cgi-bin/carddisp.pl?gene=CCND3
HBS1L	HBS1 Like Translational GTPase	Protein Coding	40	GC06M134960	9.952188	https://www.genecards.org/cgi-bin/carddisp.pl?gene=HBS1L
CES3	Carboxylesterase 3	Protein Coding	40	GC16P066963	9.941638	https://www.genecards.org/cgi-bin/carddisp.pl?gene=CES3
DEFB1	Defensin Beta 1	Protein Coding	40	GC08M006870	9.94135	https://www.genecards.org/cgi-bin/carddisp.pl?gene=DEFB1
HTR7	5-Hydroxytryptamine Receptor 7	Protein Coding	48	GC10M090740	9.931	https://www.genecards.org/cgi-bin/carddisp.pl?gene=HTR7
MLC1	Modulator Of VRAC Current 1	Protein Coding	42	GC22M050059	9.927547	https://www.genecards.org/cgi-bin/carddisp.pl?gene=MLC1
HINT1	Histidine Triad Nucleotide Binding Protein 1	Protein Coding	45	GC05M131159	9.924173	https://www.genecards.org/cgi-bin/carddisp.pl?gene=HINT1
PTS	6-Pyruvoyltetrahydropterin Synthase	Protein Coding	48	GC11P112226	9.922934	https://www.genecards.org/cgi-bin/carddisp.pl?gene=PTS
SGK1	Serum/Glucocorticoid Regulated Kinase 1	Protein Coding	50	GC06M134169	9.90464	https://www.genecards.org/cgi-bin/carddisp.pl?gene=SGK1
RBP5	Retinol Binding Protein 5	Protein Coding	37	GC12M007194	9.903232	https://www.genecards.org/cgi-bin/carddisp.pl?gene=RBP5
NLRC4	NLR Family CARD Domain Containing 4	Protein Coding	45	GC02M032224	9.898811	https://www.genecards.org/cgi-bin/carddisp.pl?gene=NLRC4

EFTUD2	Elongation Factor Tu GTP Binding Domain Containing 2	Protein Coding	43	GC17M044852	9.892878	https://www.genecards.org/cgi-bin/carddisp.pl?gene=EFTUD2
DSTYK	Dual Serine/Threonine And Tyrosine Protein Kinase	Protein Coding	42	GC01M205111	9.860373	https://www.genecards.org/cgi-bin/carddisp.pl?gene=DSTYK
HSF1	Heat Shock Transcription Factor 1	Protein Coding	45	GC08P144291	9.852005	https://www.genecards.org/cgi-bin/carddisp.pl?gene=HSF1
LAT	Linker For Activation Of T Cells	Protein Coding	47	GC16P032276	9.851159	https://www.genecards.org/cgi-bin/carddisp.pl?gene=LAT
GPD2	Glycerol-3-Phosphate Dehydrogenase 2	Protein Coding	47	GC02P156435	9.848467	https://www.genecards.org/cgi-bin/carddisp.pl?gene=GPD2
APLN	Apelin	Protein Coding	37	GC0XM129645	9.84015	https://www.genecards.org/cgi-bin/carddisp.pl?gene=APLN
MUC2	Mucin 2, Oligomeric Mucus/Gel-Forming	Protein Coding	40	GC11P001074	9.836618	https://www.genecards.org/cgi-bin/carddisp.pl?gene=MUC2
MC1R	Melanocortin 1 Receptor	Protein Coding	48	GC16P089912	9.835869	https://www.genecards.org/cgi-bin/carddisp.pl?gene=MC1R
RBBP8	RB Binding Protein 8, Endonuclease	Protein Coding	45	GC18P022798	9.835569	https://www.genecards.org/cgi-bin/carddisp.pl?gene=RBBP8
SLC26A2	Solute Carrier Family 26 Member 2	Protein Coding	45	GC05P149944	9.832773	https://www.genecards.org/cgi-bin/carddisp.pl?gene=SLC26A2
SNRNP200	Small Nuclear Ribonucleoprotein U5 Subunit 200	Protein Coding	42	GC02M096363	9.823989	https://www.genecards.org/cgi-bin/carddisp.pl?gene=SNRNP200
ALDH1A2	Aldehyde Dehydrogenase 1 Family Member A2	Protein Coding	48	GC15M067289	9.82165	https://www.genecards.org/cgi-bin/carddisp.pl?gene=ALDH1A2
YWHAE	Tyrosine 3-Monooxygenase/Tryptophan 5-Monooxygenase Activation Protein Epsilon	Protein Coding	50	GC17M001346	9.817562	https://www.genecards.org/cgi-bin/carddisp.pl?gene=YWHAE
SIX1	SIX Homeobox 1	Protein Coding	45	GC14M060643	9.815974	https://www.genecards.org/cgi-bin/carddisp.pl?gene=SIX1
TRIB1	Tribbles Pseudokinase 1	Protein Coding	38	GC08P125430	9.814937	https://www.genecards.org/cgi-bin/carddisp.pl?gene=TRIB1
MIR133A1	MicroRNA 133a-1	RNA Gene	18	GC18M021826	9.812485	https://www.genecards.org/cgi-bin/carddisp.pl?gene=MIR133A1

BMPR1B	Bone Morphogenetic Protein Receptor Type 1B	Protein Coding	51	GC04P094757	9.811538	https://www.genecards.org/cgi-bin/carddisp.pl?gene=BMPR1B
SIN3A	SIN3 Transcription Regulator Family Member A	Protein Coding	47	GC15M075369	9.811155	https://www.genecards.org/cgi-bin/carddisp.pl?gene=SIN3A
PLA2G15	Phospholipase A2 Group XV	Protein Coding	37	GC16P068245	9.802227	https://www.genecards.org/cgi-bin/carddisp.pl?gene=PLA2G15
GDF5	Growth Differentiation Factor 5	Protein Coding	47	GC20M035433	9.798262	https://www.genecards.org/cgi-bin/carddisp.pl?gene=GDF5
UCA1	Urothelial Cancer Associated 1	RNA Gene	22	GC19P015828	9.798042	https://www.genecards.org/cgi-bin/carddisp.pl?gene=UCA1
ARVCF	ARVCF Delta Catenin Family Member	Protein Coding	38	GC22M019966	9.794188	https://www.genecards.org/cgi-bin/carddisp.pl?gene=ARVCF
ACY1	Aminoacylase 1	Protein Coding	47	GC03P051983	9.782999	https://www.genecards.org/cgi-bin/carddisp.pl?gene=ACY1
KCNA1	Potassium Voltage-Gated Channel Subfamily A Member 1	Protein Coding	46	GC12P011800	9.775926	https://www.genecards.org/cgi-bin/carddisp.pl?gene=KCNA1
LACTB	Lactamase Beta	Protein Coding	36	GC15P077630	9.773698	https://www.genecards.org/cgi-bin/carddisp.pl?gene=LACTB
LAP3	Leucine Aminopeptidase 3	Protein Coding	44	GC04P017578	9.765542	https://www.genecards.org/cgi-bin/carddisp.pl?gene=LAP3
HOXA13	Homeobox A13	Protein Coding	44	GC07M027347	9.745737	https://www.genecards.org/cgi-bin/carddisp.pl?gene=HOXA13
DPYSL2	Dihydropyrimidinase Like 2	Protein Coding	46	GC08P026514	9.740933	https://www.genecards.org/cgi-bin/carddisp.pl?gene=DPYSL2
PINX1	PIN2 (TERF1) Interacting Telomerase Inhibitor 1	Protein Coding	39	GC08M010726	9.737506	https://www.genecards.org/cgi-bin/carddisp.pl?gene=PINX1
IBSP	Integrin Binding Sialoprotein	Protein Coding	38	GC04P087799	9.730112	https://www.genecards.org/cgi-bin/carddisp.pl?gene=IBSP
RRAS	RAS Related	Protein Coding	44	GC19M049635	9.728992	https://www.genecards.org/cgi-bin/carddisp.pl?gene=RRAS
LGALS1	Galectin 1	Protein Coding	44	GC22P037675	9.727539	https://www.genecards.org/cgi-bin/carddisp.pl?gene=LGALS1
CGB5	Chorionic Gonadotropin Subunit Beta 5	Protein Coding	33	GC19P049043	9.710341	https://www.genecards.org/cgi-bin/carddisp.pl?gene=CGB5
TRPS1	Transcriptional Repressor GATA Binding 1	Protein Coding	46	GC08M115408	9.708651	https://www.genecards.org/cgi-bin/carddisp.pl?gene=TRPS1

HEPN1	Hepatocellular Carcinoma, Down-Regulated 1	RNA Gene	25	GC11P124919	9.708522	https://www.genecards.org/cgi-bin/carddisp.pl?gene=HEPN1
PIN1	Peptidylprolyl Cis/Trans Isomerase, NIMA-Interacting 1	Protein Coding	48	GC19P009835	9.707603	https://www.genecards.org/cgi-bin/carddisp.pl?gene=PIN1
MLN	Motilin	Protein Coding	35	GC06M033794	9.707564	https://www.genecards.org/cgi-bin/carddisp.pl?gene=MLN
RAB5A	RAB5A, Member RAS Oncogene Family	Protein Coding	47	GC03P019963	9.707016	https://www.genecards.org/cgi-bin/carddisp.pl?gene=RAB5A
MLX	MAX Dimerization Protein MLX	Protein Coding	44	GC17P042567	9.701429	https://www.genecards.org/cgi-bin/carddisp.pl?gene=MLX
EEF2	Eukaryotic Translation Elongation Factor 2	Protein Coding	49	GC19M003976	9.701408	https://www.genecards.org/cgi-bin/carddisp.pl?gene=EEF2
NDUFC2	NADH:Ubiquinone Oxidoreductase Subunit C2	Protein Coding	41	GC11M078068	9.700644	https://www.genecards.org/cgi-bin/carddisp.pl?gene=NDUFC2
EXT2	Exostosin Glycosyltransferase 2	Protein Coding	47	GC11P044095	9.695638	https://www.genecards.org/cgi-bin/carddisp.pl?gene=EXT2
KLB	Klotho Beta	Protein Coding	40	GC04P039408	9.694351	https://www.genecards.org/cgi-bin/carddisp.pl?gene=KLB
NOS1AP	Nitric Oxide Synthase 1 Adaptor Protein	Protein Coding	39	GC01P162069	9.693832	https://www.genecards.org/cgi-bin/carddisp.pl?gene=NOS1AP
ESD	Esterase D	Protein Coding	44	GC13M046771	9.693583	https://www.genecards.org/cgi-bin/carddisp.pl?gene=ESD
TNFRSF6B	TNF Receptor Superfamily Member 6b	Protein Coding	43	GC20P063696	9.691906	https://www.genecards.org/cgi-bin/carddisp.pl?gene=TNFRSF6B
LAMB3	Laminin Subunit Beta 3	Protein Coding	46	GC01M209614	9.690313	https://www.genecards.org/cgi-bin/carddisp.pl?gene=LAMB3
IRF4	Interferon Regulatory Factor 4	Protein Coding	44	GC06P000391	9.684427	https://www.genecards.org/cgi-bin/carddisp.pl?gene=IRF4
CACNA1G	Calcium Voltage-Gated Channel Subunit Alpha1 G	Protein Coding	50	GC17P050561	9.672054	https://www.genecards.org/cgi-bin/carddisp.pl?gene=CACNA1G
PEBP1	Phosphatidylethanolamine Binding Protein 1	Protein Coding	45	GC12P118135	9.668007	https://www.genecards.org/cgi-bin/carddisp.pl?gene=PEBP1
LIPI	Lipase I	Protein Coding	37	GC21M014108	9.665094	https://www.genecards.org/cgi-bin/carddisp.pl?gene=LIPI

ITPR2	Inositol 1,4,5-Trisphosphate Receptor Type 2	Protein Coding	47	GC12M026336	9.661568	https://www.genecards.org/cgi-bin/carddisp.pl?gene=ITPR2
ROS1	ROS Proto-Oncogene 1, Receptor Tyrosine Kinase	Protein Coding	45	GC06M117287	9.660614	https://www.genecards.org/cgi-bin/carddisp.pl?gene=ROS1
CCR4	C-C Motif Chemokine Receptor 4	Protein Coding	45	GC03P032951	9.656151	https://www.genecards.org/cgi-bin/carddisp.pl?gene=CCR4
CLDN2	Claudin 2	Protein Coding	42	GC0XP106900	9.653544	https://www.genecards.org/cgi-bin/carddisp.pl?gene=CLDN2
SRPRA	SRP Receptor Subunit Alpha	Protein Coding	31	GC11M126255	9.650832	https://www.genecards.org/cgi-bin/carddisp.pl?gene=SRPRA
SLC25A22	Solute Carrier Family 25 Member 22	Protein Coding	41	GC11M001138	9.641376	https://www.genecards.org/cgi-bin/carddisp.pl?gene=SLC25A22
PSMC4	Proteasome 26S Subunit, ATPase 4	Protein Coding	41	GC19P040529	9.641056	https://www.genecards.org/cgi-bin/carddisp.pl?gene=PSMC4
GPI	Glucose-6-Phosphate Isomerase	Protein Coding	47	GC19P034359	9.637764	https://www.genecards.org/cgi-bin/carddisp.pl?gene=GPI
MIR206	MicroRNA 206	RNA Gene	20	GC06P052144	9.633758	https://www.genecards.org/cgi-bin/carddisp.pl?gene=MIR206
TBX3	T-Box Transcription Factor 3	Protein Coding	46	GC12M114670	9.630539	https://www.genecards.org/cgi-bin/carddisp.pl?gene=TBX3
MYH10	Myosin Heavy Chain 10	Protein Coding	47	GC17M008474	9.621515	https://www.genecards.org/cgi-bin/carddisp.pl?gene=MYH10
FDXR	Ferredoxin Reductase	Protein Coding	45	GC17M074862	9.619843	https://www.genecards.org/cgi-bin/carddisp.pl?gene=FDXR
CYP46A1	Cytochrome P450 Family 46 Subfamily A Member 1	Protein Coding	42	GC14P099684	9.607603	https://www.genecards.org/cgi-bin/carddisp.pl?gene=CYP46A1
MIR101-1	MicroRNA 101-1	RNA Gene	19	GC01M065058	9.602142	https://www.genecards.org/cgi-bin/carddisp.pl?gene=MIR101-1
ANO1	Anoctamin 1	Protein Coding	41	GC11P069986	9.599813	https://www.genecards.org/cgi-bin/carddisp.pl?gene=ANO1
FGF4	Fibroblast Growth Factor 4	Protein Coding	44	GC11M070438	9.595882	https://www.genecards.org/cgi-bin/carddisp.pl?gene=FGF4
KLF4	Kruppel Like Factor 4	Protein Coding	46	GC09M107484	9.595719	https://www.genecards.org/cgi-bin/carddisp.pl?gene=KLF4
CYP4F12	Cytochrome P450 Family 4 Subfamily F Member 12	Protein Coding	43	GC19P015672	9.591684	https://www.genecards.org/cgi-bin/carddisp.pl?gene=CYP4F12
CDH4	Cadherin 4	Protein Coding	43	GC20P061252	9.591014	https://www.genecards.org/cgi-bin/carddisp.pl?gene=CDH4
CYP8B1	Cytochrome P450 Family 8 Subfamily B Member 1	Protein Coding	41	GC03M042856	9.584629	https://www.genecards.org/cgi-bin/carddisp.pl?gene=CYP8B1

MIRLET7I	MicroRNA Let-7i	RNA Gene	20	GC12P062606	9.58396	https://www.genecards.org/cgi-bin/carddisp.pl?gene=MIRLET7I
PLIN5	Perilipin 5	Protein Coding	33	GC19M004522	9.575938	https://www.genecards.org/cgi-bin/carddisp.pl?gene=PLIN5
SSTR2	Somatostatin Receptor 2	Protein Coding	48	GC17P073165	9.569769	https://www.genecards.org/cgi-bin/carddisp.pl?gene=SSTR2
IL1RAPL1	Interleukin 1 Receptor Accessory Protein Like 1	Protein Coding	43	GC0XP028605	9.563024	https://www.genecards.org/cgi-bin/carddisp.pl?gene=IL1RAPL1
PCSK1	Proprotein Convertase Subtilisin/Kexin Type 1	Protein Coding	47	GC05M096391	9.562442	https://www.genecards.org/cgi-bin/carddisp.pl?gene=PCSK1
CXCL11	C-X-C Motif Chemokine Ligand 11	Protein Coding	41	GC04M076033	9.558039	https://www.genecards.org/cgi-bin/carddisp.pl?gene=CXCL11
POLR3B	RNA Polymerase III Subunit B	Protein Coding	44	GC12P106357	9.551197	https://www.genecards.org/cgi-bin/carddisp.pl?gene=POLR3B
SLC22A2	Solute Carrier Family 22 Member 2	Protein Coding	45	GC06M160173	9.550129	https://www.genecards.org/cgi-bin/carddisp.pl?gene=SLC22A2
GCLM	Glutamate-Cysteine Ligase Modifier Subunit	Protein Coding	43	GC01M093885	9.543106	https://www.genecards.org/cgi-bin/carddisp.pl?gene=GCLM
NR4A1	Nuclear Receptor Subfamily 4 Group A Member 1	Protein Coding	47	GC12P052022	9.542297	https://www.genecards.org/cgi-bin/carddisp.pl?gene=NR4A1
SACS	Sacsin Molecular Chaperone	Protein Coding	36	GC13M023328	9.54215	https://www.genecards.org/cgi-bin/carddisp.pl?gene=SACS
LCN1	Lipocalin 1	Protein Coding	40	GC09P135521	9.528162	https://www.genecards.org/cgi-bin/carddisp.pl?gene=LCN1
CD58	CD58 Molecule	Protein Coding	40	GC01M116514	9.52731	https://www.genecards.org/cgi-bin/carddisp.pl?gene=CD58
IMMT	Inner Membrane Mitochondrial Protein	Protein Coding	40	GC02M086144	9.525806	https://www.genecards.org/cgi-bin/carddisp.pl?gene=IMMT
PIBF1	Progesterone Immunomodulatory Binding Factor 1	Protein Coding	41	GC13P072782	9.525472	https://www.genecards.org/cgi-bin/carddisp.pl?gene=PIBF1
RAD21	RAD21 Cohesin Complex Component	Protein Coding	46	GC08M116846	9.523025	https://www.genecards.org/cgi-bin/carddisp.pl?gene=RAD21
CSNK2B	Casein Kinase 2 Beta	Protein Coding	50	GC06P055205	9.521455	https://www.genecards.org/cgi-bin/carddisp.pl?gene=CSNK2B
PDXK	Pyridoxal Kinase	Protein Coding	49	GC21P043719	9.514806	https://www.genecards.org/cgi-bin/carddisp.pl?gene=PDXK

CDKN2C	Cyclin Dependent Kinase Inhibitor 2C	Protein Coding	47	GC01P050960	9.511284	https://www.genecards.org/cgi-bin/carddisp.pl?gene=CDKN2C
COX7B	Cytochrome C Oxidase Subunit 7B	Protein Coding	41	GC0XP077899	9.507709	https://www.genecards.org/cgi-bin/carddisp.pl?gene=COX7B
LYN	LYN Proto-Oncogene, Src Family Tyrosine Kinase	Protein Coding	50	GC08P055879	9.483194	https://www.genecards.org/cgi-bin/carddisp.pl?gene=LYN
ZFPM2	Zinc Finger Protein, FOG Family Member 2	Protein Coding	43	GC08P104590	9.482496	https://www.genecards.org/cgi-bin/carddisp.pl?gene=ZFPM2
PRDX2	Peroxiredoxin 2	Protein Coding	47	GC19M012796	9.481212	https://www.genecards.org/cgi-bin/carddisp.pl?gene=PRDX2
TCN2	Transcobalamin 2	Protein Coding	44	GC22P030606	9.473805	https://www.genecards.org/cgi-bin/carddisp.pl?gene=TCN2
ALAS1	5'-Aminolevulinate Synthase 1	Protein Coding	45	GC03P052198	9.471748	https://www.genecards.org/cgi-bin/carddisp.pl?gene=ALAS1
ZBTB16	Zinc Finger And BTB Domain Containing 16	Protein Coding	47	GC11P114059	9.471152	https://www.genecards.org/cgi-bin/carddisp.pl?gene=ZBTB16
SKP2	S-Phase Kinase Associated Protein 2	Protein Coding	45	GC05P036151	9.466455	https://www.genecards.org/cgi-bin/carddisp.pl?gene=SKP2
GRIK1	Glutamate Ionotropic Receptor Kainate Type Subunit 1	Protein Coding	45	GC21M029536	9.466169	https://www.genecards.org/cgi-bin/carddisp.pl?gene=GRIK1
CACNA2D1	Calcium Voltage-Gated Channel Auxiliary Subunit Alpha2delta 1	Protein Coding	47	GC07M081946	9.455549	https://www.genecards.org/cgi-bin/carddisp.pl?gene=CACNA2D1
AZGP1	Alpha-2-Glycoprotein 1, Zinc-Binding	Protein Coding	44	GC07M099967	9.447481	https://www.genecards.org/cgi-bin/carddisp.pl?gene=AZGP1
ATP1A2	ATPase Na+/K+ Transporting Subunit Alpha 2	Protein Coding	48	GC01P160115	9.445	https://www.genecards.org/cgi-bin/carddisp.pl?gene=ATP1A2
PRPF3	Pre-MRNA Processing Factor 3	Protein Coding	44	GC01P150321	9.435816	https://www.genecards.org/cgi-bin/carddisp.pl?gene=PRPF3
RPL10	Ribosomal Protein L10	Protein Coding	45	GC0XP154389	9.427575	https://www.genecards.org/cgi-bin/carddisp.pl?gene=RPL10

IFI27	Interferon Alpha Inducible Protein 27	Protein Coding	38	GC14P094104	9.423713	https://www.genecards.org/cgi-bin/carddisp.pl?gene=IFI27
COG5	Component Of Oligomeric Golgi Complex 5	Protein Coding	39	GC07M107201	9.401989	https://www.genecards.org/cgi-bin/carddisp.pl?gene=COG5
PTGER4	Prostaglandin E Receptor 4	Protein Coding	47	GC05P040679	9.398602	https://www.genecards.org/cgi-bin/carddisp.pl?gene=PTGER4
CYP4A22	Cytochrome P450 Family 4 Subfamily A Member 22	Protein Coding	36	GC01P047142	9.385258	https://www.genecards.org/cgi-bin/carddisp.pl?gene=CYP4A22
HMGA2	High Mobility Group AT-Hook 2	Protein Coding	45	GC12P065824	9.385069	https://www.genecards.org/cgi-bin/carddisp.pl?gene=HMGA2
CTNND2	Catenin Delta 2	Protein Coding	43	GC05M010971	9.384844	https://www.genecards.org/cgi-bin/carddisp.pl?gene=CTNND2
NCOA1	Nuclear Receptor Coactivator 1	Protein Coding	45	GC02P024492	9.384642	https://www.genecards.org/cgi-bin/carddisp.pl?gene=NCOA1
GRK2	G Protein-Coupled Receptor Kinase 2	Protein Coding	38	GC11P067266	9.383251	https://www.genecards.org/cgi-bin/carddisp.pl?gene=GRK2
SHOC2	SHOC2 Leucine Rich Repeat Scaffold Protein	Protein Coding	42	GC10P110919	9.375777	https://www.genecards.org/cgi-bin/carddisp.pl?gene=SHOC2
CYP26A1	Cytochrome P450 Family 26 Subfamily A Member 1	Protein Coding	45	GC10P093073	9.37438	https://www.genecards.org/cgi-bin/carddisp.pl?gene=CYP26A1
HOTTIP	HOXA Distal Transcript Antisense RNA	RNA Gene	22	GC07P027198	9.369722	https://www.genecards.org/cgi-bin/carddisp.pl?gene=HOTTIP
RXRB	Retinoid X Receptor Beta	Protein Coding	48	GC06M033193	9.362427	https://www.genecards.org/cgi-bin/carddisp.pl?gene=RXRB
TBCK	TBC1 Domain Containing Kinase	Protein Coding	41	GC04M106041	9.361692	https://www.genecards.org/cgi-bin/carddisp.pl?gene=TBCK
SLC5A7	Solute Carrier Family 5 Member 7	Protein Coding	44	GC02P107969	9.35228	https://www.genecards.org/cgi-bin/carddisp.pl?gene=SLC5A7
PAK3	P21 (RAC1) Activated Kinase 3	Protein Coding	51	GC0XP110944	9.349835	https://www.genecards.org/cgi-bin/carddisp.pl?gene=PAK3
RNF113A	Ring Finger Protein 113A	Protein Coding	37	GC0XM119870	9.347163	https://www.genecards.org/cgi-bin/carddisp.pl?gene=RNF113A
MUSK	Muscle Associated Receptor Tyrosine Kinase	Protein Coding	47	GC09P110668	9.346512	https://www.genecards.org/cgi-bin/carddisp.pl?gene=MUSK
TFDP1	Transcription Factor Dp-1	Protein Coding	45	GC13P113584	9.346258	https://www.genecards.org/cgi-bin/carddisp.pl?gene=TFDP1

MIR103A1	MicroRNA 103a-1	RNA Gene	18	GC05M168560	9.344606	https://www.genecards.org/cgi-bin/carddisp.pl?gene=MIR103A1
PYGB	Glycogen Phosphorylase B	Protein Coding	45	GC20P025248	9.343521	https://www.genecards.org/cgi-bin/carddisp.pl?gene=PYGB
F2RL1	F2R Like Trypsin Receptor 1	Protein Coding	47	GC05P076818	9.332706	https://www.genecards.org/cgi-bin/carddisp.pl?gene=F2RL1
ADRA2A	Adrenoceptor Alpha 2A	Protein Coding	47	GC10P111077	9.331366	https://www.genecards.org/cgi-bin/carddisp.pl?gene=ADRA2A
FIS1	Fission, Mitochondrial 1	Protein Coding	39	GC07M101239	9.312301	https://www.genecards.org/cgi-bin/carddisp.pl?gene=FIS1
APOM	Apolipoprotein M	Protein Coding	40	GC06P055209	9.311287	https://www.genecards.org/cgi-bin/carddisp.pl?gene=APOM
SLX4	SLX4 Structure-Specific Endonuclease Subunit	Protein Coding	38	GC16M003785	9.304653	https://www.genecards.org/cgi-bin/carddisp.pl?gene=SLX4
HSD17B8	Hydroxysteroid 17-Beta Dehydrogenase 8	Protein Coding	41	GC06P055257	9.303826	https://www.genecards.org/cgi-bin/carddisp.pl?gene=HSD17B8
MYO6	Myosin VI	Protein Coding	47	GC06P075749	9.302847	https://www.genecards.org/cgi-bin/carddisp.pl?gene=MYO6
FZD5	Frizzled Class Receptor 5	Protein Coding	48	GC02M207762	9.291054	https://www.genecards.org/cgi-bin/carddisp.pl?gene=FZD5
HAND2	Heart And Neural Crest Derivatives Expressed 2	Protein Coding	45	GC04M173524	9.285707	https://www.genecards.org/cgi-bin/carddisp.pl?gene=HAND2
VPS37A	VPS37A Subunit Of ESCRT-I	Protein Coding	42	GC08P017246	9.284735	https://www.genecards.org/cgi-bin/carddisp.pl?gene=VPS37A
MOGAT2	Monoacylglycerol O-Acyltransferase 2	Protein Coding	38	GC11P075717	9.283306	https://www.genecards.org/cgi-bin/carddisp.pl?gene=MOGAT2
CXCR5	C-X-C Motif Chemokine Receptor 5	Protein Coding	42	GC11P118908	9.276999	https://www.genecards.org/cgi-bin/carddisp.pl?gene=CXCR5
SLC39A4	Solute Carrier Family 39 Member 4	Protein Coding	44	GC08M144409	9.274855	https://www.genecards.org/cgi-bin/carddisp.pl?gene=SLC39A4
MICB	MHC Class I Polypeptide-Related Sequence B	Protein Coding	42	GC06P055197	9.270586	https://www.genecards.org/cgi-bin/carddisp.pl?gene=MICB
ALDH4A1	Aldehyde Dehydrogenase 4 Family Member A1	Protein Coding	45	GC01M018871	9.266251	https://www.genecards.org/cgi-bin/carddisp.pl?gene=ALDH4A1
PTPRD	Protein Tyrosine Phosphatase Receptor Type D	Protein Coding	45	GC09M008307	9.259614	https://www.genecards.org/cgi-bin/carddisp.pl?gene=PTPRD

TSG101	Tumor Susceptibility 101	Protein Coding	43	GC11M018468	9.257687	https://www.genecards.org/cgi-bin/carddisp.pl?gene=TSG101
MAP3K20	Mitogen-Activated Protein Kinase Kinase Kinase 20	Protein Coding	40	GC02P173076	9.256441	https://www.genecards.org/cgi-bin/carddisp.pl?gene=MAP3K20
MAPK8IP1	Mitogen-Activated Protein Kinase 8 Interacting Protein 1	Protein Coding	46	GC11P046089	9.254983	https://www.genecards.org/cgi-bin/carddisp.pl?gene=MAPK8IP1
KRT13	Keratin 13	Protein Coding	44	GC17M041500	9.236635	https://www.genecards.org/cgi-bin/carddisp.pl?gene=KRT13
CACNA1B	Calcium Voltage-Gated Channel Subunit Alpha1 B	Protein Coding	50	GC09P137877	9.232675	https://www.genecards.org/cgi-bin/carddisp.pl?gene=CACNA1B
SLC8A1	Solute Carrier Family 8 Member A1	Protein Coding	45	GC02M040078	9.23237	https://www.genecards.org/cgi-bin/carddisp.pl?gene=SLC8A1
PRKAG1	Protein Kinase AMP-Activated Non-Catalytic Subunit Gamma 1	Protein Coding	49	GC12M049002	9.229853	https://www.genecards.org/cgi-bin/carddisp.pl?gene=PRKAG1
GRM7	Glutamate Metabotropic Receptor 7	Protein Coding	46	GC03P006770	9.225903	https://www.genecards.org/cgi-bin/carddisp.pl?gene=GRM7
PLN	Phospholamban	Protein Coding	45	GC06P118548	9.225259	https://www.genecards.org/cgi-bin/carddisp.pl?gene=PLN
SUMO1	Small Ubiquitin Like Modifier 1	Protein Coding	47	GC02M202206	9.220978	https://www.genecards.org/cgi-bin/carddisp.pl?gene=SUMO1
INA	Internexin Neuronal Intermediate Filament Protein Alpha	Protein Coding	40	GC10P103277	9.219435	https://www.genecards.org/cgi-bin/carddisp.pl?gene=INA
ASXL3	ASXL Transcriptional Regulator 3	Protein Coding	34	GC18P033578	9.213747	https://www.genecards.org/cgi-bin/carddisp.pl?gene=ASXL3
AGRP	Agouti Related Neuropeptide	Protein Coding	44	GC16M067482	9.213259	https://www.genecards.org/cgi-bin/carddisp.pl?gene=AGRP
CRY2	Cryptochrome Circadian Regulator 2	Protein Coding	42	GC11P046088	9.211872	https://www.genecards.org/cgi-bin/carddisp.pl?gene=CRY2
TTF2	Transcription Termination Factor 2	Protein Coding	40	GC01P117060	9.206949	https://www.genecards.org/cgi-bin/carddisp.pl?gene=TTF2
FST	Follistatin	Protein Coding	47	GC05P053480	9.196891	https://www.genecards.org/cgi-bin/carddisp.pl?gene=FST
GNAI2	G Protein Subunit Alpha I2	Protein Coding	48	GC03P050226	9.1966	https://www.genecards.org/cgi-bin/carddisp.pl?gene=GNAI2

CYP51A1	Cytochrome P450 Family 51 Subfamily A Member 1	Protein Coding	45	GC07M092112	9.190638	https://www.genecards.org/cgi-bin/carddisp.pl?gene=CYP51A1
TXNRD1	Thioredoxin Reductase 1	Protein Coding	48	GC12P104215	9.189701	https://www.genecards.org/cgi-bin/carddisp.pl?gene=TXNRD1
CASP6	Caspase 6	Protein Coding	49	GC04M109688	9.187647	https://www.genecards.org/cgi-bin/carddisp.pl?gene=CASP6
NFATC1	Nuclear Factor Of Activated T Cells 1	Protein Coding	48	GC18P079395	9.186362	https://www.genecards.org/cgi-bin/carddisp.pl?gene=NFATC1
RAPSN	Receptor Associated Protein Of The Synapse	Protein Coding	43	GC11M069002	9.18441	https://www.genecards.org/cgi-bin/carddisp.pl?gene=RAPSN
FZD2	Frizzled Class Receptor 2	Protein Coding	48	GC17P044557	9.182476	https://www.genecards.org/cgi-bin/carddisp.pl?gene=FZD2
RTN4IP1	Reticulon 4 Interacting Protein 1	Protein Coding	43	GC06M106571	9.171808	https://www.genecards.org/cgi-bin/carddisp.pl?gene=RTN4IP1
ARHGDIA	Rho GDP Dissociation Inhibitor Alpha	Protein Coding	48	GC17M081867	9.168827	https://www.genecards.org/cgi-bin/carddisp.pl?gene=ARHGDIA
ANKRD26	Ankyrin Repeat Domain 26	Protein Coding	39	GC10M026938	9.167913	https://www.genecards.org/cgi-bin/carddisp.pl?gene=ANKRD26
MIR363	MicroRNA 363	RNA Gene	15	GC0XM134205	9.164549	https://www.genecards.org/cgi-bin/carddisp.pl?gene=MIR363
GLYAT	Glycine-N-Acyltransferase	Protein Coding	42	GC11M069098	9.160475	https://www.genecards.org/cgi-bin/carddisp.pl?gene=GLYAT
LAMA3	Laminin Subunit Alpha 3	Protein Coding	45	GC18P023689	9.158698	https://www.genecards.org/cgi-bin/carddisp.pl?gene=LAMA3
MRC1	Mannose Receptor C-Type 1	Protein Coding	40	GC10P017809	9.155283	https://www.genecards.org/cgi-bin/carddisp.pl?gene=MRC1
AP2M1	Adaptor Related Protein Complex 2 Subunit Mu 1	Protein Coding	45	GC03P184174	9.151634	https://www.genecards.org/cgi-bin/carddisp.pl?gene=AP2M1
VANGL1	VANGL Planar Cell Polarity Protein 1	Protein Coding	43	GC01P115641	9.146841	https://www.genecards.org/cgi-bin/carddisp.pl?gene=VANGL1
AGPAT1	1-Acylglycerol-3-Phosphate O-Acyltransferase 1	Protein Coding	43	GC06M032168	9.145647	https://www.genecards.org/cgi-bin/carddisp.pl?gene=AGPAT1
ITGA7	Integrin Subunit Alpha 7	Protein Coding	48	GC12M055684	9.142559	https://www.genecards.org/cgi-bin/carddisp.pl?gene=ITGA7
ERG	ETS Transcription Factor ERG	Protein Coding	46	GC21M038367	9.142559	https://www.genecards.org/cgi-bin/carddisp.pl?gene=ERG

ASCC1	Activating Signal Cointegrator 1 Complex Subunit 1	Protein Coding	42	GC10M072096	9.135374	https://www.genecards.org/cgi-bin/carddisp.pl?gene=ASCC1
TYSND1	Trypsin Like Peroxisomal Matrix Peptidase 1	Protein Coding	35	GC10M070137	9.134365	https://www.genecards.org/cgi-bin/carddisp.pl?gene=TYSND1
NCOA2	Nuclear Receptor Coactivator 2	Protein Coding	45	GC08M070109	9.131163	https://www.genecards.org/cgi-bin/carddisp.pl?gene=NCOA2
TGFBR3	Transforming Growth Factor Beta Receptor 3	Protein Coding	47	GC01M091680	9.129333	https://www.genecards.org/cgi-bin/carddisp.pl?gene=TGFBR3
SLC16A1	Solute Carrier Family 16 Member 1	Protein Coding	49	GC01M112926	9.128418	https://www.genecards.org/cgi-bin/carddisp.pl?gene=SLC16A1
COMMD1	Copper Metabolism Domain Containing 1	Protein Coding	43	GC02P061888	9.111311	https://www.genecards.org/cgi-bin/carddisp.pl?gene=COMMD1
GUCA1A	Guanylate Cyclase Activator 1A	Protein Coding	43	GC06P055395	9.106963	https://www.genecards.org/cgi-bin/carddisp.pl?gene=GUCA1A
CYP11B1	Cytochrome P450 Family 11 Subfamily B Member 1	Protein Coding	50	GC08M142872	9.10541	https://www.genecards.org/cgi-bin/carddisp.pl?gene=CYP11B1
UQCRC1	Ubiquinol-Cytochrome C Reductase Core Protein 1	Protein Coding	44	GC03M048598	9.099617	https://www.genecards.org/cgi-bin/carddisp.pl?gene=UQCRC1
KDM6A	Lysine Demethylase 6A	Protein Coding	47	GC0XP044873	9.097195	https://www.genecards.org/cgi-bin/carddisp.pl?gene=KDM6A
ALCAM	Activated Leukocyte Cell Adhesion Molecule	Protein Coding	43	GC03P105366	9.088394	https://www.genecards.org/cgi-bin/carddisp.pl?gene=ALCAM
CLPX	Caseinolytic Mitochondrial Matrix Peptidase Chaperone Subunit X	Protein Coding	41	GC15M065148	9.085342	https://www.genecards.org/cgi-bin/carddisp.pl?gene=CLPX
CBR1	Carbonyl Reductase 1	Protein Coding	47	GC21P036069	9.084871	https://www.genecards.org/cgi-bin/carddisp.pl?gene=CBR1
SF3B4	Splicing Factor 3b Subunit 4	Protein Coding	44	GC01M149923	9.082061	https://www.genecards.org/cgi-bin/carddisp.pl?gene=SF3B4
LHCGR	Luteinizing Hormone/Choriogonadotropin Receptor	Protein Coding	48	GC02M048686	9.080131	https://www.genecards.org/cgi-bin/carddisp.pl?gene=LHCGR

EEF1A1	Eukaryotic Translation Elongation Factor 1 Alpha 1	Protein Coding	44	GC06M073515	9.069714	https://www.genecards.org/cgi-bin/carddisp.pl?gene=EEF1A1
NR2F2	Nuclear Receptor Subfamily 2 Group F Member 2	Protein Coding	50	GC15P096325	9.065069	https://www.genecards.org/cgi-bin/carddisp.pl?gene=NR2F2
SLC33A1	Solute Carrier Family 33 Member 1	Protein Coding	44	GC03M155821	9.061843	https://www.genecards.org/cgi-bin/carddisp.pl?gene=SLC33A1
GIP	Gastric Inhibitory Polypeptide	Protein Coding	40	GC17M048958	9.061798	https://www.genecards.org/cgi-bin/carddisp.pl?gene=GIP
RANBP2	RAN Binding Protein 2	Protein Coding	46	GC02P108719	9.060944	https://www.genecards.org/cgi-bin/carddisp.pl?gene=RANBP2
COL13A1	Collagen Type XIII Alpha 1 Chain	Protein Coding	41	GC10P069801	9.056405	https://www.genecards.org/cgi-bin/carddisp.pl?gene=COL13A1
CCL26	C-C Motif Chemokine Ligand 26	Protein Coding	38	GC07M075769	9.055018	https://www.genecards.org/cgi-bin/carddisp.pl?gene=CCL26
NANOG	Nanog Homeobox	Protein Coding	40	GC12P007787	9.053321	https://www.genecards.org/cgi-bin/carddisp.pl?gene=NANOG
MSMO1	Methylsterol Monooxygenase 1	Protein Coding	44	GC04P165327	9.04858	https://www.genecards.org/cgi-bin/carddisp.pl?gene=MSMO1
ZC4H2	Zinc Finger C4H2-Type Containing	Protein Coding	37	GC0XM064915	9.047223	https://www.genecards.org/cgi-bin/carddisp.pl?gene=ZC4H2
HNF1A-AS1	HNF1A Antisense RNA 1	RNA Gene	19	GC12M121474	9.047003	https://www.genecards.org/cgi-bin/carddisp.pl?gene=HNF1A-AS1
KIFBP	Kinesin Family Binding Protein	Protein Coding	30	GC10P068990	9.041518	https://www.genecards.org/cgi-bin/carddisp.pl?gene=KIFBP
TUFM	Tu Translation Elongation Factor, Mitochondrial	Protein Coding	45	GC16M029277	9.039992	https://www.genecards.org/cgi-bin/carddisp.pl?gene=TUFM
RACK1	Receptor For Activated C Kinase 1	Protein Coding	38	GC05M181374	9.031844	https://www.genecards.org/cgi-bin/carddisp.pl?gene=RACK1
HSPA1B	Heat Shock Protein Family A (Hsp70) Member 1B	Protein Coding	40	GC06P055218	9.030623	https://www.genecards.org/cgi-bin/carddisp.pl?gene=HSPA1B
MIR455	MicroRNA 455	RNA Gene	18	GC09P114209	9.027328	https://www.genecards.org/cgi-bin/carddisp.pl?gene=MIR455
APEH	Acylaminoacyl-Peptide Hydrolase	Protein Coding	41	GC03P049673	9.024514	https://www.genecards.org/cgi-bin/carddisp.pl?gene=APEH
TNC	Tenascin C	Protein Coding	50	GC09M115019	9.021313	https://www.genecards.org/cgi-bin/carddisp.pl?gene=TNC
DOLK	Dolichol Kinase	Protein Coding	39	GC09M128945	9.017328	https://www.genecards.org/cgi-bin/carddisp.pl?gene=DOLK

COASY	Coenzyme A Synthase	Protein Coding	46	GC17P042561	9.011848	https://www.genecards.org/cgi-bin/carddisp.pl?gene=COASY
RAD50	RAD50 Double Strand Break Repair Protein	Protein Coding	50	GC05P132556	9.00979	https://www.genecards.org/cgi-bin/carddisp.pl?gene=RAD50
RPS15	Ribosomal Protein S15	Protein Coding	41	GC19P001438	8.997669	https://www.genecards.org/cgi-bin/carddisp.pl?gene=RPS15
PI3	Peptidase Inhibitor 3	Protein Coding	40	GC20P045174	8.994905	https://www.genecards.org/cgi-bin/carddisp.pl?gene=PI3
PSMD4	Proteasome 26S Subunit Ubiquitin Receptor, Non-ATPase 4	Protein Coding	44	GC01P151256	8.979827	https://www.genecards.org/cgi-bin/carddisp.pl?gene=PSMD4
WDR62	WD Repeat Domain 62	Protein Coding	38	GC19P036054	8.971237	https://www.genecards.org/cgi-bin/carddisp.pl?gene=WDR62
FRRS1L	Ferric Chelate Reductase 1 Like	Protein Coding	34	GC09M109130	8.967488	https://www.genecards.org/cgi-bin/carddisp.pl?gene=FRRS1L
ARRB2	Arrestin Beta 2	Protein Coding	45	GC17P004711	8.957425	https://www.genecards.org/cgi-bin/carddisp.pl?gene=ARRB2
SLC3A1	Solute Carrier Family 3 Member 1	Protein Coding	48	GC02P044275	8.952633	https://www.genecards.org/cgi-bin/carddisp.pl?gene=SLC3A1
STRA6	Signaling Receptor And Transporter Of Retinol STRA6	Protein Coding	41	GC15M074179	8.944153	https://www.genecards.org/cgi-bin/carddisp.pl?gene=STRA6
NUP160	Nucleoporin 160	Protein Coding	40	GC11M069013	8.94405	https://www.genecards.org/cgi-bin/carddisp.pl?gene=NUP160
PIK3R4	Phosphoinositide-3-Kinase Regulatory Subunit 4	Protein Coding	47	GC03M130678	8.943337	https://www.genecards.org/cgi-bin/carddisp.pl?gene=PIK3R4
RPS6KA1	Ribosomal Protein S6 Kinase A1	Protein Coding	51	GC01P026540	8.932425	https://www.genecards.org/cgi-bin/carddisp.pl?gene=RPS6KA1
PER1	Period Circadian Regulator 1	Protein Coding	43	GC17M009186	8.924578	https://www.genecards.org/cgi-bin/carddisp.pl?gene=PER1
DDR2	Discoidin Domain Receptor Tyrosine Kinase 2	Protein Coding	51	GC01P162631	8.920695	https://www.genecards.org/cgi-bin/carddisp.pl?gene=DDR2
TRAP1	TNF Receptor Associated Protein 1	Protein Coding	44	GC16M003667	8.918041	https://www.genecards.org/cgi-bin/carddisp.pl?gene=TRAP1
SLC34A2	Solute Carrier Family 34 Member 2	Protein Coding	47	GC04P025657	8.91736	https://www.genecards.org/cgi-bin/carddisp.pl?gene=SLC34A2
FTCD	Formimidoyltransferase Cyclodeaminase	Protein Coding	44	GC21M048321	8.912099	https://www.genecards.org/cgi-bin/carddisp.pl?gene=FTCD
KIF11	Kinesin Family Member 11	Protein Coding	48	GC10P092574	8.90839	https://www.genecards.org/cgi-bin/carddisp.pl?gene=KIF11

APOBEC3G	Apolipoprotein B MRNA Editing Enzyme Catalytic Subunit 3G	Protein Coding	42	GC22P039078	8.903831	https://www.genecards.org/cgi-bin/carddisp.pl?gene=APOBEC3G
CTNND1	Catenin Delta 1	Protein Coding	46	GC11P057879	8.90342	https://www.genecards.org/cgi-bin/carddisp.pl?gene=CTNND1
AIMP2	Aminoacyl TRNA Synthetase Complex Interacting Multifunctional Protein 2	Protein Coding	42	GC07P006016	8.902922	https://www.genecards.org/cgi-bin/carddisp.pl?gene=AIMP2
POLB	DNA Polymerase Beta	Protein Coding	47	GC08P042338	8.895884	https://www.genecards.org/cgi-bin/carddisp.pl?gene=POLB
PPP2R5D	Protein Phosphatase 2 Regulatory Subunit B'Delta	Protein Coding	46	GC06P055406	8.895657	https://www.genecards.org/cgi-bin/carddisp.pl?gene=PPP2R5D
TFAP2B	Transcription Factor AP-2 Beta	Protein Coding	45	GC06P055469	8.890379	https://www.genecards.org/cgi-bin/carddisp.pl?gene=TFAP2B
MMADHC	Metabolism Of Cobalamin Associated D	Protein Coding	43	GC02M149569	8.887528	https://www.genecards.org/cgi-bin/carddisp.pl?gene=MMADHC
RPSA	Ribosomal Protein SA	Protein Coding	45	GC03P039406	8.886271	https://www.genecards.org/cgi-bin/carddisp.pl?gene=RPSA
CAPN2	Calpain 2	Protein Coding	48	GC01P223701	8.885628	https://www.genecards.org/cgi-bin/carddisp.pl?gene=CAPN2
SALL4	Spalt Like Transcription Factor 4	Protein Coding	44	GC20M051784	8.873285	https://www.genecards.org/cgi-bin/carddisp.pl?gene=SALL4
TNFRSF4	TNF Receptor Superfamily Member 4	Protein Coding	43	GC01M001211	8.872699	https://www.genecards.org/cgi-bin/carddisp.pl?gene=TNFRSF4
MEGF8	Multiple EGF Like Domains 8	Protein Coding	37	GC19P042325	8.867714	https://www.genecards.org/cgi-bin/carddisp.pl?gene=MEGF8
DCX	Doublecortin	Protein Coding	46	GC0XM111293	8.864035	https://www.genecards.org/cgi-bin/carddisp.pl?gene=DCX
TRIP4	Thyroid Hormone Receptor Interactor 4	Protein Coding	41	GC15P077081	8.862574	https://www.genecards.org/cgi-bin/carddisp.pl?gene=TRIP4
PDHB	Pyruvate Dehydrogenase E1 Subunit Beta	Protein Coding	47	GC03M058428	8.859496	https://www.genecards.org/cgi-bin/carddisp.pl?gene=PDHB
WDR26	WD Repeat Domain 26	Protein Coding	41	GC01M224385	8.853918	https://www.genecards.org/cgi-bin/carddisp.pl?gene=WDR26
NT5C2	5'-Nucleotidase, Cytosolic II	Protein Coding	47	GC10M103088	8.849819	https://www.genecards.org/cgi-bin/carddisp.pl?gene=NT5C2

BCL11B	BAF Chromatin Remodeling Complex Subunit BCL11B	Protein Coding	43	GC14M099169	8.849645	https://www.genecards.org/cgi-bin/carddisp.pl?gene=BCL11B
PRKACG	Protein Kinase CAMP-Activated Catalytic Subunit Gamma	Protein Coding	48	GC09M069013	8.848991	https://www.genecards.org/cgi-bin/carddisp.pl?gene=PRKACG
CHKB	Choline Kinase Beta	Protein Coding	46	GC22M050578	8.848111	https://www.genecards.org/cgi-bin/carddisp.pl?gene=CHKB
RAD51L3-RFFL	RAD51L3-RFFL Readthrough	RNA Gene	9	GC17M035009	8.848001	https://www.genecards.org/cgi-bin/carddisp.pl?gene=RAD51L3-RFFL
CSNK1D	Casein Kinase 1 Delta	Protein Coding	51	GC17M082239	8.847673	https://www.genecards.org/cgi-bin/carddisp.pl?gene=CSNK1D
TLR8	Toll Like Receptor 8	Protein Coding	48	GC0XP012924	8.847321	https://www.genecards.org/cgi-bin/carddisp.pl?gene=TLR8
RNASE1	Ribonuclease A Family Member 1, Pancreatic	Protein Coding	40	GC14M020801	8.847293	https://www.genecards.org/cgi-bin/carddisp.pl?gene=RNASE1
HDAC5	Histone Deacetylase 5	Protein Coding	48	GC17M044076	8.844287	https://www.genecards.org/cgi-bin/carddisp.pl?gene=HDAC5
SUGCT	Succinyl-CoA:Glutarate-CoA Transferase	Protein Coding	37	GC07P040134	8.842239	https://www.genecards.org/cgi-bin/carddisp.pl?gene=SUGCT
UBR1	Ubiquitin Protein Ligase E3 Component N-Recognin 1	Protein Coding	44	GC15M042942	8.840374	https://www.genecards.org/cgi-bin/carddisp.pl?gene=UBR1
HESX1	HESX Homeobox 1	Protein Coding	40	GC03M057207	8.838783	https://www.genecards.org/cgi-bin/carddisp.pl?gene=HESX1
SMCHD1	Structural Maintenance Of Chromosomes Flexible Hinge Domain Containing 1	Protein Coding	41	GC18P002649	8.837733	https://www.genecards.org/cgi-bin/carddisp.pl?gene=SMCHD1
PYCR1	Pyrroline-5-Carboxylate Reductase 1	Protein Coding	48	GC17M081932	8.836712	https://www.genecards.org/cgi-bin/carddisp.pl?gene=PYCR1
KCNH1	Potassium Voltage-Gated Channel Subfamily H Member 1	Protein Coding	47	GC01M210678	8.835409	https://www.genecards.org/cgi-bin/carddisp.pl?gene=KCNH1
FOLR1	Folate Receptor Alpha	Protein Coding	47	GC11P072190	8.835241	https://www.genecards.org/cgi-bin/carddisp.pl?gene=FOLR1
EDA	Ectodysplasin A	Protein Coding	41	GC0XP069618	8.831638	https://www.genecards.org/cgi-bin/carddisp.pl?gene=EDA

GLO1	Glyoxalase I	Protein Coding	47	GC06M047047	8.830947	https://www.genecards.org/cgi-bin/carddisp.pl?gene=GLO1
MUC4	Mucin 4, Cell Surface Associated	Protein Coding	38	GC03M195746	8.830017	https://www.genecards.org/cgi-bin/carddisp.pl?gene=MUC4
SERPINB1	Serpin Family B Member 1	Protein Coding	40	GC06M002833	8.82894	https://www.genecards.org/cgi-bin/carddisp.pl?gene=SERPINB1
VAC14	VAC14 Component Of PIKFYVE Complex	Protein Coding	43	GC16M070688	8.824135	https://www.genecards.org/cgi-bin/carddisp.pl?gene=VAC14
TDGF1	Teratocarcinoma-Derived Growth Factor 1	Protein Coding	42	GC03P046576	8.823638	https://www.genecards.org/cgi-bin/carddisp.pl?gene=TDGF1
SIRT6	Sirtuin 6	Protein Coding	47	GC19M004174	8.816259	https://www.genecards.org/cgi-bin/carddisp.pl?gene=SIRT6
HDGF	Heparin Binding Growth Factor	Protein Coding	41	GC01M156786	8.814076	https://www.genecards.org/cgi-bin/carddisp.pl?gene=HDGF
ATG7	Autophagy Related 7	Protein Coding	43	GC03P011273	8.813376	https://www.genecards.org/cgi-bin/carddisp.pl?gene=ATG7
ARNT	Aryl Hydrocarbon Receptor Nuclear Translocator	Protein Coding	44	GC01M150809	8.808784	https://www.genecards.org/cgi-bin/carddisp.pl?gene=ARNT
MCU	Mitochondrial Calcium Uniporter	Protein Coding	37	GC10P072692	8.804451	https://www.genecards.org/cgi-bin/carddisp.pl?gene=MCU
TRAF2	TNF Receptor Associated Factor 2	Protein Coding	45	GC09P136881	8.803548	https://www.genecards.org/cgi-bin/carddisp.pl?gene=TRAF2
NCAN	Neurocan	Protein Coding	41	GC19P026662	8.800243	https://www.genecards.org/cgi-bin/carddisp.pl?gene=NCAN
HLA-DMA	Major Histocompatibility Complex, Class II, DM Alpha	Protein Coding	41	GC06M046951	8.792204	https://www.genecards.org/cgi-bin/carddisp.pl?gene=HLA-DMA
TP53BP1	Tumor Protein P53 Binding Protein 1	Protein Coding	45	GC15M043403	8.791351	https://www.genecards.org/cgi-bin/carddisp.pl?gene=TP53BP1
DGKQ	Diacylglycerol Kinase Theta	Protein Coding	43	GC04M000942	8.791092	https://www.genecards.org/cgi-bin/carddisp.pl?gene=DGKQ
PLIN3	Perilipin 3	Protein Coding	41	GC19M004839	8.781133	https://www.genecards.org/cgi-bin/carddisp.pl?gene=PLIN3
TRPM7	Transient Receptor Potential Cation Channel Subfamily M Member 7	Protein Coding	46	GC15M050552	8.781031	https://www.genecards.org/cgi-bin/carddisp.pl?gene=TRPM7
NHLRC2	NHL Repeat Containing 2	Protein Coding	36	GC10P113854	8.777525	https://www.genecards.org/cgi-bin/carddisp.pl?gene=NHLRC2

SLC4A4	Solute Carrier Family 4 Member 4	Protein Coding	47	GC04P071063	8.773716	https://www.genecards.org/cgi-bin/carddisp.pl?gene=SLC4A4
SPHK1	Sphingosine Kinase 1	Protein Coding	48	GC17P076376	8.765442	https://www.genecards.org/cgi-bin/carddisp.pl?gene=SPHK1
FOSL1	FOS Like 1, AP-1 Transcription Factor Subunit	Protein Coding	45	GC11M069434	8.754175	https://www.genecards.org/cgi-bin/carddisp.pl?gene=FOSL1
PPIA	Peptidylprolyl Isomerase A	Protein Coding	48	GC07P044807	8.749454	https://www.genecards.org/cgi-bin/carddisp.pl?gene=PPIA
GATAD2B	GATA Zinc Finger Domain Containing 2B	Protein Coding	43	GC01M153805	8.748327	https://www.genecards.org/cgi-bin/carddisp.pl?gene=GATAD2B
SPECC1L	Sperm Antigen With Calponin Homology And Coiled-Coil Domains 1 Like	Protein Coding	38	GC22P026707	8.74824	https://www.genecards.org/cgi-bin/carddisp.pl?gene=SPECC1L
RAB11A	RAB11A, Member RAS Oncogene Family	Protein Coding	48	GC15P077096	8.747763	https://www.genecards.org/cgi-bin/carddisp.pl?gene=RAB11A
ADRA2B	Adrenoceptor Alpha 2B	Protein Coding	47	GC02M096112	8.740982	https://www.genecards.org/cgi-bin/carddisp.pl?gene=ADRA2B
CH25H	Cholesterol 25-Hydroxylase	Protein Coding	37	GC10M089205	8.74045	https://www.genecards.org/cgi-bin/carddisp.pl?gene=CH25H
RGN	Regucalcin	Protein Coding	40	GC0XP047189	8.73871	https://www.genecards.org/cgi-bin/carddisp.pl?gene=RGN
AZU1	Azurocidin 1	Protein Coding	40	GC19P000825	8.734966	https://www.genecards.org/cgi-bin/carddisp.pl?gene=AZU1
ZIC2	Zic Family Member 2	Protein Coding	44	GC13P099981	8.733965	https://www.genecards.org/cgi-bin/carddisp.pl?gene=ZIC2
FCGR1A	Fc Fragment Of IgG Receptor Ia	Protein Coding	42	GC01P149754	8.733392	https://www.genecards.org/cgi-bin/carddisp.pl?gene=FCGR1A
MIR502	MicroRNA 502	RNA Gene	16	GC0XP050014	8.726672	https://www.genecards.org/cgi-bin/carddisp.pl?gene=MIR502
ATG5	Autophagy Related 5	Protein Coding	44	GC06M106045	8.725743	https://www.genecards.org/cgi-bin/carddisp.pl?gene=ATG5
FUCA2	Alpha-L-Fucosidase 2	Protein Coding	41	GC06M143494	8.725465	https://www.genecards.org/cgi-bin/carddisp.pl?gene=FUCA2
NR1D1	Nuclear Receptor Subfamily 1 Group D Member 1	Protein Coding	48	GC17M040092	8.724136	https://www.genecards.org/cgi-bin/carddisp.pl?gene=NR1D1
GJA8	Gap Junction Protein Alpha 8	Protein Coding	45	GC01P147902	8.71728	https://www.genecards.org/cgi-bin/carddisp.pl?gene=GJA8
DHODH	Dihydroorotate Dehydrogenase (Quinone)	Protein Coding	46	GC16P072008	8.713929	https://www.genecards.org/cgi-bin/carddisp.pl?gene=DHODH
DPEP1	Dipeptidase 1	Protein Coding	43	GC16P089613	8.691988	https://www.genecards.org/cgi-bin/carddisp.pl?gene=DPEP1

VAMP2	Vesicle Associated Membrane Protein 2	Protein Coding	44	GC17M009187	8.682579	https://www.genecards.org/cgi-bin/carddisp.pl?gene=VAMP2
TOP3A	DNA Topoisomerase III Alpha	Protein Coding	42	GC17M018271	8.667412	https://www.genecards.org/cgi-bin/carddisp.pl?gene=TOP3A
NPAS2	Neuronal PAS Domain Protein 2	Protein Coding	42	GC02P100820	8.664587	https://www.genecards.org/cgi-bin/carddisp.pl?gene=NPAS2
ERLIN2	ER Lipid Raft Associated 2	Protein Coding	41	GC08P037736	8.662991	https://www.genecards.org/cgi-bin/carddisp.pl?gene=ERLIN2
HPS1	HPS1 Biogenesis Of Lysosomal Organelles Complex 3 Subunit 1	Protein Coding	43	GC10M098416	8.660741	https://www.genecards.org/cgi-bin/carddisp.pl?gene=HPS1
RGR	Retinal G Protein Coupled Receptor	Protein Coding	43	GC10P085402	8.643101	https://www.genecards.org/cgi-bin/carddisp.pl?gene=RGR
PPIB	Peptidylprolyl Isomerase B	Protein Coding	50	GC15M064155	8.639938	https://www.genecards.org/cgi-bin/carddisp.pl?gene=PPIB
APLP2	Amyloid Beta Precursor Like Protein 2	Protein Coding	43	GC11P130069	8.637795	https://www.genecards.org/cgi-bin/carddisp.pl?gene=APLP2
SNX14	Sorting Nexin 14	Protein Coding	39	GC06M085505	8.632335	https://www.genecards.org/cgi-bin/carddisp.pl?gene=SNX14
MSN	Moesin	Protein Coding	48	GC0XP065588	8.63151	https://www.genecards.org/cgi-bin/carddisp.pl?gene=MSN
JUNB	JunB Proto-Oncogene, AP-1 Transcription Factor Subunit	Protein Coding	43	GC19P012791	8.629303	https://www.genecards.org/cgi-bin/carddisp.pl?gene=JUNB
ARAF	A-Raf Proto-Oncogene, Serine/Threonine Kinase	Protein Coding	47	GC0XP047562	8.626293	https://www.genecards.org/cgi-bin/carddisp.pl?gene=ARAF
SGCG	Sarcoglycan Gamma	Protein Coding	43	GC13P023160	8.621014	https://www.genecards.org/cgi-bin/carddisp.pl?gene=SGCG
WNT10B	Wnt Family Member 10B	Protein Coding	48	GC12M048965	8.610108	https://www.genecards.org/cgi-bin/carddisp.pl?gene=WNT10B
PSMA7	Proteasome 20S Subunit Alpha 7	Protein Coding	45	GC20M062136	8.6087	https://www.genecards.org/cgi-bin/carddisp.pl?gene=PSMA7
ALOX15B	Arachidonate 15-Lipoxygenase Type B	Protein Coding	43	GC17P008039	8.607839	https://www.genecards.org/cgi-bin/carddisp.pl?gene=ALOX15B
GPNMB	Glycoprotein Nmb	Protein Coding	44	GC07P023238	8.607162	https://www.genecards.org/cgi-bin/carddisp.pl?gene=GPNMB
ELK1	ETS Transcription Factor ELK1	Protein Coding	44	GC0XM047635	8.606547	https://www.genecards.org/cgi-bin/carddisp.pl?gene=ELK1

PDSS2	Decaprenyl Diphosphate Synthase Subunit 2	Protein Coding	41	GC06M107152	8.598368	https://www.genecards.org/cgi-bin/carddisp.pl?gene=PDSS2
SRA1	Steroid Receptor RNA Activator 1	Protein Coding	38	GC05M140537	8.594357	https://www.genecards.org/cgi-bin/carddisp.pl?gene=SRA1
PADI4	Peptidyl Arginine Deiminase 4	Protein Coding	44	GC01P017308	8.591413	https://www.genecards.org/cgi-bin/carddisp.pl?gene=PADI4
CPLX1	Complexin 1	Protein Coding	43	GC04M000784	8.591339	https://www.genecards.org/cgi-bin/carddisp.pl?gene=CPLX1
CSK	C-Terminal Src Kinase	Protein Coding	48	GC15P074782	8.589247	https://www.genecards.org/cgi-bin/carddisp.pl?gene=CSK
PAPPA	Pappalysin 1	Protein Coding	41	GC09P116231	8.587589	https://www.genecards.org/cgi-bin/carddisp.pl?gene=PAPPA
UGT8	UDP Glycosyltransferase 8	Protein Coding	44	GC04P114598	8.586639	https://www.genecards.org/cgi-bin/carddisp.pl?gene=UGT8
NRBF2	Nuclear Receptor Binding Factor 2	Protein Coding	39	GC10P063133	8.582347	https://www.genecards.org/cgi-bin/carddisp.pl?gene=NRBF2
SLC1A1	Solute Carrier Family 1 Member 1	Protein Coding	48	GC09P004490	8.58135	https://www.genecards.org/cgi-bin/carddisp.pl?gene=SLC1A1
CFD	Complement Factor D	Protein Coding	45	GC19P000859	8.577744	https://www.genecards.org/cgi-bin/carddisp.pl?gene=CFD
NARS1	Asparaginyl-TRNA Synthetase 1	Protein Coding	36	GC18M057601	8.577522	https://www.genecards.org/cgi-bin/carddisp.pl?gene=NARS1
LSR	Lipolysis Stimulated Lipoprotein Receptor	Protein Coding	41	GC19P040343	8.573154	https://www.genecards.org/cgi-bin/carddisp.pl?gene=LSR
ERFE	Erythroferrone	Protein Coding	24	GC02P238159	8.57283	https://www.genecards.org/cgi-bin/carddisp.pl?gene=ERFE
CD5	CD5 Molecule	Protein Coding	41	GC11P061114	8.570673	https://www.genecards.org/cgi-bin/carddisp.pl?gene=CD5
SULT1E1	Sulfotransferase Family 1E Member 1	Protein Coding	45	GC04M069841	8.568748	https://www.genecards.org/cgi-bin/carddisp.pl?gene=SULT1E1
NAB2	NGFI-A Binding Protein 2	Protein Coding	41	GC12P057088	8.567242	https://www.genecards.org/cgi-bin/carddisp.pl?gene=NAB2
TBL1X	Transducin Beta Like 1 X-Linked	Protein Coding	38	GC0XP009463	8.562752	https://www.genecards.org/cgi-bin/carddisp.pl?gene=TBL1X
AQP9	Aquaporin 9	Protein Coding	44	GC15P058138	8.561291	https://www.genecards.org/cgi-bin/carddisp.pl?gene=AQP9
RHBDF2	Rhomboid 5 Homolog 2	Protein Coding	41	GC17M076470	8.559841	https://www.genecards.org/cgi-bin/carddisp.pl?gene=RHBDF2
NEFM	Neurofilament Medium Chain	Protein Coding	43	GC08P024913	8.559085	https://www.genecards.org/cgi-bin/carddisp.pl?gene=NEFM
IGES	Immunoglobulin E Concentration, Serum	Genetic Locus	4	GC05U990033	8.558283	https://www.genecards.org/cgi-bin/carddisp.pl?gene=IGES

MIR490	MicroRNA 490	RNA Gene	16	GC07P136903	8.544781	https://www.genecards.org/cgi-bin/carddisp.pl?gene=MIR490
MIR129-1	MicroRNA 129-1	RNA Gene	18	GC07P128207	8.542717	https://www.genecards.org/cgi-bin/carddisp.pl?gene=MIR129-1
S100A4	S100 Calcium Binding Protein A4	Protein Coding	44	GC01M153543	8.542686	https://www.genecards.org/cgi-bin/carddisp.pl?gene=S100A4
UGDH	UDP-Glucose 6-Dehydrogenase	Protein Coding	47	GC04M039502	8.533478	https://www.genecards.org/cgi-bin/carddisp.pl?gene=UGDH
CRY1	Cryptochrome Circadian Regulator 1	Protein Coding	44	GC12M106991	8.533283	https://www.genecards.org/cgi-bin/carddisp.pl?gene=CRY1
RHOC	Ras Homolog Family Member C	Protein Coding	41	GC01M112701	8.522123	https://www.genecards.org/cgi-bin/carddisp.pl?gene=RHOC
CCL7	C-C Motif Chemokine Ligand 7	Protein Coding	42	GC17P034270	8.517108	https://www.genecards.org/cgi-bin/carddisp.pl?gene=CCL7
KAT5	Lysine Acetyltransferase 5	Protein Coding	47	GC11P065711	8.496277	https://www.genecards.org/cgi-bin/carddisp.pl?gene=KAT5
SGCA	Sarcoglycan Alpha	Protein Coding	43	GC17P050164	8.490235	https://www.genecards.org/cgi-bin/carddisp.pl?gene=SGCA
ESRRB	Estrogen Related Receptor Beta	Protein Coding	50	GC14P076310	8.489886	https://www.genecards.org/cgi-bin/carddisp.pl?gene=ESRRB
PDE3B	Phosphodiesterase 3B	Protein Coding	44	GC11P014643	8.488647	https://www.genecards.org/cgi-bin/carddisp.pl?gene=PDE3B
TNRC6A	Trinucleotide Repeat Containing Adaptor 6A	Protein Coding	40	GC16P024611	8.47611	https://www.genecards.org/cgi-bin/carddisp.pl?gene=TNRC6A
CSN2	Casein Beta	Protein Coding	37	GC04M069955	8.473336	https://www.genecards.org/cgi-bin/carddisp.pl?gene=CSN2
MYO1E	Myosin IE	Protein Coding	44	GC15M059132	8.47229	https://www.genecards.org/cgi-bin/carddisp.pl?gene=MYO1E
WDR81	WD Repeat Domain 81	Protein Coding	37	GC17P001716	8.471716	https://www.genecards.org/cgi-bin/carddisp.pl?gene=WDR81
RPL26	Ribosomal Protein L26	Protein Coding	43	GC17M008377	8.470219	https://www.genecards.org/cgi-bin/carddisp.pl?gene=RPL26
DUSP1	Dual Specificity Phosphatase 1	Protein Coding	48	GC05M172768	8.468688	https://www.genecards.org/cgi-bin/carddisp.pl?gene=DUSP1
LEMD3	LEM Domain Containing 3	Protein Coding	41	GC12P065169	8.465513	https://www.genecards.org/cgi-bin/carddisp.pl?gene=LEMD3
L2HGDH	L-2-Hydroxyglutarate Dehydrogenase	Protein Coding	41	GC14M050237	8.464959	https://www.genecards.org/cgi-bin/carddisp.pl?gene=L2HGDH
DCXR	Dicarbonyl And L-Xylulose Reductase	Protein Coding	45	GC17M082036	8.46411	https://www.genecards.org/cgi-bin/carddisp.pl?gene=DCXR
PDSS1	Decaprenyl Diphosphate Synthase Subunit 1	Protein Coding	43	GC10P026697	8.464005	https://www.genecards.org/cgi-bin/carddisp.pl?gene=PDSS1

MFF	Mitochondrial Fission Factor	Protein Coding	38	GC02P227325	8.461156	https://www.genecards.org/cgi-bin/carddisp.pl?gene=MFF
ACER3	Alkaline Ceramidase 3	Protein Coding	39	GC11P076860	8.453665	https://www.genecards.org/cgi-bin/carddisp.pl?gene=ACER3
KYNU	Kynureninase	Protein Coding	47	GC02P142877	8.449566	https://www.genecards.org/cgi-bin/carddisp.pl?gene=KYNU
REL	REL Proto-Oncogene, NF-KB Subunit	Protein Coding	47	GC02P060881	8.447259	https://www.genecards.org/cgi-bin/carddisp.pl?gene=REL
PARVB	Parvin Beta	Protein Coding	40	GC22P043999	8.441185	https://www.genecards.org/cgi-bin/carddisp.pl?gene=PARVB
ROBO1	Roundabout Guidance Receptor 1	Protein Coding	45	GC03M078597	8.439934	https://www.genecards.org/cgi-bin/carddisp.pl?gene=ROBO1
DEAF1	DEAF1 Transcription Factor	Protein Coding	40	GC11M000644	8.436068	https://www.genecards.org/cgi-bin/carddisp.pl?gene=DEAF1
PIKFYVE	Phosphoinositide Kinase, FYVE-Type Zinc Finger Containing	Protein Coding	48	GC02P208266	8.434423	https://www.genecards.org/cgi-bin/carddisp.pl?gene=PIKFYVE
DDB1	Damage Specific DNA Binding Protein 1	Protein Coding	43	GC11M069196	8.432693	https://www.genecards.org/cgi-bin/carddisp.pl?gene=DDB1
GPIHBP1	Glycosylphosphatidylinositol Anchored High Density Lipoprotein Binding Protein 1	Protein Coding	35	GC08P143213	8.431719	https://www.genecards.org/cgi-bin/carddisp.pl?gene=GPIHBP1
HAP1	Huntingtin Associated Protein 1	Protein Coding	40	GC17M041717	8.429021	https://www.genecards.org/cgi-bin/carddisp.pl?gene=HAP1
KISS1	KiSS-1 Metastasis Suppressor	Protein Coding	42	GC01M204190	8.422831	https://www.genecards.org/cgi-bin/carddisp.pl?gene=KISS1
ARID5B	AT-Rich Interaction Domain 5B	Protein Coding	41	GC10P061901	8.419536	https://www.genecards.org/cgi-bin/carddisp.pl?gene=ARID5B
CHRND	Cholinergic Receptor Nicotinic Delta Subunit	Protein Coding	43	GC02P232525	8.418051	https://www.genecards.org/cgi-bin/carddisp.pl?gene=CHRND
LTA4H	Leukotriene A4 Hydrolase	Protein Coding	47	GC12M096000	8.418037	https://www.genecards.org/cgi-bin/carddisp.pl?gene=LTA4H
UGT2B17	UDP Glucuronosyltransferase Family 2 Member B17	Protein Coding	41	GC04M068537	8.417027	https://www.genecards.org/cgi-bin/carddisp.pl?gene=UGT2B17
POLR1D	RNA Polymerase I And III Subunit D	Protein Coding	45	GC13P027620	8.416927	https://www.genecards.org/cgi-bin/carddisp.pl?gene=POLR1D

FSCN1	Fascin Actin-Bundling Protein 1	Protein Coding	44	GC07P005592	8.416878	https://www.genecards.org/cgi-bin/carddisp.pl?gene=FSCN1
TUBB2A	Tubulin Beta 2A Class IIa	Protein Coding	46	GC06M003153	8.416239	https://www.genecards.org/cgi-bin/carddisp.pl?gene=TUBB2A
AZIN1	Antizyme Inhibitor 1	Protein Coding	40	GC08M102826	8.412376	https://www.genecards.org/cgi-bin/carddisp.pl?gene=AZIN1
GNAI1	G Protein Subunit Alpha I1	Protein Coding	48	GC07P007969	8.41148	https://www.genecards.org/cgi-bin/carddisp.pl?gene=GNAI1
RPS17	Ribosomal Protein S17	Protein Coding	43	GC15M082536	8.403085	https://www.genecards.org/cgi-bin/carddisp.pl?gene=RPS17
MARK3	Microtubule Affinity Regulating Kinase 3	Protein Coding	49	GC14P103385	8.402725	https://www.genecards.org/cgi-bin/carddisp.pl?gene=MARK3
CCAT1	Colon Cancer Associated Transcript 1	RNA Gene	14	GC08M127207	8.400127	https://www.genecards.org/cgi-bin/carddisp.pl?gene=CCAT1
TRDN	Triadin	Protein Coding	41	GC06M123198	8.396246	https://www.genecards.org/cgi-bin/carddisp.pl?gene=TRDN
HSPA6	Heat Shock Protein Family A (Hsp70) Member 6	Protein Coding	45	GC01P161524	8.396116	https://www.genecards.org/cgi-bin/carddisp.pl?gene=HSPA6
H4-16	H4 Histone 16	Protein Coding	34	GC12M015752	8.393721	https://www.genecards.org/cgi-bin/carddisp.pl?gene=H4-16
GPT2	Glutamic--Pyruvic Transaminase 2	Protein Coding	48	GC16P046885	8.390795	https://www.genecards.org/cgi-bin/carddisp.pl?gene=GPT2
NCOR2	Nuclear Receptor Corepressor 2	Protein Coding	45	GC12M124324	8.388487	https://www.genecards.org/cgi-bin/carddisp.pl?gene=NCOR2
POMK	Protein O-Mannose Kinase	Protein Coding	38	GC08P004309 3	8.372009	https://www.genecards.org/cgi-bin/carddisp.pl?gene=POMK
APOF	Apolipoprotein F	Protein Coding	39	GC12M056371	8.370247	https://www.genecards.org/cgi-bin/carddisp.pl?gene=APOF
DSC3	Desmocollin 3	Protein Coding	43	GC18M030990	8.368109	https://www.genecards.org/cgi-bin/carddisp.pl?gene=DSC3
MIR574	MicroRNA 574	RNA Gene	18	GC04P003887 2	8.364106	https://www.genecards.org/cgi-bin/carddisp.pl?gene=MIR574
SLC25A47	Solute Carrier Family 25 Member 47	Protein Coding	35	GC14P100323	8.361531	https://www.genecards.org/cgi-bin/carddisp.pl?gene=SLC25A47
INPPL1	Inositol Polyphosphate Phosphatase Like 1	Protein Coding	50	GC11P072223	8.360861	https://www.genecards.org/cgi-bin/carddisp.pl?gene=INPPL1
BOLA3	BolA Family Member 3	Protein Coding	38	GC02M074136	8.35907	https://www.genecards.org/cgi-bin/carddisp.pl?gene=BOLA3
ST3GAL5	ST3 Beta-Galactoside Alpha-2,3-Sialyltransferase 5	Protein Coding	48	GC02M085839	8.357241	https://www.genecards.org/cgi-bin/carddisp.pl?gene=ST3GAL5

CEP63	Centrosomal Protein 63	Protein Coding	41	GC03P134485	8.354568	https://www.genecards.org/cgi-bin/carddisp.pl?gene=CEP63
PDHX	Pyruvate Dehydrogenase Complex Component X	Protein Coding	48	GC11P034894	8.349999	https://www.genecards.org/cgi-bin/carddisp.pl?gene=PDHX
SIK1	Salt Inducible Kinase 1	Protein Coding	47	GC21M043414	8.349818	https://www.genecards.org/cgi-bin/carddisp.pl?gene=SIK1
TBL2	Transducin Beta Like 2	Protein Coding	40	GC07M073568	8.347002	https://www.genecards.org/cgi-bin/carddisp.pl?gene=TBL2
CIB1	Calcium And Integrin Binding 1	Protein Coding	42	GC15M090229	8.34471	https://www.genecards.org/cgi-bin/carddisp.pl?gene=CIB1
CD24	CD24 Molecule	Protein Coding	35	GC06M106969	8.344307	https://www.genecards.org/cgi-bin/carddisp.pl?gene=CD24
UBQLN1	Ubiquilin 1	Protein Coding	43	GC09M083659	8.344234	https://www.genecards.org/cgi-bin/carddisp.pl?gene=UBQLN1
H2AX	H2A.X Variant Histone	Protein Coding	36	GC11M119097	8.340709	https://www.genecards.org/cgi-bin/carddisp.pl?gene=H2AX
TPK1	Thiamin Pyrophosphokinase 1	Protein Coding	47	GC07M144451	8.335488	https://www.genecards.org/cgi-bin/carddisp.pl?gene=TPK1
RECK	Reversion Inducing Cysteine Rich Protein With Kazal Motifs	Protein Coding	41	GC09P036036	8.331703	https://www.genecards.org/cgi-bin/carddisp.pl?gene=RECK
ALPG	Alkaline Phosphatase, Germ Cell	Protein Coding	33	GC02P232407	8.32972	https://www.genecards.org/cgi-bin/carddisp.pl?gene=ALPG
MYH2	Myosin Heavy Chain 2	Protein Coding	47	GC17M010521	8.329264	https://www.genecards.org/cgi-bin/carddisp.pl?gene=MYH2
EPHA2	EPH Receptor A2	Protein Coding	53	GC01M016124	8.328029	https://www.genecards.org/cgi-bin/carddisp.pl?gene=EPHA2
NEDD4	NEDD4 E3 Ubiquitin Protein Ligase	Protein Coding	47	GC15M055826	8.325156	https://www.genecards.org/cgi-bin/carddisp.pl?gene=NEDD4
ACAT2	Acetyl-CoA Acetyltransferase 2	Protein Coding	47	GC06P159760	8.323915	https://www.genecards.org/cgi-bin/carddisp.pl?gene=ACAT2
GPC6	Glypican 6	Protein Coding	44	GC13P093226	8.320648	https://www.genecards.org/cgi-bin/carddisp.pl?gene=GPC6
CCN1	Cellular Communication Network Factor 1	Protein Coding	31	GC01P085581	8.315063	https://www.genecards.org/cgi-bin/carddisp.pl?gene=CCN1
KCNAB2	Potassium Voltage-Gated Channel Subfamily A Regulatory Beta Subunit 2	Protein Coding	43	GC01P006020	8.309748	https://www.genecards.org/cgi-bin/carddisp.pl?gene=KCNAB2
ZNF292	Zinc Finger Protein 292	Protein Coding	36	GC06P087153	8.308033	https://www.genecards.org/cgi-bin/carddisp.pl?gene=ZNF292
CALM3	Calmodulin 3	Protein Coding	43	GC19P046601	8.303206	https://www.genecards.org/cgi-bin/carddisp.pl?gene=CALM3

CXADR	CXADR Ig-Like Cell Adhesion Molecule	Protein Coding	44	GC21P017513	8.302159	https://www.genecards.org/cgi-bin/carddisp.pl?gene=CXADR
ATOX1	Antioxidant 1 Copper Chaperone	Protein Coding	41	GC05M151743	8.299313	https://www.genecards.org/cgi-bin/carddisp.pl?gene=ATOX1
EBP	EBP Cholestenol Delta-Isomerase	Protein Coding	44	GC0XP048521	8.296646	https://www.genecards.org/cgi-bin/carddisp.pl?gene=EBP
PSMC6	Proteasome 26S Subunit, ATPase 6	Protein Coding	41	GC14P052707	8.295078	https://www.genecards.org/cgi-bin/carddisp.pl?gene=PSMC6
IER3IP1	Immediate Early Response 3 Interacting Protein 1	Protein Coding	38	GC18M047152	8.287568	https://www.genecards.org/cgi-bin/carddisp.pl?gene=IER3IP1
PRDM2	PR/SET Domain 2	Protein Coding	41	GC01P013755	8.281184	https://www.genecards.org/cgi-bin/carddisp.pl?gene=PRDM2
BUB1B	BUB1 Mitotic Checkpoint Serine/Threonine Kinase B	Protein Coding	50	GC15P040161	8.279211	https://www.genecards.org/cgi-bin/carddisp.pl?gene=BUB1B
EIF3F	Eukaryotic Translation Initiation Factor 3 Subunit F	Protein Coding	44	GC11P007966	8.27682	https://www.genecards.org/cgi-bin/carddisp.pl?gene=EIF3F
CSN1S1	Casein Alpha S1	Protein Coding	34	GC04P069932	8.275702	https://www.genecards.org/cgi-bin/carddisp.pl?gene=CSN1S1
BLOC1S3	Biogenesis Of Lysosomal Organelles Complex 1 Subunit 3	Protein Coding	36	GC19P045178	8.273204	https://www.genecards.org/cgi-bin/carddisp.pl?gene=BLOC1S3
COPS5	COP9 Signalosome Subunit 5	Protein Coding	43	GC08M067043	8.270369	https://www.genecards.org/cgi-bin/carddisp.pl?gene=COPS5
ANXA4	Annexin A4	Protein Coding	45	GC02P069644	8.268375	https://www.genecards.org/cgi-bin/carddisp.pl?gene=ANXA4
CISD2	CDGSH Iron Sulfur Domain 2	Protein Coding	42	GC04P102868	8.261267	https://www.genecards.org/cgi-bin/carddisp.pl?gene=CISD2
TREM1	Triggering Receptor Expressed On Myeloid Cells 1	Protein Coding	41	GC06M041267	8.256388	https://www.genecards.org/cgi-bin/carddisp.pl?gene=TREM1
ATP8A1	ATPase Phospholipid Transporting 8A1	Protein Coding	43	GC04M042410	8.255957	https://www.genecards.org/cgi-bin/carddisp.pl?gene=ATP8A1
ATP5F1B	ATP Synthase F1 Subunit Beta	Protein Coding	34	GC12M056639	8.253869	https://www.genecards.org/cgi-bin/carddisp.pl?gene=ATP5F1B
HLA-E	Major Histocompatibility Complex, Class I, E	Protein Coding	42	GC06P055176	8.248782	https://www.genecards.org/cgi-bin/carddisp.pl?gene=HLA-E

MIR361	MicroRNA 361	RNA Gene	16	GC0XM085903	8.242687	https://www.genecards.org/cgi-bin/carddisp.pl?gene=MIR361
SERPINB2	Serpin Family B Member 2	Protein Coding	44	GC18P063871	8.236845	https://www.genecards.org/cgi-bin/carddisp.pl?gene=SERPINB2
CRADD	CASP2 And RIPK1 Domain Containing Adaptor With Death Domain	Protein Coding	44	GC12P093677	8.234291	https://www.genecards.org/cgi-bin/carddisp.pl?gene=CRADD
ABHD2	Abhydrolase Domain Containing 2, Acylglycerol Lipase	Protein Coding	38	GC15P089087	8.227392	https://www.genecards.org/cgi-bin/carddisp.pl?gene=ABHD2
IL1RL1	Interleukin 1 Receptor Like 1	Protein Coding	41	GC02P102294	8.225016	https://www.genecards.org/cgi-bin/carddisp.pl?gene=IL1RL1
GYPB	Glycophorin B (MNS Blood Group)	Protein Coding	38	GC04M143996	8.224806	https://www.genecards.org/cgi-bin/carddisp.pl?gene=GYPB
CDIPT	CDP-Diacylglycerol--Inositol 3-Phosphatidyltransferase	Protein Coding	43	GC16M031170	8.222588	https://www.genecards.org/cgi-bin/carddisp.pl?gene=CDIPT
FSHR	Follicle Stimulating Hormone Receptor	Protein Coding	50	GC02M048953	8.221106	https://www.genecards.org/cgi-bin/carddisp.pl?gene=FSHR
SAA4	Serum Amyloid A4, Constitutive	Protein Coding	40	GC11M018234	8.220345	https://www.genecards.org/cgi-bin/carddisp.pl?gene=SAA4
NFIC	Nuclear Factor I C	Protein Coding	41	GC19P003314	8.215161	https://www.genecards.org/cgi-bin/carddisp.pl?gene=NFIC
TACR3	Tachykinin Receptor 3	Protein Coding	51	GC04M103586	8.210999	https://www.genecards.org/cgi-bin/carddisp.pl?gene=TACR3
KDSR	3-Ketodihydrosphingosine Reductase	Protein Coding	41	GC18M063327	8.208986	https://www.genecards.org/cgi-bin/carddisp.pl?gene=KDSR
PGD	Phosphogluconate Dehydrogenase	Protein Coding	47	GC01P010398	8.203134	https://www.genecards.org/cgi-bin/carddisp.pl?gene=PGD
TFE3	Transcription Factor Binding To IGHM Enhancer 3	Protein Coding	44	GC0XM049028	8.202929	https://www.genecards.org/cgi-bin/carddisp.pl?gene=TFE3
MASP1	MBL Associated Serine Protease 1	Protein Coding	47	GC03M187216	8.202531	https://www.genecards.org/cgi-bin/carddisp.pl?gene=MASP1
FUNDC2	FUN14 Domain Containing 2	Protein Coding	35	GC0XP155025	8.196218	https://www.genecards.org/cgi-bin/carddisp.pl?gene=FUNDC2

BMI1	BMI1 Proto-Oncogene, Polycomb Ring Finger	Protein Coding	44	GC10P022326	8.191759	https://www.genecards.org/cgi-bin/carddisp.pl?gene=BMI1
MSH3	MutS Homolog 3	Protein Coding	44	GC05P080654	8.187807	https://www.genecards.org/cgi-bin/carddisp.pl?gene=MSH3
AMPD3	Adenosine Monophosphate Deaminase 3	Protein Coding	46	GC11P010309	8.181871	https://www.genecards.org/cgi-bin/carddisp.pl?gene=AMPD3
SF3B2	Splicing Factor 3b Subunit 2	Protein Coding	40	GC11P066050	8.181301	https://www.genecards.org/cgi-bin/carddisp.pl?gene=SF3B2
MTA1	Metastasis Associated 1	Protein Coding	43	GC14P105419	8.178825	https://www.genecards.org/cgi-bin/carddisp.pl?gene=MTA1
TBX2	T-Box Transcription Factor 2	Protein Coding	45	GC17P061399	8.178806	https://www.genecards.org/cgi-bin/carddisp.pl?gene=TBX2
CD151	CD151 Molecule (Raph Blood Group)	Protein Coding	46	GC11P000996	8.178188	https://www.genecards.org/cgi-bin/carddisp.pl?gene=CD151
CD3E	CD3e Molecule	Protein Coding	47	GC11P118304	8.174352	https://www.genecards.org/cgi-bin/carddisp.pl?gene=CD3E
SOX4	SRY-Box Transcription Factor 4	Protein Coding	44	GC06P021593	8.172842	https://www.genecards.org/cgi-bin/carddisp.pl?gene=SOX4
MIR590	MicroRNA 590	RNA Gene	18	GC07P074191	8.172476	https://www.genecards.org/cgi-bin/carddisp.pl?gene=MIR590
ITLN1	Intelectin 1	Protein Coding	39	GC01M160876	8.169151	https://www.genecards.org/cgi-bin/carddisp.pl?gene=ITLN1
IL27	Interleukin 27	Protein Coding	38	GC16M028514	8.163682	https://www.genecards.org/cgi-bin/carddisp.pl?gene=IL27
IGFBP5	Insulin Like Growth Factor Binding Protein 5	Protein Coding	43	GC02M216672	8.163429	https://www.genecards.org/cgi-bin/carddisp.pl?gene=IGFBP5
PPM1G	Protein Phosphatase, Mg2+/Mn2+ Dependent 1G	Protein Coding	43	GC02M027399	8.16276	https://www.genecards.org/cgi-bin/carddisp.pl?gene=PPM1G
PSMC2	Proteasome 26S Subunit, ATPase 2	Protein Coding	41	GC07P103344	8.161751	https://www.genecards.org/cgi-bin/carddisp.pl?gene=PSMC2
FOXL2	Forkhead Box L2	Protein Coding	41	GC03M138944	8.161029	https://www.genecards.org/cgi-bin/carddisp.pl?gene=FOXL2
SERPINA12	Serpin Family A Member 12	Protein Coding	38	GC14M095825	8.160327	https://www.genecards.org/cgi-bin/carddisp.pl?gene=SERPINA12
PER3	Period Circadian Regulator 3	Protein Coding	44	GC01P007785	8.159691	https://www.genecards.org/cgi-bin/carddisp.pl?gene=PER3
MTAP	Methylthioadenosine Phosphorylase	Protein Coding	48	GC09P021792	8.159391	https://www.genecards.org/cgi-bin/carddisp.pl?gene=MTAP
BRD4	Bromodomain Containing 4	Protein Coding	46	GC19M015236	8.156195	https://www.genecards.org/cgi-bin/carddisp.pl?gene=BRD4
NUP214	Nucleoporin 214	Protein Coding	44	GC09P131125	8.15501	https://www.genecards.org/cgi-bin/carddisp.pl?gene=NUP214

A1CF	APOBEC1 Complementation Factor	Protein Coding	37	GC10M050799	8.146441	https://www.genecards.org/cgi-bin/carddisp.pl?gene=A1CF
SYT1	Synaptotagmin 1	Protein Coding	48	GC12P078863	8.142479	https://www.genecards.org/cgi-bin/carddisp.pl?gene=SYT1
ULK1	Unc-51 Like Autophagy Activating Kinase 1	Protein Coding	45	GC12P131894	8.140206	https://www.genecards.org/cgi-bin/carddisp.pl?gene=ULK1
RPS27	Ribosomal Protein S27	Protein Coding	43	GC01P153991	8.128654	https://www.genecards.org/cgi-bin/carddisp.pl?gene=RPS27
REG1A	Regenerating Family Member 1 Alpha	Protein Coding	41	GC02P079120	8.12756	https://www.genecards.org/cgi-bin/carddisp.pl?gene=REG1A
COLEC10	Collectin Subfamily Member 10	Protein Coding	40	GC08P118952	8.124879	https://www.genecards.org/cgi-bin/carddisp.pl?gene=COLEC10
KCNN3	Potassium Calcium-Activated Channel Subfamily N Member 3	Protein Coding	45	GC01M154697	8.123093	https://www.genecards.org/cgi-bin/carddisp.pl?gene=KCNN3
DBN1	Drebrin 1	Protein Coding	40	GC05M177456	8.118693	https://www.genecards.org/cgi-bin/carddisp.pl?gene=DBN1
SSB	Small RNA Binding Exonuclease Protection Factor La	Protein Coding	43	GC02P169791	8.116309	https://www.genecards.org/cgi-bin/carddisp.pl?gene=SSB
ALDH6A1	Aldehyde Dehydrogenase 6 Family Member A1	Protein Coding	47	GC14M074059	8.115782	https://www.genecards.org/cgi-bin/carddisp.pl?gene=ALDH6A1
CLEC16A	C-Type Lectin Domain Containing 16A	Protein Coding	37	GC16P010944	8.11161	https://www.genecards.org/cgi-bin/carddisp.pl?gene=CLEC16A
NPEPPS	Aminopeptidase Puromycin Sensitive	Protein Coding	42	GC17P047522	8.109356	https://www.genecards.org/cgi-bin/carddisp.pl?gene=NPEPPS
KDM1A	Lysine Demethylase 1A	Protein Coding	48	GC01P023019	8.106125	https://www.genecards.org/cgi-bin/carddisp.pl?gene=KDM1A
CYP2A13	Cytochrome P450 Family 2 Subfamily A Member 13	Protein Coding	42	GC19P041088	8.105606	https://www.genecards.org/cgi-bin/carddisp.pl?gene=CYP2A13
RPS6	Ribosomal Protein S6	Protein Coding	44	GC09M019375	8.104778	https://www.genecards.org/cgi-bin/carddisp.pl?gene=RPS6
HCCS	Holocytochrome C Synthase	Protein Coding	42	GC0XP011111	8.102562	https://www.genecards.org/cgi-bin/carddisp.pl?gene=HCCS
MAP3K8	Mitogen-Activated Protein Kinase Kinase Kinase 8	Protein Coding	47	GC10P030458	8.100418	https://www.genecards.org/cgi-bin/carddisp.pl?gene=MAP3K8

SLC6A9	Solute Carrier Family 6 Member 9	Protein Coding	50	GC01M043991	8.098776	https://www.genecards.org/cgi-bin/carddisp.pl?gene=SLC6A9
PPP5C	Protein Phosphatase 5 Catalytic Subunit	Protein Coding	45	GC19P046346	8.093467	https://www.genecards.org/cgi-bin/carddisp.pl?gene=PPP5C
LSS	Lanosterol Synthase	Protein Coding	46	GC21M048323	8.092251	https://www.genecards.org/cgi-bin/carddisp.pl?gene=LSS
STEAP4	STEAP4 Metalloreductase	Protein Coding	41	GC07M088315	8.090548	https://www.genecards.org/cgi-bin/carddisp.pl?gene=STEAP4
UROC1	Urocanate Hydratase 1	Protein Coding	40	GC03M126481	8.083823	https://www.genecards.org/cgi-bin/carddisp.pl?gene=UROC1
PEG10	Paternally Expressed 10	Protein Coding	41	GC07P094656	8.078861	https://www.genecards.org/cgi-bin/carddisp.pl?gene=PEG10
C12orf57	Chromosome 12 Open Reading Frame 57	Protein Coding	37	GC12P011870	8.078166	https://www.genecards.org/cgi-bin/carddisp.pl?gene=C12orf57
ABRAXAS1	Abraxas 1, BRCA1 A Complex Subunit	Protein Coding	30	GC04M083460	8.053703	https://www.genecards.org/cgi-bin/carddisp.pl?gene=ABRAXAS1
AMPH	Amphiphysin	Protein Coding	45	GC07M038782	8.049717	https://www.genecards.org/cgi-bin/carddisp.pl?gene=AMPH
AHNAK	AHNAK Nucleoprotein	Protein Coding	37	GC11M069238	8.047614	https://www.genecards.org/cgi-bin/carddisp.pl?gene=AHNAK
PTGR1	Prostaglandin Reductase 1	Protein Coding	43	GC09M111549	8.04496	https://www.genecards.org/cgi-bin/carddisp.pl?gene=PTGR1
GAB1	GRB2 Associated Binding Protein 1	Protein Coding	45	GC04P143336	8.042448	https://www.genecards.org/cgi-bin/carddisp.pl?gene=GAB1
AURKB	Aurora Kinase B	Protein Coding	50	GC17M009194	8.041707	https://www.genecards.org/cgi-bin/carddisp.pl?gene=AURKB
SPHK2	Sphingosine Kinase 2	Protein Coding	45	GC19P048619	8.038082	https://www.genecards.org/cgi-bin/carddisp.pl?gene=SPHK2
MSX2	Msh Homeobox 2	Protein Coding	48	GC05P174724	8.038054	https://www.genecards.org/cgi-bin/carddisp.pl?gene=MSX2
TFPI	Tissue Factor Pathway Inhibitor	Protein Coding	45	GC02M187464	8.02977	https://www.genecards.org/cgi-bin/carddisp.pl?gene=TFPI
TRIM5	Tripartite Motif Containing 5	Protein Coding	42	GC11M005753	8.029466	https://www.genecards.org/cgi-bin/carddisp.pl?gene=TRIM5
PXN	Paxillin	Protein Coding	46	GC12M120210	8.022019	https://www.genecards.org/cgi-bin/carddisp.pl?gene=PXN
EPHA3	EPH Receptor A3	Protein Coding	47	GC03P089077	8.020586	https://www.genecards.org/cgi-bin/carddisp.pl?gene=EPHA3
MADD	MAP Kinase Activating Death Domain	Protein Coding	42	GC11P047290	8.008732	https://www.genecards.org/cgi-bin/carddisp.pl?gene=MADD
FCER2	Fc Fragment Of IgE Receptor II	Protein Coding	45	GC19M007689	7.998783	https://www.genecards.org/cgi-bin/carddisp.pl?gene=FCER2

HSD3B2	Hydroxy-Delta-5-Steroid Dehydrogenase, 3 Beta- And Steroid Delta-Isomerase 2	Protein Coding	47	GC01P119414	7.996996	https://www.genecards.org/cgi-bin/carddisp.pl?gene=HSD3B2
DMP1	Dentin Matrix Acidic Phosphoprotein 1	Protein Coding	42	GC04P087650	7.978456	https://www.genecards.org/cgi-bin/carddisp.pl?gene=DMP1
NPY5R	Neuropeptide Y Receptor Y5	Protein Coding	43	GC04P163343	7.978161	https://www.genecards.org/cgi-bin/carddisp.pl?gene=NPY5R
DROSHA	Drosha Ribonuclease III	Protein Coding	43	GC05M031401	7.977524	https://www.genecards.org/cgi-bin/carddisp.pl?gene=DROSHA
SLC44A1	Solute Carrier Family 44 Member 1	Protein Coding	45	GC09P105244	7.974358	https://www.genecards.org/cgi-bin/carddisp.pl?gene=SLC44A1
APH1A	Aph-1 Homolog A, Gamma-Secretase Subunit	Protein Coding	43	GC01M150265	7.97285	https://www.genecards.org/cgi-bin/carddisp.pl?gene=APH1A
TRPC1	Transient Receptor Potential Cation Channel Subfamily C Member 1	Protein Coding	43	GC03P142724	7.968467	https://www.genecards.org/cgi-bin/carddisp.pl?gene=TRPC1
POLE	DNA Polymerase Epsilon, Catalytic Subunit	Protein Coding	50	GC12M132637	7.966372	https://www.genecards.org/cgi-bin/carddisp.pl?gene=POLE
HELLS	Helicase, Lymphoid Specific	Protein Coding	46	GC10P094501	7.965317	https://www.genecards.org/cgi-bin/carddisp.pl?gene=HELLS
PLD2	Phospholipase D2	Protein Coding	48	GC17P004808	7.964011	https://www.genecards.org/cgi-bin/carddisp.pl?gene=PLD2
STAG2	Stromal Antigen 2	Protein Coding	45	GC0XP123960	7.958353	https://www.genecards.org/cgi-bin/carddisp.pl?gene=STAG2
YWHAG	Tyrosine 3-Monooxygenase/Tryptophan 5-Monooxygenase Activation Protein Gamma	Protein Coding	50	GC07M076327	7.955872	https://www.genecards.org/cgi-bin/carddisp.pl?gene=YWHAG
CHRNG	Cholinergic Receptor Nicotinic Gamma Subunit	Protein Coding	41	GC02P232539	7.953361	https://www.genecards.org/cgi-bin/carddisp.pl?gene=CHRNG

DMGDH	Dimethylglycine Dehydrogenase	Protein Coding	44	GC05M078997	7.953049	https://www.genecards.org/cgi-bin/carddisp.pl?gene=DMGDH
ZNF365	Zinc Finger Protein 365	Protein Coding	38	GC10P062374	7.95028	https://www.genecards.org/cgi-bin/carddisp.pl?gene=ZNF365
SYNPO	Synaptopodin	Protein Coding	38	GC05P150601	7.948679	https://www.genecards.org/cgi-bin/carddisp.pl?gene=SYNPO
PRKAB2	Protein Kinase AMP-Activated Non-Catalytic Subunit Beta 2	Protein Coding	47	GC01M147155	7.947288	https://www.genecards.org/cgi-bin/carddisp.pl?gene=PRKAB2
PITX3	Paired Like Homeodomain 3	Protein Coding	41	GC10M102230	7.943286	https://www.genecards.org/cgi-bin/carddisp.pl?gene=PITX3
CD22	CD22 Molecule	Protein Coding	46	GC19P035319	7.941979	https://www.genecards.org/cgi-bin/carddisp.pl?gene=CD22
E2F3	E2F Transcription Factor 3	Protein Coding	43	GC06P020402	7.941366	https://www.genecards.org/cgi-bin/carddisp.pl?gene=E2F3
PPP1R3A	Protein Phosphatase 1 Regulatory Subunit 3A	Protein Coding	43	GC07M113876	7.940125	https://www.genecards.org/cgi-bin/carddisp.pl?gene=PPP1R3A
MIR675	MicroRNA 675	RNA Gene	16	GC11M001997	7.937644	https://www.genecards.org/cgi-bin/carddisp.pl?gene=MIR675
FAM20C	FAM20C Golgi Associated Secretory Pathway Kinase	Protein Coding	42	GC07P000192	7.936943	https://www.genecards.org/cgi-bin/carddisp.pl?gene=FAM20C
MAPK12	Mitogen-Activated Protein Kinase 12	Protein Coding	49	GC22M050246	7.930736	https://www.genecards.org/cgi-bin/carddisp.pl?gene=MAPK12
MIR491	MicroRNA 491	RNA Gene	19	GC09P020716	7.929972	https://www.genecards.org/cgi-bin/carddisp.pl?gene=MIR491
AFF4	AF4/FMR2 Family Member 4	Protein Coding	42	GC05M132875	7.928484	https://www.genecards.org/cgi-bin/carddisp.pl?gene=AFF4
CCT5	Chaperonin Containing TCP1 Subunit 5	Protein Coding	45	GC05P010236	7.923199	https://www.genecards.org/cgi-bin/carddisp.pl?gene=CCT5
PPP2CA	Protein Phosphatase 2 Catalytic Subunit Alpha	Protein Coding	50	GC05M134194	7.920739	https://www.genecards.org/cgi-bin/carddisp.pl?gene=PPP2CA
SRCAP	Snf2 Related CREBBP Activator Protein	Protein Coding	40	GC16P032468	7.912808	https://www.genecards.org/cgi-bin/carddisp.pl?gene=SRCAP
SETDB1	SET Domain Bifurcated Histone Lysine Methyltransferase 1	Protein Coding	43	GC01P150926	7.899225	https://www.genecards.org/cgi-bin/carddisp.pl?gene=SETDB1

CAV2	Caveolin 2	Protein Coding	43	GC07P116287	7.898973	https://www.genecards.org/cgi-bin/carddisp.pl?gene=CAV2
PROP1	PROP Paired-Like Homeobox 1	Protein Coding	41	GC05M177992	7.895069	https://www.genecards.org/cgi-bin/carddisp.pl?gene=PROP1
PXK	PX Domain Containing Serine/Threonine Kinase Like	Protein Coding	39	GC03P058333	7.895031	https://www.genecards.org/cgi-bin/carddisp.pl?gene=PXK
CALM2	Calmodulin 2	Protein Coding	45	GC02M047124	7.892985	https://www.genecards.org/cgi-bin/carddisp.pl?gene=CALM2
MYT1L	Myelin Transcription Factor 1 Like	Protein Coding	41	GC02M001789	7.88828	https://www.genecards.org/cgi-bin/carddisp.pl?gene=MYT1L
UBE3B	Ubiquitin Protein Ligase E3B	Protein Coding	44	GC12P109477	7.882938	https://www.genecards.org/cgi-bin/carddisp.pl?gene=UBE3B
ISL1	ISL LIM Homeobox 1	Protein Coding	47	GC05P051383	7.878289	https://www.genecards.org/cgi-bin/carddisp.pl?gene=ISL1
DLG3	Discs Large MAGUK Scaffold Protein 3	Protein Coding	43	GC0XP070444	7.875561	https://www.genecards.org/cgi-bin/carddisp.pl?gene=DLG3
AQP3	Aquaporin 3 (Gill Blood Group)	Protein Coding	48	GC09M033431	7.875342	https://www.genecards.org/cgi-bin/carddisp.pl?gene=AQP3
CDSN	Corneodesmosin	Protein Coding	41	GC06M031115	7.871379	https://www.genecards.org/cgi-bin/carddisp.pl?gene=CDSN
FRMD4A	FERM Domain Containing 4A	Protein Coding	37	GC10M013643	7.870653	https://www.genecards.org/cgi-bin/carddisp.pl?gene=FRMD4A
NDUFA4	NDUFA4 Mitochondrial Complex Associated	Protein Coding	44	GC07M010938	7.867542	https://www.genecards.org/cgi-bin/carddisp.pl?gene=NDUFA4
NRG3	Neuregulin 3	Protein Coding	43	GC10P085390	7.864625	https://www.genecards.org/cgi-bin/carddisp.pl?gene=NRG3
PLAG1	PLAG1 Zinc Finger	Protein Coding	40	GC08M056161	7.863031	https://www.genecards.org/cgi-bin/carddisp.pl?gene=PLAG1
MAGED2	MAGE Family Member D2	Protein Coding	40	GC0XP054807	7.861149	https://www.genecards.org/cgi-bin/carddisp.pl?gene=MAGED2
PLK1	Polo Like Kinase 1	Protein Coding	50	GC16P023958	7.860513	https://www.genecards.org/cgi-bin/carddisp.pl?gene=PLK1
DUOX1	Dual Oxidase 1	Protein Coding	42	GC15P045129	7.850125	https://www.genecards.org/cgi-bin/carddisp.pl?gene=DUOX1
PSMD10	Proteasome 26S Subunit, Non-ATPase 10	Protein Coding	41	GC0XM108084	7.84796	https://www.genecards.org/cgi-bin/carddisp.pl?gene=PSMD10
XIST	X Inactive Specific Transcript	RNA Gene	24	GC0XM073820	7.84722	https://www.genecards.org/cgi-bin/carddisp.pl?gene=XIST
SLC25A3	Solute Carrier Family 25 Member 3	Protein Coding	45	GC12P098593	7.846549	https://www.genecards.org/cgi-bin/carddisp.pl?gene=SLC25A3

PAM	Peptidylglycine Alpha-Amidating Monooxygenase	Protein Coding	44	GC05P102753	7.84424	https://www.genecards.org/cgi-bin/carddisp.pl?gene=PAM
HMMR	Hyaluronan Mediated Motility Receptor	Protein Coding	43	GC05P163480	7.843009	https://www.genecards.org/cgi-bin/carddisp.pl?gene=HMMR
APOBEC1	Apolipoprotein B MRNA Editing Enzyme Catalytic Subunit 1	Protein Coding	39	GC12M007649	7.839725	https://www.genecards.org/cgi-bin/carddisp.pl?gene=APOBEC1
ARHGEF2	Rho/Rac Guanine Nucleotide Exchange Factor 2	Protein Coding	47	GC01M155946	7.837776	https://www.genecards.org/cgi-bin/carddisp.pl?gene=ARHGEF2
TBCD	Tubulin Folding Cofactor D	Protein Coding	41	GC17P082752	7.82603	https://www.genecards.org/cgi-bin/carddisp.pl?gene=TBCD
PAK4	P21 (RAC1) Activated Kinase 4	Protein Coding	49	GC19P039125	7.82106	https://www.genecards.org/cgi-bin/carddisp.pl?gene=PAK4
TRAF1	TNF Receptor Associated Factor 1	Protein Coding	44	GC09M120902	7.819151	https://www.genecards.org/cgi-bin/carddisp.pl?gene=TRAF1
RPL27	Ribosomal Protein L27	Protein Coding	43	GC17P042998	7.817882	https://www.genecards.org/cgi-bin/carddisp.pl?gene=RPL27
TMCO1	Transmembrane And Coiled-Coil Domains 1	Protein Coding	40	GC01M165724	7.812384	https://www.genecards.org/cgi-bin/carddisp.pl?gene=TMCO1
AUP1	AUP1 Lipid Droplet Regulating VLDL Assembly Factor	Protein Coding	37	GC02M074526	7.811285	https://www.genecards.org/cgi-bin/carddisp.pl?gene=AUP1
SLC5A6	Solute Carrier Family 5 Member 6	Protein Coding	47	GC02M027201	7.809017	https://www.genecards.org/cgi-bin/carddisp.pl?gene=SLC5A6
PDPN	Podoplanin	Protein Coding	40	GC01P013583	7.803065	https://www.genecards.org/cgi-bin/carddisp.pl?gene=PDPN
KCNJ18	Potassium Inwardly Rectifying Channel Subfamily J Member 18	Protein Coding	26	GC17P029486	7.79836	https://www.genecards.org/cgi-bin/carddisp.pl?gene=KCNJ18
GSTA4	Glutathione S-Transferase Alpha 4	Protein Coding	44	GC06M052977	7.798082	https://www.genecards.org/cgi-bin/carddisp.pl?gene=GSTA4
SPOP	Speckle Type BTB/POZ Protein	Protein Coding	44	GC17M049598	7.794477	https://www.genecards.org/cgi-bin/carddisp.pl?gene=SPOP
UFM1	Ubiquitin Fold Modifier 1	Protein Coding	41	GC13P038349	7.78791	https://www.genecards.org/cgi-bin/carddisp.pl?gene=UFM1

IGFBP7	Insulin Like Growth Factor Binding Protein 7	Protein Coding	47	GC04M057030	7.786259	https://www.genecards.org/cgi-bin/carddisp.pl?gene=IGFBP7
NDUFC2-KCTD14	NDUFC2-KCTD14 Readthrough	Protein Coding	22	GC11M078015	7.785832	https://www.genecards.org/cgi-bin/carddisp.pl?gene=NDUFC2-KCTD14
CKM	Creatine Kinase, M-Type	Protein Coding	45	GC19M045306	7.776155	https://www.genecards.org/cgi-bin/carddisp.pl?gene=CKM
ST6GAL1	ST6 Beta-Galactoside Alpha-2,6-Sialyltransferase 1	Protein Coding	44	GC03P186930	7.773654	https://www.genecards.org/cgi-bin/carddisp.pl?gene=ST6GAL1
SNRNP70	Small Nuclear Ribonucleoprotein U1 Subunit 70	Protein Coding	40	GC19P049085	7.771424	https://www.genecards.org/cgi-bin/carddisp.pl?gene=SNRNP70
PHF8	PHD Finger Protein 8	Protein Coding	42	GC0XM053936	7.771198	https://www.genecards.org/cgi-bin/carddisp.pl?gene=PHF8
FES	FES Proto-Oncogene, Tyrosine Kinase	Protein Coding	48	GC15P090883	7.76962	https://www.genecards.org/cgi-bin/carddisp.pl?gene=FES
HLA-DQA2	Major Histocompatibility Complex, Class II, DQ Alpha 2	Protein Coding	37	GC06P032741	7.766288	https://www.genecards.org/cgi-bin/carddisp.pl?gene=HLA-DQA2
LRP1B	LDL Receptor Related Protein 1B	Protein Coding	40	GC02M140231	7.751904	https://www.genecards.org/cgi-bin/carddisp.pl?gene=LRP1B
LOXL2	Lysyl Oxidase Like 2	Protein Coding	44	GC08M023296	7.751643	https://www.genecards.org/cgi-bin/carddisp.pl?gene=LOXL2
VPS26A	VPS26, Retromer Complex Component A	Protein Coding	40	GC10P069123	7.749689	https://www.genecards.org/cgi-bin/carddisp.pl?gene=VPS26A
OLIG2	Oligodendrocyte Transcription Factor 2	Protein Coding	41	GC21P033025	7.748407	https://www.genecards.org/cgi-bin/carddisp.pl?gene=OLIG2
RALGAPA1	Ral GTPase Activating Protein Catalytic Subunit Alpha 1	Protein Coding	40	GC14M035538	7.748217	https://www.genecards.org/cgi-bin/carddisp.pl?gene=RALGAPA1
RAB3GAP2	RAB3 GTPase Activating Non-Catalytic Protein Subunit 2	Protein Coding	40	GC01M220149	7.741985	https://www.genecards.org/cgi-bin/carddisp.pl?gene=RAB3GAP2

LRPAP1	LDL Receptor Related Protein Associated Protein 1	Protein Coding	44	GC04M003508	7.738897	https://www.genecards.org/cgi-bin/carddisp.pl?gene=LRPAP1
PPP2R1B	Protein Phosphatase 2 Scaffold Subunit Abeta	Protein Coding	47	GC11M111695	7.738574	https://www.genecards.org/cgi-bin/carddisp.pl?gene=PPP2R1B
E2F2	E2F Transcription Factor 2	Protein Coding	43	GC01M023541	7.736928	https://www.genecards.org/cgi-bin/carddisp.pl?gene=E2F2
NRIP1	Nuclear Receptor Interacting Protein 1	Protein Coding	44	GC21M014961	7.728158	https://www.genecards.org/cgi-bin/carddisp.pl?gene=NRIP1
COL4A6	Collagen Type IV Alpha 6 Chain	Protein Coding	45	GC0XM108155	7.722342	https://www.genecards.org/cgi-bin/carddisp.pl?gene=COL4A6
DGCR8	DGCR8 Microprocessor Complex Subunit	Protein Coding	42	GC22P020080	7.721457	https://www.genecards.org/cgi-bin/carddisp.pl?gene=DGCR8
CD1C	CD1c Molecule	Protein Coding	41	GC01P158289	7.720401	https://www.genecards.org/cgi-bin/carddisp.pl?gene=CD1C
ACTL6A	Actin Like 6A	Protein Coding	42	GC03P179562	7.718887	https://www.genecards.org/cgi-bin/carddisp.pl?gene=ACTL6A
AFAP1-AS1	AFAP1 Antisense RNA 1	RNA Gene	16	GC04P007756	7.713319	https://www.genecards.org/cgi-bin/carddisp.pl?gene=AFAP1-AS1
ACAD10	Acyl-CoA Dehydrogenase Family Member 10	Protein Coding	39	GC12P111686	7.707722	https://www.genecards.org/cgi-bin/carddisp.pl?gene=ACAD10
AMFR	Autocrine Motility Factor Receptor	Protein Coding	44	GC16M056361	7.706252	https://www.genecards.org/cgi-bin/carddisp.pl?gene=AMFR
KCNA5	Potassium Voltage-Gated Channel Subfamily A Member 5	Protein Coding	46	GC12P005043	7.70451	https://www.genecards.org/cgi-bin/carddisp.pl?gene=KCNA5
OBSCN	Obscurin, Cytoskeletal Calmodulin And Titin-Interacting RhoGEF	Protein Coding	40	GC01P228208	7.704167	https://www.genecards.org/cgi-bin/carddisp.pl?gene=OBSCN
NUMA1	Nuclear Mitotic Apparatus Protein 1	Protein Coding	44	GC11M072002	7.703823	https://www.genecards.org/cgi-bin/carddisp.pl?gene=NUMA1
PUF60	Poly(U) Binding Splicing Factor 60	Protein Coding	43	GC08M143816	7.69935	https://www.genecards.org/cgi-bin/carddisp.pl?gene=PUF60
PKNOX2	PBX/Knotted 1 Homeobox 2	Protein Coding	37	GC11P125164	7.691162	https://www.genecards.org/cgi-bin/carddisp.pl?gene=PKNOX2

CDIN1	CDAN1 Interacting Nuclease 1	Protein Coding	30	GC15P036580	7.689857	https://www.genecards.org/cgi-bin/carddisp.pl?gene=CDIN1
SAMM50	SAMM50 Sorting And Assembly Machinery Component	Protein Coding	38	GC22P043955	7.688614	https://www.genecards.org/cgi-bin/carddisp.pl?gene=SAMM50
EGLN1	Egl-9 Family Hypoxia Inducible Factor 1	Protein Coding	50	GC01M231363	7.683287	https://www.genecards.org/cgi-bin/carddisp.pl?gene=EGLN1
NSD2	Nuclear Receptor Binding SET Domain Protein 2	Protein Coding	36	GC04P001872	7.682654	https://www.genecards.org/cgi-bin/carddisp.pl?gene=NSD2
ITGAV	Integrin Subunit Alpha V	Protein Coding	47	GC02P186589	7.682635	https://www.genecards.org/cgi-bin/carddisp.pl?gene=ITGAV
PRKD3	Protein Kinase D3	Protein Coding	46	GC02M037251	7.682086	https://www.genecards.org/cgi-bin/carddisp.pl?gene=PRKD3
MIR615	MicroRNA 615	RNA Gene	20	GC12P054033	7.675932	https://www.genecards.org/cgi-bin/carddisp.pl?gene=MIR615
MAPKAPK2	MAPK Activated Protein Kinase 2	Protein Coding	49	GC01P206684	7.672384	https://www.genecards.org/cgi-bin/carddisp.pl?gene=MAPKAPK2
HYAL1	Hyaluronidase 1	Protein Coding	47	GC03M050299	7.66921	https://www.genecards.org/cgi-bin/carddisp.pl?gene=HYAL1
SPRY4-IT1	SPRY4 Intronic Transcript 1	RNA Gene	15	GC05M142318	7.666531	https://www.genecards.org/cgi-bin/carddisp.pl?gene=SPRY4-IT1
PFAS	Phosphoribosylformylglycinamidine Synthase	Protein Coding	44	GC17P008247	7.664503	https://www.genecards.org/cgi-bin/carddisp.pl?gene=PFAS
ST3GAL3	ST3 Beta-Galactoside Alpha-2,3-Sialyltransferase 3	Protein Coding	47	GC01P043705	7.660644	https://www.genecards.org/cgi-bin/carddisp.pl?gene=ST3GAL3
TSHB	Thyroid Stimulating Hormone Subunit Beta	Protein Coding	43	GC01P115029	7.649669	https://www.genecards.org/cgi-bin/carddisp.pl?gene=TSHB
MMP11	Matrix Metallopeptidase 11	Protein Coding	46	GC22P023768	7.644756	https://www.genecards.org/cgi-bin/carddisp.pl?gene=MMP11
DLG1	Discs Large MAGUK Scaffold Protein 1	Protein Coding	44	GC03M197042	7.644752	https://www.genecards.org/cgi-bin/carddisp.pl?gene=DLG1
TPX2	TPX2 Microtubule Nucleation Factor	Protein Coding	42	GC20P031739	7.640526	https://www.genecards.org/cgi-bin/carddisp.pl?gene=TPX2

CAD	Carbamoyl-Phosphate Synthetase 2, Aspartate Transcarbamylase, And Dihydroorotase	Protein Coding	51	GC02P027217	7.636114	https://www.genecards.org/cgi-bin/carddisp.pl?gene=CAD
BCAR1	BCAR1 Scaffold Protein, Cas Family Member	Protein Coding	45	GC16M075228	7.63121	https://www.genecards.org/cgi-bin/carddisp.pl?gene=BCAR1
FHL2	Four And A Half LIM Domains 2	Protein Coding	47	GC02M105357	7.629701	https://www.genecards.org/cgi-bin/carddisp.pl?gene=FHL2
TLL1	Tolloid Like 1	Protein Coding	44	GC04P165873	7.624031	https://www.genecards.org/cgi-bin/carddisp.pl?gene=TLL1
RBFOX1	RNA Binding Fox-1 Homolog 1	Protein Coding	38	GC16P005688	7.623807	https://www.genecards.org/cgi-bin/carddisp.pl?gene=RBFOX1
RCAN1	Regulator Of Calcineurin 1	Protein Coding	44	GC21M034513	7.623222	https://www.genecards.org/cgi-bin/carddisp.pl?gene=RCAN1
SNTA1	Syntrophin Alpha 1	Protein Coding	45	GC20M033407	7.621838	https://www.genecards.org/cgi-bin/carddisp.pl?gene=SNTA1
RPLP0	Ribosomal Protein Lateral Stalk Subunit P0	Protein Coding	42	GC12M120196	7.61553	https://www.genecards.org/cgi-bin/carddisp.pl?gene=RPLP0
ALDH1L1	Aldehyde Dehydrogenase 1 Family Member L1	Protein Coding	41	GC03M126103	7.611825	https://www.genecards.org/cgi-bin/carddisp.pl?gene=ALDH1L1
MFN1	Mitofusin 1	Protein Coding	43	GC03P179347	7.609973	https://www.genecards.org/cgi-bin/carddisp.pl?gene=MFN1
S100A7	S100 Calcium Binding Protein A7	Protein Coding	41	GC01M153457	7.608862	https://www.genecards.org/cgi-bin/carddisp.pl?gene=S100A7
TMEM38B	Transmembrane Protein 38B	Protein Coding	43	GC09P105694	7.603172	https://www.genecards.org/cgi-bin/carddisp.pl?gene=TMEM38B
MIR26A2	MicroRNA 26a-2	RNA Gene	20	GC12M057824	7.601744	https://www.genecards.org/cgi-bin/carddisp.pl?gene=MIR26A2
CARS1	Cysteinyl-TRNA Synthetase 1	Protein Coding	34	GC11M003000	7.596472	https://www.genecards.org/cgi-bin/carddisp.pl?gene=CARS1
GSTM2	Glutathione S-Transferase Mu 2	Protein Coding	42	GC01P109668	7.592142	https://www.genecards.org/cgi-bin/carddisp.pl?gene=GSTM2
RDH11	Retinol Dehydrogenase 11	Protein Coding	45	GC14M067676	7.589113	https://www.genecards.org/cgi-bin/carddisp.pl?gene=RDH11
MEIS1	Meis Homeobox 1	Protein Coding	43	GC02P066433	7.587683	https://www.genecards.org/cgi-bin/carddisp.pl?gene=MEIS1
PSMD9	Proteasome 26S Subunit, Non-ATPase 9	Protein Coding	44	GC12P123591	7.585121	https://www.genecards.org/cgi-bin/carddisp.pl?gene=PSMD9

CPN1	Carboxypeptidase N Subunit 1	Protein Coding	44	GC10M100042	7.582891	https://www.genecards.org/cgi-bin/carddisp.pl?gene=CPN1
SPART	Spartin	Protein Coding	31	GC13M036302	7.577155	https://www.genecards.org/cgi-bin/carddisp.pl?gene=SPART
CES5A	Carboxylesterase 5A	Protein Coding	33	GC16M055881	7.57197	https://www.genecards.org/cgi-bin/carddisp.pl?gene=CES5A
FCGRT	Fc Fragment Of IgG Receptor And Transporter	Protein Coding	40	GC19P049506	7.5684	https://www.genecards.org/cgi-bin/carddisp.pl?gene=FCGRT
PSAT1	Phosphoserine Aminotransferase 1	Protein Coding	48	GC09P078297	7.566948	https://www.genecards.org/cgi-bin/carddisp.pl?gene=PSAT1
LTBP1	Latent Transforming Growth Factor Beta Binding Protein 1	Protein Coding	43	GC02P032949	7.566686	https://www.genecards.org/cgi-bin/carddisp.pl?gene=LTBP1
GPLD1	Glycosylphosphatidylinositol Specific Phospholipase D1	Protein Coding	43	GC06M024438	7.561166	https://www.genecards.org/cgi-bin/carddisp.pl?gene=GPLD1
RAPGEF3	Rap Guanine Nucleotide Exchange Factor 3	Protein Coding	45	GC12M047736	7.556888	https://www.genecards.org/cgi-bin/carddisp.pl?gene=RAPGEF3
PUS10	Pseudouridine Synthase 10	Protein Coding	35	GC02M060940	7.556578	https://www.genecards.org/cgi-bin/carddisp.pl?gene=PUS10
CDH11	Cadherin 11	Protein Coding	48	GC16M064943	7.553524	https://www.genecards.org/cgi-bin/carddisp.pl?gene=CDH11
CD2	CD2 Molecule	Protein Coding	44	GC01P116754	7.553261	https://www.genecards.org/cgi-bin/carddisp.pl?gene=CD2
PTPN6	Protein Tyrosine Phosphatase Non-Receptor Type 6	Protein Coding	50	GC12P011869	7.551253	https://www.genecards.org/cgi-bin/carddisp.pl?gene=PTPN6
PSMA3	Proteasome 20S Subunit Alpha 3	Protein Coding	45	GC14P058244	7.548352	https://www.genecards.org/cgi-bin/carddisp.pl?gene=PSMA3
CTBP2	C-Terminal Binding Protein 2	Protein Coding	45	GC10M124984	7.545674	https://www.genecards.org/cgi-bin/carddisp.pl?gene=CTBP2
PTPRG	Protein Tyrosine Phosphatase Receptor Type G	Protein Coding	43	GC03P061561	7.5432	https://www.genecards.org/cgi-bin/carddisp.pl?gene=PTPRG
ESCO2	Establishment Of Sister Chromatid Cohesion N-Acetyltransferase 2	Protein Coding	38	GC08P027771	7.536304	https://www.genecards.org/cgi-bin/carddisp.pl?gene=ESCO2

RNPC3	RNA Binding Region (RNP1, RRM) Containing 3	Protein Coding	36	GC01P103525	7.536044	https://www.genecards.org/cgi-bin/carddisp.pl?gene=RNPC3
CBR4	Carbonyl Reductase 4	Protein Coding	39	GC04M168864	7.528988	https://www.genecards.org/cgi-bin/carddisp.pl?gene=CBR4
MACROD2	Mono-ADP Ribosylhydrolase 2	Protein Coding	36	GC20P013925	7.527433	https://www.genecards.org/cgi-bin/carddisp.pl?gene=MACROD2
C12orf43	Chromosome 12 Open Reading Frame 43	Protein Coding	33	GC12M121000	7.525036	https://www.genecards.org/cgi-bin/carddisp.pl?gene=C12orf43
IFNL4	Interferon Lambda 4 (Gene/Pseudogene)	Protein Coding	19	GC19M039246	7.520307	https://www.genecards.org/cgi-bin/carddisp.pl?gene=IFNL4
ATOH7	Atonal BHLH Transcription Factor 7	Protein Coding	41	GC10M068230	7.520124	https://www.genecards.org/cgi-bin/carddisp.pl?gene=ATOH7
FREM1	FRAS1 Related Extracellular Matrix 1	Protein Coding	40	GC09M014734	7.518405	https://www.genecards.org/cgi-bin/carddisp.pl?gene=FREM1
PENK	Proenkephalin	Protein Coding	38	GC08M056436	7.517125	https://www.genecards.org/cgi-bin/carddisp.pl?gene=PENK
ATG14	Autophagy Related 14	Protein Coding	37	GC14M055366	7.515988	https://www.genecards.org/cgi-bin/carddisp.pl?gene=ATG14
PSMD2	Proteasome 26S Subunit Ubiquitin Receptor, Non-ATPase 2	Protein Coding	44	GC03P184298	7.508441	https://www.genecards.org/cgi-bin/carddisp.pl?gene=PSMD2
HMOX2	Heme Oxygenase 2	Protein Coding	48	GC16P004474	7.506993	https://www.genecards.org/cgi-bin/carddisp.pl?gene=HMOX2
ING1	Inhibitor Of Growth Family Member 1	Protein Coding	44	GC13P110712	7.504208	https://www.genecards.org/cgi-bin/carddisp.pl?gene=ING1
PRKCH	Protein Kinase C Eta	Protein Coding	51	GC14P061187	7.501637	https://www.genecards.org/cgi-bin/carddisp.pl?gene=PRKCH
NISCH	Nischarin	Protein Coding	41	GC03P052455	7.498565	https://www.genecards.org/cgi-bin/carddisp.pl?gene=NISCH
HSPE1	Heat Shock Protein Family E (Hsp10) Member 1	Protein Coding	41	GC02P197501	7.496153	https://www.genecards.org/cgi-bin/carddisp.pl?gene=HSPE1
NUP98	Nucleoporin 98 And 96 Precursor	Protein Coding	44	GC11M003671	7.492716	https://www.genecards.org/cgi-bin/carddisp.pl?gene=NUP98
SRSF1	Serine And Arginine Rich Splicing Factor 1	Protein Coding	43	GC17M058000	7.491862	https://www.genecards.org/cgi-bin/carddisp.pl?gene=SRSF1
TMSB4X	Thymosin Beta 4 X-Linked	Protein Coding	40	GC0XP012975	7.489902	https://www.genecards.org/cgi-bin/carddisp.pl?gene=TMSB4X
MST1R	Macrophage Stimulating 1 Receptor	Protein Coding	49	GC03M050077	7.487237	https://www.genecards.org/cgi-bin/carddisp.pl?gene=MST1R

ATAD3A	ATPase Family AAA Domain Containing 3A	Protein Coding	40	GC01P002047	7.482653	https://www.genecards.org/cgi-bin/carddisp.pl?gene=ATAD3A
GADD45A	Growth Arrest And DNA Damage Inducible Alpha	Protein Coding	45	GC01P067685	7.478284	https://www.genecards.org/cgi-bin/carddisp.pl?gene=GADD45A
ESRRG	Estrogen Related Receptor Gamma	Protein Coding	46	GC01M216503	7.477733	https://www.genecards.org/cgi-bin/carddisp.pl?gene=ESRRG
MIAT	Myocardial Infarction Associated Transcript	RNA Gene	23	GC22P026646	7.475718	https://www.genecards.org/cgi-bin/carddisp.pl?gene=MIAT
IGHG1	Immunoglobulin Heavy Constant Gamma 1 (G1m Marker)	Protein Coding	33	GC14M105736	7.475567	https://www.genecards.org/cgi-bin/carddisp.pl?gene=IGHG1
H2BC21	H2B Clustered Histone 21	Protein Coding	33	GC01M150135	7.474762	https://www.genecards.org/cgi-bin/carddisp.pl?gene=H2BC21
H4C1	H4 Clustered Histone 1	Protein Coding	33	GC06P054981	7.473227	https://www.genecards.org/cgi-bin/carddisp.pl?gene=H4C1
CYTOR	Cytoskeleton Regulator RNA	RNA Gene	18	GC02P087600	7.469452	https://www.genecards.org/cgi-bin/carddisp.pl?gene=CYTOR
LINC-ROR	Long Intergenic Non-Protein Coding RNA, Regulator Of Reprogramming	RNA Gene	17	GC18M057054	7.464442	https://www.genecards.org/cgi-bin/carddisp.pl?gene=LINC-ROR
TMEM176A	Transmembrane Protein 176A	Protein Coding	34	GC07P150800	7.46336	https://www.genecards.org/cgi-bin/carddisp.pl?gene=TMEM176A
KCNK9	Potassium Two Pore Domain Channel Subfamily K Member 9	Protein Coding	47	GC08M139585	7.462872	https://www.genecards.org/cgi-bin/carddisp.pl?gene=KCNK9
SIRT4	Sirtuin 4	Protein Coding	40	GC12P120291	7.461133	https://www.genecards.org/cgi-bin/carddisp.pl?gene=SIRT4
TRIM25	Tripartite Motif Containing 25	Protein Coding	44	GC17M056836	7.456665	https://www.genecards.org/cgi-bin/carddisp.pl?gene=TRIM25
FBLN1	Fibulin 1	Protein Coding	44	GC22P045502	7.454296	https://www.genecards.org/cgi-bin/carddisp.pl?gene=FBLN1
RAN	RAN, Member RAS Oncogene Family	Protein Coding	45	GC12P130871	7.447463	https://www.genecards.org/cgi-bin/carddisp.pl?gene=RAN
H3-3A	H3.3 Histone A	Protein Coding	36	GC01P226062	7.447256	https://www.genecards.org/cgi-bin/carddisp.pl?gene=H3-3A
MIR9-3	MicroRNA 9-3	RNA Gene	20	GC15P089363	7.445541	https://www.genecards.org/cgi-bin/carddisp.pl?gene=MIR9-3

GAB2	GRB2 Associated Binding Protein 2	Protein Coding	44	GC11M078215	7.444842	https://www.genecards.org/cgi-bin/carddisp.pl?gene=GAB2
URI1	URI1 Prefoldin Like Chaperone	Protein Coding	38	GC19P029923	7.441195	https://www.genecards.org/cgi-bin/carddisp.pl?gene=URI1
CLDN10	Claudin 10	Protein Coding	43	GC13P095433	7.441037	https://www.genecards.org/cgi-bin/carddisp.pl?gene=CLDN10
CSMD1	CUB And Sushi Multiple Domains 1	Protein Coding	38	GC08M002953	7.439883	https://www.genecards.org/cgi-bin/carddisp.pl?gene=CSMD1
AGMO	Alkylglycerol Monooxygenase	Protein Coding	34	GC07M015117	7.437379	https://www.genecards.org/cgi-bin/carddisp.pl?gene=AGMO
PCLO	Piccolo Presynaptic Cytomatrix Protein	Protein Coding	41	GC07M082754	7.435509	https://www.genecards.org/cgi-bin/carddisp.pl?gene=PCLO
VDAC2	Voltage Dependent Anion Channel 2	Protein Coding	44	GC10P075210	7.435448	https://www.genecards.org/cgi-bin/carddisp.pl?gene=VDAC2
UQCC2	Ubiquinol-Cytochrome C Reductase Complex Assembly Factor 2	Protein Coding	36	GC06M033694	7.435006	https://www.genecards.org/cgi-bin/carddisp.pl?gene=UQCC2
AADAC	Arylacetamide Deacetylase	Protein Coding	42	GC03P151813	7.429881	https://www.genecards.org/cgi-bin/carddisp.pl?gene=AADAC
ADAM12	ADAM Metallopeptidase Domain 12	Protein Coding	45	GC10M126012	7.425879	https://www.genecards.org/cgi-bin/carddisp.pl?gene=ADAM12
ZNF469	Zinc Finger Protein 469	Protein Coding	34	GC16P088382	7.424431	https://www.genecards.org/cgi-bin/carddisp.pl?gene=ZNF469
MAD1L1	Mitotic Arrest Deficient 1 Like 1	Protein Coding	47	GC07M001815	7.42154	https://www.genecards.org/cgi-bin/carddisp.pl?gene=MAD1L1
ANXA7	Annexin A7	Protein Coding	43	GC10M073375	7.41814	https://www.genecards.org/cgi-bin/carddisp.pl?gene=ANXA7
BNIP3	BCL2 Interacting Protein 3	Protein Coding	43	GC10M131966	7.412077	https://www.genecards.org/cgi-bin/carddisp.pl?gene=BNIP3
CPE	Carboxypeptidase E	Protein Coding	45	GC04P165361	7.411973	https://www.genecards.org/cgi-bin/carddisp.pl?gene=CPE
WDFY3	WD Repeat And FYVE Domain Containing 3	Protein Coding	41	GC04M084669	7.402159	https://www.genecards.org/cgi-bin/carddisp.pl?gene=WDFY3
MTHFD1L	Methylenetetrahydrofolate Dehydrogenase (NADP+ Dependent) 1 Like	Protein Coding	43	GC06P150865	7.399655	https://www.genecards.org/cgi-bin/carddisp.pl?gene=MTHFD1L

SOX17	SRY-Box Transcription Factor 17	Protein Coding	44	GC08P054457	7.398008	https://www.genecards.org/cgi-bin/carddisp.pl?gene=SOX17
DANCR	Differentiation Antagonizing Non-Protein Coding RNA	RNA Gene	21	GC04P052712	7.393661	https://www.genecards.org/cgi-bin/carddisp.pl?gene=DANCR
AP1S3	Adaptor Related Protein Complex 1 Subunit Sigma 3	Protein Coding	40	GC02M223751	7.390699	https://www.genecards.org/cgi-bin/carddisp.pl?gene=AP1S3
LTC4S	Leukotriene C4 Synthase	Protein Coding	43	GC05P179793	7.390403	https://www.genecards.org/cgi-bin/carddisp.pl?gene=LTC4S
MLANA	Melan-A	Protein Coding	40	GC09P005899	7.390331	https://www.genecards.org/cgi-bin/carddisp.pl?gene=MLANA
PDIA3	Protein Disulfide Isomerase Family A Member 3	Protein Coding	46	GC15P043746	7.389163	https://www.genecards.org/cgi-bin/carddisp.pl?gene=PDIA3
ILK	Integrin Linked Kinase	Protein Coding	47	GC11P006604	7.378003	https://www.genecards.org/cgi-bin/carddisp.pl?gene=ILK
GAS6	Growth Arrest Specific 6	Protein Coding	44	GC13M113820	7.37648	https://www.genecards.org/cgi-bin/carddisp.pl?gene=GAS6
IDI1	Isopentenyl-Diphosphate Delta Isomerase 1	Protein Coding	44	GC10M001039	7.375937	https://www.genecards.org/cgi-bin/carddisp.pl?gene=IDI1
TAGLN	Transgelin	Protein Coding	44	GC11P117199	7.375097	https://www.genecards.org/cgi-bin/carddisp.pl?gene=TAGLN
MYO1B	Myosin IB	Protein Coding	39	GC02P191246	7.3727	https://www.genecards.org/cgi-bin/carddisp.pl?gene=MYO1B
ICAM3	Intercellular Adhesion Molecule 3	Protein Coding	42	GC19M010335	7.370205	https://www.genecards.org/cgi-bin/carddisp.pl?gene=ICAM3
RPS15A	Ribosomal Protein S15a	Protein Coding	43	GC16M018781	7.369634	https://www.genecards.org/cgi-bin/carddisp.pl?gene=RPS15A
FAF1	Fas Associated Factor 1	Protein Coding	44	GC01M050439	7.366925	https://www.genecards.org/cgi-bin/carddisp.pl?gene=FAF1
UBTF	Upstream Binding Transcription Factor	Protein Coding	44	GC17M044205	7.360384	https://www.genecards.org/cgi-bin/carddisp.pl?gene=UBTF
LGALS9	Galectin 9	Protein Coding	40	GC17P027629	7.359166	https://www.genecards.org/cgi-bin/carddisp.pl?gene=LGALS9
PROZ	Protein Z, Vitamin K Dependent Plasma Glycoprotein	Protein Coding	40	GC13P113158	7.358657	https://www.genecards.org/cgi-bin/carddisp.pl?gene=PROZ
RPL7	Ribosomal Protein L7	Protein Coding	43	GC08M073290	7.352774	https://www.genecards.org/cgi-bin/carddisp.pl?gene=RPL7
ZDHHC2	Zinc Finger DHHC-Type Palmitoyltransferase 2	Protein Coding	39	GC08P017156	7.350923	https://www.genecards.org/cgi-bin/carddisp.pl?gene=ZDHHC2

IQGAP1	IQ Motif Containing GTPase Activating Protein 1	Protein Coding	44	GC15P090388	7.34699	https://www.genecards.org/cgi-bin/carddisp.pl?gene=IQGAP1
BIRC2	Baculoviral IAP Repeat Containing 2	Protein Coding	46	GC11P102347	7.346878	https://www.genecards.org/cgi-bin/carddisp.pl?gene=BIRC2
SLC25A24	Solute Carrier Family 25 Member 24	Protein Coding	44	GC01M108134	7.345687	https://www.genecards.org/cgi-bin/carddisp.pl?gene=SLC25A24
PMVK	Phosphomevalonate Kinase	Protein Coding	45	GC01M154924	7.338291	https://www.genecards.org/cgi-bin/carddisp.pl?gene=PMVK
SVBP	Small Vasohibin Binding Protein	Protein Coding	27	GC01M042808	7.330774	https://www.genecards.org/cgi-bin/carddisp.pl?gene=SVBP
CCL22	C-C Motif Chemokine Ligand 22	Protein Coding	38	GC16P057359	7.329659	https://www.genecards.org/cgi-bin/carddisp.pl?gene=CCL22
SAT1	Spermidine/Spermine N1-Acetyltransferase 1	Protein Coding	46	GC0XP023784	7.311718	https://www.genecards.org/cgi-bin/carddisp.pl?gene=SAT1
CCL27	C-C Motif Chemokine Ligand 27	Protein Coding	38	GC09M034662	7.310849	https://www.genecards.org/cgi-bin/carddisp.pl?gene=CCL27
RIPK2	Receptor Interacting Serine/Threonine Kinase 2	Protein Coding	47	GC08P089758	7.309886	https://www.genecards.org/cgi-bin/carddisp.pl?gene=RIPK2
NECTIN2	Nectin Cell Adhesion Molecule 2	Protein Coding	37	GC19P044849	7.304943	https://www.genecards.org/cgi-bin/carddisp.pl?gene=NECTIN2
ARF6	ADP Ribosylation Factor 6	Protein Coding	45	GC14P049895	7.303465	https://www.genecards.org/cgi-bin/carddisp.pl?gene=ARF6
KCNK3	Potassium Two Pore Domain Channel Subfamily K Member 3	Protein Coding	50	GC02P026692	7.295532	https://www.genecards.org/cgi-bin/carddisp.pl?gene=KCNK3
ANO3	Anoctamin 3	Protein Coding	39	GC11P026188	7.295217	https://www.genecards.org/cgi-bin/carddisp.pl?gene=ANO3
AMBRA1	Autophagy And Beclin 1 Regulator 1	Protein Coding	39	GC11M068969	7.29107	https://www.genecards.org/cgi-bin/carddisp.pl?gene=AMBRA1
CEP85L	Centrosomal Protein 85 Like	Protein Coding	35	GC06M118460	7.290624	https://www.genecards.org/cgi-bin/carddisp.pl?gene=CEP85L
LPAR6	Lysophosphatidic Acid Receptor 6	Protein Coding	46	GC13M048389	7.288377	https://www.genecards.org/cgi-bin/carddisp.pl?gene=LPAR6
TNFSF13	TNF Superfamily Member 13	Protein Coding	45	GC17P007558	7.284657	https://www.genecards.org/cgi-bin/carddisp.pl?gene=TNFSF13
CMA1	Chymase 1	Protein Coding	44	GC14M024506	7.282912	https://www.genecards.org/cgi-bin/carddisp.pl?gene=CMA1

CD5L	CD5 Molecule Like	Protein Coding	39	GC01M157800	7.271056	https://www.genecards.org/cgi-bin/carddisp.pl?gene=CD5L
PZP	PZP Alpha-2-Macroglobulin Like	Protein Coding	40	GC12M009148	7.263736	https://www.genecards.org/cgi-bin/carddisp.pl?gene=PZP
PSMB5	Proteasome 20S Subunit Beta 5	Protein Coding	45	GC14M023016	7.252934	https://www.genecards.org/cgi-bin/carddisp.pl?gene=PSMB5
CSNK1E	Casein Kinase 1 Epsilon	Protein Coding	48	GC22M048647	7.252754	https://www.genecards.org/cgi-bin/carddisp.pl?gene=CSNK1E
RIPK3	Receptor Interacting Serine/Threonine Kinase 3	Protein Coding	43	GC14M024336	7.244665	https://www.genecards.org/cgi-bin/carddisp.pl?gene=RIPK3
FBL	Fibrillarin	Protein Coding	44	GC19M039834	7.244516	https://www.genecards.org/cgi-bin/carddisp.pl?gene=FBL
ZNF335	Zinc Finger Protein 335	Protein Coding	38	GC20M045948	7.24309	https://www.genecards.org/cgi-bin/carddisp.pl?gene=ZNF335
UBA5	Ubiquitin Like Modifier Activating Enzyme 5	Protein Coding	43	GC03P132654	7.242118	https://www.genecards.org/cgi-bin/carddisp.pl?gene=UBA5
KLC1	Kinesin Light Chain 1	Protein Coding	42	GC14P106043	7.239972	https://www.genecards.org/cgi-bin/carddisp.pl?gene=KLC1
SMARCD1	SWI/SNF Related, Matrix Associated, Actin Dependent Regulator Of Chromatin, Subfamily D, Member 1	Protein Coding	43	GC12P050085	7.238363	https://www.genecards.org/cgi-bin/carddisp.pl?gene=SMARCD1
CSTA	Cystatin A	Protein Coding	44	GC03P122325	7.232214	https://www.genecards.org/cgi-bin/carddisp.pl?gene=CSTA
STT3A	STT3 Oligosaccharyltransferase Complex Catalytic Subunit A	Protein Coding	45	GC11P125592	7.230584	https://www.genecards.org/cgi-bin/carddisp.pl?gene=STT3A
DCLK1	Doublecortin Like Kinase 1	Protein Coding	44	GC13M035768	7.229346	https://www.genecards.org/cgi-bin/carddisp.pl?gene=DCLK1
TRIM63	Tripartite Motif Containing 63	Protein Coding	41	GC01M026062	7.22347	https://www.genecards.org/cgi-bin/carddisp.pl?gene=TRIM63
EXOC3L2	Exocyst Complex Component 3 Like 2	Protein Coding	34	GC19M045212	7.218055	https://www.genecards.org/cgi-bin/carddisp.pl?gene=EXOC3L2
PRDX4	Peroxiredoxin 4	Protein Coding	44	GC0XP023665	7.215439	https://www.genecards.org/cgi-bin/carddisp.pl?gene=PRDX4
SLC15A1	Solute Carrier Family 15 Member 1	Protein Coding	45	GC13M098683	7.212022	https://www.genecards.org/cgi-bin/carddisp.pl?gene=SLC15A1
UBE2D2	Ubiquitin Conjugating Enzyme E2 D2	Protein Coding	45	GC05P139526	7.208131	https://www.genecards.org/cgi-bin/carddisp.pl?gene=UBE2D2

MIR136	MicroRNA 136	RNA Gene	19	GC14P105979	7.201509	https://www.genecards.org/cgi-bin/carddisp.pl?gene=MIR136
CLEC12A	C-Type Lectin Domain Family 12 Member A	Protein Coding	38	GC12P009951	7.200759	https://www.genecards.org/cgi-bin/carddisp.pl?gene=CLEC12A
LGALS3BP	Galectin 3 Binding Protein	Protein Coding	41	GC17M078971	7.200254	https://www.genecards.org/cgi-bin/carddisp.pl?gene=LGALS3BP
SLC5A8	Solute Carrier Family 5 Member 8	Protein Coding	43	GC12M101155	7.198617	https://www.genecards.org/cgi-bin/carddisp.pl?gene=SLC5A8
MIR494	MicroRNA 494	RNA Gene	16	GC14P106333	7.196767	https://www.genecards.org/cgi-bin/carddisp.pl?gene=MIR494
RBX1	Ring-Box 1	Protein Coding	43	GC22P040951	7.196194	https://www.genecards.org/cgi-bin/carddisp.pl?gene=RBX1
SETD1A	SET Domain Containing 1A, Histone Lysine Methyltransferase	Protein Coding	41	GC16P032486	7.195914	https://www.genecards.org/cgi-bin/carddisp.pl?gene=SETD1A
CYP2A7	Cytochrome P450 Family 2 Subfamily A Member 7	Protein Coding	39	GC19M040875	7.193451	https://www.genecards.org/cgi-bin/carddisp.pl?gene=CYP2A7
HM13	Histocompatibility Minor 13	Protein Coding	39	GC20P031514	7.192185	https://www.genecards.org/cgi-bin/carddisp.pl?gene=HM13
CEP152	Centrosomal Protein 152	Protein Coding	39	GC15M048663	7.186653	https://www.genecards.org/cgi-bin/carddisp.pl?gene=CEP152
S100A6	S100 Calcium Binding Protein A6	Protein Coding	43	GC01M153535	7.183607	https://www.genecards.org/cgi-bin/carddisp.pl?gene=S100A6
TMLHE	Trimethyllysine Hydroxylase, Epsilon	Protein Coding	43	GC0XM155489	7.180274	https://www.genecards.org/cgi-bin/carddisp.pl?gene=TMLHE
CA9	Carbonic Anhydrase 9	Protein Coding	45	GC09P035673	7.177135	https://www.genecards.org/cgi-bin/carddisp.pl?gene=CA9
MAX	MYC Associated Factor X	Protein Coding	48	GC14M065009	7.176951	https://www.genecards.org/cgi-bin/carddisp.pl?gene=MAX
PPARGC1B	PPARG Coactivator 1 Beta	Protein Coding	36	GC05P149730	7.171171	https://www.genecards.org/cgi-bin/carddisp.pl?gene=PPARGC1B
ACYP1	Acylphosphatase 1	Protein Coding	38	GC14M075053	7.169619	https://www.genecards.org/cgi-bin/carddisp.pl?gene=ACYP1
E2F4	E2F Transcription Factor 4	Protein Coding	46	GC16P067192	7.16264	https://www.genecards.org/cgi-bin/carddisp.pl?gene=E2F4
TK1	Thymidine Kinase 1	Protein Coding	47	GC17M078175	7.157782	https://www.genecards.org/cgi-bin/carddisp.pl?gene=TK1
PTGER2	Prostaglandin E Receptor 2	Protein Coding	49	GC14P052314	7.149096	https://www.genecards.org/cgi-bin/carddisp.pl?gene=PTGER2
SLC22A3	Solute Carrier Family 22 Member 3	Protein Coding	45	GC06P160348	7.147097	https://www.genecards.org/cgi-bin/carddisp.pl?gene=SLC22A3

MAP3K11	Mitogen-Activated Protein Kinase Kinase Kinase 11	Protein Coding	48	GC11M069402	7.14581	https://www.genecards.org/cgi-bin/carddisp.pl?gene=MAP3K11
TAF6	TATA-Box Binding Protein Associated Factor 6	Protein Coding	41	GC07M100107	7.142289	https://www.genecards.org/cgi-bin/carddisp.pl?gene=TAF6
MACROH2A1	MacroH2A.1 Histone	Protein Coding	36	GC05M135334	7.131829	https://www.genecards.org/cgi-bin/carddisp.pl?gene=MACROH2A1
INPP4A	Inositol Polyphosphate-4-Phosphatase Type I A	Protein Coding	43	GC02P098465	7.124414	https://www.genecards.org/cgi-bin/carddisp.pl?gene=INPP4A
APOC4	Apolipoprotein C4	Protein Coding	36	GC19P044943	7.117778	https://www.genecards.org/cgi-bin/carddisp.pl?gene=APOC4
PREP	Prolyl Endopeptidase	Protein Coding	43	GC06M105277	7.115729	https://www.genecards.org/cgi-bin/carddisp.pl?gene=PREP
DNM3	Dynamin 3	Protein Coding	44	GC01P171841	7.115562	https://www.genecards.org/cgi-bin/carddisp.pl?gene=DNM3
DDHD1	DDHD Domain Containing 1	Protein Coding	39	GC14M053036	7.115196	https://www.genecards.org/cgi-bin/carddisp.pl?gene=DDHD1
RPS25	Ribosomal Protein S25	Protein Coding	38	GC11M119015	7.112805	https://www.genecards.org/cgi-bin/carddisp.pl?gene=RPS25
SLC5A1	Solute Carrier Family 5 Member 1	Protein Coding	50	GC22P032043	7.112461	https://www.genecards.org/cgi-bin/carddisp.pl?gene=SLC5A1
HSD17B3	Hydroxysteroid 17-Beta Dehydrogenase 3	Protein Coding	48	GC09M096240	7.112155	https://www.genecards.org/cgi-bin/carddisp.pl?gene=HSD17B3
MIR125B2	MicroRNA 125b-2	RNA Gene	21	GC21P016590	7.100262	https://www.genecards.org/cgi-bin/carddisp.pl?gene=MIR125B2
TRIO	Trio Rho Guanine Nucleotide Exchange Factor	Protein Coding	45	GC05P014143	7.086922	https://www.genecards.org/cgi-bin/carddisp.pl?gene=TRIO
NUDT7	Nudix Hydrolase 7	Protein Coding	38	GC16P077722	7.081073	https://www.genecards.org/cgi-bin/carddisp.pl?gene=NUDT7
HSPA2	Heat Shock Protein Family A (Hsp70) Member 2	Protein Coding	44	GC14P064535	7.076354	https://www.genecards.org/cgi-bin/carddisp.pl?gene=HSPA2
ASNS	Asparagine Synthetase (Glutamine-Hydrolyzing)	Protein Coding	47	GC07M097854	7.069455	https://www.genecards.org/cgi-bin/carddisp.pl?gene=ASNS
GJA4	Gap Junction Protein Alpha 4	Protein Coding	44	GC01P034792	7.066598	https://www.genecards.org/cgi-bin/carddisp.pl?gene=GJA4
INSIG2	Insulin Induced Gene 2	Protein Coding	41	GC02P118088	7.063419	https://www.genecards.org/cgi-bin/carddisp.pl?gene=INSIG2

OSMR	Oncostatin M Receptor	Protein Coding	45	GC05P038845	7.063321	https://www.genecards.org/cgi-bin/carddisp.pl?gene=OSMR
SERPINB5	Serpin Family B Member 5	Protein Coding	44	GC18P063476	7.055657	https://www.genecards.org/cgi-bin/carddisp.pl?gene=SERPINB5
PTGES	Prostaglandin E Synthase	Protein Coding	42	GC09M129738	7.054958	https://www.genecards.org/cgi-bin/carddisp.pl?gene=PTGES
LYVE1	Lymphatic Vessel Endothelial Hyaluronan Receptor 1	Protein Coding	43	GC11M010713	7.053092	https://www.genecards.org/cgi-bin/carddisp.pl?gene=LYVE1
SOX11	SRY-Box Transcription Factor 11	Protein Coding	42	GC02P005703	7.051542	https://www.genecards.org/cgi-bin/carddisp.pl?gene=SOX11
CSF2RA	Colony Stimulating Factor 2 Receptor Subunit Alpha	Protein Coding	46	GC0XP001333	7.043605	https://www.genecards.org/cgi-bin/carddisp.pl?gene=CSF2RA
GEMIN4	Gem Nuclear Organelle Associated Protein 4	Protein Coding	39	GC17M000744	7.038875	https://www.genecards.org/cgi-bin/carddisp.pl?gene=GEMIN4
AQP5	Aquaporin 5	Protein Coding	47	GC12P049961	7.038698	https://www.genecards.org/cgi-bin/carddisp.pl?gene=AQP5
H1-4	H1.4 Linker Histone, Cluster Member	Protein Coding	36	GC06P055576	7.035132	https://www.genecards.org/cgi-bin/carddisp.pl?gene=H1-4
VAT1	Vesicle Amine Transport 1	Protein Coding	38	GC17M043014	7.031759	https://www.genecards.org/cgi-bin/carddisp.pl?gene=VAT1
CMKLR1	Chemerin Chemokine-Like Receptor 1	Protein Coding	41	GC12M108288	7.027706	https://www.genecards.org/cgi-bin/carddisp.pl?gene=CMKLR1
CENPJ	Centromere Protein J	Protein Coding	43	GC13M024882	7.025882	https://www.genecards.org/cgi-bin/carddisp.pl?gene=CENPJ
POLR2B	RNA Polymerase II Subunit B	Protein Coding	43	GC04P056977	7.024299	https://www.genecards.org/cgi-bin/carddisp.pl?gene=POLR2B
BRD7	Bromodomain Containing 7	Protein Coding	43	GC16M050313	7.023968	https://www.genecards.org/cgi-bin/carddisp.pl?gene=BRD7
VPS51	VPS51 Subunit Of GARP Complex	Protein Coding	37	GC11P065089	7.020533	https://www.genecards.org/cgi-bin/carddisp.pl?gene=VPS51
SDR9C7	Short Chain Dehydrogenase/Reductase Family 9C Member 7	Protein Coding	38	GC12M056923	7.01707	https://www.genecards.org/cgi-bin/carddisp.pl?gene=SDR9C7
CHGB	Chromogranin B	Protein Coding	40	GC20P005911	7.013568	https://www.genecards.org/cgi-bin/carddisp.pl?gene=CHGB
ADAM9	ADAM Metallopeptidase Domain 9	Protein Coding	48	GC08P038996	7.013069	https://www.genecards.org/cgi-bin/carddisp.pl?gene=ADAM9
SULF1	Sulfatase 1	Protein Coding	42	GC08P069466	7.010864	https://www.genecards.org/cgi-bin/carddisp.pl?gene=SULF1

MYF5	Myogenic Factor 5	Protein Coding	41	GC12P080716	7.008314	https://www.genecards.org/cgi-bin/carddisp.pl?gene=MYF5
AOC1	Amine Oxidase Copper Containing 1	Protein Coding	40	GC07P150824	7.006145	https://www.genecards.org/cgi-bin/carddisp.pl?gene=AOC1
HSPB3	Heat Shock Protein Family B (Small) Member 3	Protein Coding	40	GC05P054456	6.995833	https://www.genecards.org/cgi-bin/carddisp.pl?gene=HSPB3
CCNH	Cyclin H	Protein Coding	47	GC05M087311	6.994796	https://www.genecards.org/cgi-bin/carddisp.pl?gene=CCNH
SLC5A2	Solute Carrier Family 5 Member 2	Protein Coding	47	GC16P032579	6.99471	https://www.genecards.org/cgi-bin/carddisp.pl?gene=SLC5A2
JUND	JunD Proto-Oncogene, AP-1 Transcription Factor Subunit	Protein Coding	43	GC19M018279	6.993617	https://www.genecards.org/cgi-bin/carddisp.pl?gene=JUND
WNT2B	Wnt Family Member 2B	Protein Coding	45	GC01P112466	6.985282	https://www.genecards.org/cgi-bin/carddisp.pl?gene=WNT2B
TGM3	Transglutaminase 3	Protein Coding	43	GC20P002296	6.98355	https://www.genecards.org/cgi-bin/carddisp.pl?gene=TGM3
B3GAT1	Beta-1,3-Glucuronyltransferase 1	Protein Coding	44	GC11M134378	6.974327	https://www.genecards.org/cgi-bin/carddisp.pl?gene=B3GAT1
MIR296	MicroRNA 296	RNA Gene	17	GC20M058817	6.972168	https://www.genecards.org/cgi-bin/carddisp.pl?gene=MIR296
PSMC3	Proteasome 26S Subunit, ATPase 3	Protein Coding	43	GC11M069001	6.966324	https://www.genecards.org/cgi-bin/carddisp.pl?gene=PSMC3
CAMKK2	Calcium/Calmodulin Dependent Protein Kinase Kinase 2	Protein Coding	47	GC12M121496	6.966138	https://www.genecards.org/cgi-bin/carddisp.pl?gene=CAMKK2
FDX1	Ferredoxin 1	Protein Coding	41	GC11P110429	6.962153	https://www.genecards.org/cgi-bin/carddisp.pl?gene=FDX1
CANT1	Calcium Activated Nucleotidase 1	Protein Coding	45	GC17M078992	6.959641	https://www.genecards.org/cgi-bin/carddisp.pl?gene=CANT1
LUM	Lumican	Protein Coding	42	GC12M091102	6.958346	https://www.genecards.org/cgi-bin/carddisp.pl?gene=LUM
IFT57	Intraflagellar Transport 57	Protein Coding	40	GC03M108160	6.954612	https://www.genecards.org/cgi-bin/carddisp.pl?gene=IFT57
LAMA1	Laminin Subunit Alpha 1	Protein Coding	47	GC18M006941	6.948433	https://www.genecards.org/cgi-bin/carddisp.pl?gene=LAMA1
MIR532	MicroRNA 532	RNA Gene	16	GC0XP050004	6.94752	https://www.genecards.org/cgi-bin/carddisp.pl?gene=MIR532
CKB	Creatine Kinase B	Protein Coding	46	GC14M103519	6.943268	https://www.genecards.org/cgi-bin/carddisp.pl?gene=CKB
GNRHR	Gonadotropin Releasing Hormone Receptor	Protein Coding	48	GC04M067737	6.940283	https://www.genecards.org/cgi-bin/carddisp.pl?gene=GNRHR
CUL1	Cullin 1	Protein Coding	45	GC07P148697	6.936001	https://www.genecards.org/cgi-bin/carddisp.pl?gene=CUL1

SMPD2	Sphingomyelin Phosphodiesterase 2	Protein Coding	41	GC06P109440	6.931005	https://www.genecards.org/cgi-bin/carddisp.pl?gene=SMPD2
LDHB	Lactate Dehydrogenase B	Protein Coding	47	GC12M021635	6.926804	https://www.genecards.org/cgi-bin/carddisp.pl?gene=LDHB
POLA1	DNA Polymerase Alpha 1, Catalytic Subunit	Protein Coding	46	GC0XP024693	6.925795	https://www.genecards.org/cgi-bin/carddisp.pl?gene=POLA1
RBM20	RNA Binding Motif Protein 20	Protein Coding	36	GC10P110644	6.923946	https://www.genecards.org/cgi-bin/carddisp.pl?gene=RBM20
PTDSS1	Phosphatidylserine Synthase 1	Protein Coding	45	GC08P096261	6.920557	https://www.genecards.org/cgi-bin/carddisp.pl?gene=PTDSS1
NRGN	Neurogranin	Protein Coding	39	GC11P124739	6.917172	https://www.genecards.org/cgi-bin/carddisp.pl?gene=NRGN
EFNB1	Ephrin B1	Protein Coding	47	GC0XP068828	6.912486	https://www.genecards.org/cgi-bin/carddisp.pl?gene=EFNB1
FLOT1	Flotillin 1	Protein Coding	42	GC06M046803	6.89647	https://www.genecards.org/cgi-bin/carddisp.pl?gene=FLOT1
PBRM1	Polybromo 1	Protein Coding	45	GC03M052545	6.892432	https://www.genecards.org/cgi-bin/carddisp.pl?gene=PBRM1
POU1F1	POU Class 1 Homeobox 1	Protein Coding	44	GC03M087259	6.891148	https://www.genecards.org/cgi-bin/carddisp.pl?gene=POU1F1
MGAT1	Alpha-1,3-Mannosyl-Glycoprotein 2-Beta-N-Acetylglucosaminyltransferase	Protein Coding	44	GC05M180936	6.882869	https://www.genecards.org/cgi-bin/carddisp.pl?gene=MGAT1
SLURP1	Secreted LY6/PLAUR Domain Containing 1	Protein Coding	43	GC08M142740	6.877222	https://www.genecards.org/cgi-bin/carddisp.pl?gene=SLURP1
RDX	Radixin	Protein Coding	50	GC11M109864	6.876036	https://www.genecards.org/cgi-bin/carddisp.pl?gene=RDX
ONECUT2	One Cut Homeobox 2	Protein Coding	36	GC18P057436	6.871656	https://www.genecards.org/cgi-bin/carddisp.pl?gene=ONECUT2
RER1	Retention In Endoplasmic Reticulum Sorting Receptor 1	Protein Coding	35	GC01P002391	6.869596	https://www.genecards.org/cgi-bin/carddisp.pl?gene=RER1
MCPH1	Microcephalin 1	Protein Coding	41	GC08P006406	6.862211	https://www.genecards.org/cgi-bin/carddisp.pl?gene=MCPH1
NFU1	NFU1 Iron-Sulfur Cluster Scaffold	Protein Coding	43	GC02M069395	6.861397	https://www.genecards.org/cgi-bin/carddisp.pl?gene=NFU1
BDH1	3-Hydroxybutyrate Dehydrogenase 1	Protein Coding	44	GC03M197519	6.854402	https://www.genecards.org/cgi-bin/carddisp.pl?gene=BDH1

LPAR1	Lysophosphatidic Acid Receptor 1	Protein Coding	47	GC09M110873	6.854399	https://www.genecards.org/cgi-bin/carddisp.pl?gene=LPAR1
TRPV6	Transient Receptor Potential Cation Channel Subfamily V Member 6	Protein Coding	47	GC07M142871	6.849349	https://www.genecards.org/cgi-bin/carddisp.pl?gene=TRPV6
CBFB	Core-Binding Factor Subunit Beta	Protein Coding	45	GC16P067063	6.847279	https://www.genecards.org/cgi-bin/carddisp.pl?gene=CBFB
KHDRBS3	KH RNA Binding Domain Containing, Signal Transduction Associated 3	Protein Coding	39	GC08P135457	6.846521	https://www.genecards.org/cgi-bin/carddisp.pl?gene=KHDRBS3
TULP3	TUB Like Protein 3	Protein Coding	41	GC12P002870	6.843833	https://www.genecards.org/cgi-bin/carddisp.pl?gene=TULP3
MYOM1	Myomesin 1	Protein Coding	40	GC18M003066	6.843328	https://www.genecards.org/cgi-bin/carddisp.pl?gene=MYOM1
BCL2L2	BCL2 Like 2	Protein Coding	45	GC14P026355	6.826106	https://www.genecards.org/cgi-bin/carddisp.pl?gene=BCL2L2
RICTOR	RPTOR Independent Companion Of MTOR Complex 2	Protein Coding	45	GC05M038937	6.825677	https://www.genecards.org/cgi-bin/carddisp.pl?gene=RICTOR
HNRNPC	Heterogeneous Nuclear Ribonucleoprotein C	Protein Coding	42	GC14M021210	6.823275	https://www.genecards.org/cgi-bin/carddisp.pl?gene=HNRNPC
MTDH	Metadherin	Protein Coding	41	GC08P097643	6.820546	https://www.genecards.org/cgi-bin/carddisp.pl?gene=MTDH
MEOX2	Mesenchyme Homeobox 2	Protein Coding	43	GC07M015617	6.816932	https://www.genecards.org/cgi-bin/carddisp.pl?gene=MEOX2
RBM39	RNA Binding Motif Protein 39	Protein Coding	38	GC20M035703	6.813349	https://www.genecards.org/cgi-bin/carddisp.pl?gene=RBM39
HPR	Haptoglobin-Related Protein	Protein Coding	40	GC16P072097	6.810152	https://www.genecards.org/cgi-bin/carddisp.pl?gene=HPR
KCND2	Potassium Voltage-Gated Channel Subfamily D Member 2	Protein Coding	45	GC07P120273	6.809456	https://www.genecards.org/cgi-bin/carddisp.pl?gene=KCND2
SH2B1	SH2B Adaptor Protein 1	Protein Coding	44	GC16P032268	6.80704	https://www.genecards.org/cgi-bin/carddisp.pl?gene=SH2B1
FPGS	Folylpolyglutamate Synthase	Protein Coding	43	GC09P127794	6.807037	https://www.genecards.org/cgi-bin/carddisp.pl?gene=FPGS
LNPEP	Leucyl And Cystinyl Aminopeptidase	Protein Coding	46	GC05P096935	6.797159	https://www.genecards.org/cgi-bin/carddisp.pl?gene=LNPEP

NNT	Nicotinamide Nucleotide Transhydrogenase	Protein Coding	46	GC05P043668	6.795671	https://www.genecards.org/cgi-bin/carddisp.pl?gene=NNT
CHN1	Chimerin 1	Protein Coding	48	GC02M174799	6.795224	https://www.genecards.org/cgi-bin/carddisp.pl?gene=CHN1
SELENOI	Selenoprotein I	Protein Coding	30	GC02P026310	6.792241	https://www.genecards.org/cgi-bin/carddisp.pl?gene=SELENOI
MCTP2	Multiple C2 And Transmembrane Domain Containing 2	Protein Coding	41	GC15P100162	6.787868	https://www.genecards.org/cgi-bin/carddisp.pl?gene=MCTP2
INSIG1	Insulin Induced Gene 1	Protein Coding	40	GC07P155297	6.787679	https://www.genecards.org/cgi-bin/carddisp.pl?gene=INSIG1
GALNT2	Polypeptide N-Acetylgalactosaminyltransferase 2	Protein Coding	47	GC01P230057	6.787605	https://www.genecards.org/cgi-bin/carddisp.pl?gene=GALNT2
GNAI3	G Protein Subunit Alpha I3	Protein Coding	48	GC01P109548	6.782274	https://www.genecards.org/cgi-bin/carddisp.pl?gene=GNAI3
DEGS2	Delta 4-Desaturase, Sphingolipid 2	Protein Coding	38	GC14M100143	6.781742	https://www.genecards.org/cgi-bin/carddisp.pl?gene=DEGS2
SEMA5A	Semaphorin 5A	Protein Coding	41	GC05M009036	6.778819	https://www.genecards.org/cgi-bin/carddisp.pl?gene=SEMA5A
PITRM1	Pitrilysin Metallopeptidase 1	Protein Coding	40	GC10M003138	6.769207	https://www.genecards.org/cgi-bin/carddisp.pl?gene=PITRM1
HAS2	Hyaluronan Synthase 2	Protein Coding	42	GC08M121594	6.768855	https://www.genecards.org/cgi-bin/carddisp.pl?gene=HAS2
ELAVL4	ELAV Like RNA Binding Protein 4	Protein Coding	40	GC01P050025	6.762087	https://www.genecards.org/cgi-bin/carddisp.pl?gene=ELAVL4
NFIA	Nuclear Factor I A	Protein Coding	45	GC01P060865	6.761713	https://www.genecards.org/cgi-bin/carddisp.pl?gene=NFIA
SORCS3	Sortilin Related VPS10 Domain Containing Receptor 3	Protein Coding	37	GC10P104642	6.760242	https://www.genecards.org/cgi-bin/carddisp.pl?gene=SORCS3
SECISBP2	SECIS Binding Protein 2	Protein Coding	43	GC09P089318	6.754535	https://www.genecards.org/cgi-bin/carddisp.pl?gene=SECISBP2
ZFAS1	ZNFX1 Antisense RNA 1	RNA Gene	19	GC20P049276	6.752926	https://www.genecards.org/cgi-bin/carddisp.pl?gene=ZFAS1
YBX1	Y-Box Binding Protein 1	Protein Coding	40	GC01P042682	6.752318	https://www.genecards.org/cgi-bin/carddisp.pl?gene=YBX1
RAB3IL1	RAB3A Interacting Protein Like 1	Protein Coding	38	GC11M061897	6.750793	https://www.genecards.org/cgi-bin/carddisp.pl?gene=RAB3IL1
EHMT2	Euchromatic Histone Lysine Methyltransferase 2	Protein Coding	46	GC06M031879	6.746386	https://www.genecards.org/cgi-bin/carddisp.pl?gene=EHMT2

BRAP	BRCA1 Associated Protein	Protein Coding	42	GC12M111642	6.735047	https://www.genecards.org/cgi-bin/carddisp.pl?gene=BRAP
VEGFD	Vascular Endothelial Growth Factor D	Protein Coding	34	GC0XM015345	6.731565	https://www.genecards.org/cgi-bin/carddisp.pl?gene=VEGFD
TP53COR1	Tumor Protein P53 Pathway Corepressor 1	RNA Gene	8	GC06U903133	6.727714	https://www.genecards.org/cgi-bin/carddisp.pl?gene=TP53COR1
CDK13	Cyclin Dependent Kinase 13	Protein Coding	42	GC07P040149	6.727438	https://www.genecards.org/cgi-bin/carddisp.pl?gene=CDK13
H4C3	H4 Clustered Histone 3	Protein Coding	30	GC06P054986	6.726777	https://www.genecards.org/cgi-bin/carddisp.pl?gene=H4C3
CARTPT	CART Prepropeptide	Protein Coding	44	GC05P071719	6.725451	https://www.genecards.org/cgi-bin/carddisp.pl?gene=CARTPT
HNF4G	Hepatocyte Nuclear Factor 4 Gamma	Protein Coding	44	GC08P075407	6.725281	https://www.genecards.org/cgi-bin/carddisp.pl?gene=HNF4G
SLC25A37	Solute Carrier Family 25 Member 37	Protein Coding	39	GC08P023528	6.721336	https://www.genecards.org/cgi-bin/carddisp.pl?gene=SLC25A37
CSNK2A2	Casein Kinase 2 Alpha 2	Protein Coding	48	GC16M058157	6.711905	https://www.genecards.org/cgi-bin/carddisp.pl?gene=CSNK2A2
KIF23	Kinesin Family Member 23	Protein Coding	44	GC15P069414	6.71165	https://www.genecards.org/cgi-bin/carddisp.pl?gene=KIF23
MVD	Mevalonate Diphosphate Decarboxylase	Protein Coding	46	GC16M088651	6.707953	https://www.genecards.org/cgi-bin/carddisp.pl?gene=MVD
CSNK1A1L	Casein Kinase 1 Alpha 1 Like	Protein Coding	37	GC13M037103	6.701494	https://www.genecards.org/cgi-bin/carddisp.pl?gene=CSNK1A1L
HES1	Hes Family BHLH Transcription Factor 1	Protein Coding	44	GC03P194136	6.700509	https://www.genecards.org/cgi-bin/carddisp.pl?gene=HES1
MARCKS	Myristoylated Alanine Rich Protein Kinase C Substrate	Protein Coding	40	GC06P113857	6.700388	https://www.genecards.org/cgi-bin/carddisp.pl?gene=MARCKS
TOMM20	Translocase Of Outer Mitochondrial Membrane 20	Protein Coding	40	GC01M235109	6.699883	https://www.genecards.org/cgi-bin/carddisp.pl?gene=TOMM20
ARRB1	Arrestin Beta 1	Protein Coding	44	GC11M075261	6.697263	https://www.genecards.org/cgi-bin/carddisp.pl?gene=ARRB1
RHOD	Ras Homolog Family Member D	Protein Coding	40	GC11P067057	6.696239	https://www.genecards.org/cgi-bin/carddisp.pl?gene=RHOD
SESN2	Sestrin 2	Protein Coding	39	GC01P028270	6.693996	https://www.genecards.org/cgi-bin/carddisp.pl?gene=SESN2
FBP2	Fructose-Bisphosphatase 2	Protein Coding	44	GC09M094558	6.69334	https://www.genecards.org/cgi-bin/carddisp.pl?gene=FBP2
H4C2	H4 Clustered Histone 2	Protein Coding	31	GC06M026026	6.685936	https://www.genecards.org/cgi-bin/carddisp.pl?gene=H4C2

PHACTR1	Phosphatase And Actin Regulator 1	Protein Coding	38	GC06P012717	6.685384	https://www.genecards.org/cgi-bin/carddisp.pl?gene=PHACTR1
CDC6	Cell Division Cycle 6	Protein Coding	46	GC17P040287	6.677649	https://www.genecards.org/cgi-bin/carddisp.pl?gene=CDC6
PTBP1	Polypyrimidine Tract Binding Protein 1	Protein Coding	43	GC19P000797	6.677478	https://www.genecards.org/cgi-bin/carddisp.pl?gene=PTBP1
TXNIP	Thioredoxin Interacting Protein	Protein Coding	38	GC01M145992	6.677048	https://www.genecards.org/cgi-bin/carddisp.pl?gene=TXNIP
PBX1	PBX Homeobox 1	Protein Coding	50	GC01P164524	6.676983	https://www.genecards.org/cgi-bin/carddisp.pl?gene=PBX1
CHEK1	Checkpoint Kinase 1	Protein Coding	50	GC11P125625	6.676565	https://www.genecards.org/cgi-bin/carddisp.pl?gene=CHEK1
SEPTIN5	Septin 5	Protein Coding	32	GC22P019714	6.67613	https://www.genecards.org/cgi-bin/carddisp.pl?gene=SEPTIN5
RSPO1	R-Spondin 1	Protein Coding	44	GC01M037612	6.67139	https://www.genecards.org/cgi-bin/carddisp.pl?gene=RSPO1
DYRK1B	Dual Specificity Tyrosine Phosphorylation Regulated Kinase 1B	Protein Coding	47	GC19M039825	6.666582	https://www.genecards.org/cgi-bin/carddisp.pl?gene=DYRK1B
NECTIN1	Nectin Cell Adhesion Molecule 1	Protein Coding	38	GC11M119624	6.663525	https://www.genecards.org/cgi-bin/carddisp.pl?gene=NECTIN1
MIR103A2	MicroRNA 103a-2	RNA Gene	18	GC20P003917	6.662079	https://www.genecards.org/cgi-bin/carddisp.pl?gene=MIR103A2
SIPA1L2	Signal Induced Proliferation Associated 1 Like 2	Protein Coding	38	GC01M232397	6.658369	https://www.genecards.org/cgi-bin/carddisp.pl?gene=SIPA1L2
CLDN3	Claudin 3	Protein Coding	41	GC07M073768	6.655747	https://www.genecards.org/cgi-bin/carddisp.pl?gene=CLDN3
MIR372	MicroRNA 372	RNA Gene	16	GC19P056407	6.654529	https://www.genecards.org/cgi-bin/carddisp.pl?gene=MIR372
SLC2A4RG	SLC2A4 Regulator	Protein Coding	37	GC20P063739	6.652842	https://www.genecards.org/cgi-bin/carddisp.pl?gene=SLC2A4RG
ADTRP	Androgen Dependent TFPI Regulating Protein	Protein Coding	29	GC06M011712	6.650177	https://www.genecards.org/cgi-bin/carddisp.pl?gene=ADTRP
PLAGL1	PLAG1 Like Zinc Finger 1	Protein Coding	44	GC06M143940	6.649423	https://www.genecards.org/cgi-bin/carddisp.pl?gene=PLAGL1
UGT2B28	UDP Glucuronosyltransferase Family 2 Member B28	Protein Coding	40	GC04P069280	6.649005	https://www.genecards.org/cgi-bin/carddisp.pl?gene=UGT2B28
VEGFB	Vascular Endothelial Growth Factor B	Protein Coding	45	GC11P064234	6.646336	https://www.genecards.org/cgi-bin/carddisp.pl?gene=VEGFB

PNLIPRP2	Pancreatic Lipase Related Protein 2 (Gene/Pseudogene)	Protein Coding	37	GC10P116620	6.646216	https://www.genecards.org/cgi-bin/carddisp.pl?gene=PNLIPRP2
SDC2	Syndecan 2	Protein Coding	45	GC08P096495	6.644638	https://www.genecards.org/cgi-bin/carddisp.pl?gene=SDC2
SFN	Stratifin	Protein Coding	47	GC01P026904	6.642867	https://www.genecards.org/cgi-bin/carddisp.pl?gene=SFN
PDPK1	3-Phosphoinositide Dependent Protein Kinase 1	Protein Coding	50	GC16P002537	6.630094	https://www.genecards.org/cgi-bin/carddisp.pl?gene=PDPK1
PYCARD	PYD And CARD Domain Containing	Protein Coding	43	GC16M031201	6.626009	https://www.genecards.org/cgi-bin/carddisp.pl?gene=PYCARD
UFC1	Ubiquitin-Fold Modifier Conjugating Enzyme 1	Protein Coding	38	GC01P161177	6.619278	https://www.genecards.org/cgi-bin/carddisp.pl?gene=UFC1
CDKAL1	CDK5 Regulatory Subunit Associated Protein 1 Like 1	Protein Coding	40	GC06P020534	6.618275	https://www.genecards.org/cgi-bin/carddisp.pl?gene=CDKAL1
INTS2	Integrator Complex Subunit 2	Protein Coding	35	GC17M061865	6.614825	https://www.genecards.org/cgi-bin/carddisp.pl?gene=INTS2
PARD3	Par-3 Family Cell Polarity Regulator	Protein Coding	43	GC10M034110	6.608632	https://www.genecards.org/cgi-bin/carddisp.pl?gene=PARD3
CEP135	Centrosomal Protein 135	Protein Coding	39	GC04P055948	6.60545	https://www.genecards.org/cgi-bin/carddisp.pl?gene=CEP135
DCK	Deoxycytidine Kinase	Protein Coding	45	GC04P070992	6.604548	https://www.genecards.org/cgi-bin/carddisp.pl?gene=DCK
ASPH	Aspartate Beta-Hydroxylase	Protein Coding	43	GC08M061500	6.598381	https://www.genecards.org/cgi-bin/carddisp.pl?gene=ASPH
LPXN	Leupaxin	Protein Coding	41	GC11M069097	6.595394	https://www.genecards.org/cgi-bin/carddisp.pl?gene=LPXN
PSMC1	Proteasome 26S Subunit, ATPase 1	Protein Coding	43	GC14P090256	6.595159	https://www.genecards.org/cgi-bin/carddisp.pl?gene=PSMC1
RNF146	Ring Finger Protein 146	Protein Coding	37	GC06P127266	6.594392	https://www.genecards.org/cgi-bin/carddisp.pl?gene=RNF146
DHPS	Deoxyhypusine Synthase	Protein Coding	43	GC19M013051	6.587067	https://www.genecards.org/cgi-bin/carddisp.pl?gene=DHPS
SARS1	Seryl-TRNA Synthetase 1	Protein Coding	36	GC01P109214	6.586814	https://www.genecards.org/cgi-bin/carddisp.pl?gene=SARS1
LMOD3	Leiomodin 3	Protein Coding	39	GC03M069156	6.586119	https://www.genecards.org/cgi-bin/carddisp.pl?gene=LMOD3
VAMP1	Vesicle Associated Membrane Protein 1	Protein Coding	46	GC12M006462	6.585022	https://www.genecards.org/cgi-bin/carddisp.pl?gene=VAMP1

CRNDE	Colorectal Neoplasia Differentially Expressed	RNA Gene	20	GC16M054845	6.58463	https://www.genecards.org/cgi-bin/carddisp.pl?gene=CRNDE
TBXA2R	Thromboxane A2 Receptor	Protein Coding	49	GC19M003594	6.579034	https://www.genecards.org/cgi-bin/carddisp.pl?gene=TBXA2R
HOXA11-AS	HOXA11 Antisense RNA	RNA Gene	20	GC07P027184	6.57717	https://www.genecards.org/cgi-bin/carddisp.pl?gene=HOXA11-AS
MIR661	MicroRNA 661	RNA Gene	18	GC08M143945	6.575136	https://www.genecards.org/cgi-bin/carddisp.pl?gene=MIR661
SPRY4	Sprouty RTK Signaling Antagonist 4	Protein Coding	45	GC05M142310	6.573835	https://www.genecards.org/cgi-bin/carddisp.pl?gene=SPRY4
SAA2	Serum Amyloid A2	Protein Coding	36	GC11M018238	6.571986	https://www.genecards.org/cgi-bin/carddisp.pl?gene=SAA2
ANXA6	Annexin A6	Protein Coding	43	GC05M151100	6.569563	https://www.genecards.org/cgi-bin/carddisp.pl?gene=ANXA6
SLC25A10	Solute Carrier Family 25 Member 10	Protein Coding	43	GC17P081712	6.568927	https://www.genecards.org/cgi-bin/carddisp.pl?gene=SLC25A10
PCSK2	Proprotein Convertase Subtilisin/Kexin Type 2	Protein Coding	41	GC20P017226	6.566692	https://www.genecards.org/cgi-bin/carddisp.pl?gene=PCSK2
TIMELESS	Timeless Circadian Regulator	Protein Coding	39	GC12M056416	6.565112	https://www.genecards.org/cgi-bin/carddisp.pl?gene=TIMELESS
ARID4A	AT-Rich Interaction Domain 4A	Protein Coding	38	GC14P058298	6.563344	https://www.genecards.org/cgi-bin/carddisp.pl?gene=ARID4A
CNTN2	Contactin 2	Protein Coding	46	GC01P205043	6.560995	https://www.genecards.org/cgi-bin/carddisp.pl?gene=CNTN2
ATF3	Activating Transcription Factor 3	Protein Coding	45	GC01P212565	6.560956	https://www.genecards.org/cgi-bin/carddisp.pl?gene=ATF3
TNFRSF9	TNF Receptor Superfamily Member 9	Protein Coding	45	GC01M007915	6.560941	https://www.genecards.org/cgi-bin/carddisp.pl?gene=TNFRSF9
ARCN1	Archain 1	Protein Coding	42	GC11P118572	6.557966	https://www.genecards.org/cgi-bin/carddisp.pl?gene=ARCN1
SLC7A2	Solute Carrier Family 7 Member 2	Protein Coding	43	GC08P017497	6.557402	https://www.genecards.org/cgi-bin/carddisp.pl?gene=SLC7A2
PLA2G2E	Phospholipase A2 Group IIE	Protein Coding	39	GC01M019920	6.548486	https://www.genecards.org/cgi-bin/carddisp.pl?gene=PLA2G2E
TRRAP	Transformation/Transcription Domain Associated Protein	Protein Coding	46	GC07P098877	6.5484	https://www.genecards.org/cgi-bin/carddisp.pl?gene=TRRAP
KDM6B	Lysine Demethylase 6B	Protein Coding	44	GC17P007834	6.542434	https://www.genecards.org/cgi-bin/carddisp.pl?gene=KDM6B
PKP1	Plakophilin 1	Protein Coding	42	GC01P201283	6.539439	https://www.genecards.org/cgi-bin/carddisp.pl?gene=PKP1

FGF5	Fibroblast Growth Factor 5	Protein Coding	45	GC04P080266	6.534393	https://www.genecards.org/cgi-bin/carddisp.pl?gene=FGF5
LAMA4	Laminin Subunit Alpha 4	Protein Coding	45	GC06M112107	6.533856	https://www.genecards.org/cgi-bin/carddisp.pl?gene=LAMA4
KAT2B	Lysine Acetyltransferase 2B	Protein Coding	48	GC03P020043	6.530809	https://www.genecards.org/cgi-bin/carddisp.pl?gene=KAT2B
RMC1	Regulator Of MON1-CCZ1	Protein Coding	25	GC18P023504	6.528017	https://www.genecards.org/cgi-bin/carddisp.pl?gene=RMC1
NUP188	Nucleoporin 188	Protein Coding	37	GC09P128947	6.526526	https://www.genecards.org/cgi-bin/carddisp.pl?gene=NUP188
ADAMTS9	ADAM Metallopeptidase With Thrombospondin Type 1 Motif 9	Protein Coding	38	GC03M064501	6.520804	https://www.genecards.org/cgi-bin/carddisp.pl?gene=ADAMTS9
CCAT2	Colon Cancer Associated Transcript 2	RNA Gene	13	GC08P127400	6.512988	https://www.genecards.org/cgi-bin/carddisp.pl?gene=CCAT2
OXTR	Oxytocin Receptor	Protein Coding	47	GC03M008767	6.510406	https://www.genecards.org/cgi-bin/carddisp.pl?gene=OXTR
SLC1A4	Solute Carrier Family 1 Member 4	Protein Coding	45	GC02P064988	6.50598	https://www.genecards.org/cgi-bin/carddisp.pl?gene=SLC1A4
PAK1	P21 (RAC1) Activated Kinase 1	Protein Coding	49	GC11M077321	6.505352	https://www.genecards.org/cgi-bin/carddisp.pl?gene=PAK1
GGT2	Gamma-Glutamyltransferase 2	Protein Coding	31	GC22M021207	6.501858	https://www.genecards.org/cgi-bin/carddisp.pl?gene=GGT2
ENPEP	Glutamyl Aminopeptidase	Protein Coding	45	GC04P110365	6.499918	https://www.genecards.org/cgi-bin/carddisp.pl?gene=ENPEP
BCL2A1	BCL2 Related Protein A1	Protein Coding	41	GC15M079961	6.490554	https://www.genecards.org/cgi-bin/carddisp.pl?gene=BCL2A1
PPL	Periplakin	Protein Coding	41	GC16M004872	6.489831	https://www.genecards.org/cgi-bin/carddisp.pl?gene=PPL
RDH10	Retinol Dehydrogenase 10	Protein Coding	40	GC08P073294	6.48808	https://www.genecards.org/cgi-bin/carddisp.pl?gene=RDH10
SNHG20	Small Nucleolar RNA Host Gene 20	RNA Gene	17	GC17P077087	6.486632	https://www.genecards.org/cgi-bin/carddisp.pl?gene=SNHG20
DARS1	Aspartyl-TRNA Synthetase 1	Protein Coding	37	GC02M135905	6.485133	https://www.genecards.org/cgi-bin/carddisp.pl?gene=DARS1
RPTOR	Regulatory Associated Protein Of MTOR Complex 1	Protein Coding	44	GC17P080544	6.483601	https://www.genecards.org/cgi-bin/carddisp.pl?gene=RPTOR
THBS4	Thrombospondin 4	Protein Coding	44	GC05P079991	6.47765	https://www.genecards.org/cgi-bin/carddisp.pl?gene=THBS4

FAM126A	Family With Sequence Similarity 126 Member A	Protein Coding	37	GC07M022889	6.476423	https://www.genecards.org/cgi-bin/carddisp.pl?gene=FAM126A
ATP5F1C	ATP Synthase F1 Subunit Gamma	Protein Coding	33	GC10P007789	6.472447	https://www.genecards.org/cgi-bin/carddisp.pl?gene=ATP5F1C
NELFA	Negative Elongation Factor Complex Member A	Protein Coding	37	GC04M001985	6.46648	https://www.genecards.org/cgi-bin/carddisp.pl?gene=NELFA
RBFOX3	RNA Binding Fox-1 Homolog 3	Protein Coding	36	GC17M079089	6.465144	https://www.genecards.org/cgi-bin/carddisp.pl?gene=RBFOX3
PPP1R3B	Protein Phosphatase 1 Regulatory Subunit 3B	Protein Coding	37	GC08M009136	6.460268	https://www.genecards.org/cgi-bin/carddisp.pl?gene=PPP1R3B
NTNG1	Netrin G1	Protein Coding	41	GC01P107140	6.458458	https://www.genecards.org/cgi-bin/carddisp.pl?gene=NTNG1
TSEN2	TRNA Splicing Endonuclease Subunit 2	Protein Coding	41	GC03P012484	6.457306	https://www.genecards.org/cgi-bin/carddisp.pl?gene=TSEN2
ZHX2	Zinc Fingers And Homeoboxes 2	Protein Coding	39	GC08P122781	6.454482	https://www.genecards.org/cgi-bin/carddisp.pl?gene=ZHX2
CAMK4	Calcium/Calmodulin Dependent Protein Kinase IV	Protein Coding	47	GC05P111223	6.452702	https://www.genecards.org/cgi-bin/carddisp.pl?gene=CAMK4
GJC1	Gap Junction Protein Gamma 1	Protein Coding	43	GC17M044800	6.452277	https://www.genecards.org/cgi-bin/carddisp.pl?gene=GJC1
AOPEP	Aminopeptidase O (Putative)	Protein Coding	31	GC09P094728	6.451938	https://www.genecards.org/cgi-bin/carddisp.pl?gene=AOPEP
PIGN	Phosphatidylinositol Glycan Anchor Biosynthesis Class N	Protein Coding	41	GC18M061905	6.449343	https://www.genecards.org/cgi-bin/carddisp.pl?gene=PIGN
RBP2	Retinol Binding Protein 2	Protein Coding	39	GC03M139452	6.445167	https://www.genecards.org/cgi-bin/carddisp.pl?gene=RBP2
MLST8	MTOR Associated Protein, LST8 Homolog	Protein Coding	43	GC16P002204	6.442444	https://www.genecards.org/cgi-bin/carddisp.pl?gene=MLST8
UBE2D1	Ubiquitin Conjugating Enzyme E2 D1	Protein Coding	46	GC10P058334	6.441568	https://www.genecards.org/cgi-bin/carddisp.pl?gene=UBE2D1
PDZK1	PDZ Domain Containing 1	Protein Coding	42	GC01M145670	6.441489	https://www.genecards.org/cgi-bin/carddisp.pl?gene=PDZK1
TPD52	Tumor Protein D52	Protein Coding	42	GC08M079920	6.439872	https://www.genecards.org/cgi-bin/carddisp.pl?gene=TPD52
DPH1	Diphthamide Biosynthesis 1	Protein Coding	41	GC17P002030	6.437938	https://www.genecards.org/cgi-bin/carddisp.pl?gene=DPH1

XYLB	Xylulokinase	Protein Coding	40	GC03P038363	6.437343	https://www.genecards.org/cgi-bin/carddisp.pl?gene=XYLB
DAB2	DAB Adaptor Protein 2	Protein Coding	44	GC05M039371	6.435457	https://www.genecards.org/cgi-bin/carddisp.pl?gene=DAB2
BAIAP2L1	BAR/IMD Domain Containing Adaptor Protein 2 Like 1	Protein Coding	40	GC07M098294	6.43115	https://www.genecards.org/cgi-bin/carddisp.pl?gene=BAIAP2L1
CYP2F1	Cytochrome P450 Family 2 Subfamily F Member 1	Protein Coding	43	GC19P041114	6.4306	https://www.genecards.org/cgi-bin/carddisp.pl?gene=CYP2F1
ZMIZ1	Zinc Finger MIZ-Type Containing 1	Protein Coding	41	GC10P079068	6.430305	https://www.genecards.org/cgi-bin/carddisp.pl?gene=ZMIZ1
MPC1	Mitochondrial Pyruvate Carrier 1	Protein Coding	43	GC06M166364	6.426732	https://www.genecards.org/cgi-bin/carddisp.pl?gene=MPC1
TPM4	Tropomyosin 4	Protein Coding	41	GC19P026610	6.424023	https://www.genecards.org/cgi-bin/carddisp.pl?gene=TPM4
RBM10	RNA Binding Motif Protein 10	Protein Coding	41	GC0XP047206	6.420727	https://www.genecards.org/cgi-bin/carddisp.pl?gene=RBM10
KDM5B	Lysine Demethylase 5B	Protein Coding	44	GC01M202696	6.419094	https://www.genecards.org/cgi-bin/carddisp.pl?gene=KDM5B
PTN	Pleiotrophin	Protein Coding	43	GC07M137227	6.416771	https://www.genecards.org/cgi-bin/carddisp.pl?gene=PTN
IQGAP2	IQ Motif Containing GTPase Activating Protein 2	Protein Coding	43	GC05P076403	6.412855	https://www.genecards.org/cgi-bin/carddisp.pl?gene=IQGAP2
ST14	ST14 Transmembrane Serine Protease Matriptase	Protein Coding	47	GC11P130159	6.41197	https://www.genecards.org/cgi-bin/carddisp.pl?gene=ST14
SRSF6	Serine And Arginine Rich Splicing Factor 6	Protein Coding	40	GC20P043457	6.410023	https://www.genecards.org/cgi-bin/carddisp.pl?gene=SRSF6
FAP	Fibroblast Activation Protein Alpha	Protein Coding	43	GC02M162170	6.409836	https://www.genecards.org/cgi-bin/carddisp.pl?gene=FAP
P3H2	Prolyl 3-Hydroxylase 2	Protein Coding	37	GC03M189956	6.409812	https://www.genecards.org/cgi-bin/carddisp.pl?gene=P3H2
SHARPIN	SHANK Associated RH Domain Interactor	Protein Coding	41	GC08M144098	6.40668	https://www.genecards.org/cgi-bin/carddisp.pl?gene=SHARPIN
H4C11	H4 Clustered Histone 11	Protein Coding	30	GC06P055048	6.40627	https://www.genecards.org/cgi-bin/carddisp.pl?gene=H4C11

FRAS1	Fraser Extracellular Matrix Complex Subunit 1	Protein Coding	38	GC04P078056	6.403532	https://www.genecards.org/cgi-bin/carddisp.pl?gene=FRAS1
SLC7A5	Solute Carrier Family 7 Member 5	Protein Coding	45	GC16M087830	6.400654	https://www.genecards.org/cgi-bin/carddisp.pl?gene=SLC7A5
H4C8	H4 Clustered Histone 8	Protein Coding	30	GC06M047225	6.39635	https://www.genecards.org/cgi-bin/carddisp.pl?gene=H4C8
TET1	Tet Methylcytosine Dioxygenase 1	Protein Coding	38	GC10P068560	6.394946	https://www.genecards.org/cgi-bin/carddisp.pl?gene=TET1
NDRG2	NDRG Family Member 2	Protein Coding	39	GC14M021016	6.393259	https://www.genecards.org/cgi-bin/carddisp.pl?gene=NDRG2
SLC31A1	Solute Carrier Family 31 Member 1	Protein Coding	43	GC09P113221	6.392159	https://www.genecards.org/cgi-bin/carddisp.pl?gene=SLC31A1
LAMP3	Lysosomal Associated Membrane Protein 3	Protein Coding	40	GC03M183122	6.389036	https://www.genecards.org/cgi-bin/carddisp.pl?gene=LAMP3
FITM2	Fat Storage Inducing Transmembrane Protein 2	Protein Coding	36	GC20M044302	6.386168	https://www.genecards.org/cgi-bin/carddisp.pl?gene=FITM2
RING1	Ring Finger Protein 1	Protein Coding	42	GC06P033208	6.384512	https://www.genecards.org/cgi-bin/carddisp.pl?gene=RING1
H4C4	H4 Clustered Histone 4	Protein Coding	27	GC06M047223	6.377773	https://www.genecards.org/cgi-bin/carddisp.pl?gene=H4C4
SIRT2	Sirtuin 2	Protein Coding	48	GC19M038878	6.374105	https://www.genecards.org/cgi-bin/carddisp.pl?gene=SIRT2
UBE4B	Ubiquitination Factor E4B	Protein Coding	41	GC01P010032	6.374067	https://www.genecards.org/cgi-bin/carddisp.pl?gene=UBE4B
SNHG15	Small Nucleolar RNA Host Gene 15	RNA Gene	19	GC07M044983	6.373295	https://www.genecards.org/cgi-bin/carddisp.pl?gene=SNHG15
NAA40	N-Alpha-Acetyltransferase 40, NatD Catalytic Subunit	Protein Coding	34	GC11P063938	6.372422	https://www.genecards.org/cgi-bin/carddisp.pl?gene=NAA40
STIL	STIL Centriolar Assembly Protein	Protein Coding	42	GC01M047250	6.37236	https://www.genecards.org/cgi-bin/carddisp.pl?gene=STIL
MIR134	MicroRNA 134	RNA Gene	19	GC14P106302	6.368529	https://www.genecards.org/cgi-bin/carddisp.pl?gene=MIR134
SOCS2	Suppressor Of Cytokine Signaling 2	Protein Coding	45	GC12P093569	6.367354	https://www.genecards.org/cgi-bin/carddisp.pl?gene=SOCS2
IKBKE	Inhibitor Of Nuclear Factor Kappa B Kinase Subunit Epsilon	Protein Coding	46	GC01P206470	6.35701	https://www.genecards.org/cgi-bin/carddisp.pl?gene=IKBKE

PPP3R1	Protein Phosphatase 3 Regulatory Subunit B, Alpha	Protein Coding	46	GC02M068143	6.345759	https://www.genecards.org/cgi-bin/carddisp.pl?gene=PPP3R1
FMN1	Formin 1	Protein Coding	39	GC15M032765	6.339075	https://www.genecards.org/cgi-bin/carddisp.pl?gene=FMN1
FPR2	Formyl Peptide Receptor 2	Protein Coding	47	GC19P051752	6.339007	https://www.genecards.org/cgi-bin/carddisp.pl?gene=FPR2
KISS1R	KISS1 Receptor	Protein Coding	45	GC19P000917	6.336868	https://www.genecards.org/cgi-bin/carddisp.pl?gene=KISS1R
GPR84	G Protein-Coupled Receptor 84	Protein Coding	40	GC12M054362	6.334286	https://www.genecards.org/cgi-bin/carddisp.pl?gene=GPR84
CXCL16	C-X-C Motif Chemokine Ligand 16	Protein Coding	40	GC17M004733	6.331749	https://www.genecards.org/cgi-bin/carddisp.pl?gene=CXCL16
SFPQ	Splicing Factor Proline And Glutamine Rich	Protein Coding	44	GC01M035176	6.326203	https://www.genecards.org/cgi-bin/carddisp.pl?gene=SFPQ
APLNR	Apelin Receptor	Protein Coding	44	GC11M057233	6.323127	https://www.genecards.org/cgi-bin/carddisp.pl?gene=APLNR
FBXO11	F-Box Protein 11	Protein Coding	43	GC02M047789	6.320556	https://www.genecards.org/cgi-bin/carddisp.pl?gene=FBXO11
PTENP1	Phosphatase And Tensin Homolog Pseudogene 1	Pseudogene	20	GC09M033673	6.317821	https://www.genecards.org/cgi-bin/carddisp.pl?gene=PTENP1
HLA-DOA	Major Histocompatibility Complex, Class II, DO Alpha	Protein Coding	41	GC06M033004	6.314027	https://www.genecards.org/cgi-bin/carddisp.pl?gene=HLA-DOA
MVP	Major Vault Protein	Protein Coding	41	GC16P032377	6.313807	https://www.genecards.org/cgi-bin/carddisp.pl?gene=MVP
ESRRA	Estrogen Related Receptor Alpha	Protein Coding	48	GC11P064305	6.30768	https://www.genecards.org/cgi-bin/carddisp.pl?gene=ESRRA
DAB1	DAB Adaptor Protein 1	Protein Coding	44	GC01M056994	6.304649	https://www.genecards.org/cgi-bin/carddisp.pl?gene=DAB1
AP2B1	Adaptor Related Protein Complex 2 Subunit Beta 1	Protein Coding	43	GC17P035578	6.303818	https://www.genecards.org/cgi-bin/carddisp.pl?gene=AP2B1
MIR124-2	MicroRNA 124-2	RNA Gene	19	GC08P064379	6.303351	https://www.genecards.org/cgi-bin/carddisp.pl?gene=MIR124-2
TOP2B	DNA Topoisomerase II Beta	Protein Coding	47	GC03M025598	6.302116	https://www.genecards.org/cgi-bin/carddisp.pl?gene=TOP2B
POU2F1	POU Class 2 Homeobox 1	Protein Coding	45	GC01P167190	6.300186	https://www.genecards.org/cgi-bin/carddisp.pl?gene=POU2F1
RPS18	Ribosomal Protein S18	Protein Coding	41	GC06P055262	6.299579	https://www.genecards.org/cgi-bin/carddisp.pl?gene=RPS18

RARG	Retinoic Acid Receptor Gamma	Protein Coding	48	GC12M053210	6.294356	https://www.genecards.org/cgi-bin/carddisp.pl?gene=RARG
CASC2	Cancer Susceptibility 2	RNA Gene	24	GC10P118046	6.291429	https://www.genecards.org/cgi-bin/carddisp.pl?gene=CASC2
GPER1	G Protein-Coupled Estrogen Receptor 1	Protein Coding	38	GC07P001368	6.287693	https://www.genecards.org/cgi-bin/carddisp.pl?gene=GPER1
CCL19	C-C Motif Chemokine Ligand 19	Protein Coding	42	GC09M034692	6.28407	https://www.genecards.org/cgi-bin/carddisp.pl?gene=CCL19
EMG1	EMG1 N1-Specific Pseudouridine Methyltransferase	Protein Coding	41	GC12P006970	6.281708	https://www.genecards.org/cgi-bin/carddisp.pl?gene=EMG1
MAP2K7	Mitogen-Activated Protein Kinase Kinase 7	Protein Coding	47	GC19P007903	6.277817	https://www.genecards.org/cgi-bin/carddisp.pl?gene=MAP2K7
YWHAB	Tyrosine 3-Monooxygenase/Tryptophan 5-Monooxygenase Activation Protein Beta	Protein Coding	49	GC20P044885	6.274555	https://www.genecards.org/cgi-bin/carddisp.pl?gene=YWHAB
LTB4R	Leukotriene B4 Receptor	Protein Coding	45	GC14P024311	6.272876	https://www.genecards.org/cgi-bin/carddisp.pl?gene=LTB4R
EIF6	Eukaryotic Translation Initiation Factor 6	Protein Coding	41	GC20M035278	6.272304	https://www.genecards.org/cgi-bin/carddisp.pl?gene=EIF6
MCM2	Minichromosome Maintenance Complex Component 2	Protein Coding	48	GC03P127598	6.268583	https://www.genecards.org/cgi-bin/carddisp.pl?gene=MCM2
ABCC11	ATP Binding Cassette Subfamily C Member 11	Protein Coding	43	GC16M048166	6.26734	https://www.genecards.org/cgi-bin/carddisp.pl?gene=ABCC11
ATXN2L	Ataxin 2 Like	Protein Coding	38	GC16P032257	6.266768	https://www.genecards.org/cgi-bin/carddisp.pl?gene=ATXN2L
RHOB	Ras Homolog Family Member B	Protein Coding	44	GC02P020447	6.265263	https://www.genecards.org/cgi-bin/carddisp.pl?gene=RHOB
ABHD6	Abhydrolase Domain Containing 6, Acylglycerol Lipase	Protein Coding	39	GC03P058238	6.265128	https://www.genecards.org/cgi-bin/carddisp.pl?gene=ABHD6
MDC1	Mediator Of DNA Damage Checkpoint 1	Protein Coding	41	GC06M046797	6.262409	https://www.genecards.org/cgi-bin/carddisp.pl?gene=MDC1

PTTG1	PTTG1 Regulator Of Sister Chromatid Separation, Securin	Protein Coding	44	GC05P160422	6.259799	https://www.genecards.org/cgi-bin/carddisp.pl?gene=PTTG1
TEC	Tec Protein Tyrosine Kinase	Protein Coding	46	GC04M048137	6.247668	https://www.genecards.org/cgi-bin/carddisp.pl?gene=TEC
NTN1	Netrin 1	Protein Coding	45	GC17P009021	6.244952	https://www.genecards.org/cgi-bin/carddisp.pl?gene=NTN1
POLR2L	RNA Polymerase II, I And III Subunit L	Protein Coding	41	GC11M001144	6.234688	https://www.genecards.org/cgi-bin/carddisp.pl?gene=POLR2L
FLVCR2	FLVCR Heme Transporter 2	Protein Coding	42	GC14P075578	6.234234	https://www.genecards.org/cgi-bin/carddisp.pl?gene=FLVCR2
ARG2	Arginase 2	Protein Coding	46	GC14P067619	6.23372	https://www.genecards.org/cgi-bin/carddisp.pl?gene=ARG2
VDAC3	Voltage Dependent Anion Channel 3	Protein Coding	44	GC08P042392	6.233534	https://www.genecards.org/cgi-bin/carddisp.pl?gene=VDAC3
SLC25A28	Solute Carrier Family 25 Member 28	Protein Coding	38	GC10M099610	6.23326	https://www.genecards.org/cgi-bin/carddisp.pl?gene=SLC25A28
MED1	Mediator Complex Subunit 1	Protein Coding	42	GC17M039404	6.227978	https://www.genecards.org/cgi-bin/carddisp.pl?gene=MED1
NUDT6	Nudix Hydrolase 6	Protein Coding	38	GC04M122888	6.227676	https://www.genecards.org/cgi-bin/carddisp.pl?gene=NUDT6
H4C9	H4 Clustered Histone 9	Protein Coding	30	GC06P055592	6.225471	https://www.genecards.org/cgi-bin/carddisp.pl?gene=H4C9
TPPP3	Tubulin Polymerization Promoting Protein Family Member 3	Protein Coding	38	GC16M067389	6.223899	https://www.genecards.org/cgi-bin/carddisp.pl?gene=TPPP3
G3BP1	G3BP Stress Granule Assembly Factor 1	Protein Coding	41	GC05P151771	6.222131	https://www.genecards.org/cgi-bin/carddisp.pl?gene=G3BP1
BCL3	BCL3 Transcription Coactivator	Protein Coding	41	GC19P044747	6.218973	https://www.genecards.org/cgi-bin/carddisp.pl?gene=BCL3
MIR33B	MicroRNA 33b	RNA Gene	18	GC17M017813	6.212285	https://www.genecards.org/cgi-bin/carddisp.pl?gene=MIR33B
PSMD3	Proteasome 26S Subunit, Non-ATPase 3	Protein Coding	43	GC17P039980	6.210496	https://www.genecards.org/cgi-bin/carddisp.pl?gene=PSMD3
HAO2	Hydroxyacid Oxidase 2	Protein Coding	38	GC01P119368	6.205287	https://www.genecards.org/cgi-bin/carddisp.pl?gene=HAO2
NUDC	Nuclear Distribution C, Dynein Complex Regulator	Protein Coding	43	GC01P026925	6.203939	https://www.genecards.org/cgi-bin/carddisp.pl?gene=NUDC

PLCB3	Phospholipase C Beta 3	Protein Coding	49	GC11P064251	6.202496	https://www.genecards.org/cgi-bin/carddisp.pl?gene=PLCB3
CRKL	CRK Like Proto-Oncogene, Adaptor Protein	Protein Coding	47	GC22P020917	6.201339	https://www.genecards.org/cgi-bin/carddisp.pl?gene=CRKL
TRIB3	Tribbles Pseudokinase 3	Protein Coding	44	GC20P000361	6.200017	https://www.genecards.org/cgi-bin/carddisp.pl?gene=TRIB3
S1PR1	Sphingosine-1-Phosphate Receptor 1	Protein Coding	45	GC01P101236	6.194642	https://www.genecards.org/cgi-bin/carddisp.pl?gene=S1PR1
LOC107548112	REN Promoter And Enhancer Region	Biological Region	2	GC01P204162	6.193439	https://www.genecards.org/cgi-bin/carddisp.pl?gene=LOC107548112
FLOT2	Flotillin 2	Protein Coding	42	GC17M031085	6.1912	https://www.genecards.org/cgi-bin/carddisp.pl?gene=FLOT2
GLRA2	Glycine Receptor Alpha 2	Protein Coding	45	GC0XP014529	6.188403	https://www.genecards.org/cgi-bin/carddisp.pl?gene=GLRA2
ATIC	5-Aminoimidazole-4-Carboxamide Ribonucleotide Formyltransferase/IMP Cyclohydrolase	Protein Coding	47	GC02P215311	6.184664	https://www.genecards.org/cgi-bin/carddisp.pl?gene=ATIC
SLC38A8	Solute Carrier Family 38 Member 8	Protein Coding	37	GC16M084009	6.182121	https://www.genecards.org/cgi-bin/carddisp.pl?gene=SLC38A8
CTHRC1	Collagen Triple Helix Repeat Containing 1	Protein Coding	41	GC08P103371	6.182034	https://www.genecards.org/cgi-bin/carddisp.pl?gene=CTHRC1
MACF1	Microtubule Actin Crosslinking Factor 1	Protein Coding	41	GC01P039082	6.179041	https://www.genecards.org/cgi-bin/carddisp.pl?gene=MACF1
MAML2	Mastermind Like Transcriptional Coactivator 2	Protein Coding	38	GC11M095976	6.160441	https://www.genecards.org/cgi-bin/carddisp.pl?gene=MAML2
SLC25A27	Solute Carrier Family 25 Member 27	Protein Coding	39	GC06P046652	6.158172	https://www.genecards.org/cgi-bin/carddisp.pl?gene=SLC25A27
COPB2	COPI Coat Complex Subunit Beta 2	Protein Coding	45	GC03M139355	6.154596	https://www.genecards.org/cgi-bin/carddisp.pl?gene=COPB2
PIGV	Phosphatidylinositol Glycan Anchor Biosynthesis Class V	Protein Coding	43	GC01P026787	6.149278	https://www.genecards.org/cgi-bin/carddisp.pl?gene=PIGV
RPL7A	Ribosomal Protein L7a	Protein Coding	43	GC09P133348	6.145533	https://www.genecards.org/cgi-bin/carddisp.pl?gene=RPL7A
YEATS2	YEATS Domain Containing 2	Protein Coding	37	GC03P183698	6.14296	https://www.genecards.org/cgi-bin/carddisp.pl?gene=YEATS2

ZFP57	ZFP57 Zinc Finger Protein	Protein Coding	40	GC06M029672	6.140587	https://www.genecards.org/cgi-bin/carddisp.pl?gene=ZFP57
PDGFC	Platelet Derived Growth Factor C	Protein Coding	44	GC04M156760	6.137277	https://www.genecards.org/cgi-bin/carddisp.pl?gene=PDGFC
KLF5	Kruppel Like Factor 5	Protein Coding	45	GC13P073054	6.136822	https://www.genecards.org/cgi-bin/carddisp.pl?gene=KLF5
KERA	Keratocan	Protein Coding	42	GC12M091050	6.135474	https://www.genecards.org/cgi-bin/carddisp.pl?gene=KERA
CTRL	Chymotrypsin Like	Protein Coding	40	GC16M067927	6.135232	https://www.genecards.org/cgi-bin/carddisp.pl?gene=CTRL
CRK	CRK Proto-Oncogene, Adaptor Protein	Protein Coding	46	GC17M001420	6.130989	https://www.genecards.org/cgi-bin/carddisp.pl?gene=CRK
H4C13	H4 Clustered Histone 13	Protein Coding	29	GC06M047221	6.130874	https://www.genecards.org/cgi-bin/carddisp.pl?gene=H4C13
ROCK1	Rho Associated Coiled-Coil Containing Protein Kinase 1	Protein Coding	51	GC18M020946	6.129965	https://www.genecards.org/cgi-bin/carddisp.pl?gene=ROCK1
H4C6	H4 Clustered Histone 6	Protein Coding	29	GC06P055591	6.127167	https://www.genecards.org/cgi-bin/carddisp.pl?gene=H4C6
HKDC1	Hexokinase Domain Containing 1	Protein Coding	41	GC10P069220	6.124722	https://www.genecards.org/cgi-bin/carddisp.pl?gene=HKDC1
CENPE	Centromere Protein E	Protein Coding	45	GC04M103105	6.124363	https://www.genecards.org/cgi-bin/carddisp.pl?gene=CENPE
CERK	Ceramide Kinase	Protein Coding	43	GC22M046684	6.123432	https://www.genecards.org/cgi-bin/carddisp.pl?gene=CERK
GCSH	Glycine Cleavage System Protein H	Protein Coding	44	GC16M081081	6.122847	https://www.genecards.org/cgi-bin/carddisp.pl?gene=GCSH
MIR520C	MicroRNA 520c	RNA Gene	18	GC19P056391	6.120534	https://www.genecards.org/cgi-bin/carddisp.pl?gene=MIR520C
H4C14	H4 Clustered Histone 14	Protein Coding	29	GC01P149832	6.118846	https://www.genecards.org/cgi-bin/carddisp.pl?gene=H4C14
H4C15	H4 Clustered Histone 15	Protein Coding	27	GC01M150133	6.118846	https://www.genecards.org/cgi-bin/carddisp.pl?gene=H4C15
USP7	Ubiquitin Specific Peptidase 7	Protein Coding	49	GC16M008892	6.116426	https://www.genecards.org/cgi-bin/carddisp.pl?gene=USP7
H4C12	H4 Clustered Histone 12	Protein Coding	29	GC06M047220	6.115572	https://www.genecards.org/cgi-bin/carddisp.pl?gene=H4C12
PANDAR	Promoter Of CDKN1A Antisense DNA Damage Activated RNA	RNA Gene	12	GC06M036673	6.11512	https://www.genecards.org/cgi-bin/carddisp.pl?gene=PANDAR
NDUFS5	NADH:Ubiquinone Oxidoreductase Subunit S5	Protein Coding	41	GC01P039026	6.11116	https://www.genecards.org/cgi-bin/carddisp.pl?gene=NDUFS5

ST3GAL4	ST3 Beta-Galactoside Alpha-2,3-Sialyltransferase 4	Protein Coding	43	GC11P126355	6.109054	https://www.genecards.org/cgi-bin/carddisp.pl?gene=ST3GAL4
SERPINE2	Serpin Family E Member 2	Protein Coding	42	GC02M223975	6.106835	https://www.genecards.org/cgi-bin/carddisp.pl?gene=SERPINE2
NOTUM	Notum, Palmitoleoyl-Protein Carboxylesterase	Protein Coding	36	GC17M081952	6.104705	https://www.genecards.org/cgi-bin/carddisp.pl?gene=NOTUM
H4C5	H4 Clustered Histone 5	Protein Coding	28	GC06P055590	6.102196	https://www.genecards.org/cgi-bin/carddisp.pl?gene=H4C5
HAGLR	HOXD Antisense Growth-Associated Long Non-Coding RNA	RNA Gene	16	GC02M176173	6.101118	https://www.genecards.org/cgi-bin/carddisp.pl?gene=HAGLR
STK4	Serine/Threonine Kinase 4	Protein Coding	50	GC20P044966	6.097652	https://www.genecards.org/cgi-bin/carddisp.pl?gene=STK4
PCSK5	Proprotein Convertase Subtilisin/Kexin Type 5	Protein Coding	41	GC09P075890	6.091687	https://www.genecards.org/cgi-bin/carddisp.pl?gene=PCSK5
FPR1	Formyl Peptide Receptor 1	Protein Coding	48	GC19M051745	6.088187	https://www.genecards.org/cgi-bin/carddisp.pl?gene=FPR1
SH3GL1	SH3 Domain Containing GRB2 Like 1, Endophilin A2	Protein Coding	44	GC19M004360	6.081384	https://www.genecards.org/cgi-bin/carddisp.pl?gene=SH3GL1
LCP1	Lymphocyte Cytosolic Protein 1	Protein Coding	43	GC13M046132	6.080808	https://www.genecards.org/cgi-bin/carddisp.pl?gene=LCP1
TUBB4B	Tubulin Beta 4B Class IVb	Protein Coding	45	GC09P137241	6.08025	https://www.genecards.org/cgi-bin/carddisp.pl?gene=TUBB4B
IRS4	Insulin Receptor Substrate 4	Protein Coding	40	GC0XM108720	6.077898	https://www.genecards.org/cgi-bin/carddisp.pl?gene=IRS4
AQP7	Aquaporin 7	Protein Coding	45	GC09M033384	6.076461	https://www.genecards.org/cgi-bin/carddisp.pl?gene=AQP7
STMN1	Stathmin 1	Protein Coding	44	GC01M025884	6.072724	https://www.genecards.org/cgi-bin/carddisp.pl?gene=STMN1
ALDH3B1	Aldehyde Dehydrogenase 3 Family Member B1	Protein Coding	43	GC11P068027	6.072433	https://www.genecards.org/cgi-bin/carddisp.pl?gene=ALDH3B1
DR1	Down-Regulator Of Transcription 1	Protein Coding	39	GC01P093345	6.064288	https://www.genecards.org/cgi-bin/carddisp.pl?gene=DR1
ANKS1B	Ankyrin Repeat And Sterile Alpha Motif Domain Containing 1B	Protein Coding	40	GC12M098726	6.060338	https://www.genecards.org/cgi-bin/carddisp.pl?gene=ANKS1B
TUBG1	Tubulin Gamma 1	Protein Coding	48	GC17P042609	6.055662	https://www.genecards.org/cgi-bin/carddisp.pl?gene=TUBG1

RPLP2	Ribosomal Protein Lateral Stalk Subunit P2	Protein Coding	42	GC11P000984	6.04952	https://www.genecards.org/cgi-bin/carddisp.pl?gene=RPLP2
ORM1	Orosomucoid 1	Protein Coding	40	GC09P114323	6.049231	https://www.genecards.org/cgi-bin/carddisp.pl?gene=ORM1
HSP90AB1	Heat Shock Protein 90 Alpha Family Class B Member 1	Protein Coding	46	GC06P044246	6.049201	https://www.genecards.org/cgi-bin/carddisp.pl?gene=HSP90AB1
CDC25C	Cell Division Cycle 25C	Protein Coding	49	GC05M138285	6.048976	https://www.genecards.org/cgi-bin/carddisp.pl?gene=CDC25C
SERPINA2	Serpin Family A Member 2 (Gene/Pseudogene)	Protein Coding	27	GC14M095824	6.040594	https://www.genecards.org/cgi-bin/carddisp.pl?gene=SERPINA2
CDK9	Cyclin Dependent Kinase 9	Protein Coding	47	GC09P127852	6.038595	https://www.genecards.org/cgi-bin/carddisp.pl?gene=CDK9
CYP20A1	Cytochrome P450 Family 20 Subfamily A Member 1	Protein Coding	37	GC02P203238	6.032894	https://www.genecards.org/cgi-bin/carddisp.pl?gene=CYP20A1
SLC22A6	Solute Carrier Family 22 Member 6	Protein Coding	45	GC11M069278	6.0297	https://www.genecards.org/cgi-bin/carddisp.pl?gene=SLC22A6
CALD1	Caldesmon 1	Protein Coding	43	GC07P134744	6.025928	https://www.genecards.org/cgi-bin/carddisp.pl?gene=CALD1
GMNN	Geminin DNA Replication Inhibitor	Protein Coding	46	GC06P024779	6.023028	https://www.genecards.org/cgi-bin/carddisp.pl?gene=GMNN
NSDHL	NAD(P) Dependent Steroid Dehydrogenase-Like	Protein Coding	45	GC0XP152830	6.021006	https://www.genecards.org/cgi-bin/carddisp.pl?gene=NSDHL
PFKP	Phosphofructokinase, Platelet	Protein Coding	46	GC10P003066	6.019036	https://www.genecards.org/cgi-bin/carddisp.pl?gene=PFKP
DAGLA	Diacylglycerol Lipase Alpha	Protein Coding	43	GC11P061680	6.015265	https://www.genecards.org/cgi-bin/carddisp.pl?gene=DAGLA
CBLB	Cbl Proto-Oncogene B	Protein Coding	45	GC03M105655	6.014181	https://www.genecards.org/cgi-bin/carddisp.pl?gene=CBLB
RPL13	Ribosomal Protein L13	Protein Coding	43	GC16P089802	6.010226	https://www.genecards.org/cgi-bin/carddisp.pl?gene=RPL13
DDR1	Discoidin Domain Receptor Tyrosine Kinase 1	Protein Coding	47	GC06P055184	6.007812	https://www.genecards.org/cgi-bin/carddisp.pl?gene=DDR1
H3-3B	H3.3 Histone B	Protein Coding	33	GC17M075789	6.005374	https://www.genecards.org/cgi-bin/carddisp.pl?gene=H3-3B
MIR510	MicroRNA 510	RNA Gene	15	GC0XM147272	6.005273	https://www.genecards.org/cgi-bin/carddisp.pl?gene=MIR510
PRKCI	Protein Kinase C Iota	Protein Coding	49	GC03P170222	6.003612	https://www.genecards.org/cgi-bin/carddisp.pl?gene=PRKCI

HCK	HCK Proto-Oncogene, Src Family Tyrosine Kinase	Protein Coding	50	GC20P032052	6.003108	https://www.genecards.org/cgi-bin/carddisp.pl?gene=HCK
TPT1	Tumor Protein, Translationally-Controlled 1	Protein Coding	47	GC13M045333	5.998327	https://www.genecards.org/cgi-bin/carddisp.pl?gene=TPT1
PLAA	Phospholipase A2 Activating Protein	Protein Coding	45	GC09M026903	5.997488	https://www.genecards.org/cgi-bin/carddisp.pl?gene=PLAA
GHRHR	Growth Hormone Releasing Hormone Receptor	Protein Coding	44	GC07P030938	5.995602	https://www.genecards.org/cgi-bin/carddisp.pl?gene=GHRHR
NCAPH2	Non-SMC Condensin II Complex Subunit H2	Protein Coding	38	GC22P050508	5.99403	https://www.genecards.org/cgi-bin/carddisp.pl?gene=NCAPH2
SUZ12	SUZ12 Polycomb Repressive Complex 2 Subunit	Protein Coding	41	GC17P031937	5.989062	https://www.genecards.org/cgi-bin/carddisp.pl?gene=SUZ12
RPL6	Ribosomal Protein L6	Protein Coding	40	GC12M112320	5.987618	https://www.genecards.org/cgi-bin/carddisp.pl?gene=RPL6
KHDRBS1	KH RNA Binding Domain Containing, Signal Transduction Associated 1	Protein Coding	43	GC01P032013	5.987313	https://www.genecards.org/cgi-bin/carddisp.pl?gene=KHDRBS1
RPN1	Ribophorin I	Protein Coding	44	GC03M128619	5.986773	https://www.genecards.org/cgi-bin/carddisp.pl?gene=RPN1
MAPK11	Mitogen-Activated Protein Kinase 11	Protein Coding	48	GC22M050263	5.986226	https://www.genecards.org/cgi-bin/carddisp.pl?gene=MAPK11
GK2	Glycerol Kinase 2	Protein Coding	41	GC04M079406	5.985163	https://www.genecards.org/cgi-bin/carddisp.pl?gene=GK2
LNCRNA-ATB	Long Noncoding RNA Activated By TGF-Beta	RNA Gene	4	GC14P021619	5.985048	https://www.genecards.org/cgi-bin/carddisp.pl?gene=LNCRNA-ATB
EFNB2	Ephrin B2	Protein Coding	44	GC13M106489	5.981742	https://www.genecards.org/cgi-bin/carddisp.pl?gene=EFNB2
WT1-AS	WT1 Antisense RNA	RNA Gene	25	GC11P032434	5.974708	https://www.genecards.org/cgi-bin/carddisp.pl?gene=WT1-AS
MNX1	Motor Neuron And Pancreas Homeobox 1	Protein Coding	43	GC07M156994	5.974269	https://www.genecards.org/cgi-bin/carddisp.pl?gene=MNX1
IFI16	Interferon Gamma Inducible Protein 16	Protein Coding	43	GC01P158969	5.973341	https://www.genecards.org/cgi-bin/carddisp.pl?gene=IFI16

EIF2S3	Eukaryotic Translation Initiation Factor 2 Subunit Gamma	Protein Coding	46	GC0XP024054	5.972932	https://www.genecards.org/cgi-bin/carddisp.pl?gene=EIF2S3
CYP4F8	Cytochrome P450 Family 4 Subfamily F Member 8	Protein Coding	36	GC19P015617	5.971796	https://www.genecards.org/cgi-bin/carddisp.pl?gene=CYP4F8
COA6	Cytochrome C Oxidase Assembly Factor 6	Protein Coding	36	GC01P234374	5.971249	https://www.genecards.org/cgi-bin/carddisp.pl?gene=COA6
TP53I3	Tumor Protein P53 Inducible Protein 3	Protein Coding	43	GC02M024078	5.968201	https://www.genecards.org/cgi-bin/carddisp.pl?gene=TP53I3
NGF-AS1	NGF Antisense RNA 1	RNA Gene	10	GC01P115280	5.968042	https://www.genecards.org/cgi-bin/carddisp.pl?gene=NGF-AS1
TNR	Tenascin R	Protein Coding	43	GC01M175291	5.96588	https://www.genecards.org/cgi-bin/carddisp.pl?gene=TNR
TREH	Trehalase	Protein Coding	43	GC11M118657	5.961953	https://www.genecards.org/cgi-bin/carddisp.pl?gene=TREH
RAB1A	RAB1A, Member RAS Oncogene Family	Protein Coding	43	GC02M065072	5.957717	https://www.genecards.org/cgi-bin/carddisp.pl?gene=RAB1A
TAF4	TATA-Box Binding Protein Associated Factor 4	Protein Coding	40	GC20M061994	5.955125	https://www.genecards.org/cgi-bin/carddisp.pl?gene=TAF4
PAPSS2	3'-Phosphoadenosine 5'-Phosphosulfate Synthase 2	Protein Coding	45	GC10P087659	5.954879	https://www.genecards.org/cgi-bin/carddisp.pl?gene=PAPSS2
CAPZA2	Capping Actin Protein Of Muscle Z-Line Subunit Alpha 2	Protein Coding	42	GC07P116811	5.953159	https://www.genecards.org/cgi-bin/carddisp.pl?gene=CAPZA2
HS3ST1	Heparan Sulfate-Glucosamine 3-Sulfotransferase 1	Protein Coding	43	GC04M011394	5.952091	https://www.genecards.org/cgi-bin/carddisp.pl?gene=HS3ST1
RAB18	RAB18, Member RAS Oncogene Family	Protein Coding	41	GC10P027504	5.950797	https://www.genecards.org/cgi-bin/carddisp.pl?gene=RAB18

LAMTOR5	Late Endosomal/Lysosomal Adaptor, MAPK And MTOR Activator 5	Protein Coding	37	GC01M110401	5.950655	https://www.genecards.org/cgi-bin/carddisp.pl?gene=LAMTOR5
DNAJA3	DnaJ Heat Shock Protein Family (Hsp40) Member A3	Protein Coding	41	GC16P004425	5.950645	https://www.genecards.org/cgi-bin/carddisp.pl?gene=DNAJA3
GLRX	Glutaredoxin	Protein Coding	45	GC05M095752	5.950015	https://www.genecards.org/cgi-bin/carddisp.pl?gene=GLRX
MCM3	Minichromosome Maintenance Complex Component 3	Protein Coding	46	GC06M052264	5.94873	https://www.genecards.org/cgi-bin/carddisp.pl?gene=MCM3
USP24	Ubiquitin Specific Peptidase 24	Protein Coding	44	GC01M055066	5.946398	https://www.genecards.org/cgi-bin/carddisp.pl?gene=USP24
RSAD2	Radical S-Adenosyl Methionine Domain Containing 2	Protein Coding	41	GC02P006865	5.936877	https://www.genecards.org/cgi-bin/carddisp.pl?gene=RSAD2
IL1R2	Interleukin 1 Receptor Type 2	Protein Coding	45	GC02P101991	5.934972	https://www.genecards.org/cgi-bin/carddisp.pl?gene=IL1R2
RPL12	Ribosomal Protein L12	Protein Coding	43	GC09M127447	5.934464	https://www.genecards.org/cgi-bin/carddisp.pl?gene=RPL12
APBA2	Amyloid Beta Precursor Protein Binding Family A Member 2	Protein Coding	43	GC15P029286	5.932015	https://www.genecards.org/cgi-bin/carddisp.pl?gene=APBA2
NDUFA8	NADH:Ubiquinone Oxidoreductase Subunit A8	Protein Coding	43	GC09M122132	5.925574	https://www.genecards.org/cgi-bin/carddisp.pl?gene=NDUFA8
UQCR11	Ubiquinol-Cytochrome C Reductase, Complex III Subunit XI	Protein Coding	37	GC19M001597	5.922102	https://www.genecards.org/cgi-bin/carddisp.pl?gene=UQCR11
SAR1A	Secretion Associated Ras Related GTPase 1A	Protein Coding	43	GC10M070147	5.919758	https://www.genecards.org/cgi-bin/carddisp.pl?gene=SAR1A
KPNB1	Karyopherin Subunit Beta 1	Protein Coding	44	GC17P047649	5.907356	https://www.genecards.org/cgi-bin/carddisp.pl?gene=KPNB1
NDUFA5	NADH:Ubiquinone Oxidoreductase Subunit A5	Protein Coding	43	GC07M123536	5.905532	https://www.genecards.org/cgi-bin/carddisp.pl?gene=NDUFA5

CRHR2	Corticotropin Releasing Hormone Receptor 2	Protein Coding	43	GC07M030651	5.905317	https://www.genecards.org/cgi-bin/carddisp.pl?gene=CRHR2
SPTLC3	Serine Palmitoyltransferase Long Chain Base Subunit 3	Protein Coding	41	GC20P013008	5.905272	https://www.genecards.org/cgi-bin/carddisp.pl?gene=SPTLC3
DDX6	DEAD-Box Helicase 6	Protein Coding	47	GC11M118748	5.899421	https://www.genecards.org/cgi-bin/carddisp.pl?gene=DDX6
FKBP1A	FKBP Prolyl Isomerase 1A	Protein Coding	47	GC20M001369	5.898264	https://www.genecards.org/cgi-bin/carddisp.pl?gene=FKBP1A
TP73-AS1	TP73 Antisense RNA 1	RNA Gene	22	GC01M003735	5.895761	https://www.genecards.org/cgi-bin/carddisp.pl?gene=TP73-AS1
RHEB	Ras Homolog, MTORC1 Binding	Protein Coding	49	GC07M151466	5.892027	https://www.genecards.org/cgi-bin/carddisp.pl?gene=RHEB
GPX7	Glutathione Peroxidase 7	Protein Coding	44	GC01P052602	5.891564	https://www.genecards.org/cgi-bin/carddisp.pl?gene=GPX7
RTL1	Retrotransposon Gag Like 1	Protein Coding	31	GC14M100880	5.889184	https://www.genecards.org/cgi-bin/carddisp.pl?gene=RTL1
NFE2L1	Nuclear Factor, Erythroid 2 Like 1	Protein Coding	44	GC17P048049	5.88813	https://www.genecards.org/cgi-bin/carddisp.pl?gene=NFE2L1
HSPBP1	HSPA (Hsp70) Binding Protein 1	Protein Coding	37	GC19M055262	5.887337	https://www.genecards.org/cgi-bin/carddisp.pl?gene=HSPBP1
PABPC1	Poly(A) Binding Protein Cytoplasmic 1	Protein Coding	43	GC08M100685	5.88552	https://www.genecards.org/cgi-bin/carddisp.pl?gene=PABPC1
ST8SIA1	ST8 Alpha-N-Acetyl-Neuraminide Alpha-2,8-Sialyltransferase 1	Protein Coding	41	GC12M022063	5.883532	https://www.genecards.org/cgi-bin/carddisp.pl?gene=ST8SIA1
TMPRSS2	Transmembrane Serine Protease 2	Protein Coding	45	GC21M041464	5.883377	https://www.genecards.org/cgi-bin/carddisp.pl?gene=TMPRSS2
ACP4	Acid Phosphatase 4	Protein Coding	29	GC19P050791	5.882225	https://www.genecards.org/cgi-bin/carddisp.pl?gene=ACP4
YBX3	Y-Box Binding Protein 3	Protein Coding	40	GC12M015696	5.881638	https://www.genecards.org/cgi-bin/carddisp.pl?gene=YBX3
SRPRB	SRP Receptor Subunit Beta	Protein Coding	38	GC03P133784	5.881456	https://www.genecards.org/cgi-bin/carddisp.pl?gene=SRPRB
PLCD1	Phospholipase C Delta 1	Protein Coding	48	GC03M038008	5.879849	https://www.genecards.org/cgi-bin/carddisp.pl?gene=PLCD1
ERRFI1	ERBB Receptor Feedback Inhibitor 1	Protein Coding	38	GC01M008004	5.879412	https://www.genecards.org/cgi-bin/carddisp.pl?gene=ERRFI1
ZNF395	Zinc Finger Protein 395	Protein Coding	32	GC08M028345	5.876712	https://www.genecards.org/cgi-bin/carddisp.pl?gene=ZNF395
SELPLG	Selectin P Ligand	Protein Coding	43	GC12M108621	5.872985	https://www.genecards.org/cgi-bin/carddisp.pl?gene=SELPLG

CCRL2	C-C Motif Chemokine Receptor Like 2	Protein Coding	40	GC03P046407	5.868557	https://www.genecards.org/cgi-bin/carddisp.pl?gene=CCRL2
TBC1D20	TBC1 Domain Family Member 20	Protein Coding	38	GC20M000423	5.86662	https://www.genecards.org/cgi-bin/carddisp.pl?gene=TBC1D20
DUSP6	Dual Specificity Phosphatase 6	Protein Coding	50	GC12M089347	5.859518	https://www.genecards.org/cgi-bin/carddisp.pl?gene=DUSP6
AGPAT5	1-Acylglycerol-3-Phosphate O-Acyltransferase 5	Protein Coding	42	GC08P006708	5.856453	https://www.genecards.org/cgi-bin/carddisp.pl?gene=AGPAT5
VAT1L	Vesicle Amine Transport 1 Like	Protein Coding	36	GC16P077788	5.855437	https://www.genecards.org/cgi-bin/carddisp.pl?gene=VAT1L
GRK5	G Protein-Coupled Receptor Kinase 5	Protein Coding	44	GC10P119207	5.855268	https://www.genecards.org/cgi-bin/carddisp.pl?gene=GRK5
TNFRSF12A	TNF Receptor Superfamily Member 12A	Protein Coding	44	GC16P003018	5.851414	https://www.genecards.org/cgi-bin/carddisp.pl?gene=TNFRSF12A
LSP1	Lymphocyte Specific Protein 1	Protein Coding	43	GC11P001852	5.848055	https://www.genecards.org/cgi-bin/carddisp.pl?gene=LSP1
IL34	Interleukin 34	Protein Coding	40	GC16P070656	5.845536	https://www.genecards.org/cgi-bin/carddisp.pl?gene=IL34
SIRT5	Sirtuin 5	Protein Coding	45	GC06P013574	5.840052	https://www.genecards.org/cgi-bin/carddisp.pl?gene=SIRT5
HSD17B1	Hydroxysteroid 17-Beta Dehydrogenase 1	Protein Coding	44	GC17P042548	5.839081	https://www.genecards.org/cgi-bin/carddisp.pl?gene=HSD17B1
CCNC	Cyclin C	Protein Coding	41	GC06M099542	5.837391	https://www.genecards.org/cgi-bin/carddisp.pl?gene=CCNC
GCN1	GCN1 Activator Of EIF2AK4	Protein Coding	29	GC12M120128	5.835604	https://www.genecards.org/cgi-bin/carddisp.pl?gene=GCN1
SMAD1	SMAD Family Member 1	Protein Coding	44	GC04P145481	5.8343	https://www.genecards.org/cgi-bin/carddisp.pl?gene=SMAD1
ACBD3	Acyl-CoA Binding Domain Containing 3	Protein Coding	39	GC01M226144	5.83311	https://www.genecards.org/cgi-bin/carddisp.pl?gene=ACBD3
ROR2	Receptor Tyrosine Kinase Like Orphan Receptor 2	Protein Coding	49	GC09M091564	5.827224	https://www.genecards.org/cgi-bin/carddisp.pl?gene=ROR2
PIAS1	Protein Inhibitor Of Activated STAT 1	Protein Coding	45	GC15P068054	5.825125	https://www.genecards.org/cgi-bin/carddisp.pl?gene=PIAS1
CA3	Carbonic Anhydrase 3	Protein Coding	42	GC08P085373	5.824769	https://www.genecards.org/cgi-bin/carddisp.pl?gene=CA3

PCBP2	Poly(RC) Binding Protein 2	Protein Coding	42	GC12P053452	5.822852	https://www.genecards.org/cgi-bin/carddisp.pl?gene=PCBP2
RCVRN	Recoverin	Protein Coding	39	GC17M009896	5.8212	https://www.genecards.org/cgi-bin/carddisp.pl?gene=RCVRN
BCAT1	Branched Chain Amino Acid Transaminase 1	Protein Coding	47	GC12M024732	5.816948	https://www.genecards.org/cgi-bin/carddisp.pl?gene=BCAT1
HNRNPH1	Heterogeneous Nuclear Ribonucleoprotein H1	Protein Coding	40	GC05M179614	5.814712	https://www.genecards.org/cgi-bin/carddisp.pl?gene=HNRNPH1
SEMA4G	Semaphorin 4G	Protein Coding	40	GC10P100969	5.813663	https://www.genecards.org/cgi-bin/carddisp.pl?gene=SEMA4G
PLAAT3	Phospholipase A And Acyltransferase 3	Protein Coding	34	GC11M069298	5.813105	https://www.genecards.org/cgi-bin/carddisp.pl?gene=PLAAT3
UBR5	Ubiquitin Protein Ligase E3 Component N-Recognin 5	Protein Coding	43	GC08M102252	5.810266	https://www.genecards.org/cgi-bin/carddisp.pl?gene=UBR5
METAP2	Methionyl Aminopeptidase 2	Protein Coding	45	GC12P095473	5.806044	https://www.genecards.org/cgi-bin/carddisp.pl?gene=METAP2
PFKFB3	6-Phosphofructo-2-Kinase/Fructose-2,6-Biphosphatase 3	Protein Coding	45	GC10P006144	5.805576	https://www.genecards.org/cgi-bin/carddisp.pl?gene=PFKFB3
MN1	MN1 Proto-Oncogene, Transcriptional Regulator	Protein Coding	43	GC22M027748	5.804852	https://www.genecards.org/cgi-bin/carddisp.pl?gene=MN1
TAC3	Tachykinin Precursor 3	Protein Coding	45	GC12M057009	5.802037	https://www.genecards.org/cgi-bin/carddisp.pl?gene=TAC3
ARHGAP1	Rho GTPase Activating Protein 1	Protein Coding	44	GC11M068978	5.801293	https://www.genecards.org/cgi-bin/carddisp.pl?gene=ARHGAP1
RPS11	Ribosomal Protein S11	Protein Coding	40	GC19P049496	5.797147	https://www.genecards.org/cgi-bin/carddisp.pl?gene=RPS11
DUSP19	Dual Specificity Phosphatase 19	Protein Coding	39	GC02P183078	5.794615	https://www.genecards.org/cgi-bin/carddisp.pl?gene=DUSP19
IL3RA	Interleukin 3 Receptor Subunit Alpha	Protein Coding	43	GC0XP001336	5.793341	https://www.genecards.org/cgi-bin/carddisp.pl?gene=IL3RA
CADPS	Calcium Dependent Secretion Activator	Protein Coding	41	GC03M062398	5.792899	https://www.genecards.org/cgi-bin/carddisp.pl?gene=CADPS

IGF2BP1	Insulin Like Growth Factor 2 MRNA Binding Protein 1	Protein Coding	41	GC17P048997	5.786264	https://www.genecards.org/cgi-bin/carddisp.pl?gene=IGF2BP1
SVIL	Supervillin	Protein Coding	42	GC10M031105	5.781621	https://www.genecards.org/cgi-bin/carddisp.pl?gene=SVIL
SLC3A2	Solute Carrier Family 3 Member 2	Protein Coding	43	GC11P062856	5.775519	https://www.genecards.org/cgi-bin/carddisp.pl?gene=SLC3A2
AKAP13	A-Kinase Anchoring Protein 13	Protein Coding	44	GC15P085388	5.773824	https://www.genecards.org/cgi-bin/carddisp.pl?gene=AKAP13
PRKACB	Protein Kinase CAMP-Activated Catalytic Subunit Beta	Protein Coding	50	GC01P084078	5.772684	https://www.genecards.org/cgi-bin/carddisp.pl?gene=PRKACB
SLC25A11	Solute Carrier Family 25 Member 11	Protein Coding	44	GC17M004937	5.769321	https://www.genecards.org/cgi-bin/carddisp.pl?gene=SLC25A11
DHDH	Dihydrodiol Dehydrogenase	Protein Coding	37	GC19P048934	5.766547	https://www.genecards.org/cgi-bin/carddisp.pl?gene=DHDH
USF2	Upstream Transcription Factor 2, C-Fos Interacting	Protein Coding	40	GC19P035268	5.763486	https://www.genecards.org/cgi-bin/carddisp.pl?gene=USF2
BCAT2	Branched Chain Amino Acid Transaminase 2	Protein Coding	46	GC19M048795	5.760746	https://www.genecards.org/cgi-bin/carddisp.pl?gene=BCAT2
MOGAT3	Monoacylglycerol O-Acyltransferase 3	Protein Coding	33	GC07M101192	5.758605	https://www.genecards.org/cgi-bin/carddisp.pl?gene=MOGAT3
NORAD	Non-Coding RNA Activated By DNA Damage	RNA Gene	14	GC20M036096	5.754325	https://www.genecards.org/cgi-bin/carddisp.pl?gene=NORAD
PADI2	Peptidyl Arginine Deiminase 2	Protein Coding	42	GC01M017066	5.74716	https://www.genecards.org/cgi-bin/carddisp.pl?gene=PADI2
CTF1	Cardiotrophin 1	Protein Coding	40	GC16P032484	5.746569	https://www.genecards.org/cgi-bin/carddisp.pl?gene=CTF1
BCYRN1	Brain Cytoplasmic RNA 1	RNA Gene	18	GC02P047331	5.746406	https://www.genecards.org/cgi-bin/carddisp.pl?gene=BCYRN1
PRDM5	PR/SET Domain 5	Protein Coding	40	GC04M120686	5.746255	https://www.genecards.org/cgi-bin/carddisp.pl?gene=PRDM5
ARNT2	Aryl Hydrocarbon Receptor Nuclear Translocator 2	Protein Coding	44	GC15P080404	5.744976	https://www.genecards.org/cgi-bin/carddisp.pl?gene=ARNT2
PLS1	Plastin 1	Protein Coding	42	GC03P142596	5.738134	https://www.genecards.org/cgi-bin/carddisp.pl?gene=PLS1

SGMS2	Sphingomyelin Synthase 2	Protein Coding	37	GC04P107824	5.73733	https://www.genecards.org/cgi-bin/carddisp.pl?gene=SGMS2
IL5RA	Interleukin 5 Receptor Subunit Alpha	Protein Coding	47	GC03M003066	5.736702	https://www.genecards.org/cgi-bin/carddisp.pl?gene=IL5RA
DEFB103B	Defensin Beta 103B	Protein Coding	30	GC08M007430	5.735948	https://www.genecards.org/cgi-bin/carddisp.pl?gene=DEFB103B
SLC4A7	Solute Carrier Family 4 Member 7	Protein Coding	43	GC03M027372	5.735288	https://www.genecards.org/cgi-bin/carddisp.pl?gene=SLC4A7
BRD2	Bromodomain Containing 2	Protein Coding	45	GC06P055236	5.73501	https://www.genecards.org/cgi-bin/carddisp.pl?gene=BRD2
GGPS1	Geranylgeranyl Diphosphate Synthase 1	Protein Coding	45	GC01P235327	5.733788	https://www.genecards.org/cgi-bin/carddisp.pl?gene=GGPS1
PPP1CA	Protein Phosphatase 1 Catalytic Subunit Alpha	Protein Coding	49	GC11M069512	5.73245	https://www.genecards.org/cgi-bin/carddisp.pl?gene=PPP1CA
UBE2N	Ubiquitin Conjugating Enzyme E2 N	Protein Coding	47	GC12M093406	5.730811	https://www.genecards.org/cgi-bin/carddisp.pl?gene=UBE2N
GJD2	Gap Junction Protein Delta 2	Protein Coding	43	GC15M034751	5.728963	https://www.genecards.org/cgi-bin/carddisp.pl?gene=GJD2
MIR20B	MicroRNA 20b	RNA Gene	15	GC0XM134217	5.726795	https://www.genecards.org/cgi-bin/carddisp.pl?gene=MIR20B
PANX1	Pannexin 1	Protein Coding	44	GC11P094128	5.713655	https://www.genecards.org/cgi-bin/carddisp.pl?gene=PANX1
HS6ST1	Heparan Sulfate 6-O-Sulfotransferase 1	Protein Coding	43	GC02M128236	5.709884	https://www.genecards.org/cgi-bin/carddisp.pl?gene=HS6ST1
NPY2R	Neuropeptide Y Receptor Y2	Protein Coding	44	GC04P155173	5.705987	https://www.genecards.org/cgi-bin/carddisp.pl?gene=NPY2R
LARP7	La Ribonucleoprotein 7, Transcriptional Regulator	Protein Coding	42	GC04P112636	5.698277	https://www.genecards.org/cgi-bin/carddisp.pl?gene=LARP7
DCT	Dopachrome Tautomerase	Protein Coding	43	GC13M094436	5.69643	https://www.genecards.org/cgi-bin/carddisp.pl?gene=DCT
HOXA-AS2	HOXA Cluster Antisense RNA 2	RNA Gene	18	GC07P027107	5.695453	https://www.genecards.org/cgi-bin/carddisp.pl?gene=HOXA-AS2
RAB6A	RAB6A, Member RAS Oncogene Family	Protein Coding	41	GC11M073676	5.693328	https://www.genecards.org/cgi-bin/carddisp.pl?gene=RAB6A
HIBADH	3-Hydroxyisobutyrate Dehydrogenase	Protein Coding	42	GC07M027525	5.691217	https://www.genecards.org/cgi-bin/carddisp.pl?gene=HIBADH
ADCY1	Adenylate Cyclase 1	Protein Coding	49	GC07P045580	5.690069	https://www.genecards.org/cgi-bin/carddisp.pl?gene=ADCY1
NFAT5	Nuclear Factor Of Activated T Cells 5	Protein Coding	43	GC16P069565	5.689214	https://www.genecards.org/cgi-bin/carddisp.pl?gene=NFAT5

RNF216	Ring Finger Protein 216	Protein Coding	43	GC07M005620	5.687888	https://www.genecards.org/cgi-bin/carddisp.pl?gene=RNF216
MAPK7	Mitogen-Activated Protein Kinase 7	Protein Coding	49	GC17P019379	5.687428	https://www.genecards.org/cgi-bin/carddisp.pl?gene=MAPK7
DGCR2	DiGeorge Syndrome Critical Region Gene 2	Protein Coding	40	GC22M019037	5.686914	https://www.genecards.org/cgi-bin/carddisp.pl?gene=DGCR2
AGO2	Argonaute RISC Catalytic Component 2	Protein Coding	41	GC08M140522	5.685075	https://www.genecards.org/cgi-bin/carddisp.pl?gene=AGO2
SSRP1	Structure Specific Recognition Protein 1	Protein Coding	41	GC11M069072	5.6831	https://www.genecards.org/cgi-bin/carddisp.pl?gene=SSRP1
CWF19L1	CWF19 Like Cell Cycle Control Factor 1	Protein Coding	39	GC10M100232	5.680615	https://www.genecards.org/cgi-bin/carddisp.pl?gene=CWF19L1
RTN4R	Reticulon 4 Receptor	Protein Coding	43	GC22M020241	5.679897	https://www.genecards.org/cgi-bin/carddisp.pl?gene=RTN4R
EMSY	EMSY Transcriptional Repressor, BRCA2 Interacting	Protein Coding	33	GC11P076445	5.677684	https://www.genecards.org/cgi-bin/carddisp.pl?gene=EMSY
PDE2A	Phosphodiesterase 2A	Protein Coding	46	GC11M072576	5.6731	https://www.genecards.org/cgi-bin/carddisp.pl?gene=PDE2A
MIR500A	MicroRNA 500a	RNA Gene	16	GC0XP050008	5.672725	https://www.genecards.org/cgi-bin/carddisp.pl?gene=MIR500A
EMX2	Empty Spiracles Homeobox 2	Protein Coding	44	GC10P117542	5.669665	https://www.genecards.org/cgi-bin/carddisp.pl?gene=EMX2
ACSS1	Acyl-CoA Synthetase Short Chain Family Member 1	Protein Coding	40	GC20M024986	5.665409	https://www.genecards.org/cgi-bin/carddisp.pl?gene=ACSS1
SORCS2	Sortilin Related VPS10 Domain Containing Receptor 2	Protein Coding	36	GC04P007196	5.664694	https://www.genecards.org/cgi-bin/carddisp.pl?gene=SORCS2
LMAN1	Lectin, Mannose Binding 1	Protein Coding	46	GC18M059327	5.660633	https://www.genecards.org/cgi-bin/carddisp.pl?gene=LMAN1
CYSLTR2	Cysteinyl Leukotriene Receptor 2	Protein Coding	48	GC13P048653	5.660558	https://www.genecards.org/cgi-bin/carddisp.pl?gene=CYSLTR2
POLH	DNA Polymerase Eta	Protein Coding	49	GC06P043576	5.659925	https://www.genecards.org/cgi-bin/carddisp.pl?gene=POLH
MYO1A	Myosin IA	Protein Coding	40	GC12M057028	5.657652	https://www.genecards.org/cgi-bin/carddisp.pl?gene=MYO1A
FOXN1	Forkhead Box N1	Protein Coding	42	GC17P028506	5.657597	https://www.genecards.org/cgi-bin/carddisp.pl?gene=FOXN1

BPIFA1	BPI Fold Containing Family A Member 1	Protein Coding	37	GC20P033235	5.656493	https://www.genecards.org/cgi-bin/carddisp.pl?gene=BPIFA1
RORB	RAR Related Orphan Receptor B	Protein Coding	48	GC09P074497	5.650209	https://www.genecards.org/cgi-bin/carddisp.pl?gene=RORB
AIM2	Absent In Melanoma 2	Protein Coding	43	GC01M159062	5.648614	https://www.genecards.org/cgi-bin/carddisp.pl?gene=AIM2
NR2F1	Nuclear Receptor Subfamily 2 Group F Member 1	Protein Coding	46	GC05P093583	5.640584	https://www.genecards.org/cgi-bin/carddisp.pl?gene=NR2F1
HNRNPM	Heterogeneous Nuclear Ribonucleoprotein M	Protein Coding	40	GC19P008444	5.631485	https://www.genecards.org/cgi-bin/carddisp.pl?gene=HNRNPM
INHBA	Inhibin Subunit Beta A	Protein Coding	45	GC07M041668	5.630105	https://www.genecards.org/cgi-bin/carddisp.pl?gene=INHBA
SPEN	Spen Family Transcriptional Repressor	Protein Coding	38	GC01P015848	5.609621	https://www.genecards.org/cgi-bin/carddisp.pl?gene=SPEN
NDUFB7	NADH:Ubiquinone Oxidoreductase Subunit B7	Protein Coding	40	GC19M014566	5.60186	https://www.genecards.org/cgi-bin/carddisp.pl?gene=NDUFB7
EFS	Embryonal Fyn-Associated Substrate	Protein Coding	36	GC14M023356	5.595194	https://www.genecards.org/cgi-bin/carddisp.pl?gene=EFS
ING3	Inhibitor Of Growth Family Member 3	Protein Coding	40	GC07P120950	5.594891	https://www.genecards.org/cgi-bin/carddisp.pl?gene=ING3
NUDT4	Nudix Hydrolase 4	Protein Coding	40	GC12P093392	5.590994	https://www.genecards.org/cgi-bin/carddisp.pl?gene=NUDT4
NR2C2	Nuclear Receptor Subfamily 2 Group C Member 2	Protein Coding	45	GC03P014947	5.587327	https://www.genecards.org/cgi-bin/carddisp.pl?gene=NR2C2
CPLANE2	Ciliogenesis And Planar Polarity Effector 2	Protein Coding	25	GC01M016232	5.583817	https://www.genecards.org/cgi-bin/carddisp.pl?gene=CPLANE2
HSD17B11	Hydroxysteroid 17-Beta Dehydrogenase 11	Protein Coding	41	GC04M087336	5.576313	https://www.genecards.org/cgi-bin/carddisp.pl?gene=HSD17B11
GDF3	Growth Differentiation Factor 3	Protein Coding	41	GC12M007689	5.574468	https://www.genecards.org/cgi-bin/carddisp.pl?gene=GDF3
EHBP1L1	EH Domain Binding Protein 1 Like 1	Protein Coding	32	GC11P066231	5.569793	https://www.genecards.org/cgi-bin/carddisp.pl?gene=EHBP1L1
CNDP2	Carnosine Dipeptidase 2	Protein Coding	44	GC18P074495	5.569459	https://www.genecards.org/cgi-bin/carddisp.pl?gene=CNDP2

IL13RA2	Interleukin 13 Receptor Subunit Alpha 2	Protein Coding	41	GC0XM115003	5.568297	https://www.genecards.org/cgi-bin/carddisp.pl?gene=IL13RA2
ESM1	Endothelial Cell Specific Molecule 1	Protein Coding	40	GC05M054977	5.567933	https://www.genecards.org/cgi-bin/carddisp.pl?gene=ESM1
CBX5	Chromobox 5	Protein Coding	45	GC12M054230	5.563202	https://www.genecards.org/cgi-bin/carddisp.pl?gene=CBX5
GRHL2	Grainyhead Like Transcription Factor 2	Protein Coding	41	GC08P101492	5.555061	https://www.genecards.org/cgi-bin/carddisp.pl?gene=GRHL2
AEBP1	AE Binding Protein 1	Protein Coding	41	GC07P044106	5.553801	https://www.genecards.org/cgi-bin/carddisp.pl?gene=AEBP1
CCL24	C-C Motif Chemokine Ligand 24	Protein Coding	37	GC07M075815	5.551489	https://www.genecards.org/cgi-bin/carddisp.pl?gene=CCL24
NNT-AS1	NNT Antisense RNA 1	RNA Gene	14	GC05M043741	5.5483	https://www.genecards.org/cgi-bin/carddisp.pl?gene=NNT-AS1
HGS	Hepatocyte Growth Factor-Regulated Tyrosine Kinase Substrate	Protein Coding	45	GC17P081683	5.548202	https://www.genecards.org/cgi-bin/carddisp.pl?gene=HGS
NANS	N-Acetylneuraminate Synthase	Protein Coding	45	GC09P098056	5.546607	https://www.genecards.org/cgi-bin/carddisp.pl?gene=NANS
ADAMTS1	ADAM Metallopeptidase With Thrombospondin Type 1 Motif 1	Protein Coding	45	GC21M026835	5.544445	https://www.genecards.org/cgi-bin/carddisp.pl?gene=ADAMTS1
MYL1	Myosin Light Chain 1	Protein Coding	43	GC02M210290	5.541769	https://www.genecards.org/cgi-bin/carddisp.pl?gene=MYL1
RGS4	Regulator Of G Protein Signaling 4	Protein Coding	44	GC01P163038	5.540104	https://www.genecards.org/cgi-bin/carddisp.pl?gene=RGS4
IGFBP6	Insulin Like Growth Factor Binding Protein 6	Protein Coding	43	GC12P053097	5.538567	https://www.genecards.org/cgi-bin/carddisp.pl?gene=IGFBP6
TCF12	Transcription Factor 12	Protein Coding	47	GC15P056918	5.535733	https://www.genecards.org/cgi-bin/carddisp.pl?gene=TCF12
RPL10A	Ribosomal Protein L10a	Protein Coding	43	GC06P055328	5.527932	https://www.genecards.org/cgi-bin/carddisp.pl?gene=RPL10A
NUDT1	Nudix Hydrolase 1	Protein Coding	44	GC07P002242	5.527001	https://www.genecards.org/cgi-bin/carddisp.pl?gene=NUDT1
IGFBP4	Insulin Like Growth Factor Binding Protein 4	Protein Coding	43	GC17P040443	5.526307	https://www.genecards.org/cgi-bin/carddisp.pl?gene=IGFBP4
ACER2	Alkaline Ceramidase 2	Protein Coding	37	GC09P019408	5.523192	https://www.genecards.org/cgi-bin/carddisp.pl?gene=ACER2

FARP1	FERM, ARH/RhoGEF And Pleckstrin Domain Protein 1	Protein Coding	38	GC13P098142	5.523026	https://www.genecards.org/cgi-bin/carddisp.pl?gene=FARP1
NCL	Nucleolin	Protein Coding	43	GC02M231453	5.522409	https://www.genecards.org/cgi-bin/carddisp.pl?gene=NCL
VPS13D	Vacuolar Protein Sorting 13 Homolog D	Protein Coding	34	GC01P012231	5.520661	https://www.genecards.org/cgi-bin/carddisp.pl?gene=VPS13D
UBE2G2	Ubiquitin Conjugating Enzyme E2 G2	Protein Coding	45	GC21M044768	5.51684	https://www.genecards.org/cgi-bin/carddisp.pl?gene=UBE2G2
P2RY2	Purinergic Receptor P2Y2	Protein Coding	47	GC11P073202	5.513649	https://www.genecards.org/cgi-bin/carddisp.pl?gene=P2RY2
H2AC20	H2A Clustered Histone 20	Protein Coding	31	GC01P149978	5.510845	https://www.genecards.org/cgi-bin/carddisp.pl?gene=H2AC20
ID1	Inhibitor Of DNA Binding 1, HLH Protein	Protein Coding	44	GC20P031605	5.510283	https://www.genecards.org/cgi-bin/carddisp.pl?gene=ID1
MIR409	MicroRNA 409	RNA Gene	18	GC14P106324	5.501606	https://www.genecards.org/cgi-bin/carddisp.pl?gene=MIR409
CTPS1	CTP Synthase 1	Protein Coding	45	GC01P040979	5.500762	https://www.genecards.org/cgi-bin/carddisp.pl?gene=CTPS1
SLC17A1	Solute Carrier Family 17 Member 1	Protein Coding	40	GC06M025723	5.49742	https://www.genecards.org/cgi-bin/carddisp.pl?gene=SLC17A1
ENPP7	Ectonucleotide Pyrophosphatase/Phosphodiesterase 7	Protein Coding	38	GC17P079730	5.491338	https://www.genecards.org/cgi-bin/carddisp.pl?gene=ENPP7
FRK	Fyn Related Src Family Tyrosine Kinase	Protein Coding	45	GC06M115931	5.490207	https://www.genecards.org/cgi-bin/carddisp.pl?gene=FRK
NUP155	Nucleoporin 155	Protein Coding	45	GC05M037288	5.48787	https://www.genecards.org/cgi-bin/carddisp.pl?gene=NUP155
PSMD7	Proteasome 26S Subunit, Non-ATPase 7	Protein Coding	44	GC16P074296	5.485281	https://www.genecards.org/cgi-bin/carddisp.pl?gene=PSMD7
YWHAZ	Tyrosine 3-Monooxygenase/Tryptophan 5-Monooxygenase Activation Protein Zeta	Protein Coding	48	GC08M100917	5.480808	https://www.genecards.org/cgi-bin/carddisp.pl?gene=YWHAZ
PLA2G12A	Phospholipase A2 Group XIIA	Protein Coding	42	GC04M109712	5.473032	https://www.genecards.org/cgi-bin/carddisp.pl?gene=PLA2G12A
GNB5	G Protein Subunit Beta 5	Protein Coding	45	GC15M067468	5.466584	https://www.genecards.org/cgi-bin/carddisp.pl?gene=GNB5
CLDN4	Claudin 4	Protein Coding	42	GC07P073799	5.464571	https://www.genecards.org/cgi-bin/carddisp.pl?gene=CLDN4
URGCP	Upregulator Of Cell Proliferation	Protein Coding	34	GC07M043876	5.455553	https://www.genecards.org/cgi-bin/carddisp.pl?gene=URGCP

GRM4	Glutamate Metabotropic Receptor 4	Protein Coding	45	GC06M047013	5.453603	https://www.genecards.org/cgi-bin/carddisp.pl?gene=GRM4
ME1	Malic Enzyme 1	Protein Coding	43	GC06M083210	5.449981	https://www.genecards.org/cgi-bin/carddisp.pl?gene=ME1
GJC3	Gap Junction Protein Gamma 3	Protein Coding	40	GC07M099923	5.445215	https://www.genecards.org/cgi-bin/carddisp.pl?gene=GJC3
TSBP1-AS1	TSBP1 And BTNL2 Antisense RNA 1	RNA Gene	9	GC06P058058	5.443883	https://www.genecards.org/cgi-bin/carddisp.pl?gene=TSBP1-AS1
UQCR10	Ubiquinol-Cytochrome C Reductase, Complex III Subunit X	Protein Coding	40	GC22P029767	5.443162	https://www.genecards.org/cgi-bin/carddisp.pl?gene=UQCR10
IL1RAP	Interleukin 1 Receptor Accessory Protein	Protein Coding	44	GC03P190514	5.442783	https://www.genecards.org/cgi-bin/carddisp.pl?gene=IL1RAP
S100A10	S100 Calcium Binding Protein A10	Protein Coding	45	GC01M151955	5.439061	https://www.genecards.org/cgi-bin/carddisp.pl?gene=S100A10
CPSF6	Cleavage And Polyadenylation Specific Factor 6	Protein Coding	40	GC12P069239	5.438096	https://www.genecards.org/cgi-bin/carddisp.pl?gene=CPSF6
IL32	Interleukin 32	Protein Coding	40	GC16P005617	5.431749	https://www.genecards.org/cgi-bin/carddisp.pl?gene=IL32
CNTN5	Contactin 5	Protein Coding	37	GC11P099020	5.429941	https://www.genecards.org/cgi-bin/carddisp.pl?gene=CNTN5
TRA2B	Transformer 2 Beta Homolog	Protein Coding	40	GC03M185914	5.42932	https://www.genecards.org/cgi-bin/carddisp.pl?gene=TRA2B
CREB3	CAMP Responsive Element Binding Protein 3	Protein Coding	39	GC09P035960	5.421911	https://www.genecards.org/cgi-bin/carddisp.pl?gene=CREB3
CLDN7	Claudin 7	Protein Coding	42	GC17M007259	5.421853	https://www.genecards.org/cgi-bin/carddisp.pl?gene=CLDN7
LAPTM4A	Lysosomal Protein Transmembrane 4 Alpha	Protein Coding	38	GC02M020032	5.418144	https://www.genecards.org/cgi-bin/carddisp.pl?gene=LAPTM4A
MIR497	MicroRNA 497	RNA Gene	18	GC17M007022	5.416584	https://www.genecards.org/cgi-bin/carddisp.pl?gene=MIR497
NAGK	N-Acetylglucosamine Kinase	Protein Coding	44	GC02P071064	5.416053	https://www.genecards.org/cgi-bin/carddisp.pl?gene=NAGK
CD276	CD276 Molecule	Protein Coding	40	GC15P073683	5.408948	https://www.genecards.org/cgi-bin/carddisp.pl?gene=CD276
MIRLET7F2	MicroRNA Let-7f-2	RNA Gene	18	GC0XM053596	5.407678	https://www.genecards.org/cgi-bin/carddisp.pl?gene=MIRLET7F2

ORC1	Origin Recognition Complex Subunit 1	Protein Coding	44	GC01M052372	5.406778	https://www.genecards.org/cgi-bin/carddisp.pl?gene=ORC1
FAF2	Fas Associated Factor Family Member 2	Protein Coding	38	GC05P176447	5.404696	https://www.genecards.org/cgi-bin/carddisp.pl?gene=FAF2
TOLLIP	Toll Interacting Protein	Protein Coding	44	GC11M001274	5.40376	https://www.genecards.org/cgi-bin/carddisp.pl?gene=TOLLIP
MDH1	Malate Dehydrogenase 1	Protein Coding	47	GC02P063557	5.403732	https://www.genecards.org/cgi-bin/carddisp.pl?gene=MDH1
PTPRU	Protein Tyrosine Phosphatase Receptor Type U	Protein Coding	43	GC01P029236	5.398726	https://www.genecards.org/cgi-bin/carddisp.pl?gene=PTPRU
GGH	Gamma-Glutamyl Hydrolase	Protein Coding	44	GC08M063015	5.397708	https://www.genecards.org/cgi-bin/carddisp.pl?gene=GGH
CDK7	Cyclin Dependent Kinase 7	Protein Coding	47	GC05P069263	5.396034	https://www.genecards.org/cgi-bin/carddisp.pl?gene=CDK7
SEC24B	SEC24 Homolog B, COPII Coat Complex Component	Protein Coding	40	GC04P109433	5.395859	https://www.genecards.org/cgi-bin/carddisp.pl?gene=SEC24B
BCORL1	BCL6 Corepressor Like 1	Protein Coding	35	GC0XP129980	5.395348	https://www.genecards.org/cgi-bin/carddisp.pl?gene=BCORL1
ZIC1	Zic Family Member 1	Protein Coding	46	GC03P147393	5.393857	https://www.genecards.org/cgi-bin/carddisp.pl?gene=ZIC1
ATCAY	ATCAY Kinesin Light Chain Interacting Caytaxin	Protein Coding	38	GC19P003880	5.388311	https://www.genecards.org/cgi-bin/carddisp.pl?gene=ATCAY
SLC25A32	Solute Carrier Family 25 Member 32	Protein Coding	41	GC08M103398	5.387505	https://www.genecards.org/cgi-bin/carddisp.pl?gene=SLC25A32
MCM5	Minichromosome Maintenance Complex Component 5	Protein Coding	46	GC22P035400	5.386617	https://www.genecards.org/cgi-bin/carddisp.pl?gene=MCM5
RPL3	Ribosomal Protein L3	Protein Coding	42	GC22M048526	5.386133	https://www.genecards.org/cgi-bin/carddisp.pl?gene=RPL3
HTR4	5-Hydroxytryptamine Receptor 4	Protein Coding	45	GC05M148451	5.383527	https://www.genecards.org/cgi-bin/carddisp.pl?gene=HTR4
DDIT4	DNA Damage Inducible Transcript 4	Protein Coding	44	GC10P072273	5.382831	https://www.genecards.org/cgi-bin/carddisp.pl?gene=DDIT4
CCL13	C-C Motif Chemokine Ligand 13	Protein Coding	38	GC17P034356	5.382045	https://www.genecards.org/cgi-bin/carddisp.pl?gene=CCL13

NIN	Ninein	Protein Coding	43	GC14M050719	5.380236	https://www.genecards.org/cgi-bin/carddisp.pl?gene=NIN
SENP7	SUMO Specific Peptidase 7	Protein Coding	37	GC03M101324	5.376885	https://www.genecards.org/cgi-bin/carddisp.pl?gene=SENP7
PRKAG3	Protein Kinase AMP-Activated Non-Catalytic Subunit Gamma 3	Protein Coding	44	GC02M218823	5.376197	https://www.genecards.org/cgi-bin/carddisp.pl?gene=PRKAG3
CCT7	Chaperonin Containing TCP1 Subunit 7	Protein Coding	41	GC02P073233	5.373137	https://www.genecards.org/cgi-bin/carddisp.pl?gene=CCT7
COX5B	Cytochrome C Oxidase Subunit 5B	Protein Coding	42	GC02P097628	5.372967	https://www.genecards.org/cgi-bin/carddisp.pl?gene=COX5B
CTSS	Cathepsin S	Protein Coding	46	GC01M150730	5.369229	https://www.genecards.org/cgi-bin/carddisp.pl?gene=CTSS
DEK	DEK Proto-Oncogene	Protein Coding	41	GC06M018224	5.353565	https://www.genecards.org/cgi-bin/carddisp.pl?gene=DEK
RPLP1	Ribosomal Protein Lateral Stalk Subunit P1	Protein Coding	41	GC15P077111	5.348802	https://www.genecards.org/cgi-bin/carddisp.pl?gene=RPLP1
HRH3	Histamine Receptor H3	Protein Coding	43	GC20M062214	5.346373	https://www.genecards.org/cgi-bin/carddisp.pl?gene=HRH3
PPP1R15B	Protein Phosphatase 1 Regulatory Subunit 15B	Protein Coding	38	GC01M204400	5.345397	https://www.genecards.org/cgi-bin/carddisp.pl?gene=PPP1R15B
FBXO32	F-Box Protein 32	Protein Coding	41	GC08M123498	5.343992	https://www.genecards.org/cgi-bin/carddisp.pl?gene=FBXO32
GDF9	Growth Differentiation Factor 9	Protein Coding	40	GC05M132861	5.343338	https://www.genecards.org/cgi-bin/carddisp.pl?gene=GDF9
GGT3P	Gamma-Glutamyltransferase 3 Pseudogene	Pseudogene	18	GC22M018773	5.342343	https://www.genecards.org/cgi-bin/carddisp.pl?gene=GGT3P
NEGR1	Neuronal Growth Regulator 1	Protein Coding	40	GC01M071395	5.337886	https://www.genecards.org/cgi-bin/carddisp.pl?gene=NEGR1
MRTFA	Myocardin Related Transcription Factor A	Protein Coding	35	GC22M049024	5.33699	https://www.genecards.org/cgi-bin/carddisp.pl?gene=MRTFA
CHDH	Choline Dehydrogenase	Protein Coding	41	GC03M053812	5.330841	https://www.genecards.org/cgi-bin/carddisp.pl?gene=CHDH
HNRNPA3	Heterogeneous Nuclear Ribonucleoprotein A3	Protein Coding	38	GC02P177212	5.330005	https://www.genecards.org/cgi-bin/carddisp.pl?gene=HNRNPA3
KPTN	Kaptin, Actin Binding Protein	Protein Coding	37	GC19M047475	5.329216	https://www.genecards.org/cgi-bin/carddisp.pl?gene=KPTN

RPL30	Ribosomal Protein L30	Protein Coding	41	GC08M098024	5.327885	https://www.genecards.org/cgi-bin/carddisp.pl?gene=RPL30
RPS23	Ribosomal Protein S23	Protein Coding	41	GC05M082273	5.326473	https://www.genecards.org/cgi-bin/carddisp.pl?gene=RPS23
ZNF462	Zinc Finger Protein 462	Protein Coding	40	GC09P106860	5.326409	https://www.genecards.org/cgi-bin/carddisp.pl?gene=ZNF462
DIAPH1	Diaphanous Related Formin 1	Protein Coding	48	GC05M141516	5.325309	https://www.genecards.org/cgi-bin/carddisp.pl?gene=DIAPH1
SGO1	Shugoshin 1	Protein Coding	35	GC03M020159	5.324475	https://www.genecards.org/cgi-bin/carddisp.pl?gene=SGO1
CCNG1	Cyclin G1	Protein Coding	43	GC05P163438	5.324472	https://www.genecards.org/cgi-bin/carddisp.pl?gene=CCNG1
CLASRP	CLK4 Associating Serine/Arginine Rich Protein	Protein Coding	33	GC19P045039	5.322005	https://www.genecards.org/cgi-bin/carddisp.pl?gene=CLASRP
PAEP	Progestagen Associated Endometrial Protein	Protein Coding	40	GC09P135561	5.31604	https://www.genecards.org/cgi-bin/carddisp.pl?gene=PAEP
ASXL2	ASXL Transcriptional Regulator 2	Protein Coding	38	GC02M025733	5.315167	https://www.genecards.org/cgi-bin/carddisp.pl?gene=ASXL2
ITGA5	Integrin Subunit Alpha 5	Protein Coding	49	GC12M054398	5.312746	https://www.genecards.org/cgi-bin/carddisp.pl?gene=ITGA5
ANGPTL6	Angiopoietin Like 6	Protein Coding	40	GC19M010092	5.31098	https://www.genecards.org/cgi-bin/carddisp.pl?gene=ANGPTL6
TAB1	TGF-Beta Activated Kinase 1 (MAP3K7) Binding Protein 1	Protein Coding	43	GC22P039403	5.310094	https://www.genecards.org/cgi-bin/carddisp.pl?gene=TAB1
SCG5	Secretogranin V	Protein Coding	38	GC15P032641	5.309402	https://www.genecards.org/cgi-bin/carddisp.pl?gene=SCG5
CERS2	Ceramide Synthase 2	Protein Coding	40	GC01M150934	5.306704	https://www.genecards.org/cgi-bin/carddisp.pl?gene=CERS2
WDR11	WD Repeat Domain 11	Protein Coding	38	GC10P120851	5.305739	https://www.genecards.org/cgi-bin/carddisp.pl?gene=WDR11
LATS1	Large Tumor Suppressor Kinase 1	Protein Coding	46	GC06M149658	5.300023	https://www.genecards.org/cgi-bin/carddisp.pl?gene=LATS1
PHB2	Prohibitin 2	Protein Coding	41	GC12M006965	5.298043	https://www.genecards.org/cgi-bin/carddisp.pl?gene=PHB2
PDCD1LG2	Programmed Cell Death 1 Ligand 2	Protein Coding	42	GC09P005510	5.297392	https://www.genecards.org/cgi-bin/carddisp.pl?gene=PDCD1LG2
H3C14	H3 Clustered Histone 14	Protein Coding	29	GC01M150132	5.295563	https://www.genecards.org/cgi-bin/carddisp.pl?gene=H3C14
BLMH	Bleomycin Hydrolase	Protein Coding	44	GC17M030248	5.294733	https://www.genecards.org/cgi-bin/carddisp.pl?gene=BLMH
TUBGCP6	Tubulin Gamma Complex Associated Protein 6	Protein Coding	40	GC22M050217	5.293694	https://www.genecards.org/cgi-bin/carddisp.pl?gene=TUBGCP6

TARBP1	TAR (HIV-1) RNA Binding Protein 1	Protein Coding	39	GC01M234391	5.293316	https://www.genecards.org/cgi-bin/carddisp.pl?gene=TARBP1
HNRNPUL1	Heterogeneous Nuclear Ribonucleoprotein U Like 1	Protein Coding	38	GC19P041262	5.292706	https://www.genecards.org/cgi-bin/carddisp.pl?gene=HNRNPUL1
CSN3	Casein Kappa	Protein Coding	35	GC04P070242	5.28902	https://www.genecards.org/cgi-bin/carddisp.pl?gene=CSN3
GBF1	Golgi Brefeldin A Resistant Guanine Nucleotide Exchange Factor 1	Protein Coding	44	GC10P102245	5.288924	https://www.genecards.org/cgi-bin/carddisp.pl?gene=GBF1
RXRG	Retinoid X Receptor Gamma	Protein Coding	43	GC01M165401	5.285746	https://www.genecards.org/cgi-bin/carddisp.pl?gene=RXRG
HNRNPF	Heterogeneous Nuclear Ribonucleoprotein F	Protein Coding	41	GC10M043385	5.282759	https://www.genecards.org/cgi-bin/carddisp.pl?gene=HNRNPF
SORCS1	Sortilin Related VPS10 Domain Containing Receptor 1	Protein Coding	39	GC10M106573	5.279704	https://www.genecards.org/cgi-bin/carddisp.pl?gene=SORCS1
TNFRSF21	TNF Receptor Superfamily Member 21	Protein Coding	45	GC06M047231	5.269147	https://www.genecards.org/cgi-bin/carddisp.pl?gene=TNFRSF21
CYSLTR1	Cysteinyl Leukotriene Receptor 1	Protein Coding	44	GC0XM078271	5.267043	https://www.genecards.org/cgi-bin/carddisp.pl?gene=CYSLTR1
H3C1	H3 Clustered Histone 1	Protein Coding	33	GC06P054980	5.262321	https://www.genecards.org/cgi-bin/carddisp.pl?gene=H3C1
TUBA8	Tubulin Alpha 8	Protein Coding	45	GC22P018110	5.253999	https://www.genecards.org/cgi-bin/carddisp.pl?gene=TUBA8
STRADA	STE20 Related Adaptor Alpha	Protein Coding	46	GC17M063683	5.251883	https://www.genecards.org/cgi-bin/carddisp.pl?gene=STRADA
FETUB	Fetuin B	Protein Coding	37	GC03P186635	5.250519	https://www.genecards.org/cgi-bin/carddisp.pl?gene=FETUB
SLC16A9	Solute Carrier Family 16 Member 9	Protein Coding	40	GC10M059650	5.249752	https://www.genecards.org/cgi-bin/carddisp.pl?gene=SLC16A9
PDCD4	Programmed Cell Death 4	Protein Coding	43	GC10P110871	5.247894	https://www.genecards.org/cgi-bin/carddisp.pl?gene=PDCD4
CKS1B	CDC28 Protein Kinase Regulatory Subunit 1B	Protein Coding	43	GC01P154974	5.244964	https://www.genecards.org/cgi-bin/carddisp.pl?gene=CKS1B
ATP2B1	ATPase Plasma Membrane Ca2+ Transporting 1	Protein Coding	45	GC12M089588	5.244315	https://www.genecards.org/cgi-bin/carddisp.pl?gene=ATP2B1
EFNA1	Ephrin A1	Protein Coding	44	GC01P155127	5.244009	https://www.genecards.org/cgi-bin/carddisp.pl?gene=EFNA1
PCBP1	Poly(RC) Binding Protein 1	Protein Coding	43	GC02P070087	5.239912	https://www.genecards.org/cgi-bin/carddisp.pl?gene=PCBP1

HIPK2	Homeodomain Interacting Protein Kinase 2	Protein Coding	45	GC07M139561	5.239304	https://www.genecards.org/cgi-bin/carddisp.pl?gene=HIPK2
MIR708	MicroRNA 708	RNA Gene	18	GC11M079402	5.237569	https://www.genecards.org/cgi-bin/carddisp.pl?gene=MIR708
RAB10	RAB10, Member RAS Oncogene Family	Protein Coding	44	GC02P026033	5.231869	https://www.genecards.org/cgi-bin/carddisp.pl?gene=RAB10
YIPF1	Yip1 Domain Family Member 1	Protein Coding	36	GC01M053851	5.226182	https://www.genecards.org/cgi-bin/carddisp.pl?gene=YIPF1
NFE2	Nuclear Factor, Erythroid 2	Protein Coding	41	GC12M054292	5.225863	https://www.genecards.org/cgi-bin/carddisp.pl?gene=NFE2
RBSN	Rabenosyn, RAB Effector	Protein Coding	38	GC03M015070	5.224337	https://www.genecards.org/cgi-bin/carddisp.pl?gene=RBSN
TAPBPL	TAP Binding Protein Like	Protein Coding	36	GC12P006451	5.223886	https://www.genecards.org/cgi-bin/carddisp.pl?gene=TAPBPL
NKILA	NF-KappaB Interacting LncRNA	RNA Gene	11	GC20P057711	5.223182	https://www.genecards.org/cgi-bin/carddisp.pl?gene=NKILA
HLX	H2.0 Like Homeobox	Protein Coding	41	GC01P220879	5.220929	https://www.genecards.org/cgi-bin/carddisp.pl?gene=HLX
CRTC1	CREB Regulated Transcription Coactivator 1	Protein Coding	43	GC19P026660	5.217909	https://www.genecards.org/cgi-bin/carddisp.pl?gene=CRTC1
GMDS	GDP-Mannose 4,6-Dehydratase	Protein Coding	45	GC06M001624	5.214139	https://www.genecards.org/cgi-bin/carddisp.pl?gene=GMDS
ETV5	ETS Variant Transcription Factor 5	Protein Coding	40	GC03M186046	5.212271	https://www.genecards.org/cgi-bin/carddisp.pl?gene=ETV5
COX7A2L	Cytochrome C Oxidase Subunit 7A2 Like	Protein Coding	42	GC02M042312	5.210041	https://www.genecards.org/cgi-bin/carddisp.pl?gene=COX7A2L
NACC1	Nucleus Accumbens Associated 1	Protein Coding	41	GC19P013117	5.208193	https://www.genecards.org/cgi-bin/carddisp.pl?gene=NACC1
DRAIC	Downregulated RNA In Cancer, Inhibitor Of Cell Invasion And Migration	RNA Gene	14	GC15P077110	5.206625	https://www.genecards.org/cgi-bin/carddisp.pl?gene=DRAIC
EPS8	Epidermal Growth Factor Receptor Pathway Substrate 8	Protein Coding	46	GC12M015765	5.204076	https://www.genecards.org/cgi-bin/carddisp.pl?gene=EPS8
SNX5	Sorting Nexin 5	Protein Coding	43	GC20M017989	5.197326	https://www.genecards.org/cgi-bin/carddisp.pl?gene=SNX5

NDUFB6	NADH:Ubiquinone Oxidoreductase Subunit B6	Protein Coding	40	GC09M032553	5.196278	https://www.genecards.org/cgi-bin/carddisp.pl?gene=NDUFB6
VAPA	VAMP Associated Protein A	Protein Coding	45	GC18P009904	5.193147	https://www.genecards.org/cgi-bin/carddisp.pl?gene=VAPA
LMF1	Lipase Maturation Factor 1	Protein Coding	39	GC16M000853	5.186234	https://www.genecards.org/cgi-bin/carddisp.pl?gene=LMF1
CREB5	CAMP Responsive Element Binding Protein 5	Protein Coding	41	GC07P028305	5.186048	https://www.genecards.org/cgi-bin/carddisp.pl?gene=CREB5
CCAR2	Cell Cycle And Apoptosis Regulator 2	Protein Coding	37	GC08P022604	5.180923	https://www.genecards.org/cgi-bin/carddisp.pl?gene=CCAR2
ADI1	Acireductone Dioxygenase 1	Protein Coding	41	GC02M003501	5.177456	https://www.genecards.org/cgi-bin/carddisp.pl?gene=ADI1
PAICS	Phosphoribosylaminoimidazole Carboxylase And Phosphoribosylaminoimidazolesuccinocarboxamide Synthase	Protein Coding	43	GC04P056410	5.174293	https://www.genecards.org/cgi-bin/carddisp.pl?gene=PAICS
SERPINA4	Serpin Family A Member 4	Protein Coding	41	GC14P094561	5.171369	https://www.genecards.org/cgi-bin/carddisp.pl?gene=SERPINA4
DNAJA1	DnaJ Heat Shock Protein Family (Hsp40) Member A1	Protein Coding	42	GC09P033025	5.169736	https://www.genecards.org/cgi-bin/carddisp.pl?gene=DNAJA1
DDX5	DEAD-Box Helicase 5	Protein Coding	47	GC17M064498	5.166311	https://www.genecards.org/cgi-bin/carddisp.pl?gene=DDX5
CDC45	Cell Division Cycle 45	Protein Coding	45	GC22P019479	5.163903	https://www.genecards.org/cgi-bin/carddisp.pl?gene=CDC45
PTGER3	Prostaglandin E Receptor 3	Protein Coding	47	GC01M070852	5.162956	https://www.genecards.org/cgi-bin/carddisp.pl?gene=PTGER3
MIR449A	MicroRNA 449a	RNA Gene	20	GC05M055171	5.161534	https://www.genecards.org/cgi-bin/carddisp.pl?gene=MIR449A
EPHB1	EPH Receptor B1	Protein Coding	48	GC03P134598	5.158829	https://www.genecards.org/cgi-bin/carddisp.pl?gene=EPHB1
ACTN3	Actinin Alpha 3	Protein Coding	40	GC11P066546	5.152532	https://www.genecards.org/cgi-bin/carddisp.pl?gene=ACTN3
SLC46A3	Solute Carrier Family 46 Member 3	Protein Coding	37	GC13M028700	5.150859	https://www.genecards.org/cgi-bin/carddisp.pl?gene=SLC46A3
PIK3C2G	Phosphatidylinositol-4-Phosphate 3-Kinase Catalytic Subunit Type 2 Gamma	Protein Coding	45	GC12P018242	5.150577	https://www.genecards.org/cgi-bin/carddisp.pl?gene=PIK3C2G

CCDC88A	Coiled-Coil Domain Containing 88A	Protein Coding	41	GC02M055287	5.148807	https://www.genecards.org/cgi-bin/carddisp.pl?gene=CCDC88A
SLC20A1	Solute Carrier Family 20 Member 1	Protein Coding	45	GC02P116687	5.147126	https://www.genecards.org/cgi-bin/carddisp.pl?gene=SLC20A1
ADRA1D	Adrenoceptor Alpha 1D	Protein Coding	47	GC20M004220	5.139572	https://www.genecards.org/cgi-bin/carddisp.pl?gene=ADRA1D
HTR1D	5-Hydroxytryptamine Receptor 1D	Protein Coding	47	GC01M023191	5.136132	https://www.genecards.org/cgi-bin/carddisp.pl?gene=HTR1D
ARHGAP15	Rho GTPase Activating Protein 15	Protein Coding	40	GC02P143070	5.135595	https://www.genecards.org/cgi-bin/carddisp.pl?gene=ARHGAP15
BST2	Bone Marrow Stromal Cell Antigen 2	Protein Coding	40	GC19M017403	5.132555	https://www.genecards.org/cgi-bin/carddisp.pl?gene=BST2
HIC1	HIC ZBTB Transcriptional Repressor 1	Protein Coding	41	GC17P002054	5.129323	https://www.genecards.org/cgi-bin/carddisp.pl?gene=HIC1
PVR	PVR Cell Adhesion Molecule	Protein Coding	45	GC19P044644	5.129155	https://www.genecards.org/cgi-bin/carddisp.pl?gene=PVR
GABARAP	GABA Type A Receptor-Associated Protein	Protein Coding	45	GC17M007240	5.123386	https://www.genecards.org/cgi-bin/carddisp.pl?gene=GABARAP
ISCA1	Iron-Sulfur Cluster Assembly 1	Protein Coding	38	GC09M086264	5.119548	https://www.genecards.org/cgi-bin/carddisp.pl?gene=ISCA1
RPL9	Ribosomal Protein L9	Protein Coding	43	GC04M039452	5.117983	https://www.genecards.org/cgi-bin/carddisp.pl?gene=RPL9
RPS2	Ribosomal Protein S2	Protein Coding	44	GC16M003048	5.1156	https://www.genecards.org/cgi-bin/carddisp.pl?gene=RPS2
SLC30A9	Solute Carrier Family 30 Member 9	Protein Coding	42	GC04P041992	5.1155	https://www.genecards.org/cgi-bin/carddisp.pl?gene=SLC30A9
MBOAT2	Membrane Bound O-Acyltransferase Domain Containing 2	Protein Coding	36	GC02M008853	5.114184	https://www.genecards.org/cgi-bin/carddisp.pl?gene=MBOAT2
EDC3	Enhancer Of MRNA Decapping 3	Protein Coding	43	GC15M074631	5.112063	https://www.genecards.org/cgi-bin/carddisp.pl?gene=EDC3
ABCA2	ATP Binding Cassette Subfamily A Member 2	Protein Coding	42	GC09M137007	5.106331	https://www.genecards.org/cgi-bin/carddisp.pl?gene=ABCA2
POU3F2	POU Class 3 Homeobox 2	Protein Coding	43	GC06P098834	5.104515	https://www.genecards.org/cgi-bin/carddisp.pl?gene=POU3F2
CD47	CD47 Molecule	Protein Coding	44	GC03M108043	5.103173	https://www.genecards.org/cgi-bin/carddisp.pl?gene=CD47
LPAR3	Lysophosphatidic Acid Receptor 3	Protein Coding	44	GC01M084811	5.102558	https://www.genecards.org/cgi-bin/carddisp.pl?gene=LPAR3

CLPS	Colipase	Protein Coding	41	GC06M047029	5.101025	https://www.genecards.org/cgi-bin/carddisp.pl?gene=CLPS
KAT2A	Lysine Acetyltransferase 2A	Protein Coding	48	GC17M042113	5.100401	https://www.genecards.org/cgi-bin/carddisp.pl?gene=KAT2A
H3-2	H3.2 Histone (Putative)	Protein Coding	17	GC01M143894	5.099957	https://www.genecards.org/cgi-bin/carddisp.pl?gene=H3-2
CCN4	Cellular Communication Network Factor 4	Protein Coding	31	GC08P133192	5.098077	https://www.genecards.org/cgi-bin/carddisp.pl?gene=CCN4
SUGP1	SURP And G-Patch Domain Containing 1	Protein Coding	35	GC19M019276	5.097383	https://www.genecards.org/cgi-bin/carddisp.pl?gene=SUGP1
ZBP1	Z-DNA Binding Protein 1	Protein Coding	39	GC20M057603	5.08673	https://www.genecards.org/cgi-bin/carddisp.pl?gene=ZBP1
CDCA7	Cell Division Cycle Associated 7	Protein Coding	43	GC02P173354	5.085614	https://www.genecards.org/cgi-bin/carddisp.pl?gene=CDCA7
SLC23A1	Solute Carrier Family 23 Member 1	Protein Coding	43	GC05M139377	5.083595	https://www.genecards.org/cgi-bin/carddisp.pl?gene=SLC23A1
IRF2	Interferon Regulatory Factor 2	Protein Coding	44	GC04M184387	5.082381	https://www.genecards.org/cgi-bin/carddisp.pl?gene=IRF2
CLCC1	Chloride Channel CLIC Like 1	Protein Coding	39	GC01M108946	5.079663	https://www.genecards.org/cgi-bin/carddisp.pl?gene=CLCC1
NUB1	Negative Regulator Of Ubiquitin Like Proteins 1	Protein Coding	38	GC07P151341	5.078976	https://www.genecards.org/cgi-bin/carddisp.pl?gene=NUB1
BCO1	Beta-Carotene Oxygenase 1	Protein Coding	37	GC16P081238	5.078071	https://www.genecards.org/cgi-bin/carddisp.pl?gene=BCO1
RPS9	Ribosomal Protein S9	Protein Coding	43	GC19P056806	5.077768	https://www.genecards.org/cgi-bin/carddisp.pl?gene=RPS9
LPCAT1	Lysophosphatidylcholine Acyltransferase 1	Protein Coding	37	GC05M001456	5.075504	https://www.genecards.org/cgi-bin/carddisp.pl?gene=LPCAT1
TSEN34	TRNA Splicing Endonuclease Subunit 34	Protein Coding	39	GC19P056807	5.072359	https://www.genecards.org/cgi-bin/carddisp.pl?gene=TSEN34
BAG1	BAG Cochaperone 1	Protein Coding	43	GC09M033245	5.067912	https://www.genecards.org/cgi-bin/carddisp.pl?gene=BAG1
GPR3	G Protein-Coupled Receptor 3	Protein Coding	38	GC01P027393	5.062634	https://www.genecards.org/cgi-bin/carddisp.pl?gene=GPR3
MYO18A	Myosin XVIIIA	Protein Coding	38	GC17M031089	5.058605	https://www.genecards.org/cgi-bin/carddisp.pl?gene=MYO18A
PRRX1	Paired Related Homeobox 1	Protein Coding	43	GC01P170662	5.056304	https://www.genecards.org/cgi-bin/carddisp.pl?gene=PRRX1
NPRL2	NPR2 Like, GATOR1 Complex Subunit	Protein Coding	40	GC03M050432	5.05545	https://www.genecards.org/cgi-bin/carddisp.pl?gene=NPRL2

GMPS	Guanine Monophosphate Synthase	Protein Coding	45	GC03P155870	5.05473	https://www.genecards.org/cgi-bin/carddisp.pl?gene=GMPS
NPR3	Natriuretic Peptide Receptor 3	Protein Coding	45	GC05P032689	5.052431	https://www.genecards.org/cgi-bin/carddisp.pl?gene=NPR3
GATAD2A	GATA Zinc Finger Domain Containing 2A	Protein Coding	39	GC19P026663	5.045151	https://www.genecards.org/cgi-bin/carddisp.pl?gene=GATAD2A
HSPH1	Heat Shock Protein Family H (Hsp110) Member 1	Protein Coding	44	GC13M031134	5.043779	https://www.genecards.org/cgi-bin/carddisp.pl?gene=HSPH1
SORBS1	Sorbin And SH3 Domain Containing 1	Protein Coding	42	GC10M095311	5.04074	https://www.genecards.org/cgi-bin/carddisp.pl?gene=SORBS1
H1-2	H1.2 Linker Histone, Cluster Member	Protein Coding	33	GC06M026056	5.039464	https://www.genecards.org/cgi-bin/carddisp.pl?gene=H1-2
PITPNA	Phosphatidylinositol Transfer Protein Alpha	Protein Coding	41	GC17M001530	5.034901	https://www.genecards.org/cgi-bin/carddisp.pl?gene=PITPNA
TSC22D3	TSC22 Domain Family Member 3	Protein Coding	40	GC0XM107713	5.034853	https://www.genecards.org/cgi-bin/carddisp.pl?gene=TSC22D3
TRIM33	Tripartite Motif Containing 33	Protein Coding	44	GC01M114392	5.034423	https://www.genecards.org/cgi-bin/carddisp.pl?gene=TRIM33
ACSS3	Acyl-CoA Synthetase Short Chain Family Member 3	Protein Coding	40	GC12P080936	5.027635	https://www.genecards.org/cgi-bin/carddisp.pl?gene=ACSS3
TRMT1	TRNA Methyltransferase 1	Protein Coding	43	GC19M013104	5.026994	https://www.genecards.org/cgi-bin/carddisp.pl?gene=TRMT1
ADORA3	Adenosine A3 Receptor	Protein Coding	47	GC01M111499	5.025189	https://www.genecards.org/cgi-bin/carddisp.pl?gene=ADORA3
MIR339	MicroRNA 339	RNA Gene	19	GC07M001022	5.021282	https://www.genecards.org/cgi-bin/carddisp.pl?gene=MIR339
ADARB1	Adenosine Deaminase RNA Specific B1	Protein Coding	45	GC21P045073	5.019946	https://www.genecards.org/cgi-bin/carddisp.pl?gene=ADARB1
AFM	Afamin	Protein Coding	39	GC04P073481	5.019556	https://www.genecards.org/cgi-bin/carddisp.pl?gene=AFM
ZFP36	ZFP36 Ring Finger Protein	Protein Coding	40	GC19P039406	5.019497	https://www.genecards.org/cgi-bin/carddisp.pl?gene=ZFP36
ITGB5	Integrin Subunit Beta 5	Protein Coding	47	GC03M124761	5.017797	https://www.genecards.org/cgi-bin/carddisp.pl?gene=ITGB5
RAD51B	RAD51 Paralog B	Protein Coding	39	GC14P067819	5.014824	https://www.genecards.org/cgi-bin/carddisp.pl?gene=RAD51B
DDX1	DEAD-Box Helicase 1	Protein Coding	44	GC02P015591	5.01444	https://www.genecards.org/cgi-bin/carddisp.pl?gene=DDX1
PPM1B	Protein Phosphatase, Mg2+/Mn2+ Dependent 1B	Protein Coding	45	GC02P044167	5.0135	https://www.genecards.org/cgi-bin/carddisp.pl?gene=PPM1B

NRP2	Neuropilin 2	Protein Coding	44	GC02P205681	5.013129	https://www.genecards.org/cgi-bin/carddisp.pl?gene=NRP2
ETV4	ETS Variant Transcription Factor 4	Protein Coding	44	GC17M043527	5.005972	https://www.genecards.org/cgi-bin/carddisp.pl?gene=ETV4
ELAVL2	ELAV Like RNA Binding Protein 2	Protein Coding	43	GC09M023690	5.004536	https://www.genecards.org/cgi-bin/carddisp.pl?gene=ELAVL2
CLDN11	Claudin 11	Protein Coding	41	GC03P170418	5.000122	https://www.genecards.org/cgi-bin/carddisp.pl?gene=CLDN11
ABHD4	Abhydrolase Domain Containing 4, N-Acyl Phospholipase B	Protein Coding	38	GC14P026301	4.995811	https://www.genecards.org/cgi-bin/carddisp.pl?gene=ABHD4
VIL1	Villin 1	Protein Coding	40	GC02P218419	4.985763	https://www.genecards.org/cgi-bin/carddisp.pl?gene=VIL1
TRMT10A	TRNA Methyltransferase 10A	Protein Coding	37	GC04M099546	4.983926	https://www.genecards.org/cgi-bin/carddisp.pl?gene=TRMT10A
RGS1	Regulator Of G Protein Signaling 1	Protein Coding	39	GC01P192575	4.97747	https://www.genecards.org/cgi-bin/carddisp.pl?gene=RGS1
CLRN1-AS1	CLRN1 Antisense RNA 1	RNA Gene	17	GC03P150852	4.974565	https://www.genecards.org/cgi-bin/carddisp.pl?gene=CLRN1-AS1
TCIM	Transcriptional And Immune Response Regulator	Protein Coding	26	GC08P040153	4.973174	https://www.genecards.org/cgi-bin/carddisp.pl?gene=TCIM
ANOS1	Anosmin 1	Protein Coding	36	GC0XM008528	4.971453	https://www.genecards.org/cgi-bin/carddisp.pl?gene=ANOS1
ASH2L	ASH2 Like, Histone Lysine Methyltransferase Complex Subunit	Protein Coding	43	GC08P038104	4.9712	https://www.genecards.org/cgi-bin/carddisp.pl?gene=ASH2L
LAMC1	Laminin Subunit Gamma 1	Protein Coding	44	GC01P182992	4.96647	https://www.genecards.org/cgi-bin/carddisp.pl?gene=LAMC1
BTG1	BTG Anti-Proliferation Factor 1	Protein Coding	42	GC12M092140	4.961415	https://www.genecards.org/cgi-bin/carddisp.pl?gene=BTG1
FGF17	Fibroblast Growth Factor 17	Protein Coding	46	GC08P022042	4.959321	https://www.genecards.org/cgi-bin/carddisp.pl?gene=FGF17
SH3GL2	SH3 Domain Containing GRB2 Like 2, Endophilin A1	Protein Coding	44	GC09P017569	4.958919	https://www.genecards.org/cgi-bin/carddisp.pl?gene=SH3GL2
SASH1	SAM And SH3 Domain Containing 1	Protein Coding	40	GC06P148193	4.9572	https://www.genecards.org/cgi-bin/carddisp.pl?gene=SASH1
SMPD3	Sphingomyelin Phosphodiesterase 3	Protein Coding	41	GC16M068358	4.953752	https://www.genecards.org/cgi-bin/carddisp.pl?gene=SMPD3

ILF3	Interleukin Enhancer Binding Factor 3	Protein Coding	39	GC19P010625	4.949592	https://www.genecards.org/cgi-bin/carddisp.pl?gene=ILF3
UHRF1	Ubiquitin Like With PHD And Ring Finger Domains 1	Protein Coding	40	GC19P004910	4.949112	https://www.genecards.org/cgi-bin/carddisp.pl?gene=UHRF1
KPNA1	Karyopherin Subunit Alpha 1	Protein Coding	42	GC03M122421	4.948489	https://www.genecards.org/cgi-bin/carddisp.pl?gene=KPNA1
DEPTOR	DEP Domain Containing MTOR Interacting Protein	Protein Coding	40	GC08P119873	4.945203	https://www.genecards.org/cgi-bin/carddisp.pl?gene=DEPTOR
QARS1	Glutaminyl-TRNA Synthetase 1	Protein Coding	26	GC03M049445	4.941287	https://www.genecards.org/cgi-bin/carddisp.pl?gene=QARS1
GRHL3	Grainyhead Like Transcription Factor 3	Protein Coding	39	GC01P024319	4.940988	https://www.genecards.org/cgi-bin/carddisp.pl?gene=GRHL3
RALA	RAS Like Proto-Oncogene A	Protein Coding	46	GC07P039622	4.939548	https://www.genecards.org/cgi-bin/carddisp.pl?gene=RALA
MBTPS1	Membrane Bound Transcription Factor Peptidase, Site 1	Protein Coding	44	GC16M084053	4.933723	https://www.genecards.org/cgi-bin/carddisp.pl?gene=MBTPS1
PMPCB	Peptidase, Mitochondrial Processing Subunit Beta	Protein Coding	44	GC07P103297	4.930259	https://www.genecards.org/cgi-bin/carddisp.pl?gene=PMPCB
NSF	N-Ethylmaleimide Sensitive Factor, Vesicle Fusing ATPase	Protein Coding	45	GC17P046590	4.923376	https://www.genecards.org/cgi-bin/carddisp.pl?gene=NSF
AHSA1	Activator Of HSP90 ATPase Activity 1	Protein Coding	40	GC14P077457	4.922819	https://www.genecards.org/cgi-bin/carddisp.pl?gene=AHSA1
IGF2BP3	Insulin Like Growth Factor 2 MRNA Binding Protein 3	Protein Coding	42	GC07M023316	4.922432	https://www.genecards.org/cgi-bin/carddisp.pl?gene=IGF2BP3
DHX9	DExH-Box Helicase 9	Protein Coding	41	GC01P182839	4.920265	https://www.genecards.org/cgi-bin/carddisp.pl?gene=DHX9
LINC01018	Long Intergenic Non-Protein Coding RNA 1018	RNA Gene	16	GC05P006582	4.91708	https://www.genecards.org/cgi-bin/carddisp.pl?gene=LINC01018

PRUNE1	Prune Exopolyphosphatase 1	Protein Coding	33	GC01P151008	4.913444	https://www.genecards.org/cgi-bin/carddisp.pl?gene=PRUNE1
PAPSS1	3'-Phosphoadenosine 5'-Phosphosulfate Synthase 1	Protein Coding	44	GC04M107590	4.909002	https://www.genecards.org/cgi-bin/carddisp.pl?gene=PAPSS1
GTF2H4	General Transcription Factor IIH Subunit 4	Protein Coding	41	GC06P055185	4.904338	https://www.genecards.org/cgi-bin/carddisp.pl?gene=GTF2H4
CSAD	Cysteine Sulfinic Acid Decarboxylase	Protein Coding	41	GC12M053160	4.903358	https://www.genecards.org/cgi-bin/carddisp.pl?gene=CSAD
RBM28	RNA Binding Motif Protein 28	Protein Coding	39	GC07M128320	4.903311	https://www.genecards.org/cgi-bin/carddisp.pl?gene=RBM28
CERNA2	Competing Endogenous LncRNA 2 For MicroRNA Let-7b	RNA Gene	11	GC10M084169	4.899542	https://www.genecards.org/cgi-bin/carddisp.pl?gene=CERNA2
SERPINB4	Serpin Family B Member 4	Protein Coding	39	GC18M063637	4.897576	https://www.genecards.org/cgi-bin/carddisp.pl?gene=SERPINB4
ZP4	Zona Pellucida Glycoprotein 4	Protein Coding	33	GC01M237877	4.897009	https://www.genecards.org/cgi-bin/carddisp.pl?gene=ZP4
RSPO4	R-Spondin 4	Protein Coding	37	GC20M000958	4.895551	https://www.genecards.org/cgi-bin/carddisp.pl?gene=RSPO4
GPN1	GPN-Loop GTPase 1	Protein Coding	38	GC02P027628	4.889051	https://www.genecards.org/cgi-bin/carddisp.pl?gene=GPN1
NMT2	N-Myristoyltransferase 2	Protein Coding	41	GC10M015115	4.888861	https://www.genecards.org/cgi-bin/carddisp.pl?gene=NMT2
SLC23A2	Solute Carrier Family 23 Member 2	Protein Coding	43	GC20M004852	4.887442	https://www.genecards.org/cgi-bin/carddisp.pl?gene=SLC23A2
HPGDS	Hematopoietic Prostaglandin D Synthase	Protein Coding	43	GC04M094298	4.882298	https://www.genecards.org/cgi-bin/carddisp.pl?gene=HPGDS
MED19	Mediator Complex Subunit 19	Protein Coding	33	GC11M057703	4.881899	https://www.genecards.org/cgi-bin/carddisp.pl?gene=MED19
CASP4	Caspase 4	Protein Coding	47	GC11M104942	4.878319	https://www.genecards.org/cgi-bin/carddisp.pl?gene=CASP4
MAD2L1	Mitotic Arrest Deficient 2 Like 1	Protein Coding	46	GC04M120055	4.868076	https://www.genecards.org/cgi-bin/carddisp.pl?gene=MAD2L1
PELP1	Proline, Glutamate And Leucine Rich Protein 1	Protein Coding	40	GC17M004669	4.866016	https://www.genecards.org/cgi-bin/carddisp.pl?gene=PELP1
PNPLA7	Patatin Like Phospholipase Domain Containing 7	Protein Coding	34	GC09M137459	4.864989	https://www.genecards.org/cgi-bin/carddisp.pl?gene=PNPLA7

KPNA2	Karyopherin Subunit Alpha 2	Protein Coding	46	GC17P068035	4.86267	https://www.genecards.org/cgi-bin/carddisp.pl?gene=KPNA2
EEF1D	Eukaryotic Translation Elongation Factor 1 Delta	Protein Coding	43	GC08M143579	4.859773	https://www.genecards.org/cgi-bin/carddisp.pl?gene=EEF1D
RLIM	Ring Finger Protein, LIM Domain Interacting	Protein Coding	40	GC0XM074612	4.859679	https://www.genecards.org/cgi-bin/carddisp.pl?gene=RLIM
SP3	Sp3 Transcription Factor	Protein Coding	43	GC02M173882	4.850002	https://www.genecards.org/cgi-bin/carddisp.pl?gene=SP3
TMEM119	Transmembrane Protein 119	Protein Coding	33	GC12M108589	4.849542	https://www.genecards.org/cgi-bin/carddisp.pl?gene=TMEM119
CHL1	Cell Adhesion Molecule L1 Like	Protein Coding	40	GC03P000213	4.849366	https://www.genecards.org/cgi-bin/carddisp.pl?gene=CHL1
ABCG4	ATP Binding Cassette Subfamily G Member 4	Protein Coding	40	GC11P119150	4.835479	https://www.genecards.org/cgi-bin/carddisp.pl?gene=ABCG4
PACSIN3	Protein Kinase C And Casein Kinase Substrate In Neurons 3	Protein Coding	38	GC11M068990	4.833481	https://www.genecards.org/cgi-bin/carddisp.pl?gene=PACSIN3
HIF1AN	Hypoxia Inducible Factor 1 Subunit Alpha Inhibitor	Protein Coding	45	GC10P100529	4.83341	https://www.genecards.org/cgi-bin/carddisp.pl?gene=HIF1AN
RNF114	Ring Finger Protein 114	Protein Coding	40	GC20P049936	4.831755	https://www.genecards.org/cgi-bin/carddisp.pl?gene=RNF114
CCNE2	Cyclin E2	Protein Coding	43	GC08M094879	4.827953	https://www.genecards.org/cgi-bin/carddisp.pl?gene=CCNE2
SAFB	Scaffold Attachment Factor B	Protein Coding	40	GC19P005623	4.825727	https://www.genecards.org/cgi-bin/carddisp.pl?gene=SAFB
SULF2	Sulfatase 2	Protein Coding	41	GC20M047656	4.820007	https://www.genecards.org/cgi-bin/carddisp.pl?gene=SULF2
WEE1	WEE1 G2 Checkpoint Kinase	Protein Coding	47	GC11P009573	4.817445	https://www.genecards.org/cgi-bin/carddisp.pl?gene=WEE1
CAMK2D	Calcium/Calmodulin Dependent Protein Kinase II Delta	Protein Coding	50	GC04M113452	4.813475	https://www.genecards.org/cgi-bin/carddisp.pl?gene=CAMK2D
FAM107B	Family With Sequence Similarity 107 Member B	Protein Coding	34	GC10M014519	4.809675	https://www.genecards.org/cgi-bin/carddisp.pl?gene=FAM107B
NDUFA3	NADH:Ubiquinone Oxidoreductase Subunit A3	Protein Coding	39	GC19P054102	4.80818	https://www.genecards.org/cgi-bin/carddisp.pl?gene=NDUFA3

SH3BP5	SH3 Domain Binding Protein 5	Protein Coding	41	GC03M016936	4.805813	https://www.genecards.org/cgi-bin/carddisp.pl?gene=SH3BP5
ENAH	ENAH Actin Regulator	Protein Coding	42	GC01M225486	4.804333	https://www.genecards.org/cgi-bin/carddisp.pl?gene=ENAH
MRC2	Mannose Receptor C Type 2	Protein Coding	41	GC17P062627	4.803771	https://www.genecards.org/cgi-bin/carddisp.pl?gene=MRC2
ROCK2	Rho Associated Coiled-Coil Containing Protein Kinase 2	Protein Coding	48	GC02M011227	4.803446	https://www.genecards.org/cgi-bin/carddisp.pl?gene=ROCK2
LINC01772	Long Intergenic Non-Protein Coding RNA 1772	RNA Gene	11	GC01P016507	4.801759	https://www.genecards.org/cgi-bin/carddisp.pl?gene=LINC01772
MIR202	MicroRNA 202	RNA Gene	18	GC10M133247	4.797756	https://www.genecards.org/cgi-bin/carddisp.pl?gene=MIR202
PROK2	Prokineticin 2	Protein Coding	44	GC03M071771	4.796023	https://www.genecards.org/cgi-bin/carddisp.pl?gene=PROK2
E2F7	E2F Transcription Factor 7	Protein Coding	38	GC12M077021	4.793831	https://www.genecards.org/cgi-bin/carddisp.pl?gene=E2F7
DISC2	Disrupted In Schizophrenia 2	RNA Gene	19	GC01M231814	4.791626	https://www.genecards.org/cgi-bin/carddisp.pl?gene=DISC2
MCM7	Minichromosome Maintenance Complex Component 7	Protein Coding	45	GC07M100092	4.789493	https://www.genecards.org/cgi-bin/carddisp.pl?gene=MCM7
MAP2K5	Mitogen-Activated Protein Kinase Kinase 5	Protein Coding	47	GC15P077101	4.786411	https://www.genecards.org/cgi-bin/carddisp.pl?gene=MAP2K5
MAGI2-AS3	MAGI2 Antisense RNA 3	RNA Gene	16	GC07P079452	4.784665	https://www.genecards.org/cgi-bin/carddisp.pl?gene=MAGI2-AS3
NRG4	Neuregulin 4	Protein Coding	40	GC15M075935	4.78399	https://www.genecards.org/cgi-bin/carddisp.pl?gene=NRG4
ANXA9	Annexin A9	Protein Coding	39	GC01P150982	4.783628	https://www.genecards.org/cgi-bin/carddisp.pl?gene=ANXA9
RPS4X	Ribosomal Protein S4 X-Linked	Protein Coding	40	GC0XM072255	4.781923	https://www.genecards.org/cgi-bin/carddisp.pl?gene=RPS4X
ARID4B	AT-Rich Interaction Domain 4B	Protein Coding	37	GC01M235133	4.776742	https://www.genecards.org/cgi-bin/carddisp.pl?gene=ARID4B
CA1	Carbonic Anhydrase 1	Protein Coding	47	GC08M085327	4.775258	https://www.genecards.org/cgi-bin/carddisp.pl?gene=CA1
BBC3	BCL2 Binding Component 3	Protein Coding	41	GC19M047220	4.775219	https://www.genecards.org/cgi-bin/carddisp.pl?gene=BBC3
POMP	Proteasome Maturation Protein	Protein Coding	43	GC13P028659	4.772102	https://www.genecards.org/cgi-bin/carddisp.pl?gene=POMP

SERPINB7	Serpin Family B Member 7	Protein Coding	41	GC18P063752	4.769883	https://www.genecards.org/cgi-bin/carddisp.pl?gene=SERPINB7
WIF1	WNT Inhibitory Factor 1	Protein Coding	45	GC12M065050	4.769465	https://www.genecards.org/cgi-bin/carddisp.pl?gene=WIF1
EI24	EI24 Autophagy Associated Transmembrane Protein	Protein Coding	37	GC11P125570	4.764522	https://www.genecards.org/cgi-bin/carddisp.pl?gene=EI24
ZDHHC17	Zinc Finger DHHC-Type Palmitoyltransferase 17	Protein Coding	38	GC12P076763	4.758674	https://www.genecards.org/cgi-bin/carddisp.pl?gene=ZDHHC17
CABIN1	Calcineurin Binding Protein 1	Protein Coding	42	GC22P024011	4.75823	https://www.genecards.org/cgi-bin/carddisp.pl?gene=CABIN1
RPS3A	Ribosomal Protein S3A	Protein Coding	42	GC04P151099	4.758105	https://www.genecards.org/cgi-bin/carddisp.pl?gene=RPS3A
NPY4R	Neuropeptide Y Receptor Y4	Protein Coding	37	GC10M046461	4.756795	https://www.genecards.org/cgi-bin/carddisp.pl?gene=NPY4R
TNFRSF10D	TNF Receptor Superfamily Member 10d	Protein Coding	43	GC08M023135	4.756449	https://www.genecards.org/cgi-bin/carddisp.pl?gene=TNFRSF10D
SLC35B2	Solute Carrier Family 35 Member B2	Protein Coding	40	GC06M044254	4.751011	https://www.genecards.org/cgi-bin/carddisp.pl?gene=SLC35B2
TRAIP	TRAF Interacting Protein	Protein Coding	41	GC03M050057	4.748334	https://www.genecards.org/cgi-bin/carddisp.pl?gene=TRAIP
EPS15	Epidermal Growth Factor Receptor Pathway Substrate 15	Protein Coding	46	GC01M051354	4.747359	https://www.genecards.org/cgi-bin/carddisp.pl?gene=EPS15
TEKT3	Tektin 3	Protein Coding	35	GC17M015303	4.746962	https://www.genecards.org/cgi-bin/carddisp.pl?gene=TEKT3
PCM1	Pericentriolar Material 1	Protein Coding	43	GC08P017922	4.744633	https://www.genecards.org/cgi-bin/carddisp.pl?gene=PCM1
UBA52	Ubiquitin A-52 Residue Ribosomal Protein Fusion Product 1	Protein Coding	41	GC19P018563	4.743694	https://www.genecards.org/cgi-bin/carddisp.pl?gene=UBA52
RPL4	Ribosomal Protein L4	Protein Coding	43	GC15M066498	4.740825	https://www.genecards.org/cgi-bin/carddisp.pl?gene=RPL4
PLK4	Polo Like Kinase 4	Protein Coding	48	GC04P127880	4.740572	https://www.genecards.org/cgi-bin/carddisp.pl?gene=PLK4
INTS4	Integrator Complex Subunit 4	Protein Coding	37	GC11M077878	4.737488	https://www.genecards.org/cgi-bin/carddisp.pl?gene=INTS4
RPL8	Ribosomal Protein L8	Protein Coding	42	GC08M144821	4.734969	https://www.genecards.org/cgi-bin/carddisp.pl?gene=RPL8
TAS2R38	Taste 2 Receptor Member 38	Protein Coding	36	GC07M141972	4.734341	https://www.genecards.org/cgi-bin/carddisp.pl?gene=TAS2R38
STC1	Stanniocalcin 1	Protein Coding	41	GC08M023841	4.730952	https://www.genecards.org/cgi-bin/carddisp.pl?gene=STC1

PRRC2A	Proline Rich Coiled-Coil 2A	Protein Coding	33	GC06P055204	4.730647	https://www.genecards.org/cgi-bin/carddisp.pl?gene=PRRC2A
LDB1	LIM Domain Binding 1	Protein Coding	40	GC10M102106	4.727544	https://www.genecards.org/cgi-bin/carddisp.pl?gene=LDB1
HNRNPL	Heterogeneous Nuclear Ribonucleoprotein L	Protein Coding	40	GC19M038836	4.727404	https://www.genecards.org/cgi-bin/carddisp.pl?gene=HNRNPL
MAPKAPK5	MAPK Activated Protein Kinase 5	Protein Coding	45	GC12P111842	4.727202	https://www.genecards.org/cgi-bin/carddisp.pl?gene=MAPKAPK5
ANKS1A	Ankyrin Repeat And Sterile Alpha Motif Domain Containing 1A	Protein Coding	36	GC06P055319	4.726923	https://www.genecards.org/cgi-bin/carddisp.pl?gene=ANKS1A
AP2A2	Adaptor Related Protein Complex 2 Subunit Alpha 2	Protein Coding	38	GC11P000924	4.715266	https://www.genecards.org/cgi-bin/carddisp.pl?gene=AP2A2
RBBP4	RB Binding Protein 4, Chromatin Remodeling Factor	Protein Coding	44	GC01P032651	4.71513	https://www.genecards.org/cgi-bin/carddisp.pl?gene=RBBP4
MAL	Mal, T Cell Differentiation Protein	Protein Coding	38	GC02P095025	4.71438	https://www.genecards.org/cgi-bin/carddisp.pl?gene=MAL
C1QTNF1	C1q And TNF Related 1	Protein Coding	40	GC17P079022	4.713194	https://www.genecards.org/cgi-bin/carddisp.pl?gene=C1QTNF1
POLR3H	RNA Polymerase III Subunit H	Protein Coding	38	GC22M041525	4.711223	https://www.genecards.org/cgi-bin/carddisp.pl?gene=POLR3H
CCT4	Chaperonin Containing TCP1 Subunit 4	Protein Coding	40	GC02M061868	4.703442	https://www.genecards.org/cgi-bin/carddisp.pl?gene=CCT4
BICD1	BICD Cargo Adaptor 1	Protein Coding	40	GC12P032107	4.702754	https://www.genecards.org/cgi-bin/carddisp.pl?gene=BICD1
EIF2S2	Eukaryotic Translation Initiation Factor 2 Subunit Beta	Protein Coding	41	GC20M034088	4.700258	https://www.genecards.org/cgi-bin/carddisp.pl?gene=EIF2S2
SLC15A2	Solute Carrier Family 15 Member 2	Protein Coding	40	GC03P121894	4.698466	https://www.genecards.org/cgi-bin/carddisp.pl?gene=SLC15A2
BACH1	BTB Domain And CNC Homolog 1	Protein Coding	42	GC21P029194	4.694157	https://www.genecards.org/cgi-bin/carddisp.pl?gene=BACH1
SQLE	Squalene Epoxidase	Protein Coding	44	GC08P124998	4.693421	https://www.genecards.org/cgi-bin/carddisp.pl?gene=SQLE

HTR6	5-Hydroxytryptamine Receptor 6	Protein Coding	44	GC01P019666	4.6865	https://www.genecards.org/cgi-bin/carddisp.pl?gene=HTR6
PXYLP1	2-Phosphoxylose Phosphatase 1	Protein Coding	32	GC03P141228	4.681754	https://www.genecards.org/cgi-bin/carddisp.pl?gene=PXYLP1
PPP1R17	Protein Phosphatase 1 Regulatory Subunit 17	Protein Coding	38	GC07P031726	4.67995	https://www.genecards.org/cgi-bin/carddisp.pl?gene=PPP1R17
GLTP	Glycolipid Transfer Protein	Protein Coding	37	GC12M109850	4.679826	https://www.genecards.org/cgi-bin/carddisp.pl?gene=GLTP
TANK	TRAF Family Member Associated NFKB Activator	Protein Coding	44	GC02P161136	4.678485	https://www.genecards.org/cgi-bin/carddisp.pl?gene=TANK
RPS3	Ribosomal Protein S3	Protein Coding	45	GC11P076260	4.677998	https://www.genecards.org/cgi-bin/carddisp.pl?gene=RPS3
RANGAP1	Ran GTPase Activating Protein 1	Protein Coding	43	GC22M041244	4.677173	https://www.genecards.org/cgi-bin/carddisp.pl?gene=RANGAP1
EIF4A1	Eukaryotic Translation Initiation Factor 4A1	Protein Coding	45	GC17P007572	4.676231	https://www.genecards.org/cgi-bin/carddisp.pl?gene=EIF4A1
C4BPA	Complement Component 4 Binding Protein Alpha	Protein Coding	41	GC01P207105	4.665201	https://www.genecards.org/cgi-bin/carddisp.pl?gene=C4BPA
PDIA4	Protein Disulfide Isomerase Family A Member 4	Protein Coding	43	GC07M149003	4.662331	https://www.genecards.org/cgi-bin/carddisp.pl?gene=PDIA4
STK24	Serine/Threonine Kinase 24	Protein Coding	45	GC13M098445	4.662202	https://www.genecards.org/cgi-bin/carddisp.pl?gene=STK24
SCG2	Secretogranin II	Protein Coding	40	GC02M223596	4.661969	https://www.genecards.org/cgi-bin/carddisp.pl?gene=SCG2
PGAM5	PGAM Family Member 5, Mitochondrial Serine/Threonine Protein Phosphatase	Protein Coding	34	GC12P132710	4.66085	https://www.genecards.org/cgi-bin/carddisp.pl?gene=PGAM5
SMOC1	SPARC Related Modular Calcium Binding 1	Protein Coding	41	GC14P069854	4.660049	https://www.genecards.org/cgi-bin/carddisp.pl?gene=SMOC1
CDC16	Cell Division Cycle 16	Protein Coding	40	GC13P114234	4.659441	https://www.genecards.org/cgi-bin/carddisp.pl?gene=CDC16
AGFG1	ArfGAP With FG Repeats 1	Protein Coding	40	GC02P227473	4.659343	https://www.genecards.org/cgi-bin/carddisp.pl?gene=AGFG1
ANAPC1	Anaphase Promoting Complex Subunit 1	Protein Coding	41	GC02M111611	4.656997	https://www.genecards.org/cgi-bin/carddisp.pl?gene=ANAPC1

MDM4	MDM4 Regulator Of P53	Protein Coding	45	GC01P204516	4.656155	https://www.genecards.org/cgi-bin/carddisp.pl?gene=MDM4
RASIP1	Ras Interacting Protein 1	Protein Coding	37	GC19M048720	4.655036	https://www.genecards.org/cgi-bin/carddisp.pl?gene=RASIP1
CDR2	Cerebellar Degeneration Related Protein 2	Protein Coding	39	GC16M022357	4.650725	https://www.genecards.org/cgi-bin/carddisp.pl?gene=CDR2
MYO1D	Myosin ID	Protein Coding	40	GC17M032492	4.650517	https://www.genecards.org/cgi-bin/carddisp.pl?gene=MYO1D
CDC25B	Cell Division Cycle 25B	Protein Coding	47	GC20P003794	4.64867	https://www.genecards.org/cgi-bin/carddisp.pl?gene=CDC25B
USP1	Ubiquitin Specific Peptidase 1	Protein Coding	45	GC01P062436	4.647277	https://www.genecards.org/cgi-bin/carddisp.pl?gene=USP1
PSG1	Pregnancy Specific Beta-1-Glycoprotein 1	Protein Coding	39	GC19M042866	4.643477	https://www.genecards.org/cgi-bin/carddisp.pl?gene=PSG1
GART	Phosphoribosylglycinamide Formyltransferase, Phosphoribosylglycinamide Synthetase, Phosphoribosylaminoimidazole Synthetase	Protein Coding	43	GC21M033503	4.642777	https://www.genecards.org/cgi-bin/carddisp.pl?gene=GART
TFPT	TCF3 Fusion Partner	Protein Coding	36	GC19M054107	4.637411	https://www.genecards.org/cgi-bin/carddisp.pl?gene=TFPT
BAG6	BAG Cochaperone 6	Protein Coding	36	GC06M031639	4.63588	https://www.genecards.org/cgi-bin/carddisp.pl?gene=BAG6
UBE2D3	Ubiquitin Conjugating Enzyme E2 D3	Protein Coding	47	GC04M102794	4.634063	https://www.genecards.org/cgi-bin/carddisp.pl?gene=UBE2D3
AGR2	Anterior Gradient 2, Protein Disulphide Isomerase Family Member	Protein Coding	41	GC07M016892	4.630882	https://www.genecards.org/cgi-bin/carddisp.pl?gene=AGR2
TMBIM1	Transmembrane BAX Inhibitor Motif Containing 1	Protein Coding	36	GC02M218274	4.627502	https://www.genecards.org/cgi-bin/carddisp.pl?gene=TMBIM1
GANC	Glucosidase Alpha, Neutral C	Protein Coding	37	GC15P042273	4.622423	https://www.genecards.org/cgi-bin/carddisp.pl?gene=GANC
S1PR3	Sphingosine-1-Phosphate Receptor 3	Protein Coding	45	GC09P088991	4.62067	https://www.genecards.org/cgi-bin/carddisp.pl?gene=S1PR3
SLC24A5	Solute Carrier Family 24 Member 5	Protein Coding	41	GC15P048120	4.620585	https://www.genecards.org/cgi-bin/carddisp.pl?gene=SLC24A5

CD83	CD83 Molecule	Protein Coding	40	GC06P014117	4.618192	https://www.genecards.org/cgi-bin/carddisp.pl?gene=CD83
RBL2	RB Transcriptional Corepressor Like 2	Protein Coding	44	GC16P053433	4.617309	https://www.genecards.org/cgi-bin/carddisp.pl?gene=RBL2
SLC7A1	Solute Carrier Family 7 Member 1	Protein Coding	44	GC13M029509	4.615957	https://www.genecards.org/cgi-bin/carddisp.pl?gene=SLC7A1
RETSAT	Retinol Saturase	Protein Coding	38	GC02M085344	4.613746	https://www.genecards.org/cgi-bin/carddisp.pl?gene=RETSAT
GUCA2A	Guanylate Cyclase Activator 2A	Protein Coding	38	GC01M042162	4.610352	https://www.genecards.org/cgi-bin/carddisp.pl?gene=GUCA2A
TXNL1	Thioredoxin Like 1	Protein Coding	40	GC18M056597	4.604498	https://www.genecards.org/cgi-bin/carddisp.pl?gene=TXNL1
KPNA3	Karyopherin Subunit Alpha 3	Protein Coding	43	GC13M049699	4.601851	https://www.genecards.org/cgi-bin/carddisp.pl?gene=KPNA3
SETD1B	SET Domain Containing 1B, Histone Lysine Methyltransferase	Protein Coding	37	GC12P123586	4.599613	https://www.genecards.org/cgi-bin/carddisp.pl?gene=SETD1B
MED24	Mediator Complex Subunit 24	Protein Coding	37	GC17M040019	4.599255	https://www.genecards.org/cgi-bin/carddisp.pl?gene=MED24
STX17	Syntaxin 17	Protein Coding	36	GC09P099906	4.597912	https://www.genecards.org/cgi-bin/carddisp.pl?gene=STX17
NKX2-8	NK2 Homeobox 8	Protein Coding	37	GC14M036580	4.597596	https://www.genecards.org/cgi-bin/carddisp.pl?gene=NKX2-8
SLC9A3R2	SLC9A3 Regulator 2	Protein Coding	40	GC16P005577	4.597387	https://www.genecards.org/cgi-bin/carddisp.pl?gene=SLC9A3R2
SNX6	Sorting Nexin 6	Protein Coding	37	GC14M034561	4.591009	https://www.genecards.org/cgi-bin/carddisp.pl?gene=SNX6
SEL1L3	SEL1L Family Member 3	Protein Coding	33	GC04M025715	4.589505	https://www.genecards.org/cgi-bin/carddisp.pl?gene=SEL1L3
DHRS11	Dehydrogenase/Reductase 11	Protein Coding	37	GC17P036888	4.587685	https://www.genecards.org/cgi-bin/carddisp.pl?gene=DHRS11
AGAP1	ArfGAP With GTPase Domain, Ankyrin Repeat And PH Domain 1	Protein Coding	41	GC02P235494	4.579213	https://www.genecards.org/cgi-bin/carddisp.pl?gene=AGAP1
PEDS1	Plasmanylethanolamine Desaturase 1	Protein Coding	26	GC20M050119	4.572634	https://www.genecards.org/cgi-bin/carddisp.pl?gene=PEDS1
PDK1	Pyruvate Dehydrogenase Kinase 1	Protein Coding	47	GC02P172555	4.572374	https://www.genecards.org/cgi-bin/carddisp.pl?gene=PDK1
RUVBL1	RuvB Like AAA ATPase 1	Protein Coding	45	GC03M128064	4.571053	https://www.genecards.org/cgi-bin/carddisp.pl?gene=RUVBL1
RPS13	Ribosomal Protein S13	Protein Coding	43	GC11M017274	4.570422	https://www.genecards.org/cgi-bin/carddisp.pl?gene=RPS13

MED15	Mediator Complex Subunit 15	Protein Coding	40	GC22P020506	4.567909	https://www.genecards.org/cgi-bin/carddisp.pl?gene=MED15
COPB1	COPI Coat Complex Subunit Beta 1	Protein Coding	39	GC11M014436	4.564624	https://www.genecards.org/cgi-bin/carddisp.pl?gene=COPB1
SLC25A2	Solute Carrier Family 25 Member 2	Protein Coding	34	GC05M141302	4.563865	https://www.genecards.org/cgi-bin/carddisp.pl?gene=SLC25A2
PCYT2	Phosphate Cytidylyltransferase 2, Ethanolamine	Protein Coding	43	GC17M081900	4.562902	https://www.genecards.org/cgi-bin/carddisp.pl?gene=PCYT2
SFXN4	Sideroflexin 4	Protein Coding	41	GC10M119140	4.554611	https://www.genecards.org/cgi-bin/carddisp.pl?gene=SFXN4
CEBPD	CCAAT Enhancer Binding Protein Delta	Protein Coding	38	GC08M047759	4.550325	https://www.genecards.org/cgi-bin/carddisp.pl?gene=CEBPD
TACC2	Transforming Acidic Coiled-Coil Containing Protein 2	Protein Coding	38	GC10P121989	4.545766	https://www.genecards.org/cgi-bin/carddisp.pl?gene=TACC2
EXOSC10	Exosome Component 10	Protein Coding	39	GC01M011067	4.542109	https://www.genecards.org/cgi-bin/carddisp.pl?gene=EXOSC10
CADM2	Cell Adhesion Molecule 2	Protein Coding	37	GC03P085008	4.54075	https://www.genecards.org/cgi-bin/carddisp.pl?gene=CADM2
COX6C	Cytochrome C Oxidase Subunit 6C	Protein Coding	40	GC08M099899	4.54062	https://www.genecards.org/cgi-bin/carddisp.pl?gene=COX6C
CCNT1	Cyclin T1	Protein Coding	41	GC12M048688	4.539225	https://www.genecards.org/cgi-bin/carddisp.pl?gene=CCNT1
WASL	WASP Like Actin Nucleation Promoting Factor	Protein Coding	43	GC07M123681	4.538745	https://www.genecards.org/cgi-bin/carddisp.pl?gene=WASL
SNRPD2	Small Nuclear Ribonucleoprotein D2 Polypeptide	Protein Coding	41	GC19M046999	4.537465	https://www.genecards.org/cgi-bin/carddisp.pl?gene=SNRPD2
LOC110366354	CYP3A4 5' Regulatory Region	Biological Region	1	GC07P099784	4.537452	https://www.genecards.org/cgi-bin/carddisp.pl?gene=LOC110366354
PDE7B	Phosphodiesterase 7B	Protein Coding	39	GC06P135795	4.536767	https://www.genecards.org/cgi-bin/carddisp.pl?gene=PDE7B
MANF	Mesencephalic Astrocyte Derived Neurotrophic Factor	Protein Coding	40	GC03P051385	4.53442	https://www.genecards.org/cgi-bin/carddisp.pl?gene=MANF
BCAS3	BCAS3 Microtubule Associated Cell Migration Factor	Protein Coding	37	GC17P060678	4.533676	https://www.genecards.org/cgi-bin/carddisp.pl?gene=BCAS3

NSMF	NMDA Receptor Synaptonuclear Signaling And Neuronal Migration Factor	Protein Coding	38	GC09M137447	4.533435	https://www.genecards.org/cgi-bin/carddisp.pl?gene=NSMF
ACTR2	Actin Related Protein 2	Protein Coding	44	GC02P065227	4.530648	https://www.genecards.org/cgi-bin/carddisp.pl?gene=ACTR2
ALPK1	Alpha Kinase 1	Protein Coding	40	GC04P112285	4.528509	https://www.genecards.org/cgi-bin/carddisp.pl?gene=ALPK1
TRIM22	Tripartite Motif Containing 22	Protein Coding	40	GC11P005689	4.528236	https://www.genecards.org/cgi-bin/carddisp.pl?gene=TRIM22
MIR198	MicroRNA 198	RNA Gene	16	GC03M120395	4.52667	https://www.genecards.org/cgi-bin/carddisp.pl?gene=MIR198
NDUFB4	NADH:Ubiquinone Oxidoreductase Subunit B4	Protein Coding	41	GC03P120596	4.522985	https://www.genecards.org/cgi-bin/carddisp.pl?gene=NDUFB4
CST6	Cystatin E/M	Protein Coding	40	GC11P066311	4.521043	https://www.genecards.org/cgi-bin/carddisp.pl?gene=CST6
FAT1	FAT Atypical Cadherin 1	Protein Coding	40	GC04M186587	4.509996	https://www.genecards.org/cgi-bin/carddisp.pl?gene=FAT1
MTIF2	Mitochondrial Translational Initiation Factor 2	Protein Coding	40	GC02M055236	4.509862	https://www.genecards.org/cgi-bin/carddisp.pl?gene=MTIF2
FIBP	FGF1 Intracellular Binding Protein	Protein Coding	41	GC11M069430	4.509194	https://www.genecards.org/cgi-bin/carddisp.pl?gene=FIBP
NUCB2	Nucleobindin 2	Protein Coding	39	GC11P017237	4.508267	https://www.genecards.org/cgi-bin/carddisp.pl?gene=NUCB2
MBD2	Methyl-CpG Binding Domain Protein 2	Protein Coding	41	GC18M054151	4.506716	https://www.genecards.org/cgi-bin/carddisp.pl?gene=MBD2
CAND1	Cullin Associated And Neddylation Dissociated 1	Protein Coding	39	GC12P067270	4.506351	https://www.genecards.org/cgi-bin/carddisp.pl?gene=CAND1
PTPRN2	Protein Tyrosine Phosphatase Receptor Type N2	Protein Coding	45	GC07M157539	4.505263	https://www.genecards.org/cgi-bin/carddisp.pl?gene=PTPRN2
PPP1CC	Protein Phosphatase 1 Catalytic Subunit Gamma	Protein Coding	46	GC12M110709	4.50234	https://www.genecards.org/cgi-bin/carddisp.pl?gene=PPP1CC
CDR1-AS	CDR1 Antisense RNA	RNA Gene	6	GC0XU902169	4.502142	https://www.genecards.org/cgi-bin/carddisp.pl?gene=CDR1-AS
MACROD1	Mono-ADP Ribosylhydrolase 1	Protein Coding	34	GC11M063998	4.498342	https://www.genecards.org/cgi-bin/carddisp.pl?gene=MACROD1

YLPM1	YLP Motif Containing 1	Protein Coding	33	GC14P074763	4.498254	https://www.genecards.org/cgi-bin/carddisp.pl?gene=YLPM1
TRIM24	Tripartite Motif Containing 24	Protein Coding	44	GC07P138460	4.497521	https://www.genecards.org/cgi-bin/carddisp.pl?gene=TRIM24
POLR2D	RNA Polymerase II Subunit D	Protein Coding	43	GC02M128042	4.497245	https://www.genecards.org/cgi-bin/carddisp.pl?gene=POLR2D
TNRC6B	Trinucleotide Repeat Containing Adaptor 6B	Protein Coding	37	GC22P040044	4.496908	https://www.genecards.org/cgi-bin/carddisp.pl?gene=TNRC6B
XAB2	XPA Binding Protein 2	Protein Coding	38	GC19M007619	4.493793	https://www.genecards.org/cgi-bin/carddisp.pl?gene=XAB2
RSRC1	Arginine And Serine Rich Coiled-Coil 1	Protein Coding	40	GC03P158105	4.492593	https://www.genecards.org/cgi-bin/carddisp.pl?gene=RSRC1
RPL24	Ribosomal Protein L24	Protein Coding	40	GC03M101681	4.49227	https://www.genecards.org/cgi-bin/carddisp.pl?gene=RPL24
APPL2	Adaptor Protein, Phosphotyrosine Interacting With PH Domain And Leucine Zipper 2	Protein Coding	36	GC12M105173	4.491245	https://www.genecards.org/cgi-bin/carddisp.pl?gene=APPL2
IL6-AS1	IL6 Antisense RNA 1	RNA Gene	11	GC07M022728	4.488552	https://www.genecards.org/cgi-bin/carddisp.pl?gene=IL6-AS1
RNF125	Ring Finger Protein 125	Protein Coding	42	GC18P032025	4.486342	https://www.genecards.org/cgi-bin/carddisp.pl?gene=RNF125
EIF4B	Eukaryotic Translation Initiation Factor 4B	Protein Coding	43	GC12P053006	4.486102	https://www.genecards.org/cgi-bin/carddisp.pl?gene=EIF4B
CRLS1	Cardiolipin Synthase 1	Protein Coding	40	GC20P006005	4.481825	https://www.genecards.org/cgi-bin/carddisp.pl?gene=CRLS1
ENHO	Energy Homeostasis Associated	Protein Coding	30	GC09M034516	4.481272	https://www.genecards.org/cgi-bin/carddisp.pl?gene=ENHO
GCNT2	Glucosaminyl (N-Acetyl) Transferase 2 (I Blood Group)	Protein Coding	45	GC06P010492	4.479461	https://www.genecards.org/cgi-bin/carddisp.pl?gene=GCNT2
LEPROT	Leptin Receptor Overlapping Transcript	Protein Coding	33	GC01P065420	4.47036	https://www.genecards.org/cgi-bin/carddisp.pl?gene=LEPROT
RDH13	Retinol Dehydrogenase 13	Protein Coding	40	GC19M055039	4.467848	https://www.genecards.org/cgi-bin/carddisp.pl?gene=RDH13
MYCBP2	MYC Binding Protein 2	Protein Coding	38	GC13M077044	4.46719	https://www.genecards.org/cgi-bin/carddisp.pl?gene=MYCBP2
ARHGAP24	Rho GTPase Activating Protein 24	Protein Coding	40	GC04P085475	4.464039	https://www.genecards.org/cgi-bin/carddisp.pl?gene=ARHGAP24
MED17	Mediator Complex Subunit 17	Protein Coding	41	GC11P093784	4.463892	https://www.genecards.org/cgi-bin/carddisp.pl?gene=MED17

CISD1	CDGSH Iron Sulfur Domain 1	Protein Coding	39	GC10P058269	4.463004	https://www.genecards.org/cgi-bin/carddisp.pl?gene=CISD1
SMURF1	SMAD Specific E3 Ubiquitin Protein Ligase 1	Protein Coding	45	GC07M099027	4.457187	https://www.genecards.org/cgi-bin/carddisp.pl?gene=SMURF1
NUFIP1	Nuclear FMR1 Interacting Protein 1	Protein Coding	35	GC13M044939	4.456027	https://www.genecards.org/cgi-bin/carddisp.pl?gene=NUFIP1
EDC4	Enhancer Of MRNA Decapping 4	Protein Coding	39	GC16P067873	4.453725	https://www.genecards.org/cgi-bin/carddisp.pl?gene=EDC4
KIFC3	Kinesin Family Member C3	Protein Coding	39	GC16M057758	4.44997	https://www.genecards.org/cgi-bin/carddisp.pl?gene=KIFC3
ITSN1	Intersectin 1	Protein Coding	42	GC21P033642	4.44868	https://www.genecards.org/cgi-bin/carddisp.pl?gene=ITSN1
POLR1H	RNA Polymerase I Subunit H	Protein Coding	32	GC06P057843	4.445566	https://www.genecards.org/cgi-bin/carddisp.pl?gene=POLR1H
CD74	CD74 Molecule	Protein Coding	44	GC05M150378	4.44493	https://www.genecards.org/cgi-bin/carddisp.pl?gene=CD74
EGLN2	Egl-9 Family Hypoxia Inducible Factor 2	Protein Coding	45	GC19P041111	4.436359	https://www.genecards.org/cgi-bin/carddisp.pl?gene=EGLN2
CCT6A	Chaperonin Containing TCP1 Subunit 6A	Protein Coding	40	GC07P056051	4.434839	https://www.genecards.org/cgi-bin/carddisp.pl?gene=CCT6A
C5AR2	Complement Component 5a Receptor 2	Protein Coding	40	GC19P047333	4.431859	https://www.genecards.org/cgi-bin/carddisp.pl?gene=C5AR2
SENP3	SUMO Specific Peptidase 3	Protein Coding	40	GC17P009106	4.430599	https://www.genecards.org/cgi-bin/carddisp.pl?gene=SENP3
ABCF1	ATP Binding Cassette Subfamily F Member 1	Protein Coding	40	GC06P030571	4.429214	https://www.genecards.org/cgi-bin/carddisp.pl?gene=ABCF1
RAB11B	RAB11B, Member RAS Oncogene Family	Protein Coding	43	GC19P008393	4.428178	https://www.genecards.org/cgi-bin/carddisp.pl?gene=RAB11B
SEMA3E	Semaphorin 3E	Protein Coding	43	GC07M083363	4.42813	https://www.genecards.org/cgi-bin/carddisp.pl?gene=SEMA3E
HAPLN1	Hyaluronan And Proteoglycan Link Protein 1	Protein Coding	43	GC05M083637	4.427074	https://www.genecards.org/cgi-bin/carddisp.pl?gene=HAPLN1
PGC	Progastricsin	Protein Coding	43	GC06M041736	4.424487	https://www.genecards.org/cgi-bin/carddisp.pl?gene=PGC
CDT1	Chromatin Licensing And DNA Replication Factor 1	Protein Coding	44	GC16P088803	4.422527	https://www.genecards.org/cgi-bin/carddisp.pl?gene=CDT1

NEDD8	NEDD8 Ubiquitin Like Modifier	Protein Coding	41	GC14M024216	4.41927	https://www.genecards.org/cgi-bin/carddisp.pl?gene=NEDD8
NCOA4	Nuclear Receptor Coactivator 4	Protein Coding	40	GC10M046005	4.417659	https://www.genecards.org/cgi-bin/carddisp.pl?gene=NCOA4
EIF4A3	Eukaryotic Translation Initiation Factor 4A3	Protein Coding	44	GC17M080135	4.417566	https://www.genecards.org/cgi-bin/carddisp.pl?gene=EIF4A3
MAN2C1	Mannosidase Alpha Class 2C Member 1	Protein Coding	38	GC15M075355	4.417305	https://www.genecards.org/cgi-bin/carddisp.pl?gene=MAN2C1
ARRDC3	Arrestin Domain Containing 3	Protein Coding	38	GC05M091368	4.414473	https://www.genecards.org/cgi-bin/carddisp.pl?gene=ARRDC3
EREG	Epiregulin	Protein Coding	42	GC04P074366	4.414438	https://www.genecards.org/cgi-bin/carddisp.pl?gene=EREG
DDX60L	DExD/H-Box 60 Like	Protein Coding	33	GC04M168356	4.411598	https://www.genecards.org/cgi-bin/carddisp.pl?gene=DDX60L
CEPT1	Choline/Ethanolamine Phosphotransferase 1	Protein Coding	40	GC01P111139	4.408087	https://www.genecards.org/cgi-bin/carddisp.pl?gene=CEPT1
SMPD4	Sphingomyelin Phosphodiesterase 4	Protein Coding	38	GC02M130151	4.40805	https://www.genecards.org/cgi-bin/carddisp.pl?gene=SMPD4
INO80	INO80 Complex ATPase Subunit	Protein Coding	37	GC15M040979	4.404651	https://www.genecards.org/cgi-bin/carddisp.pl?gene=INO80
SERPINB6	Serpin Family B Member 6	Protein Coding	44	GC06M002948	4.401329	https://www.genecards.org/cgi-bin/carddisp.pl?gene=SERPINB6
G0S2	G0/G1 Switch 2	Protein Coding	33	GC01P209675	4.398935	https://www.genecards.org/cgi-bin/carddisp.pl?gene=G0S2
FOXK1	Forkhead Box K1	Protein Coding	35	GC07P004682	4.395694	https://www.genecards.org/cgi-bin/carddisp.pl?gene=FOXK1
MTARC1	Mitochondrial Amidoxime Reducing Component 1	Protein Coding	28	GC01P220788	4.391452	https://www.genecards.org/cgi-bin/carddisp.pl?gene=MTARC1
CILP2	Cartilage Intermediate Layer Protein 2	Protein Coding	37	GC19P019538	4.386812	https://www.genecards.org/cgi-bin/carddisp.pl?gene=CILP2
PPP1R14A	Protein Phosphatase 1 Regulatory Inhibitor Subunit 14A	Protein Coding	41	GC19M038251	4.384929	https://www.genecards.org/cgi-bin/carddisp.pl?gene=PPP1R14A
NOVA1	NOVA Alternative Splicing Regulator 1	Protein Coding	40	GC14M026443	4.384608	https://www.genecards.org/cgi-bin/carddisp.pl?gene=NOVA1
EFHD1	EF-Hand Domain Family Member D1	Protein Coding	36	GC02P232606	4.383911	https://www.genecards.org/cgi-bin/carddisp.pl?gene=EFHD1
MIR124-3	MicroRNA 124-3	RNA Gene	18	GC20P063180	4.382467	https://www.genecards.org/cgi-bin/carddisp.pl?gene=MIR124-3

EIF3A	Eukaryotic Translation Initiation Factor 3 Subunit A	Protein Coding	43	GC10M119034	4.37745	https://www.genecards.org/cgi-bin/carddisp.pl?gene=EIF3A
ROR1	Receptor Tyrosine Kinase Like Orphan Receptor 1	Protein Coding	48	GC01P063774	4.374794	https://www.genecards.org/cgi-bin/carddisp.pl?gene=ROR1
SCARF2	Scavenger Receptor Class F Member 2	Protein Coding	41	GC22M020424	4.37427	https://www.genecards.org/cgi-bin/carddisp.pl?gene=SCARF2
PPAT	Phosphoribosyl Pyrophosphate Amidotransferase	Protein Coding	44	GC04M056393	4.370205	https://www.genecards.org/cgi-bin/carddisp.pl?gene=PPAT
MIR199A2	MicroRNA 199a-2	RNA Gene	19	GC01M172235	4.361644	https://www.genecards.org/cgi-bin/carddisp.pl?gene=MIR199A2
POU3F1	POU Class 3 Homeobox 1	Protein Coding	37	GC01M038044	4.356398	https://www.genecards.org/cgi-bin/carddisp.pl?gene=POU3F1
TP53BP2	Tumor Protein P53 Binding Protein 2	Protein Coding	43	GC01M223779	4.355329	https://www.genecards.org/cgi-bin/carddisp.pl?gene=TP53BP2
CCDC63	Coiled-Coil Domain Containing 63	Protein Coding	34	GC12P110846	4.354627	https://www.genecards.org/cgi-bin/carddisp.pl?gene=CCDC63
ACBD4	Acyl-CoA Binding Domain Containing 4	Protein Coding	38	GC17P045132	4.351822	https://www.genecards.org/cgi-bin/carddisp.pl?gene=ACBD4
NDUFB5	NADH:Ubiquinone Oxidoreductase Subunit B5	Protein Coding	37	GC03P179604	4.350846	https://www.genecards.org/cgi-bin/carddisp.pl?gene=NDUFB5
TNFSF14	TNF Superfamily Member 14	Protein Coding	41	GC19M006663	4.349636	https://www.genecards.org/cgi-bin/carddisp.pl?gene=TNFSF14
RBMS3	RNA Binding Motif Single Stranded Interacting Protein 3	Protein Coding	36	GC03P028575	4.341599	https://www.genecards.org/cgi-bin/carddisp.pl?gene=RBMS3
SRSF7	Serine And Arginine Rich Splicing Factor 7	Protein Coding	40	GC02M038743	4.3395	https://www.genecards.org/cgi-bin/carddisp.pl?gene=SRSF7
IGLON5	IgLON Family Member 5	Protein Coding	33	GC19P051311	4.339272	https://www.genecards.org/cgi-bin/carddisp.pl?gene=IGLON5
MIR31HG	MIR31 Host Gene	RNA Gene	19	GC09M021439	4.33896	https://www.genecards.org/cgi-bin/carddisp.pl?gene=MIR31HG
PKNOX1	PBX/Knotted 1 Homeobox 1	Protein Coding	41	GC21P042974	4.336639	https://www.genecards.org/cgi-bin/carddisp.pl?gene=PKNOX1
MC3R	Melanocortin 3 Receptor	Protein Coding	44	GC20P056248	4.334254	https://www.genecards.org/cgi-bin/carddisp.pl?gene=MC3R

GNG12	G Protein Subunit Gamma 12	Protein Coding	42	GC01M067701	4.333367	https://www.genecards.org/cgi-bin/carddisp.pl?gene=GNG12
KIF14	Kinesin Family Member 14	Protein Coding	40	GC01M200551	4.330287	https://www.genecards.org/cgi-bin/carddisp.pl?gene=KIF14
PATJ	PATJ Crumbs Cell Polarity Complex Component	Protein Coding	32	GC01P061743	4.320234	https://www.genecards.org/cgi-bin/carddisp.pl?gene=PATJ
PIP5K1B	Phosphatidylinositol-4-Phosphate 5-Kinase Type 1 Beta	Protein Coding	44	GC09P068705	4.318393	https://www.genecards.org/cgi-bin/carddisp.pl?gene=PIP5K1B
POLR2E	RNA Polymerase II, I And III Subunit E	Protein Coding	44	GC19M001086	4.318276	https://www.genecards.org/cgi-bin/carddisp.pl?gene=POLR2E
BAG2	BAG Cochaperone 2	Protein Coding	41	GC06P057172	4.317065	https://www.genecards.org/cgi-bin/carddisp.pl?gene=BAG2
UQCRH	Ubiquinol-Cytochrome C Reductase Hinge Protein	Protein Coding	38	GC01P046303	4.313158	https://www.genecards.org/cgi-bin/carddisp.pl?gene=UQCRH
PYCR2	Pyrroline-5-Carboxylate Reductase 2	Protein Coding	45	GC01M225919	4.312352	https://www.genecards.org/cgi-bin/carddisp.pl?gene=PYCR2
PTPN13	Protein Tyrosine Phosphatase Non-Receptor Type 13	Protein Coding	44	GC04P086594	4.306979	https://www.genecards.org/cgi-bin/carddisp.pl?gene=PTPN13
TUBA1C	Tubulin Alpha 1c	Protein Coding	41	GC12P049188	4.306669	https://www.genecards.org/cgi-bin/carddisp.pl?gene=TUBA1C
NEPRO	Nucleolus And Neural Progenitor Protein	Protein Coding	31	GC03M113009	4.305442	https://www.genecards.org/cgi-bin/carddisp.pl?gene=NEPRO
NDUFB2	NADH:Ubiquinone Oxidoreductase Subunit B2	Protein Coding	40	GC07P140690	4.303535	https://www.genecards.org/cgi-bin/carddisp.pl?gene=NDUFB2
REEP3	Receptor Accessory Protein 3	Protein Coding	37	GC10P063521	4.297606	https://www.genecards.org/cgi-bin/carddisp.pl?gene=REEP3
DKK3	Dickkopf WNT Signaling Pathway Inhibitor 3	Protein Coding	41	GC11M011962	4.296789	https://www.genecards.org/cgi-bin/carddisp.pl?gene=DKK3
RFK	Riboflavin Kinase	Protein Coding	40	GC09M076385	4.295729	https://www.genecards.org/cgi-bin/carddisp.pl?gene=RFK
AP1G1	Adaptor Related Protein Complex 1 Subunit Gamma 1	Protein Coding	41	GC16M071729	4.295501	https://www.genecards.org/cgi-bin/carddisp.pl?gene=AP1G1

KLK10	Kallikrein Related Peptidase 10	Protein Coding	40	GC19M051012	4.29087	https://www.genecards.org/cgi-bin/carddisp.pl?gene=KLK10
MIR873	MicroRNA 873	RNA Gene	16	GC09M028880	4.289502	https://www.genecards.org/cgi-bin/carddisp.pl?gene=MIR873
MAP4	Microtubule Associated Protein 4	Protein Coding	42	GC03M047850	4.288494	https://www.genecards.org/cgi-bin/carddisp.pl?gene=MAP4
UBAC1	UBA Domain Containing 1	Protein Coding	37	GC09M135932	4.288327	https://www.genecards.org/cgi-bin/carddisp.pl?gene=UBAC1
OSBPL5	Oxysterol Binding Protein Like 5	Protein Coding	40	GC11M003088	4.28806	https://www.genecards.org/cgi-bin/carddisp.pl?gene=OSBPL5
NPY1R	Neuropeptide Y Receptor Y1	Protein Coding	47	GC04M163323	4.286477	https://www.genecards.org/cgi-bin/carddisp.pl?gene=NPY1R
CDC27	Cell Division Cycle 27	Protein Coding	42	GC17M047117	4.285986	https://www.genecards.org/cgi-bin/carddisp.pl?gene=CDC27
GTPBP4	GTP Binding Protein 4	Protein Coding	39	GC10P000988	4.285235	https://www.genecards.org/cgi-bin/carddisp.pl?gene=GTPBP4
APOL2	Apolipoprotein L2	Protein Coding	40	GC22M036226	4.280738	https://www.genecards.org/cgi-bin/carddisp.pl?gene=APOL2
GMPR	Guanosine Monophosphate Reductase	Protein Coding	44	GC06P016238	4.278691	https://www.genecards.org/cgi-bin/carddisp.pl?gene=GMPR
PDCD6IP	Programmed Cell Death 6 Interacting Protein	Protein Coding	44	GC03P033798	4.274946	https://www.genecards.org/cgi-bin/carddisp.pl?gene=PDCD6IP
PPME1	Protein Phosphatase Methylesterase 1	Protein Coding	39	GC11P074170	4.268126	https://www.genecards.org/cgi-bin/carddisp.pl?gene=PPME1
HSPA14	Heat Shock Protein Family A (Hsp70) Member 14	Protein Coding	40	GC10P014847	4.266142	https://www.genecards.org/cgi-bin/carddisp.pl?gene=HSPA14
MCF2L2	MCF.2 Cell Line Derived Transforming Sequence-Like 2	Protein Coding	34	GC03M183183	4.262735	https://www.genecards.org/cgi-bin/carddisp.pl?gene=MCF2L2
CUL7	Cullin 7	Protein Coding	43	GC06M043037	4.261546	https://www.genecards.org/cgi-bin/carddisp.pl?gene=CUL7
SLC30A6	Solute Carrier Family 30 Member 6	Protein Coding	39	GC02P032166	4.261125	https://www.genecards.org/cgi-bin/carddisp.pl?gene=SLC30A6
AP2A1	Adaptor Related Protein Complex 2 Subunit Alpha 1	Protein Coding	40	GC19P049766	4.259474	https://www.genecards.org/cgi-bin/carddisp.pl?gene=AP2A1
MTCH2	Mitochondrial Carrier 2	Protein Coding	41	GC11M047604	4.258552	https://www.genecards.org/cgi-bin/carddisp.pl?gene=MTCH2

NPLOC4	NPL4 Homolog, Ubiquitin Recognition Factor	Protein Coding	40	GC17M081556	4.258315	https://www.genecards.org/cgi-bin/carddisp.pl?gene=NPLOC4
IMPDH2	Inosine Monophosphate Dehydrogenase 2	Protein Coding	47	GC03M049439	4.256704	https://www.genecards.org/cgi-bin/carddisp.pl?gene=IMPDH2
RANBP1	RAN Binding Protein 1	Protein Coding	44	GC22P020115	4.254214	https://www.genecards.org/cgi-bin/carddisp.pl?gene=RANBP1
RPL34	Ribosomal Protein L34	Protein Coding	40	GC04P108620	4.252873	https://www.genecards.org/cgi-bin/carddisp.pl?gene=RPL34
H2AZ1	H2A.Z Variant Histone 1	Protein Coding	34	GC04M099949	4.25223	https://www.genecards.org/cgi-bin/carddisp.pl?gene=H2AZ1
RBM14	RNA Binding Motif Protein 14	Protein Coding	37	GC11P066785	4.250051	https://www.genecards.org/cgi-bin/carddisp.pl?gene=RBM14
PCDH7	Protocadherin 7	Protein Coding	40	GC04P030722	4.24526	https://www.genecards.org/cgi-bin/carddisp.pl?gene=PCDH7
MTA2	Metastasis Associated 1 Family Member 2	Protein Coding	40	GC11M069248	4.24508	https://www.genecards.org/cgi-bin/carddisp.pl?gene=MTA2
H2BS1	H2B.S Histone 1	Protein Coding	23	GC21P043567	4.242882	https://www.genecards.org/cgi-bin/carddisp.pl?gene=H2BS1
NTM	Neurotrimin	Protein Coding	41	GC11P131370	4.242783	https://www.genecards.org/cgi-bin/carddisp.pl?gene=NTM
ARHGEF1	Rho Guanine Nucleotide Exchange Factor 1	Protein Coding	47	GC19P041883	4.241294	https://www.genecards.org/cgi-bin/carddisp.pl?gene=ARHGEF1
OSBPL11	Oxysterol Binding Protein Like 11	Protein Coding	36	GC03M125529	4.23335	https://www.genecards.org/cgi-bin/carddisp.pl?gene=OSBPL11
SLC6A11	Solute Carrier Family 6 Member 11	Protein Coding	43	GC03P011027	4.232661	https://www.genecards.org/cgi-bin/carddisp.pl?gene=SLC6A11
FBXO42	F-Box Protein 42	Protein Coding	34	GC01M016292	4.231699	https://www.genecards.org/cgi-bin/carddisp.pl?gene=FBXO42
DAGLB	Diacylglycerol Lipase Beta	Protein Coding	40	GC07M006416	4.229289	https://www.genecards.org/cgi-bin/carddisp.pl?gene=DAGLB
PIAS3	Protein Inhibitor Of Activated STAT 3	Protein Coding	41	GC01M145848	4.226283	https://www.genecards.org/cgi-bin/carddisp.pl?gene=PIAS3
UBE2I	Ubiquitin Conjugating Enzyme E2 I	Protein Coding	48	GC16P005523	4.225298	https://www.genecards.org/cgi-bin/carddisp.pl?gene=UBE2I
STMP1	Short Transmembrane Mitochondrial Protein 1	Protein Coding	22	GC07P135671	4.223236	https://www.genecards.org/cgi-bin/carddisp.pl?gene=STMP1
CLEC4E	C-Type Lectin Domain Family 4 Member E	Protein Coding	37	GC12M008535	4.219957	https://www.genecards.org/cgi-bin/carddisp.pl?gene=CLEC4E

ACTR3	Actin Related Protein 3	Protein Coding	43	GC02P113889	4.219325	https://www.genecards.org/cgi-bin/carddisp.pl?gene=ACTR3
TRIM39	Tripartite Motif Containing 39	Protein Coding	38	GC06P055171	4.217925	https://www.genecards.org/cgi-bin/carddisp.pl?gene=TRIM39
STIM2	Stromal Interaction Molecule 2	Protein Coding	40	GC04P026859	4.214132	https://www.genecards.org/cgi-bin/carddisp.pl?gene=STIM2
BRD1	Bromodomain Containing 1	Protein Coding	41	GC22M049773	4.214014	https://www.genecards.org/cgi-bin/carddisp.pl?gene=BRD1
SLC38A2	Solute Carrier Family 38 Member 2	Protein Coding	43	GC12M046358	4.211423	https://www.genecards.org/cgi-bin/carddisp.pl?gene=SLC38A2
MAP3K2	Mitogen-Activated Protein Kinase Kinase Kinase 2	Protein Coding	45	GC02M127298	4.208899	https://www.genecards.org/cgi-bin/carddisp.pl?gene=MAP3K2
DNAJC14	DnaJ Heat Shock Protein Family (Hsp40) Member C14	Protein Coding	34	GC12M055820	4.208716	https://www.genecards.org/cgi-bin/carddisp.pl?gene=DNAJC14
PSMD13	Proteasome 26S Subunit, Non-ATPase 13	Protein Coding	41	GC11P000236	4.207955	https://www.genecards.org/cgi-bin/carddisp.pl?gene=PSMD13
DFFA	DNA Fragmentation Factor Subunit Alpha	Protein Coding	45	GC01M010456	4.207635	https://www.genecards.org/cgi-bin/carddisp.pl?gene=DFFA
PTPRE	Protein Tyrosine Phosphatase Receptor Type E	Protein Coding	41	GC10P127907	4.204255	https://www.genecards.org/cgi-bin/carddisp.pl?gene=PTPRE
GJB5	Gap Junction Protein Beta 5	Protein Coding	41	GC01P034755	4.201731	https://www.genecards.org/cgi-bin/carddisp.pl?gene=GJB5
MYLIP	Myosin Regulatory Light Chain Interacting Protein	Protein Coding	40	GC06P016129	4.198773	https://www.genecards.org/cgi-bin/carddisp.pl?gene=MYLIP
ELP3	Elongator Acetyltransferase Complex Subunit 3	Protein Coding	40	GC08P028089	4.196687	https://www.genecards.org/cgi-bin/carddisp.pl?gene=ELP3
SIK2	Salt Inducible Kinase 2	Protein Coding	44	GC11P111633	4.194995	https://www.genecards.org/cgi-bin/carddisp.pl?gene=SIK2
PIGW	Phosphatidylinositol Glycan Anchor Biosynthesis Class W	Protein Coding	38	GC17P036534	4.19407	https://www.genecards.org/cgi-bin/carddisp.pl?gene=PIGW
KCNK18	Potassium Two Pore Domain Channel Subfamily K Member 18	Protein Coding	38	GC10P117197	4.19254	https://www.genecards.org/cgi-bin/carddisp.pl?gene=KCNK18

PRMT1	Protein Arginine Methyltransferase 1	Protein Coding	48	GC19P049675	4.192341	https://www.genecards.org/cgi-bin/carddisp.pl?gene=PRMT1
VNN1	Vanin 1	Protein Coding	44	GC06M132680	4.187796	https://www.genecards.org/cgi-bin/carddisp.pl?gene=VNN1
CRYGS	Crystallin Gamma S	Protein Coding	41	GC03M186538	4.184273	https://www.genecards.org/cgi-bin/carddisp.pl?gene=CRYGS
NCOA6	Nuclear Receptor Coactivator 6	Protein Coding	39	GC20M034700	4.180696	https://www.genecards.org/cgi-bin/carddisp.pl?gene=NCOA6
ANKRD2	Ankyrin Repeat Domain 2	Protein Coding	37	GC10P097572	4.176078	https://www.genecards.org/cgi-bin/carddisp.pl?gene=ANKRD2
KNL1	Kinetochore Scaffold 1	Protein Coding	34	GC15P040595	4.168321	https://www.genecards.org/cgi-bin/carddisp.pl?gene=KNL1
STARD3	StAR Related Lipid Transfer Domain Containing 3	Protein Coding	40	GC17P039637	4.16773	https://www.genecards.org/cgi-bin/carddisp.pl?gene=STARD3
SNX1	Sorting Nexin 1	Protein Coding	42	GC15P064094	4.166001	https://www.genecards.org/cgi-bin/carddisp.pl?gene=SNX1
FER	FER Tyrosine Kinase	Protein Coding	47	GC05P108747	4.163055	https://www.genecards.org/cgi-bin/carddisp.pl?gene=FER
PMAIP1	Phorbol-12-Myristate-13-Acetate-Induced Protein 1	Protein Coding	40	GC18P059899	4.163055	https://www.genecards.org/cgi-bin/carddisp.pl?gene=PMAIP1
SLC1A5	Solute Carrier Family 1 Member 5	Protein Coding	44	GC19M047159	4.158493	https://www.genecards.org/cgi-bin/carddisp.pl?gene=SLC1A5
FKBP4	FKBP Prolyl Isomerase 4	Protein Coding	45	GC12P002795	4.157864	https://www.genecards.org/cgi-bin/carddisp.pl?gene=FKBP4
PDLIM5	PDZ And LIM Domain 5	Protein Coding	40	GC04P094451	4.154386	https://www.genecards.org/cgi-bin/carddisp.pl?gene=PDLIM5
GIMAP5	GTPase, IMAP Family Member 5	Protein Coding	37	GC07P150722	4.152097	https://www.genecards.org/cgi-bin/carddisp.pl?gene=GIMAP5
NDUFV3	NADH:Ubiquinone Oxidoreductase Subunit V3	Protein Coding	41	GC21P042879	4.151853	https://www.genecards.org/cgi-bin/carddisp.pl?gene=NDUFV3
CCT8	Chaperonin Containing TCP1 Subunit 8	Protein Coding	40	GC21M029055	4.139132	https://www.genecards.org/cgi-bin/carddisp.pl?gene=CCT8
MOB3B	MOB Kinase Activator 3B	Protein Coding	36	GC09M027582	4.137991	https://www.genecards.org/cgi-bin/carddisp.pl?gene=MOB3B
PTGIR	Prostaglandin I2 Receptor	Protein Coding	48	GC19M047072	4.137783	https://www.genecards.org/cgi-bin/carddisp.pl?gene=PTGIR
B3GALT4	Beta-1,3-Galactosyltransferase 4	Protein Coding	41	GC06P033277	4.134985	https://www.genecards.org/cgi-bin/carddisp.pl?gene=B3GALT4
COX7C	Cytochrome C Oxidase Subunit 7C	Protein Coding	38	GC05P086617	4.133422	https://www.genecards.org/cgi-bin/carddisp.pl?gene=COX7C
SIRT7	Sirtuin 7	Protein Coding	42	GC17M081911	4.130557	https://www.genecards.org/cgi-bin/carddisp.pl?gene=SIRT7

TMC4	Transmembrane Channel Like 4	Protein Coding	35	GC19M054160	4.127429	https://www.genecards.org/cgi-bin/carddisp.pl?gene=TMC4
ALDH8A1	Aldehyde Dehydrogenase 8 Family Member A1	Protein Coding	38	GC06M134917	4.126362	https://www.genecards.org/cgi-bin/carddisp.pl?gene=ALDH8A1
PTGES2	Prostaglandin E Synthase 2	Protein Coding	44	GC09M128120	4.12583	https://www.genecards.org/cgi-bin/carddisp.pl?gene=PTGES2
CRACR2A	Calcium Release Activated Channel Regulator 2A	Protein Coding	31	GC12M004349	4.123631	https://www.genecards.org/cgi-bin/carddisp.pl?gene=CRACR2A
SART1	Spliceosome Associated Factor 1, Recruiter Of U4/U6.U5 Tri-SnRNP	Protein Coding	41	GC11P066273	4.122902	https://www.genecards.org/cgi-bin/carddisp.pl?gene=SART1
FGR	FGR Proto-Oncogene, Src Family Tyrosine Kinase	Protein Coding	48	GC01M027622	4.119474	https://www.genecards.org/cgi-bin/carddisp.pl?gene=FGR
C2orf16	Chromosome 2 Open Reading Frame 16	Protein Coding	27	GC02P027611	4.117465	https://www.genecards.org/cgi-bin/carddisp.pl?gene=C2orf16
GYPE	Glycophorin E (MNS Blood Group)	Protein Coding	31	GC04M143870	4.11249	https://www.genecards.org/cgi-bin/carddisp.pl?gene=GYPE
ACP6	Acid Phosphatase 6, Lysophosphatidic	Protein Coding	40	GC01M147630	4.110131	https://www.genecards.org/cgi-bin/carddisp.pl?gene=ACP6
NDUFC1	NADH:Ubiquinone Oxidoreductase Subunit C1	Protein Coding	36	GC04M139267	4.109852	https://www.genecards.org/cgi-bin/carddisp.pl?gene=NDUFC1
PSME2	Proteasome Activator Subunit 2	Protein Coding	43	GC14M024143	4.100591	https://www.genecards.org/cgi-bin/carddisp.pl?gene=PSME2
PDXDC1	Pyridoxal Dependent Decarboxylase Domain Containing 1	Protein Coding	38	GC16P014974	4.095699	https://www.genecards.org/cgi-bin/carddisp.pl?gene=PDXDC1
MIR29B2	MicroRNA 29b-2	RNA Gene	18	GC01M207806	4.092883	https://www.genecards.org/cgi-bin/carddisp.pl?gene=MIR29B2
MEF2D	Myocyte Enhancer Factor 2D	Protein Coding	45	GC01M156463	4.09202	https://www.genecards.org/cgi-bin/carddisp.pl?gene=MEF2D
SEC16B	SEC16 Homolog B, Endoplasmic Reticulum Export Factor	Protein Coding	38	GC01M177923	4.091877	https://www.genecards.org/cgi-bin/carddisp.pl?gene=SEC16B

PTGES3	Prostaglandin E Synthase 3	Protein Coding	44	GC12M056667	4.089228	https://www.genecards.org/cgi-bin/carddisp.pl?gene=PTGES3
ZNF202	Zinc Finger Protein 202	Protein Coding	40	GC11M123724	4.088984	https://www.genecards.org/cgi-bin/carddisp.pl?gene=ZNF202
TMBIM6	Transmembrane BAX Inhibitor Motif Containing 6	Protein Coding	37	GC12P049707	4.088861	https://www.genecards.org/cgi-bin/carddisp.pl?gene=TMBIM6
ATF1	Activating Transcription Factor 1	Protein Coding	47	GC12P050763	4.086227	https://www.genecards.org/cgi-bin/carddisp.pl?gene=ATF1
ANO2	Anoctamin 2	Protein Coding	37	GC12M005532	4.08603	https://www.genecards.org/cgi-bin/carddisp.pl?gene=ANO2
KPNA4	Karyopherin Subunit Alpha 4	Protein Coding	43	GC03M160494	4.085062	https://www.genecards.org/cgi-bin/carddisp.pl?gene=KPNA4
ADCY8	Adenylate Cyclase 8	Protein Coding	45	GC08M130780	4.084551	https://www.genecards.org/cgi-bin/carddisp.pl?gene=ADCY8
GRB7	Growth Factor Receptor Bound Protein 7	Protein Coding	44	GC17P039744	4.083256	https://www.genecards.org/cgi-bin/carddisp.pl?gene=GRB7
KCNK10	Potassium Two Pore Domain Channel Subfamily K Member 10	Protein Coding	40	GC14M088180	4.082622	https://www.genecards.org/cgi-bin/carddisp.pl?gene=KCNK10
IPPK	Inositol-Pentakisphosphate 2-Kinase	Protein Coding	38	GC09M092613	4.082585	https://www.genecards.org/cgi-bin/carddisp.pl?gene=IPPK
ISYNA1	Inositol-3-Phosphate Synthase 1	Protein Coding	41	GC19M018406	4.079656	https://www.genecards.org/cgi-bin/carddisp.pl?gene=ISYNA1
NUDT21	Nudix Hydrolase 21	Protein Coding	38	GC16M056429	4.0745	https://www.genecards.org/cgi-bin/carddisp.pl?gene=NUDT21
COX7A1	Cytochrome C Oxidase Subunit 7A1	Protein Coding	37	GC19M047084	4.07343	https://www.genecards.org/cgi-bin/carddisp.pl?gene=COX7A1
PSME3	Proteasome Activator Subunit 3	Protein Coding	43	GC17P042824	4.071575	https://www.genecards.org/cgi-bin/carddisp.pl?gene=PSME3
MACROH2A2	MacroH2A.2 Histone	Protein Coding	30	GC10P070053	4.069562	https://www.genecards.org/cgi-bin/carddisp.pl?gene=MACROH2A2
PLXND1	Plexin D1	Protein Coding	41	GC03M129555	4.069346	https://www.genecards.org/cgi-bin/carddisp.pl?gene=PLXND1
RIPK4	Receptor Interacting Serine/Threonine Kinase 4	Protein Coding	44	GC21M041739	4.068773	https://www.genecards.org/cgi-bin/carddisp.pl?gene=RIPK4
STARD10	StAR Related Lipid Transfer Domain Containing 10	Protein Coding	37	GC11M072774	4.068071	https://www.genecards.org/cgi-bin/carddisp.pl?gene=STARD10
RPA2	Replication Protein A2	Protein Coding	45	GC01M027902	4.065948	https://www.genecards.org/cgi-bin/carddisp.pl?gene=RPA2

DLEU1	Deleted In Lymphocytic Leukemia 1	RNA Gene	28	GC13P050098	4.058257	https://www.genecards.org/cgi-bin/carddisp.pl?gene=DLEU1
CBLL1	Cbl Proto-Oncogene Like 1	Protein Coding	37	GC07P107743	4.05614	https://www.genecards.org/cgi-bin/carddisp.pl?gene=CBLL1
EPS8L3	EPS8 Like 3	Protein Coding	38	GC01M109751	4.055779	https://www.genecards.org/cgi-bin/carddisp.pl?gene=EPS8L3
PTCD1	Pentatricopeptide Repeat Domain 1	Protein Coding	34	GC07M099429	4.051902	https://www.genecards.org/cgi-bin/carddisp.pl?gene=PTCD1
C1QTNF9	C1q And TNF Related 9	Protein Coding	34	GC13P024307	4.048708	https://www.genecards.org/cgi-bin/carddisp.pl?gene=C1QTNF9
TLCD4-RWDD3	TLCD4-RWDD3 Readthrough	Protein Coding	16	GC01P095119	4.047915	https://www.genecards.org/cgi-bin/carddisp.pl?gene=TLCD4-RWDD3
RAP1B	RAP1B, Member Of RAS Oncogene Family	Protein Coding	45	GC12P068610	4.047623	https://www.genecards.org/cgi-bin/carddisp.pl?gene=RAP1B
UCN2	Urocortin 2	Protein Coding	34	GC03M048561	4.046426	https://www.genecards.org/cgi-bin/carddisp.pl?gene=UCN2
HNRNPR	Heterogeneous Nuclear Ribonucleoprotein R	Protein Coding	40	GC01M023303	4.04319	https://www.genecards.org/cgi-bin/carddisp.pl?gene=HNRNPR
GSTK1	Glutathione S-Transferase Kappa 1	Protein Coding	42	GC07P145809	4.041059	https://www.genecards.org/cgi-bin/carddisp.pl?gene=GSTK1
ATAD2	ATPase Family AAA Domain Containing 2	Protein Coding	40	GC08M123319	4.03664	https://www.genecards.org/cgi-bin/carddisp.pl?gene=ATAD2
SMARCC1	SWI/SNF Related, Matrix Associated, Actin Dependent Regulator Of Chromatin Subfamily C Member 1	Protein Coding	44	GC03M047585	4.035441	https://www.genecards.org/cgi-bin/carddisp.pl?gene=SMARCC1
GDE1	Glycerophosphodiester Phosphodiesterase 1	Protein Coding	37	GC16M019513	4.034425	https://www.genecards.org/cgi-bin/carddisp.pl?gene=GDE1
SIM1	SIM BHLH Transcription Factor 1	Protein Coding	41	GC06M100386	4.033214	https://www.genecards.org/cgi-bin/carddisp.pl?gene=SIM1
HOXC13	Homeobox C13	Protein Coding	41	GC12P053938	4.031942	https://www.genecards.org/cgi-bin/carddisp.pl?gene=HOXC13
SLC7A11	Solute Carrier Family 7 Member 11	Protein Coding	45	GC04M138164	4.026815	https://www.genecards.org/cgi-bin/carddisp.pl?gene=SLC7A11
EIF3B	Eukaryotic Translation Initiation Factor 3 Subunit B	Protein Coding	40	GC07P002354	4.025175	https://www.genecards.org/cgi-bin/carddisp.pl?gene=EIF3B

INTS5	Integrator Complex Subunit 5	Protein Coding	35	GC11M069255	4.022593	https://www.genecards.org/cgi-bin/carddisp.pl?gene=INTS5
PEA15	Proliferation And Apoptosis Adaptor Protein 15	Protein Coding	44	GC01P160205	4.0219	https://www.genecards.org/cgi-bin/carddisp.pl?gene=PEA15
AFAP1	Actin Filament Associated Protein 1	Protein Coding	39	GC04M007758	4.019042	https://www.genecards.org/cgi-bin/carddisp.pl?gene=AFAP1
ILVBL	IlvB Acetolactate Synthase Like	Protein Coding	38	GC19M015116	4.017715	https://www.genecards.org/cgi-bin/carddisp.pl?gene=ILVBL
TCP1	T-Complex 1	Protein Coding	41	GC06M159778	4.017245	https://www.genecards.org/cgi-bin/carddisp.pl?gene=TCP1
MPZL1	Myelin Protein Zero Like 1	Protein Coding	41	GC01P167721	4.017209	https://www.genecards.org/cgi-bin/carddisp.pl?gene=MPZL1
CAMKMT	Calmodulin-Lysine N-Methyltransferase	Protein Coding	36	GC02P044361	4.016611	https://www.genecards.org/cgi-bin/carddisp.pl?gene=CAMKMT
RIOK2	RIO Kinase 2	Protein Coding	41	GC05M097160	4.015095	https://www.genecards.org/cgi-bin/carddisp.pl?gene=RIOK2
FLRT1	Fibronectin Leucine Rich Transmembrane Protein 1	Protein Coding	40	GC11P064036	4.012539	https://www.genecards.org/cgi-bin/carddisp.pl?gene=FLRT1
RNF7	Ring Finger Protein 7	Protein Coding	43	GC03P141738	4.011736	https://www.genecards.org/cgi-bin/carddisp.pl?gene=RNF7
RRAGC	Ras Related GTP Binding C	Protein Coding	43	GC01M038847	4.002389	https://www.genecards.org/cgi-bin/carddisp.pl?gene=RRAGC
EBF1	EBF Transcription Factor 1	Protein Coding	41	GC05M158695	4.000239	https://www.genecards.org/cgi-bin/carddisp.pl?gene=EBF1
RBM38	RNA Binding Motif Protein 38	Protein Coding	37	GC20P057391	3.999289	https://www.genecards.org/cgi-bin/carddisp.pl?gene=RBM38
TUNAR	TCL1 Upstream Neural Differentiation-Associated RNA	RNA Gene	16	GC14P095876	3.998121	https://www.genecards.org/cgi-bin/carddisp.pl?gene=TUNAR
GRK6	G Protein-Coupled Receptor Kinase 6	Protein Coding	45	GC05P177403	3.997712	https://www.genecards.org/cgi-bin/carddisp.pl?gene=GRK6
SPRR1B	Small Proline Rich Protein 1B	Protein Coding	37	GC01P153031	3.993895	https://www.genecards.org/cgi-bin/carddisp.pl?gene=SPRR1B
CCNT2	Cyclin T2	Protein Coding	39	GC02P134918	3.991917	https://www.genecards.org/cgi-bin/carddisp.pl?gene=CCNT2
NLRP6	NLR Family Pyrin Domain Containing 6	Protein Coding	38	GC11P000269	3.990225	https://www.genecards.org/cgi-bin/carddisp.pl?gene=NLRP6
MYO1C	Myosin IC	Protein Coding	44	GC17M001464	3.990014	https://www.genecards.org/cgi-bin/carddisp.pl?gene=MYO1C

FBXO5	F-Box Protein 5	Protein Coding	38	GC06M152970	3.988118	https://www.genecards.org/cgi-bin/carddisp.pl?gene=FBXO5
SFRP5	Secreted Frizzled Related Protein 5	Protein Coding	40	GC10M097766	3.984463	https://www.genecards.org/cgi-bin/carddisp.pl?gene=SFRP5
SEC16A	SEC16 Homolog A, Endoplasmic Reticulum Export Factor	Protein Coding	37	GC09M136440	3.98049	https://www.genecards.org/cgi-bin/carddisp.pl?gene=SEC16A
CLDN5	Claudin 5	Protein Coding	43	GC22M019523	3.97901	https://www.genecards.org/cgi-bin/carddisp.pl?gene=CLDN5
PHF2	PHD Finger Protein 2	Protein Coding	39	GC09P093576	3.975384	https://www.genecards.org/cgi-bin/carddisp.pl?gene=PHF2
TOM1	Target Of Myb1 Membrane Trafficking Protein	Protein Coding	43	GC22P035299	3.970874	https://www.genecards.org/cgi-bin/carddisp.pl?gene=TOM1
TOX3	TOX High Mobility Group Box Family Member 3	Protein Coding	38	GC16M052440	3.96215	https://www.genecards.org/cgi-bin/carddisp.pl?gene=TOX3
ATP5MJ	ATP Synthase Membrane Subunit J	Protein Coding	27	GC14M103917	3.96122	https://www.genecards.org/cgi-bin/carddisp.pl?gene=ATP5MJ
SCP2D1	SCP2 Sterol Binding Domain Containing 1	Protein Coding	29	GC20P018813	3.960956	https://www.genecards.org/cgi-bin/carddisp.pl?gene=SCP2D1
RAD52	RAD52 Homolog, DNA Repair Protein	Protein Coding	42	GC12M000912	3.958795	https://www.genecards.org/cgi-bin/carddisp.pl?gene=RAD52
SP4	Sp4 Transcription Factor	Protein Coding	42	GC07P021434	3.957705	https://www.genecards.org/cgi-bin/carddisp.pl?gene=SP4
TRADD	TNFRSF1A Associated Via Death Domain	Protein Coding	44	GC16M067154	3.957182	https://www.genecards.org/cgi-bin/carddisp.pl?gene=TRADD
RPS8	Ribosomal Protein S8	Protein Coding	40	GC01P044775	3.949356	https://www.genecards.org/cgi-bin/carddisp.pl?gene=RPS8
KIR2DS1	Killer Cell Immunoglobulin Like Receptor, Two Ig Domains And Short Cytoplasmic Tail 1	Protein Coding	23	GC19Mr00063	3.948604	https://www.genecards.org/cgi-bin/carddisp.pl?gene=KIR2DS1
RPL23A	Ribosomal Protein L23a	Protein Coding	42	GC17P028719	3.946427	https://www.genecards.org/cgi-bin/carddisp.pl?gene=RPL23A
RGS2	Regulator Of G Protein Signaling 2	Protein Coding	43	GC01P192809	3.94433	https://www.genecards.org/cgi-bin/carddisp.pl?gene=RGS2

NDUFA7	NADH:Ubiquinone Oxidoreductase Subunit A7	Protein Coding	38	GC19M008308	3.93049	https://www.genecards.org/cgi-bin/carddisp.pl?gene=NDUFA7
PDF	Peptide Deformylase, Mitochondrial	Protein Coding	35	GC16M069656	3.927101	https://www.genecards.org/cgi-bin/carddisp.pl?gene=PDF
RUVBL2	RuvB Like AAA ATPase 2	Protein Coding	45	GC19P048993	3.926382	https://www.genecards.org/cgi-bin/carddisp.pl?gene=RUVBL2
MTMR9	Myotubularin Related Protein 9	Protein Coding	38	GC08P011284	3.91943	https://www.genecards.org/cgi-bin/carddisp.pl?gene=MTMR9
OLA1	Obg Like ATPase 1	Protein Coding	43	GC02M174072	3.915539	https://www.genecards.org/cgi-bin/carddisp.pl?gene=OLA1
PLPPR4	Phospholipid Phosphatase Related 4	Protein Coding	31	GC01P099262	3.913586	https://www.genecards.org/cgi-bin/carddisp.pl?gene=PLPPR4
NCS1	Neuronal Calcium Sensor 1	Protein Coding	43	GC09P130172	3.909512	https://www.genecards.org/cgi-bin/carddisp.pl?gene=NCS1
PPP1R12A	Protein Phosphatase 1 Regulatory Subunit 12A	Protein Coding	44	GC12M079773	3.907771	https://www.genecards.org/cgi-bin/carddisp.pl?gene=PPP1R12A
CHML	CHM Like Rab Escort Protein	Protein Coding	38	GC01M241628	3.905396	https://www.genecards.org/cgi-bin/carddisp.pl?gene=CHML
CCT2	Chaperonin Containing TCP1 Subunit 2	Protein Coding	41	GC12P069585	3.9032	https://www.genecards.org/cgi-bin/carddisp.pl?gene=CCT2
ACTL6B	Actin Like 6B	Protein Coding	43	GC07M100643	3.902606	https://www.genecards.org/cgi-bin/carddisp.pl?gene=ACTL6B
MIRLET7F1	MicroRNA Let-7f-1	RNA Gene	19	GC09P094193	3.900208	https://www.genecards.org/cgi-bin/carddisp.pl?gene=MIRLET7F1
PLEKHA1	Pleckstrin Homology Domain Containing A1	Protein Coding	42	GC10P122374	3.896281	https://www.genecards.org/cgi-bin/carddisp.pl?gene=PLEKHA1
SPATA13	Spermatogenesis Associated 13	Protein Coding	38	GC13P024016	3.895677	https://www.genecards.org/cgi-bin/carddisp.pl?gene=SPATA13
USP15	Ubiquitin Specific Peptidase 15	Protein Coding	48	GC12P062260	3.894948	https://www.genecards.org/cgi-bin/carddisp.pl?gene=USP15
PCSK7	Proprotein Convertase Subtilisin/Kexin Type 7	Protein Coding	44	GC11M117199	3.892378	https://www.genecards.org/cgi-bin/carddisp.pl?gene=PCSK7
ATHS	Atherosclerosis Susceptibility (Lipoprotein Associated)	Genetic Locus	4	GC19U990005	3.891316	https://www.genecards.org/cgi-bin/carddisp.pl?gene=ATHS
ZNF512	Zinc Finger Protein 512	Protein Coding	32	GC02P027582	3.890964	https://www.genecards.org/cgi-bin/carddisp.pl?gene=ZNF512

PABPC4	Poly(A) Binding Protein Cytoplasmic 4	Protein Coding	42	GC01M039560	3.883965	https://www.genecards.org/cgi-bin/carddisp.pl?gene=PABPC4
SEC22B	SEC22 Homolog B, Vesicle Trafficking Protein	Protein Coding	36	GC01M120150	3.879227	https://www.genecards.org/cgi-bin/carddisp.pl?gene=SEC22B
PABIR1	PP2A Aalpha (PPP2R1A) And B55A (PPP2R2A) Interacting Phosphatase Regulator 1	Protein Coding	25	GC09P068781	3.878857	https://www.genecards.org/cgi-bin/carddisp.pl?gene=PABIR1
ABI3	ABI Family Member 3	Protein Coding	38	GC17P049210	3.876922	https://www.genecards.org/cgi-bin/carddisp.pl?gene=ABI3
RTRAF	RNA Transcription, Translation And Transport Factor	Protein Coding	29	GC14P051992	3.875666	https://www.genecards.org/cgi-bin/carddisp.pl?gene=RTRAF
KCNA4	Potassium Voltage-Gated Channel Subfamily A Member 4	Protein Coding	44	GC11M030009	3.871591	https://www.genecards.org/cgi-bin/carddisp.pl?gene=KCNA4
STAMBP	STAM Binding Protein	Protein Coding	47	GC02P073828	3.871397	https://www.genecards.org/cgi-bin/carddisp.pl?gene=STAMBP
RWDD3	RWD Domain Containing 3	Protein Coding	36	GC01P095171	3.868048	https://www.genecards.org/cgi-bin/carddisp.pl?gene=RWDD3
MAPK13	Mitogen-Activated Protein Kinase 13	Protein Coding	48	GC06P055340	3.867134	https://www.genecards.org/cgi-bin/carddisp.pl?gene=MAPK13
CUL5	Cullin 5	Protein Coding	45	GC11P108008	3.865067	https://www.genecards.org/cgi-bin/carddisp.pl?gene=CUL5
SDK1	Sidekick Cell Adhesion Molecule 1	Protein Coding	36	GC07P003307	3.860195	https://www.genecards.org/cgi-bin/carddisp.pl?gene=SDK1
TUT1	Terminal Uridylyl Transferase 1, U6 SnRNA-Specific	Protein Coding	36	GC11M069245	3.857761	https://www.genecards.org/cgi-bin/carddisp.pl?gene=TUT1
SLC30A2	Solute Carrier Family 30 Member 2	Protein Coding	44	GC01M026048	3.856939	https://www.genecards.org/cgi-bin/carddisp.pl?gene=SLC30A2
RRBP1	Ribosome Binding Protein 1	Protein Coding	40	GC20M017613	3.853372	https://www.genecards.org/cgi-bin/carddisp.pl?gene=RRBP1
ADGRL2	Adhesion G Protein-Coupled Receptor L2	Protein Coding	38	GC01P081306	3.850107	https://www.genecards.org/cgi-bin/carddisp.pl?gene=ADGRL2
SELENOO	Selenoprotein O	Protein Coding	24	GC22P050201	3.84838	https://www.genecards.org/cgi-bin/carddisp.pl?gene=SELENOO

MIR1296	MicroRNA 1296	RNA Gene	16	GC10M063372	3.842494	https://www.genecards.org/cgi-bin/carddisp.pl?gene=MIR1296
SYT14	Synaptotagmin 14	Protein Coding	38	GC01P209900	3.841401	https://www.genecards.org/cgi-bin/carddisp.pl?gene=SYT14
SRRM1	Serine And Arginine Repetitive Matrix 1	Protein Coding	38	GC01P024631	3.840693	https://www.genecards.org/cgi-bin/carddisp.pl?gene=SRRM1
CHCHD3	Coiled-Coil-Helix-Coiled-Coil-Helix Domain Containing 3	Protein Coding	38	GC07M132784	3.840507	https://www.genecards.org/cgi-bin/carddisp.pl?gene=CHCHD3
SLC22A8	Solute Carrier Family 22 Member 8	Protein Coding	43	GC11M069279	3.837129	https://www.genecards.org/cgi-bin/carddisp.pl?gene=SLC22A8
RGS19	Regulator Of G Protein Signaling 19	Protein Coding	41	GC20M064073	3.836257	https://www.genecards.org/cgi-bin/carddisp.pl?gene=RGS19
AATF	Apoptosis Antagonizing Transcription Factor	Protein Coding	40	GC17P036948	3.835102	https://www.genecards.org/cgi-bin/carddisp.pl?gene=AATF
FGFRL1	Fibroblast Growth Factor Receptor Like 1	Protein Coding	43	GC04P001058	3.832848	https://www.genecards.org/cgi-bin/carddisp.pl?gene=FGFRL1
SSR3	Signal Sequence Receptor Subunit 3	Protein Coding	38	GC03M156540	3.832595	https://www.genecards.org/cgi-bin/carddisp.pl?gene=SSR3
CCNA1	Cyclin A1	Protein Coding	43	GC13P036431	3.832235	https://www.genecards.org/cgi-bin/carddisp.pl?gene=CCNA1
SENP1	SUMO Specific Peptidase 1	Protein Coding	43	GC12M048042	3.83194	https://www.genecards.org/cgi-bin/carddisp.pl?gene=SENP1
CCT3	Chaperonin Containing TCP1 Subunit 3	Protein Coding	42	GC01M156308	3.822279	https://www.genecards.org/cgi-bin/carddisp.pl?gene=CCT3
ARL15	ADP Ribosylation Factor Like GTPase 15	Protein Coding	36	GC05M053883	3.821368	https://www.genecards.org/cgi-bin/carddisp.pl?gene=ARL15
TTK	TTK Protein Kinase	Protein Coding	48	GC06P080003	3.820232	https://www.genecards.org/cgi-bin/carddisp.pl?gene=TTK
WDR7	WD Repeat Domain 7	Protein Coding	34	GC18P056651	3.814718	https://www.genecards.org/cgi-bin/carddisp.pl?gene=WDR7
TIE1	Tyrosine Kinase With Immunoglobulin Like And EGF Like Domains 1	Protein Coding	43	GC01P043300	3.808009	https://www.genecards.org/cgi-bin/carddisp.pl?gene=TIE1
ATP10D	ATPase Phospholipid Transporting 10D (Putative)	Protein Coding	39	GC04P047490	3.807865	https://www.genecards.org/cgi-bin/carddisp.pl?gene=ATP10D

H3C3	H3 Clustered Histone 3	Protein Coding	31	GC06P054978	3.804129	https://www.genecards.org/cgi-bin/carddisp.pl?gene=H3C3
IFIT3	Interferon Induced Protein With Tetratricopeptide Repeats 3	Protein Coding	39	GC10P089327	3.799982	https://www.genecards.org/cgi-bin/carddisp.pl?gene=IFIT3
COX8C	Cytochrome C Oxidase Subunit 8C	Protein Coding	32	GC14P093348	3.797909	https://www.genecards.org/cgi-bin/carddisp.pl?gene=COX8C
CUTA	CutA Divalent Cation Tolerance Homolog	Protein Coding	36	GC06M046993	3.797663	https://www.genecards.org/cgi-bin/carddisp.pl?gene=CUTA
LOC101928118	Uncharacterized LOC101928118	RNA Gene	11	GC01M095163	3.790787	https://www.genecards.org/cgi-bin/carddisp.pl?gene=LOC101928118
RNGTT	RNA Guanylyltransferase And 5'-Phosphatase	Protein Coding	44	GC06M088609	3.789963	https://www.genecards.org/cgi-bin/carddisp.pl?gene=RNGTT
SYT7	Synaptotagmin 7	Protein Coding	40	GC11M061513	3.787489	https://www.genecards.org/cgi-bin/carddisp.pl?gene=SYT7
ITFG1	Integrin Alpha FG-GAP Repeat Containing 1	Protein Coding	37	GC16M047156	3.785235	https://www.genecards.org/cgi-bin/carddisp.pl?gene=ITFG1
METTL14	Methyltransferase Like 14	Protein Coding	38	GC04P118685	3.782868	https://www.genecards.org/cgi-bin/carddisp.pl?gene=METTL14
MAP4K4	Mitogen-Activated Protein Kinase Kinase Kinase Kinase 4	Protein Coding	48	GC02P101773	3.778296	https://www.genecards.org/cgi-bin/carddisp.pl?gene=MAP4K4
GPAA1	Glycosylphosphatidylinositol Anchor Attachment 1	Protein Coding	43	GC08P144082	3.777599	https://www.genecards.org/cgi-bin/carddisp.pl?gene=GPAA1
ID4	Inhibitor Of DNA Binding 4, HLH Protein	Protein Coding	40	GC06P019837	3.774964	https://www.genecards.org/cgi-bin/carddisp.pl?gene=ID4
NDUFB1	NADH:Ubiquinone Oxidoreductase Subunit B1	Protein Coding	37	GC14M092116	3.77119	https://www.genecards.org/cgi-bin/carddisp.pl?gene=NDUFB1
BRMS1	BRMS1 Transcriptional Repressor And Anoikis Regulator	Protein Coding	38	GC11M069459	3.768492	https://www.genecards.org/cgi-bin/carddisp.pl?gene=BRMS1
RBM5	RNA Binding Motif Protein 5	Protein Coding	40	GC03P050139	3.759135	https://www.genecards.org/cgi-bin/carddisp.pl?gene=RBM5
BDH2	3-Hydroxybutyrate Dehydrogenase 2	Protein Coding	39	GC04M103077	3.753363	https://www.genecards.org/cgi-bin/carddisp.pl?gene=BDH2

SF3B3	Splicing Factor 3b Subunit 3	Protein Coding	40	GC16P070523	3.752798	https://www.genecards.org/cgi-bin/carddisp.pl?gene=SF3B3
NUCKS1	Nuclear Casein Kinase And Cyclin Dependent Kinase Substrate 1	Protein Coding	36	GC01M205712	3.750539	https://www.genecards.org/cgi-bin/carddisp.pl?gene=NUCKS1
TACC1	Transforming Acidic Coiled-Coil Containing Protein 1	Protein Coding	41	GC08P038728	3.749468	https://www.genecards.org/cgi-bin/carddisp.pl?gene=TACC1
SRSF4	Serine And Arginine Rich Splicing Factor 4	Protein Coding	39	GC01M029147	3.747962	https://www.genecards.org/cgi-bin/carddisp.pl?gene=SRSF4
FEZF1	FEZ Family Zinc Finger 1	Protein Coding	40	GC07M122301	3.735251	https://www.genecards.org/cgi-bin/carddisp.pl?gene=FEZF1
RIC8A	RIC8 Guanine Nucleotide Exchange Factor A	Protein Coding	36	GC11P000207	3.734514	https://www.genecards.org/cgi-bin/carddisp.pl?gene=RIC8A
SPATA5	Spermatogenesis Associated 5	Protein Coding	41	GC04P122923	3.72644	https://www.genecards.org/cgi-bin/carddisp.pl?gene=SPATA5
BCAR3	BCAR3 Adaptor Protein, NSP Family Member	Protein Coding	40	GC01M093561	3.726127	https://www.genecards.org/cgi-bin/carddisp.pl?gene=BCAR3
FAM3C	FAM3 Metabolism Regulating Signaling Molecule C	Protein Coding	40	GC07M121349	3.722619	https://www.genecards.org/cgi-bin/carddisp.pl?gene=FAM3C
AGR3	Anterior Gradient 3, Protein Disulphide Isomerase Family Member	Protein Coding	38	GC07M016854	3.721906	https://www.genecards.org/cgi-bin/carddisp.pl?gene=AGR3
PIAS4	Protein Inhibitor Of Activated STAT 4	Protein Coding	43	GC19P004007	3.720944	https://www.genecards.org/cgi-bin/carddisp.pl?gene=PIAS4
ZFP36L1	ZFP36 Ring Finger Protein Like 1	Protein Coding	44	GC14M068787	3.716752	https://www.genecards.org/cgi-bin/carddisp.pl?gene=ZFP36L1
KIF16B	Kinesin Family Member 16B	Protein Coding	38	GC20M016272	3.716726	https://www.genecards.org/cgi-bin/carddisp.pl?gene=KIF16B
ANXA3	Annexin A3	Protein Coding	43	GC04P078551	3.714436	https://www.genecards.org/cgi-bin/carddisp.pl?gene=ANXA3
ZFP36L2	ZFP36 Ring Finger Protein Like 2	Protein Coding	38	GC02M043184	3.714127	https://www.genecards.org/cgi-bin/carddisp.pl?gene=ZFP36L2

GABPA	GA Binding Protein Transcription Factor Subunit Alpha	Protein Coding	41	GC21P025734	3.711926	https://www.genecards.org/cgi-bin/carddisp.pl?gene=GABPA
PTGFR	Prostaglandin F Receptor	Protein Coding	45	GC01P078303	3.709012	https://www.genecards.org/cgi-bin/carddisp.pl?gene=PTGFR
TNFAIP6	TNF Alpha Induced Protein 6	Protein Coding	41	GC02P151357	3.707534	https://www.genecards.org/cgi-bin/carddisp.pl?gene=TNFAIP6
KCNK2	Potassium Two Pore Domain Channel Subfamily K Member 2	Protein Coding	40	GC01P215005	3.706043	https://www.genecards.org/cgi-bin/carddisp.pl?gene=KCNK2
IER3	Immediate Early Response 3	Protein Coding	38	GC06M030743	3.703523	https://www.genecards.org/cgi-bin/carddisp.pl?gene=IER3
RABL3	RAB, Member Of RAS Oncogene Family Like 3	Protein Coding	34	GC03M120686	3.700064	https://www.genecards.org/cgi-bin/carddisp.pl?gene=RABL3
RND3	Rho Family GTPase 3	Protein Coding	43	GC02M150468	3.699605	https://www.genecards.org/cgi-bin/carddisp.pl?gene=RND3
ANAPC5	Anaphase Promoting Complex Subunit 5	Protein Coding	38	GC12M121308	3.695754	https://www.genecards.org/cgi-bin/carddisp.pl?gene=ANAPC5
ADH5P4	ADH5 Pseudogene 4	Pseudogene	8	GC06M065835	3.69552	https://www.genecards.org/cgi-bin/carddisp.pl?gene=ADH5P4
NRBP1	Nuclear Receptor Binding Protein 1	Protein Coding	40	GC02P027427	3.686369	https://www.genecards.org/cgi-bin/carddisp.pl?gene=NRBP1
BMP8B	Bone Morphogenetic Protein 8b	Protein Coding	39	GC01M039757	3.684701	https://www.genecards.org/cgi-bin/carddisp.pl?gene=BMP8B
DNAJC15	DnaJ Heat Shock Protein Family (Hsp40) Member C15	Protein Coding	37	GC13P043023	3.683096	https://www.genecards.org/cgi-bin/carddisp.pl?gene=DNAJC15
RBBP7	RB Binding Protein 7, Chromatin Remodeling Factor	Protein Coding	41	GC0XM016839	3.682717	https://www.genecards.org/cgi-bin/carddisp.pl?gene=RBBP7
RGPD2	RANBP2 Like And GRIP Domain Containing 2	Protein Coding	30	GC02M087755	3.680158	https://www.genecards.org/cgi-bin/carddisp.pl?gene=RGPD2
ZDHHC13	Zinc Finger DHHC-Type Palmitoyltransferase 13	Protein Coding	38	GC11P019095	3.67967	https://www.genecards.org/cgi-bin/carddisp.pl?gene=ZDHHC13
VPS52	VPS52 Subunit Of GARP Complex	Protein Coding	36	GC06M046975	3.678367	https://www.genecards.org/cgi-bin/carddisp.pl?gene=VPS52

GGT5	Gamma-Glutamyltransferase 5	Protein Coding	40	GC22M024219	3.673661	https://www.genecards.org/cgi-bin/carddisp.pl?gene=GGT5
DDX17	DEAD-Box Helicase 17	Protein Coding	41	GC22M038483	3.672779	https://www.genecards.org/cgi-bin/carddisp.pl?gene=DDX17
DSCAML1	DS Cell Adhesion Molecule Like 1	Protein Coding	38	GC11M117427	3.670866	https://www.genecards.org/cgi-bin/carddisp.pl?gene=DSCAML1
MTF2	Metal Response Element Binding Transcription Factor 2	Protein Coding	39	GC01P093079	3.669576	https://www.genecards.org/cgi-bin/carddisp.pl?gene=MTF2
GAL3ST1	Galactose-3-O-Sulfotransferase 1	Protein Coding	37	GC22M030554	3.666687	https://www.genecards.org/cgi-bin/carddisp.pl?gene=GAL3ST1
TEX36	Testis Expressed 36	Protein Coding	29	GC10M125576	3.665281	https://www.genecards.org/cgi-bin/carddisp.pl?gene=TEX36
TFAP2C	Transcription Factor AP-2 Gamma	Protein Coding	43	GC20P056629	3.662132	https://www.genecards.org/cgi-bin/carddisp.pl?gene=TFAP2C
SNAP23	Synaptosome Associated Protein 23	Protein Coding	45	GC15P042491	3.660439	https://www.genecards.org/cgi-bin/carddisp.pl?gene=SNAP23
TMOD3	Tropomodulin 3	Protein Coding	40	GC15P051829	3.659048	https://www.genecards.org/cgi-bin/carddisp.pl?gene=TMOD3
CARD6	Caspase Recruitment Domain Family Member 6	Protein Coding	38	GC05P040841	3.656702	https://www.genecards.org/cgi-bin/carddisp.pl?gene=CARD6
DGKA	Diacylglycerol Kinase Alpha	Protein Coding	45	GC12P055927	3.654264	https://www.genecards.org/cgi-bin/carddisp.pl?gene=DGKA
DDX50	DExD-Box Helicase 50	Protein Coding	40	GC10P068901	3.652528	https://www.genecards.org/cgi-bin/carddisp.pl?gene=DDX50
RBMX	RNA Binding Motif Protein X-Linked	Protein Coding	43	GC0XM136848	3.652502	https://www.genecards.org/cgi-bin/carddisp.pl?gene=RBMX
TPD52L2	TPD52 Like 2	Protein Coding	38	GC20P063865	3.652063	https://www.genecards.org/cgi-bin/carddisp.pl?gene=TPD52L2
LIMA1	LIM Domain And Actin Binding 1	Protein Coding	40	GC12M050175	3.651401	https://www.genecards.org/cgi-bin/carddisp.pl?gene=LIMA1
MIR217	MicroRNA 217	RNA Gene	18	GC02M055982	3.64604	https://www.genecards.org/cgi-bin/carddisp.pl?gene=MIR217
PIM3	Pim-3 Proto-Oncogene, Serine/Threonine Kinase	Protein Coding	40	GC22P049960	3.643878	https://www.genecards.org/cgi-bin/carddisp.pl?gene=PIM3
IFITM2	Interferon Induced Transmembrane Protein 2	Protein Coding	37	GC11P000300	3.6438	https://www.genecards.org/cgi-bin/carddisp.pl?gene=IFITM2
TBC1D15	TBC1 Domain Family Member 15	Protein Coding	38	GC12P071839	3.642407	https://www.genecards.org/cgi-bin/carddisp.pl?gene=TBC1D15

CTCFL	CCCTC-Binding Factor Like	Protein Coding	40	GC20M057495	3.640864	https://www.genecards.org/cgi-bin/carddisp.pl?gene=CTCFL
CDC34	Cell Division Cycle 34, Ubiqiutin Conjugating Enzyme	Protein Coding	48	GC19P000532	3.640702	https://www.genecards.org/cgi-bin/carddisp.pl?gene=CDC34
CAPZB	Capping Actin Protein Of Muscle Z-Line Subunit Beta	Protein Coding	41	GC01M019339	3.640276	https://www.genecards.org/cgi-bin/carddisp.pl?gene=CAPZB
CMPK1	Cytidine/Uridine Monophosphate Kinase 1	Protein Coding	44	GC01P047333	3.640109	https://www.genecards.org/cgi-bin/carddisp.pl?gene=CMPK1
PPA1	Inorganic Pyrophosphatase 1	Protein Coding	41	GC10M070202	3.634335	https://www.genecards.org/cgi-bin/carddisp.pl?gene=PPA1
LOC111365141	NOS2 5' Regulatory Region	Biological Region	2	GC17P027800	3.630841	https://www.genecards.org/cgi-bin/carddisp.pl?gene=LOC111365141
ATP2B4	ATPase Plasma Membrane Ca2+ Transporting 4	Protein Coding	45	GC01P203626	3.630209	https://www.genecards.org/cgi-bin/carddisp.pl?gene=ATP2B4
SLC9A8	Solute Carrier Family 9 Member A8	Protein Coding	40	GC20P049813	3.630065	https://www.genecards.org/cgi-bin/carddisp.pl?gene=SLC9A8
FAM171A1	Family With Sequence Similarity 171 Member A1	Protein Coding	33	GC10M015254	3.627962	https://www.genecards.org/cgi-bin/carddisp.pl?gene=FAM171A1
SF3A1	Splicing Factor 3a Subunit 1	Protein Coding	40	GC22M030331	3.627717	https://www.genecards.org/cgi-bin/carddisp.pl?gene=SF3A1
GLIS1	GLIS Family Zinc Finger 1	Protein Coding	34	GC01M053507	3.626914	https://www.genecards.org/cgi-bin/carddisp.pl?gene=GLIS1
PLXNA1	Plexin A1	Protein Coding	44	GC03P126988	3.624239	https://www.genecards.org/cgi-bin/carddisp.pl?gene=PLXNA1
DGKB	Diacylglycerol Kinase Beta	Protein Coding	46	GC07M014145	3.616496	https://www.genecards.org/cgi-bin/carddisp.pl?gene=DGKB
VAMP7	Vesicle Associated Membrane Protein 7	Protein Coding	43	GC0XP155881	3.61413	https://www.genecards.org/cgi-bin/carddisp.pl?gene=VAMP7
PTK6	Protein Tyrosine Kinase 6	Protein Coding	48	GC20M063528	3.601345	https://www.genecards.org/cgi-bin/carddisp.pl?gene=PTK6
GSDMD	Gasdermin D	Protein Coding	40	GC08P143553	3.599214	https://www.genecards.org/cgi-bin/carddisp.pl?gene=GSDMD
IBTK	Inhibitor Of Bruton Tyrosine Kinase	Protein Coding	40	GC06M082169	3.597259	https://www.genecards.org/cgi-bin/carddisp.pl?gene=IBTK
EFNA5	Ephrin A5	Protein Coding	44	GC05M107376	3.596578	https://www.genecards.org/cgi-bin/carddisp.pl?gene=EFNA5
TMEM108	Transmembrane Protein 108	Protein Coding	36	GC03P133038	3.59309	https://www.genecards.org/cgi-bin/carddisp.pl?gene=TMEM108

EIF3L	Eukaryotic Translation Initiation Factor 3 Subunit L	Protein Coding	39	GC22P037848	3.589599	https://www.genecards.org/cgi-bin/carddisp.pl?gene=EIF3L
PRICKLE4	Prickle Planar Cell Polarity Protein 4	Protein Coding	36	GC06P041780	3.58647	https://www.genecards.org/cgi-bin/carddisp.pl?gene=PRICKLE4
AMY1A	Amylase Alpha 1A	Protein Coding	35	GC01P103651	3.584145	https://www.genecards.org/cgi-bin/carddisp.pl?gene=AMY1A
CIDEA	Cell Death Inducing DFFA Like Effector A	Protein Coding	41	GC18P012254	3.584048	https://www.genecards.org/cgi-bin/carddisp.pl?gene=CIDEA
CHST2	Carbohydrate Sulfotransferase 2	Protein Coding	38	GC03P143119	3.583963	https://www.genecards.org/cgi-bin/carddisp.pl?gene=CHST2
TKTL1	Transketolase Like 1	Protein Coding	42	GC0XP154295	3.578979	https://www.genecards.org/cgi-bin/carddisp.pl?gene=TKTL1
BIRC6	Baculoviral IAP Repeat Containing 6	Protein Coding	43	GC02P032357	3.575604	https://www.genecards.org/cgi-bin/carddisp.pl?gene=BIRC6
UBE2J1	Ubiquitin Conjugating Enzyme E2 J1	Protein Coding	41	GC06M089326	3.575598	https://www.genecards.org/cgi-bin/carddisp.pl?gene=UBE2J1
TAX1BP3	Tax1 Binding Protein 3	Protein Coding	38	GC17M003666	3.573484	https://www.genecards.org/cgi-bin/carddisp.pl?gene=TAX1BP3
PHF5A	PHD Finger Protein 5A	Protein Coding	37	GC22M041459	3.568998	https://www.genecards.org/cgi-bin/carddisp.pl?gene=PHF5A
ABI1	Abl Interactor 1	Protein Coding	43	GC10M026746	3.567733	https://www.genecards.org/cgi-bin/carddisp.pl?gene=ABI1
BASP1	Brain Abundant Membrane Attached Signal Protein 1	Protein Coding	37	GC05P017065	3.55845	https://www.genecards.org/cgi-bin/carddisp.pl?gene=BASP1
ANKRD30A	Ankyrin Repeat Domain 30A	Protein Coding	36	GC10P037134	3.557813	https://www.genecards.org/cgi-bin/carddisp.pl?gene=ANKRD30A
PKD1P6-NPIPP1	PKD1P6-NPIPP1 Readthrough	Pseudogene	6	GC16M015262	3.557201	https://www.genecards.org/cgi-bin/carddisp.pl?gene=PKD1P6-NPIPP1
ZFP90	ZFP90 Zinc Finger Protein	Protein Coding	36	GC16P068530	3.54882	https://www.genecards.org/cgi-bin/carddisp.pl?gene=ZFP90
OSBP	Oxysterol Binding Protein	Protein Coding	40	GC11M069113	3.544468	https://www.genecards.org/cgi-bin/carddisp.pl?gene=OSBP
NLRP2	NLR Family Pyrin Domain Containing 2	Protein Coding	42	GC19P054953	3.54446	https://www.genecards.org/cgi-bin/carddisp.pl?gene=NLRP2
FGF12	Fibroblast Growth Factor 12	Protein Coding	44	GC03M192139	3.541733	https://www.genecards.org/cgi-bin/carddisp.pl?gene=FGF12
IL36A	Interleukin 36 Alpha	Protein Coding	37	GC02P113005	3.540015	https://www.genecards.org/cgi-bin/carddisp.pl?gene=IL36A
FGF13	Fibroblast Growth Factor 13	Protein Coding	43	GC0XM138615	3.526624	https://www.genecards.org/cgi-bin/carddisp.pl?gene=FGF13

BTN2A1	Butyrophilin Subfamily 2 Member A1	Protein Coding	38	GC06P026457	3.524532	https://www.genecards.org/cgi-bin/carddisp.pl?gene=BTN2A1
HDLBP	High Density Lipoprotein Binding Protein	Protein Coding	41	GC02M241227	3.524413	https://www.genecards.org/cgi-bin/carddisp.pl?gene=HDLBP
ABCC12	ATP Binding Cassette Subfamily C Member 12	Protein Coding	40	GC16M048081	3.523999	https://www.genecards.org/cgi-bin/carddisp.pl?gene=ABCC12
ARF4	ADP Ribosylation Factor 4	Protein Coding	44	GC03M057559	3.521961	https://www.genecards.org/cgi-bin/carddisp.pl?gene=ARF4
CYB5B	Cytochrome B5 Type B	Protein Coding	42	GC16P069424	3.520857	https://www.genecards.org/cgi-bin/carddisp.pl?gene=CYB5B
UBR4	Ubiquitin Protein Ligase E3 Component N-Recognin 4	Protein Coding	39	GC01M019074	3.520182	https://www.genecards.org/cgi-bin/carddisp.pl?gene=UBR4
ITGA11	Integrin Subunit Alpha 11	Protein Coding	43	GC15M068296	3.519771	https://www.genecards.org/cgi-bin/carddisp.pl?gene=ITGA11
NCOA5	Nuclear Receptor Coactivator 5	Protein Coding	37	GC20M046060	3.519367	https://www.genecards.org/cgi-bin/carddisp.pl?gene=NCOA5
CENPT	Centromere Protein T	Protein Coding	38	GC16M067828	3.516394	https://www.genecards.org/cgi-bin/carddisp.pl?gene=CENPT
MTPN	Myotrophin	Protein Coding	36	GC07M135926	3.515093	https://www.genecards.org/cgi-bin/carddisp.pl?gene=MTPN
GNG2	G Protein Subunit Gamma 2	Protein Coding	44	GC14P051826	3.512416	https://www.genecards.org/cgi-bin/carddisp.pl?gene=GNG2
RSPO3	R-Spondin 3	Protein Coding	38	GC06P127118	3.509968	https://www.genecards.org/cgi-bin/carddisp.pl?gene=RSPO3
RERG	RAS Like Estrogen Regulated Growth Inhibitor	Protein Coding	38	GC12M015760	3.508299	https://www.genecards.org/cgi-bin/carddisp.pl?gene=RERG
DUSP3	Dual Specificity Phosphatase 3	Protein Coding	45	GC17M043766	3.50618	https://www.genecards.org/cgi-bin/carddisp.pl?gene=DUSP3
SLC26A6	Solute Carrier Family 26 Member 6	Protein Coding	39	GC03M048625	3.499781	https://www.genecards.org/cgi-bin/carddisp.pl?gene=SLC26A6
CALML4	Calmodulin Like 4	Protein Coding	34	GC15M068190	3.499511	https://www.genecards.org/cgi-bin/carddisp.pl?gene=CALML4
USP4	Ubiquitin Specific Peptidase 4	Protein Coding	43	GC03M049277	3.497966	https://www.genecards.org/cgi-bin/carddisp.pl?gene=USP4
C1QTNF3	C1q And TNF Related 3	Protein Coding	38	GC05M034017	3.497666	https://www.genecards.org/cgi-bin/carddisp.pl?gene=C1QTNF3
PPP2R5C	Protein Phosphatase 2 Regulatory Subunit B'Gamma	Protein Coding	45	GC14P106421	3.497166	https://www.genecards.org/cgi-bin/carddisp.pl?gene=PPP2R5C

TRAPPC4	Trafficking Protein Particle Complex Subunit 4	Protein Coding	41	GC11P119018	3.495054	https://www.genecards.org/cgi-bin/carddisp.pl?gene=TRAPPC4
SSR1	Signal Sequence Receptor Subunit 1	Protein Coding	41	GC06M007268	3.494134	https://www.genecards.org/cgi-bin/carddisp.pl?gene=SSR1
SNHG16	Small Nucleolar RNA Host Gene 16	RNA Gene	18	GC17P076767	3.49216	https://www.genecards.org/cgi-bin/carddisp.pl?gene=SNHG16
UBXN1	UBX Domain Protein 1	Protein Coding	36	GC11M069259	3.491509	https://www.genecards.org/cgi-bin/carddisp.pl?gene=UBXN1
MT1A	Metallothionein 1A	Protein Coding	38	GC16P056638	3.482947	https://www.genecards.org/cgi-bin/carddisp.pl?gene=MT1A
PSMB6	Proteasome 20S Subunit Beta 6	Protein Coding	43	GC17P004796	3.481221	https://www.genecards.org/cgi-bin/carddisp.pl?gene=PSMB6
NCBP1	Nuclear Cap Binding Protein Subunit 1	Protein Coding	39	GC09P097633	3.480496	https://www.genecards.org/cgi-bin/carddisp.pl?gene=NCBP1
ILF2	Interleukin Enhancer Binding Factor 2	Protein Coding	40	GC01M153661	3.477452	https://www.genecards.org/cgi-bin/carddisp.pl?gene=ILF2
VIRMA	Vir Like M6A Methyltransferase Associated	Protein Coding	27	GC08M094488	3.473283	https://www.genecards.org/cgi-bin/carddisp.pl?gene=VIRMA
TFEC	Transcription Factor EC	Protein Coding	40	GC07M115935	3.471693	https://www.genecards.org/cgi-bin/carddisp.pl?gene=TFEC
ADAT3	Adenosine Deaminase TRNA Specific 3	Protein Coding	35	GC19P001906	3.469581	https://www.genecards.org/cgi-bin/carddisp.pl?gene=ADAT3
SGIP1	SH3GL Interacting Endocytic Adaptor 1	Protein Coding	35	GC01P066533	3.46732	https://www.genecards.org/cgi-bin/carddisp.pl?gene=SGIP1
EIF3E	Eukaryotic Translation Initiation Factor 3 Subunit E	Protein Coding	39	GC08M108163	3.466106	https://www.genecards.org/cgi-bin/carddisp.pl?gene=EIF3E
VPS54	VPS54 Subunit Of GARP Complex	Protein Coding	37	GC02M063892	3.465575	https://www.genecards.org/cgi-bin/carddisp.pl?gene=VPS54
DUSP13	Dual Specificity Phosphatase 13	Protein Coding	40	GC10M075094	3.465465	https://www.genecards.org/cgi-bin/carddisp.pl?gene=DUSP13
MPIG6B	Megakaryocyte And Platelet Inhibitory Receptor G6b	Protein Coding	32	GC06P056499	3.464864	https://www.genecards.org/cgi-bin/carddisp.pl?gene=MPIG6B

ARPC2	Actin Related Protein 2/3 Complex Subunit 2	Protein Coding	43	GC02P218217	3.462652	https://www.genecards.org/cgi-bin/carddisp.pl?gene=ARPC2
RCN1	Reticulocalbin 1	Protein Coding	40	GC11P032090	3.462087	https://www.genecards.org/cgi-bin/carddisp.pl?gene=RCN1
SAP30BP	SAP30 Binding Protein	Protein Coding	38	GC17P075667	3.4575	https://www.genecards.org/cgi-bin/carddisp.pl?gene=SAP30BP
SLC25A18	Solute Carrier Family 25 Member 18	Protein Coding	40	GC22P017689	3.454096	https://www.genecards.org/cgi-bin/carddisp.pl?gene=SLC25A18
RYBP	RING1 And YY1 Binding Protein	Protein Coding	41	GC03M072371	3.453499	https://www.genecards.org/cgi-bin/carddisp.pl?gene=RYBP
MATK	Megakaryocyte-Associated Tyrosine Kinase	Protein Coding	46	GC19M003777	3.453198	https://www.genecards.org/cgi-bin/carddisp.pl?gene=MATK
USP48	Ubiquitin Specific Peptidase 48	Protein Coding	40	GC01M021678	3.444248	https://www.genecards.org/cgi-bin/carddisp.pl?gene=USP48
LINC02694	Long Intergenic Non-Protein Coding RNA 2694	RNA Gene	23	GC15P038622	3.443242	https://www.genecards.org/cgi-bin/carddisp.pl?gene=LINC02694
WDR20	WD Repeat Domain 20	Protein Coding	35	GC14P106033	3.442783	https://www.genecards.org/cgi-bin/carddisp.pl?gene=WDR20
CIDEB	Cell Death Inducing DFFA Like Effector B	Protein Coding	37	GC14M024305	3.441411	https://www.genecards.org/cgi-bin/carddisp.pl?gene=CIDEB
LOC643339	Uncharacterized LOC643339	RNA Gene	9	GC12M093005	3.432151	https://www.genecards.org/cgi-bin/carddisp.pl?gene=LOC643339
HSD17B7	Hydroxysteroid 17-Beta Dehydrogenase 7	Protein Coding	44	GC01P162790	3.429786	https://www.genecards.org/cgi-bin/carddisp.pl?gene=HSD17B7
VSIG4	V-Set And Immunoglobulin Domain Containing 4	Protein Coding	37	GC0XM066021	3.427186	https://www.genecards.org/cgi-bin/carddisp.pl?gene=VSIG4
ZNF629	Zinc Finger Protein 629	Protein Coding	31	GC16M031317	3.426744	https://www.genecards.org/cgi-bin/carddisp.pl?gene=ZNF629
SMC4	Structural Maintenance Of Chromosomes 4	Protein Coding	40	GC03P160399	3.426485	https://www.genecards.org/cgi-bin/carddisp.pl?gene=SMC4
TIMM22	Translocase Of Inner Mitochondrial Membrane 22	Protein Coding	37	GC17P000997	3.425393	https://www.genecards.org/cgi-bin/carddisp.pl?gene=TIMM22
UBR2	Ubiquitin Protein Ligase E3 Component N-Recognin 2	Protein Coding	40	GC06P055398	3.423468	https://www.genecards.org/cgi-bin/carddisp.pl?gene=UBR2

RNU6-53P	RNA, U6 Small Nuclear 53, Pseudogene	Pseudogene	8	GC13M028703	3.423291	https://www.genecards.org/cgi-bin/carddisp.pl?gene=RNU6-53P
SLC24A3	Solute Carrier Family 24 Member 3	Protein Coding	40	GC20P019212	3.42275	https://www.genecards.org/cgi-bin/carddisp.pl?gene=SLC24A3
LASP1	LIM And SH3 Protein 1	Protein Coding	43	GC17P038869	3.422331	https://www.genecards.org/cgi-bin/carddisp.pl?gene=LASP1
JRKL	JRK Like	Protein Coding	33	GC11P096389	3.421736	https://www.genecards.org/cgi-bin/carddisp.pl?gene=JRKL
PTDSS2	Phosphatidylserine Synthase 2	Protein Coding	37	GC11P000448	3.421223	https://www.genecards.org/cgi-bin/carddisp.pl?gene=PTDSS2
TIAM1	TIAM Rac1 Associated GEF 1	Protein Coding	45	GC21M031118	3.420755	https://www.genecards.org/cgi-bin/carddisp.pl?gene=TIAM1
SCGB2A2	Secretoglobin Family 2A Member 2	Protein Coding	37	GC11P062269	3.417606	https://www.genecards.org/cgi-bin/carddisp.pl?gene=SCGB2A2
MAPKAPK3	MAPK Activated Protein Kinase 3	Protein Coding	50	GC03P050611	3.413744	https://www.genecards.org/cgi-bin/carddisp.pl?gene=MAPKAPK3
HIGD1A	HIG1 Hypoxia Inducible Domain Family Member 1A	Protein Coding	38	GC03M042782	3.410537	https://www.genecards.org/cgi-bin/carddisp.pl?gene=HIGD1A
GPR161	G Protein-Coupled Receptor 161	Protein Coding	41	GC01M168080	3.408909	https://www.genecards.org/cgi-bin/carddisp.pl?gene=GPR161
NDUFA4L2	NDUFA4 Mitochondrial Complex Associated Like 2	Protein Coding	37	GC12M057234	3.407495	https://www.genecards.org/cgi-bin/carddisp.pl?gene=NDUFA4L2
HSD17B14	Hydroxysteroid 17-Beta Dehydrogenase 14	Protein Coding	38	GC19M048813	3.405724	https://www.genecards.org/cgi-bin/carddisp.pl?gene=HSD17B14
APPBP2	Amyloid Beta Precursor Protein Binding Protein 2	Protein Coding	37	GC17M060443	3.401546	https://www.genecards.org/cgi-bin/carddisp.pl?gene=APPBP2
MYBBP1A	MYB Binding Protein 1a	Protein Coding	40	GC17M004538	3.400179	https://www.genecards.org/cgi-bin/carddisp.pl?gene=MYBBP1A
XPO4	Exportin 4	Protein Coding	38	GC13M020777	3.394728	https://www.genecards.org/cgi-bin/carddisp.pl?gene=XPO4
CAPZA1	Capping Actin Protein Of Muscle Z-Line Subunit Alpha 1	Protein Coding	42	GC01P112619	3.394477	https://www.genecards.org/cgi-bin/carddisp.pl?gene=CAPZA1
LGSN	Lengsin, Lens Protein With Glutamine Synthetase Domain	Protein Coding	34	GC06M063275	3.39157	https://www.genecards.org/cgi-bin/carddisp.pl?gene=LGSN

EIF3J	Eukaryotic Translation Initiation Factor 3 Subunit J	Protein Coding	40	GC15P044537	3.389582	https://www.genecards.org/cgi-bin/carddisp.pl?gene=EIF3J
ENSG00000266919		RNA Gene	5	GC17M031119	3.388794	https://www.genecards.org/cgi-bin/carddisp.pl?gene=ENSG00000266919
HIPK3	Homeodomain Interacting Protein Kinase 3	Protein Coding	43	GC11P033278	3.386807	https://www.genecards.org/cgi-bin/carddisp.pl?gene=HIPK3
EIF3K	Eukaryotic Translation Initiation Factor 3 Subunit K	Protein Coding	39	GC19P038619	3.385266	https://www.genecards.org/cgi-bin/carddisp.pl?gene=EIF3K
ZFYVE9	Zinc Finger FYVE-Type Containing 9	Protein Coding	43	GC01P052142	3.384112	https://www.genecards.org/cgi-bin/carddisp.pl?gene=ZFYVE9
TLCD4	TLC Domain Containing 4	Protein Coding	27	GC01P095092	3.381307	https://www.genecards.org/cgi-bin/carddisp.pl?gene=TLCD4
ECT2	Epithelial Cell Transforming 2	Protein Coding	41	GC03P172750	3.38014	https://www.genecards.org/cgi-bin/carddisp.pl?gene=ECT2
MAD2L1BP	MAD2L1 Binding Protein	Protein Coding	36	GC06P043629	3.378428	https://www.genecards.org/cgi-bin/carddisp.pl?gene=MAD2L1BP
MAPK6	Mitogen-Activated Protein Kinase 6	Protein Coding	45	GC15P051952	3.3756	https://www.genecards.org/cgi-bin/carddisp.pl?gene=MAPK6
CCDC141	Coiled-Coil Domain Containing 141	Protein Coding	36	GC02M178829	3.375553	https://www.genecards.org/cgi-bin/carddisp.pl?gene=CCDC141
CKAP4	Cytoskeleton Associated Protein 4	Protein Coding	38	GC12M106237	3.37349	https://www.genecards.org/cgi-bin/carddisp.pl?gene=CKAP4
AHCYL1	Adenosylhomocysteinase Like 1	Protein Coding	44	GC01P109984	3.372233	https://www.genecards.org/cgi-bin/carddisp.pl?gene=AHCYL1
ZBTB7A	Zinc Finger And BTB Domain Containing 7A	Protein Coding	37	GC19M004045	3.370528	https://www.genecards.org/cgi-bin/carddisp.pl?gene=ZBTB7A
MYO18B	Myosin XVIIIB	Protein Coding	39	GC22P025742	3.368923	https://www.genecards.org/cgi-bin/carddisp.pl?gene=MYO18B
ADAMTS9-AS2	ADAMTS9 Antisense RNA 2	RNA Gene	16	GC03P064671	3.368214	https://www.genecards.org/cgi-bin/carddisp.pl?gene=ADAMTS9-AS2
RPL32	Ribosomal Protein L32	Protein Coding	40	GC03M012834	3.36744	https://www.genecards.org/cgi-bin/carddisp.pl?gene=RPL32
LINC00460	Long Intergenic Non-Protein Coding RNA 460	RNA Gene	16	GC13P106376	3.364998	https://www.genecards.org/cgi-bin/carddisp.pl?gene=LINC00460

PSG2	Pregnancy Specific Beta-1-Glycoprotein 2	Protein Coding	34	GC19M043064	3.364372	https://www.genecards.org/cgi-bin/carddisp.pl?gene=PSG2
POC1A	POC1 Centriolar Protein A	Protein Coding	38	GC03M052109	3.354208	https://www.genecards.org/cgi-bin/carddisp.pl?gene=POC1A
ICE2	Interactor Of Little Elongation Complex ELL Subunit 2	Protein Coding	33	GC15M060419	3.352345	https://www.genecards.org/cgi-bin/carddisp.pl?gene=ICE2
CCNK	Cyclin K	Protein Coding	43	GC14P099483	3.352274	https://www.genecards.org/cgi-bin/carddisp.pl?gene=CCNK
KIF13B	Kinesin Family Member 13B	Protein Coding	41	GC08M029067	3.350354	https://www.genecards.org/cgi-bin/carddisp.pl?gene=KIF13B
PLXNA4	Plexin A4	Protein Coding	40	GC07M132123	3.346508	https://www.genecards.org/cgi-bin/carddisp.pl?gene=PLXNA4
RFX3	Regulatory Factor X3	Protein Coding	40	GC09M003214	3.345685	https://www.genecards.org/cgi-bin/carddisp.pl?gene=RFX3
ORC5	Origin Recognition Complex Subunit 5	Protein Coding	37	GC07M104126	3.339668	https://www.genecards.org/cgi-bin/carddisp.pl?gene=ORC5
FGF6	Fibroblast Growth Factor 6	Protein Coding	40	GC12M004429	3.3385	https://www.genecards.org/cgi-bin/carddisp.pl?gene=FGF6
OLIG1	Oligodendrocyte Transcription Factor 1	Protein Coding	38	GC21P033070	3.337327	https://www.genecards.org/cgi-bin/carddisp.pl?gene=OLIG1
LINC01405	Long Intergenic Non-Protein Coding RNA 1405	RNA Gene	15	GC12P110936	3.332382	https://www.genecards.org/cgi-bin/carddisp.pl?gene=LINC01405
SND1	Staphylococcal Nuclease And Tudor Domain Containing 1	Protein Coding	43	GC07P127652	3.329912	https://www.genecards.org/cgi-bin/carddisp.pl?gene=SND1
LRRFIP2	LRR Binding FLII Interacting Protein 2	Protein Coding	38	GC03M037052	3.320521	https://www.genecards.org/cgi-bin/carddisp.pl?gene=LRRFIP2
SVIL-AS1	SVIL Antisense RNA 1	RNA Gene	13	GC10P029410	3.319946	https://www.genecards.org/cgi-bin/carddisp.pl?gene=SVIL-AS1
MIR345	MicroRNA 345	RNA Gene	19	GC14P100307	3.316504	https://www.genecards.org/cgi-bin/carddisp.pl?gene=MIR345
CLDND1	Claudin Domain Containing 1	Protein Coding	37	GC03M098497	3.315968	https://www.genecards.org/cgi-bin/carddisp.pl?gene=CLDND1
IMPA1	Inositol Monophosphatase 1	Protein Coding	49	GC08M081656	3.315798	https://www.genecards.org/cgi-bin/carddisp.pl?gene=IMPA1
RAB37	RAB37, Member RAS Oncogene Family	Protein Coding	39	GC17P074671	3.313342	https://www.genecards.org/cgi-bin/carddisp.pl?gene=RAB37

ICAM5	Intercellular Adhesion Molecule 5	Protein Coding	40	GC19P010289	3.311417	https://www.genecards.org/cgi-bin/carddisp.pl?gene=ICAM5
MRTFB	Myocardin Related Transcription Factor B	Protein Coding	30	GC16P014071	3.309253	https://www.genecards.org/cgi-bin/carddisp.pl?gene=MRTFB
BMP10	Bone Morphogenetic Protein 10	Protein Coding	39	GC02M068865	3.306949	https://www.genecards.org/cgi-bin/carddisp.pl?gene=BMP10
RBM6	RNA Binding Motif Protein 6	Protein Coding	37	GC03P049940	3.302695	https://www.genecards.org/cgi-bin/carddisp.pl?gene=RBM6
MSBP2	Minisatellite Binding Protein 2	Protein Coding	4	GC00U990214	3.301336	https://www.genecards.org/cgi-bin/carddisp.pl?gene=MSBP2
MLEC	Malectin	Protein Coding	37	GC12P120687	3.301245	https://www.genecards.org/cgi-bin/carddisp.pl?gene=MLEC
RNH1	Ribonuclease/Angiogenin Inhibitor 1	Protein Coding	38	GC11M001079	3.296893	https://www.genecards.org/cgi-bin/carddisp.pl?gene=RNH1
SMOC2	SPARC Related Modular Calcium Binding 2	Protein Coding	42	GC06P168441	3.296794	https://www.genecards.org/cgi-bin/carddisp.pl?gene=SMOC2
PLEKHA8	Pleckstrin Homology Domain Containing A8	Protein Coding	37	GC07P030027	3.296571	https://www.genecards.org/cgi-bin/carddisp.pl?gene=PLEKHA8
SLC16A10	Solute Carrier Family 16 Member 10	Protein Coding	40	GC06P111087	3.293602	https://www.genecards.org/cgi-bin/carddisp.pl?gene=SLC16A10
SUB1	SUB1 Regulator Of Transcription	Protein Coding	39	GC05P032540	3.287555	https://www.genecards.org/cgi-bin/carddisp.pl?gene=SUB1
ACYP2	Acylphosphatase 2	Protein Coding	40	GC02P053970	3.286894	https://www.genecards.org/cgi-bin/carddisp.pl?gene=ACYP2
LATS2	Large Tumor Suppressor Kinase 2	Protein Coding	46	GC13M020973	3.286833	https://www.genecards.org/cgi-bin/carddisp.pl?gene=LATS2
PTGER1	Prostaglandin E Receptor 1	Protein Coding	41	GC19M014444	3.286324	https://www.genecards.org/cgi-bin/carddisp.pl?gene=PTGER1
ZMYND8	Zinc Finger MYND-Type Containing 8	Protein Coding	40	GC20M047209	3.285129	https://www.genecards.org/cgi-bin/carddisp.pl?gene=ZMYND8
MAGI1	Membrane Associated Guanylate Kinase, WW And PDZ Domain Containing 1	Protein Coding	41	GC03M065330	3.282785	https://www.genecards.org/cgi-bin/carddisp.pl?gene=MAGI1
P2RY1	Purinergic Receptor P2Y1	Protein Coding	47	GC03P152835	3.282743	https://www.genecards.org/cgi-bin/carddisp.pl?gene=P2RY1
MTA3	Metastasis Associated 1 Family Member 3	Protein Coding	43	GC02P042494	3.279561	https://www.genecards.org/cgi-bin/carddisp.pl?gene=MTA3

GNL3	G Protein Nucleolar 3	Protein Coding	41	GC03P052681	3.279166	https://www.genecards.org/cgi-bin/carddisp.pl?gene=GNL3
ARHGEF7	Rho Guanine Nucleotide Exchange Factor 7	Protein Coding	44	GC13P111114	3.274893	https://www.genecards.org/cgi-bin/carddisp.pl?gene=ARHGEF7
PRPF38B	Pre-MRNA Processing Factor 38B	Protein Coding	33	GC01P108692	3.273608	https://www.genecards.org/cgi-bin/carddisp.pl?gene=PRPF38B
TAX1BP1	Tax1 Binding Protein 1	Protein Coding	41	GC07P027739	3.272031	https://www.genecards.org/cgi-bin/carddisp.pl?gene=TAX1BP1
CEACAM7	CEA Cell Adhesion Molecule 7	Protein Coding	38	GC19M041673	3.269249	https://www.genecards.org/cgi-bin/carddisp.pl?gene=CEACAM7
JMJD6	Jumonji Domain Containing 6, Arginine Demethylase And Lysine Hydroxylase	Protein Coding	43	GC17M076718	3.268786	https://www.genecards.org/cgi-bin/carddisp.pl?gene=JMJD6
XCL1	X-C Motif Chemokine Ligand 1	Protein Coding	38	GC01P168576	3.263575	https://www.genecards.org/cgi-bin/carddisp.pl?gene=XCL1
FOXN3	Forkhead Box N3	Protein Coding	39	GC14M095787	3.257763	https://www.genecards.org/cgi-bin/carddisp.pl?gene=FOXN3
XRN1	5'-3' Exoribonuclease 1	Protein Coding	36	GC03M142306	3.257639	https://www.genecards.org/cgi-bin/carddisp.pl?gene=XRN1
HEATR3	HEAT Repeat Containing 3	Protein Coding	31	GC16P050065	3.256466	https://www.genecards.org/cgi-bin/carddisp.pl?gene=HEATR3
RANBP9	RAN Binding Protein 9	Protein Coding	42	GC06M013621	3.254754	https://www.genecards.org/cgi-bin/carddisp.pl?gene=RANBP9
NTAN1	N-Terminal Asparagine Amidase	Protein Coding	38	GC16M015037	3.254645	https://www.genecards.org/cgi-bin/carddisp.pl?gene=NTAN1
SESN3	Sestrin 3	Protein Coding	40	GC11M095166	3.249538	https://www.genecards.org/cgi-bin/carddisp.pl?gene=SESN3
RLN2	Relaxin 2	Protein Coding	37	GC09M005306	3.248283	https://www.genecards.org/cgi-bin/carddisp.pl?gene=RLN2
RGL1	Ral Guanine Nucleotide Dissociation Stimulator Like 1	Protein Coding	40	GC01P183636	3.247843	https://www.genecards.org/cgi-bin/carddisp.pl?gene=RGL1
S100A2	S100 Calcium Binding Protein A2	Protein Coding	41	GC01M153561	3.242202	https://www.genecards.org/cgi-bin/carddisp.pl?gene=S100A2
PANK3	Pantothenate Kinase 3	Protein Coding	40	GC05M168549	3.240339	https://www.genecards.org/cgi-bin/carddisp.pl?gene=PANK3
TNPO1	Transportin 1	Protein Coding	41	GC05P072816	3.236463	https://www.genecards.org/cgi-bin/carddisp.pl?gene=TNPO1
STK16	Serine/Threonine Kinase 16	Protein Coding	41	GC02P219248	3.236006	https://www.genecards.org/cgi-bin/carddisp.pl?gene=STK16
CHD9	Chromodomain Helicase DNA Binding Protein 9	Protein Coding	38	GC16P053041	3.233136	https://www.genecards.org/cgi-bin/carddisp.pl?gene=CHD9

GSTT2	Glutathione S-Transferase Theta 2 (Gene/Pseudogene)	Protein Coding	36	GC22P023980	3.232046	https://www.genecards.org/cgi-bin/carddisp.pl?gene=GSTT2
GAR1	GAR1 Ribonucleoprotein	Protein Coding	37	GC04P109815	3.223464	https://www.genecards.org/cgi-bin/carddisp.pl?gene=GAR1
ITGA9	Integrin Subunit Alpha 9	Protein Coding	44	GC03P037468	3.222739	https://www.genecards.org/cgi-bin/carddisp.pl?gene=ITGA9
USP10	Ubiquitin Specific Peptidase 10	Protein Coding	44	GC16P084734	3.22244	https://www.genecards.org/cgi-bin/carddisp.pl?gene=USP10
SAFB2	Scaffold Attachment Factor B2	Protein Coding	37	GC19M005587	3.219259	https://www.genecards.org/cgi-bin/carddisp.pl?gene=SAFB2
H3C2	H3 Clustered Histone 2	Protein Coding	31	GC06M026032	3.216128	https://www.genecards.org/cgi-bin/carddisp.pl?gene=H3C2
WASF3	WASP Family Member 3	Protein Coding	41	GC13P026557	3.214986	https://www.genecards.org/cgi-bin/carddisp.pl?gene=WASF3
VAMP4	Vesicle Associated Membrane Protein 4	Protein Coding	43	GC01M171700	3.209022	https://www.genecards.org/cgi-bin/carddisp.pl?gene=VAMP4
GJD3	Gap Junction Protein Delta 3	Protein Coding	33	GC17M040360	3.208241	https://www.genecards.org/cgi-bin/carddisp.pl?gene=GJD3
ELP2	Elongator Acetyltransferase Complex Subunit 2	Protein Coding	38	GC18P036129	3.206686	https://www.genecards.org/cgi-bin/carddisp.pl?gene=ELP2
TRMT44	TRNA Methyltransferase 44 Homolog	Protein Coding	32	GC04P008439	3.204755	https://www.genecards.org/cgi-bin/carddisp.pl?gene=TRMT44
FAM3B	FAM3 Metabolism Regulating Signaling Molecule B	Protein Coding	39	GC21P041304	3.203745	https://www.genecards.org/cgi-bin/carddisp.pl?gene=FAM3B
SLC9A4	Solute Carrier Family 9 Member A4	Protein Coding	36	GC02P102456	3.202937	https://www.genecards.org/cgi-bin/carddisp.pl?gene=SLC9A4
FBXW11	F-Box And WD Repeat Domain Containing 11	Protein Coding	45	GC05M171861	3.19717	https://www.genecards.org/cgi-bin/carddisp.pl?gene=FBXW11
HSALNG0013825		RNA Gene	1	GC02P027507	3.196733	https://www.genecards.org/cgi-bin/carddisp.pl?gene=HSALNG0013825
SNRNP40	Small Nuclear Ribonucleoprotein U5 Subunit 40	Protein Coding	37	GC01M031259	3.196248	https://www.genecards.org/cgi-bin/carddisp.pl?gene=SNRNP40
ZDHHC20	Zinc Finger DHHC-Type Palmitoyltransferase 20	Protein Coding	37	GC13M021372	3.194844	https://www.genecards.org/cgi-bin/carddisp.pl?gene=ZDHHC20

CCDC26	CCDC26 Long Non-Coding RNA	RNA Gene	26	GC08M128925	3.185689	https://www.genecards.org/cgi-bin/carddisp.pl?gene=CCDC26
MED4	Mediator Complex Subunit 4	Protein Coding	38	GC13M048053	3.184414	https://www.genecards.org/cgi-bin/carddisp.pl?gene=MED4
ALX1	ALX Homeobox 1	Protein Coding	40	GC12P085279	3.178168	https://www.genecards.org/cgi-bin/carddisp.pl?gene=ALX1
ASRGL1	Asparaginase And Isoaspartyl Peptidase 1	Protein Coding	43	GC11P062337	3.173931	https://www.genecards.org/cgi-bin/carddisp.pl?gene=ASRGL1
SRRM2	Serine/Arginine Repetitive Matrix 2	Protein Coding	34	GC16P005607	3.173657	https://www.genecards.org/cgi-bin/carddisp.pl?gene=SRRM2
LOC101929710	Uncharacterized LOC101929710	RNA Gene	11	GC05P095962	3.171996	https://www.genecards.org/cgi-bin/carddisp.pl?gene=LOC101929710
PLPP7	Phospholipid Phosphatase 7 (Inactive)	Protein Coding	28	GC09P131290	3.164884	https://www.genecards.org/cgi-bin/carddisp.pl?gene=PLPP7
POLM	DNA Polymerase Mu	Protein Coding	40	GC07M044143	3.164086	https://www.genecards.org/cgi-bin/carddisp.pl?gene=POLM
COX7B2	Cytochrome C Oxidase Subunit 7B2	Protein Coding	32	GC04M046734	3.158653	https://www.genecards.org/cgi-bin/carddisp.pl?gene=COX7B2
COTL1	Coactosin Like F-Actin Binding Protein 1	Protein Coding	41	GC16M084566	3.15034	https://www.genecards.org/cgi-bin/carddisp.pl?gene=COTL1
BZW1	Basic Leucine Zipper And W2 Domains 1	Protein Coding	36	GC02P200810	3.149478	https://www.genecards.org/cgi-bin/carddisp.pl?gene=BZW1
TUSC1	Tumor Suppressor Candidate 1	Protein Coding	30	GC09M025668	3.146367	https://www.genecards.org/cgi-bin/carddisp.pl?gene=TUSC1
WSPAR	WNT Signaling Pathway Activating Non-Coding RNA	RNA Gene	11	GC05P133914	3.146203	https://www.genecards.org/cgi-bin/carddisp.pl?gene=WSPAR
SLC39A7	Solute Carrier Family 39 Member 7	Protein Coding	41	GC06P033200	3.13961	https://www.genecards.org/cgi-bin/carddisp.pl?gene=SLC39A7
ERMN	Ermin	Protein Coding	36	GC02M157318	3.136434	https://www.genecards.org/cgi-bin/carddisp.pl?gene=ERMN
SPDEF	SAM Pointed Domain Containing ETS Transcription Factor	Protein Coding	40	GC06M047018	3.135393	https://www.genecards.org/cgi-bin/carddisp.pl?gene=SPDEF
CCL28	C-C Motif Chemokine Ligand 28	Protein Coding	41	GC05M043356	3.135077	https://www.genecards.org/cgi-bin/carddisp.pl?gene=CCL28
DDX27	DEAD-Box Helicase 27	Protein Coding	36	GC20P049219	3.134715	https://www.genecards.org/cgi-bin/carddisp.pl?gene=DDX27

PRMT2	Protein Arginine Methyltransferase 2	Protein Coding	42	GC21P046635	3.130123	https://www.genecards.org/cgi-bin/carddisp.pl?gene=PRMT2
MYL6B	Myosin Light Chain 6B	Protein Coding	40	GC12P056388	3.127315	https://www.genecards.org/cgi-bin/carddisp.pl?gene=MYL6B
RGS10	Regulator Of G Protein Signaling 10	Protein Coding	44	GC10M119499	3.124254	https://www.genecards.org/cgi-bin/carddisp.pl?gene=RGS10
DIRC3	Disrupted In Renal Carcinoma 3	RNA Gene	22	GC02M217284	3.123611	https://www.genecards.org/cgi-bin/carddisp.pl?gene=DIRC3
CERS6	Ceramide Synthase 6	Protein Coding	38	GC02P168455	3.123132	https://www.genecards.org/cgi-bin/carddisp.pl?gene=CERS6
YES1	YES Proto-Oncogene 1, Src Family Tyrosine Kinase	Protein Coding	49	GC18M000721	3.119767	https://www.genecards.org/cgi-bin/carddisp.pl?gene=YES1
LINC00917	Long Intergenic Non-Protein Coding RNA 917	RNA Gene	15	GC16M086366	3.117719	https://www.genecards.org/cgi-bin/carddisp.pl?gene=LINC00917
TAMM41	TAM41 Mitochondrial Translocator Assembly And Maintenance Homolog	Protein Coding	33	GC03M011721	3.115762	https://www.genecards.org/cgi-bin/carddisp.pl?gene=TAMM41
NASP	Nuclear Autoantigenic Sperm Protein	Protein Coding	37	GC01P045583	3.11498	https://www.genecards.org/cgi-bin/carddisp.pl?gene=NASP
ZNF569	Zinc Finger Protein 569	Protein Coding	34	GC19M046714	3.113239	https://www.genecards.org/cgi-bin/carddisp.pl?gene=ZNF569
ARHGAP8	Rho GTPase Activating Protein 8	Protein Coding	34	GC22P044752	3.110983	https://www.genecards.org/cgi-bin/carddisp.pl?gene=ARHGAP8
CLTCL1	Clathrin Heavy Chain Like 1	Protein Coding	41	GC22M019180	3.110639	https://www.genecards.org/cgi-bin/carddisp.pl?gene=CLTCL1
ST18	ST18 C2H2C-Type Zinc Finger Transcription Factor	Protein Coding	38	GC08M052110	3.109979	https://www.genecards.org/cgi-bin/carddisp.pl?gene=ST18
INTS6	Integrator Complex Subunit 6	Protein Coding	38	GC13M051354	3.108181	https://www.genecards.org/cgi-bin/carddisp.pl?gene=INTS6
DUSP26	Dual Specificity Phosphatase 26	Protein Coding	37	GC08M033591	3.105825	https://www.genecards.org/cgi-bin/carddisp.pl?gene=DUSP26
PIGU	Phosphatidylinositol Glycan Anchor Biosynthesis Class U	Protein Coding	37	GC20M034560	3.103941	https://www.genecards.org/cgi-bin/carddisp.pl?gene=PIGU

SLC39A6	Solute Carrier Family 39 Member 6	Protein Coding	40	GC18M036108	3.103225	https://www.genecards.org/cgi-bin/carddisp.pl?gene=SLC39A6
MED20	Mediator Complex Subunit 20	Protein Coding	37	GC06M041905	3.102738	https://www.genecards.org/cgi-bin/carddisp.pl?gene=MED20
KLF12	Kruppel Like Factor 12	Protein Coding	39	GC13M073686	3.099366	https://www.genecards.org/cgi-bin/carddisp.pl?gene=KLF12
VWA5A	Von Willebrand Factor A Domain Containing 5A	Protein Coding	34	GC11P124115	3.098876	https://www.genecards.org/cgi-bin/carddisp.pl?gene=VWA5A
FAM3A	FAM3 Metabolism Regulating Signaling Molecule A	Protein Coding	37	GC0XM154506	3.098682	https://www.genecards.org/cgi-bin/carddisp.pl?gene=FAM3A
PUS7	Pseudouridine Synthase 7	Protein Coding	41	GC07M105439	3.096957	https://www.genecards.org/cgi-bin/carddisp.pl?gene=PUS7
DEFA1	Defensin Alpha 1	Protein Coding	41	GC08M006977	3.092986	https://www.genecards.org/cgi-bin/carddisp.pl?gene=DEFA1
KRTAP5-5	Keratin Associated Protein 5-5	Protein Coding	26	GC11P001629	3.092125	https://www.genecards.org/cgi-bin/carddisp.pl?gene=KRTAP5-5
SEC14L1	SEC14 Like Lipid Binding 1	Protein Coding	39	GC17P077086	3.087129	https://www.genecards.org/cgi-bin/carddisp.pl?gene=SEC14L1
DDX46	DEAD-Box Helicase 46	Protein Coding	40	GC05P134758	3.086512	https://www.genecards.org/cgi-bin/carddisp.pl?gene=DDX46
BHLHA9	Basic Helix-Loop-Helix Family Member A9	Protein Coding	28	GC17P001270	3.085235	https://www.genecards.org/cgi-bin/carddisp.pl?gene=BHLHA9
AVEN	Apoptosis And Caspase Activation Inhibitor	Protein Coding	37	GC15M033853	3.084106	https://www.genecards.org/cgi-bin/carddisp.pl?gene=AVEN
NBPF1	NBPF Member 1	Protein Coding	31	GC01M016562	3.081224	https://www.genecards.org/cgi-bin/carddisp.pl?gene=NBPF1
KLHL6	Kelch Like Family Member 6	Protein Coding	36	GC03M183487	3.080543	https://www.genecards.org/cgi-bin/carddisp.pl?gene=KLHL6
ASAH2B	N-Acylsphingosine Amidohydrolase 2B	Protein Coding	27	GC10P050739	3.079664	https://www.genecards.org/cgi-bin/carddisp.pl?gene=ASAH2B
CDK19	Cyclin Dependent Kinase 19	Protein Coding	43	GC06M110609	3.078501	https://www.genecards.org/cgi-bin/carddisp.pl?gene=CDK19
CDX1	Caudal Type Homeobox 1	Protein Coding	37	GC05P150166	3.071558	https://www.genecards.org/cgi-bin/carddisp.pl?gene=CDX1
MIR181D	MicroRNA 181d	RNA Gene	18	GC19P013874	3.067154	https://www.genecards.org/cgi-bin/carddisp.pl?gene=MIR181D
SLC5A11	Solute Carrier Family 5 Member 11	Protein Coding	41	GC16P024859	3.061815	https://www.genecards.org/cgi-bin/carddisp.pl?gene=SLC5A11

LOC110973015	NOS3 5' Regulatory Region	Biological Region	2	GC07P150988	3.061494	https://www.genecards.org/cgi-bin/carddisp.pl?gene=LOC110973015
CDH8	Cadherin 8	Protein Coding	41	GC16M061647	3.059145	https://www.genecards.org/cgi-bin/carddisp.pl?gene=CDH8
KCNIP4	Potassium Voltage-Gated Channel Interacting Protein 4	Protein Coding	39	GC04M020728	3.058533	https://www.genecards.org/cgi-bin/carddisp.pl?gene=KCNIP4
SNHG18	Small Nucleolar RNA Host Gene 18	RNA Gene	15	GC05P009562	3.055027	https://www.genecards.org/cgi-bin/carddisp.pl?gene=SNHG18
H2AC4	H2A Clustered Histone 4	Protein Coding	30	GC06M026034	3.052876	https://www.genecards.org/cgi-bin/carddisp.pl?gene=H2AC4
FNBP1L	Formin Binding Protein 1 Like	Protein Coding	36	GC01P093448	3.051878	https://www.genecards.org/cgi-bin/carddisp.pl?gene=FNBP1L
PAXIP1	PAX Interacting Protein 1	Protein Coding	37	GC07M154943	3.049388	https://www.genecards.org/cgi-bin/carddisp.pl?gene=PAXIP1
GREB1	Growth Regulating Estrogen Receptor Binding 1	Protein Coding	37	GC02P011482	3.048043	https://www.genecards.org/cgi-bin/carddisp.pl?gene=GREB1
LIMK2	LIM Domain Kinase 2	Protein Coding	48	GC22P031212	3.04756	https://www.genecards.org/cgi-bin/carddisp.pl?gene=LIMK2
COL21A1	Collagen Type XXI Alpha 1 Chain	Protein Coding	37	GC06M056057	3.045572	https://www.genecards.org/cgi-bin/carddisp.pl?gene=COL21A1
LINC01016	Long Intergenic Non-Protein Coding RNA 1016	RNA Gene	13	GC06M047659	3.04506	https://www.genecards.org/cgi-bin/carddisp.pl?gene=LINC01016
HAS1	Hyaluronan Synthase 1	Protein Coding	40	GC19M051714	3.044197	https://www.genecards.org/cgi-bin/carddisp.pl?gene=HAS1
EPS15L1	Epidermal Growth Factor Receptor Pathway Substrate 15 Like 1	Protein Coding	41	GC19M016333	3.040388	https://www.genecards.org/cgi-bin/carddisp.pl?gene=EPS15L1
GNAZ	G Protein Subunit Alpha Z	Protein Coding	45	GC22P023070	3.037711	https://www.genecards.org/cgi-bin/carddisp.pl?gene=GNAZ
HBP1	HMG-Box Transcription Factor 1	Protein Coding	39	GC07P107168	3.036912	https://www.genecards.org/cgi-bin/carddisp.pl?gene=HBP1
EIF4A2	Eukaryotic Translation Initiation Factor 4A2	Protein Coding	45	GC03P186783	3.035983	https://www.genecards.org/cgi-bin/carddisp.pl?gene=EIF4A2
TLN2	Talin 2	Protein Coding	40	GC15P062390	3.034485	https://www.genecards.org/cgi-bin/carddisp.pl?gene=TLN2

PWWP3A	PWWP Domain Containing 3A, DNA Repair Factor	Protein Coding	21	GC19P001625	3.027288	https://www.genecards.org/cgi-bin/carddisp.pl?gene=PWWP3A
PPCS	Phosphopantothenoylcysteine Synthetase	Protein Coding	40	GC01P042456	3.023721	https://www.genecards.org/cgi-bin/carddisp.pl?gene=PPCS
TIMM8B	Translocase Of Inner Mitochondrial Membrane 8 Homolog B	Protein Coding	38	GC11M112084	3.023216	https://www.genecards.org/cgi-bin/carddisp.pl?gene=TIMM8B
CDYL	Chromodomain Y Like	Protein Coding	41	GC06P004706	3.021195	https://www.genecards.org/cgi-bin/carddisp.pl?gene=CDYL
MAU2	MAU2 Sister Chromatid Cohesion Factor	Protein Coding	33	GC19P019320	3.019058	https://www.genecards.org/cgi-bin/carddisp.pl?gene=MAU2
KDM4B	Lysine Demethylase 4B	Protein Coding	44	GC19P004969	3.015503	https://www.genecards.org/cgi-bin/carddisp.pl?gene=KDM4B
SGK3	Serum/Glucocorticoid Regulated Kinase Family Member 3	Protein Coding	43	GC08P066712	3.014399	https://www.genecards.org/cgi-bin/carddisp.pl?gene=SGK3
CYB561	Cytochrome B561	Protein Coding	41	GC17M063432	3.011662	https://www.genecards.org/cgi-bin/carddisp.pl?gene=CYB561
AMOTL1	Angiomotin Like 1	Protein Coding	38	GC11P094686	3.004309	https://www.genecards.org/cgi-bin/carddisp.pl?gene=AMOTL1
GTPBP2	GTP Binding Protein 2	Protein Coding	39	GC06M043605	3.004073	https://www.genecards.org/cgi-bin/carddisp.pl?gene=GTPBP2
MIOX	Myo-Inositol Oxygenase	Protein Coding	39	GC22P050486	3.00245	https://www.genecards.org/cgi-bin/carddisp.pl?gene=MIOX
UIMC1	Ubiquitin Interaction Motif Containing 1	Protein Coding	41	GC05M176905	2.999886	https://www.genecards.org/cgi-bin/carddisp.pl?gene=UIMC1
MIR1183	MicroRNA 1183	RNA Gene	15	GC07P021510	2.996562	https://www.genecards.org/cgi-bin/carddisp.pl?gene=MIR1183
CDH26	Cadherin 26	Protein Coding	36	GC20P059958	2.993687	https://www.genecards.org/cgi-bin/carddisp.pl?gene=CDH26
LGALS7	Galectin 7	Protein Coding	38	GC19M038770	2.990034	https://www.genecards.org/cgi-bin/carddisp.pl?gene=LGALS7
HIF3A	Hypoxia Inducible Factor 3 Subunit Alpha	Protein Coding	40	GC19P046297	2.98899	https://www.genecards.org/cgi-bin/carddisp.pl?gene=HIF3A
CPLX3	Complexin 3	Protein Coding	36	GC15P074826	2.984288	https://www.genecards.org/cgi-bin/carddisp.pl?gene=CPLX3
HOXA7	Homeobox A7	Protein Coding	41	GC07M027153	2.982553	https://www.genecards.org/cgi-bin/carddisp.pl?gene=HOXA7
PDE1C	Phosphodiesterase 1C	Protein Coding	46	GC07M031616	2.979583	https://www.genecards.org/cgi-bin/carddisp.pl?gene=PDE1C

AP1M2	Adaptor Related Protein Complex 1 Subunit Mu 2	Protein Coding	41	GC19M010572	2.975761	https://www.genecards.org/cgi-bin/carddisp.pl?gene=AP1M2
FOXC2-AS1	FOXC2 Antisense RNA 1	RNA Gene	15	GC16M086566	2.975297	https://www.genecards.org/cgi-bin/carddisp.pl?gene=FOXC2-AS1
TMEM97	Transmembrane Protein 97	Protein Coding	36	GC17P028319	2.974014	https://www.genecards.org/cgi-bin/carddisp.pl?gene=TMEM97
EPHA7	EPH Receptor A7	Protein Coding	49	GC06M093240	2.972818	https://www.genecards.org/cgi-bin/carddisp.pl?gene=EPHA7
NRDC	Nardilysin Convertase	Protein Coding	31	GC01M051790	2.965019	https://www.genecards.org/cgi-bin/carddisp.pl?gene=NRDC
INTS11	Integrator Complex Subunit 11	Protein Coding	30	GC01M002704	2.958541	https://www.genecards.org/cgi-bin/carddisp.pl?gene=INTS11
ZNF217	Zinc Finger Protein 217	Protein Coding	39	GC20M053567	2.958487	https://www.genecards.org/cgi-bin/carddisp.pl?gene=ZNF217
COX6B2	Cytochrome C Oxidase Subunit 6B2	Protein Coding	32	GC19M055350	2.953898	https://www.genecards.org/cgi-bin/carddisp.pl?gene=COX6B2
EEF2K	Eukaryotic Elongation Factor 2 Kinase	Protein Coding	47	GC16P022217	2.949379	https://www.genecards.org/cgi-bin/carddisp.pl?gene=EEF2K
SVEP1	Sushi, Von Willebrand Factor Type A, EGF And Pentraxin Domain Containing 1	Protein Coding	36	GC09M110365	2.948899	https://www.genecards.org/cgi-bin/carddisp.pl?gene=SVEP1
CNOT4	CCR4-NOT Transcription Complex Subunit 4	Protein Coding	41	GC07M135361	2.946622	https://www.genecards.org/cgi-bin/carddisp.pl?gene=CNOT4
QSOX1	Quiescin Sulfhydryl Oxidase 1	Protein Coding	38	GC01P180154	2.940781	https://www.genecards.org/cgi-bin/carddisp.pl?gene=QSOX1
SYNPO2	Synaptopodin 2	Protein Coding	36	GC04P118850	2.930421	https://www.genecards.org/cgi-bin/carddisp.pl?gene=SYNPO2
H2BC11	H2B Clustered Histone 11	Protein Coding	29	GC06M046548	2.928202	https://www.genecards.org/cgi-bin/carddisp.pl?gene=H2BC11
ENTPD2	Ectonucleoside Triphosphate Diphosphohydrolase 2	Protein Coding	40	GC09M137048	2.925421	https://www.genecards.org/cgi-bin/carddisp.pl?gene=ENTPD2
LINC00472	Long Intergenic Non-Protein Coding RNA 472	RNA Gene	22	GC06M071344	2.925195	https://www.genecards.org/cgi-bin/carddisp.pl?gene=LINC00472
MSL2	MSL Complex Subunit 2	Protein Coding	34	GC03M136149	2.922533	https://www.genecards.org/cgi-bin/carddisp.pl?gene=MSL2
ENSG00000253111		RNA Gene	7	GC08P125466	2.917216	https://www.genecards.org/cgi-bin/carddisp.pl?gene=ENSG00000253111

TPD52L1	TPD52 Like 1	Protein Coding	40	GC06P125119	2.912905	https://www.genecards.org/cgi-bin/carddisp.pl?gene=TPD52L1
ODR4	Odr-4 GPCR Localization Factor Homolog	Protein Coding	25	GC01P186376	2.910861	https://www.genecards.org/cgi-bin/carddisp.pl?gene=ODR4
RAB32	RAB32, Member RAS Oncogene Family	Protein Coding	39	GC06P146543	2.908951	https://www.genecards.org/cgi-bin/carddisp.pl?gene=RAB32
DTD1	D-Aminoacyl-TRNA Deacylase 1	Protein Coding	36	GC20P018587	2.904941	https://www.genecards.org/cgi-bin/carddisp.pl?gene=DTD1
SMIM20	Small Integral Membrane Protein 20	Protein Coding	29	GC04P025865	2.903644	https://www.genecards.org/cgi-bin/carddisp.pl?gene=SMIM20
PARP10	Poly(ADP-Ribose) Polymerase Family Member 10	Protein Coding	38	GC08M143977	2.898402	https://www.genecards.org/cgi-bin/carddisp.pl?gene=PARP10
SNAPIN	SNAP Associated Protein	Protein Coding	38	GC01P153660	2.894905	https://www.genecards.org/cgi-bin/carddisp.pl?gene=SNAPIN
TRPC4AP	Transient Receptor Potential Cation Channel Subfamily C Member 4 Associated Protein	Protein Coding	37	GC20M035002	2.888846	https://www.genecards.org/cgi-bin/carddisp.pl?gene=TRPC4AP
SEC23IP	SEC23 Interacting Protein	Protein Coding	40	GC10P119892	2.888827	https://www.genecards.org/cgi-bin/carddisp.pl?gene=SEC23IP
SCGB1D2	Secretoglobin Family 1D Member 2	Protein Coding	33	GC11P062260	2.880019	https://www.genecards.org/cgi-bin/carddisp.pl?gene=SCGB1D2
FAM186B	Family With Sequence Similarity 186 Member B	Protein Coding	33	GC12M049582	2.879512	https://www.genecards.org/cgi-bin/carddisp.pl?gene=FAM186B
LINC01194	Long Intergenic Non-Protein Coding RNA 1194	RNA Gene	16	GC05P012578	2.878832	https://www.genecards.org/cgi-bin/carddisp.pl?gene=LINC01194
BECN2	Beclin 2	Protein Coding	23	GC01P241957	2.877337	https://www.genecards.org/cgi-bin/carddisp.pl?gene=BECN2
ANP32A	Acidic Nuclear Phosphoprotein 32 Family Member A	Protein Coding	43	GC15M068778	2.875695	https://www.genecards.org/cgi-bin/carddisp.pl?gene=ANP32A
WDR59	WD Repeat Domain 59	Protein Coding	37	GC16M074871	2.874957	https://www.genecards.org/cgi-bin/carddisp.pl?gene=WDR59

LELP1	Late Cornified Envelope Like Proline Rich 1	Protein Coding	32	GC01P153175	2.873115	https://www.genecards.org/cgi-bin/carddisp.pl?gene=LELP1
PIP5K1A	Phosphatidylinositol-4-Phosphate 5-Kinase Type 1 Alpha	Protein Coding	45	GC01P151198	2.86884	https://www.genecards.org/cgi-bin/carddisp.pl?gene=PIP5K1A
NLN	Neurolysin	Protein Coding	41	GC05P065722	2.859017	https://www.genecards.org/cgi-bin/carddisp.pl?gene=NLN
CPSF1	Cleavage And Polyadenylation Specific Factor 1	Protein Coding	40	GC08M144393	2.858375	https://www.genecards.org/cgi-bin/carddisp.pl?gene=CPSF1
CHAF1A	Chromatin Assembly Factor 1 Subunit A	Protein Coding	38	GC19P004402	2.856692	https://www.genecards.org/cgi-bin/carddisp.pl?gene=CHAF1A
VKORC1L1	Vitamin K Epoxide Reductase Complex Subunit 1 Like 1	Protein Coding	38	GC07P066213	2.855765	https://www.genecards.org/cgi-bin/carddisp.pl?gene=VKORC1L1
ZNFX1	Zinc Finger NFX1-Type Containing 1	Protein Coding	34	GC20M049238	2.855133	https://www.genecards.org/cgi-bin/carddisp.pl?gene=ZNFX1
BFAR	Bifunctional Apoptosis Regulator	Protein Coding	38	GC16P014637	2.848508	https://www.genecards.org/cgi-bin/carddisp.pl?gene=BFAR
AZIN2	Antizyme Inhibitor 2	Protein Coding	36	GC01P033081	2.847816	https://www.genecards.org/cgi-bin/carddisp.pl?gene=AZIN2
MAFF	MAF BZIP Transcription Factor F	Protein Coding	41	GC22P038200	2.845722	https://www.genecards.org/cgi-bin/carddisp.pl?gene=MAFF
GLT8D2	Glycosyltransferase 8 Domain Containing 2	Protein Coding	36	GC12M103988	2.843195	https://www.genecards.org/cgi-bin/carddisp.pl?gene=GLT8D2
DHX15	DEAH-Box Helicase 15	Protein Coding	38	GC04M024519	2.840756	https://www.genecards.org/cgi-bin/carddisp.pl?gene=DHX15
BCL2L15	BCL2 Like 15	Protein Coding	33	GC01M113876	2.840091	https://www.genecards.org/cgi-bin/carddisp.pl?gene=BCL2L15
ACTR5	Actin Related Protein 5	Protein Coding	35	GC20P038748	2.83811	https://www.genecards.org/cgi-bin/carddisp.pl?gene=ACTR5
SEMA7A	Semaphorin 7A (John Milton Hagen Blood Group)	Protein Coding	43	GC15M074409	2.837412	https://www.genecards.org/cgi-bin/carddisp.pl?gene=SEMA7A
B4GALT6	Beta-1,4-Galactosyltransferase 6	Protein Coding	41	GC18M031622	2.836326	https://www.genecards.org/cgi-bin/carddisp.pl?gene=B4GALT6
DGKH	Diacylglycerol Kinase Eta	Protein Coding	42	GC13P042040	2.833042	https://www.genecards.org/cgi-bin/carddisp.pl?gene=DGKH
IFN1@	Interferon, Type 1, Cluster	Gene Cluster	4	GC09U990039	2.83261	https://www.genecards.org/cgi-bin/carddisp.pl?gene=IFN1%40
RNF13	Ring Finger Protein 13	Protein Coding	42	GC03P149812	2.828979	https://www.genecards.org/cgi-bin/carddisp.pl?gene=RNF13

ULK2	Unc-51 Like Autophagy Activating Kinase 2	Protein Coding	41	GC17M021706	2.828438	https://www.genecards.org/cgi-bin/carddisp.pl?gene=ULK2
PIK3C2B	Phosphatidylinositol-4-Phosphate 3-Kinase Catalytic Subunit Type 2 Beta	Protein Coding	48	GC01M204422	2.825679	https://www.genecards.org/cgi-bin/carddisp.pl?gene=PIK3C2B
MLXIP	MLX Interacting Protein	Protein Coding	33	GC12P122078	2.82372	https://www.genecards.org/cgi-bin/carddisp.pl?gene=MLXIP
RUSC2	RUN And SH3 Domain Containing 2	Protein Coding	37	GC09P035490	2.823524	https://www.genecards.org/cgi-bin/carddisp.pl?gene=RUSC2
DDX60	DExD/H-Box Helicase 60	Protein Coding	35	GC04M168216	2.821466	https://www.genecards.org/cgi-bin/carddisp.pl?gene=DDX60
MSL1	MSL Complex Subunit 1	Protein Coding	34	GC17P040122	2.816136	https://www.genecards.org/cgi-bin/carddisp.pl?gene=MSL1
TSNAX-DISC1	TSNAX-DISC1 Readthrough (NMD Candidate)	RNA Gene	19	GC01P231528	2.8153	https://www.genecards.org/cgi-bin/carddisp.pl?gene=TSNAX-DISC1
CAPSL	Calcyphosine Like	Protein Coding	34	GC05M035906	2.814205	https://www.genecards.org/cgi-bin/carddisp.pl?gene=CAPSL
SUPT7L	SPT7 Like, STAGA Complex Subunit Gamma	Protein Coding	32	GC02M027711	2.812148	https://www.genecards.org/cgi-bin/carddisp.pl?gene=SUPT7L
CEP170	Centrosomal Protein 170	Protein Coding	40	GC01M243124	2.810482	https://www.genecards.org/cgi-bin/carddisp.pl?gene=CEP170
PLEKHA7	Pleckstrin Homology Domain Containing A7	Protein Coding	38	GC11M016778	2.809566	https://www.genecards.org/cgi-bin/carddisp.pl?gene=PLEKHA7
CLCA2	Chloride Channel Accessory 2	Protein Coding	41	GC01P086424	2.805415	https://www.genecards.org/cgi-bin/carddisp.pl?gene=CLCA2
TM4SF5	Transmembrane 4 L Six Family Member 5	Protein Coding	34	GC17P004771	2.804374	https://www.genecards.org/cgi-bin/carddisp.pl?gene=TM4SF5
H3C4	H3 Clustered Histone 4	Protein Coding	31	GC06M047216	2.797997	https://www.genecards.org/cgi-bin/carddisp.pl?gene=H3C4
CAAP1	Caspase Activity And Apoptosis Inhibitor 1	Protein Coding	31	GC09M026840	2.794522	https://www.genecards.org/cgi-bin/carddisp.pl?gene=CAAP1
GCAT	Glycine C-Acetyltransferase	Protein Coding	43	GC22P037807	2.793952	https://www.genecards.org/cgi-bin/carddisp.pl?gene=GCAT
COPZ1	COPI Coat Complex Subunit Zeta 1	Protein Coding	40	GC12P054301	2.788088	https://www.genecards.org/cgi-bin/carddisp.pl?gene=COPZ1

UQCRHL	Ubiquinol-Cytochrome C Reductase Hinge Protein Like	Protein Coding	23	GC01M015808	2.786937	https://www.genecards.org/cgi-bin/carddisp.pl?gene=UQCRHL
DGAT2L6	Diacylglycerol O-Acyltransferase 2 Like 6	Protein Coding	30	GC0XP070177	2.786317	https://www.genecards.org/cgi-bin/carddisp.pl?gene=DGAT2L6
H3C15	H3 Clustered Histone 15	Protein Coding	27	GC01P149970	2.786226	https://www.genecards.org/cgi-bin/carddisp.pl?gene=H3C15
SDF2L1	Stromal Cell Derived Factor 2 Like 1	Protein Coding	35	GC22P026487	2.786216	https://www.genecards.org/cgi-bin/carddisp.pl?gene=SDF2L1
FHL5	Four And A Half LIM Domains 5	Protein Coding	38	GC06P096562	2.786175	https://www.genecards.org/cgi-bin/carddisp.pl?gene=FHL5
TCF7L1	Transcription Factor 7 Like 1	Protein Coding	42	GC02P085133	2.783878	https://www.genecards.org/cgi-bin/carddisp.pl?gene=TCF7L1
PLSCR1	Phospholipid Scramblase 1	Protein Coding	44	GC03M146515	2.782262	https://www.genecards.org/cgi-bin/carddisp.pl?gene=PLSCR1
EBNA1BP2	EBNA1 Binding Protein 2	Protein Coding	38	GC01M043165	2.773913	https://www.genecards.org/cgi-bin/carddisp.pl?gene=EBNA1BP2
RUBCNL	Rubicon Like Autophagy Enhancer	Protein Coding	26	GC13M046343	2.773623	https://www.genecards.org/cgi-bin/carddisp.pl?gene=RUBCNL
H3C6	H3 Clustered Histone 6	Protein Coding	28	GC06P055588	2.769617	https://www.genecards.org/cgi-bin/carddisp.pl?gene=H3C6
HMGB2	High Mobility Group Box 2	Protein Coding	41	GC04M173331	2.767611	https://www.genecards.org/cgi-bin/carddisp.pl?gene=HMGB2
PPM1A	Protein Phosphatase, Mg2+/Mn2+ Dependent 1A	Protein Coding	45	GC14P060245	2.765466	https://www.genecards.org/cgi-bin/carddisp.pl?gene=PPM1A
ELF3	E74 Like ETS Transcription Factor 3	Protein Coding	40	GC01P202007	2.764115	https://www.genecards.org/cgi-bin/carddisp.pl?gene=ELF3
FZR1	Fizzy And Cell Division Cycle 20 Related 1	Protein Coding	43	GC19P003506	2.760143	https://www.genecards.org/cgi-bin/carddisp.pl?gene=FZR1
ZNF101	Zinc Finger Protein 101	Protein Coding	34	GC19P026665	2.759101	https://www.genecards.org/cgi-bin/carddisp.pl?gene=ZNF101
SPCS2	Signal Peptidase Complex Subunit 2	Protein Coding	35	GC11P076258	2.759062	https://www.genecards.org/cgi-bin/carddisp.pl?gene=SPCS2
RESF1	Retroelement Silencing Factor 1	Protein Coding	25	GC12P032003	2.757162	https://www.genecards.org/cgi-bin/carddisp.pl?gene=RESF1
MAFA	MAF BZIP Transcription Factor A	Protein Coding	42	GC08M143419	2.757057	https://www.genecards.org/cgi-bin/carddisp.pl?gene=MAFA
STRA8	Stimulated By Retinoic Acid 8	Protein Coding	36	GC07P135231	2.756898	https://www.genecards.org/cgi-bin/carddisp.pl?gene=STRA8
H3C11	H3 Clustered Histone 11	Protein Coding	28	GC06M047214	2.754311	https://www.genecards.org/cgi-bin/carddisp.pl?gene=H3C11

SURF4	Surfeit 4	Protein Coding	39	GC09M133361	2.752711	https://www.genecards.org/cgi-bin/carddisp.pl?gene=SURF4
RALBP1	RalA Binding Protein 1	Protein Coding	45	GC18P009465	2.752016	https://www.genecards.org/cgi-bin/carddisp.pl?gene=RALBP1
RAB25	RAB25, Member RAS Oncogene Family	Protein Coding	40	GC01P156061	2.751238	https://www.genecards.org/cgi-bin/carddisp.pl?gene=RAB25
TIMD4	T Cell Immunoglobulin And Mucin Domain Containing 4	Protein Coding	37	GC05M156919	2.750753	https://www.genecards.org/cgi-bin/carddisp.pl?gene=TIMD4
TMEM109	Transmembrane Protein 109	Protein Coding	32	GC11P060914	2.74667	https://www.genecards.org/cgi-bin/carddisp.pl?gene=TMEM109
ZC3HAV1	Zinc Finger CCCH-Type Containing, Antiviral 1	Protein Coding	38	GC07M139044	2.746261	https://www.genecards.org/cgi-bin/carddisp.pl?gene=ZC3HAV1
AHRR	Aryl-Hydrocarbon Receptor Repressor	Protein Coding	36	GC05P000321	2.744704	https://www.genecards.org/cgi-bin/carddisp.pl?gene=AHRR
ZBTB2	Zinc Finger And BTB Domain Containing 2	Protein Coding	34	GC06M151364	2.742771	https://www.genecards.org/cgi-bin/carddisp.pl?gene=ZBTB2
GMFB	Glia Maturation Factor Beta	Protein Coding	41	GC14M054474	2.742506	https://www.genecards.org/cgi-bin/carddisp.pl?gene=GMFB
PCDHB4	Protocadherin Beta 4	Protein Coding	37	GC05P143796	2.741513	https://www.genecards.org/cgi-bin/carddisp.pl?gene=PCDHB4
VAMP3	Vesicle Associated Membrane Protein 3	Protein Coding	43	GC01P007765	2.735823	https://www.genecards.org/cgi-bin/carddisp.pl?gene=VAMP3
PARP2	Poly(ADP-Ribose) Polymerase 2	Protein Coding	47	GC14P020343	2.731313	https://www.genecards.org/cgi-bin/carddisp.pl?gene=PARP2
ZNF267	Zinc Finger Protein 267	Protein Coding	36	GC16P032629	2.73066	https://www.genecards.org/cgi-bin/carddisp.pl?gene=ZNF267
KLK13	Kallikrein Related Peptidase 13	Protein Coding	40	GC19M051056	2.728004	https://www.genecards.org/cgi-bin/carddisp.pl?gene=KLK13
GLCE	Glucuronic Acid Epimerase	Protein Coding	40	GC15P077108	2.72756	https://www.genecards.org/cgi-bin/carddisp.pl?gene=GLCE
PGAP6	Post-Glycosylphosphatidylinositol Attachment To Proteins 6	Protein Coding	26	GC16M000370	2.723347	https://www.genecards.org/cgi-bin/carddisp.pl?gene=PGAP6
RTCB	RNA 2',3'-Cyclic Phosphate And 5'-OH Ligase	Protein Coding	36	GC22M032387	2.72297	https://www.genecards.org/cgi-bin/carddisp.pl?gene=RTCB

KTN1	Kinectin 1	Protein Coding	40	GC14P055559	2.722031	https://www.genecards.org/cgi-bin/carddisp.pl?gene=KTN1
SLC45A3	Solute Carrier Family 45 Member 3	Protein Coding	40	GC01M205626	2.7165	https://www.genecards.org/cgi-bin/carddisp.pl?gene=SLC45A3
APOBR	Apolipoprotein B Receptor	Protein Coding	33	GC16P028494	2.710127	https://www.genecards.org/cgi-bin/carddisp.pl?gene=APOBR
IPO7	Importin 7	Protein Coding	38	GC11P009384	2.707721	https://www.genecards.org/cgi-bin/carddisp.pl?gene=IPO7
EPSTI1	Epithelial Stromal Interaction 1	Protein Coding	36	GC13M042886	2.702394	https://www.genecards.org/cgi-bin/carddisp.pl?gene=EPSTI1
NECTIN4	Nectin Cell Adhesion Molecule 4	Protein Coding	38	GC01M161071	2.696864	https://www.genecards.org/cgi-bin/carddisp.pl?gene=NECTIN4
RAC3	Rac Family Small GTPase 3	Protein Coding	46	GC17P082031	2.693315	https://www.genecards.org/cgi-bin/carddisp.pl?gene=RAC3
H3C8	H3 Clustered Histone 8	Protein Coding	29	GC06M047218	2.689676	https://www.genecards.org/cgi-bin/carddisp.pl?gene=H3C8
SLC2A6	Solute Carrier Family 2 Member 6	Protein Coding	42	GC09M133471	2.687112	https://www.genecards.org/cgi-bin/carddisp.pl?gene=SLC2A6
ANGPTL2	Angiopoietin Like 2	Protein Coding	36	GC09M127087	2.68692	https://www.genecards.org/cgi-bin/carddisp.pl?gene=ANGPTL2
MIR646HG	MIR646 Host Gene	RNA Gene	15	GC20P060088	2.681141	https://www.genecards.org/cgi-bin/carddisp.pl?gene=MIR646HG
PRR5-ARHGAP8	PRR5-ARHGAP8 Readthrough	Protein Coding	27	GC22P044702	2.681102	https://www.genecards.org/cgi-bin/carddisp.pl?gene=PRR5-ARHGAP8
MED14	Mediator Complex Subunit 14	Protein Coding	40	GC0XM040648	2.68012	https://www.genecards.org/cgi-bin/carddisp.pl?gene=MED14
RAMP3	Receptor Activity Modifying Protein 3	Protein Coding	41	GC07P045163	2.679981	https://www.genecards.org/cgi-bin/carddisp.pl?gene=RAMP3
SMC6	Structural Maintenance Of Chromosomes 6	Protein Coding	35	GC02M017663	2.67877	https://www.genecards.org/cgi-bin/carddisp.pl?gene=SMC6
HCAR2	Hydroxycarboxylic Acid Receptor 2	Protein Coding	38	GC12M122701	2.676188	https://www.genecards.org/cgi-bin/carddisp.pl?gene=HCAR2
ZNF574	Zinc Finger Protein 574	Protein Coding	33	GC19P042068	2.670589	https://www.genecards.org/cgi-bin/carddisp.pl?gene=ZNF574
COP1	COP1 E3 Ubiquitin Ligase	Protein Coding	34	GC01M175944	2.670171	https://www.genecards.org/cgi-bin/carddisp.pl?gene=COP1
TLE3	TLE Family Member 3, Transcriptional Corepressor	Protein Coding	44	GC15M070047	2.668141	https://www.genecards.org/cgi-bin/carddisp.pl?gene=TLE3
HDAC11	Histone Deacetylase 11	Protein Coding	44	GC03P013478	2.667408	https://www.genecards.org/cgi-bin/carddisp.pl?gene=HDAC11

BCAR4	Breast Cancer Anti-Estrogen Resistance 4	RNA Gene	17	GC16M011819	2.667309	https://www.genecards.org/cgi-bin/carddisp.pl?gene=BCAR4
SART3	Spliceosome Associated Factor 3, U4/U6 Recycling Protein	Protein Coding	37	GC12M108522	2.6658	https://www.genecards.org/cgi-bin/carddisp.pl?gene=SART3
DDO	D-Aspartate Oxidase	Protein Coding	40	GC06M110391	2.664339	https://www.genecards.org/cgi-bin/carddisp.pl?gene=DDO
MUCL1	Mucin Like 1	Protein Coding	32	GC12P054830	2.664232	https://www.genecards.org/cgi-bin/carddisp.pl?gene=MUCL1
PLCH1	Phospholipase C Eta 1	Protein Coding	39	GC03M155381	2.663233	https://www.genecards.org/cgi-bin/carddisp.pl?gene=PLCH1
LOC110596866	CYP7A1 5' Regulatory Region	Biological Region	1	GC08P058498	2.656094	https://www.genecards.org/cgi-bin/carddisp.pl?gene=LOC110596866
PGA3	Pepsinogen A3	Protein Coding	34	GC11P061203	2.651723	https://www.genecards.org/cgi-bin/carddisp.pl?gene=PGA3
EIF5B	Eukaryotic Translation Initiation Factor 5B	Protein Coding	38	GC02P099320	2.645843	https://www.genecards.org/cgi-bin/carddisp.pl?gene=EIF5B
BRMS1L	BRMS1 Like Transcriptional Repressor	Protein Coding	35	GC14P035840	2.643856	https://www.genecards.org/cgi-bin/carddisp.pl?gene=BRMS1L
GPR107	G Protein-Coupled Receptor 107	Protein Coding	36	GC09P130053	2.64118	https://www.genecards.org/cgi-bin/carddisp.pl?gene=GPR107
POLR1G	RNA Polymerase I Subunit G	Protein Coding	27	GC19P045409	2.638262	https://www.genecards.org/cgi-bin/carddisp.pl?gene=POLR1G
PTPN9	Protein Tyrosine Phosphatase Non-Receptor Type 9	Protein Coding	41	GC15M075463	2.637581	https://www.genecards.org/cgi-bin/carddisp.pl?gene=PTPN9
TUBGCP4	Tubulin Gamma Complex Associated Protein 4	Protein Coding	39	GC15P043369	2.626695	https://www.genecards.org/cgi-bin/carddisp.pl?gene=TUBGCP4
ENSG00000231487		RNA Gene	6	GC11M001683	2.623965	https://www.genecards.org/cgi-bin/carddisp.pl?gene=ENSG00000231487
TRMT2A	TRNA Methyltransferase 2 Homolog A	Protein Coding	39	GC22M020099	2.621841	https://www.genecards.org/cgi-bin/carddisp.pl?gene=TRMT2A
ZDHHC18	Zinc Finger DHHC-Type Palmitoyltransferase 18	Protein Coding	34	GC01P026911	2.621691	https://www.genecards.org/cgi-bin/carddisp.pl?gene=ZDHHC18
C16orf95	Chromosome 16 Open Reading Frame 95	Protein Coding	26	GC16M087119	2.620677	https://www.genecards.org/cgi-bin/carddisp.pl?gene=C16orf95

H2BC18	H2B Clustered Histone 18	Protein Coding	27	GC01M150113	2.620003	https://www.genecards.org/cgi-bin/carddisp.pl?gene=H2BC18
NCAPG2	Non-SMC Condensin II Complex Subunit G2	Protein Coding	39	GC07M158631	2.618309	https://www.genecards.org/cgi-bin/carddisp.pl?gene=NCAPG2
CCNB2	Cyclin B2	Protein Coding	45	GC15P059105	2.618125	https://www.genecards.org/cgi-bin/carddisp.pl?gene=CCNB2
PLEKHM2	Pleckstrin Homology And RUN Domain Containing M2	Protein Coding	36	GC01P015694	2.61637	https://www.genecards.org/cgi-bin/carddisp.pl?gene=PLEKHM2
ZNF318	Zinc Finger Protein 318	Protein Coding	37	GC06M047133	2.613955	https://www.genecards.org/cgi-bin/carddisp.pl?gene=ZNF318
FOXL1	Forkhead Box L1	Protein Coding	38	GC16P086576	2.610366	https://www.genecards.org/cgi-bin/carddisp.pl?gene=FOXL1
MIR6090	MicroRNA 6090	RNA Gene	10	GC11P128522	2.609474	https://www.genecards.org/cgi-bin/carddisp.pl?gene=MIR6090
SLC2A8	Solute Carrier Family 2 Member 8	Protein Coding	40	GC09P127398	2.607051	https://www.genecards.org/cgi-bin/carddisp.pl?gene=SLC2A8
CYB5RL	Cytochrome B5 Reductase Like	Protein Coding	30	GC01M054172	2.604855	https://www.genecards.org/cgi-bin/carddisp.pl?gene=CYB5RL
S100A14	S100 Calcium Binding Protein A14	Protein Coding	38	GC01M153614	2.60359	https://www.genecards.org/cgi-bin/carddisp.pl?gene=S100A14
IL36B	Interleukin 36 Beta	Protein Coding	35	GC02M113022	2.602809	https://www.genecards.org/cgi-bin/carddisp.pl?gene=IL36B
ETS1-AS1	ETS1 Antisense RNA 1	RNA Gene	11	GC11P128527	2.595277	https://www.genecards.org/cgi-bin/carddisp.pl?gene=ETS1-AS1
PPP4C	Protein Phosphatase 4 Catalytic Subunit	Protein Coding	46	GC16P032401	2.591996	https://www.genecards.org/cgi-bin/carddisp.pl?gene=PPP4C
RALGDS	Ral Guanine Nucleotide Dissociation Stimulator	Protein Coding	43	GC09M133097	2.586136	https://www.genecards.org/cgi-bin/carddisp.pl?gene=RALGDS
BTN2A2	Butyrophilin Subfamily 2 Member A2	Protein Coding	37	GC06P026382	2.585892	https://www.genecards.org/cgi-bin/carddisp.pl?gene=BTN2A2
DGKI	Diacylglycerol Kinase Iota	Protein Coding	43	GC07M137381	2.582622	https://www.genecards.org/cgi-bin/carddisp.pl?gene=DGKI
INPP4B	Inositol Polyphosphate-4-Phosphatase Type II B	Protein Coding	39	GC04M142023	2.581787	https://www.genecards.org/cgi-bin/carddisp.pl?gene=INPP4B
TWF1	Twinfilin Actin Binding Protein 1	Protein Coding	39	GC12M043793	2.580847	https://www.genecards.org/cgi-bin/carddisp.pl?gene=TWF1
TASP1	Taspase 1	Protein Coding	38	GC20M013105	2.579393	https://www.genecards.org/cgi-bin/carddisp.pl?gene=TASP1
DGKZ	Diacylglycerol Kinase Zeta	Protein Coding	45	GC11P046332	2.577645	https://www.genecards.org/cgi-bin/carddisp.pl?gene=DGKZ

OSBPL8	Oxysterol Binding Protein Like 8	Protein Coding	38	GC12M076354	2.577201	https://www.genecards.org/cgi-bin/carddisp.pl?gene=OSBPL8
A2MP1	Alpha-2-Macroglobulin Pseudogene 1	Pseudogene	13	GC12M009228	2.573188	https://www.genecards.org/cgi-bin/carddisp.pl?gene=A2MP1
CYB5R1	Cytochrome B5 Reductase 1	Protein Coding	42	GC01M202964	2.569713	https://www.genecards.org/cgi-bin/carddisp.pl?gene=CYB5R1
LPAR2	Lysophosphatidic Acid Receptor 2	Protein Coding	45	GC19M019624	2.568109	https://www.genecards.org/cgi-bin/carddisp.pl?gene=LPAR2
NMB	Neuromedin B	Protein Coding	42	GC15M084655	2.568079	https://www.genecards.org/cgi-bin/carddisp.pl?gene=NMB
HIC2	HIC ZBTB Transcriptional Repressor 2	Protein Coding	36	GC22P026481	2.567751	https://www.genecards.org/cgi-bin/carddisp.pl?gene=HIC2
LYPLAL1	Lysophospholipase Like 1	Protein Coding	37	GC01P219173	2.566895	https://www.genecards.org/cgi-bin/carddisp.pl?gene=LYPLAL1
ZBED4	Zinc Finger BED-Type Containing 4	Protein Coding	34	GC22P049853	2.565559	https://www.genecards.org/cgi-bin/carddisp.pl?gene=ZBED4
PPP1R14C	Protein Phosphatase 1 Regulatory Inhibitor Subunit 14C	Protein Coding	38	GC06P150143	2.564817	https://www.genecards.org/cgi-bin/carddisp.pl?gene=PPP1R14C
PLSCR3	Phospholipid Scramblase 3	Protein Coding	38	GC17M007389	2.563215	https://www.genecards.org/cgi-bin/carddisp.pl?gene=PLSCR3
EDEM2	ER Degradation Enhancing Alpha-Mannosidase Like Protein 2	Protein Coding	41	GC20M035115	2.561573	https://www.genecards.org/cgi-bin/carddisp.pl?gene=EDEM2
NEURL4	Neuralized E3 Ubiquitin Protein Ligase 4	Protein Coding	32	GC17M007315	2.558761	https://www.genecards.org/cgi-bin/carddisp.pl?gene=NEURL4
LGR6	Leucine Rich Repeat Containing G Protein-Coupled Receptor 6	Protein Coding	43	GC01P202193	2.555842	https://www.genecards.org/cgi-bin/carddisp.pl?gene=LGR6
MPP3	Membrane Palmitoylated Protein 3	Protein Coding	39	GC17M043800	2.554013	https://www.genecards.org/cgi-bin/carddisp.pl?gene=MPP3
CCSER1	Coiled-Coil Serine Rich Protein 1	Protein Coding	31	GC04P090127	2.550223	https://www.genecards.org/cgi-bin/carddisp.pl?gene=CCSER1
CYP4Z1	Cytochrome P450 Family 4 Subfamily Z Member 1	Protein Coding	36	GC01P047067	2.545819	https://www.genecards.org/cgi-bin/carddisp.pl?gene=CYP4Z1

ERCC6L	ERCC Excision Repair 6 Like, Spindle Assembly Checkpoint Helicase	Protein Coding	37	GC0XM072204	2.543537	https://www.genecards.org/cgi-bin/carddisp.pl?gene=ERCC6L
H3C13	H3 Clustered Histone 13	Protein Coding	26	GC01M150118	2.538886	https://www.genecards.org/cgi-bin/carddisp.pl?gene=H3C13
SH3BGRL2	SH3 Domain Binding Glutamate Rich Protein Like 2	Protein Coding	36	GC06P079543	2.538296	https://www.genecards.org/cgi-bin/carddisp.pl?gene=SH3BGRL2
H2BC13	H2B Clustered Histone 13	Protein Coding	27	GC06M046624	2.536497	https://www.genecards.org/cgi-bin/carddisp.pl?gene=H2BC13
CCDC71L	Coiled-Coil Domain Containing 71 Like	Protein Coding	28	GC07M106656	2.532596	https://www.genecards.org/cgi-bin/carddisp.pl?gene=CCDC71L
CDCP1	CUB Domain Containing Protein 1	Protein Coding	39	GC03M045082	2.531418	https://www.genecards.org/cgi-bin/carddisp.pl?gene=CDCP1
WDR82	WD Repeat Domain 82	Protein Coding	37	GC03M052254	2.530725	https://www.genecards.org/cgi-bin/carddisp.pl?gene=WDR82
ATG9A	Autophagy Related 9A	Protein Coding	40	GC02M219219	2.527238	https://www.genecards.org/cgi-bin/carddisp.pl?gene=ATG9A
CRACD	Capping Protein Inhibiting Regulator Of Actin Dynamics	Protein Coding	24	GC04P056050	2.525075	https://www.genecards.org/cgi-bin/carddisp.pl?gene=CRACD
MYO1F	Myosin IF	Protein Coding	38	GC19M008520	2.52281	https://www.genecards.org/cgi-bin/carddisp.pl?gene=MYO1F
ZNF44	Zinc Finger Protein 44	Protein Coding	37	GC19M012224	2.52252	https://www.genecards.org/cgi-bin/carddisp.pl?gene=ZNF44
FAIM	Fas Apoptotic Inhibitory Molecule	Protein Coding	40	GC03P138608	2.515892	https://www.genecards.org/cgi-bin/carddisp.pl?gene=FAIM
SUSD2	Sushi Domain Containing 2	Protein Coding	36	GC22P024181	2.513223	https://www.genecards.org/cgi-bin/carddisp.pl?gene=SUSD2
JDP2	Jun Dimerization Protein 2	Protein Coding	41	GC14P075427	2.511678	https://www.genecards.org/cgi-bin/carddisp.pl?gene=JDP2
ME2	Malic Enzyme 2	Protein Coding	45	GC18P050879	2.511422	https://www.genecards.org/cgi-bin/carddisp.pl?gene=ME2
CTU1	Cytosolic Thiouridylase Subunit 1	Protein Coding	35	GC19M051097	2.507203	https://www.genecards.org/cgi-bin/carddisp.pl?gene=CTU1
STOML2	Stomatin Like 2	Protein Coding	39	GC09M035099	2.503873	https://www.genecards.org/cgi-bin/carddisp.pl?gene=STOML2
SNRPB2	Small Nuclear Ribonucleoprotein Polypeptide B2	Protein Coding	38	GC20P016730	2.503085	https://www.genecards.org/cgi-bin/carddisp.pl?gene=SNRPB2
RNU6-961P	RNA, U6 Small Nuclear 961, Pseudogene	Pseudogene	8	GC02P020175	2.502647	https://www.genecards.org/cgi-bin/carddisp.pl?gene=RNU6-961P

SUMO2	Small Ubiquitin Like Modifier 2	Protein Coding	40	GC17M075165	2.498737	https://www.genecards.org/cgi-bin/carddisp.pl?gene=SUMO2
ZNF106	Zinc Finger Protein 106	Protein Coding	35	GC15M042412	2.497016	https://www.genecards.org/cgi-bin/carddisp.pl?gene=ZNF106
MIR7-3	MicroRNA 7-3	RNA Gene	17	GC19P004770	2.496814	https://www.genecards.org/cgi-bin/carddisp.pl?gene=MIR7-3
PRDM14	PR/SET Domain 14	Protein Coding	40	GC08M070063	2.496771	https://www.genecards.org/cgi-bin/carddisp.pl?gene=PRDM14
ARL14	ADP Ribosylation Factor Like GTPase 14	Protein Coding	31	GC03P160677	2.492559	https://www.genecards.org/cgi-bin/carddisp.pl?gene=ARL14
H2AC21	H2A Clustered Histone 21	Protein Coding	27	GC01M150139	2.485057	https://www.genecards.org/cgi-bin/carddisp.pl?gene=H2AC21
HCAR1	Hydroxycarboxylic Acid Receptor 1	Protein Coding	38	GC12M122726	2.481189	https://www.genecards.org/cgi-bin/carddisp.pl?gene=HCAR1
SUMO3	Small Ubiquitin Like Modifier 3	Protein Coding	43	GC21M044805	2.481158	https://www.genecards.org/cgi-bin/carddisp.pl?gene=SUMO3
ENSG00000231992		RNA Gene	8	GC01P095120	2.478649	https://www.genecards.org/cgi-bin/carddisp.pl?gene=ENSG00000231992
LSM1	LSM1 Homolog, MRNA Degradation Associated	Protein Coding	40	GC08M038163	2.477331	https://www.genecards.org/cgi-bin/carddisp.pl?gene=LSM1
SBNO1	Strawberry Notch Homolog 1	Protein Coding	36	GC12M123289	2.477088	https://www.genecards.org/cgi-bin/carddisp.pl?gene=SBNO1
ETNPPL	Ethanolamine-Phosphate Phospho-Lyase	Protein Coding	36	GC04M108742	2.476469	https://www.genecards.org/cgi-bin/carddisp.pl?gene=ETNPPL
RTKN2	Rhotekin 2	Protein Coding	36	GC10M062183	2.4759	https://www.genecards.org/cgi-bin/carddisp.pl?gene=RTKN2
LINC01684	Long Intergenic Non-Protein Coding RNA 1684	RNA Gene	9	GC21P024306	2.475893	https://www.genecards.org/cgi-bin/carddisp.pl?gene=LINC01684
INPP5A	Inositol Polyphosphate-5-Phosphatase A	Protein Coding	43	GC10P132537	2.4723	https://www.genecards.org/cgi-bin/carddisp.pl?gene=INPP5A
NR2E1	Nuclear Receptor Subfamily 2 Group E Member 1	Protein Coding	44	GC06P108166	2.468215	https://www.genecards.org/cgi-bin/carddisp.pl?gene=NR2E1
LINC01354	Long Intergenic Non-Protein Coding RNA 1354	RNA Gene	12	GC01M234528	2.466466	https://www.genecards.org/cgi-bin/carddisp.pl?gene=LINC01354

C1orf105	Chromosome 1 Open Reading Frame 105	Protein Coding	32	GC01P172419	2.466349	https://www.genecards.org/cgi-bin/carddisp.pl?gene=C1orf105
REPIN1	Replication Initiator 1	Protein Coding	34	GC07P150368	2.465403	https://www.genecards.org/cgi-bin/carddisp.pl?gene=REPIN1
REG1B	Regenerating Family Member 1 Beta	Protein Coding	39	GC02M079086	2.464712	https://www.genecards.org/cgi-bin/carddisp.pl?gene=REG1B
ARL2BPP6	ADP Ribosylation Factor Like GTPase 2 Binding Protein Pseudogene 6	Pseudogene	6	GC05P175290	2.462798	https://www.genecards.org/cgi-bin/carddisp.pl?gene=ARL2BPP6
GPSM1	G Protein Signaling Modulator 1	Protein Coding	38	GC09P136327	2.461058	https://www.genecards.org/cgi-bin/carddisp.pl?gene=GPSM1
FNDC3B	Fibronectin Type III Domain Containing 3B	Protein Coding	37	GC03P172039	2.46006	https://www.genecards.org/cgi-bin/carddisp.pl?gene=FNDC3B
FMNL1	Formin Like 1	Protein Coding	37	GC17P045222	2.459692	https://www.genecards.org/cgi-bin/carddisp.pl?gene=FMNL1
SGF29	SAGA Complex Associated Factor 29	Protein Coding	29	GC16P032174	2.459174	https://www.genecards.org/cgi-bin/carddisp.pl?gene=SGF29
GBX2	Gastrulation Brain Homeobox 2	Protein Coding	40	GC02M236165	2.458367	https://www.genecards.org/cgi-bin/carddisp.pl?gene=GBX2
EPB41L2	Erythrocyte Membrane Protein Band 4.1 Like 2	Protein Coding	40	GC06M130820	2.452523	https://www.genecards.org/cgi-bin/carddisp.pl?gene=EPB41L2
SCAMP3	Secretory Carrier Membrane Protein 3	Protein Coding	38	GC01M155255	2.446948	https://www.genecards.org/cgi-bin/carddisp.pl?gene=SCAMP3
KRBOX4	KRAB Box Domain Containing 4	Protein Coding	31	GC0XP046446	2.446542	https://www.genecards.org/cgi-bin/carddisp.pl?gene=KRBOX4
HCG18	HLA Complex Group 18	RNA Gene	16	GC06M046781	2.445027	https://www.genecards.org/cgi-bin/carddisp.pl?gene=HCG18
ZDHHC5	Zinc Finger DHHC-Type Palmitoyltransferase 5	Protein Coding	37	GC11P057670	2.440767	https://www.genecards.org/cgi-bin/carddisp.pl?gene=ZDHHC5
PFDN1	Prefoldin Subunit 1	Protein Coding	39	GC05M140263	2.438779	https://www.genecards.org/cgi-bin/carddisp.pl?gene=PFDN1
LOC100128310	Uncharacterized LOC100128310	RNA Gene	8	GC20M061718	2.437192	https://www.genecards.org/cgi-bin/carddisp.pl?gene=LOC100128310
NET1	Neuroepithelial Cell Transforming 1	Protein Coding	44	GC10P005444	2.435443	https://www.genecards.org/cgi-bin/carddisp.pl?gene=NET1

PCID2	PCI Domain Containing 2	Protein Coding	34	GC13M113167	2.416789	https://www.genecards.org/cgi-bin/carddisp.pl?gene=PCID2
lnc-TM6SF2-1		RNA Gene	4	GC19M019270	2.415186	https://www.genecards.org/cgi-bin/carddisp.pl?gene=lnc-TM6SF2-1
ENSG00000267629		RNA Gene	3	GC19M019254	2.415186	https://www.genecards.org/cgi-bin/carddisp.pl?gene=ENSG00000267629
MIR663A	MicroRNA 663a	RNA Gene	16	GC20M026189	2.410882	https://www.genecards.org/cgi-bin/carddisp.pl?gene=MIR663A
ARID3B	AT-Rich Interaction Domain 3B	Protein Coding	38	GC15P074541	2.406055	https://www.genecards.org/cgi-bin/carddisp.pl?gene=ARID3B
MAS1	MAS1 Proto-Oncogene, G Protein-Coupled Receptor	Protein Coding	40	GC06P159906	2.395122	https://www.genecards.org/cgi-bin/carddisp.pl?gene=MAS1
ENOX2	Ecto-NOX Disulfide-Thiol Exchanger 2	Protein Coding	38	GC0XM130623	2.392108	https://www.genecards.org/cgi-bin/carddisp.pl?gene=ENOX2
AKIP1	A-Kinase Interacting Protein 1	Protein Coding	34	GC11P008911	2.390536	https://www.genecards.org/cgi-bin/carddisp.pl?gene=AKIP1
ME3	Malic Enzyme 3	Protein Coding	40	GC11M086441	2.389818	https://www.genecards.org/cgi-bin/carddisp.pl?gene=ME3
HSALNG0013412		RNA Gene	4	GC02P020202	2.388499	https://www.genecards.org/cgi-bin/carddisp.pl?gene=HSALNG0013412
lnc-COA6-13		RNA Gene	4	GC01P234522	2.388499	https://www.genecards.org/cgi-bin/carddisp.pl?gene=lnc-COA6-13
RF00017-457		RNA Gene	3	GC01M234512	2.388499	https://www.genecards.org/cgi-bin/carddisp.pl?gene=RF00017-457
LINC01679	Long Intergenic Non-Protein Coding RNA 1679	RNA Gene	9	GC21M043360	2.385025	https://www.genecards.org/cgi-bin/carddisp.pl?gene=LINC01679
PGS1	Phosphatidylglycerophosphate Synthase 1	Protein Coding	39	GC17P078378	2.383802	https://www.genecards.org/cgi-bin/carddisp.pl?gene=PGS1
RTKN	Rhotekin	Protein Coding	40	GC02M074583	2.382032	https://www.genecards.org/cgi-bin/carddisp.pl?gene=RTKN
CCDC170	Coiled-Coil Domain Containing 170	Protein Coding	33	GC06P151494	2.379334	https://www.genecards.org/cgi-bin/carddisp.pl?gene=CCDC170
DNASE2B	Deoxyribonuclease 2 Beta	Protein Coding	34	GC01P084398	2.374575	https://www.genecards.org/cgi-bin/carddisp.pl?gene=DNASE2B
EIF2D	Eukaryotic Translation Initiation Factor 2D	Protein Coding	33	GC01M206571	2.370579	https://www.genecards.org/cgi-bin/carddisp.pl?gene=EIF2D
MIR637	MicroRNA 637	RNA Gene	16	GC19M003961	2.369391	https://www.genecards.org/cgi-bin/carddisp.pl?gene=MIR637

LOC389199	Uncharacterized LOC389199	Protein Coding	17	GC04P007939	2.365835	https://www.genecards.org/cgi-bin/carddisp.pl?gene=LOC389199
KCNJ4	Potassium Inwardly Rectifying Channel Subfamily J Member 4	Protein Coding	45	GC22M038426	2.361508	https://www.genecards.org/cgi-bin/carddisp.pl?gene=KCNJ4
BCAS2	BCAS2 Pre-MRNA Processing Factor	Protein Coding	38	GC01M114567	2.349597	https://www.genecards.org/cgi-bin/carddisp.pl?gene=BCAS2
SEMA4C	Semaphorin 4C	Protein Coding	38	GC02M096859	2.341803	https://www.genecards.org/cgi-bin/carddisp.pl?gene=SEMA4C
RHNO1	RAD9-HUS1-RAD1 Interacting Nuclear Orphan 1	Protein Coding	30	GC12P002876	2.338134	https://www.genecards.org/cgi-bin/carddisp.pl?gene=RHNO1
SNUPN	Snurportin 1	Protein Coding	37	GC15M075598	2.337207	https://www.genecards.org/cgi-bin/carddisp.pl?gene=SNUPN
HOPX	HOP Homeobox	Protein Coding	37	GC04M056647	2.336192	https://www.genecards.org/cgi-bin/carddisp.pl?gene=HOPX
lnc-TRIB1-7		RNA Gene	4	GC08P125495	2.335687	https://www.genecards.org/cgi-bin/carddisp.pl?gene=lnc-TRIB1-7
CTDNEP1	CTD Nuclear Envelope Phosphatase 1	Protein Coding	36	GC17M007243	2.334201	https://www.genecards.org/cgi-bin/carddisp.pl?gene=CTDNEP1
ERI1	Exoribonuclease 1	Protein Coding	38	GC08P008892	2.328356	https://www.genecards.org/cgi-bin/carddisp.pl?gene=ERI1
NPTXR	Neuronal Pentraxin Receptor	Protein Coding	40	GC22M038818	2.319512	https://www.genecards.org/cgi-bin/carddisp.pl?gene=NPTXR
SNHG7	Small Nucleolar RNA Host Gene 7	RNA Gene	20	GC09M136821	2.315902	https://www.genecards.org/cgi-bin/carddisp.pl?gene=SNHG7
ESYT2	Extended Synaptotagmin 2	Protein Coding	36	GC07M158730	2.312063	https://www.genecards.org/cgi-bin/carddisp.pl?gene=ESYT2
MRPS21	Mitochondrial Ribosomal Protein S21	Protein Coding	37	GC01P150294	2.305032	https://www.genecards.org/cgi-bin/carddisp.pl?gene=MRPS21
MIR194-1	MicroRNA 194-1	RNA Gene	16	GC01M220118	2.304888	https://www.genecards.org/cgi-bin/carddisp.pl?gene=MIR194-1
EFHD2	EF-Hand Domain Family Member D2	Protein Coding	36	GC01P015409	2.301093	https://www.genecards.org/cgi-bin/carddisp.pl?gene=EFHD2
ZNF300	Zinc Finger Protein 300	Protein Coding	37	GC05M150894	2.30036	https://www.genecards.org/cgi-bin/carddisp.pl?gene=ZNF300
H1-3	H1.3 Linker Histone, Cluster Member	Protein Coding	30	GC06M047205	2.298678	https://www.genecards.org/cgi-bin/carddisp.pl?gene=H1-3
APOL3	Apolipoprotein L3	Protein Coding	37	GC22M036140	2.297211	https://www.genecards.org/cgi-bin/carddisp.pl?gene=APOL3

MED6	Mediator Complex Subunit 6	Protein Coding	38	GC14M070581	2.295582	https://www.genecards.org/cgi-bin/carddisp.pl?gene=MED6
CLGN	Calmegin	Protein Coding	37	GC04M140388	2.295215	https://www.genecards.org/cgi-bin/carddisp.pl?gene=CLGN
HOXA5	Homeobox A5	Protein Coding	42	GC07M027142	2.294472	https://www.genecards.org/cgi-bin/carddisp.pl?gene=HOXA5
TNFAIP8	TNF Alpha Induced Protein 8	Protein Coding	38	GC05P119268	2.293043	https://www.genecards.org/cgi-bin/carddisp.pl?gene=TNFAIP8
KIF15	Kinesin Family Member 15	Protein Coding	37	GC03P045589	2.291771	https://www.genecards.org/cgi-bin/carddisp.pl?gene=KIF15
TMEM18	Transmembrane Protein 18	Protein Coding	36	GC02M000660	2.289251	https://www.genecards.org/cgi-bin/carddisp.pl?gene=TMEM18
SSX2IP	SSX Family Member 2 Interacting Protein	Protein Coding	37	GC01M084643	2.286052	https://www.genecards.org/cgi-bin/carddisp.pl?gene=SSX2IP
ABTB2	Ankyrin Repeat And BTB Domain Containing 2	Protein Coding	33	GC11M034129	2.2856	https://www.genecards.org/cgi-bin/carddisp.pl?gene=ABTB2
FECHP1	Ferrochelatase Pseudogene 1	Pseudogene	6	GC03P034873	2.277014	https://www.genecards.org/cgi-bin/carddisp.pl?gene=FECHP1
CLEC4A	C-Type Lectin Domain Family 4 Member A	Protein Coding	38	GC12P011898	2.276155	https://www.genecards.org/cgi-bin/carddisp.pl?gene=CLEC4A
LINC00322	Long Intergenic Non-Protein Coding RNA 322	RNA Gene	17	GC21M043322	2.275914	https://www.genecards.org/cgi-bin/carddisp.pl?gene=LINC00322
MIR498	MicroRNA 498	RNA Gene	16	GC19P056363	2.275613	https://www.genecards.org/cgi-bin/carddisp.pl?gene=MIR498
TINAGL1	Tubulointerstitial Nephritis Antigen Like 1	Protein Coding	39	GC01P031576	2.274616	https://www.genecards.org/cgi-bin/carddisp.pl?gene=TINAGL1
CENPM	Centromere Protein M	Protein Coding	36	GC22M048546	2.273905	https://www.genecards.org/cgi-bin/carddisp.pl?gene=CENPM
MIR1-1	MicroRNA 1-1	RNA Gene	19	GC20P062994	2.27276	https://www.genecards.org/cgi-bin/carddisp.pl?gene=MIR1-1
CCDC186	Coiled-Coil Domain Containing 186	Protein Coding	29	GC10M114120	2.270911	https://www.genecards.org/cgi-bin/carddisp.pl?gene=CCDC186
NOP16	NOP16 Nucleolar Protein	Protein Coding	33	GC05M176383	2.26935	https://www.genecards.org/cgi-bin/carddisp.pl?gene=NOP16
FNBP4	Formin Binding Protein 4	Protein Coding	36	GC11M069009	2.267901	https://www.genecards.org/cgi-bin/carddisp.pl?gene=FNBP4
ROMO1	Reactive Oxygen Species Modulator 1	Protein Coding	33	GC20P035699	2.267346	https://www.genecards.org/cgi-bin/carddisp.pl?gene=ROMO1
HOXC10	Homeobox C10	Protein Coding	37	GC12P054365	2.266742	https://www.genecards.org/cgi-bin/carddisp.pl?gene=HOXC10

IPO13	Importin 13	Protein Coding	38	GC01P043947	2.264948	https://www.genecards.org/cgi-bin/carddisp.pl?gene=IPO13
LTB4R2	Leukotriene B4 Receptor 2	Protein Coding	43	GC14P026323	2.264258	https://www.genecards.org/cgi-bin/carddisp.pl?gene=LTB4R2
ENSG00000286231		Protein Coding	5	GC01P220787	2.263066	https://www.genecards.org/cgi-bin/carddisp.pl?gene=ENSG00000286231
RNPEP	Arginyl Aminopeptidase	Protein Coding	40	GC01P201982	2.261432	https://www.genecards.org/cgi-bin/carddisp.pl?gene=RNPEP
CDV3	CDV3 Homolog	Protein Coding	31	GC03P133573	2.261302	https://www.genecards.org/cgi-bin/carddisp.pl?gene=CDV3
LOC644135	Uncharacterized LOC644135	RNA Gene	11	GC06M135856	2.260223	https://www.genecards.org/cgi-bin/carddisp.pl?gene=LOC644135
IGHG4	Immunoglobulin Heavy Constant Gamma 4 (G4m Marker)	Protein Coding	27	GC14M110309	2.255379	https://www.genecards.org/cgi-bin/carddisp.pl?gene=IGHG4
SLU7	SLU7 Homolog, Splicing Factor	Protein Coding	38	GC05M160401	2.254393	https://www.genecards.org/cgi-bin/carddisp.pl?gene=SLU7
LOC101928135	Uncharacterized LOC101928135	RNA Gene	6	GC03M034875	2.254169	https://www.genecards.org/cgi-bin/carddisp.pl?gene=LOC101928135
MBD3	Methyl-CpG Binding Domain Protein 3	Protein Coding	43	GC19M002160	2.249501	https://www.genecards.org/cgi-bin/carddisp.pl?gene=MBD3
LMAN1L	Lectin, Mannose Binding 1 Like	Protein Coding	36	GC15P074812	2.248011	https://www.genecards.org/cgi-bin/carddisp.pl?gene=LMAN1L
CUEDC2	CUE Domain Containing 2	Protein Coding	38	GC10M102424	2.24705	https://www.genecards.org/cgi-bin/carddisp.pl?gene=CUEDC2
ARPC4	Actin Related Protein 2/3 Complex Subunit 4	Protein Coding	38	GC03P009792	2.246302	https://www.genecards.org/cgi-bin/carddisp.pl?gene=ARPC4
ENSG00000287092		RNA Gene	5	GC06M155810	2.244387	https://www.genecards.org/cgi-bin/carddisp.pl?gene=ENSG00000287092
TEX36-AS1	TEX36 Antisense RNA 1	RNA Gene	11	GC10P125574	2.242818	https://www.genecards.org/cgi-bin/carddisp.pl?gene=TEX36-AS1
RAB14	RAB14, Member RAS Oncogene Family	Protein Coding	41	GC09M121178	2.235089	https://www.genecards.org/cgi-bin/carddisp.pl?gene=RAB14
ESYT1	Extended Synaptotagmin 1	Protein Coding	38	GC12P056379	2.229225	https://www.genecards.org/cgi-bin/carddisp.pl?gene=ESYT1
ITGB3BP	Integrin Subunit Beta 3 Binding Protein	Protein Coding	42	GC01M063440	2.225029	https://www.genecards.org/cgi-bin/carddisp.pl?gene=ITGB3BP

HSDL2	Hydroxysteroid Dehydrogenase Like 2	Protein Coding	34	GC09P112379	2.224586	https://www.genecards.org/cgi-bin/carddisp.pl?gene=HSDL2
METAP1	Methionyl Aminopeptidase 1	Protein Coding	41	GC04P098995	2.223778	https://www.genecards.org/cgi-bin/carddisp.pl?gene=METAP1
ARHGEF28	Rho Guanine Nucleotide Exchange Factor 28	Protein Coding	36	GC05P073626	2.222673	https://www.genecards.org/cgi-bin/carddisp.pl?gene=ARHGEF28
ANO4	Anoctamin 4	Protein Coding	35	GC12P100717	2.221799	https://www.genecards.org/cgi-bin/carddisp.pl?gene=ANO4
MTRNR2L5	MT-RNR2 Like 5	Protein Coding	20	GC10P055618	2.21551	https://www.genecards.org/cgi-bin/carddisp.pl?gene=MTRNR2L5
ZBTB17	Zinc Finger And BTB Domain Containing 17	Protein Coding	42	GC01M015943	2.21314	https://www.genecards.org/cgi-bin/carddisp.pl?gene=ZBTB17
H3C10	H3 Clustered Histone 10	Protein Coding	29	GC06P055047	2.21023	https://www.genecards.org/cgi-bin/carddisp.pl?gene=H3C10
RPS6KB2	Ribosomal Protein S6 Kinase B2	Protein Coding	47	GC11P067428	2.209503	https://www.genecards.org/cgi-bin/carddisp.pl?gene=RPS6KB2
MRPL33	Mitochondrial Ribosomal Protein L33	Protein Coding	33	GC02P027772	2.208936	https://www.genecards.org/cgi-bin/carddisp.pl?gene=MRPL33
SHB	SH2 Domain Containing Adaptor Protein B	Protein Coding	41	GC09M038348	2.207877	https://www.genecards.org/cgi-bin/carddisp.pl?gene=SHB
CCDC93	Coiled-Coil Domain Containing 93	Protein Coding	34	GC02M117915	2.203646	https://www.genecards.org/cgi-bin/carddisp.pl?gene=CCDC93
HSP90AA2P	Heat Shock Protein 90 Alpha Family Class A Member 2, Pseudogene	Pseudogene	19	GC11M027888	2.200334	https://www.genecards.org/cgi-bin/carddisp.pl?gene=HSP90AA2P
H2AC12	H2A Clustered Histone 12	Protein Coding	28	GC06P055578	2.20021	https://www.genecards.org/cgi-bin/carddisp.pl?gene=H2AC12
SIGLEC14	Sialic Acid Binding Ig Like Lectin 14	Protein Coding	33	GC19M051642	2.197856	https://www.genecards.org/cgi-bin/carddisp.pl?gene=SIGLEC14
PHACTR3	Phosphatase And Actin Regulator 3	Protein Coding	37	GC20P059577	2.197795	https://www.genecards.org/cgi-bin/carddisp.pl?gene=PHACTR3
TAS1R3	Taste 1 Receptor Member 3	Protein Coding	40	GC01P001331	2.197476	https://www.genecards.org/cgi-bin/carddisp.pl?gene=TAS1R3
CYTH2	Cytohesin 2	Protein Coding	42	GC19P048470	2.196087	https://www.genecards.org/cgi-bin/carddisp.pl?gene=CYTH2
MROH7	Maestro Heat Like Repeat Family Member 7	Protein Coding	31	GC01P054643	2.192661	https://www.genecards.org/cgi-bin/carddisp.pl?gene=MROH7

ODF1	Outer Dense Fiber Of Sperm Tails 1	Protein Coding	37	GC08P102551	2.191673	https://www.genecards.org/cgi-bin/carddisp.pl?gene=ODF1
H2AC17	H2A Clustered Histone 17	Protein Coding	29	GC06M047209	2.190752	https://www.genecards.org/cgi-bin/carddisp.pl?gene=H2AC17
SEPTIN11	Septin 11	Protein Coding	29	GC04P076950	2.186701	https://www.genecards.org/cgi-bin/carddisp.pl?gene=SEPTIN11
ENSG00000228852		RNA Gene	7	GC01M095243	2.186076	https://www.genecards.org/cgi-bin/carddisp.pl?gene=ENSG00000228852
C1orf87	Chromosome 1 Open Reading Frame 87	Protein Coding	34	GC01M059987	2.185571	https://www.genecards.org/cgi-bin/carddisp.pl?gene=C1orf87
HMGN4	High Mobility Group Nucleosomal Binding Domain 4	Protein Coding	33	GC06P026538	2.181639	https://www.genecards.org/cgi-bin/carddisp.pl?gene=HMGN4
APOL6	Apolipoprotein L6	Protein Coding	38	GC22P035648	2.17594	https://www.genecards.org/cgi-bin/carddisp.pl?gene=APOL6
EFNA3	Ephrin A3	Protein Coding	45	GC01P155078	2.174744	https://www.genecards.org/cgi-bin/carddisp.pl?gene=EFNA3
MIR302F	MicroRNA 302f	RNA Gene	9	GC18P030298	2.174495	https://www.genecards.org/cgi-bin/carddisp.pl?gene=MIR302F
TSSK2	Testis Specific Serine Kinase 2	Protein Coding	36	GC22P020052	2.173967	https://www.genecards.org/cgi-bin/carddisp.pl?gene=TSSK2
CNNM3	Cyclin And CBS Domain Divalent Metal Cation Transport Mediator 3	Protein Coding	38	GC02P096815	2.172954	https://www.genecards.org/cgi-bin/carddisp.pl?gene=CNNM3
SLC22A13	Solute Carrier Family 22 Member 13	Protein Coding	38	GC03P038265	2.169709	https://www.genecards.org/cgi-bin/carddisp.pl?gene=SLC22A13
PRMT3	Protein Arginine Methyltransferase 3	Protein Coding	43	GC11P020409	2.169315	https://www.genecards.org/cgi-bin/carddisp.pl?gene=PRMT3
ENSG00000287292		RNA Gene	6	GC04P148445	2.167833	https://www.genecards.org/cgi-bin/carddisp.pl?gene=ENSG00000287292
ENSG00000254599		RNA Gene	5	GC11P098676	2.167833	https://www.genecards.org/cgi-bin/carddisp.pl?gene=ENSG00000254599
HSALNG0133406		RNA Gene	3	GC21P043345	2.167833	https://www.genecards.org/cgi-bin/carddisp.pl?gene=HSALNG0133406
MIR383	MicroRNA 383	RNA Gene	15	GC08M014853	2.166983	https://www.genecards.org/cgi-bin/carddisp.pl?gene=MIR383
HNRNPH3	Heterogeneous Nuclear Ribonucleoprotein H3	Protein Coding	39	GC10P068331	2.154793	https://www.genecards.org/cgi-bin/carddisp.pl?gene=HNRNPH3
ZNF703	Zinc Finger Protein 703	Protein Coding	36	GC08P037695	2.153392	https://www.genecards.org/cgi-bin/carddisp.pl?gene=ZNF703

MNX1-AS1	MNX1 Antisense RNA 1 (Head To Head)	RNA Gene	16	GC07P157010	2.150087	https://www.genecards.org/cgi-bin/carddisp.pl?gene=MNX1-AS1
GINS3	GINS Complex Subunit 3	Protein Coding	34	GC16P058328	2.148112	https://www.genecards.org/cgi-bin/carddisp.pl?gene=GINS3
ANKHD1	Ankyrin Repeat And KH Domain Containing 1	Protein Coding	35	GC05P143777	2.144107	https://www.genecards.org/cgi-bin/carddisp.pl?gene=ANKHD1
H2AC7	H2A Clustered Histone 7	Protein Coding	29	GC06M047210	2.141566	https://www.genecards.org/cgi-bin/carddisp.pl?gene=H2AC7
H2AC16	H2A Clustered Histone 16	Protein Coding	29	GC06P055054	2.140799	https://www.genecards.org/cgi-bin/carddisp.pl?gene=H2AC16
NOA1	Nitric Oxide Associated 1	Protein Coding	34	GC04M056963	2.140753	https://www.genecards.org/cgi-bin/carddisp.pl?gene=NOA1
CORO2B	Coronin 2B	Protein Coding	38	GC15P077105	2.134557	https://www.genecards.org/cgi-bin/carddisp.pl?gene=CORO2B
ENSG00000272823		RNA Gene	5	GC01M220828	2.13395	https://www.genecards.org/cgi-bin/carddisp.pl?gene=ENSG00000272823
HSALNG0010758		RNA Gene	2	GC01P220796	2.13395	https://www.genecards.org/cgi-bin/carddisp.pl?gene=HSALNG0010758
lnc-MARC2-2		RNA Gene	2	GC01P220804	2.13395	https://www.genecards.org/cgi-bin/carddisp.pl?gene=lnc-MARC2-2
PCOLCE	Procollagen C-Endopeptidase Enhancer	Protein Coding	40	GC07P100602	2.132756	https://www.genecards.org/cgi-bin/carddisp.pl?gene=PCOLCE
CASP8AP2	Caspase 8 Associated Protein 2	Protein Coding	34	GC06P089829	2.128404	https://www.genecards.org/cgi-bin/carddisp.pl?gene=CASP8AP2
ZC3H12A	Zinc Finger CCCH-Type Containing 12A	Protein Coding	37	GC01P037474	2.124047	https://www.genecards.org/cgi-bin/carddisp.pl?gene=ZC3H12A
C16orf72	Chromosome 16 Open Reading Frame 72	Protein Coding	32	GC16P009103	2.117929	https://www.genecards.org/cgi-bin/carddisp.pl?gene=C16orf72
ATP6AP1L	ATPase H+ Transporting Accessory Protein 1 Like	Protein Coding	26	GC05P082279	2.116091	https://www.genecards.org/cgi-bin/carddisp.pl?gene=ATP6AP1L
H2BC5	H2B Clustered Histone 5	Protein Coding	29	GC06P055583	2.114598	https://www.genecards.org/cgi-bin/carddisp.pl?gene=H2BC5
PGLS	6-Phosphogluconolactonase	Protein Coding	39	GC19P026640	2.113848	https://www.genecards.org/cgi-bin/carddisp.pl?gene=PGLS
HMCES	5-Hydroxymethylcytosine Binding, ES Cell Specific	Protein Coding	31	GC03P129278	2.112906	https://www.genecards.org/cgi-bin/carddisp.pl?gene=HMCES

RAPGEF6	Rap Guanine Nucleotide Exchange Factor 6	Protein Coding	39	GC05M131423	2.111747	https://www.genecards.org/cgi-bin/carddisp.pl?gene=RAPGEF6
H3C7	H3 Clustered Histone 7	Protein Coding	28	GC06M047217	2.107437	https://www.genecards.org/cgi-bin/carddisp.pl?gene=H3C7
GAPDHS	Glyceraldehyde-3-Phosphate Dehydrogenase, Spermatogenic	Protein Coding	43	GC19P040351	2.107219	https://www.genecards.org/cgi-bin/carddisp.pl?gene=GAPDHS
H3C12	H3 Clustered Histone 12	Protein Coding	29	GC06M047215	2.10359	https://www.genecards.org/cgi-bin/carddisp.pl?gene=H3C12
KLK14	Kallikrein Related Peptidase 14	Protein Coding	38	GC19M051077	2.096282	https://www.genecards.org/cgi-bin/carddisp.pl?gene=KLK14
TPI1P1	Triosephosphate Isomerase 1 Pseudogene 1	Pseudogene	7	GC01P076699	2.095212	https://www.genecards.org/cgi-bin/carddisp.pl?gene=TPI1P1
RAB24	RAB24, Member RAS Oncogene Family	Protein Coding	37	GC05M177301	2.089585	https://www.genecards.org/cgi-bin/carddisp.pl?gene=RAB24
ELP6	Elongator Acetyltransferase Complex Subunit 6	Protein Coding	33	GC03M047495	2.086529	https://www.genecards.org/cgi-bin/carddisp.pl?gene=ELP6
SLFN11	Schlafen Family Member 11	Protein Coding	34	GC17M035350	2.084761	https://www.genecards.org/cgi-bin/carddisp.pl?gene=SLFN11
OLFM1	Olfactomedin 1	Protein Coding	40	GC09P135075	2.082146	https://www.genecards.org/cgi-bin/carddisp.pl?gene=OLFM1
ENSG00000255046		RNA Gene	8	GC08M011991	2.080244	https://www.genecards.org/cgi-bin/carddisp.pl?gene=ENSG00000255046
ENSG00000222042		RNA Gene	6	GC21M025169	2.080244	https://www.genecards.org/cgi-bin/carddisp.pl?gene=ENSG00000222042
OTX2P1	OTX2 Pseudogene 1	Pseudogene	5	GC09M075724	2.080244	https://www.genecards.org/cgi-bin/carddisp.pl?gene=OTX2P1
lnc-JCAD-2		RNA Gene	5	GC10M031184	2.080244	https://www.genecards.org/cgi-bin/carddisp.pl?gene=lnc-JCAD-2
ENSG00000237936		Pseudogene	4	GC10M029891	2.080244	https://www.genecards.org/cgi-bin/carddisp.pl?gene=ENSG00000237936
SLC25A48	Solute Carrier Family 25 Member 48	Protein Coding	32	GC05P135579	2.077582	https://www.genecards.org/cgi-bin/carddisp.pl?gene=SLC25A48
DAP	Death Associated Protein	Protein Coding	40	GC05M010679	2.076235	https://www.genecards.org/cgi-bin/carddisp.pl?gene=DAP
RPS27P18	Ribosomal Protein S27 Pseudogene 18	Pseudogene	6	GC10M125473	2.073174	https://www.genecards.org/cgi-bin/carddisp.pl?gene=RPS27P18

THNSL2	Threonine Synthase Like 2	Protein Coding	33	GC02P088170	2.072518	https://www.genecards.org/cgi-bin/carddisp.pl?gene=THNSL2
EXOC7	Exocyst Complex Component 7	Protein Coding	42	GC17M076080	2.06974	https://www.genecards.org/cgi-bin/carddisp.pl?gene=EXOC7
DUSP14	Dual Specificity Phosphatase 14	Protein Coding	38	GC17P037489	2.069582	https://www.genecards.org/cgi-bin/carddisp.pl?gene=DUSP14
IPO8	Importin 8	Protein Coding	36	GC12M030628	2.067233	https://www.genecards.org/cgi-bin/carddisp.pl?gene=IPO8
ARPC1A	Actin Related Protein 2/3 Complex Subunit 1A	Protein Coding	41	GC07P099325	2.066761	https://www.genecards.org/cgi-bin/carddisp.pl?gene=ARPC1A
LAMTOR1	Late Endosomal/Lysosomal Adaptor, MAPK And MTOR Activator 1	Protein Coding	36	GC11M072085	2.063451	https://www.genecards.org/cgi-bin/carddisp.pl?gene=LAMTOR1
RADX	RPA1 Related Single Stranded DNA Binding Protein, X-Linked	Protein Coding	26	GC0XP106612	2.057263	https://www.genecards.org/cgi-bin/carddisp.pl?gene=RADX
DGAT2L7P	Diacylglycerol O-Acyltransferase 2 Like 7, Pseudogene	Pseudogene	12	GC07M101201	2.055746	https://www.genecards.org/cgi-bin/carddisp.pl?gene=DGAT2L7P
ARFGEF3	ARFGEF Family Member 3	Protein Coding	30	GC06P138161	2.054548	https://www.genecards.org/cgi-bin/carddisp.pl?gene=ARFGEF3
CEP85	Centrosomal Protein 85	Protein Coding	35	GC01P026234	2.050153	https://www.genecards.org/cgi-bin/carddisp.pl?gene=CEP85
LINC00598	Long Intergenic Non-Protein Coding RNA 598	RNA Gene	16	GC13M040088	2.047847	https://www.genecards.org/cgi-bin/carddisp.pl?gene=LINC00598
MIR190B	MicroRNA 190b	RNA Gene	15	GC01M154193	2.042446	https://www.genecards.org/cgi-bin/carddisp.pl?gene=MIR190B
TRAPPC13	Trafficking Protein Particle Complex Subunit 13	Protein Coding	31	GC05P065625	2.04026	https://www.genecards.org/cgi-bin/carddisp.pl?gene=TRAPPC13
TSKU	Tsukushi, Small Leucine Rich Proteoglycan	Protein Coding	34	GC11P076782	2.038889	https://www.genecards.org/cgi-bin/carddisp.pl?gene=TSKU
SGPP1	Sphingosine-1-Phosphate Phosphatase 1	Protein Coding	40	GC14M063684	2.038044	https://www.genecards.org/cgi-bin/carddisp.pl?gene=SGPP1
PLD4	Phospholipase D Family Member 4	Protein Coding	40	GC14P104924	2.034567	https://www.genecards.org/cgi-bin/carddisp.pl?gene=PLD4

STARP1	Steroidogenic Acute Regulatory Protein Pseudogene 1	Pseudogene	7	GC13M065280	2.028617	https://www.genecards.org/cgi-bin/carddisp.pl?gene=STARP1
RAB9A	RAB9A, Member RAS Oncogene Family	Protein Coding	40	GC0XP013707	2.026031	https://www.genecards.org/cgi-bin/carddisp.pl?gene=RAB9A
GUCA2B	Guanylate Cyclase Activator 2B	Protein Coding	40	GC01P042153	2.025527	https://www.genecards.org/cgi-bin/carddisp.pl?gene=GUCA2B
ASTN1	Astrotactin 1	Protein Coding	34	GC01M176857	2.018537	https://www.genecards.org/cgi-bin/carddisp.pl?gene=ASTN1
PDIA2	Protein Disulfide Isomerase Family A Member 2	Protein Coding	39	GC16P005498	2.018224	https://www.genecards.org/cgi-bin/carddisp.pl?gene=PDIA2
PSAPL1	Prosaposin Like 1	Protein Coding	33	GC04M007434	2.018184	https://www.genecards.org/cgi-bin/carddisp.pl?gene=PSAPL1
ENSG00000234389		RNA Gene	8	GC02P102438	2.017039	https://www.genecards.org/cgi-bin/carddisp.pl?gene=ENSG00000234389
ENSG00000249631		RNA Gene	7	GC04P014604	2.017039	https://www.genecards.org/cgi-bin/carddisp.pl?gene=ENSG00000249631
HSALNG0088100		RNA Gene	4	GC11M128462	2.017039	https://www.genecards.org/cgi-bin/carddisp.pl?gene=HSALNG0088100
HSALNG0088101		RNA Gene	4	GC11P128480	2.017039	https://www.genecards.org/cgi-bin/carddisp.pl?gene=HSALNG0088101
HSALNG0088102		RNA Gene	4	GC11M128489	2.017039	https://www.genecards.org/cgi-bin/carddisp.pl?gene=HSALNG0088102
C15orf48	Chromosome 15 Open Reading Frame 48	Protein Coding	34	GC15P045430	2.01301	https://www.genecards.org/cgi-bin/carddisp.pl?gene=C15orf48
HSH2D	Hematopoietic SH2 Domain Containing	Protein Coding	35	GC19P026611	2.007876	https://www.genecards.org/cgi-bin/carddisp.pl?gene=HSH2D
BCAS1	Brain Enriched Myelin Associated Protein 1	Protein Coding	38	GC20M053936	2.00727	https://www.genecards.org/cgi-bin/carddisp.pl?gene=BCAS1
TXNDC5	Thioredoxin Domain Containing 5	Protein Coding	39	GC06M007893	2.003392	https://www.genecards.org/cgi-bin/carddisp.pl?gene=TXNDC5
LINC00355	Long Intergenic Non-Protein Coding RNA 355	RNA Gene	15	GC13M063838	2.003167	https://www.genecards.org/cgi-bin/carddisp.pl?gene=LINC00355
LOC105373170	Uncharacterized LOC105373170	RNA Gene	6	GC01M231768	2.003167	https://www.genecards.org/cgi-bin/carddisp.pl?gene=LOC105373170

PLK3	Polo Like Kinase 3	Protein Coding	44	GC01P044799	2.002878	https://www.genecards.org/cgi-bin/carddisp.pl?gene=PLK3
HIF1A-AS2	HIF1A Antisense RNA 2	RNA Gene	16	GC14M061747	2.002368	https://www.genecards.org/cgi-bin/carddisp.pl?gene=HIF1A-AS2
PRAM1	PML-RARA Regulated Adaptor Molecule 1	Protein Coding	34	GC19M008490	2.001507	https://www.genecards.org/cgi-bin/carddisp.pl?gene=PRAM1
PLPP1	Phospholipid Phosphatase 1	Protein Coding	34	GC05M055425	1.994192	https://www.genecards.org/cgi-bin/carddisp.pl?gene=PLPP1
PYHIN1	Pyrin And HIN Domain Family Member 1	Protein Coding	36	GC01P158900	1.993069	https://www.genecards.org/cgi-bin/carddisp.pl?gene=PYHIN1
DNAJC11	DnaJ Heat Shock Protein Family (Hsp40) Member C11	Protein Coding	35	GC01M006634	1.990315	https://www.genecards.org/cgi-bin/carddisp.pl?gene=DNAJC11
UGT2A1	UDP Glucuronosyltransferase Family 2 Member A1 Complex Locus	Protein Coding	34	GC04M069588	1.983822	https://www.genecards.org/cgi-bin/carddisp.pl?gene=UGT2A1
CENPW	Centromere Protein W	Protein Coding	34	GC06P126339	1.983765	https://www.genecards.org/cgi-bin/carddisp.pl?gene=CENPW
KRT8P18	Keratin 8 Pseudogene 18	Pseudogene	5	GC03M035233	1.978504	https://www.genecards.org/cgi-bin/carddisp.pl?gene=KRT8P18
BEX2	Brain Expressed X-Linked 2	Protein Coding	31	GC0XM103309	1.976324	https://www.genecards.org/cgi-bin/carddisp.pl?gene=BEX2
DHRS4L2	Dehydrogenase/Reductase 4 Like 2	Protein Coding	34	GC14P026318	1.976133	https://www.genecards.org/cgi-bin/carddisp.pl?gene=DHRS4L2
MLF2	Myeloid Leukemia Factor 2	Protein Coding	36	GC12M006750	1.974125	https://www.genecards.org/cgi-bin/carddisp.pl?gene=MLF2
FAM172A	Family With Sequence Similarity 172 Member A	Protein Coding	37	GC05M093617	1.972884	https://www.genecards.org/cgi-bin/carddisp.pl?gene=FAM172A
YIPF6	Yip1 Domain Family Member 6	Protein Coding	34	GC0XP068498	1.97256	https://www.genecards.org/cgi-bin/carddisp.pl?gene=YIPF6
TRIM47	Tripartite Motif Containing 47	Protein Coding	34	GC17M075874	1.969799	https://www.genecards.org/cgi-bin/carddisp.pl?gene=TRIM47
SLC9A9-AS1	SLC9A9 Antisense RNA 1	RNA Gene	13	GC03P143342	1.966096	https://www.genecards.org/cgi-bin/carddisp.pl?gene=SLC9A9-AS1
LOC100130331	POTE Ankyrin Domain Family, Member F Pseudogene	Pseudogene	10	GC01P237862	1.966096	https://www.genecards.org/cgi-bin/carddisp.pl?gene=LOC100130331
ENSG00000257322		RNA Gene	8	GC12M093003	1.966096	https://www.genecards.org/cgi-bin/carddisp.pl?gene=ENSG00000257322

ENSG00000236501		RNA Gene	8	GC02M176959	1.966096	https://www.genecards.org/cgi-bin/carddisp.pl?gene=ENSG00000236501
RNU6-908P	RNA, U6 Small Nuclear 908, Pseudogene	Pseudogene	7	GC10P029711	1.966096	https://www.genecards.org/cgi-bin/carddisp.pl?gene=RNU6-908P
ENSG00000250541		RNA Gene	6	GC04P025771	1.966096	https://www.genecards.org/cgi-bin/carddisp.pl?gene=ENSG00000250541
ENSG00000265994		RNA Gene	6	GC18P028638	1.966096	https://www.genecards.org/cgi-bin/carddisp.pl?gene=ENSG00000265994
ENSG00000207171		RNA Gene	6	GC04P168471	1.966096	https://www.genecards.org/cgi-bin/carddisp.pl?gene=ENSG00000207171
EEF1A1P20	Eukaryotic Translation Elongation Factor 1 Alpha 1 Pseudogene 20	Pseudogene	5	GC05P099996	1.966096	https://www.genecards.org/cgi-bin/carddisp.pl?gene=EEF1A1P20
RNU1-35P	RNA, U1 Small Nuclear 35, Pseudogene	Pseudogene	5	GC08M135742	1.966096	https://www.genecards.org/cgi-bin/carddisp.pl?gene=RNU1-35P
ENSG00000235464		RNA Gene	5	GC07M035496	1.966096	https://www.genecards.org/cgi-bin/carddisp.pl?gene=ENSG00000235464
ENSG00000236389		RNA Gene	5	GC06M134706	1.966096	https://www.genecards.org/cgi-bin/carddisp.pl?gene=ENSG00000236389
RNU6-161P	RNA, U6 Small Nuclear 161, Pseudogene	Pseudogene	5	GC01P076753	1.966096	https://www.genecards.org/cgi-bin/carddisp.pl?gene=RNU6-161P
MTND1P25	MT-ND1 Pseudogene 25	Pseudogene	4	GC01M237942	1.966096	https://www.genecards.org/cgi-bin/carddisp.pl?gene=MTND1P25
ENSG00000284676		RNA Gene	4	GC01P029755	1.966096	https://www.genecards.org/cgi-bin/carddisp.pl?gene=ENSG00000284676
ENSG00000287032		RNA Gene	4	GC07M035614	1.966096	https://www.genecards.org/cgi-bin/carddisp.pl?gene=ENSG00000287032
ENSG00000276627		RNA Gene	4	GC20P060716	1.966096	https://www.genecards.org/cgi-bin/carddisp.pl?gene=ENSG00000276627
HSALNG0076986		RNA Gene	4	GC10M031161	1.966096	https://www.genecards.org/cgi-bin/carddisp.pl?gene=HSALNG0076986
lnc-CRYAA-1		RNA Gene	4	GC21P043350	1.966096	https://www.genecards.org/cgi-bin/carddisp.pl?gene=lnc-CRYAA-1
piR-55809-001		RNA Gene	4	GC01M095168	1.966096	https://www.genecards.org/cgi-bin/carddisp.pl?gene=piR-55809-001
lnc-DDX60L-3		RNA Gene	4	GC04M168468	1.966096	https://www.genecards.org/cgi-bin/carddisp.pl?gene=lnc-DDX60L-3

LOC102723733	Uncharacterized LOC102723733	RNA Gene	3	GC04P025862	1.966096	https://www.genecards.org/cgi-bin/carddisp.pl?gene=LOC102723733
ENSG00000270927		Pseudogene	3	GC01M029904	1.966096	https://www.genecards.org/cgi-bin/carddisp.pl?gene=ENSG00000270927
ENSG00000226973		Pseudogene	3	GC01P115471	1.966096	https://www.genecards.org/cgi-bin/carddisp.pl?gene=ENSG00000226973
LOC107986198	Uncharacterized LOC107986198	RNA Gene	3	GC04M168488	1.966096	https://www.genecards.org/cgi-bin/carddisp.pl?gene=LOC107986198
lnc-EDRF1-4		RNA Gene	3	GC10P125590	1.966096	https://www.genecards.org/cgi-bin/carddisp.pl?gene=lnc-EDRF1-4
HSALNG0038430		RNA Gene	3	GC04P168487	1.966096	https://www.genecards.org/cgi-bin/carddisp.pl?gene=HSALNG0038430
HSALNG0029504		RNA Gene	3	GC03P143161	1.966096	https://www.genecards.org/cgi-bin/carddisp.pl?gene=HSALNG0029504
HSALNG0076987-001		RNA Gene	3	GC10P029811	1.966096	https://www.genecards.org/cgi-bin/carddisp.pl?gene=HSALNG0076987-001
HSALNG0076987-002		RNA Gene	3	GC10P029812	1.966096	https://www.genecards.org/cgi-bin/carddisp.pl?gene=HSALNG0076987-002
lnc-AIFM2-3		RNA Gene	3	GC10M069839	1.966096	https://www.genecards.org/cgi-bin/carddisp.pl?gene=lnc-AIFM2-3
HSALNG0093093		RNA Gene	3	GC12M093112	1.966096	https://www.genecards.org/cgi-bin/carddisp.pl?gene=HSALNG0093093
piR-38051-198		RNA Gene	3	GC04M025877	1.966096	https://www.genecards.org/cgi-bin/carddisp.pl?gene=piR-38051-198
piR-41195-080		RNA Gene	3	GC08M012002	1.966096	https://www.genecards.org/cgi-bin/carddisp.pl?gene=piR-41195-080
piR-59297-626		RNA Gene	3	GC08P011819	1.966096	https://www.genecards.org/cgi-bin/carddisp.pl?gene=piR-59297-626
AB372775		RNA Gene	2	GC10P069827	1.966096	https://www.genecards.org/cgi-bin/carddisp.pl?gene=AB372775
HSALNG0029512		RNA Gene	2	GC03P143373	1.966096	https://www.genecards.org/cgi-bin/carddisp.pl?gene=HSALNG0029512
HSALNG0093097		RNA Gene	2	GC12M093154	1.966096	https://www.genecards.org/cgi-bin/carddisp.pl?gene=HSALNG0093097
piR-31109-001		RNA Gene	2	GC10P125633	1.966096	https://www.genecards.org/cgi-bin/carddisp.pl?gene=piR-31109-001
piR-57133-376		RNA Gene	2	GC20P060683	1.966096	https://www.genecards.org/cgi-bin/carddisp.pl?gene=piR-57133-376

HSALNG0131408		RNA Gene	2	GC20M060680	1.966096	https://www.genecards.org/cgi-bin/carddisp.pl?gene=HSALNG0131408
ENSG00000254939		Pseudogene	1	GC11M098568	1.966096	https://www.genecards.org/cgi-bin/carddisp.pl?gene=ENSG00000254939
HSALNG0068856		RNA Gene	1	GC08M135698	1.966096	https://www.genecards.org/cgi-bin/carddisp.pl?gene=HSALNG0068856
H2BC9	H2B Clustered Histone 9	Protein Coding	30	GC06P055586	1.958859	https://www.genecards.org/cgi-bin/carddisp.pl?gene=H2BC9
NR6A1	Nuclear Receptor Subfamily 6 Group A Member 1	Protein Coding	41	GC09M124517	1.957877	https://www.genecards.org/cgi-bin/carddisp.pl?gene=NR6A1
POT1-AS1	POT1 Antisense RNA 1	RNA Gene	15	GC07P124929	1.951397	https://www.genecards.org/cgi-bin/carddisp.pl?gene=POT1-AS1
MRPL40	Mitochondrial Ribosomal Protein L40	Protein Coding	38	GC22P020060	1.949222	https://www.genecards.org/cgi-bin/carddisp.pl?gene=MRPL40
PBX2P1	PBX Homeobox 2 Pseudogene 1	Pseudogene	11	GC03P143176	1.94884	https://www.genecards.org/cgi-bin/carddisp.pl?gene=PBX2P1
H2AC6	H2A Clustered Histone 6	Protein Coding	27	GC06P055579	1.948534	https://www.genecards.org/cgi-bin/carddisp.pl?gene=H2AC6
FAM98A	Family With Sequence Similarity 98 Member A	Protein Coding	31	GC02M033583	1.944726	https://www.genecards.org/cgi-bin/carddisp.pl?gene=FAM98A
DPP10-AS1	DPP10 Antisense RNA 1	RNA Gene	14	GC02M115130	1.944704	https://www.genecards.org/cgi-bin/carddisp.pl?gene=DPP10-AS1
LINC02629	Long Intergenic Non-Protein Coding RNA 2629	RNA Gene	6	GC10P033910	1.943198	https://www.genecards.org/cgi-bin/carddisp.pl?gene=LINC02629
RNU6-976P	RNA, U6 Small Nuclear 976, Pseudogene	Pseudogene	5	GC14P084739	1.943198	https://www.genecards.org/cgi-bin/carddisp.pl?gene=RNU6-976P
ENSG00000268473		Pseudogene	2	GC16M086434	1.943198	https://www.genecards.org/cgi-bin/carddisp.pl?gene=ENSG00000268473
HSALNG0055801		RNA Gene	2	GC07P004174	1.943198	https://www.genecards.org/cgi-bin/carddisp.pl?gene=HSALNG0055801
LINC00520	Long Intergenic Non-Protein Coding RNA 520	RNA Gene	17	GC14M055781	1.941139	https://www.genecards.org/cgi-bin/carddisp.pl?gene=LINC00520
EPB41L4A-DT	EPB41L4A Divergent Transcript	RNA Gene	15	GC05P112421	1.937899	https://www.genecards.org/cgi-bin/carddisp.pl?gene=EPB41L4A-DT

CALCOCO1	Calcium Binding And Coiled-Coil Domain 1	Protein Coding	37	GC12M053709	1.934899	https://www.genecards.org/cgi-bin/carddisp.pl?gene=CALCOCO1
DGKG	Diacylglycerol Kinase Gamma	Protein Coding	44	GC03M186105	1.934794	https://www.genecards.org/cgi-bin/carddisp.pl?gene=DGKG
ST13P12	ST13, Hsp70 Interacting Protein Pseudogene 12	Pseudogene	6	GC05P082968	1.933577	https://www.genecards.org/cgi-bin/carddisp.pl?gene=ST13P12
H2AC1	H2A Clustered Histone 1	Protein Coding	30	GC06M025873	1.922436	https://www.genecards.org/cgi-bin/carddisp.pl?gene=H2AC1
MIR629	MicroRNA 629	RNA Gene	16	GC15M070079	1.921234	https://www.genecards.org/cgi-bin/carddisp.pl?gene=MIR629
IRAIN	IGF1R Antisense Imprinted Non-Protein Coding RNA	RNA Gene	13	GC15M098645	1.920473	https://www.genecards.org/cgi-bin/carddisp.pl?gene=IRAIN
RPL17-C18orf32	RPL17-C18orf32 Readthrough	Protein Coding	21	GC18M049481	1.91836	https://www.genecards.org/cgi-bin/carddisp.pl?gene=RPL17-C18orf32
LINC00333	Long Intergenic Non-Protein Coding RNA 333	RNA Gene	13	GC13P084140	1.910771	https://www.genecards.org/cgi-bin/carddisp.pl?gene=LINC00333
NMBR	Neuromedin B Receptor	Protein Coding	43	GC06M142059	1.907632	https://www.genecards.org/cgi-bin/carddisp.pl?gene=NMBR
LINC01818	Long Intergenic Non-Protein Coding RNA 1818	RNA Gene	8	GC02P150170	1.904036	https://www.genecards.org/cgi-bin/carddisp.pl?gene=LINC01818
lnc-TRIB1-8		RNA Gene	3	GC08P125497	1.904036	https://www.genecards.org/cgi-bin/carddisp.pl?gene=lnc-TRIB1-8
piR-37045		RNA Gene	2	GC22P044002	1.904036	https://www.genecards.org/cgi-bin/carddisp.pl?gene=piR-37045
HSALNG0135823		RNA Gene	2	GC22P043995	1.904036	https://www.genecards.org/cgi-bin/carddisp.pl?gene=HSALNG0135823
piR-39858-368		RNA Gene	2	GC20M016549	1.904036	https://www.genecards.org/cgi-bin/carddisp.pl?gene=piR-39858-368
TUBA4B	Tubulin Alpha 4b	Protein Coding	25	GC02P219253	1.902668	https://www.genecards.org/cgi-bin/carddisp.pl?gene=TUBA4B
LOC109279247	FGF21/FUT1 Promoter Region	Biological Region	1	GC19P048783	1.896202	https://www.genecards.org/cgi-bin/carddisp.pl?gene=LOC109279247
LINC02789	Long Intergenic Non-Protein Coding RNA 2789	RNA Gene	8	GC01P199149	1.891777	https://www.genecards.org/cgi-bin/carddisp.pl?gene=LINC02789

SCGB3A1	Secretoglobin Family 3A Member 1	Protein Coding	36	GC05M180590	1.888189	https://www.genecards.org/cgi-bin/carddisp.pl?gene=SCGB3A1
H2BC3	H2B Clustered Histone 3	Protein Coding	29	GC06M026044	1.885311	https://www.genecards.org/cgi-bin/carddisp.pl?gene=H2BC3
HCAR3	Hydroxycarboxylic Acid Receptor 3	Protein Coding	37	GC12M122714	1.885262	https://www.genecards.org/cgi-bin/carddisp.pl?gene=HCAR3
EGOT	Eosinophil Granule Ontogeny Transcript	RNA Gene	20	GC03M004790	1.88261	https://www.genecards.org/cgi-bin/carddisp.pl?gene=EGOT
BHLHE41	Basic Helix-Loop-Helix Family Member E41	Protein Coding	41	GC12M026120	1.879044	https://www.genecards.org/cgi-bin/carddisp.pl?gene=BHLHE41
H2AC13	H2A Clustered Histone 13	Protein Coding	30	GC06P055045	1.877702	https://www.genecards.org/cgi-bin/carddisp.pl?gene=H2AC13
CYP17A1-AS1	CYP17A1 Antisense RNA 1	RNA Gene	13	GC10P102747	1.877188	https://www.genecards.org/cgi-bin/carddisp.pl?gene=CYP17A1-AS1
PHLPP2	PH Domain And Leucine Rich Repeat Protein Phosphatase 2	Protein Coding	41	GC16M071637	1.875508	https://www.genecards.org/cgi-bin/carddisp.pl?gene=PHLPP2
FAM166B	Family With Sequence Similarity 166 Member B	Protein Coding	30	GC09M035554	1.871372	https://www.genecards.org/cgi-bin/carddisp.pl?gene=FAM166B
NBAT1	Neuroblastoma Associated Transcript 1	RNA Gene	15	GC06M022137	1.871232	https://www.genecards.org/cgi-bin/carddisp.pl?gene=NBAT1
KDM1B	Lysine Demethylase 1B	Protein Coding	37	GC06P018156	1.867278	https://www.genecards.org/cgi-bin/carddisp.pl?gene=KDM1B
LINC00339	Long Intergenic Non-Protein Coding RNA 339	RNA Gene	19	GC01P022111	1.862254	https://www.genecards.org/cgi-bin/carddisp.pl?gene=LINC00339
ENSG00000248537		RNA Gene	7	GC05P009363	1.855609	https://www.genecards.org/cgi-bin/carddisp.pl?gene=ENSG00000248537
ENSG00000250001		RNA Gene	7	GC05M009519	1.855609	https://www.genecards.org/cgi-bin/carddisp.pl?gene=ENSG00000250001
HNRNPA1P58	Heterogeneous Nuclear Ribonucleoprotein A1 Pseudogene 58	Pseudogene	4	GC06P024003	1.855609	https://www.genecards.org/cgi-bin/carddisp.pl?gene=HNRNPA1P58
LOC101060035	DnaJ Homolog Subfamily C Member 24 Pseudogene	Pseudogene	4	GC05M133720	1.855609	https://www.genecards.org/cgi-bin/carddisp.pl?gene=LOC101060035

HSALNG0040068		RNA Gene	4	GC05M009421	1.855609	https://www.genecards.org/cgi-bin/carddisp.pl?gene=HSALNG0040068
lnc-PPP1CC-3		RNA Gene	4	GC12M110993	1.855609	https://www.genecards.org/cgi-bin/carddisp.pl?gene=lnc-PPP1CC-3
SLC16A7	Solute Carrier Family 16 Member 7	Protein Coding	44	GC12P059596	1.855354	https://www.genecards.org/cgi-bin/carddisp.pl?gene=SLC16A7
MOSPD2	Motile Sperm Domain Containing 2	Protein Coding	34	GC0XP014891	1.850319	https://www.genecards.org/cgi-bin/carddisp.pl?gene=MOSPD2
LINC00888	Long Intergenic Non-Protein Coding RNA 888	RNA Gene	13	GC03P183447	1.849543	https://www.genecards.org/cgi-bin/carddisp.pl?gene=LINC00888
MCTS1	MCTS1 Re-Initiation And Release Factor	Protein Coding	36	GC0XP120594	1.84765	https://www.genecards.org/cgi-bin/carddisp.pl?gene=MCTS1
H2AC8	H2A Clustered Histone 8	Protein Coding	27	GC06P055580	1.845765	https://www.genecards.org/cgi-bin/carddisp.pl?gene=H2AC8
LINC01090	Long Intergenic Non-Protein Coding RNA 1090	RNA Gene	13	GC02M187712	1.843315	https://www.genecards.org/cgi-bin/carddisp.pl?gene=LINC01090
ENSG00000274121		RNA Gene	5	GC01P231814	1.843315	https://www.genecards.org/cgi-bin/carddisp.pl?gene=ENSG00000274121
DNAJC10	DnaJ Heat Shock Protein Family (Hsp40) Member C10	Protein Coding	38	GC02P182716	1.831709	https://www.genecards.org/cgi-bin/carddisp.pl?gene=DNAJC10
LINC00901	Long Intergenic Non-Protein Coding RNA 901	RNA Gene	15	GC03P116911	1.829658	https://www.genecards.org/cgi-bin/carddisp.pl?gene=LINC00901
C5orf67	Chromosome 5 Putative Open Reading Frame 67	RNA Gene	18	GC05M056511	1.827172	https://www.genecards.org/cgi-bin/carddisp.pl?gene=C5orf67
APOL5	Apolipoprotein L5	Protein Coding	34	GC22P035713	1.822159	https://www.genecards.org/cgi-bin/carddisp.pl?gene=APOL5
HMGN2	High Mobility Group Nucleosomal Binding Domain 2	Protein Coding	39	GC01P026473	1.813986	https://www.genecards.org/cgi-bin/carddisp.pl?gene=HMGN2
WSB1	WD Repeat And SOCS Box Containing 1	Protein Coding	40	GC17P027294	1.796367	https://www.genecards.org/cgi-bin/carddisp.pl?gene=WSB1
MIR378C	MicroRNA 378c	RNA Gene	13	GC10M130962	1.79261	https://www.genecards.org/cgi-bin/carddisp.pl?gene=MIR378C

SLC23A4P	Solute Carrier Family 23 Member 4, Pseudogene	Pseudogene	7	GC07M135247	1.792313	https://www.genecards.org/cgi-bin/carddisp.pl?gene=SLC23A4P
LINC00511	Long Intergenic Non-Protein Coding RNA 511	RNA Gene	17	GC17M072323	1.789853	https://www.genecards.org/cgi-bin/carddisp.pl?gene=LINC00511
MIR4772	MicroRNA 4772	RNA Gene	13	GC02P102432	1.777629	https://www.genecards.org/cgi-bin/carddisp.pl?gene=MIR4772
LINC01234	Long Intergenic Non-Protein Coding RNA 1234	RNA Gene	15	GC12M113584	1.768183	https://www.genecards.org/cgi-bin/carddisp.pl?gene=LINC01234
DGKD	Diacylglycerol Kinase Delta	Protein Coding	44	GC02P233356	1.764671	https://www.genecards.org/cgi-bin/carddisp.pl?gene=DGKD
LINC00993	Long Intergenic Non-Protein Coding RNA 993	RNA Gene	13	GC10P037249	1.76328	https://www.genecards.org/cgi-bin/carddisp.pl?gene=LINC00993
MTRNR2L6	MT-RNR2 Like 6	Protein Coding	19	GC07P145773	1.756919	https://www.genecards.org/cgi-bin/carddisp.pl?gene=MTRNR2L6
PDP2	Pyruvate Dehyrogenase Phosphatase Catalytic Subunit 2	Protein Coding	39	GC16P066881	1.752393	https://www.genecards.org/cgi-bin/carddisp.pl?gene=PDP2
ENSG00000266602		RNA Gene	7	GC18P001509	1.750862	https://www.genecards.org/cgi-bin/carddisp.pl?gene=ENSG00000266602
H2AW	H2A.W Histone	Protein Coding	29	GC01M228478	1.747616	https://www.genecards.org/cgi-bin/carddisp.pl?gene=H2AW
CPB2-AS1	CPB2 Antisense RNA 1	RNA Gene	14	GC13P046052	1.741461	https://www.genecards.org/cgi-bin/carddisp.pl?gene=CPB2-AS1
LINC01029	Long Intergenic Non-Protein Coding RNA 1029	RNA Gene	12	GC18M077972	1.741461	https://www.genecards.org/cgi-bin/carddisp.pl?gene=LINC01029
HDHD5-AS1	HDHD5 Antisense RNA 1	RNA Gene	12	GC22P017160	1.741461	https://www.genecards.org/cgi-bin/carddisp.pl?gene=HDHD5-AS1
LINC02534	Long Intergenic Non-Protein Coding RNA 2534	RNA Gene	9	GC06M115635	1.741461	https://www.genecards.org/cgi-bin/carddisp.pl?gene=LINC02534
LINC02392	Long Intergenic Non-Protein Coding RNA 2392	RNA Gene	9	GC12P090282	1.741461	https://www.genecards.org/cgi-bin/carddisp.pl?gene=LINC02392

SNORA63E	Small Nucleolar RNA, H/ACA Box 63E	RNA Gene	9	GC03P183457	1.741461	https://www.genecards.org/cgi-bin/carddisp.pl?gene=SNORA63E
SNORA63D	Small Nucleolar RNA, H/ACA Box 63D	RNA Gene	8	GC03P183470	1.741461	https://www.genecards.org/cgi-bin/carddisp.pl?gene=SNORA63D
ENSG00000243273		RNA Gene	8	GC03P150764	1.741461	https://www.genecards.org/cgi-bin/carddisp.pl?gene=ENSG00000243273
ENSG00000251152		RNA Gene	7	GC04P014575	1.741461	https://www.genecards.org/cgi-bin/carddisp.pl?gene=ENSG00000251152
ENSG00000249664		RNA Gene	7	GC05M083012	1.741461	https://www.genecards.org/cgi-bin/carddisp.pl?gene=ENSG00000249664
ENSG00000251186		RNA Gene	7	GC04M008455	1.741461	https://www.genecards.org/cgi-bin/carddisp.pl?gene=ENSG00000251186
ENSG00000260234		Protein Coding	7	GC03M150882	1.741461	https://www.genecards.org/cgi-bin/carddisp.pl?gene=ENSG00000260234
LINC02822	Long Intergenic Non-Protein Coding RNA 2822	RNA Gene	6	GC12P090411	1.741461	https://www.genecards.org/cgi-bin/carddisp.pl?gene=LINC02822
ENSG00000252040		RNA Gene	6	GC16M068630	1.741461	https://www.genecards.org/cgi-bin/carddisp.pl?gene=ENSG00000252040
ENSG00000253092		RNA Gene	6	GC03P183452	1.741461	https://www.genecards.org/cgi-bin/carddisp.pl?gene=ENSG00000253092
ENSG00000253354		RNA Gene	6	GC08P040370	1.741461	https://www.genecards.org/cgi-bin/carddisp.pl?gene=ENSG00000253354
ENSG00000272479		RNA Gene	6	GC08M040519	1.741461	https://www.genecards.org/cgi-bin/carddisp.pl?gene=ENSG00000272479
ENSG00000234481		RNA Gene	6	GC01P036773	1.741461	https://www.genecards.org/cgi-bin/carddisp.pl?gene=ENSG00000234481
ENSG00000241679		RNA Gene	6	GC03P143123	1.741461	https://www.genecards.org/cgi-bin/carddisp.pl?gene=ENSG00000241679
RPL35AP33	Ribosomal Protein L35a Pseudogene 33	Pseudogene	6	GC16M068489	1.741461	https://www.genecards.org/cgi-bin/carddisp.pl?gene=RPL35AP33
RNU6-655P	RNA, U6 Small Nuclear 655, Pseudogene	Pseudogene	6	GC18P078036	1.741461	https://www.genecards.org/cgi-bin/carddisp.pl?gene=RNU6-655P
LGMNP1	Legumain Pseudogene 1	Pseudogene	6	GC13M064957	1.741461	https://www.genecards.org/cgi-bin/carddisp.pl?gene=LGMNP1
ENSG00000248112		RNA Gene	6	GC05M082907	1.741461	https://www.genecards.org/cgi-bin/carddisp.pl?gene=ENSG00000248112

HNRNPA3P5	Heterogeneous Nuclear Ribonucleoprotein A3 Pseudogene 5	Pseudogene	5	GC13M065787	1.741461	https://www.genecards.org/cgi-bin/carddisp.pl?gene=HNRNPA3P5
ENSG00000259080		RNA Gene	5	GC02P027583	1.741461	https://www.genecards.org/cgi-bin/carddisp.pl?gene=ENSG00000259080
ENSG00000254171		RNA Gene	5	GC05P166128	1.741461	https://www.genecards.org/cgi-bin/carddisp.pl?gene=ENSG00000254171
ENSG00000251730		RNA Gene	5	GC03P183454	1.741461	https://www.genecards.org/cgi-bin/carddisp.pl?gene=ENSG00000251730
ENSG00000197462		RNA Gene	5	GC07P125917	1.741461	https://www.genecards.org/cgi-bin/carddisp.pl?gene=ENSG00000197462
ENSG00000234034		RNA Gene	5	GC21M022500	1.741461	https://www.genecards.org/cgi-bin/carddisp.pl?gene=ENSG00000234034
ENSG00000235503		RNA Gene	5	GC07P083355	1.741461	https://www.genecards.org/cgi-bin/carddisp.pl?gene=ENSG00000235503
LOC107987158	Uncharacterized LOC107987158	Protein Coding	5	GC11M003142	1.741461	https://www.genecards.org/cgi-bin/carddisp.pl?gene=LOC107987158
MRPL45P1	Mitochondrial Ribosomal Protein L45 Pseudogene 1	Pseudogene	5	GC02M088428	1.741461	https://www.genecards.org/cgi-bin/carddisp.pl?gene=MRPL45P1
RPL23AP20	Ribosomal Protein L23a Pseudogene 20	Pseudogene	5	GC01P241916	1.741461	https://www.genecards.org/cgi-bin/carddisp.pl?gene=RPL23AP20
RNU6-1172P	RNA, U6 Small Nuclear 1172, Pseudogene	Pseudogene	5	GC08M096431	1.741461	https://www.genecards.org/cgi-bin/carddisp.pl?gene=RNU6-1172P
lnc-PNPLA5-3		RNA Gene	5	GC22M048943	1.741461	https://www.genecards.org/cgi-bin/carddisp.pl?gene=lnc-PNPLA5-3
ENSG00000271682		Pseudogene	4	GC03M183462	1.741461	https://www.genecards.org/cgi-bin/carddisp.pl?gene=ENSG00000271682
ENSG00000233983		Pseudogene	4	GC01P099464	1.741461	https://www.genecards.org/cgi-bin/carddisp.pl?gene=ENSG00000233983
lnc-TSNAX-2		RNA Gene	4	GC01P231693	1.741461	https://www.genecards.org/cgi-bin/carddisp.pl?gene=lnc-TSNAX-2
SDR42E1P5	Short Chain Dehydrogenase/Reductase Family 42E, Member 1 Pseudogene 5	Pseudogene	4	GC02P102412	1.741461	https://www.genecards.org/cgi-bin/carddisp.pl?gene=SDR42E1P5
ENSG00000288092		RNA Gene	4	GC12P103013	1.741461	https://www.genecards.org/cgi-bin/carddisp.pl?gene=ENSG00000288092

lnc-FAM122A-1	RNA Gene	4	GC09P068711	1.741461	https://www.genecards.org/cgi-bin/carddisp.pl?gene=lnc-FAM122A-1
lnc-MRGPRG-1	RNA Gene	4	GC11M003146	1.741461	https://www.genecards.org/cgi-bin/carddisp.pl?gene=lnc-MRGPRG-1
lnc-RHOB-11	RNA Gene	4	GC02P020182	1.741461	https://www.genecards.org/cgi-bin/carddisp.pl?gene=lnc-RHOB-11
lnc-SLC46A3-1	RNA Gene	4	GC13M028701	1.741461	https://www.genecards.org/cgi-bin/carddisp.pl?gene=lnc-SLC46A3-1
lnc-WDR91-4	RNA Gene	4	GC07M135225	1.741461	https://www.genecards.org/cgi-bin/carddisp.pl?gene=lnc-WDR91-4
HSALNG0071703	RNA Gene	4	GC09M068727	1.741461	https://www.genecards.org/cgi-bin/carddisp.pl?gene=HSALNG0071703
lnc-MYL2-4	RNA Gene	4	GC12M110918	1.741461	https://www.genecards.org/cgi-bin/carddisp.pl?gene=lnc-MYL2-4
MN309223	RNA Gene	4	GC11P003129	1.741461	https://www.genecards.org/cgi-bin/carddisp.pl?gene=MN309223
ENSG00000275383	RNA Gene	3	GC16P068591	1.741461	https://www.genecards.org/cgi-bin/carddisp.pl?gene=ENSG00000275383
ENSG00000225906	Pseudogene	3	GC21P022882	1.741461	https://www.genecards.org/cgi-bin/carddisp.pl?gene=ENSG00000225906
ENSG00000231984	Pseudogene	3	GC01P199753	1.741461	https://www.genecards.org/cgi-bin/carddisp.pl?gene=ENSG00000231984
HSALNG0017419	RNA Gene	3	GC02P102419	1.741461	https://www.genecards.org/cgi-bin/carddisp.pl?gene=HSALNG0017419
ENSG00000287720	RNA Gene	3	GC03P003918	1.741461	https://www.genecards.org/cgi-bin/carddisp.pl?gene=ENSG00000287720
lnc-BICD1-6	RNA Gene	3	GC12P031995	1.741461	https://www.genecards.org/cgi-bin/carddisp.pl?gene=lnc-BICD1-6
HSALNG0023242	RNA Gene	3	GC02M235672	1.741461	https://www.genecards.org/cgi-bin/carddisp.pl?gene=HSALNG0023242
HSALNG0113292	RNA Gene	3	GC16P086387	1.741461	https://www.genecards.org/cgi-bin/carddisp.pl?gene=HSALNG0113292
HSALNG0113293	RNA Gene	3	GC16M086387	1.741461	https://www.genecards.org/cgi-bin/carddisp.pl?gene=HSALNG0113293
NONHSAG013110.2	RNA Gene	3	GC13P028704	1.741461	https://www.genecards.org/cgi-bin/carddisp.pl?gene=NONHSAG013110.2
lnc-SLC22A18-7	RNA Gene	3	GC11P003146	1.741461	https://www.genecards.org/cgi-bin/carddisp.pl?gene=lnc-SLC22A18-7

HSALNG0071704		RNA Gene	3	GC09M068734	1.741461	https://www.genecards.org/cgi-bin/carddisp.pl?gene=HSALNG0071704
hsa-miR-5096-076		RNA Gene	3	GC07M004224	1.741461	https://www.genecards.org/cgi-bin/carddisp.pl?gene=hsa-miR-5096-076
lnc-KLHL24-6		RNA Gene	3	GC03P183484	1.741461	https://www.genecards.org/cgi-bin/carddisp.pl?gene=lnc-KLHL24-6
piR-50443-100		RNA Gene	3	GC13P046157	1.741461	https://www.genecards.org/cgi-bin/carddisp.pl?gene=piR-50443-100
piR-61101-044		RNA Gene	3	GC10M015274	1.741461	https://www.genecards.org/cgi-bin/carddisp.pl?gene=piR-61101-044
ENSG00000280306		Uncategorized	2	GC16P020487	1.741461	https://www.genecards.org/cgi-bin/carddisp.pl?gene=ENSG00000280306
ENSG00000285529		RNA Gene	2	GC10P130525	1.741461	https://www.genecards.org/cgi-bin/carddisp.pl?gene=ENSG00000285529
ENSG00000258506		Pseudogene	2	GC14P084951	1.741461	https://www.genecards.org/cgi-bin/carddisp.pl?gene=ENSG00000258506
ENSG00000271078		Pseudogene	2	GC15M032251	1.741461	https://www.genecards.org/cgi-bin/carddisp.pl?gene=ENSG00000271078
HSALNG0011461		RNA Gene	2	GC01M231809	1.741461	https://www.genecards.org/cgi-bin/carddisp.pl?gene=HSALNG0011461
LOC105376433	Uncharacterized LOC105376433	RNA Gene	2	GC10P015236	1.741461	https://www.genecards.org/cgi-bin/carddisp.pl?gene=LOC105376433
LOC107986000	Uncharacterized LOC107986000	RNA Gene	2	GC02P235730	1.741461	https://www.genecards.org/cgi-bin/carddisp.pl?gene=LOC107986000
lnc-RASGRP1-1		RNA Gene	2	GC15M038754	1.741461	https://www.genecards.org/cgi-bin/carddisp.pl?gene=lnc-RASGRP1-1
HSALNG0032698		RNA Gene	2	GC04P007752	1.741461	https://www.genecards.org/cgi-bin/carddisp.pl?gene=HSALNG0032698
HSALNG0043115		RNA Gene	2	GC05M082908	1.741461	https://www.genecards.org/cgi-bin/carddisp.pl?gene=HSALNG0043115
HSALNG0043127		RNA Gene	2	GC05P083011	1.741461	https://www.genecards.org/cgi-bin/carddisp.pl?gene=HSALNG0043127
HSALNG0014027		RNA Gene	2	GC02M031425	1.741461	https://www.genecards.org/cgi-bin/carddisp.pl?gene=HSALNG0014027
HSALNG0031159		RNA Gene	2	GC03P183480	1.741461	https://www.genecards.org/cgi-bin/carddisp.pl?gene=HSALNG0031159
HSALNG0032696		RNA Gene	2	GC04M007742	1.741461	https://www.genecards.org/cgi-bin/carddisp.pl?gene=HSALNG0032696

HSALNG0056551		RNA Gene	2	GC07M021497	1.741461	https://www.genecards.org/cgi-bin/carddisp.pl?gene=HSALNG0056551
HSALNG0061543		RNA Gene	2	GC07P135291	1.741461	https://www.genecards.org/cgi-bin/carddisp.pl?gene=HSALNG0061543
NONHSAG018421.2		RNA Gene	2	GC16P005964	1.741461	https://www.genecards.org/cgi-bin/carddisp.pl?gene=NONHSAG018421.2
piR-43105-110		RNA Gene	2	GC12P031998	1.741461	https://www.genecards.org/cgi-bin/carddisp.pl?gene=piR-43105-110
piR-48799-012		RNA Gene	2	GC10P015269	1.741461	https://www.genecards.org/cgi-bin/carddisp.pl?gene=piR-48799-012
RF00017-1289		RNA Gene	2	GC13P046130	1.741461	https://www.genecards.org/cgi-bin/carddisp.pl?gene=RF00017-1289
piR-55036-040		RNA Gene	2	GC02P031489	1.741461	https://www.genecards.org/cgi-bin/carddisp.pl?gene=piR-55036-040
piR-31534-137		RNA Gene	2	GC13M028707	1.741461	https://www.genecards.org/cgi-bin/carddisp.pl?gene=piR-31534-137
piR-43106-189		RNA Gene	2	GC07P135282	1.741461	https://www.genecards.org/cgi-bin/carddisp.pl?gene=piR-43106-189
LOC107984123	Uncharacterized LOC107984123	RNA Gene	1	GC07M135264	1.741461	https://www.genecards.org/cgi-bin/carddisp.pl?gene=LOC107984123
LOC105377705	Uncharacterized LOC105377705	RNA Gene	1	GC05M166230	1.741461	https://www.genecards.org/cgi-bin/carddisp.pl?gene=LOC105377705
ENSG00000219736		Pseudogene	1	GC06M074850	1.741461	https://www.genecards.org/cgi-bin/carddisp.pl?gene=ENSG00000219736
ENSG00000229119		Pseudogene	1	GC05P166383	1.741461	https://www.genecards.org/cgi-bin/carddisp.pl?gene=ENSG00000229119
ENSG00000220748		Pseudogene	1	GC06P023972	1.741461	https://www.genecards.org/cgi-bin/carddisp.pl?gene=ENSG00000220748
LOC102724253	Uncharacterized LOC102724253	RNA Gene	1	GC15M038755	1.741461	https://www.genecards.org/cgi-bin/carddisp.pl?gene=LOC102724253
RF00026-272		RNA Gene	1	GC13M028705	1.741461	https://www.genecards.org/cgi-bin/carddisp.pl?gene=RF00026-272
HSALNG0105127-003		RNA Gene	1	GC15P038755	1.741461	https://www.genecards.org/cgi-bin/carddisp.pl?gene=HSALNG0105127-003
CR1L	Complement C3b/C4b Receptor 1 Like	Protein Coding	34	GC01P207646	1.733071	https://www.genecards.org/cgi-bin/carddisp.pl?gene=CR1L
ST8SIA6-AS1	ST8SIA6 Antisense RNA 1	RNA Gene	15	GC10P017386	1.730435	https://www.genecards.org/cgi-bin/carddisp.pl?gene=ST8SIA6-AS1

LANCL2	LanC Like 2	Protein Coding	36	GC07P055365	1.726853	https://www.genecards.org/cgi-bin/carddisp.pl?gene=LANCL2
SAP18	Sin3A Associated Protein 18	Protein Coding	40	GC13P021140	1.722666	https://www.genecards.org/cgi-bin/carddisp.pl?gene=SAP18
IGKV2D-23	Immunoglobulin Kappa Variable 2D-23 (Pseudogene)	Pseudogene	5	GC02P090720	1.71927	https://www.genecards.org/cgi-bin/carddisp.pl?gene=IGKV2D-23
LINC01116	Long Intergenic Non-Protein Coding RNA 1116	RNA Gene	16	GC02M176629	1.709733	https://www.genecards.org/cgi-bin/carddisp.pl?gene=LINC01116
UNC5B	Unc-5 Netrin Receptor B	Protein Coding	38	GC10P071212	1.704455	https://www.genecards.org/cgi-bin/carddisp.pl?gene=UNC5B
GRWD1	Glutamate Rich WD Repeat Containing 1	Protein Coding	33	GC19P048445	1.700534	https://www.genecards.org/cgi-bin/carddisp.pl?gene=GRWD1
ERVK-6	Endogenous Retrovirus Group K Member 6, Envelope	Protein Coding	16	GC07U903184	1.691116	https://www.genecards.org/cgi-bin/carddisp.pl?gene=ERVK-6
SRRM3	Serine/Arginine Repetitive Matrix 3	Protein Coding	30	GC07P076201	1.686083	https://www.genecards.org/cgi-bin/carddisp.pl?gene=SRRM3
H2BU1	H2B.U Histone 1	Protein Coding	25	GC01P228467	1.678734	https://www.genecards.org/cgi-bin/carddisp.pl?gene=H2BU1
LINC00052	Long Intergenic Non-Protein Coding RNA 52	RNA Gene	19	GC15P087576	1.677874	https://www.genecards.org/cgi-bin/carddisp.pl?gene=LINC00052
MIR613	MicroRNA 613	RNA Gene	13	GC12P012783	1.670024	https://www.genecards.org/cgi-bin/carddisp.pl?gene=MIR613
H2AC14	H2A Clustered Histone 14	Protein Coding	27	GC06M046627	1.669144	https://www.genecards.org/cgi-bin/carddisp.pl?gene=H2AC14
H2BC4	H2B Clustered Histone 4	Protein Coding	30	GC06M047212	1.666755	https://www.genecards.org/cgi-bin/carddisp.pl?gene=H2BC4
ZNF436	Zinc Finger Protein 436	Protein Coding	40	GC01M023359	1.659083	https://www.genecards.org/cgi-bin/carddisp.pl?gene=ZNF436
KLF17	Kruppel Like Factor 17	Protein Coding	38	GC01P044048	1.657151	https://www.genecards.org/cgi-bin/carddisp.pl?gene=KLF17
IPP	Intracisternal A Particle-Promoted Polypeptide	Protein Coding	36	GC01M045695	1.656377	https://www.genecards.org/cgi-bin/carddisp.pl?gene=IPP
HAT1	Histone Acetyltransferase 1	Protein Coding	44	GC02P171922	1.648816	https://www.genecards.org/cgi-bin/carddisp.pl?gene=HAT1
H2BC14	H2B Clustered Histone 14	Protein Coding	27	GC06P055046	1.645323	https://www.genecards.org/cgi-bin/carddisp.pl?gene=H2BC14
RMST	Rhabdomyosarcoma 2 Associated Transcript	RNA Gene	21	GC12P097431	1.630729	https://www.genecards.org/cgi-bin/carddisp.pl?gene=RMST

GPR12	G Protein-Coupled Receptor 12	Protein Coding	38	GC13M026755	1.630275	https://www.genecards.org/cgi-bin/carddisp.pl?gene=GPR12
SUCNR1	Succinate Receptor 1	Protein Coding	41	GC03P151873	1.623741	https://www.genecards.org/cgi-bin/carddisp.pl?gene=SUCNR1
LINC01089	Long Intergenic Non-Protein Coding RNA 1089	RNA Gene	15	GC12M121796	1.623714	https://www.genecards.org/cgi-bin/carddisp.pl?gene=LINC01089
HRK	Harakiri, BCL2 Interacting Protein	Protein Coding	39	GC12M116820	1.623511	https://www.genecards.org/cgi-bin/carddisp.pl?gene=HRK
PBOV1	Prostate And Breast Cancer Overexpressed 1	Protein Coding	27	GC06M138215	1.621172	https://www.genecards.org/cgi-bin/carddisp.pl?gene=PBOV1
KLF3-AS1	KLF3 Antisense RNA 1	RNA Gene	16	GC04M038594	1.620704	https://www.genecards.org/cgi-bin/carddisp.pl?gene=KLF3-AS1
H2BC17	H2B Clustered Histone 17	Protein Coding	26	GC06P055057	1.613958	https://www.genecards.org/cgi-bin/carddisp.pl?gene=H2BC17
LINC00574	Long Intergenic Non-Protein Coding RNA 574	RNA Gene	19	GC06P169790	1.613252	https://www.genecards.org/cgi-bin/carddisp.pl?gene=LINC00574
PDCD4-AS1	PDCD4 Antisense RNA 1	RNA Gene	16	GC10M110868	1.608485	https://www.genecards.org/cgi-bin/carddisp.pl?gene=PDCD4-AS1
TRERF1	Transcriptional Regulating Factor 1	Protein Coding	38	GC06M042224	1.598639	https://www.genecards.org/cgi-bin/carddisp.pl?gene=TRERF1
NMRAL2P	NmrA Like Redox Sensor 2, Pseudogene	Pseudogene	9	GC03P185961	1.582165	https://www.genecards.org/cgi-bin/carddisp.pl?gene=NMRAL2P
ZNF667-AS1	ZNF667 Antisense RNA 1 (Head To Head)	RNA Gene	15	GC19P056477	1.573915	https://www.genecards.org/cgi-bin/carddisp.pl?gene=ZNF667-AS1
KIAA0100	KIAA0100	Protein Coding	34	GC17M028614	1.54563	https://www.genecards.org/cgi-bin/carddisp.pl?gene=KIAA0100
MIR103B2	MicroRNA 103b-2	RNA Gene	9	GC20M003899	1.544202	https://www.genecards.org/cgi-bin/carddisp.pl?gene=MIR103B2
MIR103B1	MicroRNA 103b-1	RNA Gene	8	GC05P168560	1.544202	https://www.genecards.org/cgi-bin/carddisp.pl?gene=MIR103B1
HEATR6	HEAT Repeat Containing 6	Protein Coding	33	GC17M060041	1.539996	https://www.genecards.org/cgi-bin/carddisp.pl?gene=HEATR6
RGS16	Regulator Of G Protein Signaling 16	Protein Coding	40	GC01M182598	1.53539	https://www.genecards.org/cgi-bin/carddisp.pl?gene=RGS16
SLC16A3	Solute Carrier Family 16 Member 3	Protein Coding	45	GC17P082217	1.534593	https://www.genecards.org/cgi-bin/carddisp.pl?gene=SLC16A3
MED18	Mediator Complex Subunit 18	Protein Coding	36	GC01P028340	1.531515	https://www.genecards.org/cgi-bin/carddisp.pl?gene=MED18
H2AC11	H2A Clustered Histone 11	Protein Coding	31	GC06P055577	1.530112	https://www.genecards.org/cgi-bin/carddisp.pl?gene=H2AC11

MRS2	Magnesium Transporter MRS2	Protein Coding	38	GC06P024402	1.518554	https://www.genecards.org/cgi-bin/carddisp.pl?gene=MRS2
KCNK15-AS1	KCNK15 And WISP2 Antisense RNA 1	RNA Gene	13	GC20M044694	1.514382	https://www.genecards.org/cgi-bin/carddisp.pl?gene=KCNK15-AS1
PRSS50	Serine Protease 50	Protein Coding	34	GC03M046712	1.512659	https://www.genecards.org/cgi-bin/carddisp.pl?gene=PRSS50
KLK9	Kallikrein Related Peptidase 9	Protein Coding	33	GC19M051002	1.495976	https://www.genecards.org/cgi-bin/carddisp.pl?gene=KLK9
RHBDD2	Rhomboid Domain Containing 2	Protein Coding	36	GC07P075842	1.483984	https://www.genecards.org/cgi-bin/carddisp.pl?gene=RHBDD2
ETAA1	ETAA1 Activator Of ATR Kinase	Protein Coding	32	GC02P067397	1.482463	https://www.genecards.org/cgi-bin/carddisp.pl?gene=ETAA1
MIR2052HG	MIR2052 Host Gene	RNA Gene	12	GC08P074599	1.479439	https://www.genecards.org/cgi-bin/carddisp.pl?gene=MIR2052HG
PDR	Pigment Disorder, Reticulate	Genetic Locus	4	GC0XU990180	1.474738	https://www.genecards.org/cgi-bin/carddisp.pl?gene=PDR
ADARB2-AS1	ADARB2 Antisense RNA 1	RNA Gene	19	GC10P001526	1.473819	https://www.genecards.org/cgi-bin/carddisp.pl?gene=ADARB2-AS1
SLC24A2	Solute Carrier Family 24 Member 2	Protein Coding	40	GC09M019507	1.46725	https://www.genecards.org/cgi-bin/carddisp.pl?gene=SLC24A2
MIR570	MicroRNA 570	RNA Gene	16	GC03P195777	1.464161	https://www.genecards.org/cgi-bin/carddisp.pl?gene=MIR570
ERVH48-1	Endogenous Retrovirus Group 48 Member 1	Protein Coding	15	GC21M042916	1.456905	https://www.genecards.org/cgi-bin/carddisp.pl?gene=ERVH48-1
TRIM66	Tripartite Motif Containing 66	Protein Coding	33	GC11M008612	1.448823	https://www.genecards.org/cgi-bin/carddisp.pl?gene=TRIM66
LINP1	LncRNA In Non-Homologous End Joining Pathway 1	RNA Gene	11	GC10P006738	1.441921	https://www.genecards.org/cgi-bin/carddisp.pl?gene=LINP1
LINC00958	Long Intergenic Non-Protein Coding RNA 958	RNA Gene	16	GC11M012930	1.4415	https://www.genecards.org/cgi-bin/carddisp.pl?gene=LINC00958
DOC2B	Double C2 Domain Beta	Protein Coding	36	GC17M000142	1.43914	https://www.genecards.org/cgi-bin/carddisp.pl?gene=DOC2B
MTBP	MDM2 Binding Protein	Protein Coding	33	GC08P120426	1.429176	https://www.genecards.org/cgi-bin/carddisp.pl?gene=MTBP
FOXCUT	FOXC1 Upstream Transcript	RNA Gene	12	GC06P001604	1.426376	https://www.genecards.org/cgi-bin/carddisp.pl?gene=FOXCUT

HERC4	HECT And RLD Domain Containing E3 Ubiquitin Protein Ligase 4	Protein Coding	37	GC10M067921	1.419177	https://www.genecards.org/cgi-bin/carddisp.pl?gene=HERC4
H2BC6	H2B Clustered Histone 6	Protein Coding	27	GC06P055584	1.408377	https://www.genecards.org/cgi-bin/carddisp.pl?gene=H2BC6
H2BC15	H2B Clustered Histone 15	Protein Coding	28	GC06P055050	1.407558	https://www.genecards.org/cgi-bin/carddisp.pl?gene=H2BC15
ERN2	Endoplasmic Reticulum To Nucleus Signaling 2	Protein Coding	38	GC16M023690	1.393055	https://www.genecards.org/cgi-bin/carddisp.pl?gene=ERN2
BPIFA4P	BPI Fold Containing Family A Member 4, Pseudogene	Pseudogene	18	GC20P033193	1.382041	https://www.genecards.org/cgi-bin/carddisp.pl?gene=BPIFA4P
LINC01671	Long Intergenic Non-Protein Coding RNA 1671	RNA Gene	10	GC21M042579	1.37744	https://www.genecards.org/cgi-bin/carddisp.pl?gene=LINC01671
MANCR	Mitotically Associated Long Non Coding RNA	RNA Gene	12	GC10M004577	1.366042	https://www.genecards.org/cgi-bin/carddisp.pl?gene=MANCR
RAB13	RAB13, Member RAS Oncogene Family	Protein Coding	38	GC01M153981	1.351943	https://www.genecards.org/cgi-bin/carddisp.pl?gene=RAB13
DEDD2	Death Effector Domain Containing 2	Protein Coding	36	GC19M042198	1.338967	https://www.genecards.org/cgi-bin/carddisp.pl?gene=DEDD2
DSCAM-AS1	DSCAM Antisense RNA 1	RNA Gene	16	GC21P040383	1.332989	https://www.genecards.org/cgi-bin/carddisp.pl?gene=DSCAM-AS1
OR3A4P	Olfactory Receptor Family 3 Subfamily A Member 4 Pseudogene	Pseudogene	19	GC17P003365	1.322313	https://www.genecards.org/cgi-bin/carddisp.pl?gene=OR3A4P
LINC00628	Long Intergenic Non-Protein Coding RNA 628	RNA Gene	13	GC01M204375	1.322313	https://www.genecards.org/cgi-bin/carddisp.pl?gene=LINC00628
DHRS4L1	Dehydrogenase/Reductase 4 Like 1 (Pseudogene)	RNA Gene	27	GC14P026317	1.311215	https://www.genecards.org/cgi-bin/carddisp.pl?gene=DHRS4L1
CASC22	Cancer Susceptibility 22	RNA Gene	13	GC16P052258	1.299549	https://www.genecards.org/cgi-bin/carddisp.pl?gene=CASC22

SGPP2	Sphingosine-1-Phosphate Phosphatase 2	Protein Coding	36	GC02P222424	1.298772	https://www.genecards.org/cgi-bin/carddisp.pl?gene=SGPP2
SF3B5	Splicing Factor 3b Subunit 5	Protein Coding	35	GC06M144094	1.297816	https://www.genecards.org/cgi-bin/carddisp.pl?gene=SF3B5
FGF14-AS2	FGF14 Antisense RNA 2	RNA Gene	13	GC13P102394	1.287911	https://www.genecards.org/cgi-bin/carddisp.pl?gene=FGF14-AS2
IGKV@	Immunoglobulin Kappa Variable Cluster	Gene Cluster	8	GC02U990059	1.284459	https://www.genecards.org/cgi-bin/carddisp.pl?gene=IGKV%40
ELK4	ETS Transcription Factor ELK4	Protein Coding	37	GC01M205577	1.273318	https://www.genecards.org/cgi-bin/carddisp.pl?gene=ELK4
H2BC12	H2B Clustered Histone 12	Protein Coding	27	GC06M046553	1.256909	https://www.genecards.org/cgi-bin/carddisp.pl?gene=H2BC12
RASSF1-AS1	RASSF1 Antisense RNA 1	RNA Gene	12	GC03P050337	1.247685	https://www.genecards.org/cgi-bin/carddisp.pl?gene=RASSF1-AS1
STXBP5-AS1	STXBP5 Antisense RNA 1	RNA Gene	16	GC06M146841	1.236905	https://www.genecards.org/cgi-bin/carddisp.pl?gene=STXBP5-AS1
GAL3ST2	Galactose-3-O-Sulfotransferase 2	Protein Coding	34	GC02P241777	1.228368	https://www.genecards.org/cgi-bin/carddisp.pl?gene=GAL3ST2
LINC00160	Long Intergenic Non-Protein Coding RNA 160	RNA Gene	15	GC21M034723	1.21189	https://www.genecards.org/cgi-bin/carddisp.pl?gene=LINC00160
ENSG00000279217		RNA Gene	4	GC22P036071	1.179829	https://www.genecards.org/cgi-bin/carddisp.pl?gene=ENSG00000279217
LINC02099	Long Intergenic Non-Protein Coding RNA 2099	RNA Gene	11	GC08P029749	1.174197	https://www.genecards.org/cgi-bin/carddisp.pl?gene=LINC02099
PTPRG-AS1	PTPRG Antisense RNA 1	RNA Gene	15	GC03M062248	1.164506	https://www.genecards.org/cgi-bin/carddisp.pl?gene=PTPRG-AS1
H2BC8	H2B Clustered Histone 8	Protein Coding	27	GC06M047213	1.156155	https://www.genecards.org/cgi-bin/carddisp.pl?gene=H2BC8
FMNL3	Formin Like 3	Protein Coding	36	GC12M049636	1.154362	https://www.genecards.org/cgi-bin/carddisp.pl?gene=FMNL3
H2AC15	H2A Clustered Histone 15	Protein Coding	29	GC06M046630	1.150205	https://www.genecards.org/cgi-bin/carddisp.pl?gene=H2AC15
H2BC7	H2B Clustered Histone 7	Protein Coding	26	GC06P055585	1.13868	https://www.genecards.org/cgi-bin/carddisp.pl?gene=H2BC7
ENSG00000229282		RNA Gene	8	GC06P003038	1.138029	https://www.genecards.org/cgi-bin/carddisp.pl?gene=ENSG00000229282
H2AC19	H2A Clustered Histone 19	Protein Coding	22	GC01P149980	1.132637	https://www.genecards.org/cgi-bin/carddisp.pl?gene=H2AC19
H2BC10	H2B Clustered Histone 10	Protein Coding	27	GC06P055582	1.102045	https://www.genecards.org/cgi-bin/carddisp.pl?gene=H2BC10

LINC00636	Long Intergenic Non-Protein Coding RNA 636	RNA Gene	17	GC03P107883	1.101862	https://www.genecards.org/cgi-bin/carddisp.pl?gene=LINC00636
HMMR-AS1	HMMR Antisense RNA 1	RNA Gene	14	GC05M163482	1.101862	https://www.genecards.org/cgi-bin/carddisp.pl?gene=HMMR-AS1
JADRR	JADE1 Adjacent Regulatory RNA	RNA Gene	11	GC04P128768	1.101862	https://www.genecards.org/cgi-bin/carddisp.pl?gene=JADRR
LINC02130	Long Intergenic Non-Protein Coding RNA 2130	RNA Gene	9	GC16M014365	1.101862	https://www.genecards.org/cgi-bin/carddisp.pl?gene=LINC02130
ENSG00000235584		RNA Gene	6	GC02P095673	1.101862	https://www.genecards.org/cgi-bin/carddisp.pl?gene=ENSG00000235584
FAM174A	Family With Sequence Similarity 174 Member A	Protein Coding	32	GC05P100535	1.070906	https://www.genecards.org/cgi-bin/carddisp.pl?gene=FAM174A
RGPD4	RANBP2 Like And GRIP Domain Containing 4	Protein Coding	28	GC02P107826	1.061936	https://www.genecards.org/cgi-bin/carddisp.pl?gene=RGPD4
RNY5	RNA, Ro60-Associated Y5	RNA Gene	12	GC07P148941	0.764868	https://www.genecards.org/cgi-bin/carddisp.pl?gene=RNY5
MIR3666	MicroRNA 3666	RNA Gene	11	GC07P114653	0.753341	https://www.genecards.org/cgi-bin/carddisp.pl?gene=MIR3666
MIR4668	MicroRNA 4668	RNA Gene	12	GC09P111932	0.71616	https://www.genecards.org/cgi-bin/carddisp.pl?gene=MIR4668
OR2A4	Olfactory Receptor Family 2 Subfamily A Member 4	Protein Coding	33	GC06M131700	0.657361	https://www.genecards.org/cgi-bin/carddisp.pl?gene=OR2A4
TM6SF1	Transmembrane 6 Superfamily Member 1	Protein Coding	33	GC15P083357	0.475591	https://www.genecards.org/cgi-bin/carddisp.pl?gene=TM6SF1
