# Supplementary material for: The Study of Yin-Chen-Hao-Tang Preventing and Treating Alcoholic Fatty Liver Disease through PPAR Signaling Pathway Based on Network Pharmacology and RNA-Seq Transcriptomics
Source: Evid Based Complement Alternat Med. 2021 Dec 31;2021:8917993. doi: 10.1155/2021/8917993 (PMC8741355; doi:10.1155/2021/8917993)
Supplement: Supplementary Materials — Supplementary Material 1-1: Herb Target-Artemisiae Scopariae Herba. Supplementary Material 1-2: Herb Target-Gardeniae Fructus. Supplementary Material 1-3: Herb Target-Radix Rhei Et Rhizome. Supplementary Material 2: AFLD-GeneCards-SearchResults. Supplementary Material 3: string_interactions.tsv default node. [file 8917993.f1.zip › 8917993.f1/Supplementary material 3-string_interactions.tsv default node.pdf]

AverageShortestPathLength	BetweennessCentrality	ClosenessCentrality	ClusteringCoefficient	Degree	Eccentricity	IsSingleNode	name
1.401294498	0.090222052	0.713625866	0.2449543	187	3	FALSE	INS
1.436893204	0.053803878	0.695945946	0.271546	179	3	FALSE	AKT1
1.495145631	0.039357407	0.668831169	0.3040976	163	3	FALSE	IL6
1.501618123	0.045943614	0.665948276	0.2994565	161	3	FALSE	TP53
1.566343042	0.026984596	0.638429752	0.3587873	140	3	FALSE	CASP3
1.601941748	0.017053805	0.624242424	0.3918967	131	3	FALSE	JUN
1.601941748	0.023566407	0.624242424	0.3587358	130	3	FALSE	EGFR
1.605177994	0.020349754	0.622983871	0.3674419	130	3	FALSE	MYC
1.631067961	0.010806779	0.613095238	0.424072	127	3	FALSE	STAT3
1.634304207	0.018783076	0.611881188	0.3786667	126	3	FALSE	SRC
1.637540453	0.018563641	0.610671937	0.3910968	125	3	FALSE	MAPK1
1.624595469	0.012474778	0.615537849	0.4094194	125	3	FALSE	MAPK8
1.656957929	0.014291714	0.603515625	0.4275843	122	3	FALSE	MMP9
1.631067961	0.014919644	0.613095238	0.4232782	121	3	FALSE	PTGS2
1.647249191	0.015486062	0.607072692	0.4275744	119	3	FALSE	CXCL8
1.689320388	0.010496802	0.591954023	0.4417014	110	3	FALSE	IL1B
1.71197411	0.011397279	0.584120983	0.416234	108	3	FALSE	FOS
1.692556634	0.009592901	0.59082218	0.4182684	107	3	FALSE	ESR1
1.699029126	0.00717313	0.588571429	0.4699348	107	3	FALSE	TLR4
1.702265372	0.008210883	0.587452471	0.485348	105	3	FALSE	CCL2
1.72815534	0.005548035	0.578651685	0.460299	102	3	FALSE	CCND1
1.718446602	0.006103283	0.581920904	0.5119394	102	3	FALSE	IL10
1.731391586	0.017478127	0.577570093	0.3919373	95	3	FALSE	HSP90AA1
1.750809061	0.010926113	0.57116451	0.518925	95	3	FALSE	MMP2
1.737864078	0.011432143	0.575418994	0.3980782	94	3	FALSE	CREB1
1.734627832	0.009634772	0.576492537	0.426447	94	3	FALSE	PPARG
1.74433657	0.004500498	0.573283859	0.5430108	93	3	FALSE	ICAM1
1.750809061	0.00660551	0.57116451	0.5130902	93	3	FALSE	IL4
1.731391586	0.01065507	0.577570093	0.4124542	91	3	FALSE	NOS3
1.766990291	0.007846255	0.565934066	0.4893883	90	3	FALSE	RELA
1.760517799	0.003651266	0.568014706	0.5313358	90	3	FALSE	TLR2
1.773462783	0.003333269	0.563868613	0.5347293	89	3	FALSE	FGF2
1.757281553	0.007739484	0.569060773	0.5252809	89	3	FALSE	IL2
1.757281553	0.006485498	0.569060773	0.4555904	88	3	FALSE	ERBB2
1.776699029	0.004086081	0.56284153	0.5355277	88	3	FALSE	MAPK14
1.796116505	0.003289964	0.556756757	0.5421687	84	3	FALSE	BCL2L1
1.792880259	0.004774462	0.557761733	0.5126219	84	3	FALSE	STAT1
1.786407767	0.008290763	0.559782609	0.4655766	84	3	FALSE	EDN1
1.776699029	0.012364908	0.56284153	0.4360858	83	3	FALSE	AR
1.789644013	0.012095503	0.558770344	0.4084631	83	3	FALSE	BDNF
1.799352751	0.003634453	0.555755396	0.5700617	81	3	FALSE	VCAM1
1.796116505	0.00523462	0.556756757	0.5274691	81	3	FALSE	SERPINE1
1.796116505	0.00367629	0.556756757	0.4985394	79	3	FALSE	RHOA
1.783171521	0.006518453	0.560798548	0.5202856	79	3	FALSE	HMOX1
1.779935275	0.006792187	0.561818182	0.4826355	79	3	FALSE	CRP
1.812297735	0.003609757	0.551785714	0.5485232	79	3	FALSE	CASP8
1.825242718	0.001606452	0.54787234	0.6329825	76	3	FALSE	CSF2
1.815533981	0.001706526	0.550802139	0.6287719	76	3	FALSE	IFNG
1.815533981	0.008174407	0.550802139	0.4924102	74	3	FALSE	MPO
1.83171521	0.001741999	0.545936396	0.6000761	73	3	FALSE	PECAM1
1.838187702	0.003143652	0.544014085	0.5852359	73	3	FALSE	SPP1
1.854368932	0.003217171	0.539267016	0.5445205	73	3	FALSE	NFKB1
1.812297735	0.004160883	0.551785714	0.5199531	72	3	FALSE	HIF1A
1.838187702	0.001993966	0.544014085	0.5865303	69	3	FALSE	KDR
1.83171521	0.002028129	0.545936396	0.6666667	69	3	FALSE	TGFB1

1.854368932	0.003309075	0.539267016	0.5788576	69	3	FALSE	PLG
1.815533981	0.015143665	0.550802139	0.3410222	67	3	FALSE	F2
1.870550162	0.002089393	0.534602076	0.588345	66	3	FALSE	CDKN1A
1.828478964	0.006423614	0.546902655	0.4879808	65	3	FALSE	CAV1
1.851132686	0.002110557	0.54020979	0.63125	65	3	FALSE	CXCL10
1.84789644	0.003654722	0.541155867	0.4653846	65	3	FALSE	SP1
1.812297735	0.019922246	0.551785714	0.359127	64	3	FALSE	GPT
1.841423948	0.00474064	0.543057996	0.5248016	64	3	FALSE	NR3C1
1.883495146	0.002904276	0.530927835	0.6081349	64	3	FALSE	IL13
1.834951456	0.00495793	0.544973545	0.609319	63	3	FALSE	NOS2
1.844660194	0.00958012	0.542105263	0.3881544	62	3	FALSE	SOD1
1.886731392	0.001369886	0.530017153	0.7131148	61	3	FALSE	MMP1
1.8802589	0.001204998	0.531841652	0.6409836	61	4	FALSE	CASP9
1.893203883	0.001280446	0.528205128	0.6398907	61	3	FALSE	MCL1
1.896440129	9.34E-04	0.527303754	0.6553672	60	3	FALSE	NFKBIA
1.893203883	0.001845005	0.528205128	0.6881356	60	4	FALSE	MMP3
1.8802589	0.001684843	0.531841652	0.5464641	59	3	FALSE	PGR
1.902912621	7.68E-04	0.525510204	0.6890707	59	3	FALSE	CD40
1.906148867	8.92E-04	0.524617997	0.6885965	57	3	FALSE	SELE
1.909385113	0.001394123	0.523728814	0.7161654	57	4	FALSE	FASLG
1.899676375	6.94E-04	0.526405451	0.7067669	57	3	FALSE	CASP1
1.889967638	0.004849343	0.529109589	0.5474026	56	3	FALSE	AHR
1.919093851	9.43E-04	0.521079258	0.6967532	56	3	FALSE	CD40LG
1.925566343	0.001796257	0.519327731	0.5856045	54	3	FALSE	CDK4
1.922330097	0.002521218	0.52020202	0.5391872	53	4	FALSE	MAPK10
1.909385113	0.0018907	0.523728814	0.58418	53	3	FALSE	PLAU
1.925566343	0.00298942	0.519327731	0.3800905	52	3	FALSE	PRKCA
1.957928803	0.001970236	0.510743802	0.5356863	51	3	FALSE	CDK1
1.941747573	0.001865828	0.515	0.6282353	51	3	FALSE	SELP
1.941747573	0.003072595	0.515	0.5835294	51	3	FALSE	CDK2
1.944983819	9.67E-04	0.514143095	0.6745098	51	3	FALSE	IL1A
1.902912621	0.004941776	0.525510204	0.3771429	50	3	FALSE	SLC2A4
1.919093851	0.001507259	0.521079258	0.5281633	50	3	FALSE	GSK3B
1.902912621	0.002929183	0.525510204	0.3926531	50	3	FALSE	PPARA
1.893203883	0.006894912	0.528205128	0.355102	50	3	FALSE	GCG
1.993527508	0.001074316	0.501623377	0.6604082	50	4	FALSE	CXCL2
2	8.76E-04	0.5	0.6777211	49	4	FALSE	CCNB1
1.919093851	0.001026236	0.521079258	0.6386054	49	3	FALSE	PARP1
1.941747573	0.002770433	0.515	0.4051418	48	3	FALSE	LPL
1.964401294	0.001068123	0.509060956	0.6614246	47	3	FALSE	CCNA2
1.990291262	6.80E-04	0.502439024	0.719704	47	3	FALSE	CD86
1.98381877	0.002821353	0.504078303	0.4048309	46	4	FALSE	PRKCD
1.98381877	4.24E-04	0.504078303	0.7497585	46	4	FALSE	IRF1
1.906148867	0.001988091	0.524617997	0.6347826	46	3	FALSE	HSPB1
1.941747573	0.001123805	0.515	0.6151515	45	3	FALSE	IGF2
1.932038835	0.001414884	0.51758794	0.5739958	44	3	FALSE	NFE2L2
1.964401294	9.81E-04	0.509060956	0.6712474	44	3	FALSE	F3
1.990291262	7.07E-04	0.502439024	0.6479915	44	3	FALSE	RB1
2.009708738	3.38E-04	0.497584541	0.7399577	44	3	FALSE	CD80
2.042071197	0.001975464	0.489698891	0.4978858	44	4	FALSE	ITGB2
1.938511327	6.95E-04	0.515859766	0.7536998	44	3	FALSE	FLT1
1.912621359	0.00613243	0.52284264	0.4706534	43	3	FALSE	SLC2A1
1.957928803	0.001224455	0.510743802	0.5891473	43	3	FALSE	NCF1
1.964401294	0.002234614	0.509060956	0.4108527	43	3	FALSE	PRKCB
1.919093851	0.002327336	0.521079258	0.6046512	43	3	FALSE	HSPA5
1.961165049	0.003091618	0.50990099	0.4086379	43	3	FALSE	APOA1
1.922330097	0.006216303	0.52020202	0.2752613	42	3	FALSE	CYP3A4
1.96763754	6.81E-04	0.508223684	0.7351916	42	3	FALSE	RUNX2

1.964401294	0.001218395	0.509060956	0.6922184	42	3	FALSE	MUC1
1.98381877	0.002412874	0.504078303	0.6402439	41	4	FALSE	GJA1
1.977346278	2.86E-04	0.505728314	0.7012195	41	4	FALSE	IKBKB
1.944983819	0.00934966	0.514143095	0.3304878	41	4	FALSE	LDHA
1.993527508	0.001434995	0.501623377	0.4564103	40	4	FALSE	NCOA1
1.954692557	0.001094768	0.511589404	0.6153846	40	3	FALSE	IGFBP3
2.006472492	0.007084578	0.498387097	0.3076923	39	3	FALSE	GOT2
1.974110032	0.001607553	0.506557377	0.5533063	39	4	FALSE	CTSB
2	7.20E-04	0.5	0.608637	39	4	FALSE	BTK
1.964401294	0.002635623	0.509060956	0.5192034	38	3	FALSE	ABCG2
1.977346278	0.004031265	0.505728314	0.3997155	38	3	FALSE	OPRM1
1.925566343	0.002227707	0.519327731	0.5078236	38	3	FALSE	NQO1
1.974110032	0.001049911	0.506557377	0.5874822	38	4	FALSE	GFAP
1.98381877	5.31E-04	0.504078303	0.6216216	37	3	FALSE	PRKCZ
1.964401294	0.00251013	0.509060956	0.3857143	36	3	FALSE	NR1H4
1.977346278	4.66E-04	0.505728314	0.6809524	36	4	FALSE	ESR2
1.964401294	0.003605759	0.509060956	0.4269841	36	3	FALSE	FASN
1.957928803	0.004633545	0.510743802	0.3555556	36	3	FALSE	ACHE
2.097087379	3.07E-04	0.476851852	0.7412698	36	4	FALSE	CHEK1
1.987055016	0.003872153	0.503257329	0.3777778	36	3	FALSE	RXRA
2.009708738	0.002221285	0.497584541	0.3932773	35	4	FALSE	CYP1A1
2.145631068	5.15E-04	0.466063348	0.692437	35	4	FALSE	CXCL11
2.003236246	0.003149685	0.499192246	0.4352941	35	4	FALSE	CTSD
2.097087379	2.74E-04	0.476851852	0.7462185	35	4	FALSE	CDK6
1.977346278	0.002836838	0.505728314	0.4456328	34	3	FALSE	DPP4
2.048543689	5.31E-04	0.488151659	0.6096257	34	4	FALSE	RAC1
2	0.001059499	0.5	0.4777184	34	3	FALSE	NCOA2
1.948220065	0.001648083	0.513289037	0.6399287	34	3	FALSE	MIF
2.009708738	2.82E-04	0.497584541	0.6755793	34	3	FALSE	CHUK
2	0.001296327	0.5	0.6648841	34	4	FALSE	ALOX5
2.197411003	0.005931791	0.455081001	0.2916667	33	4	FALSE	TPI1
2.003236246	6.45E-04	0.499192246	0.6231061	33	3	FALSE	CYP19A1
1.996763754	0.002724389	0.500810373	0.5890152	33	3	FALSE	PLA2G1B
2.012944984	0.001636214	0.496784566	0.4274194	32	3	FALSE	ADRB2
2.064724919	3.75E-04	0.484326019	0.7157258	32	4	FALSE	CCND2
2.132686084	8.92E-04	0.468892261	0.5866935	32	4	FALSE	TOP2A
2.132686084	2.96E-04	0.468892261	0.7182796	31	4	FALSE	E2F1
2.106796117	0.001526104	0.474654378	0.3913978	31	4	FALSE	ADRA2A
2.216828479	0.003736821	0.451094891	0.3204301	31	4	FALSE	LDHB
2.003236246	4.41E-04	0.499192246	0.6537634	31	4	FALSE	PTPN1
2.042071197	9.59E-04	0.489698891	0.6430108	31	4	FALSE	MMP8
2.161812298	1.84E-04	0.46257485	0.7892473	31	4	FALSE	CHEK2
2.165048544	3.13E-04	0.461883408	0.7655914	31	4	FALSE	THBD
2.132686084	0.004242496	0.468892261	0.2896552	30	4	FALSE	TXNRD1
2.077669903	3.34E-04	0.481308411	0.6229885	30	4	FALSE	BCL2
2.155339806	1.70E-04	0.463963964	0.7980296	29	4	FALSE	BIRC5
2.074433657	3.42E-04	0.482059282	0.7536946	29	3	FALSE	PLAT
1.996763754	0.001109171	0.500810373	0.5026455	28	3	FALSE	UCP2
1.996763754	0.002820624	0.500810373	0.3994709	28	4	FALSE	HK2
2.077669903	9.50E-04	0.481308411	0.4656085	28	4	FALSE	FABP1
2.019417476	4.90E-04	0.495192308	0.5820106	28	4	FALSE	BACE1
2.061488673	7.14E-04	0.485086342	0.462963	28	4	FALSE	PRKCE
2.177993528	0.002066558	0.459138187	0.4045584	27	4	FALSE	ME1
2.087378641	9.74E-04	0.479069767	0.3960114	27	4	FALSE	CD36
2.03236246	0.010390073	0.492038217	0.2051282	27	4	FALSE	MGAM
2.122977346	9.57E-04	0.471036585	0.3988604	27	4	FALSE	ADRA2B
1.996763754	0.004000149	0.500810373	0.4558405	27	4	FALSE	ODC1
2.045307443	8.07E-04	0.488924051	0.5185185	27	4	FALSE	LRP1

2.012944984	5.38E-04	0.496784566	0.8262108	27	3	FALSE	ERBB3
2.129449838	8.14E-04	0.469604863	0.5099715	27	4	FALSE	PTPN6
2.174757282	0.002969601	0.459821429	0.4102564	27	4	FALSE	FH
2.1197411	0.002036609	0.471755725	0.3969231	26	3	FALSE	CCK
2.171521036	9.80E-04	0.460506706	0.3876923	26	4	FALSE	PRKCG
2.226537217	3.17E-04	0.449127907	0.6923077	26	4	FALSE	C5AR1
2.100323625	0.002272152	0.476117103	0.3938462	26	4	FALSE	GOT1
2.093851133	0.003017452	0.477588872	0.4553846	26	4	FALSE	IDH2
2.223300971	7.25E-04	0.449781659	0.6615385	26	4	FALSE	PCNA
2.058252427	0.003017597	0.485849057	0.45	25	3	FALSE	HMGCR
2	0.00661129	0.5	0.3066667	25	4	FALSE	AKR1B1
2.1197411	2.70E-04	0.471755725	0.6533333	25	4	FALSE	OLR1
2.071197411	0.001534784	0.4828125	0.4333333	25	4	FALSE	MME
2.1197411	9.28E-04	0.471755725	0.7137681	24	4	FALSE	TOP1
2.042071197	0.002523851	0.489698891	0.2898551	24	3	FALSE	BCHE
2.462783172	0.001609107	0.406044678	0.4818841	24	4	FALSE	GLUD1
2.113268608	1.72E-04	0.473200613	0.6884058	24	4	FALSE	ATF2
2.148867314	0.001987467	0.465361446	0.3985507	24	4	FALSE	GRIN2A
2.045307443	0.002035498	0.488924051	0.3952569	23	4	FALSE	SLC2A2
2.064724919	0.001055277	0.484326019	0.4466403	23	3	FALSE	NR1I2
2.074433657	0.002315355	0.482059282	0.4782609	23	4	FALSE	ACACA
2.077669903	0.002032129	0.481308411	0.5573123	23	3	FALSE	SCD
2.122977346	6.83E-04	0.471036585	0.4664032	23	4	FALSE	PON1
2.174757282	0.001492134	0.459821429	0.4347826	23	4	FALSE	SLC6A4
2.139158576	0.001800497	0.467473525	0.4110672	23	4	FALSE	HTR2A
2.165048544	8.06E-04	0.461883408	0.5059289	23	4	FALSE	PTGS1
2.148867314	3.81E-04	0.465361446	0.5238095	22	4	FALSE	CYP1B1
2.029126214	0.001841501	0.492822967	0.3463203	22	4	FALSE	TYR
2.275080906	0.001884844	0.439544808	0.3506494	22	4	FALSE	MAOA
2.087378641	3.59E-04	0.479069767	0.4675325	22	3	FALSE	INSR
2.122977346	0.005498479	0.471036585	0.2121212	22	4	FALSE	TAT
2.537216828	0.003821912	0.394132653	0.4047619	21	4	FALSE	PDHB
2.142394822	0.001898602	0.466767372	0.4666667	21	3	FALSE	PYY
2.029126214	0.00414895	0.492822967	0.3857143	21	4	FALSE	GLS2
2.223300971	6.61E-05	0.449781659	0.8095238	21	4	FALSE	CDC25B
2.15210356	9.92E-04	0.464661654	0.5368421	20	4	FALSE	SLC6A3
2.171521036	6.62E-04	0.460506706	0.4947368	20	4	FALSE	ADRA1D
2.433656958	0.001386823	0.410904255	0.4263158	20	3	FALSE	SDHA
2.15210356	4.79E-04	0.464661654	0.4947368	20	4	FALSE	MAP2
2.145631068	4.36E-04	0.466063348	0.5368421	20	4	FALSE	PTGER3
2.213592233	1.05E-04	0.451754386	0.7473684	20	4	FALSE	BAX
2.190938511	5.48E-05	0.456425406	0.8736842	20	4	FALSE	E2F2
2.155339806	6.90E-04	0.463963964	0.5263158	19	4	FALSE	DRD1
2.058252427	0.001081385	0.485849057	0.5555556	19	4	FALSE	ACTA2
2.245954693	8.18E-04	0.445244957	0.3625731	19	4	FALSE	TBXA2R
2.300970874	0.001384176	0.434599156	0.3450292	19	4	FALSE	OAT
2.203883495	3.68E-05	0.453744493	0.7953216	19	4	FALSE	RASSF1
2.200647249	0.003579543	0.454411765	0.2865497	19	4	FALSE	CTH
2.177993528	9.72E-05	0.459138187	0.8479532	19	4	FALSE	MMP12
2.210355987	6.12E-04	0.452415813	0.5847953	19	4	FALSE	SLPI
2.190938511	0.001076155	0.456425406	0.3594771	18	4	FALSE	CHRM1
2.139158576	2.58E-04	0.467473525	0.5751634	18	4	FALSE	ADRB1
2.275080906	0.001827812	0.439544808	0.3267974	18	3	FALSE	ALDH2
2.28802589	5.61E-04	0.437057992	0.4509804	18	4	FALSE	GRIA2
2.372168285	0.006447928	0.421555252	0.2279412	17	4	FALSE	AGXT
2.161812298	5.14E-04	0.46257485	0.4264706	17	4	FALSE	GSTM1
2.300970874	0.006652805	0.434599156	0.5294118	17	4	FALSE	COL3A1
2.300970874	0.001010985	0.434599156	0.4264706	17	4	FALSE	CHRM2

2.355987055	0.002363557	0.424450549	0.3088235	17	4	FALSE	OTC
2.13592233	8.13E-04	0.468181818	0.4191176	17	4	FALSE	PPARD
2.27184466	0.001109883	0.44017094	0.425	16	4	FALSE	MAOB
2.200647249	0.002712533	0.454411765	0.15	16	3	FALSE	GUSB
2.132686084	3.25E-04	0.468892261	0.6083333	16	4	FALSE	UCP3
2.110032362	5.99E-04	0.47392638	0.5166667	16	3	FALSE	AKR1C3
2.145631068	5.13E-05	0.466063348	0.7416667	16	4	FALSE	HSF1
2.090614887	4.99E-06	0.478328173	0.95	16	4	FALSE	FLT4
2.236245955	8.09E-04	0.447178003	0.2571429	15	4	FALSE	ABCC2
2.181229773	0.001027083	0.458456973	0.4761905	15	3	FALSE	F7
2.216828479	1.10E-04	0.451094891	0.6190476	15	4	FALSE	GAP43
2.223300971	0.001559245	0.449781659	0.3238095	15	4	FALSE	ALDH1B1
2.110032362	7.20E-05	0.47392638	0.6285714	15	3	FALSE	NR3C2
2.236245955	0.001400216	0.447178003	0.5142857	15	4	FALSE	ME2
2.249190939	1.69E-04	0.444604317	0.4857143	15	4	FALSE	PSMD3
2.100323625	1.82E-04	0.476117103	0.5333333	15	3	FALSE	GSTM2
2.618122977	7.83E-04	0.381953028	0.4835165	14	4	FALSE	ALDH4A1
2.323624595	1.98E-04	0.430362117	0.7032967	14	4	FALSE	TK1
2.352750809	1.96E-04	0.425034388	0.7820513	13	4	FALSE	DEFB4A
2.336569579	1.07E-04	0.427977839	0.530303	12	4	FALSE	NR1I3
2.57605178	1.18E-04	0.388190955	0.6969697	12	4	FALSE	ME3
2.404530744	5.15E-04	0.415881561	0.3181818	12	4	FALSE	LTA4H
2.25566343	3.40E-04	0.443328551	0.6060606	12	4	FALSE	LCAT
2.242718447	0.00110536	0.445887446	0.4	11	4	FALSE	AOC3
2.498381877	9.20E-05	0.400259067	0.6363636	11	4	FALSE	F10
2.372168285	3.03E-04	0.421555252	0.4545455	11	3	FALSE	F13A1
2.207119741	7.13E-04	0.453079179	0.6444444	10	4	FALSE	ABI1
2.233009709	2.05E-04	0.447826087	0.5555556	10	4	FALSE	TF
2.220064725	9.57E-04	0.450437318	0.4	10	4	FALSE	AHSA1
2.275080906	5.65E-06	0.439544808	0.9111111	10	4	FALSE	BAK1
2.71197411	1.20E-04	0.368735084	0.6	10	5	FALSE	ALDH5A1
2.307443366	2.45E-05	0.433380084	0.7777778	10	4	FALSE	CETP
2.433656958	1.41E-04	0.410904255	0.5333333	10	4	FALSE	LTF
2.281553398	2.85E-04	0.438297872	0.5	9	4	FALSE	XDH
2.508090615	0.001043461	0.398709677	0.2777778	9	4	FALSE	BHMT
2.563106796	4.10E-04	0.390151515	0.4444444	9	4	FALSE	GLRA2
2.517799353	1.85E-04	0.397172237	0.8055556	9	4	FALSE	CHRM3
2.346278317	4.46E-04	0.426206897	0.3333333	9	4	FALSE	GLB1
2.453074434	3.89E-04	0.407651715	0.4166667	9	4	FALSE	GRIK2
2.595469256	0.001092257	0.385286783	0.1944444	9	4	FALSE	CHDH
2.433656958	6.40E-04	0.410904255	0.3333333	9	4	FALSE	PYGM
2.378640777	1.39E-05	0.420408163	0.6944444	9	4	FALSE	ELK1
2.605177994	0.002642507	0.383850932	0.25	8	4	FALSE	GNMT
2.59223301	6.00E-04	0.38576779	0.1785714	8	4	FALSE	KYNU
2.783171521	1.13E-04	0.359302326	0.3571429	8	4	FALSE	CHRNA7
2.265372168	2.27E-04	0.441428571	0.6785714	8	4	FALSE	BCAT2
2.469255663	2.23E-06	0.404980341	0.8928571	8	4	FALSE	FCER2
2.378640777	3.22E-04	0.420408163	0.5714286	8	4	FALSE	NDUFS1
2.521035599	2.75E-04	0.396662388	0.2142857	8	4	FALSE	ENPEP
2.249190939	7.23E-05	0.444604317	0.5357143	8	4	FALSE	TPO
2.747572816	1.87E-04	0.363957597	0.2857143	7	4	FALSE	ALDH9A1
2.28802589	3.05E-05	0.437057992	0.6190476	7	4	FALSE	PRSS1
2.414239482	2.50E-05	0.414209115	0.7619048	7	4	FALSE	HAS2
2.265372168	1.22E-05	0.441428571	0.6666667	7	4	FALSE	TYRP1
2.766990291	2.18E-04	0.361403509	0.2666667	6	4	FALSE	ACAT2
2.72815534	3.01E-04	0.366548043	0.0666667	6	4	FALSE	GALE
2.414239482	3.94E-05	0.414209115	0.3333333	6	4	FALSE	RBP2
2.388349515	2.03E-04	0.418699187	0.3333333	6	4	FALSE	PLA2G2E

2.899676375	9.49E-05	0.344866071	0.6666667	6	5	FALSE	MTR
2.411003236	1.37E-04	0.414765101	0.4666667	6	4	FALSE	KCNH2
2.278317152	9.20E-06	0.438920455	0.8	6	4	FALSE	PDX1
2.336569579	5.43E-04	0.427977839	0.2666667	6	4	FALSE	NPEPPS
2.262135922	8.01E-05	0.442060086	0.4	5	3	FALSE	CA2
2.644012945	7.99E-05	0.378212974	0.3	5	4	FALSE	SCN5A
2.372168285	0.003908396	0.421555252	0.6	5	4	FALSE	SOAT1
2.42394822	2.79E-05	0.412550067	0.5	4	4	FALSE	ATP2C1
2.411003236	1.16E-05	0.414765101	0.6666667	4	4	FALSE	SMPD2
2.517799353	1.02E-04	0.397172237	0.5	4	4	FALSE	CLDN4
2.44012945	0	0.409814324	1	4	4	FALSE	LITAF
2.472491909	1.30E-05	0.404450262	0.5	4	4	FALSE	IVL
2.44012945	2.48E-05	0.409814324	0.1666667	4	4	FALSE	PAM
2.757281553	6.88E-06	0.362676056	0.6666667	3	4	FALSE	SLC25A13
2.734627832	3.81E-04	0.365680473	0.6666667	3	4	FALSE	PCOLCE
2.786407767	0	0.358885017	1	3	4	FALSE	SLC16A7
3.216828479	0	0.310865191	1	2	5	FALSE	GCAT
2.401294498	0	0.416442049	1	2	4	FALSE	NQO2
3.294498382	0	0.303536346	1	2	5	FALSE	PLOD1
2.708737864	3.06E-05	0.369175627	0	2	4	FALSE	EIF6
3.158576052	6.40E-05	0.316598361	0	2	5	FALSE	SLC25A10
3.029126214	0	0.330128205	0	1	5	FALSE	CA1
2.498381877	0	0.400259067	0	1	4	FALSE	ISYNA1
